# Supplementary material for: Genomic Insights into Tumorigenesis in Newly Diagnosed Multiple Myeloma
Source: Diagnostics (Basel). 2025 Aug 23;15(17):2130. doi: 10.3390/diagnostics15172130 (PMC12428068; doi:10.3390/diagnostics15172130)
Supplement: Supplementary file 1 [file diagnostics-15-02130-s001.zip › Supplementary Figure S1.pdf]

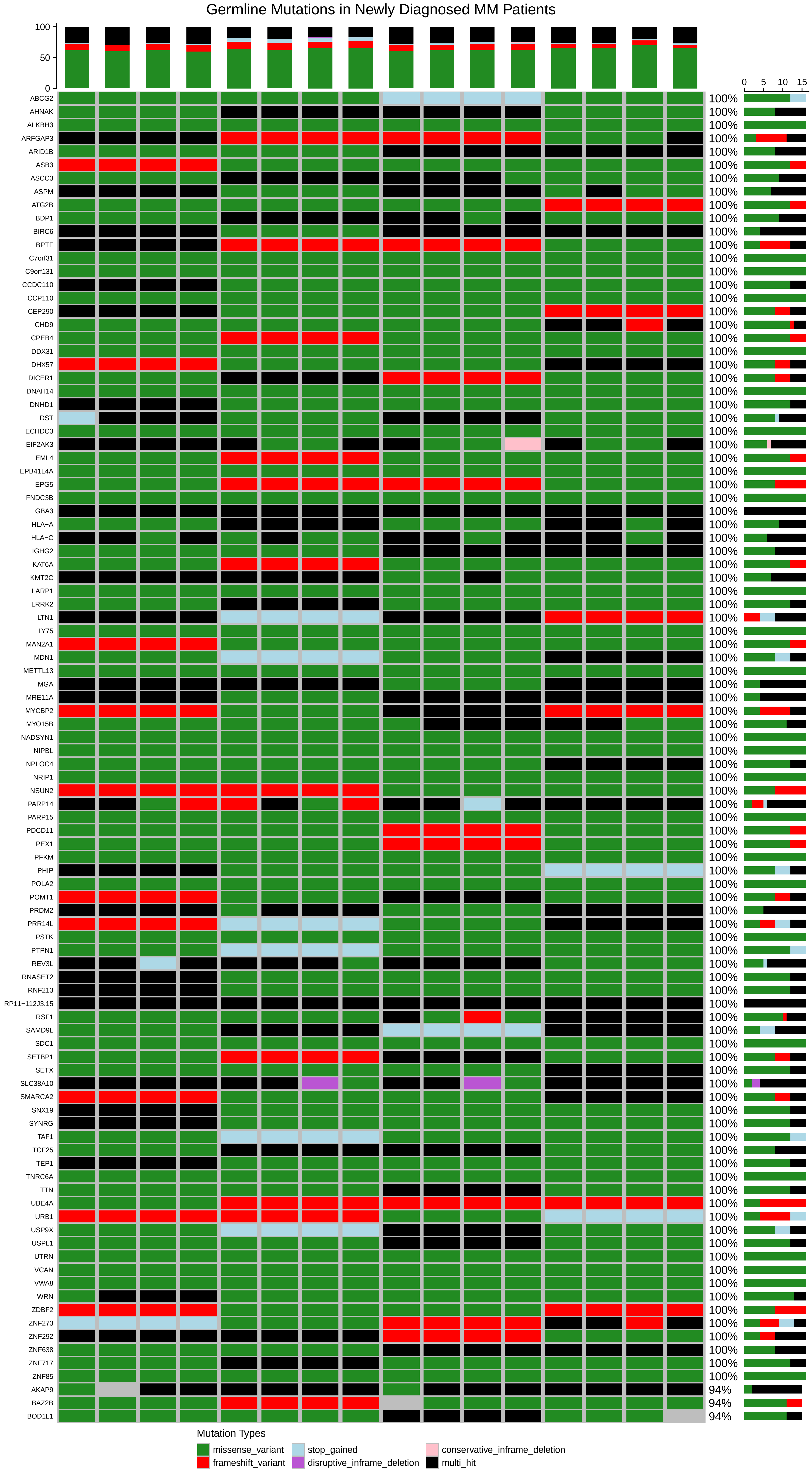

Germline Mutations in Newly Diagnosed MM Patients
100
50
0
0
5
10
15
ABCG2
AHNAK
ALKBH3
ARFGAP3
ARID1B
ASB3
ASCC3
ASPM
ATG2B
BDP1
BIRC6
BPTF
C7orf31
C9orf131
CCDC110
CCP110
CEP290
CHD9
CPEB4
DDX31
DHX57
DICER1
DNAH14
DNHD1
DST
ECHDC3
EIF2AK3
EML4
EPB41L4A
EPG5
FNDC3B
GBA3
HLA−A
HLA−C
IGHG2
KAT6A
KMT2C
LARP1
LRRK2
LTN1
LY75
MAN2A1
MDN1
METTL13
MGA
MRE11A
MYCBP2
MYO15B
NADSYN1
NIPBL
NPLOC4
NRIP1
NSUN2
PARP14
PARP15
PDCD11
PEX1
PFKM
PHIP
POLA2
POMT1
PRDM2
PRR14L
PSTK
PTPN1
REV3L
RNASET2
RNF213
RP11−112J3.15
RSF1
SAMD9L
SDC1
SETBP1
SETX
SLC38A10
SMARCA2
SNX19
SYNRG
TAF1
TCF25
TEP1
TNRC6A
TTN
UBE4A
URB1
USP9X
USPL1
UTRN
VCAN
VWA8
WRN
ZDBF2
ZNF273
ZNF292
ZNF638
ZNF717
ZNF85
AKAP9
BAZ2B
BOD1L1
100% (ABCG2 through ZNF85)
94% (AKAP9, BAZ2B, BOD1L1)
Mutation Types
missense_variant
frameshift_variant
stop_gained
disruptive_inframe_deletion
conservative_inframe_deletion
multi_hit

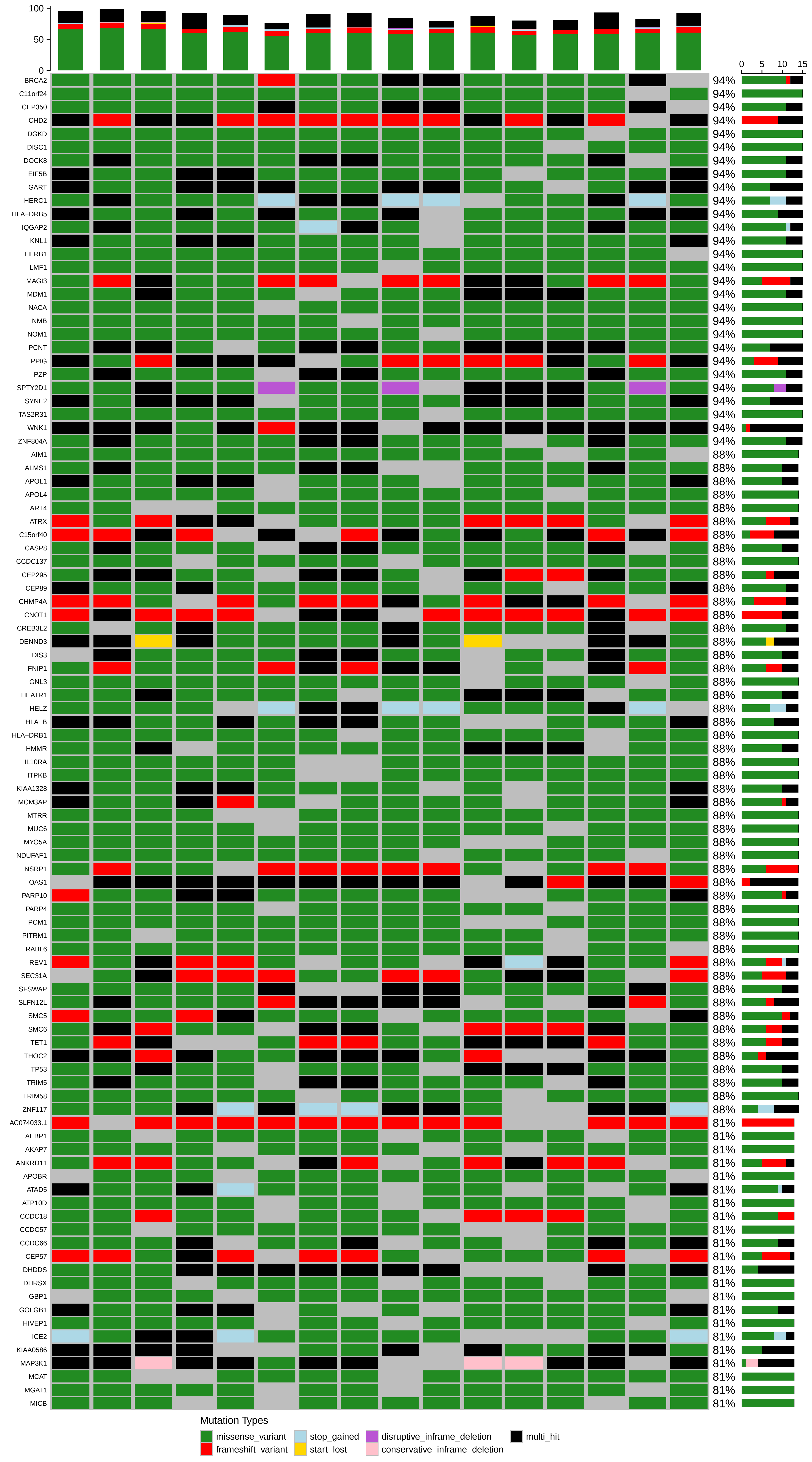
100
50
0
0 5 10 15
BRCA2 94%
C11orf24 94%
CEP350 94%
CHD2 94%
DGKD 94%
DISC1 94%
DOCK8 94%
EIF5B 94%
GART 94%
HERC1 94%
HLA-DRB5 94%
IQGAP2 94%
KNL1 94%
LILRB1 94%
LMF1 94%
MAGI3 94%
MDM1 94%
NACA 94%
NMB 94%
NOM1 94%
PCNT 94%
PPIG 94%
PZP 94%
SPTY2D1 94%
SYNE2 94%
TAS2R31 94%
WNK1 94%
ZNF804A 94%
AIM1 88%
ALMS1 88%
APOL1 88%
APOL4 88%
ART4 88%
ATRX 88%
C15orf40 88%
CASP8 88%
CCDC137 88%
CEP295 88%
CEP89 88%
CHMP4A 88%
CNOT1 88%
CREB3L2 88%
DENND3 88%
DIS3 88%
FNIP1 88%
GNL3 88%
HEATR1 88%
HELZ 88%
HLA-B 88%
HLA-DRB1 88%
HMMR 88%
IL10RA 88%
ITPKB 88%
KIAA1328 88%
MCM3AP 88%
MTRR 88%
MUC6 88%
MYO5A 88%
NDUFAF1 88%
NSRP1 88%
OAS1 88%
PARP10 88%
PARP4 88%
PCM1 88%
PITRM1 88%
RABL6 88%
REV1 88%
SEC31A 88%
SFSWAP 88%
SLFN12L 88%
SMC5 88%
SMC6 88%
TET1 88%
THOC2 88%
TP53 88%
TRIM5 88%
TRIM58 88%
ZNF117 88%
AC074033.1 81%
AEBP1 81%
AKAP7 81%
ANKRD11 81%
APOBR 81%
ATAD5 81%
ATP10D 81%
CCDC18 81%
CCDC57 81%
CCDC66 81%
CEP57 81%
DHDDS 81%
DHRSX 81%
GBP1 81%
GOLGB1 81%
HIVEP1 81%
ICE2 81%
KIAA0586 81%
MAP3K1 81%
MCAT 81%
MGAT1 81%
MICB 81%
Mutation Types
missense_variant
frameshift_variant
stop_gained
start_lost
disruptive_inframe_deletion
conservative_inframe_deletion
multi_hit

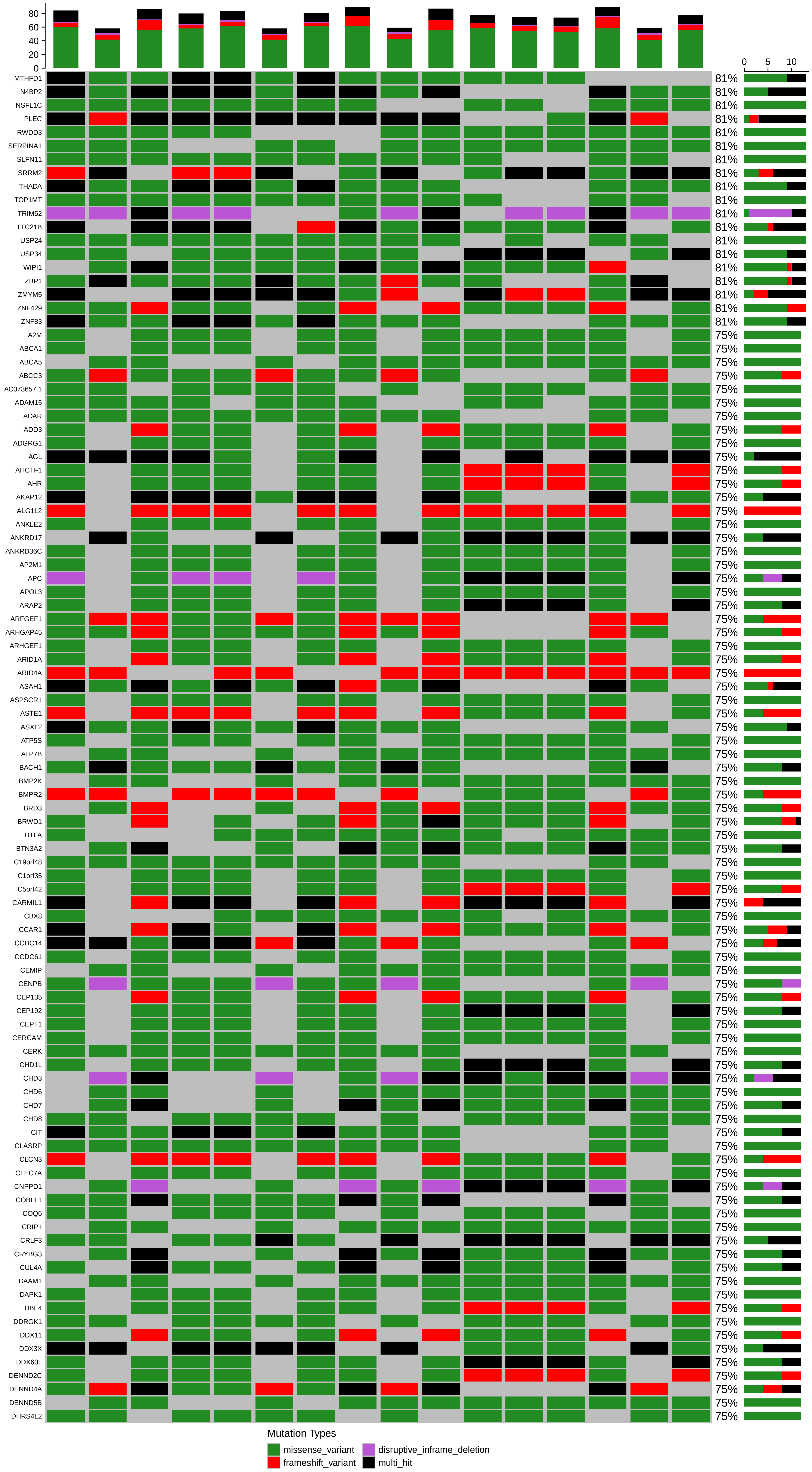
80
60
40
20
0
0
5
10
MTHFD1 81%
N4BP2 81%
NSFL1C 81%
PLEC 81%
RWDD3 81%
SERPINA1 81%
SLFN11 81%
SRRM2 81%
THADA 81%
TOP1MT 81%
TRIM52 81%
TTC21B 81%
USP24 81%
USP34 81%
WIPI1 81%
ZBP1 81%
ZMYM5 81%
ZNF429 81%
ZNF83 81%
A2M 75%
ABCA1 75%
ABCA5 75%
ABCC3 75%
AC073657.1 75%
ADAM15 75%
ADAR 75%
ADD3 75%
ADGRG1 75%
AGL 75%
AHCTF1 75%
AHR 75%
AKAP12 75%
ALG1L2 75%
ANKLE2 75%
ANKRD17 75%
ANKRD36C 75%
AP2M1 75%
APC 75%
APOL3 75%
ARAP2 75%
ARFGEF1 75%
ARHGAP45 75%
ARHGEF1 75%
ARID1A 75%
ARID4A 75%
ASAH1 75%
ASPSCR1 75%
ASTE1 75%
ASXL2 75%
ATP5S 75%
ATP7B 75%
BACH1 75%
BMP2K 75%
BMPR2 75%
BRD3 75%
BRWD1 75%
BTLA 75%
BTN3A2 75%
C19orf48 75%
C1orf35 75%
C5orf42 75%
CARMIL1 75%
CBX8 75%
CCAR1 75%
CCDC14 75%
CCDC61 75%
CEMIP 75%
CENPB 75%
CEP135 75%
CEP192 75%
CEPT1 75%
CERCAM 75%
CERK 75%
CHD1L 75%
CHD3 75%
CHD6 75%
CHD7 75%
CHD8 75%
CIT 75%
CLASRP 75%
CLCN3 75%
CLEC7A 75%
CNPPD1 75%
COBLL1 75%
COQ6 75%
CRIP1 75%
CRLF3 75%
CRYBG3 75%
CUL4A 75%
DAAM1 75%
DAPK1 75%
DBF4 75%
DDRGK1 75%
DDX11 75%
DDX3X 75%
DDX60L 75%
DENND2C 75%
DENND4A 75%
DENND5B 75%
DHRS4L2 75%
Mutation Types
missense_variant
frameshift_variant
disruptive_inframe_deletion
multi_hit

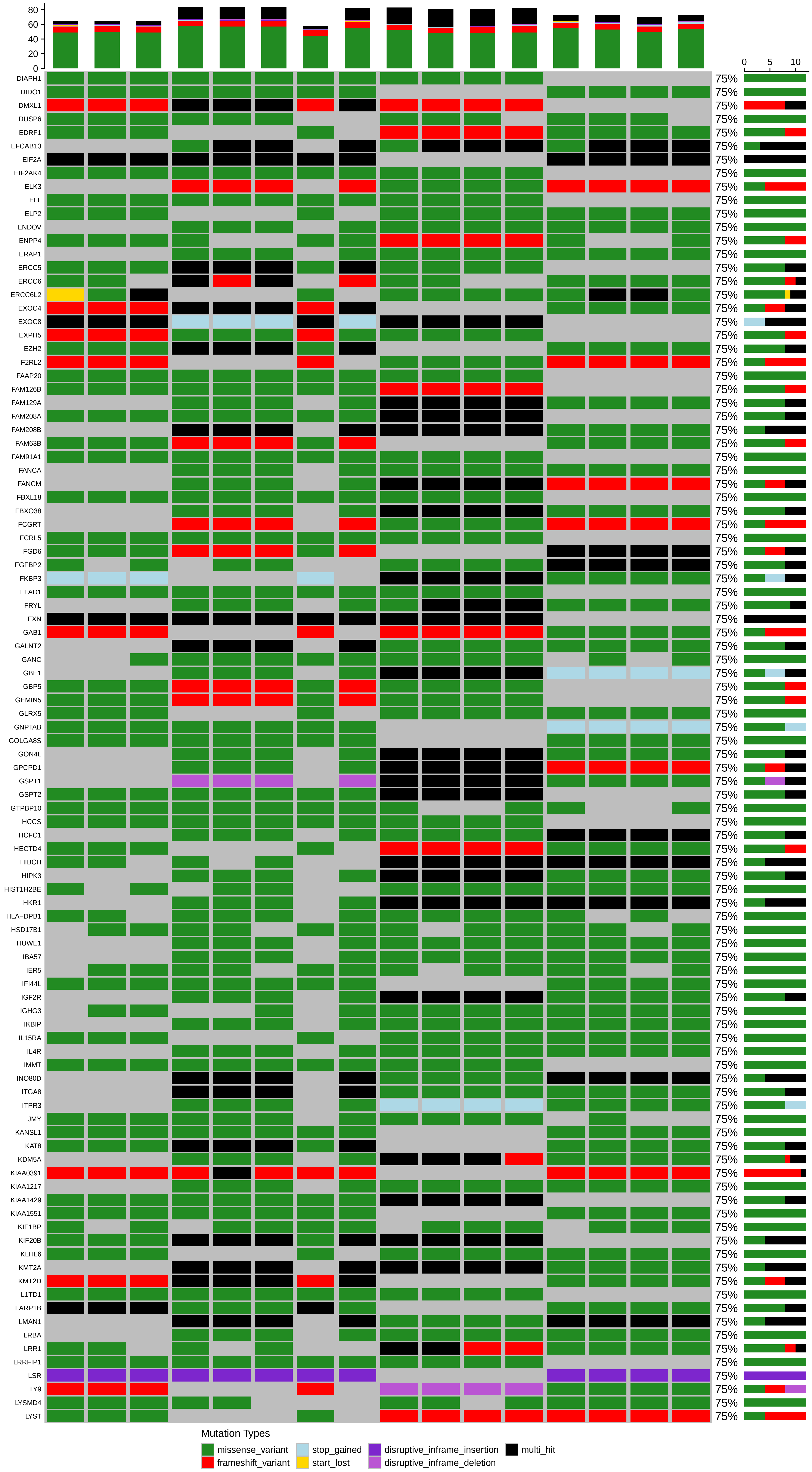

80
60
40
20
0
0
5
10
DIAPH1
DIDO1
DMXL1
DUSP6
EDRF1
EFCAB13
EIF2A
EIF2AK4
ELK3
ELL
ELP2
ENDOV
ENPP4
ERAP1
ERCC5
ERCC6
ERCC6L2
EXOC4
EXOC8
EXPH5
EZH2
F2RL2
FAAP20
FAM126B
FAM129A
FAM208A
FAM208B
FAM63B
FAM91A1
FANCA
FANCM
FBXL18
FBXO38
FCGRT
FCRL5
FGD6
FGFBP2
FKBP3
FLAD1
FRYL
FXN
GAB1
GALNT2
GANC
GBE1
GBP5
GEMIN5
GLRX5
GNPTAB
GOLGA8S
GON4L
GPCPD1
GSPT1
GSPT2
GTPBP10
HCCS
HCFC1
HECTD4
HIBCH
HIPK3
HIST1H2BE
HKR1
HLA−DPB1
HSD17B1
HUWE1
IBA57
IER5
IFI44L
IGF2R
IGHG3
IKBIP
IL15RA
IL4R
IMMT
INO80D
ITGA8
ITPR3
JMY
KANSL1
KAT8
KDM5A
KIAA0391
KIAA1217
KIAA1429
KIAA1551
KIF1BP
KIF20B
KLHL6
KMT2A
KMT2D
L1TD1
LARP1B
LMAN1
LRBA
LRR1
LRRFIP1
LSR
LY9
LYSMD4
LYST
75%
Mutation Types
missense_variant
frameshift_variant
stop_gained
start_lost
disruptive_inframe_insertion
disruptive_inframe_deletion
multi_hit

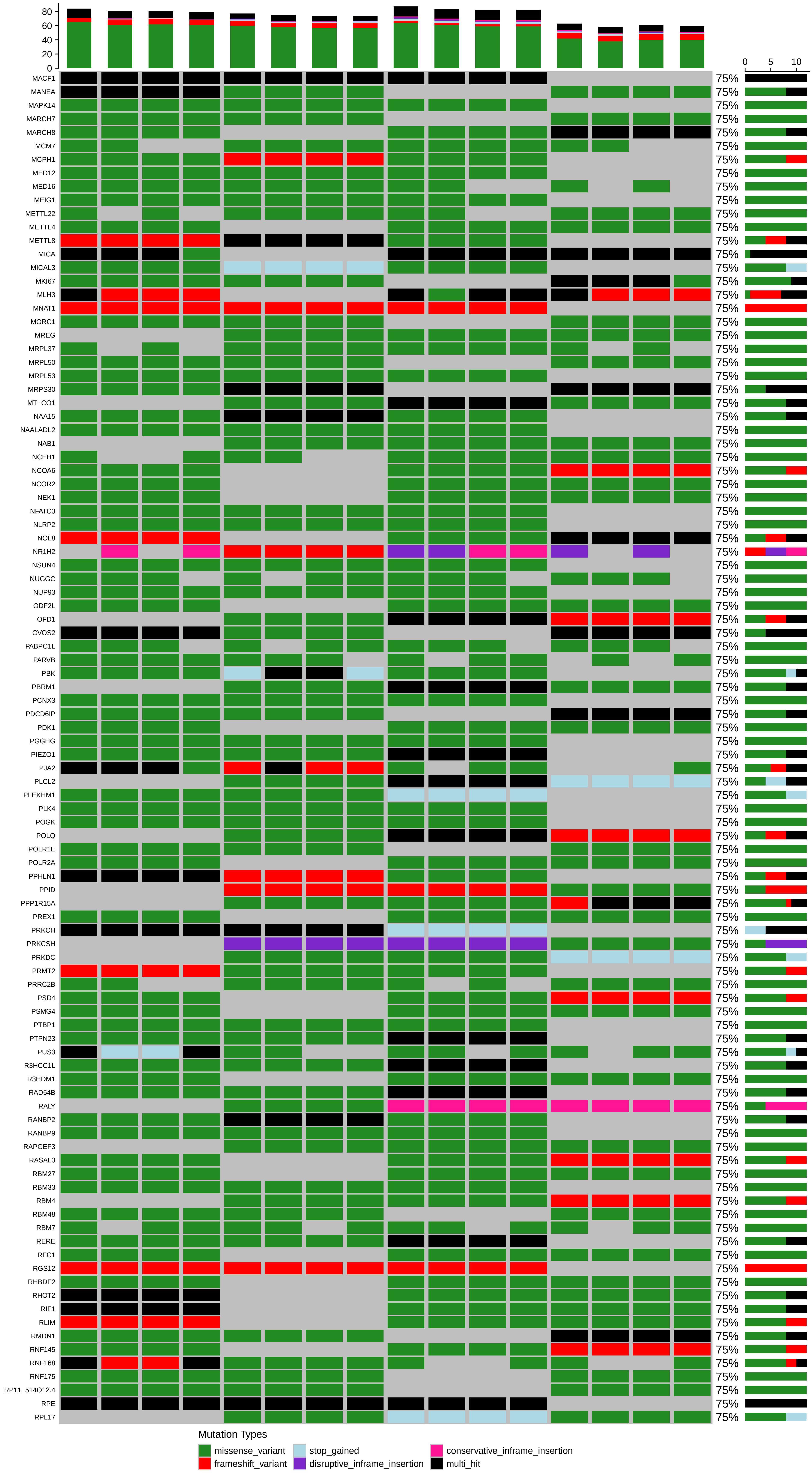
80
60
40
20
0
0
5
10
MACF1
MANEA
MAPK14
MARCH7
MARCH8
MCM7
MCPH1
MED12
MED16
MEIG1
METTL22
METTL4
METTL8
MICA
MICAL3
MKI67
MLH3
MNAT1
MORC1
MREG
MRPL37
MRPL50
MRPL53
MRPS30
MT−CO1
NAA15
NAALADL2
NAB1
NCEH1
NCOA6
NCOR2
NEK1
NFATC3
NLRP2
NOL8
NR1H2
NSUN4
NUGGC
NUP93
ODF2L
OFD1
OVOS2
PABPC1L
PARVB
PBK
PBRM1
PCNX3
PDCD6IP
PDK1
PGGHG
PIEZO1
PJA2
PLCL2
PLEKHM1
PLK4
POGK
POLQ
POLR1E
POLR2A
PPHLN1
PPID
PPP1R15A
PREX1
PRKCH
PRKCSH
PRKDC
PRMT2
PRRC2B
PSD4
PSMG4
PTBP1
PTPN23
PUS3
R3HCC1L
R3HDM1
RAD54B
RALY
RANBP2
RANBP9
RAPGEF3
RASAL3
RBM27
RBM33
RBM4
RBM48
RBM7
RERE
RFC1
RGS12
RHBDF2
RHOT2
RIF1
RLIM
RMDN1
RNF145
RNF168
RNF175
RP11−514O12.4
RPE
RPL17
75%
Mutation Types
missense_variant
frameshift_variant
stop_gained
disruptive_inframe_insertion
conservative_inframe_insertion
multi_hit

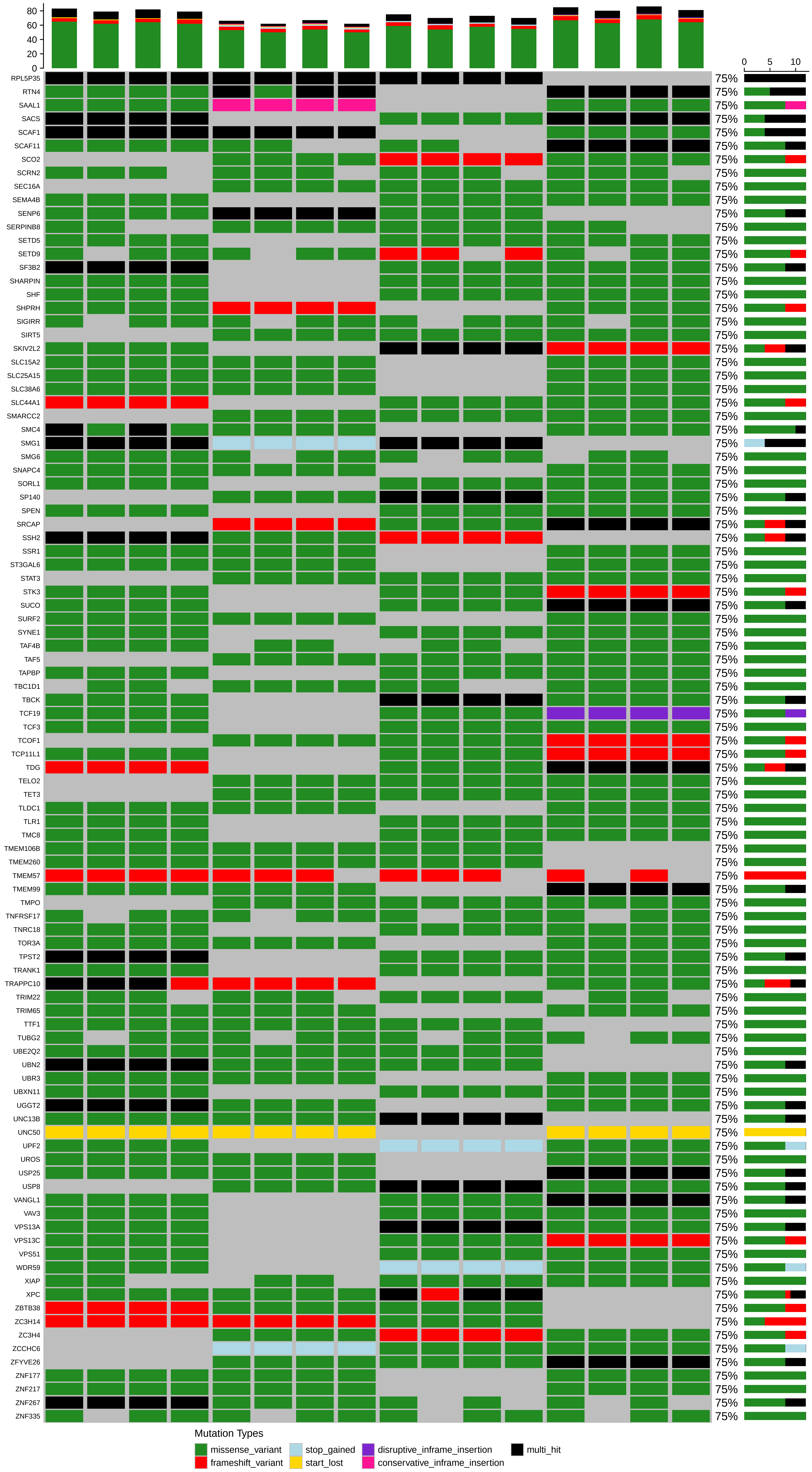

RPL5P35
RTN4
SAAL1
SACS
SCAF1
SCAF11
SCO2
SCRN2
SEC16A
SEMA4B
SENP6
SERPINB8
SETD5
SETD9
SF3B2
SHARPIN
SHF
SHPRH
SIGIRR
SIRT5
SKIV2L2
SLC15A2
SLC25A15
SLC38A6
SLC44A1
SMARCC2
SMC4
SMG1
SMG6
SNAPC4
SORL1
SP140
SPEN
SRCAP
SSH2
SSR1
ST3GAL6
STAT3
STK3
SUCO
SURF2
SYNE1
TAF4B
TAF5
TAPBP
TBC1D1
TBCK
TCF19
TCF3
TCOF1
TCP11L1
TDG
TELO2
TET3
TLDC1
TLR1
TMC8
TMEM106B
TMEM260
TMEM57
TMEM99
TMPO
TNFRSF17
TNRC18
TOR3A
TPST2
TRANK1
TRAPPC10
TRIM22
TRIM65
TTF1
TUBG2
UBE2Q2
UBN2
UBR3
UBXN11
UGGT2
UNC13B
UNC50
UPF2
UROS
USP25
USP8
VANGL1
VAV3
VPS13A
VPS13C
VPS51
WDR59
XIAP
XPC
ZBTB38
ZC3H14
ZC3H4
ZCCHC6
ZFYVE26
ZNF177
ZNF217
ZNF267
ZNF335
75%
Mutation Types
missense_variant
frameshift_variant
stop_gained
start_lost
disruptive_inframe_insertion
conservative_inframe_insertion
multi_hit

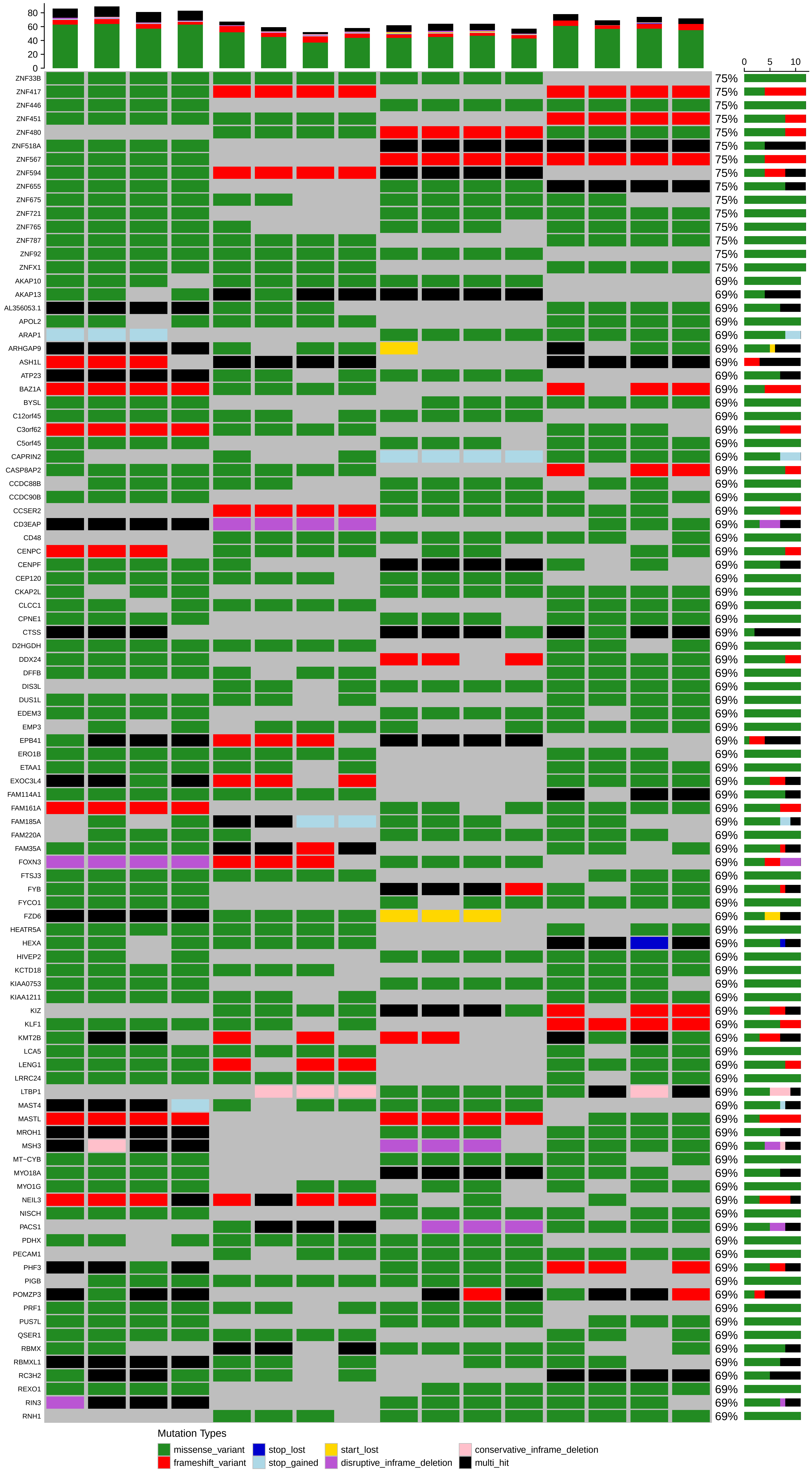

80
60
40
20
0
0
5
10
ZNF33B
ZNF417
ZNF446
ZNF451
ZNF480
ZNF518A
ZNF567
ZNF594
ZNF655
ZNF675
ZNF721
ZNF765
ZNF787
ZNF92
ZNFX1
AKAP10
AKAP13
AL356053.1
APOL2
ARAP1
ARHGAP9
ASH1L
ATP23
BAZ1A
BYSL
C12orf45
C3orf62
C5orf45
CAPRIN2
CASP8AP2
CCDC88B
CCDC90B
CCSER2
CD3EAP
CD48
CENPC
CENPF
CEP120
CKAP2L
CLCC1
CPNE1
CTSS
D2HGDH
DDX24
DFFB
DIS3L
DUS1L
EDEM3
EMP3
EPB41
ERO1B
ETAA1
EXOC3L4
FAM114A1
FAM161A
FAM185A
FAM220A
FAM35A
FOXN3
FTSJ3
FYB
FYCO1
FZD6
HEATR5A
HEXA
HIVEP2
KCTD18
KIAA0753
KIAA1211
KIZ
KLF1
KMT2B
LCA5
LENG1
LRRC24
LTBP1
MAST4
MASTL
MROH1
MSH3
MT−CYB
MYO18A
MYO1G
NEIL3
NISCH
PACS1
PDHX
PECAM1
PHF3
PIGB
POMZP3
PRF1
PUS7L
QSER1
RBMX
RBMXL1
RC3H2
REXO1
RIN3
RNH1
75% (ZNF33B–ZNFX1)
69% (AKAP10–RNH1)
Mutation Types
missense_variant
frameshift_variant
stop_lost
stop_gained
start_lost
disruptive_inframe_deletion
conservative_inframe_deletion
multi_hit

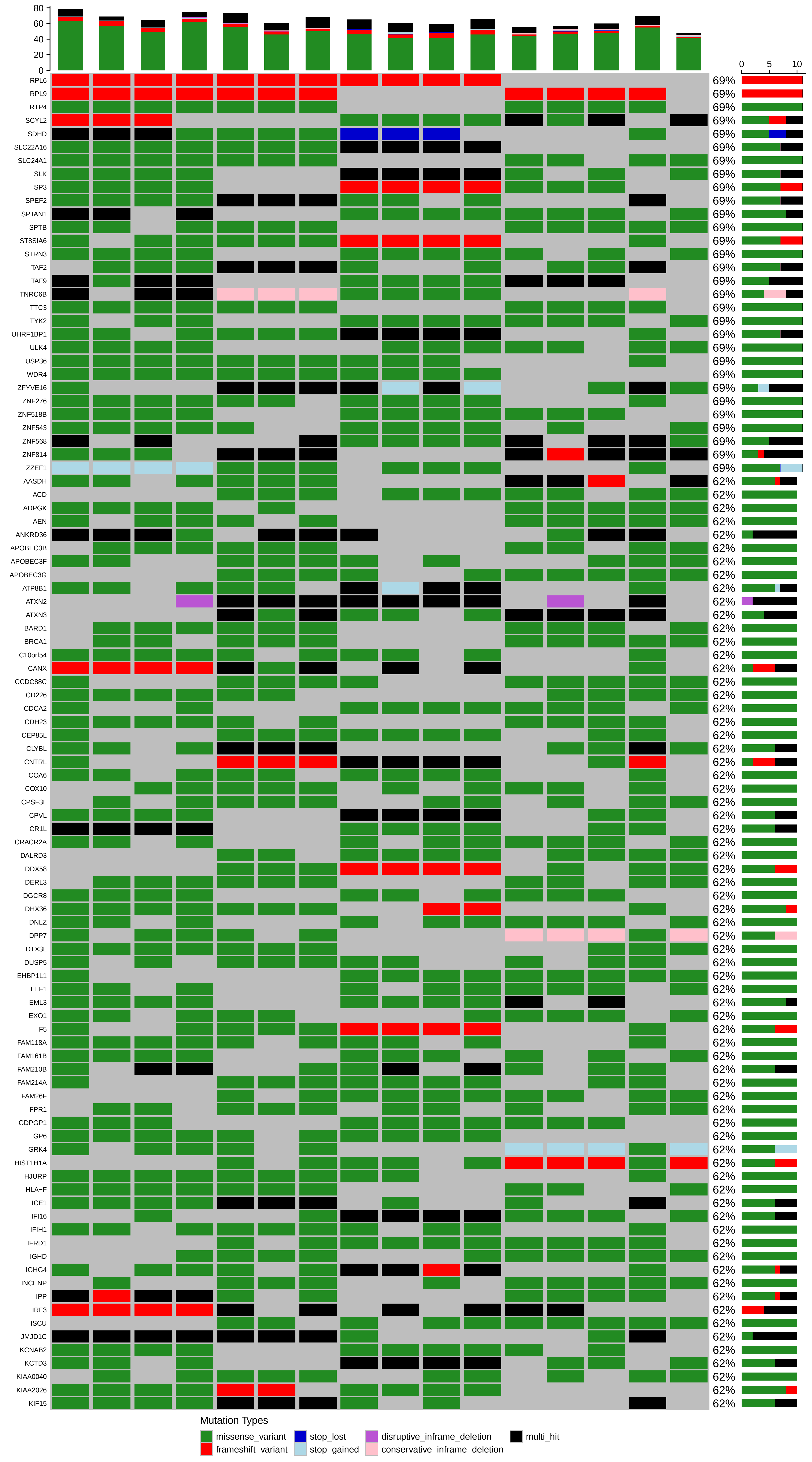

80
60
40
20
0
0
5
10
RPL6 69%
RPL9 69%
RTP4 69%
SCYL2 69%
SDHD 69%
SLC22A16 69%
SLC24A1 69%
SLK 69%
SP3 69%
SPEF2 69%
SPTAN1 69%
SPTB 69%
ST8SIA6 69%
STRN3 69%
TAF2 69%
TAF9 69%
TNRC6B 69%
TTC3 69%
TYK2 69%
UHRF1BP1 69%
ULK4 69%
USP36 69%
WDR4 69%
ZFYVE16 69%
ZNF276 69%
ZNF518B 69%
ZNF543 69%
ZNF568 69%
ZNF814 69%
ZZEF1 69%
AASDH 62%
ACD 62%
ADPGK 62%
AEN 62%
ANKRD36 62%
APOBEC3B 62%
APOBEC3F 62%
APOBEC3G 62%
ATP8B1 62%
ATXN2 62%
ATXN3 62%
BARD1 62%
BRCA1 62%
C10orf54 62%
CANX 62%
CCDC88C 62%
CD226 62%
CDCA2 62%
CDH23 62%
CEP85L 62%
CLYBL 62%
CNTRL 62%
COA6 62%
COX10 62%
CPSF3L 62%
CPVL 62%
CR1L 62%
CRACR2A 62%
DALRD3 62%
DDX58 62%
DERL3 62%
DGCR8 62%
DHX36 62%
DNLZ 62%
DPP7 62%
DTX3L 62%
DUSP5 62%
EHBP1L1 62%
ELF1 62%
EML3 62%
EXO1 62%
F5 62%
FAM118A 62%
FAM161B 62%
FAM210B 62%
FAM214A 62%
FAM26F 62%
FPR1 62%
GDPGP1 62%
GP6 62%
GRK4 62%
HIST1H1A 62%
HJURP 62%
HLA−F 62%
ICE1 62%
IFI16 62%
IFIH1 62%
IFRD1 62%
IGHD 62%
IGHG4 62%
INCENP 62%
IPP 62%
IRF3 62%
ISCU 62%
JMJD1C 62%
KCNAB2 62%
KCTD3 62%
KIAA0040 62%
KIAA2026 62%
KIF15 62%
Mutation Types
missense_variant
frameshift_variant
stop_lost
stop_gained
disruptive_inframe_deletion
conservative_inframe_deletion
multi_hit

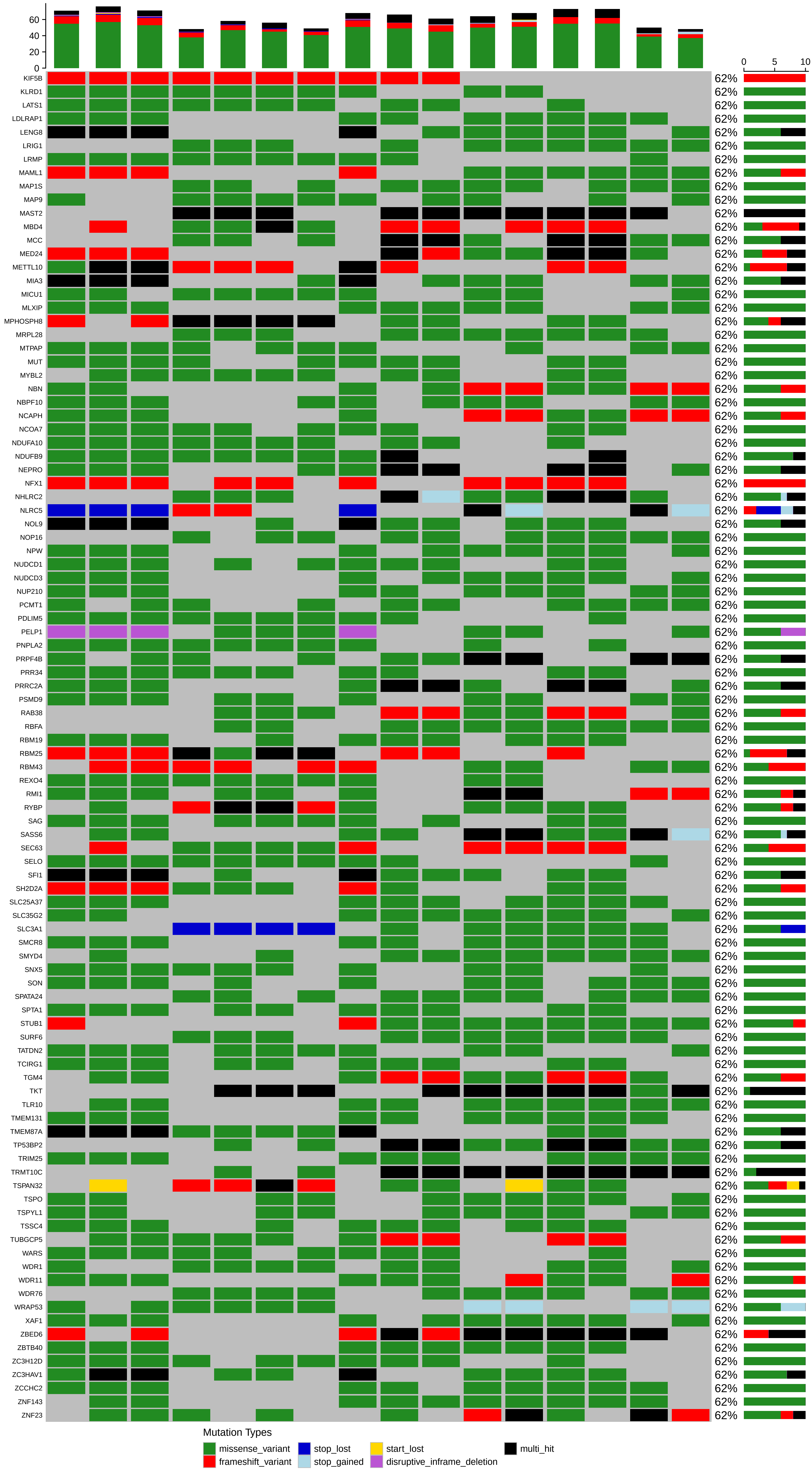

60
40
20
0
0
5
10
KIF5B
KLRD1
LATS1
LDLRAP1
LENG8
LRIG1
LRMP
MAML1
MAP1S
MAP9
MAST2
MBD4
MCC
MED24
METTL10
MIA3
MICU1
MLXIP
MPHOSPH8
MRPL28
MTPAP
MUT
MYBL2
NBN
NBPF10
NCAPH
NCOA7
NDUFA10
NDUFB9
NEPRO
NFX1
NHLRC2
NLRC5
NOL9
NOP16
NPW
NUDCD1
NUDCD3
NUP210
PCMT1
PDLIM5
PELP1
PNPLA2
PRPF4B
PRR34
PRRC2A
PSMD9
RAB38
RBFA
RBM19
RBM25
RBM43
REXO4
RMI1
RYBP
SAG
SASS6
SEC63
SELO
SFI1
SH2D2A
SLC25A37
SLC35G2
SLC3A1
SMCR8
SMYD4
SNX5
SON
SPATA24
SPTA1
STUB1
SURF6
TATDN2
TCIRG1
TGM4
TKT
TLR10
TMEM131
TMEM87A
TP53BP2
TRIM25
TRMT10C
TSPAN32
TSPO
TSPYL1
TSSC4
TUBGCP5
WARS
WDR1
WDR11
WDR76
WRAP53
XAF1
ZBED6
ZBTB40
ZC3H12D
ZC3HAV1
ZCCHC2
ZNF143
ZNF23
62%
Mutation Types
missense_variant
frameshift_variant
stop_lost
stop_gained
start_lost
disruptive_inframe_deletion
multi_hit

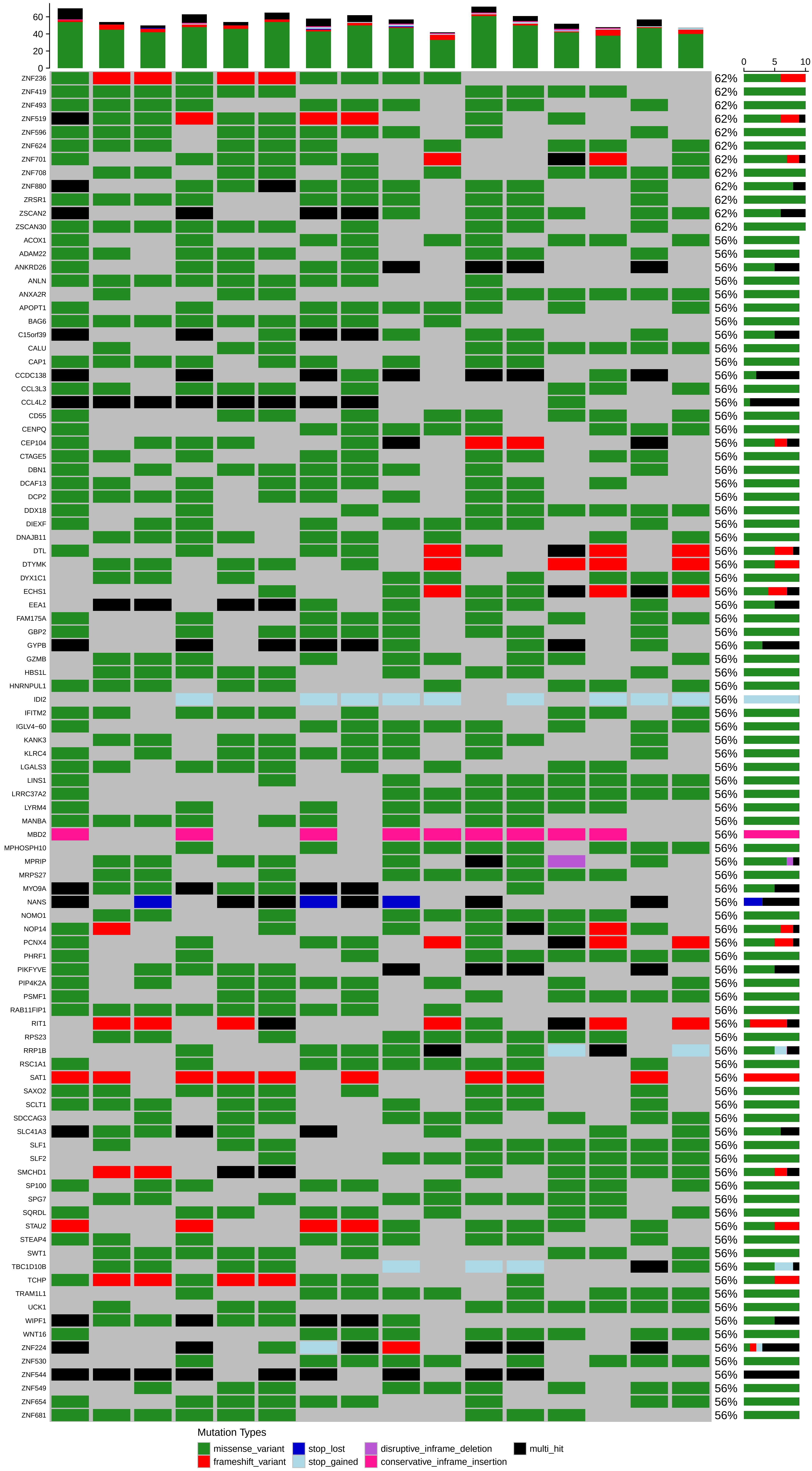

60
40
20
0
0
5
10
ZNF236 62%
ZNF419 62%
ZNF493 62%
ZNF519 62%
ZNF596 62%
ZNF624 62%
ZNF701 62%
ZNF708 62%
ZNF880 62%
ZRSR1 62%
ZSCAN2 62%
ZSCAN30 62%
ACOX1 56%
ADAM22 56%
ANKRD26 56%
ANLN 56%
ANXA2R 56%
APOPT1 56%
BAG6 56%
C15orf39 56%
CALU 56%
CAP1 56%
CCDC138 56%
CCL3L3 56%
CCL4L2 56%
CD55 56%
CENPQ 56%
CEP104 56%
CTAGE5 56%
DBN1 56%
DCAF13 56%
DCP2 56%
DDX18 56%
DIEXF 56%
DNAJB11 56%
DTL 56%
DTYMK 56%
DYX1C1 56%
ECHS1 56%
EEA1 56%
FAM175A 56%
GBP2 56%
GYPB 56%
GZMB 56%
HBS1L 56%
HNRNPUL1 56%
IDI2 56%
IFITM2 56%
IGLV4−60 56%
KANK3 56%
KLRC4 56%
LGALS3 56%
LINS1 56%
LRRC37A2 56%
LYRM4 56%
MANBA 56%
MBD2 56%
MPHOSPH10 56%
MPRIP 56%
MRPS27 56%
MYO9A 56%
NANS 56%
NOMO1 56%
NOP14 56%
PCNX4 56%
PHRF1 56%
PIKFYVE 56%
PIP4K2A 56%
PSMF1 56%
RAB11FIP1 56%
RIT1 56%
RPS23 56%
RRP1B 56%
RSC1A1 56%
SAT1 56%
SAXO2 56%
SCLT1 56%
SDCCAG3 56%
SLC41A3 56%
SLF1 56%
SLF2 56%
SMCHD1 56%
SP100 56%
SPG7 56%
SQRDL 56%
STAU2 56%
STEAP4 56%
SWT1 56%
TBC1D10B 56%
TCHP 56%
TRAM1L1 56%
UCK1 56%
WIPF1 56%
WNT16 56%
ZNF224 56%
ZNF530 56%
ZNF544 56%
ZNF549 56%
ZNF654 56%
ZNF681 56%
Mutation Types
missense_variant
frameshift_variant
stop_lost
stop_gained
disruptive_inframe_deletion
conservative_inframe_insertion
multi_hit

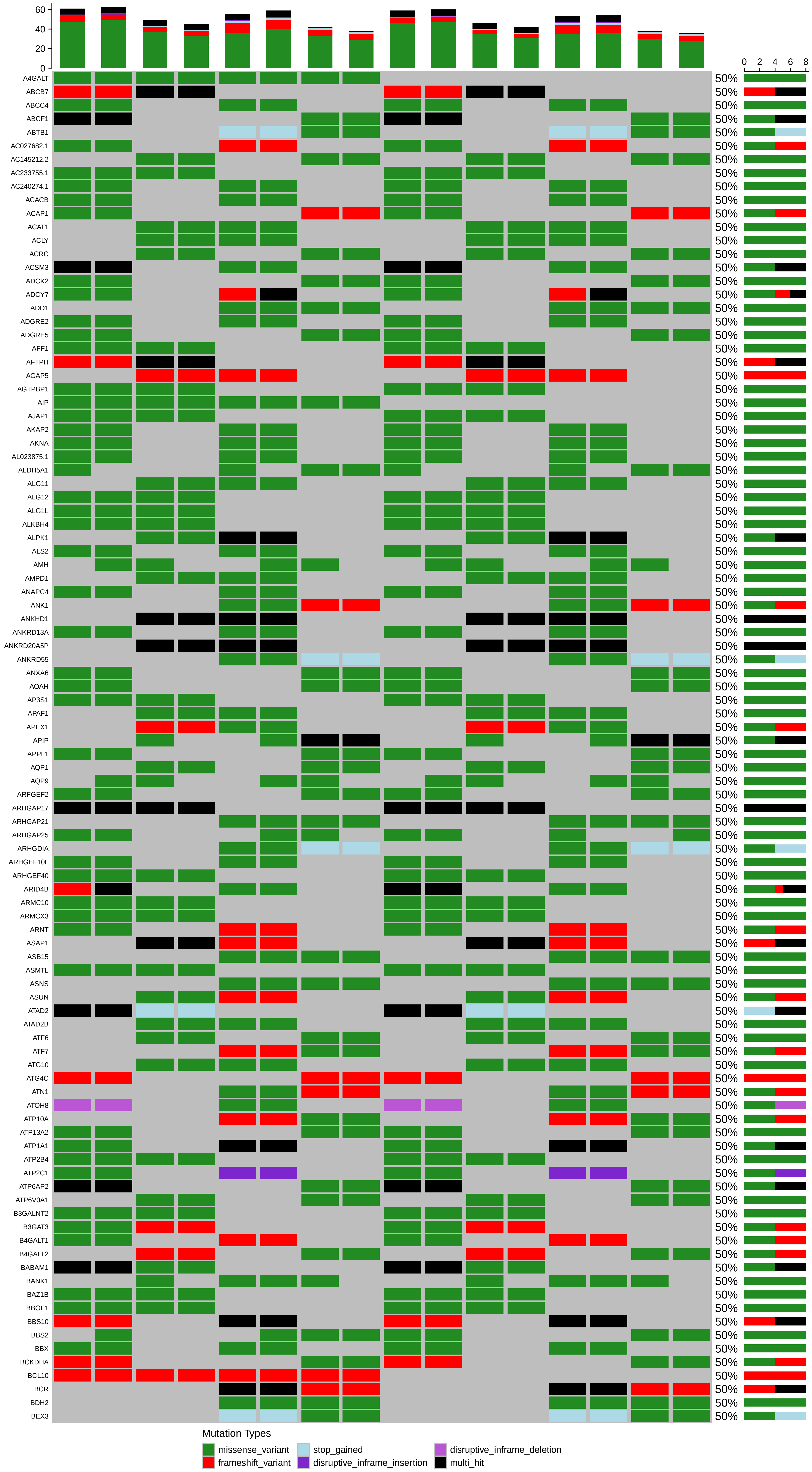

60
40
20
0
0 2 4 6 8
A4GALT
ABCB7
ABCC4
ABCF1
ABTB1
AC027682.1
AC145212.2
AC233755.1
AC240274.1
ACACB
ACAP1
ACAT1
ACLY
ACRC
ACSM3
ADCK2
ADCY7
ADD1
ADGRE2
ADGRE5
AFF1
AFTPH
AGAP5
AGTPBP1
AIP
AJAP1
AKAP2
AKNA
AL023875.1
ALDH5A1
ALG11
ALG12
ALG1L
ALKBH4
ALPK1
ALS2
AMH
AMPD1
ANAPC4
ANK1
ANKHD1
ANKRD13A
ANKRD20A5P
ANKRD55
ANXA6
AOAH
AP3S1
APAF1
APEX1
APIP
APPL1
AQP1
AQP9
ARFGEF2
ARHGAP17
ARHGAP21
ARHGAP25
ARHGDIA
ARHGEF10L
ARHGEF40
ARID4B
ARMC10
ARMCX3
ARNT
ASAP1
ASB15
ASMTL
ASNS
ASUN
ATAD2
ATAD2B
ATF6
ATF7
ATG10
ATG4C
ATN1
ATOH8
ATP10A
ATP13A2
ATP1A1
ATP2B4
ATP2C1
ATP6AP2
ATP6V0A1
B3GALNT2
B3GAT3
B4GALT1
B4GALT2
BABAM1
BANK1
BAZ1B
BBOF1
BBS10
BBS2
BBX
BCKDHA
BCL10
BCR
BDH2
BEX3
50%
Mutation Types
missense_variant
frameshift_variant
stop_gained
disruptive_inframe_insertion
disruptive_inframe_deletion
multi_hit

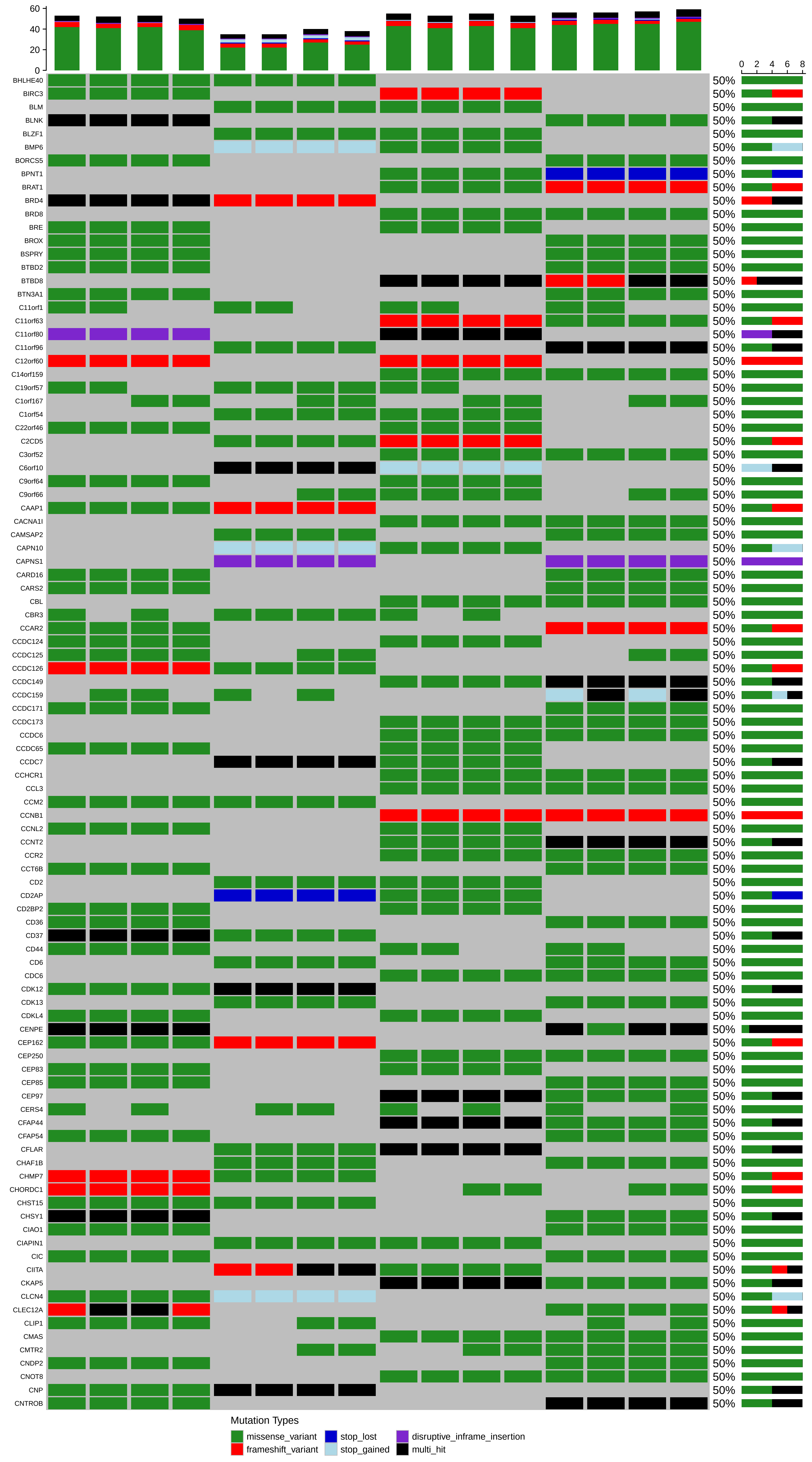

60
40
20
0
0 2 4 6 8
BHLHE40
BIRC3
BLM
BLNK
BLZF1
BMP6
BORCS5
BPNT1
BRAT1
BRD4
BRD8
BRE
BROX
BSPRY
BTBD2
BTBD8
BTN3A1
C11orf1
C11orf63
C11orf80
C11orf96
C12orf60
C14orf159
C19orf57
C1orf167
C1orf54
C22orf46
C2CD5
C3orf52
C6orf10
C9orf64
C9orf66
CAAP1
CACNA1I
CAMSAP2
CAPN10
CAPNS1
CARD16
CARS2
CBL
CBR3
CCAR2
CCDC124
CCDC125
CCDC126
CCDC149
CCDC159
CCDC171
CCDC173
CCDC6
CCDC65
CCDC7
CCHCR1
CCL3
CCM2
CCNB1
CCNL2
CCNT2
CCR2
CCT6B
CD2
CD2AP
CD2BP2
CD36
CD37
CD44
CD6
CDC6
CDK12
CDK13
CDKL4
CENPE
CEP162
CEP250
CEP83
CEP85
CEP97
CERS4
CFAP44
CFAP54
CFLAR
CHAF1B
CHMP7
CHORDC1
CHST15
CHSY1
CIAO1
CIAPIN1
CIC
CIITA
CKAP5
CLCN4
CLEC12A
CLIP1
CMAS
CMTR2
CNDP2
CNOT8
CNP
CNTROB
50%
Mutation Types
missense_variant
frameshift_variant
stop_lost
stop_gained
disruptive_inframe_insertion
multi_hit

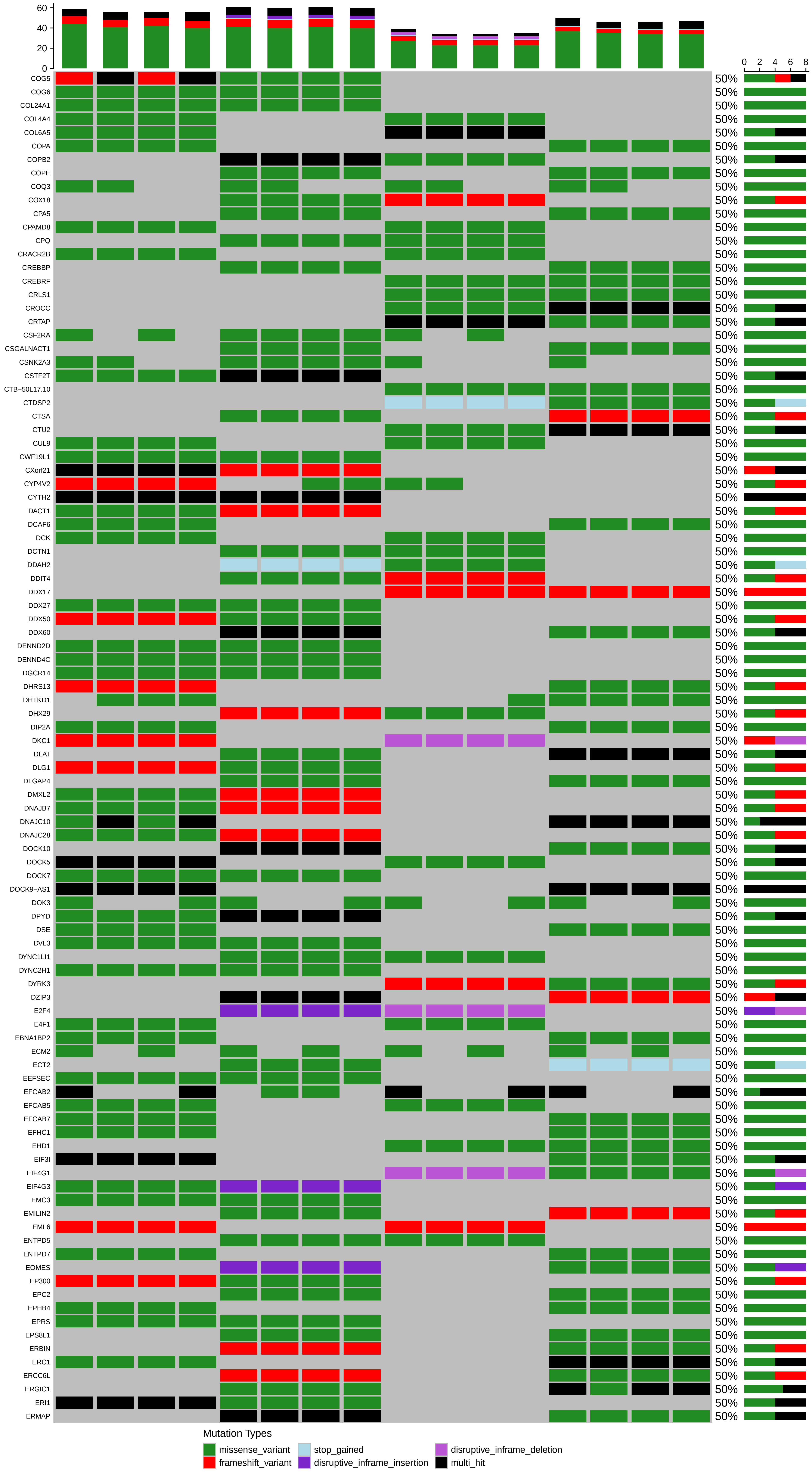

0
20
40
60
0 2 4 6 8
COG5
COG6
COL24A1
COL4A4
COL6A5
COPA
COPB2
COPE
COQ3
COX18
CPA5
CPAMD8
CPQ
CRACR2B
CREBBP
CREBRF
CRLS1
CROCC
CRTAP
CSF2RA
CSGALNACT1
CSNK2A3
CSTF2T
CTB−50L17.10
CTDSP2
CTSA
CTU2
CUL9
CWF19L1
CXorf21
CYP4V2
CYTH2
DACT1
DCAF6
DCK
DCTN1
DDAH2
DDIT4
DDX17
DDX27
DDX50
DDX60
DENND2D
DENND4C
DGCR14
DHRS13
DHTKD1
DHX29
DIP2A
DKC1
DLAT
DLG1
DLGAP4
DMXL2
DNAJB7
DNAJC10
DNAJC28
DOCK10
DOCK5
DOCK7
DOCK9−AS1
DOK3
DPYD
DSE
DVL3
DYNC1LI1
DYNC2H1
DYRK3
DZIP3
E2F4
E4F1
EBNA1BP2
ECM2
ECT2
EEFSEC
EFCAB2
EFCAB5
EFCAB7
EFHC1
EHD1
EIF3I
EIF4G1
EIF4G3
EMC3
EMILIN2
EML6
ENTPD5
ENTPD7
EOMES
EP300
EPC2
EPHB4
EPRS
EPS8L1
ERBIN
ERC1
ERCC6L
ERGIC1
ERI1
ERMAP
50%
Mutation Types
missense_variant
frameshift_variant
stop_gained
disruptive_inframe_insertion
disruptive_inframe_deletion
multi_hit

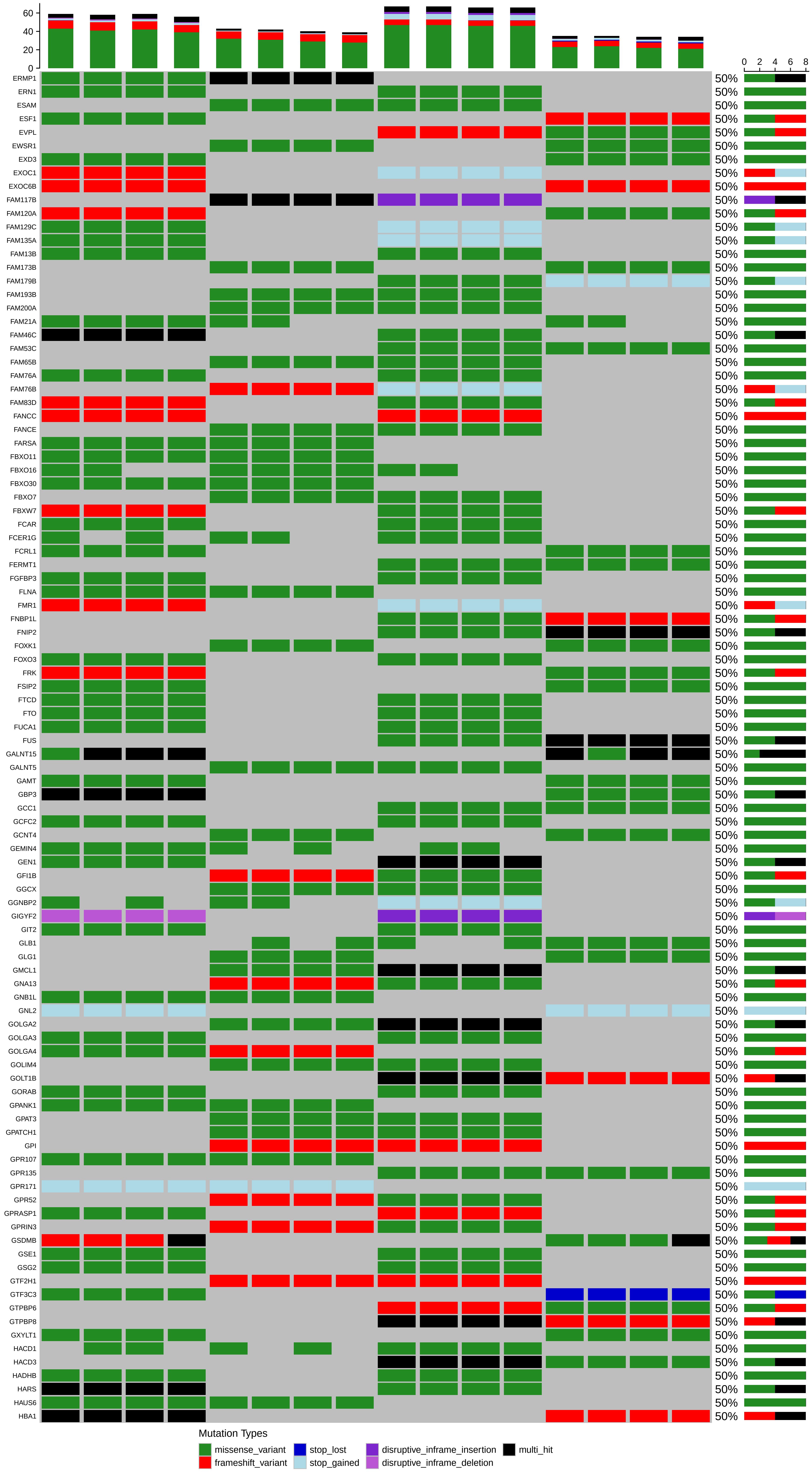
60
40
20
0
0 2 4 6 8
ERMP1
ERN1
ESAM
ESF1
EVPL
EWSR1
EXD3
EXOC1
EXOC6B
FAM117B
FAM120A
FAM129C
FAM135A
FAM13B
FAM173B
FAM179B
FAM193B
FAM200A
FAM21A
FAM46C
FAM53C
FAM65B
FAM76A
FAM76B
FAM83D
FANCC
FANCE
FARSA
FBXO11
FBXO16
FBXO30
FBXO7
FBXW7
FCAR
FCER1G
FCRL1
FERMT1
FGFBP3
FLNA
FMR1
FNBP1L
FNIP2
FOXK1
FOXO3
FRK
FSIP2
FTCD
FTO
FUCA1
FUS
GALNT15
GALNT5
GAMT
GBP3
GCC1
GCFC2
GCNT4
GEMIN4
GEN1
GFI1B
GGCX
GGNBP2
GIGYF2
GIT2
GLB1
GLG1
GMCL1
GNA13
GNB1L
GNL2
GOLGA2
GOLGA3
GOLGA4
GOLIM4
GOLT1B
GORAB
GPANK1
GPAT3
GPATCH1
GPI
GPR107
GPR135
GPR171
GPR52
GPRASP1
GPRIN3
GSDMB
GSE1
GSG2
GTF2H1
GTF3C3
GTPBP6
GTPBP8
GXYLT1
HACD1
HACD3
HADHB
HARS
HAUS6
HBA1
Mutation Types
missense_variant
frameshift_variant
stop_lost
stop_gained
disruptive_inframe_insertion
disruptive_inframe_deletion
multi_hit

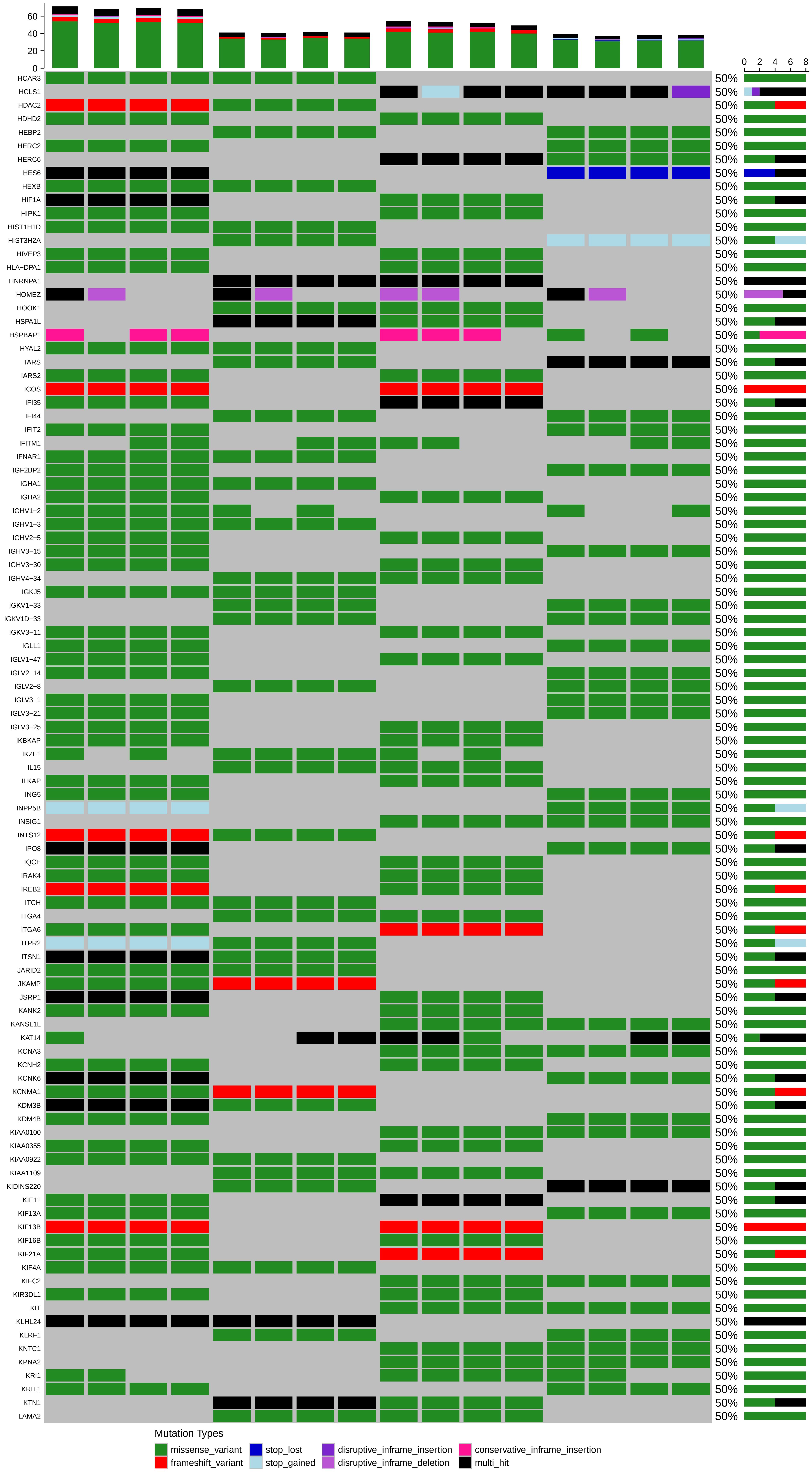

60
40
20
0
0 2 4 6 8
HCAR3
HCLS1
HDAC2
HDHD2
HEBP2
HERC2
HERC6
HES6
HEXB
HIF1A
HIPK1
HIST1H1D
HIST3H2A
HIVEP3
HLA−DPA1
HNRNPA1
HOMEZ
HOOK1
HSPA1L
HSPBAP1
HYAL2
IARS
IARS2
ICOS
IFI35
IFI44
IFIT2
IFITM1
IFNAR1
IGF2BP2
IGHA1
IGHA2
IGHV1−2
IGHV1−3
IGHV2−5
IGHV3−15
IGHV3−30
IGHV4−34
IGKJ5
IGKV1−33
IGKV1D−33
IGKV3−11
IGLL1
IGLV1−47
IGLV2−14
IGLV2−8
IGLV3−1
IGLV3−21
IGLV3−25
IKBKAP
IKZF1
IL15
ILKAP
ING5
INPP5B
INSIG1
INTS12
IPO8
IQCE
IRAK4
IREB2
ITCH
ITGA4
ITGA6
ITPR2
ITSN1
JARID2
JKAMP
JSRP1
KANK2
KANSL1L
KAT14
KCNA3
KCNH2
KCNK6
KCNMA1
KDM3B
KDM4B
KIAA0100
KIAA0355
KIAA0922
KIAA1109
KIDINS220
KIF11
KIF13A
KIF13B
KIF16B
KIF21A
KIF4A
KIFC2
KIR3DL1
KIT
KLHL24
KLRF1
KNTC1
KPNA2
KRI1
KRIT1
KTN1
LAMA2
50%
Mutation Types
missense_variant
frameshift_variant
stop_lost
stop_gained
disruptive_inframe_insertion
disruptive_inframe_deletion
conservative_inframe_insertion
multi_hit

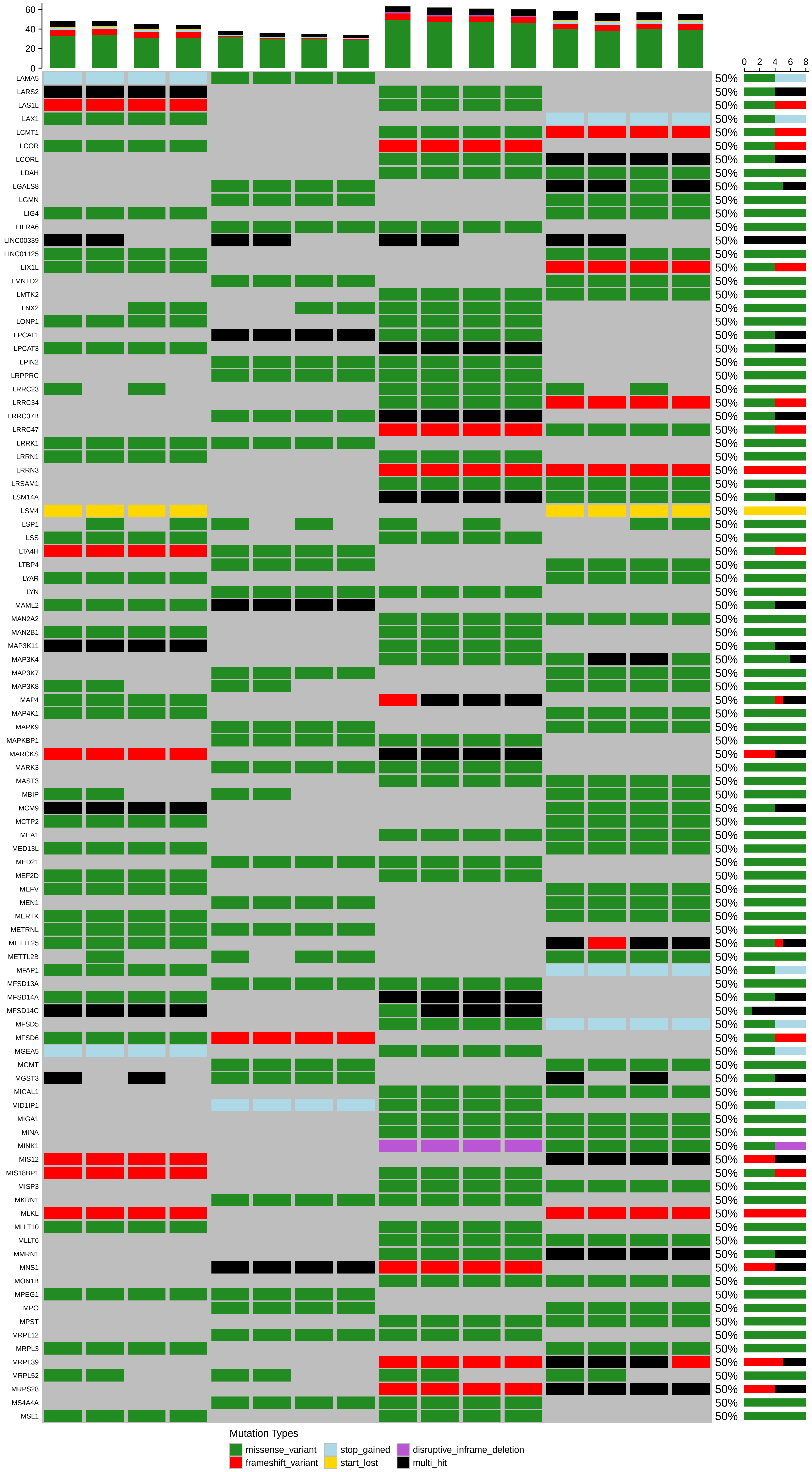

60
40
20
0
0 2 4 6 8
LAMA5
LARS2
LAS1L
LAX1
LCMT1
LCOR
LCORL
LDAH
LGALS8
LGMN
LIG4
LILRA6
LINC00339
LINC01125
LIX1L
LMNTD2
LMTK2
LNX2
LONP1
LPCAT1
LPCAT3
LPIN2
LRPPRC
LRRC23
LRRC34
LRRC37B
LRRC47
LRRK1
LRRN1
LRRN3
LRSAM1
LSM14A
LSM4
LSP1
LSS
LTA4H
LTBP4
LYAR
LYN
MAML2
MAN2A2
MAN2B1
MAP3K11
MAP3K4
MAP3K7
MAP3K8
MAP4
MAP4K1
MAPK9
MAPKBP1
MARCKS
MARK3
MAST3
MBIP
MCM9
MCTP2
MEA1
MED13L
MED21
MEF2D
MEFV
MEN1
MERTK
METRNL
METTL25
METTL2B
MFAP1
MFSD13A
MFSD14A
MFSD14C
MFSD5
MFSD6
MGEA5
MGMT
MGST3
MICAL1
MID1IP1
MIGA1
MINA
MINK1
MIS12
MIS18BP1
MISP3
MKRN1
MLKL
MLLT10
MLLT6
MMRN1
MNS1
MON1B
MPEG1
MPO
MPST
MRPL12
MRPL3
MRPL39
MRPL52
MRPS28
MS4A4A
MSL1
50%
Mutation Types
missense_variant
frameshift_variant
stop_gained
start_lost
disruptive_inframe_deletion
multi_hit

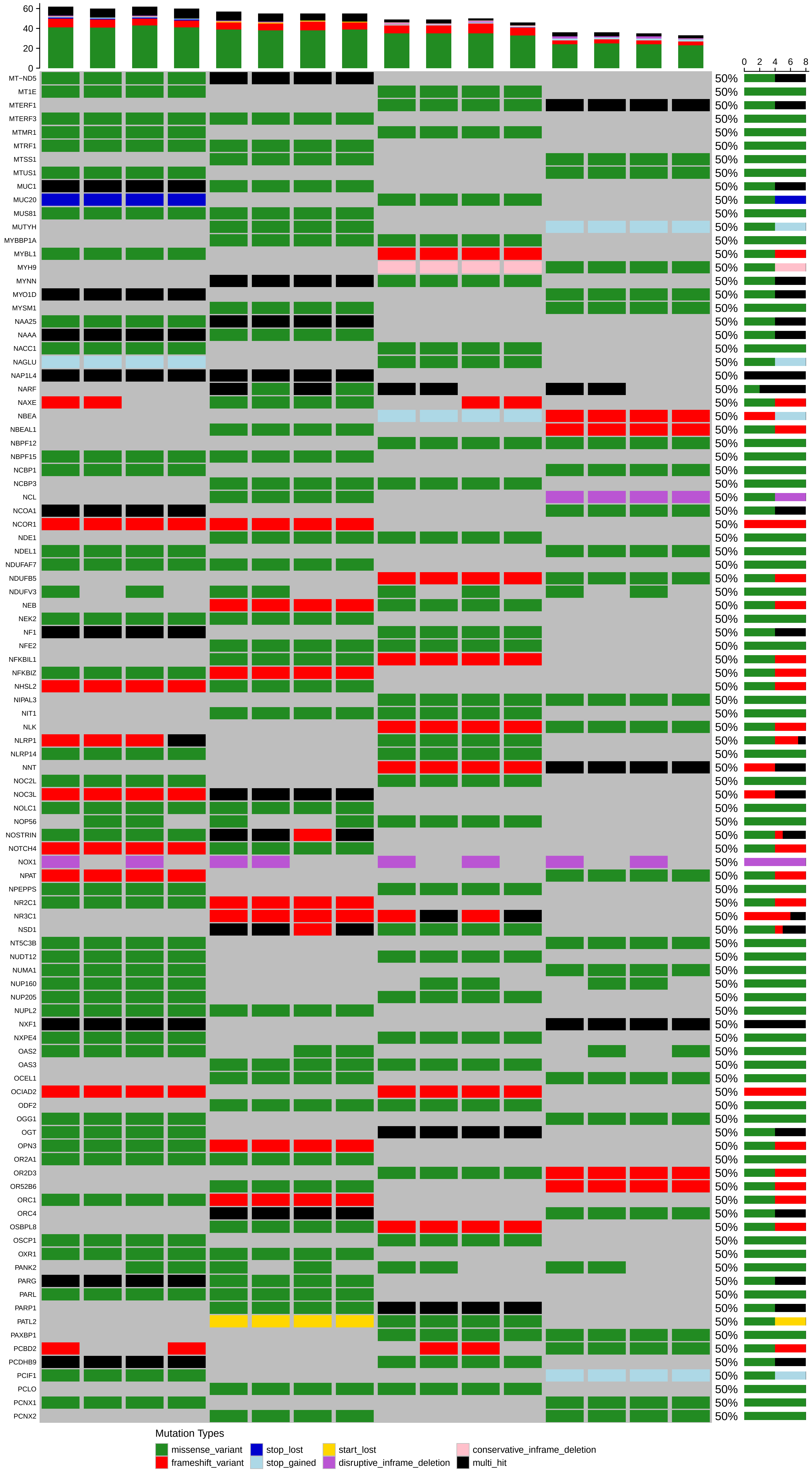

0
20
40
60
0 2 4 6 8
MT−ND5
MT1E
MTERF1
MTERF3
MTMR1
MTRF1
MTSS1
MTUS1
MUC1
MUC20
MUS81
MUTYH
MYBBP1A
MYBL1
MYH9
MYNN
MYO1D
MYSM1
NAA25
NAAA
NACC1
NAGLU
NAP1L4
NARF
NAXE
NBEA
NBEAL1
NBPF12
NBPF15
NCBP1
NCBP3
NCL
NCOA1
NCOR1
NDE1
NDEL1
NDUFAF7
NDUFB5
NDUFV3
NEB
NEK2
NF1
NFE2
NFKBIL1
NFKBIZ
NHSL2
NIPAL3
NIT1
NLK
NLRP1
NLRP14
NNT
NOC2L
NOC3L
NOLC1
NOP56
NOSTRIN
NOTCH4
NOX1
NPAT
NPEPPS
NR2C1
NR3C1
NSD1
NT5C3B
NUDT12
NUMA1
NUP160
NUP205
NUPL2
NXF1
NXPE4
OAS2
OAS3
OCEL1
OCIAD2
ODF2
OGG1
OGT
OPN3
OR2A1
OR2D3
OR52B6
ORC1
ORC4
OSBPL8
OSCP1
OXR1
PANK2
PARG
PARL
PARP1
PATL2
PAXBP1
PCBD2
PCDHB9
PCIF1
PCLO
PCNX1
PCNX2
50%
Mutation Types
missense_variant
frameshift_variant
stop_lost
stop_gained
start_lost
disruptive_inframe_deletion
conservative_inframe_deletion
multi_hit

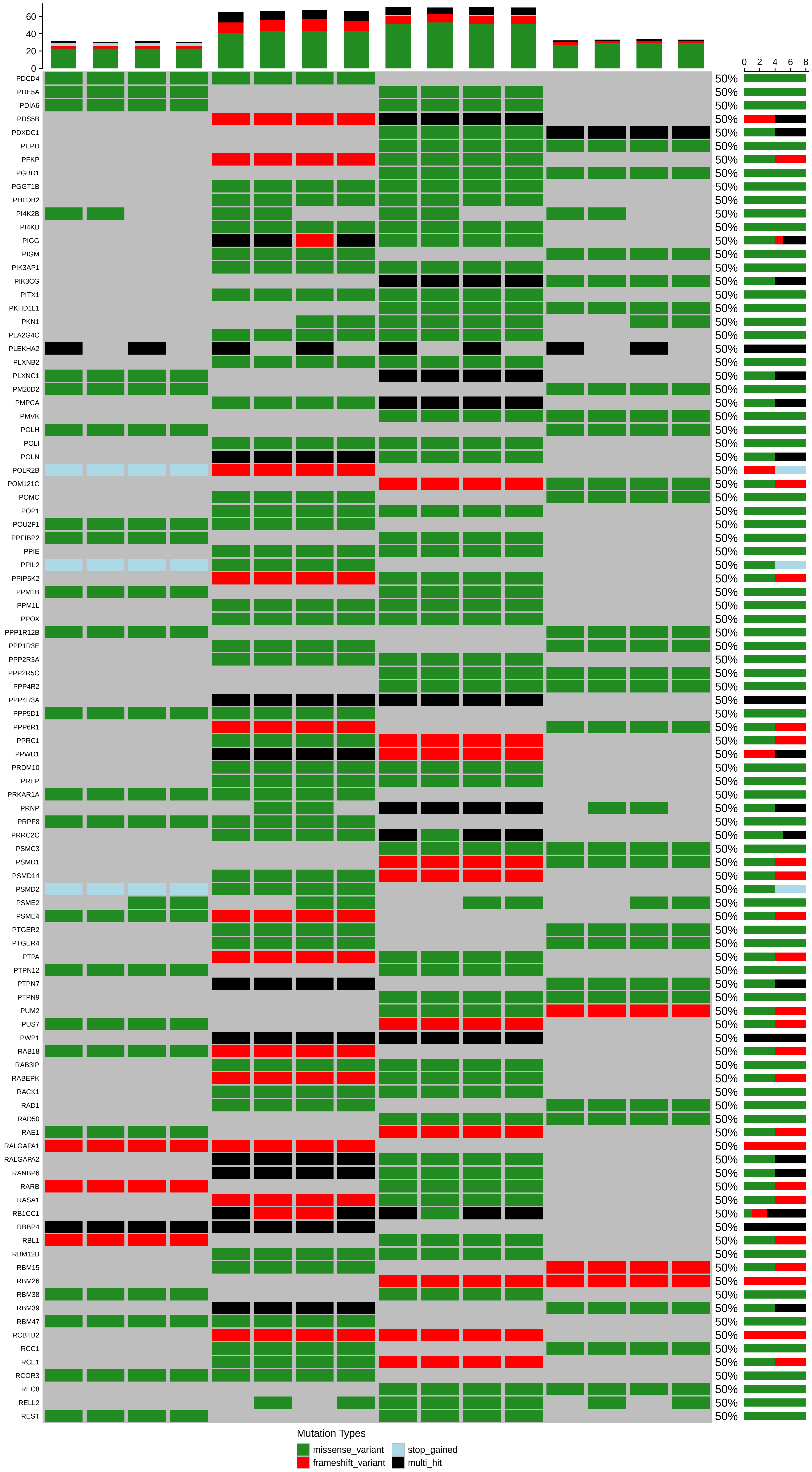

0 20 40 60
0 2 4 6 8
PDCD4
PDE5A
PDIA6
PDS5B
PDXDC1
PEPD
PFKP
PGBD1
PGGT1B
PHLDB2
PI4K2B
PI4KB
PIGG
PIGM
PIK3AP1
PIK3CG
PITX1
PKHD1L1
PKN1
PLA2G4C
PLEKHA2
PLXNB2
PLXNC1
PM20D2
PMPCA
PMVK
POLH
POLI
POLN
POLR2B
POM121C
POMC
POP1
POU2F1
PPFIBP2
PPIE
PPIL2
PPIP5K2
PPM1B
PPM1L
PPOX
PPP1R12B
PPP1R3E
PPP2R3A
PPP2R5C
PPP4R2
PPP4R3A
PPP5D1
PPP6R1
PPRC1
PPWD1
PRDM10
PREP
PRKAR1A
PRNP
PRPF8
PRRC2C
PSMC3
PSMD1
PSMD14
PSMD2
PSME2
PSME4
PTGER2
PTGER4
PTPA
PTPN12
PTPN7
PTPN9
PUM2
PUS7
PWP1
RAB18
RAB3IP
RABEPK
RACK1
RAD1
RAD50
RAE1
RALGAPA1
RALGAPA2
RANBP6
RARB
RASA1
RB1CC1
RBBP4
RBL1
RBM12B
RBM15
RBM26
RBM38
RBM39
RBM47
RCBTB2
RCC1
RCE1
RCOR3
REC8
RELL2
REST
50%
Mutation Types
missense_variant
frameshift_variant
stop_gained
multi_hit

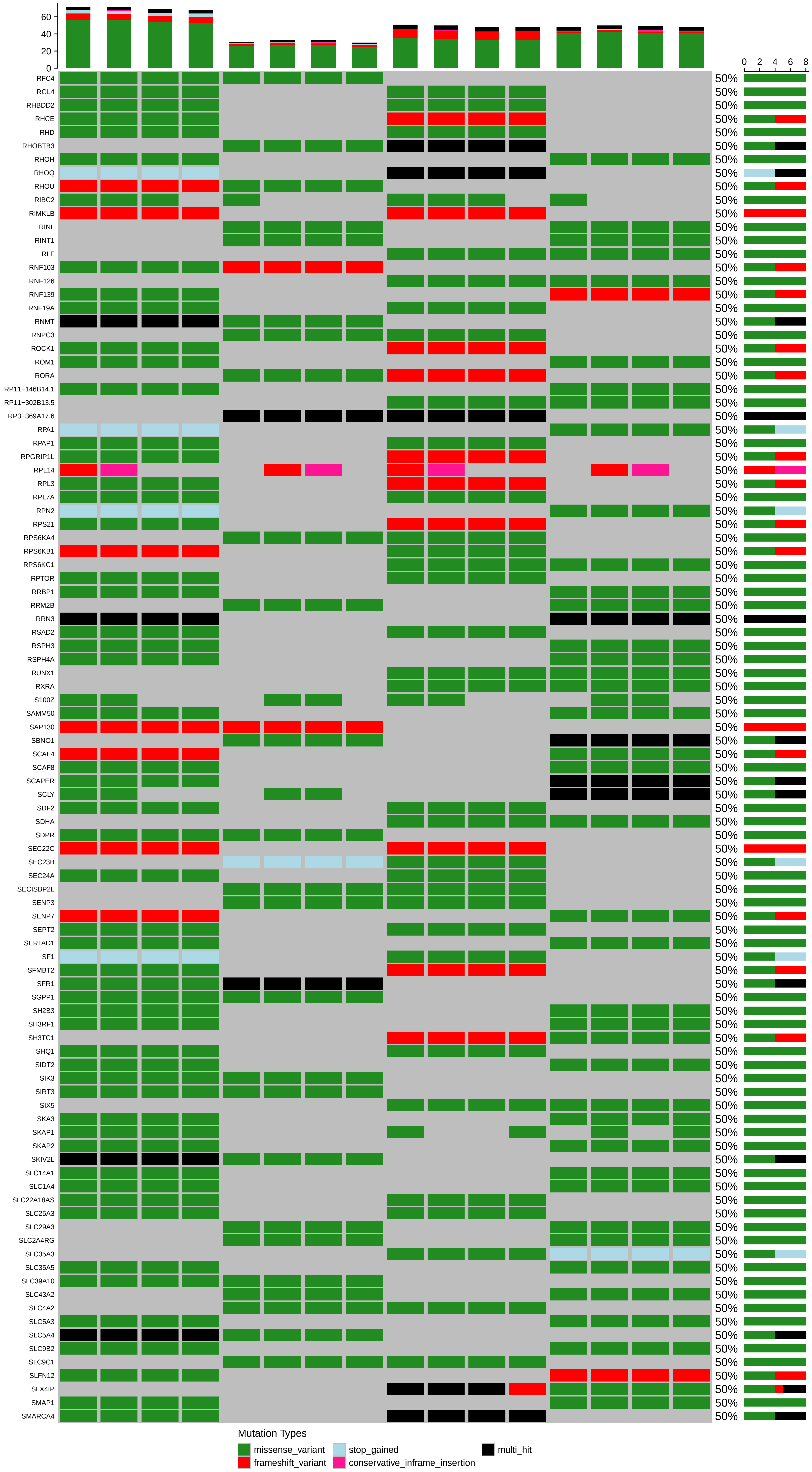

0
20
40
60
0 2 4 6 8
RFC4
RGL4
RHBDD2
RHCE
RHD
RHOBTB3
RHOH
RHOQ
RHOU
RIBC2
RIMKLB
RINL
RINT1
RLF
RNF103
RNF126
RNF139
RNF19A
RNMT
RNPC3
ROCK1
ROM1
RORA
RP11−146B14.1
RP11−302B13.5
RP3−369A17.6
RPA1
RPAP1
RPGRIP1L
RPL14
RPL3
RPL7A
RPN2
RPS21
RPS6KA4
RPS6KB1
RPS6KC1
RPTOR
RRBP1
RRM2B
RRN3
RSAD2
RSPH3
RSPH4A
RUNX1
RXRA
S100Z
SAMM50
SAP130
SBNO1
SCAF4
SCAF8
SCAPER
SCLY
SDF2
SDHA
SDPR
SEC22C
SEC23B
SEC24A
SECISBP2L
SENP3
SENP7
SEPT2
SERTAD1
SF1
SFMBT2
SFR1
SGPP1
SH2B3
SH3RF1
SH3TC1
SHQ1
SIDT2
SIK3
SIRT3
SIX5
SKA3
SKAP1
SKAP2
SKIV2L
SLC14A1
SLC1A4
SLC22A18AS
SLC25A3
SLC29A3
SLC2A4RG
SLC35A3
SLC35A5
SLC39A10
SLC43A2
SLC4A2
SLC5A3
SLC5A4
SLC9B2
SLC9C1
SLFN12
SLX4IP
SMAP1
SMARCA4
50%
Mutation Types
missense_variant
frameshift_variant
stop_gained
conservative_inframe_insertion
multi_hit

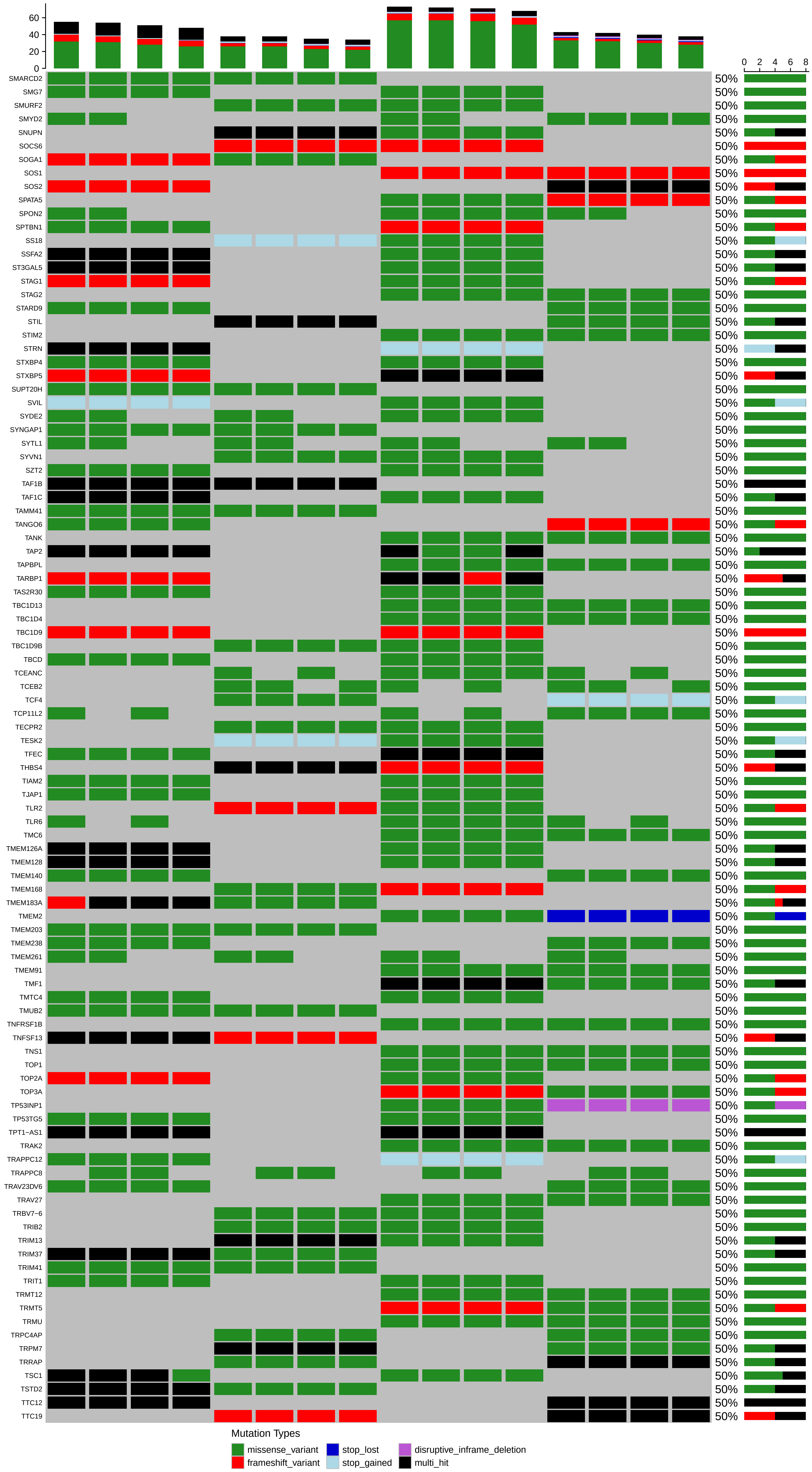

60
40
20
0
0 2 4 6 8
SMARCD2
SMG7
SMURF2
SMYD2
SNUPN
SOCS6
SOGA1
SOS1
SOS2
SPATA5
SPON2
SPTBN1
SS18
SSFA2
ST3GAL5
STAG1
STAG2
STARD9
STIL
STIM2
STRN
STXBP4
STXBP5
SUPT20H
SVIL
SYDE2
SYNGAP1
SYTL1
SYVN1
SZT2
TAF1B
TAF1C
TAMM41
TANGO6
TANK
TAP2
TAPBPL
TARBP1
TAS2R30
TBC1D13
TBC1D4
TBC1D9
TBC1D9B
TBCD
TCEANC
TCEB2
TCF4
TCP11L2
TECPR2
TESK2
TFEC
THBS4
TIAM2
TJAP1
TLR2
TLR6
TMC6
TMEM126A
TMEM128
TMEM140
TMEM168
TMEM183A
TMEM2
TMEM203
TMEM238
TMEM261
TMEM91
TMF1
TMTC4
TMUB2
TNFRSF1B
TNFSF13
TNS1
TOP1
TOP2A
TOP3A
TP53INP1
TP53TG5
TPT1−AS1
TRAK2
TRAPPC12
TRAPPC8
TRAV23DV6
TRAV27
TRBV7−6
TRIB2
TRIM13
TRIM37
TRIM41
TRIT1
TRMT12
TRMT5
TRMU
TRPC4AP
TRPM7
TRRAP
TSC1
TSTD2
TTC12
TTC19
50%
Mutation Types
missense_variant
frameshift_variant
stop_lost
stop_gained
disruptive_inframe_deletion
multi_hit

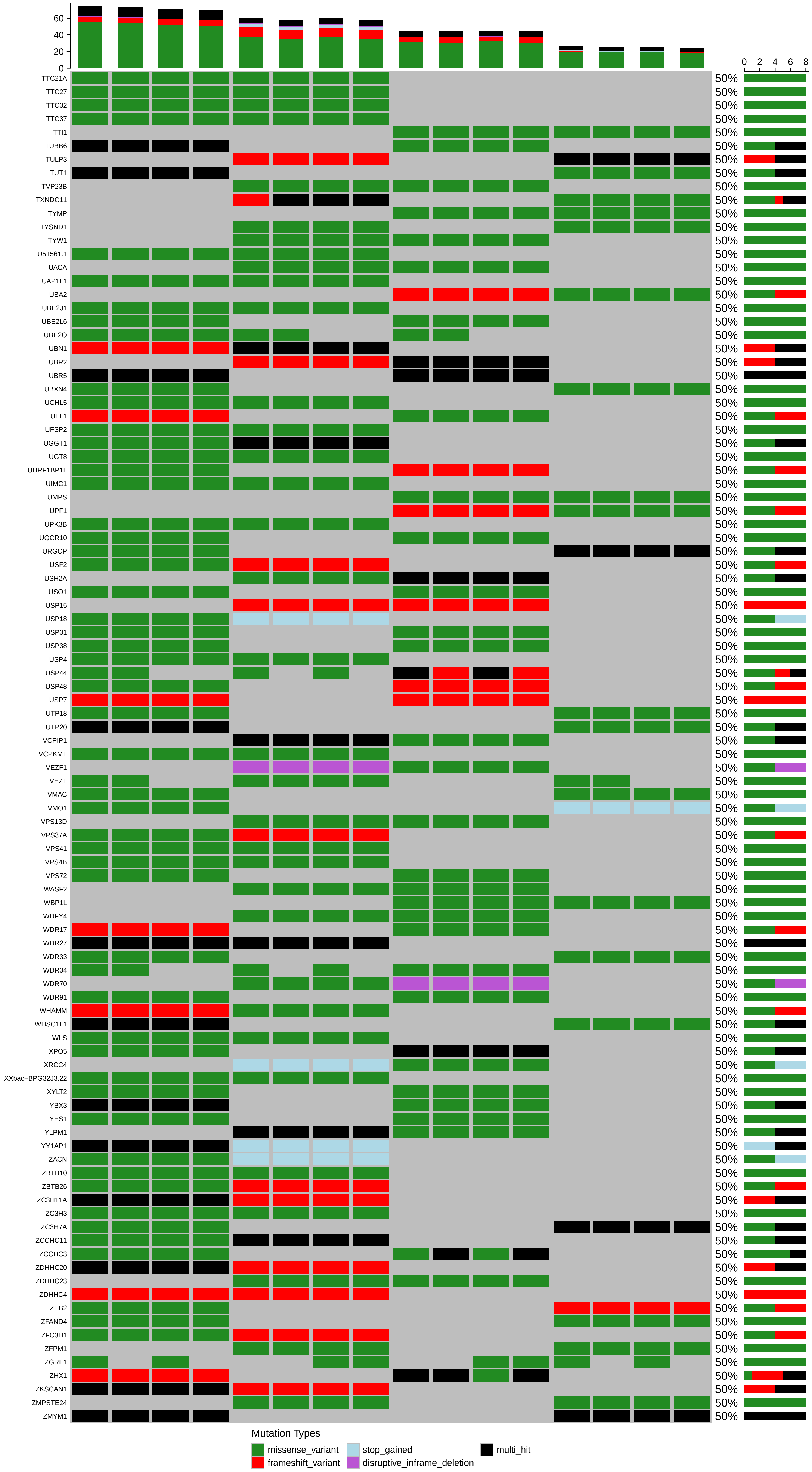

60
40
20
0
0 2 4 6 8
TTC21A
TTC27
TTC32
TTC37
TTI1
TUBB6
TULP3
TUT1
TVP23B
TXNDC11
TYMP
TYSND1
TYW1
U51561.1
UACA
UAP1L1
UBA2
UBE2J1
UBE2L6
UBE2O
UBN1
UBR2
UBR5
UBXN4
UCHL5
UFL1
UFSP2
UGGT1
UGT8
UHRF1BP1L
UIMC1
UMPS
UPF1
UPK3B
UQCR10
URGCP
USF2
USH2A
USO1
USP15
USP18
USP31
USP38
USP4
USP44
USP48
USP7
UTP18
UTP20
VCPIP1
VCPKMT
VEZF1
VEZT
VMAC
VMO1
VPS13D
VPS37A
VPS41
VPS4B
VPS72
WASF2
WBP1L
WDFY4
WDR17
WDR27
WDR33
WDR34
WDR70
WDR91
WHAMM
WHSC1L1
WLS
XPO5
XRCC4
XXbac-BPG32J3.22
XYLT2
YBX3
YES1
YLPM1
YY1AP1
ZACN
ZBTB10
ZBTB26
ZC3H11A
ZC3H3
ZC3H7A
ZCCHC11
ZCCHC3
ZDHHC20
ZDHHC23
ZDHHC4
ZEB2
ZFAND4
ZFC3H1
ZFPM1
ZGRF1
ZHX1
ZKSCAN1
ZMPSTE24
ZMYM1
50%
Mutation Types
missense_variant
frameshift_variant
stop_gained
disruptive_inframe_deletion
multi_hit

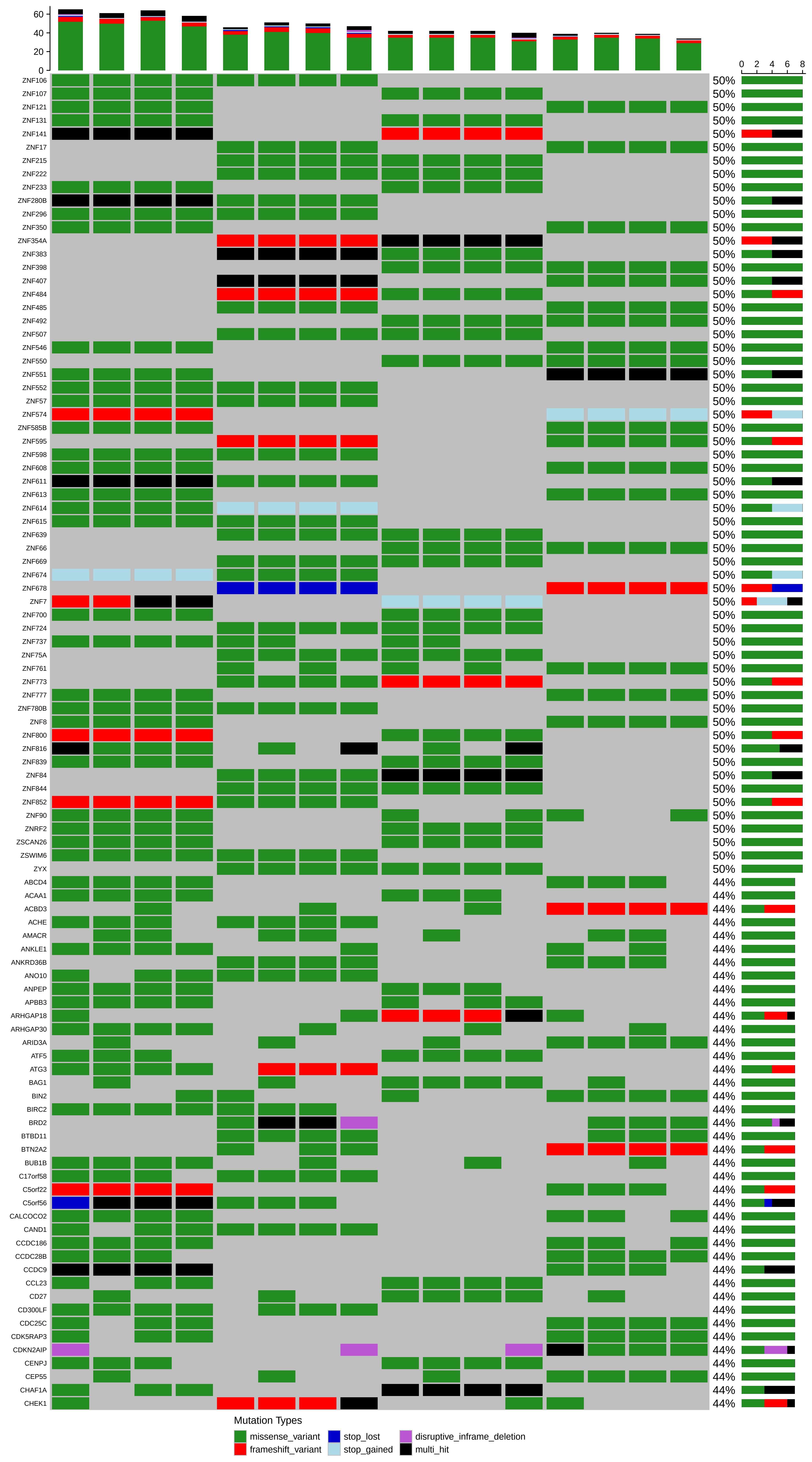
ZNF106
ZNF107
ZNF121
ZNF131
ZNF141
ZNF17
ZNF215
ZNF222
ZNF233
ZNF280B
ZNF296
ZNF350
ZNF354A
ZNF383
ZNF398
ZNF407
ZNF484
ZNF485
ZNF492
ZNF507
ZNF546
ZNF550
ZNF551
ZNF552
ZNF57
ZNF574
ZNF585B
ZNF595
ZNF598
ZNF608
ZNF611
ZNF613
ZNF614
ZNF615
ZNF639
ZNF66
ZNF669
ZNF674
ZNF678
ZNF7
ZNF700
ZNF724
ZNF737
ZNF75A
ZNF761
ZNF773
ZNF777
ZNF780B
ZNF8
ZNF800
ZNF816
ZNF839
ZNF84
ZNF844
ZNF852
ZNF90
ZNRF2
ZSCAN26
ZSWIM6
ZYX
ABCD4
ACAA1
ACBD3
ACHE
AMACR
ANKLE1
ANKRD36B
ANO10
ANPEP
APBB3
ARHGAP18
ARHGAP30
ARID3A
ATF5
ATG3
BAG1
BIN2
BIRC2
BRD2
BTBD11
BTN2A2
BUB1B
C17orf58
C5orf22
C5orf56
CALCOCO2
CAND1
CCDC186
CCDC28B
CCDC9
CCL23
CD27
CD300LF
CDC25C
CDK5RAP3
CDKN2AIP
CENPJ
CEP55
CHAF1A
CHEK1
Mutation Types
missense_variant
frameshift_variant
stop_lost
stop_gained
disruptive_inframe_deletion
multi_hit

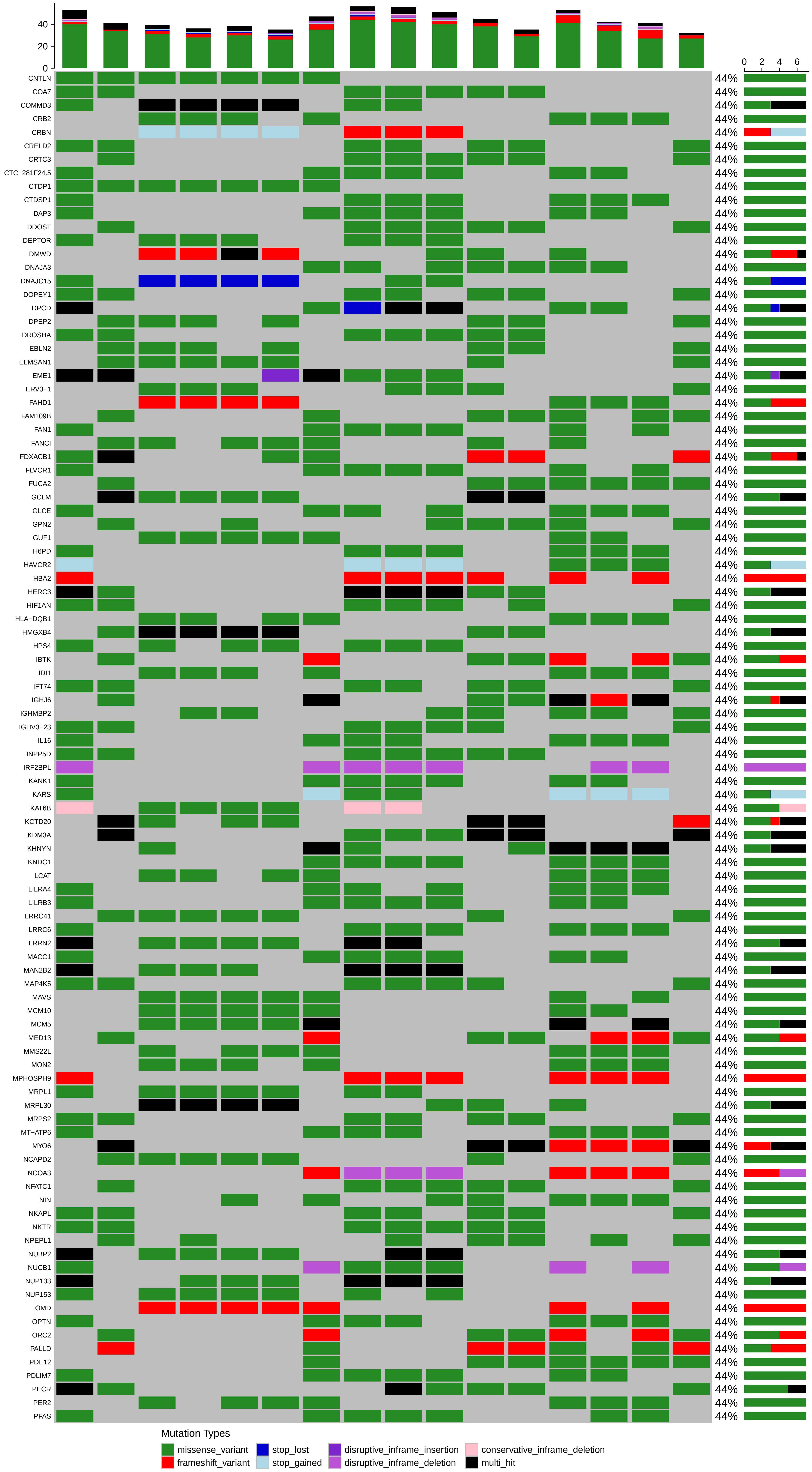
0
20
40
0 2 4 6
CNTLN 44%
COA7 44%
COMMD3 44%
CRB2 44%
CRBN 44%
CRELD2 44%
CRTC3 44%
CTC−281F24.5 44%
CTDP1 44%
CTDSP1 44%
DAP3 44%
DDOST 44%
DEPTOR 44%
DMWD 44%
DNAJA3 44%
DNAJC15 44%
DOPEY1 44%
DPCD 44%
DPEP2 44%
DROSHA 44%
EBLN2 44%
ELMSAN1 44%
EME1 44%
ERV3−1 44%
FAHD1 44%
FAM109B 44%
FAN1 44%
FANCI 44%
FDXACB1 44%
FLVCR1 44%
FUCA2 44%
GCLM 44%
GLCE 44%
GPN2 44%
GUF1 44%
H6PD 44%
HAVCR2 44%
HBA2 44%
HERC3 44%
HIF1AN 44%
HLA−DQB1 44%
HMGXB4 44%
HPS4 44%
IBTK 44%
IDI1 44%
IFT74 44%
IGHJ6 44%
IGHMBP2 44%
IGHV3−23 44%
IL16 44%
INPP5D 44%
IRF2BPL 44%
KANK1 44%
KARS 44%
KAT6B 44%
KCTD20 44%
KDM3A 44%
KHNYN 44%
KNDC1 44%
LCAT 44%
LILRA4 44%
LILRB3 44%
LRRC41 44%
LRRC6 44%
LRRN2 44%
MACC1 44%
MAN2B2 44%
MAP4K5 44%
MAVS 44%
MCM10 44%
MCM5 44%
MED13 44%
MMS22L 44%
MON2 44%
MPHOSPH9 44%
MRPL1 44%
MRPL30 44%
MRPS2 44%
MT−ATP6 44%
MYO6 44%
NCAPD2 44%
NCOA3 44%
NFATC1 44%
NIN 44%
NKAPL 44%
NKTR 44%
NPEPL1 44%
NUBP2 44%
NUCB1 44%
NUP133 44%
NUP153 44%
OMD 44%
OPTN 44%
ORC2 44%
PALLD 44%
PDE12 44%
PDLIM7 44%
PECR 44%
PER2 44%
PFAS 44%
Mutation Types
missense_variant
frameshift_variant
stop_lost
stop_gained
disruptive_inframe_insertion
disruptive_inframe_deletion
conservative_inframe_deletion
multi_hit

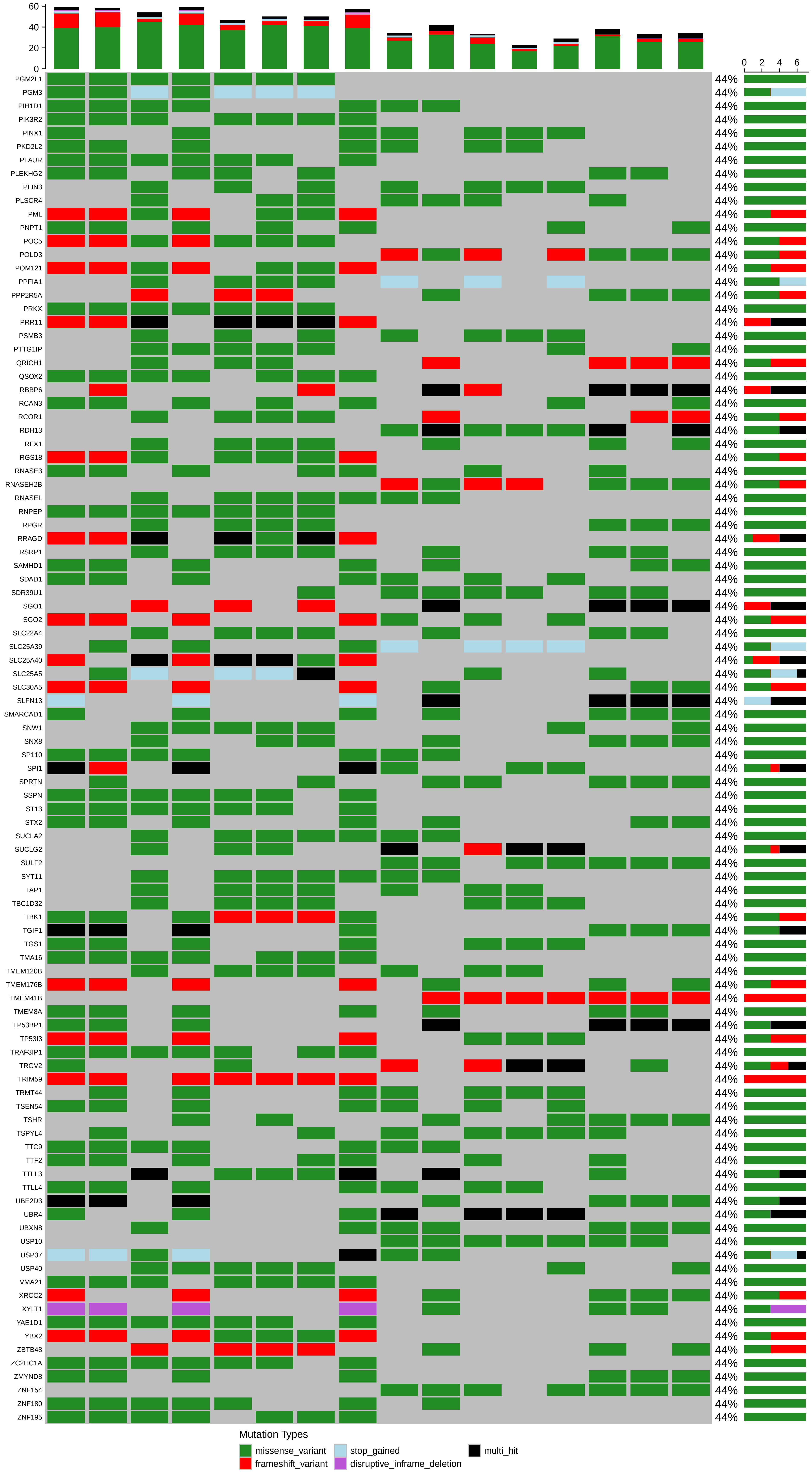

PGM2L1
PGM3
PIH1D1
PIK3R2
PINX1
PKD2L2
PLAUR
PLEKHG2
PLIN3
PLSCR4
PML
PNPT1
POC5
POLD3
POM121
PPFIA1
PPP2R5A
PRKX
PRR11
PSMB3
PTTG1IP
QRICH1
QSOX2
RBBP6
RCAN3
RCOR1
RDH13
RFX1
RGS18
RNASE3
RNASEH2B
RNASEL
RNPEP
RPGR
RRAGD
RSRP1
SAMHD1
SDAD1
SDR39U1
SGO1
SGO2
SLC22A4
SLC25A39
SLC25A40
SLC25A5
SLC30A5
SLFN13
SMARCAD1
SNW1
SNX8
SP110
SPI1
SPRTN
SSPN
ST13
STX2
SUCLA2
SUCLG2
SULF2
SYT11
TAP1
TBC1D32
TBK1
TGIF1
TGS1
TMA16
TMEM120B
TMEM176B
TMEM41B
TMEM8A
TP53BP1
TP53I3
TRAF3IP1
TRGV2
TRIM59
TRMT44
TSEN54
TSHR
TSPYL4
TTC9
TTF2
TTLL3
TTLL4
UBE2D3
UBR4
UBXN8
USP10
USP37
USP40
VMA21
XRCC2
XYLT1
YAE1D1
YBX2
ZBTB48
ZC2HC1A
ZMYND8
ZNF154
ZNF180
ZNF195
44%
Mutation Types
missense_variant
frameshift_variant
stop_gained
disruptive_inframe_deletion
multi_hit

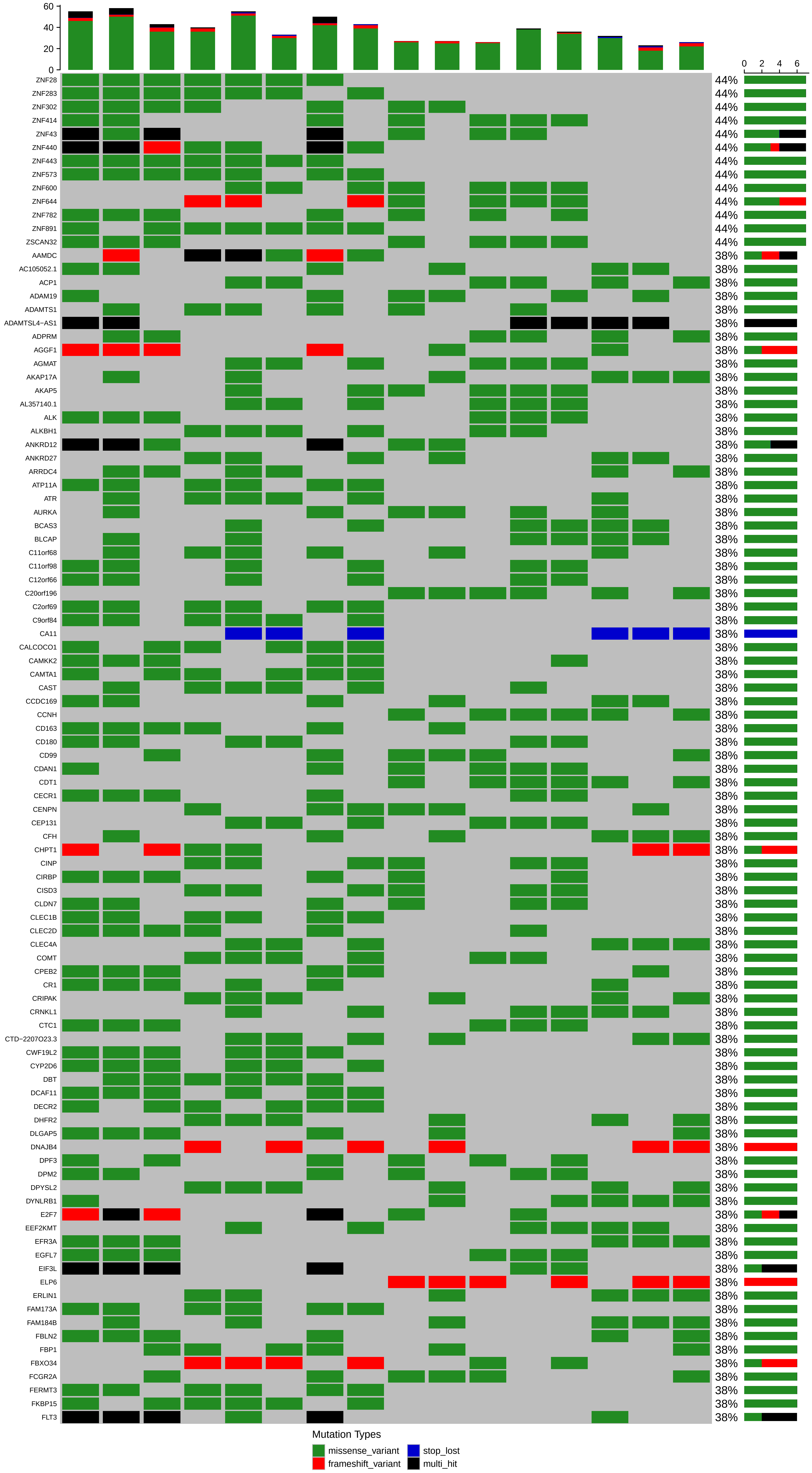

60
40
20
0
0 2 4 6
ZNF28 44%
ZNF283 44%
ZNF302 44%
ZNF414 44%
ZNF43 44%
ZNF440 44%
ZNF443 44%
ZNF573 44%
ZNF600 44%
ZNF644 44%
ZNF782 44%
ZNF891 44%
ZSCAN32 44%
AAMDC 38%
AC105052.1 38%
ACP1 38%
ADAM19 38%
ADAMTS1 38%
ADAMTSL4−AS1 38%
ADPRM 38%
AGGF1 38%
AGMAT 38%
AKAP17A 38%
AKAP5 38%
AL357140.1 38%
ALK 38%
ALKBH1 38%
ANKRD12 38%
ANKRD27 38%
ARRDC4 38%
ATP11A 38%
ATR 38%
AURKA 38%
BCAS3 38%
BLCAP 38%
C11orf68 38%
C11orf98 38%
C12orf66 38%
C20orf196 38%
C2orf69 38%
C9orf84 38%
CA11 38%
CALCOCO1 38%
CAMKK2 38%
CAMTA1 38%
CAST 38%
CCDC169 38%
CCNH 38%
CD163 38%
CD180 38%
CD99 38%
CDAN1 38%
CDT1 38%
CECR1 38%
CENPN 38%
CEP131 38%
CFH 38%
CHPT1 38%
CINP 38%
CIRBP 38%
CISD3 38%
CLDN7 38%
CLEC1B 38%
CLEC2D 38%
CLEC4A 38%
COMT 38%
CPEB2 38%
CR1 38%
CRIPAK 38%
CRNKL1 38%
CTC1 38%
CTD−2207O23.3 38%
CWF19L2 38%
CYP2D6 38%
DBT 38%
DCAF11 38%
DECR2 38%
DHFR2 38%
DLGAP5 38%
DNAJB4 38%
DPF3 38%
DPM2 38%
DPYSL2 38%
DYNLRB1 38%
E2F7 38%
EEF2KMT 38%
EFR3A 38%
EGFL7 38%
EIF3L 38%
ELP6 38%
ERLIN1 38%
FAM173A 38%
FAM184B 38%
FBLN2 38%
FBP1 38%
FBXO34 38%
FCGR2A 38%
FERMT3 38%
FKBP15 38%
FLT3 38%
Mutation Types
missense_variant
stop_lost
frameshift_variant
multi_hit

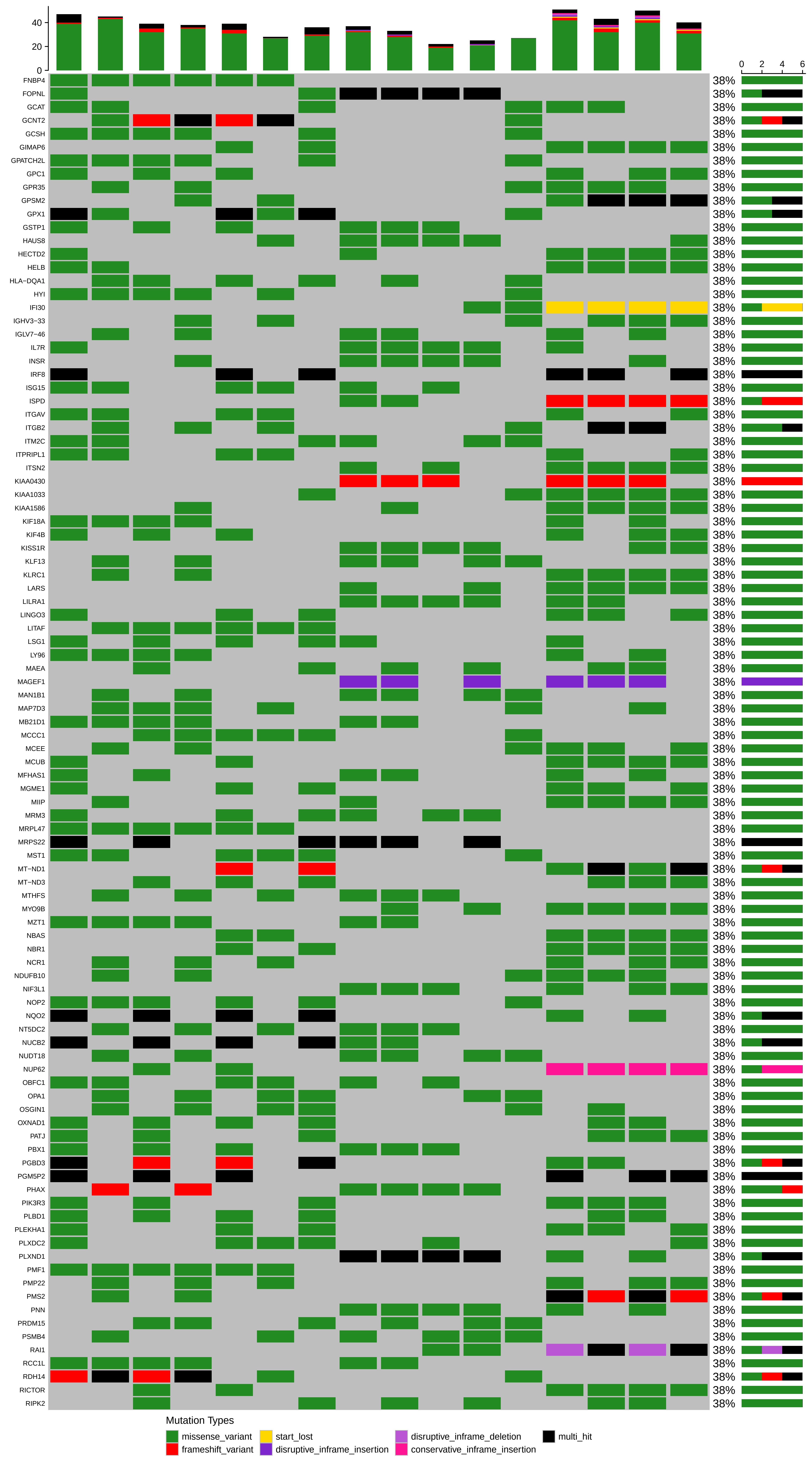

40
20
0
0
2
4
6
FNBP4
FOPNL
GCAT
GCNT2
GCSH
GIMAP6
GPATCH2L
GPC1
GPR35
GPSM2
GPX1
GSTP1
HAUS8
HECTD2
HELB
HLA−DQA1
HYI
IFI30
IGHV3−33
IGLV7−46
IL7R
INSR
IRF8
ISG15
ISPD
ITGAV
ITGB2
ITM2C
ITPRIPL1
ITSN2
KIAA0430
KIAA1033
KIAA1586
KIF18A
KIF4B
KISS1R
KLF13
KLRC1
LARS
LILRA1
LINGO3
LITAF
LSG1
LY96
MAEA
MAGEF1
MAN1B1
MAP7D3
MB21D1
MCCC1
MCEE
MCUB
MFHAS1
MGME1
MIIP
MRM3
MRPL47
MRPS22
MST1
MT−ND1
MT−ND3
MTHFS
MYO9B
MZT1
NBAS
NBR1
NCR1
NDUFB10
NIF3L1
NOP2
NQO2
NT5DC2
NUCB2
NUDT18
NUP62
OBFC1
OPA1
OSGIN1
OXNAD1
PATJ
PBX1
PGBD3
PGM5P2
PHAX
PIK3R3
PLBD1
PLEKHA1
PLXDC2
PLXND1
PMF1
PMP22
PMS2
PNN
PRDM15
PSMB4
RAI1
RCC1L
RDH14
RICTOR
RIPK2
38%
Mutation Types
missense_variant
frameshift_variant
start_lost
disruptive_inframe_insertion
disruptive_inframe_deletion
conservative_inframe_insertion
multi_hit

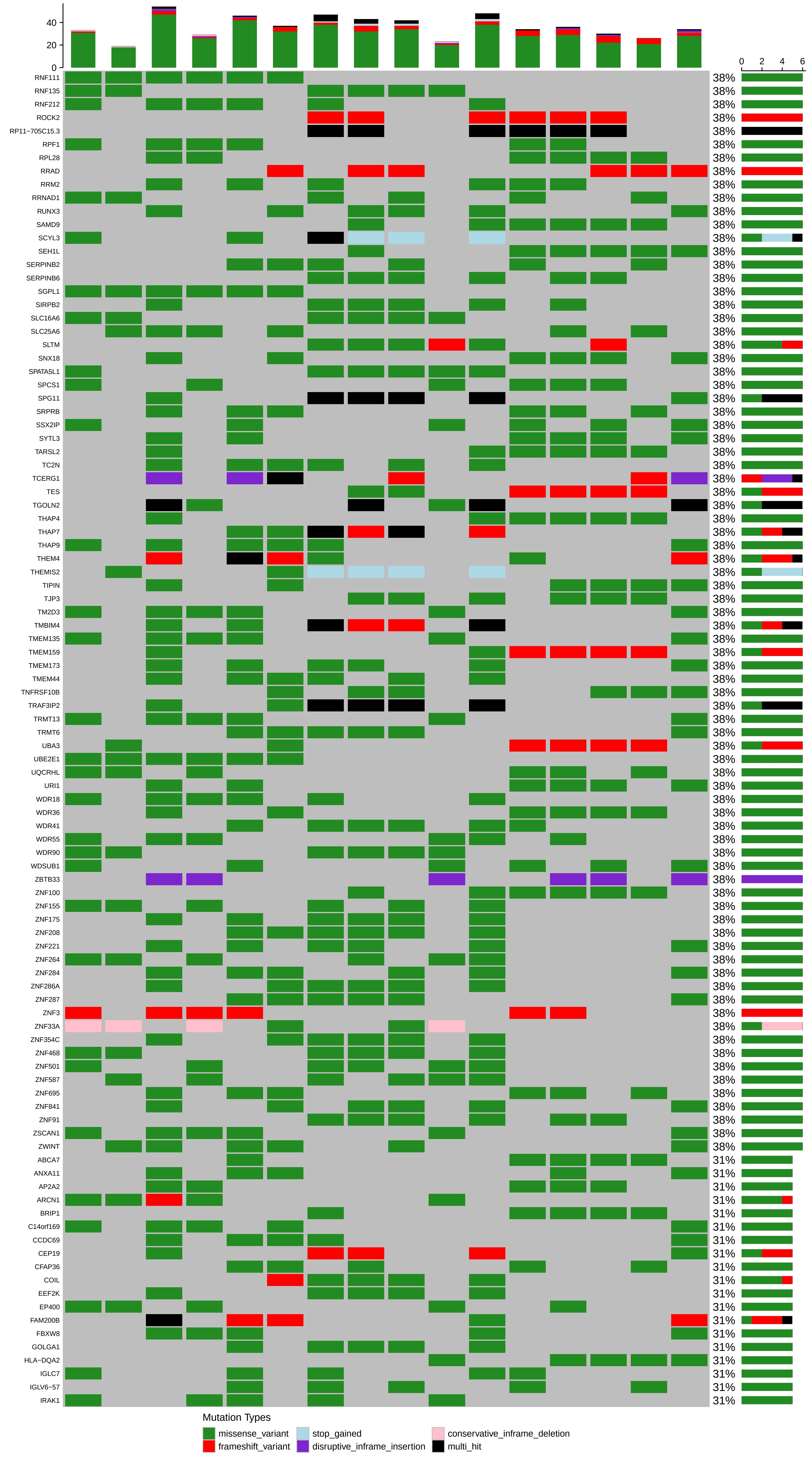
40
20
0
0 2 4 6
RNF111 38%
RNF135 38%
RNF212 38%
ROCK2 38%
RP11−705C15.3 38%
RPF1 38%
RPL28 38%
RRAD 38%
RRM2 38%
RRNAD1 38%
RUNX3 38%
SAMD9 38%
SCYL3 38%
SEH1L 38%
SERPINB2 38%
SERPINB6 38%
SGPL1 38%
SIRPB2 38%
SLC16A6 38%
SLC25A6 38%
SLTM 38%
SNX18 38%
SPATA5L1 38%
SPCS1 38%
SPG11 38%
SRPRB 38%
SSX2IP 38%
SYTL3 38%
TARSL2 38%
TC2N 38%
TCERG1 38%
TES 38%
TGOLN2 38%
THAP4 38%
THAP7 38%
THAP9 38%
THEM4 38%
THEMIS2 38%
TIPIN 38%
TJP3 38%
TM2D3 38%
TMBIM4 38%
TMEM135 38%
TMEM159 38%
TMEM173 38%
TMEM44 38%
TNFRSF10B 38%
TRAF3IP2 38%
TRMT13 38%
TRMT6 38%
UBA3 38%
UBE2E1 38%
UQCRHL 38%
URI1 38%
WDR18 38%
WDR36 38%
WDR41 38%
WDR55 38%
WDR90 38%
WDSUB1 38%
ZBTB33 38%
ZNF100 38%
ZNF155 38%
ZNF175 38%
ZNF208 38%
ZNF221 38%
ZNF264 38%
ZNF284 38%
ZNF286A 38%
ZNF287 38%
ZNF3 38%
ZNF33A 38%
ZNF354C 38%
ZNF468 38%
ZNF501 38%
ZNF587 38%
ZNF695 38%
ZNF841 38%
ZNF91 38%
ZSCAN1 38%
ZWINT 38%
ABCA7 31%
ANXA11 31%
AP2A2 31%
ARCN1 31%
BRIP1 31%
C14orf169 31%
CCDC69 31%
CEP19 31%
CFAP36 31%
COIL 31%
EEF2K 31%
EP400 31%
FAM200B 31%
FBXW8 31%
GOLGA1 31%
HLA−DQA2 31%
IGLC7 31%
IGLV6−57 31%
IRAK1 31%
Mutation Types
missense_variant
stop_gained
conservative_inframe_deletion
frameshift_variant
disruptive_inframe_insertion
multi_hit

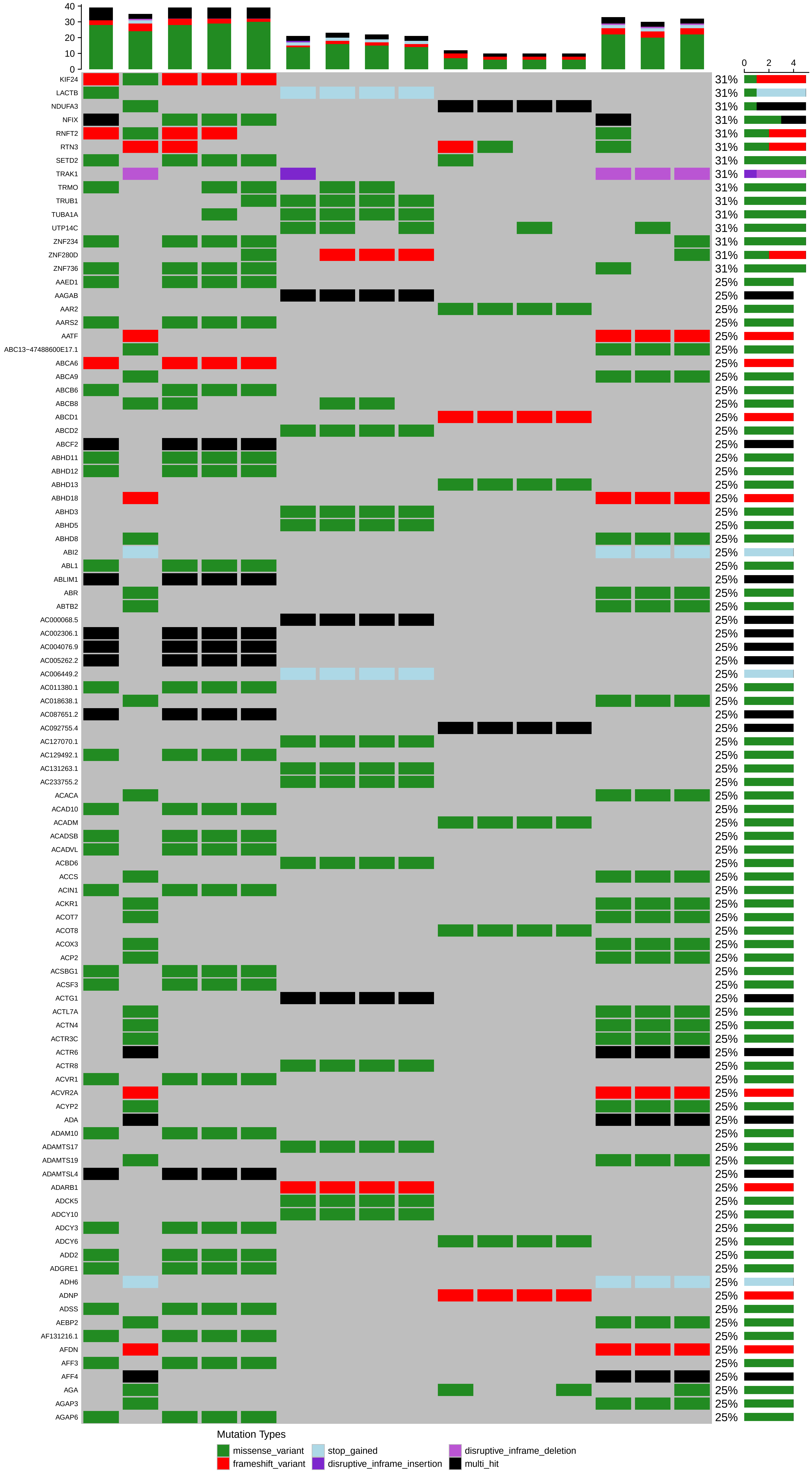

40
30
20
10
0
0
2
4
KIF24 31%
LACTB 31%
NDUFA3 31%
NFIX 31%
RNFT2 31%
RTN3 31%
SETD2 31%
TRAK1 31%
TRMO 31%
TRUB1 31%
TUBA1A 31%
UTP14C 31%
ZNF234 31%
ZNF280D 31%
ZNF736 31%
AAED1 25%
AAGAB 25%
AAR2 25%
AARS2 25%
AATF 25%
ABC13−47488600E17.1 25%
ABCA6 25%
ABCA9 25%
ABCB6 25%
ABCB8 25%
ABCD1 25%
ABCD2 25%
ABCF2 25%
ABHD11 25%
ABHD12 25%
ABHD13 25%
ABHD18 25%
ABHD3 25%
ABHD5 25%
ABHD8 25%
ABI2 25%
ABL1 25%
ABLIM1 25%
ABR 25%
ABTB2 25%
AC000068.5 25%
AC002306.1 25%
AC004076.9 25%
AC005262.2 25%
AC006449.2 25%
AC011380.1 25%
AC018638.1 25%
AC087651.2 25%
AC092755.4 25%
AC127070.1 25%
AC129492.1 25%
AC131263.1 25%
AC233755.2 25%
ACACA 25%
ACAD10 25%
ACADM 25%
ACADSB 25%
ACADVL 25%
ACBD6 25%
ACCS 25%
ACIN1 25%
ACKR1 25%
ACOT7 25%
ACOT8 25%
ACOX3 25%
ACP2 25%
ACSBG1 25%
ACSF3 25%
ACTG1 25%
ACTL7A 25%
ACTN4 25%
ACTR3C 25%
ACTR6 25%
ACTR8 25%
ACVR1 25%
ACVR2A 25%
ACYP2 25%
ADA 25%
ADAM10 25%
ADAMTS17 25%
ADAMTS19 25%
ADAMTSL4 25%
ADARB1 25%
ADCK5 25%
ADCY10 25%
ADCY3 25%
ADCY6 25%
ADD2 25%
ADGRE1 25%
ADH6 25%
ADNP 25%
ADSS 25%
AEBP2 25%
AF131216.1 25%
AFDN 25%
AFF3 25%
AFF4 25%
AGA 25%
AGAP3 25%
AGAP6 25%
Mutation Types
missense_variant
frameshift_variant
stop_gained
disruptive_inframe_insertion
disruptive_inframe_deletion
multi_hit

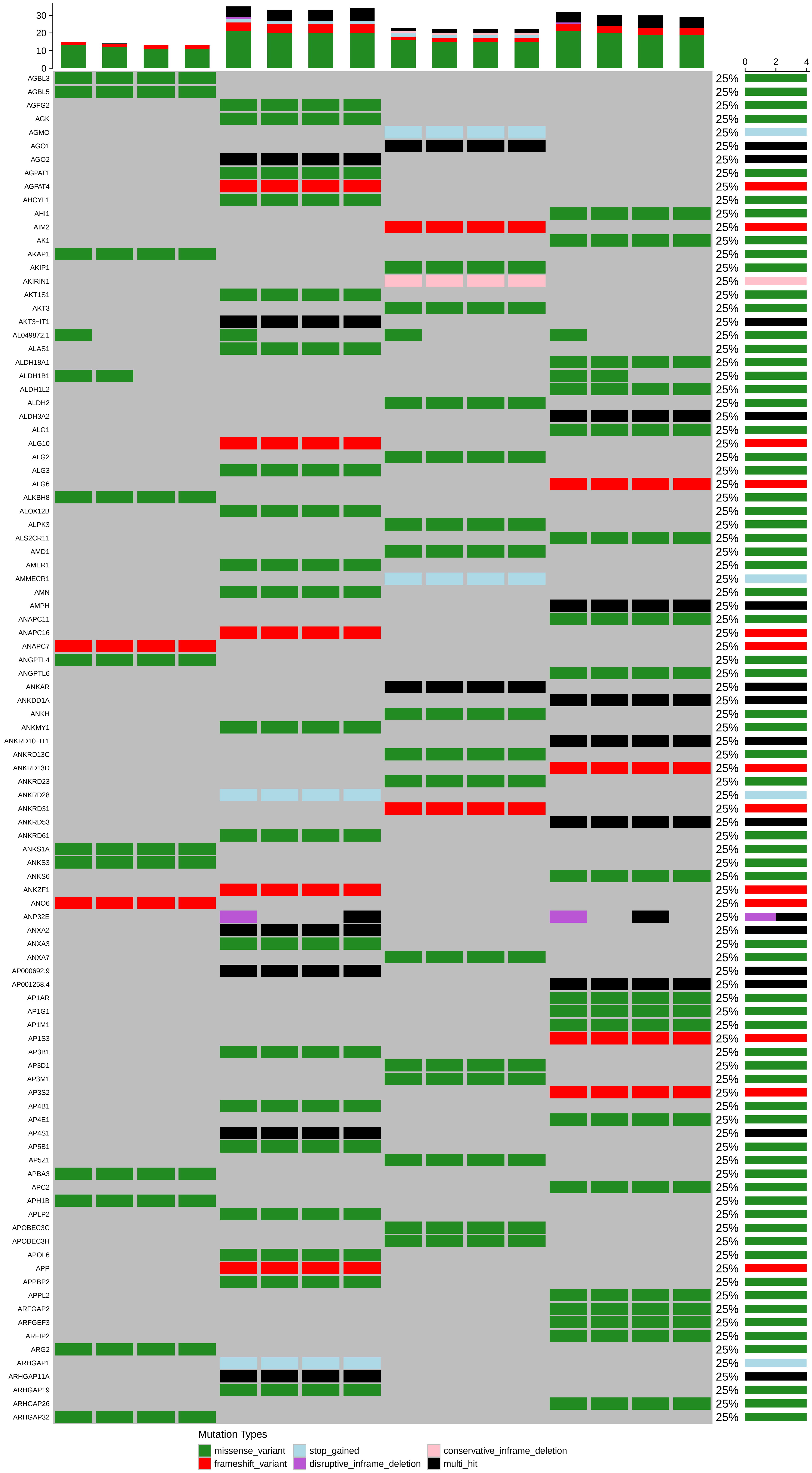

AGBL3
AGBL5
AGFG2
AGK
AGMO
AGO1
AGO2
AGPAT1
AGPAT4
AHCYL1
AHI1
AIM2
AK1
AKAP1
AKIP1
AKIRIN1
AKT1S1
AKT3
AKT3−IT1
AL049872.1
ALAS1
ALDH18A1
ALDH1B1
ALDH1L2
ALDH2
ALDH3A2
ALG1
ALG10
ALG2
ALG3
ALG6
ALKBH8
ALOX12B
ALPK3
ALS2CR11
AMD1
AMER1
AMMECR1
AMN
AMPH
ANAPC11
ANAPC16
ANAPC7
ANGPTL4
ANGPTL6
ANKAR
ANKDD1A
ANKH
ANKMY1
ANKRD10−IT1
ANKRD13C
ANKRD13D
ANKRD23
ANKRD28
ANKRD31
ANKRD53
ANKRD61
ANKS1A
ANKS3
ANKS6
ANKZF1
ANO6
ANP32E
ANXA2
ANXA3
ANXA7
AP000692.9
AP001258.4
AP1AR
AP1G1
AP1M1
AP1S3
AP3B1
AP3D1
AP3M1
AP3S2
AP4B1
AP4E1
AP4S1
AP5B1
AP5Z1
APBA3
APC2
APH1B
APLP2
APOBEC3C
APOBEC3H
APOL6
APP
APPBP2
APPL2
ARFGAP2
ARFGEF3
ARFIP2
ARG2
ARHGAP1
ARHGAP11A
ARHGAP19
ARHGAP26
ARHGAP32
25%
0
2
4
0
10
20
30
Mutation Types
missense_variant
frameshift_variant
stop_gained
disruptive_inframe_deletion
conservative_inframe_deletion
multi_hit

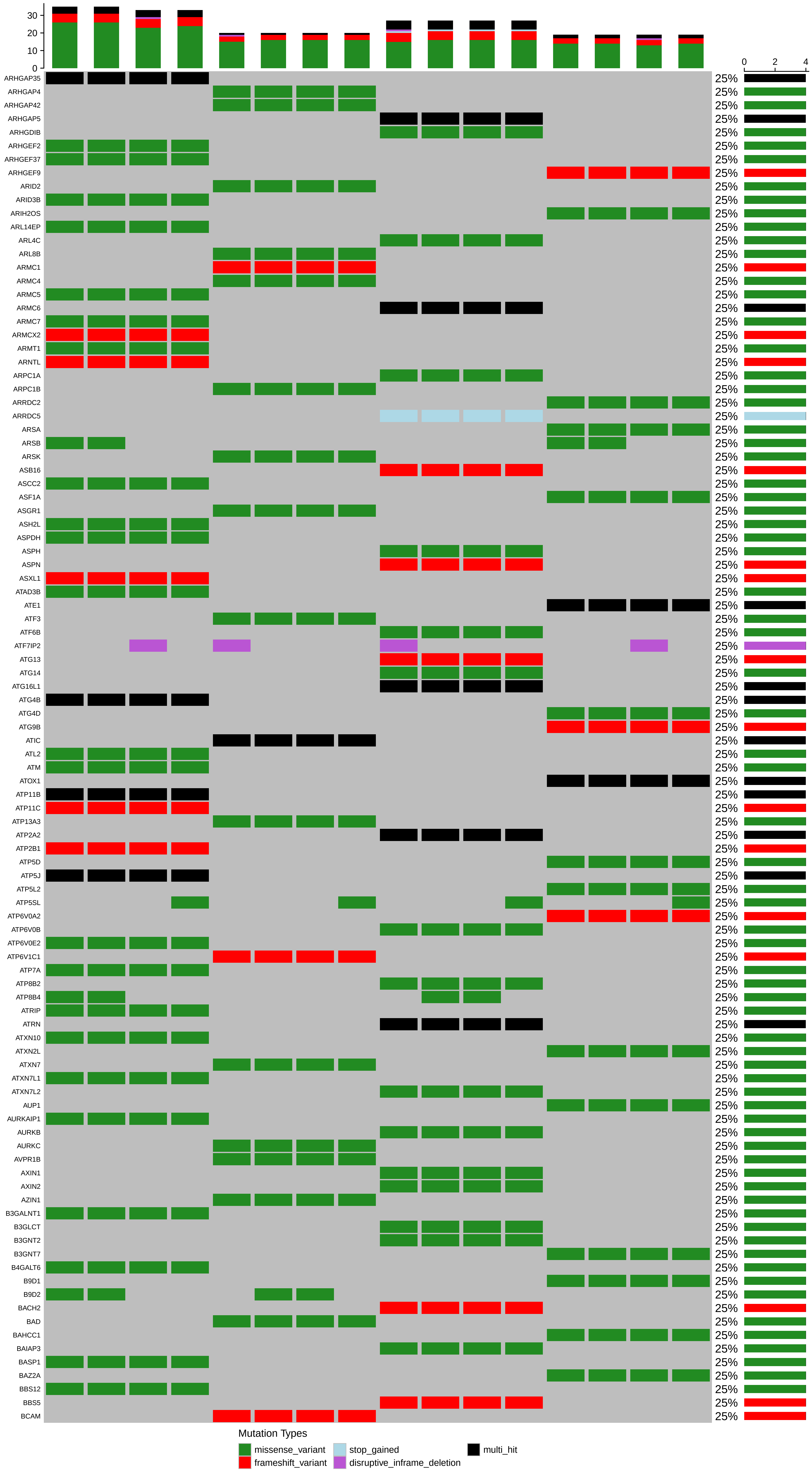

ARHGAP35
ARHGAP4
ARHGAP42
ARHGAP5
ARHGDIB
ARHGEF2
ARHGEF37
ARHGEF9
ARID2
ARID3B
ARIH2OS
ARL14EP
ARL4C
ARL8B
ARMC1
ARMC4
ARMC5
ARMC6
ARMC7
ARMCX2
ARMT1
ARNTL
ARPC1A
ARPC1B
ARRDC2
ARRDC5
ARSA
ARSB
ARSK
ASB16
ASCC2
ASF1A
ASGR1
ASH2L
ASPDH
ASPH
ASPN
ASXL1
ATAD3B
ATE1
ATF3
ATF6B
ATF7IP2
ATG13
ATG14
ATG16L1
ATG4B
ATG4D
ATG9B
ATIC
ATL2
ATM
ATOX1
ATP11B
ATP11C
ATP13A3
ATP2A2
ATP2B1
ATP5D
ATP5J
ATP5L2
ATP5SL
ATP6V0A2
ATP6V0B
ATP6V0E2
ATP6V1C1
ATP7A
ATP8B2
ATP8B4
ATRIP
ATRN
ATXN10
ATXN2L
ATXN7
ATXN7L1
ATXN7L2
AUP1
AURKAIP1
AURKB
AURKC
AVPR1B
AXIN1
AXIN2
AZIN1
B3GALNT1
B3GLCT
B3GNT2
B3GNT7
B4GALT6
B9D1
B9D2
BACH2
BAD
BAHCC1
BAIAP3
BASP1
BAZ2A
BBS12
BBS5
BCAM
Mutation Types
missense_variant
frameshift_variant
stop_gained
disruptive_inframe_deletion
multi_hit

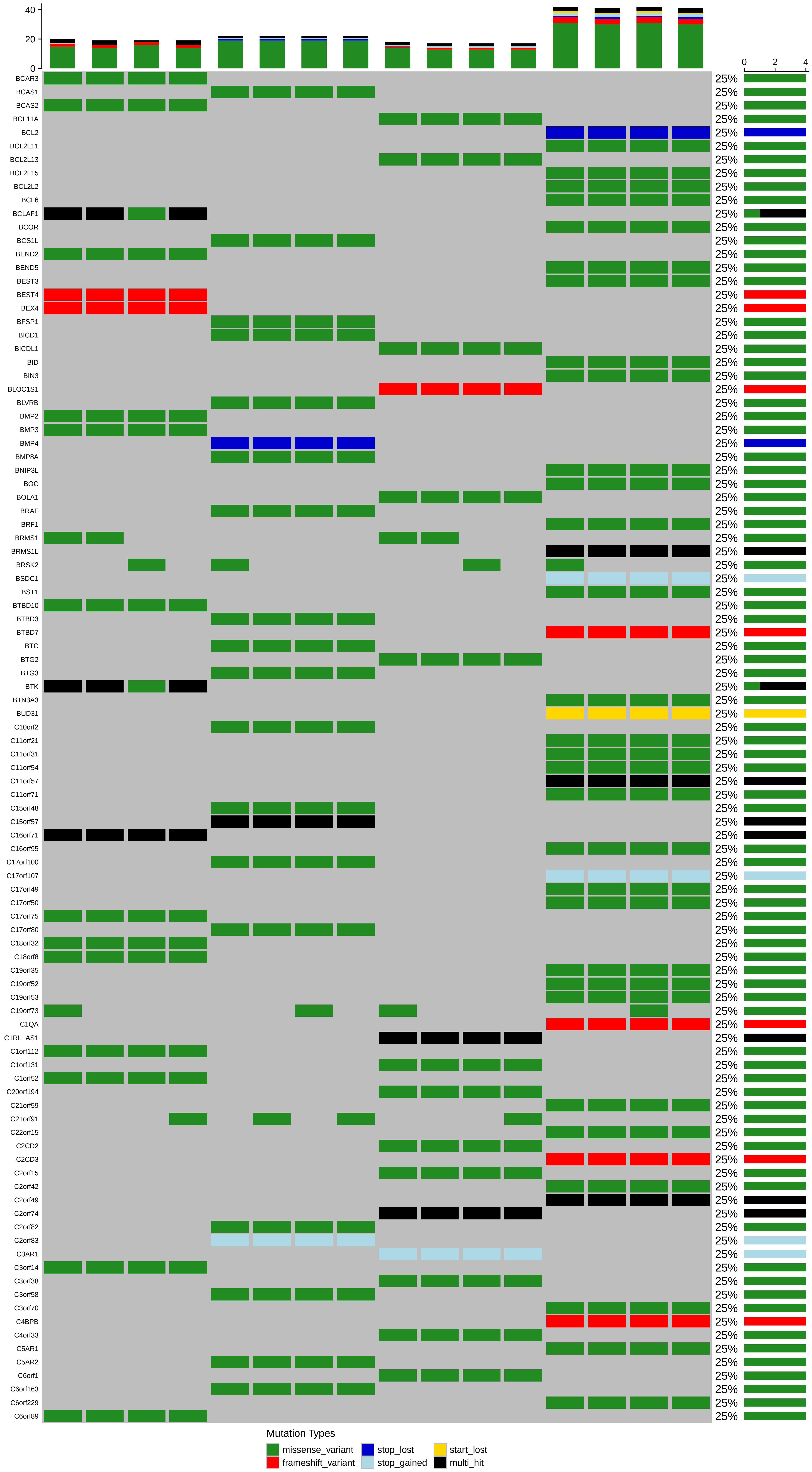

40
20
0
0
2
4
BCAR3 25%
BCAS1 25%
BCAS2 25%
BCL11A 25%
BCL2 25%
BCL2L11 25%
BCL2L13 25%
BCL2L15 25%
BCL2L2 25%
BCL6 25%
BCLAF1 25%
BCOR 25%
BCS1L 25%
BEND2 25%
BEND5 25%
BEST3 25%
BEST4 25%
BEX4 25%
BFSP1 25%
BICD1 25%
BICDL1 25%
BID 25%
BIN3 25%
BLOC1S1 25%
BLVRB 25%
BMP2 25%
BMP3 25%
BMP4 25%
BMP8A 25%
BNIP3L 25%
BOC 25%
BOLA1 25%
BRAF 25%
BRF1 25%
BRMS1 25%
BRMS1L 25%
BRSK2 25%
BSDC1 25%
BST1 25%
BTBD10 25%
BTBD3 25%
BTBD7 25%
BTC 25%
BTG2 25%
BTG3 25%
BTK 25%
BTN3A3 25%
BUD31 25%
C10orf2 25%
C11orf21 25%
C11orf31 25%
C11orf54 25%
C11orf57 25%
C11orf71 25%
C15orf48 25%
C15orf57 25%
C16orf71 25%
C16orf95 25%
C17orf100 25%
C17orf107 25%
C17orf49 25%
C17orf50 25%
C17orf75 25%
C17orf80 25%
C18orf32 25%
C18orf8 25%
C19orf35 25%
C19orf52 25%
C19orf53 25%
C19orf73 25%
C1QA 25%
C1RL−AS1 25%
C1orf112 25%
C1orf131 25%
C1orf52 25%
C20orf194 25%
C21orf59 25%
C21orf91 25%
C22orf15 25%
C2CD2 25%
C2CD3 25%
C2orf15 25%
C2orf42 25%
C2orf49 25%
C2orf74 25%
C2orf82 25%
C2orf83 25%
C3AR1 25%
C3orf14 25%
C3orf38 25%
C3orf58 25%
C3orf70 25%
C4BPB 25%
C4orf33 25%
C5AR1 25%
C5AR2 25%
C6orf1 25%
C6orf163 25%
C6orf229 25%
C6orf89 25%
Mutation Types
missense_variant
frameshift_variant
stop_lost
stop_gained
start_lost
multi_hit

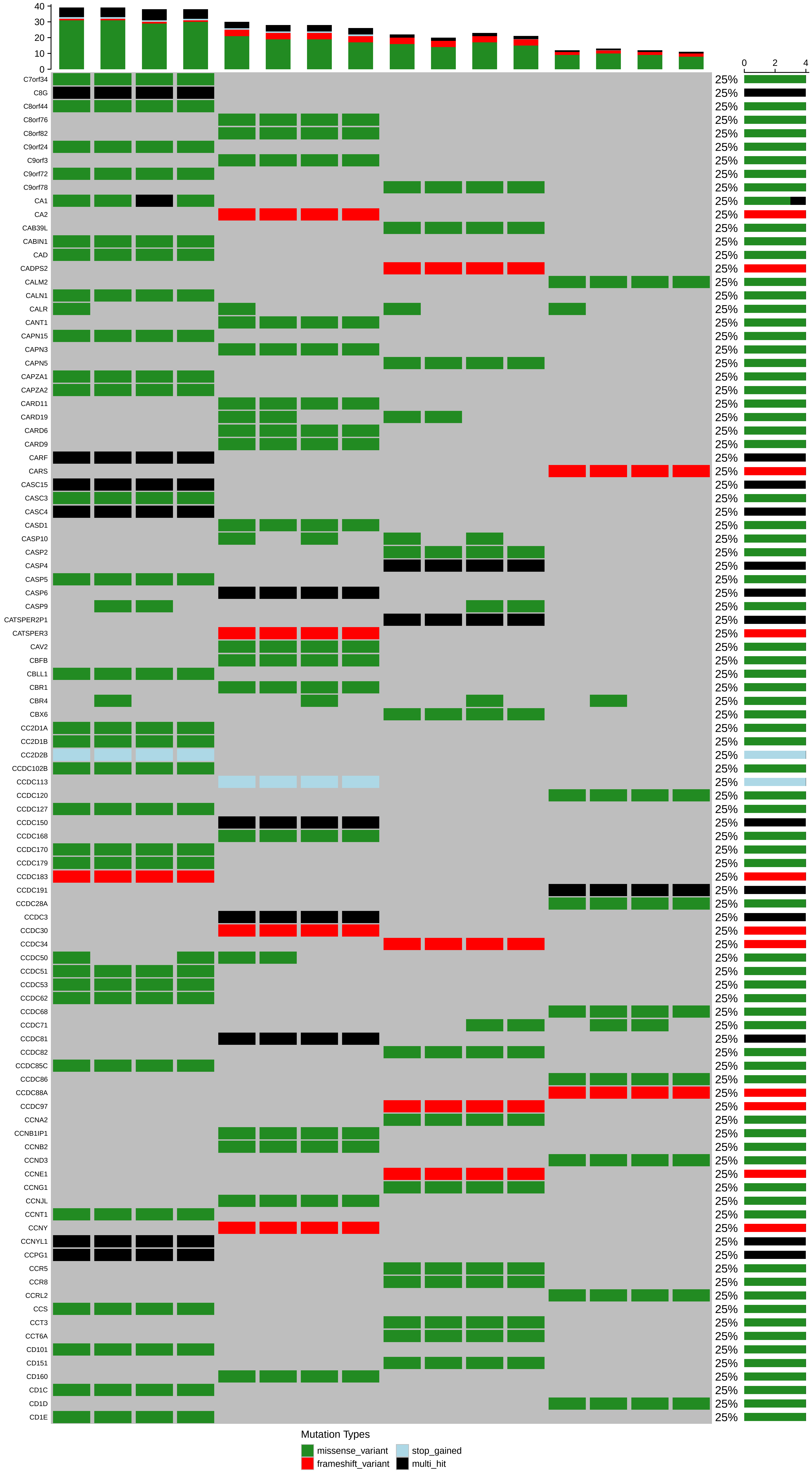

40
30
20
10
0
0
2
4
C7orf34
C8G
C8orf44
C8orf76
C8orf82
C9orf24
C9orf3
C9orf72
C9orf78
CA1
CA2
CAB39L
CABIN1
CAD
CADPS2
CALM2
CALN1
CALR
CANT1
CAPN15
CAPN3
CAPN5
CAPZA1
CAPZA2
CARD11
CARD19
CARD6
CARD9
CARF
CARS
CASC15
CASC3
CASC4
CASD1
CASP10
CASP2
CASP4
CASP5
CASP6
CASP9
CATSPER2P1
CATSPER3
CAV2
CBFB
CBLL1
CBR1
CBR4
CBX6
CC2D1A
CC2D1B
CC2D2B
CCDC102B
CCDC113
CCDC120
CCDC127
CCDC150
CCDC168
CCDC170
CCDC179
CCDC183
CCDC191
CCDC28A
CCDC3
CCDC30
CCDC34
CCDC50
CCDC51
CCDC53
CCDC62
CCDC68
CCDC71
CCDC81
CCDC82
CCDC85C
CCDC86
CCDC88A
CCDC97
CCNA2
CCNB1IP1
CCNB2
CCND3
CCNE1
CCNG1
CCNJL
CCNT1
CCNY
CCNYL1
CCPG1
CCR5
CCR8
CCRL2
CCS
CCT3
CCT6A
CD101
CD151
CD160
CD1C
CD1D
CD1E
25%
Mutation Types
missense_variant
frameshift_variant
stop_gained
multi_hit

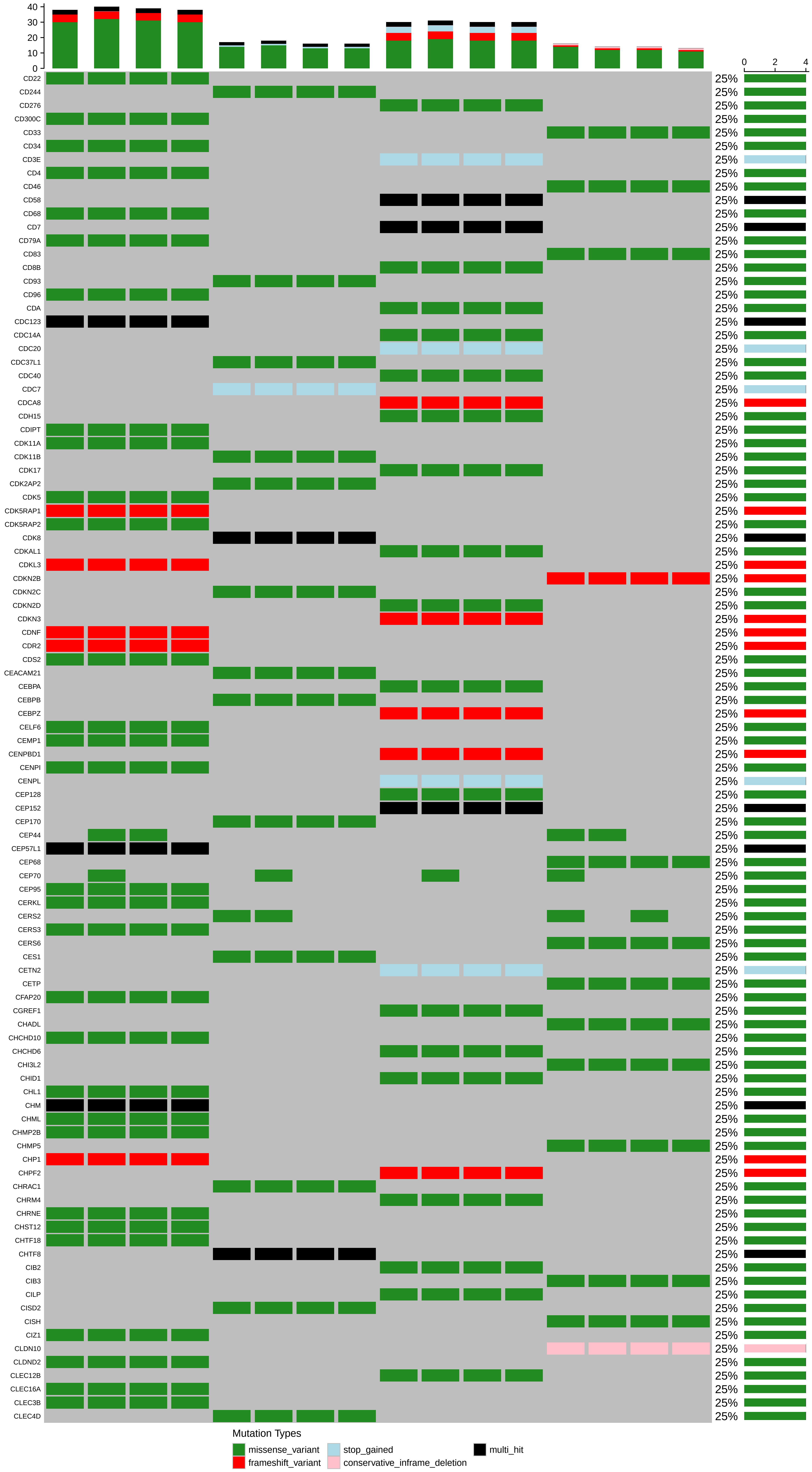

40
30
20
10
0
0
2
4
CD22
CD244
CD276
CD300C
CD33
CD34
CD3E
CD4
CD46
CD58
CD68
CD7
CD79A
CD83
CD8B
CD93
CD96
CDA
CDC123
CDC14A
CDC20
CDC37L1
CDC40
CDC7
CDCA8
CDH15
CDIPT
CDK11A
CDK11B
CDK17
CDK2AP2
CDK5
CDK5RAP1
CDK5RAP2
CDK8
CDKAL1
CDKL3
CDKN2B
CDKN2C
CDKN2D
CDKN3
CDNF
CDR2
CDS2
CEACAM21
CEBPA
CEBPB
CEBPZ
CELF6
CEMP1
CENPBD1
CENPI
CENPL
CEP128
CEP152
CEP170
CEP44
CEP57L1
CEP68
CEP70
CEP95
CERKL
CERS2
CERS3
CERS6
CES1
CETN2
CETP
CFAP20
CGREF1
CHADL
CHCHD10
CHCHD6
CHI3L2
CHID1
CHL1
CHM
CHML
CHMP2B
CHMP5
CHP1
CHPF2
CHRAC1
CHRM4
CHRNE
CHST12
CHTF18
CHTF8
CIB2
CIB3
CILP
CISD2
CISH
CIZ1
CLDN10
CLDND2
CLEC12B
CLEC16A
CLEC3B
CLEC4D
25%
Mutation Types
missense_variant
frameshift_variant
stop_gained
conservative_inframe_deletion
multi_hit

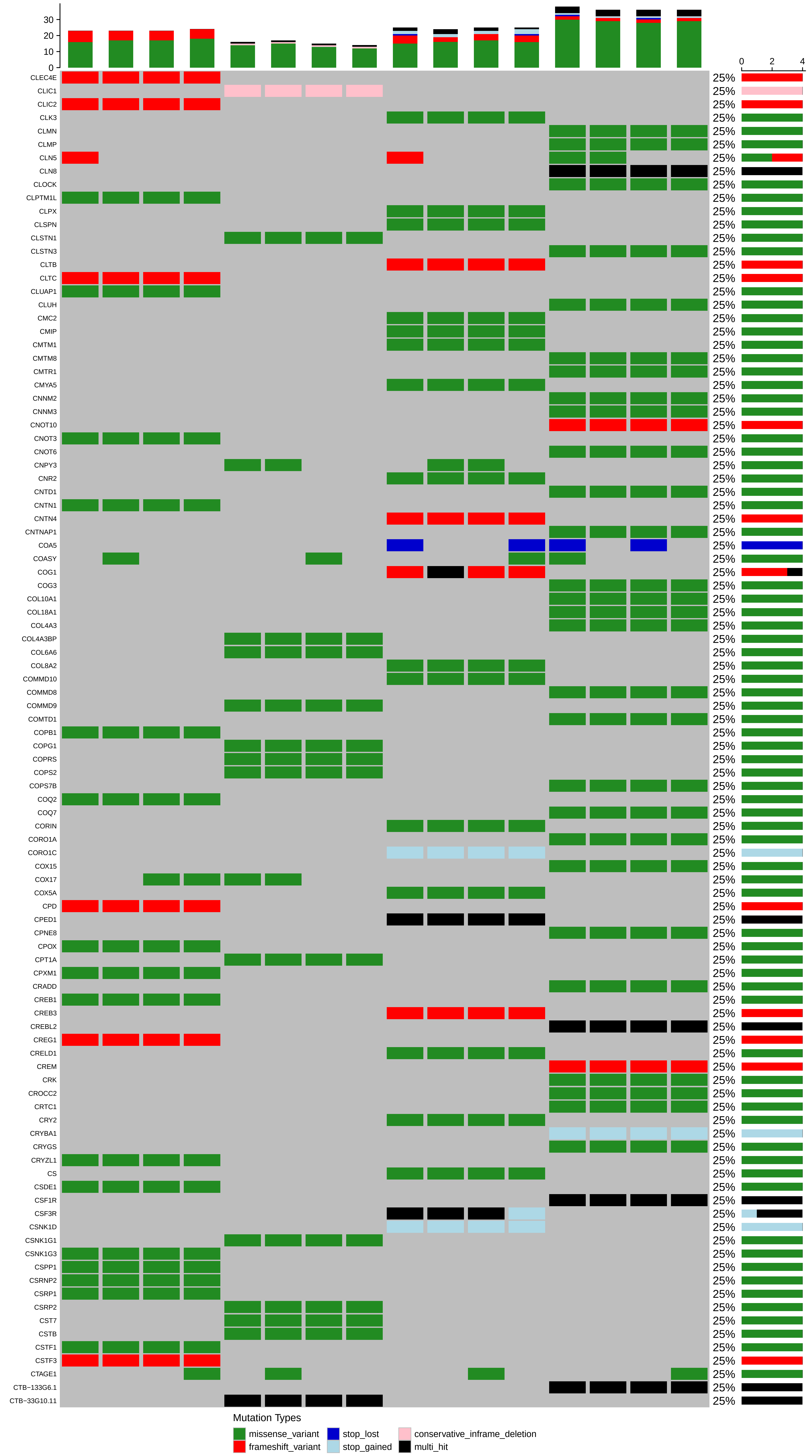

30
20
10
0
CLEC4E
CLIC1
CLIC2
CLK3
CLMN
CLMP
CLN5
CLN8
CLOCK
CLPTM1L
CLPX
CLSPN
CLSTN1
CLSTN3
CLTB
CLTC
CLUAP1
CLUH
CMC2
CMIP
CMTM1
CMTM8
CMTR1
CMYA5
CNNM2
CNNM3
CNOT10
CNOT3
CNOT6
CNPY3
CNR2
CNTD1
CNTN1
CNTN4
CNTNAP1
COA5
COASY
COG1
COG3
COL10A1
COL18A1
COL4A3
COL4A3BP
COL6A6
COL8A2
COMMD10
COMMD8
COMMD9
COMTD1
COPB1
COPG1
COPRS
COPS2
COPS7B
COQ2
COQ7
CORIN
CORO1A
CORO1C
COX15
COX17
COX5A
CPD
CPED1
CPNE8
CPOX
CPT1A
CPXM1
CRADD
CREB1
CREB3
CREBL2
CREG1
CRELD1
CREM
CRK
CROCC2
CRTC1
CRY2
CRYBA1
CRYGS
CRYZL1
CS
CSDE1
CSF1R
CSF3R
CSNK1D
CSNK1G1
CSNK1G3
CSPP1
CSRNP2
CSRP1
CSRP2
CST7
CSTB
CSTF1
CSTF3
CTAGE1
CTB−133G6.1
CTB−33G10.11
25%
0
2
4
Mutation Types
missense_variant
frameshift_variant
stop_lost
stop_gained
conservative_inframe_deletion
multi_hit

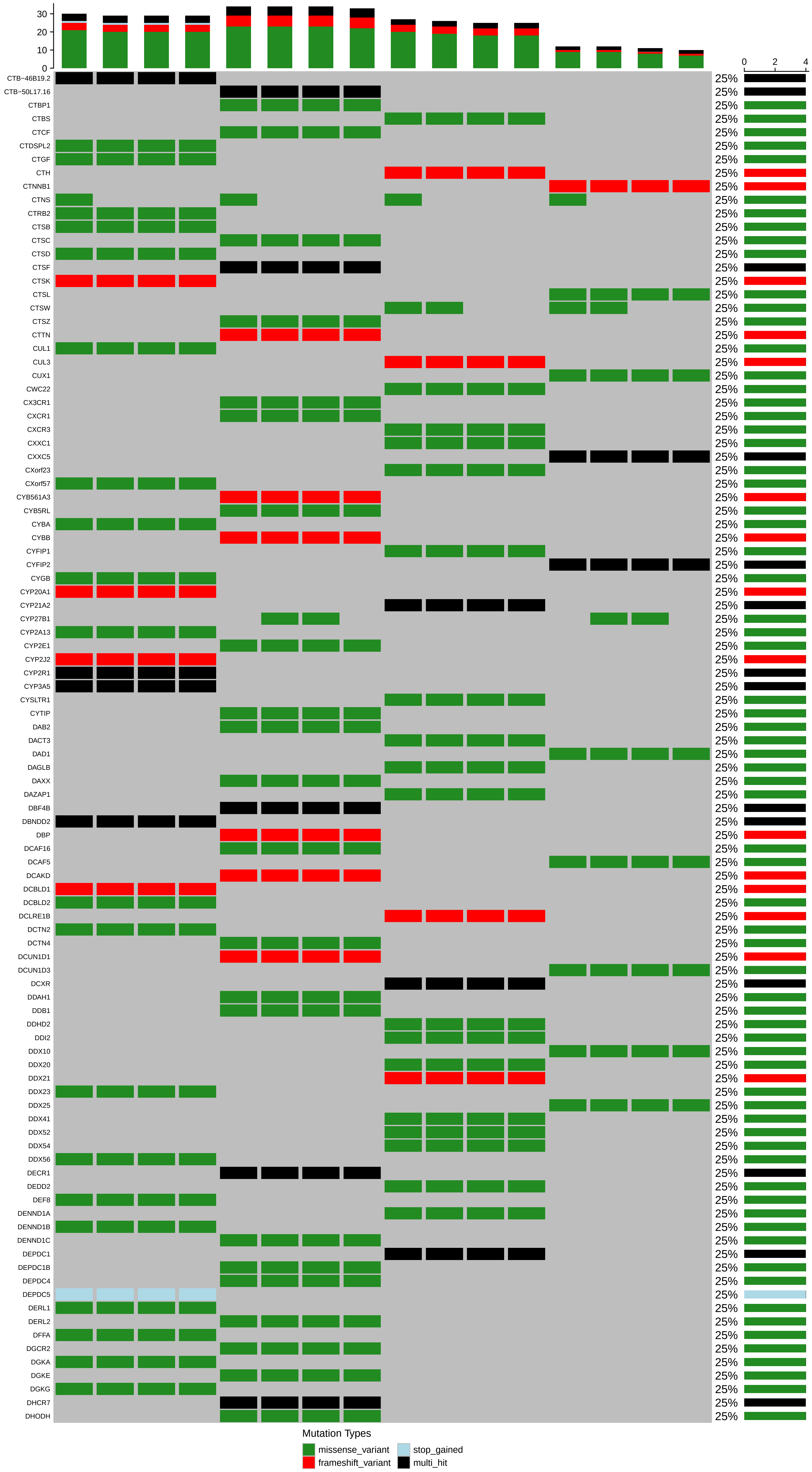

30
20
10
0
0
2
4
CTB−46B19.2
CTB−50L17.16
CTBP1
CTBS
CTCF
CTDSPL2
CTGF
CTH
CTNNB1
CTNS
CTRB2
CTSB
CTSC
CTSD
CTSF
CTSK
CTSL
CTSW
CTSZ
CTTN
CUL1
CUL3
CUX1
CWC22
CX3CR1
CXCR1
CXCR3
CXXC1
CXXC5
CXorf23
CXorf57
CYB561A3
CYB5RL
CYBA
CYBB
CYFIP1
CYFIP2
CYGB
CYP20A1
CYP21A2
CYP27B1
CYP2A13
CYP2E1
CYP2J2
CYP2R1
CYP3A5
CYSLTR1
CYTIP
DAB2
DACT3
DAD1
DAGLB
DAXX
DAZAP1
DBF4B
DBNDD2
DBP
DCAF16
DCAF5
DCAKD
DCBLD1
DCBLD2
DCLRE1B
DCTN2
DCTN4
DCUN1D1
DCUN1D3
DCXR
DDAH1
DDB1
DDHD2
DDI2
DDX10
DDX20
DDX21
DDX23
DDX25
DDX41
DDX52
DDX54
DDX56
DECR1
DEDD2
DEF8
DENND1A
DENND1B
DENND1C
DEPDC1
DEPDC1B
DEPDC4
DEPDC5
DERL1
DERL2
DFFA
DGCR2
DGKA
DGKE
DGKG
DHCR7
DHODH
25%
Mutation Types
missense_variant
frameshift_variant
stop_gained
multi_hit

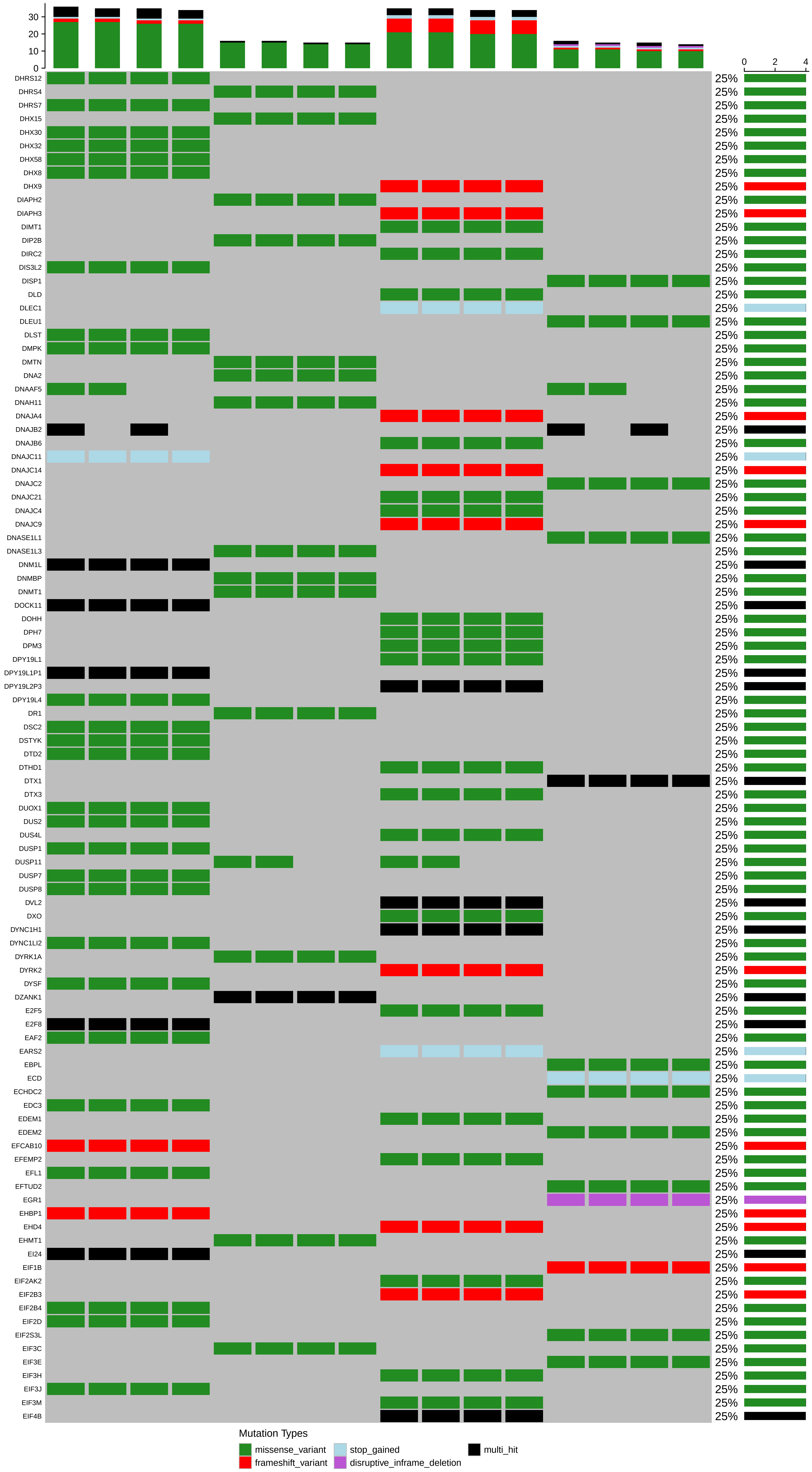

30
20
10
0
0
2
4
DHRS12
DHRS4
DHRS7
DHX15
DHX30
DHX32
DHX58
DHX8
DHX9
DIAPH2
DIAPH3
DIMT1
DIP2B
DIRC2
DIS3L2
DISP1
DLD
DLEC1
DLEU1
DLST
DMPK
DMTN
DNA2
DNAAF5
DNAH11
DNAJA4
DNAJB2
DNAJB6
DNAJC11
DNAJC14
DNAJC2
DNAJC21
DNAJC4
DNAJC9
DNASE1L1
DNASE1L3
DNM1L
DNMBP
DNMT1
DOCK11
DOHH
DPH7
DPM3
DPY19L1
DPY19L1P1
DPY19L2P3
DPY19L4
DR1
DSC2
DSTYK
DTD2
DTHD1
DTX1
DTX3
DUOX1
DUS2
DUS4L
DUSP1
DUSP11
DUSP7
DUSP8
DVL2
DXO
DYNC1H1
DYNC1LI2
DYRK1A
DYRK2
DYSF
DZANK1
E2F5
E2F8
EAF2
EARS2
EBPL
ECD
ECHDC2
EDC3
EDEM1
EDEM2
EFCAB10
EFEMP2
EFL1
EFTUD2
EGR1
EHBP1
EHD4
EHMT1
EI24
EIF1B
EIF2AK2
EIF2B3
EIF2B4
EIF2D
EIF2S3L
EIF3C
EIF3E
EIF3H
EIF3J
EIF3M
EIF4B
25%
Mutation Types
missense_variant
frameshift_variant
stop_gained
disruptive_inframe_deletion
multi_hit

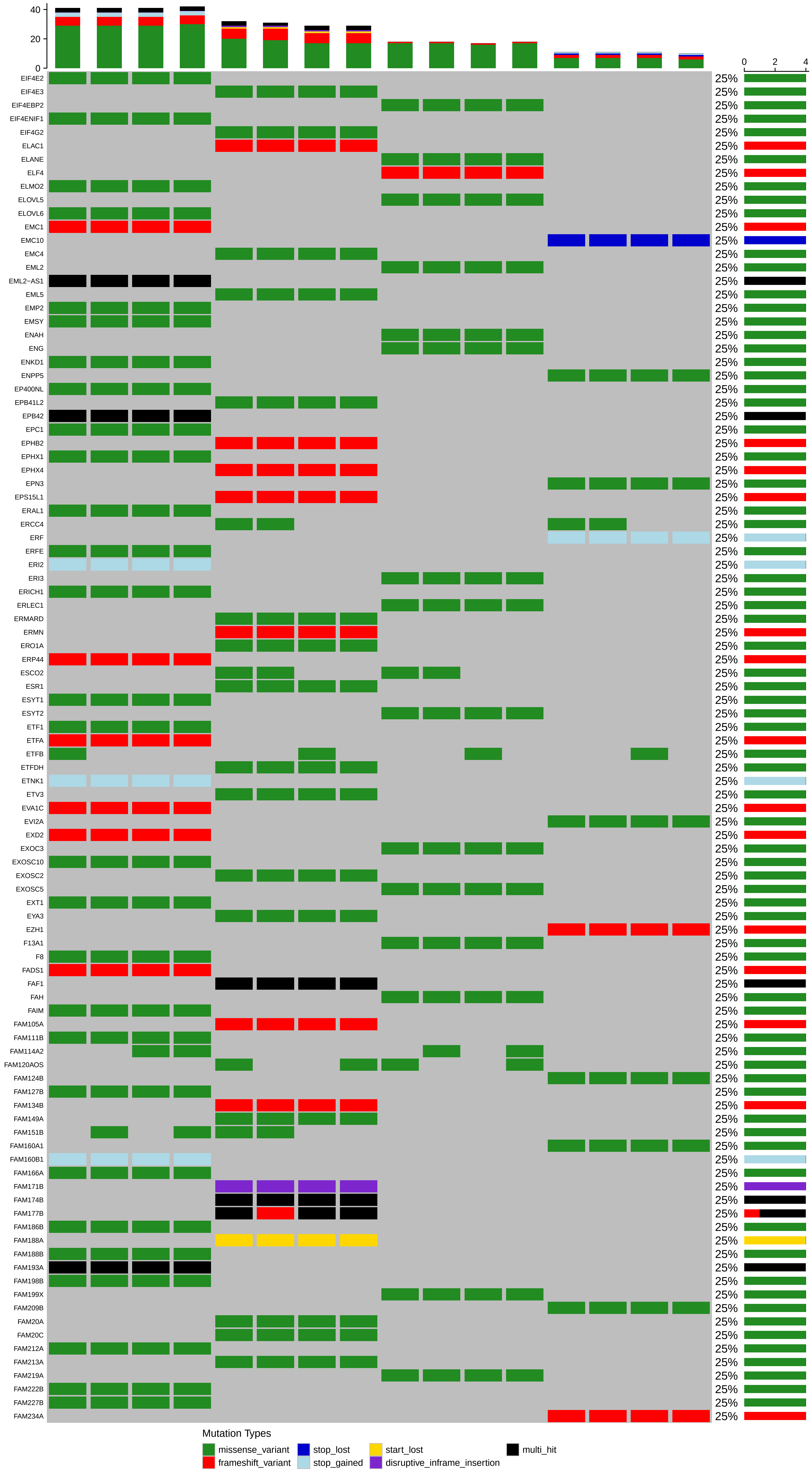

40
20
0
0 2 4
EIF4E2
EIF4E3
EIF4EBP2
EIF4ENIF1
EIF4G2
ELAC1
ELANE
ELF4
ELMO2
ELOVL5
ELOVL6
EMC1
EMC10
EMC4
EML2
EML2−AS1
EML5
EMP2
EMSY
ENAH
ENG
ENKD1
ENPP5
EP400NL
EPB41L2
EPB42
EPC1
EPHB2
EPHX1
EPHX4
EPN3
EPS15L1
ERAL1
ERCC4
ERF
ERFE
ERI2
ERI3
ERICH1
ERLEC1
ERMARD
ERMN
ERO1A
ERP44
ESCO2
ESR1
ESYT1
ESYT2
ETF1
ETFA
ETFB
ETFDH
ETNK1
ETV3
EVA1C
EVI2A
EXD2
EXOC3
EXOSC10
EXOSC2
EXOSC5
EXT1
EYA3
EZH1
F13A1
F8
FADS1
FAF1
FAH
FAIM
FAM105A
FAM111B
FAM114A2
FAM120AOS
FAM124B
FAM127B
FAM134B
FAM149A
FAM151B
FAM160A1
FAM160B1
FAM166A
FAM171B
FAM174B
FAM177B
FAM186B
FAM188A
FAM188B
FAM193A
FAM198B
FAM199X
FAM209B
FAM20A
FAM20C
FAM212A
FAM213A
FAM219A
FAM222B
FAM227B
FAM234A
25%
Mutation Types
missense_variant
frameshift_variant
stop_lost
stop_gained
start_lost
disruptive_inframe_insertion
multi_hit

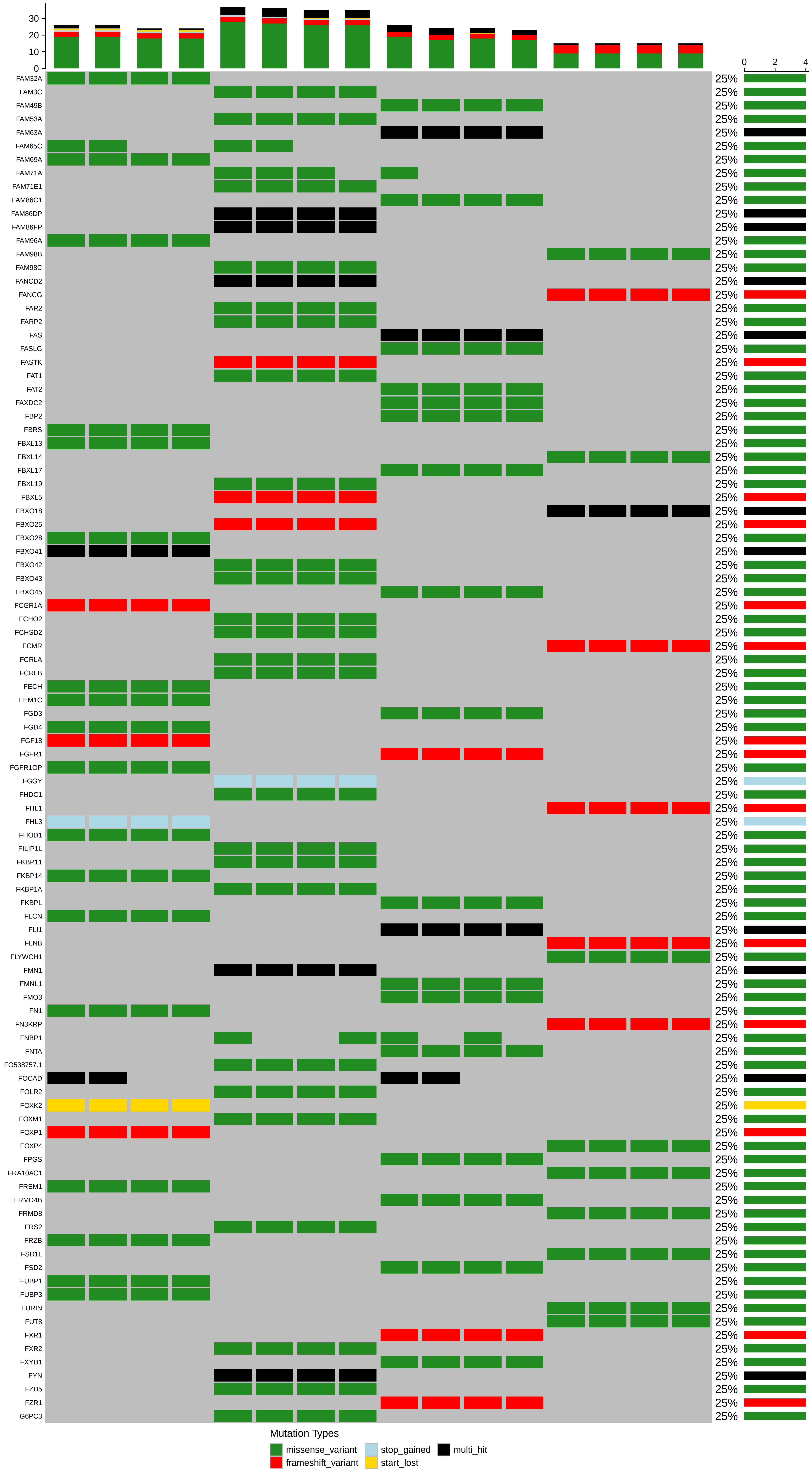

30
20
10
0
0
2
4
FAM32A
FAM3C
FAM49B
FAM53A
FAM63A
FAM65C
FAM69A
FAM71A
FAM71E1
FAM86C1
FAM86DP
FAM86FP
FAM96A
FAM98B
FAM98C
FANCD2
FANCG
FAR2
FARP2
FAS
FASLG
FASTK
FAT1
FAT2
FAXDC2
FBP2
FBRS
FBXL13
FBXL14
FBXL17
FBXL19
FBXL5
FBXO18
FBXO25
FBXO28
FBXO41
FBXO42
FBXO43
FBXO45
FCGR1A
FCHO2
FCHSD2
FCMR
FCRLA
FCRLB
FECH
FEM1C
FGD3
FGD4
FGF18
FGFR1
FGFR1OP
FGGY
FHDC1
FHL1
FHL3
FHOD1
FILIP1L
FKBP11
FKBP14
FKBP1A
FKBPL
FLCN
FLI1
FLNB
FLYWCH1
FMN1
FMNL1
FMO3
FN1
FN3KRP
FNBP1
FNTA
FO538757.1
FOCAD
FOLR2
FOXK2
FOXM1
FOXP1
FOXP4
FPGS
FRA10AC1
FREM1
FRMD4B
FRMD8
FRS2
FRZB
FSD1L
FSD2
FUBP1
FUBP3
FURIN
FUT8
FXR1
FXR2
FXYD1
FYN
FZD5
FZR1
G6PC3
25%
Mutation Types
missense_variant
frameshift_variant
stop_gained
start_lost
multi_hit

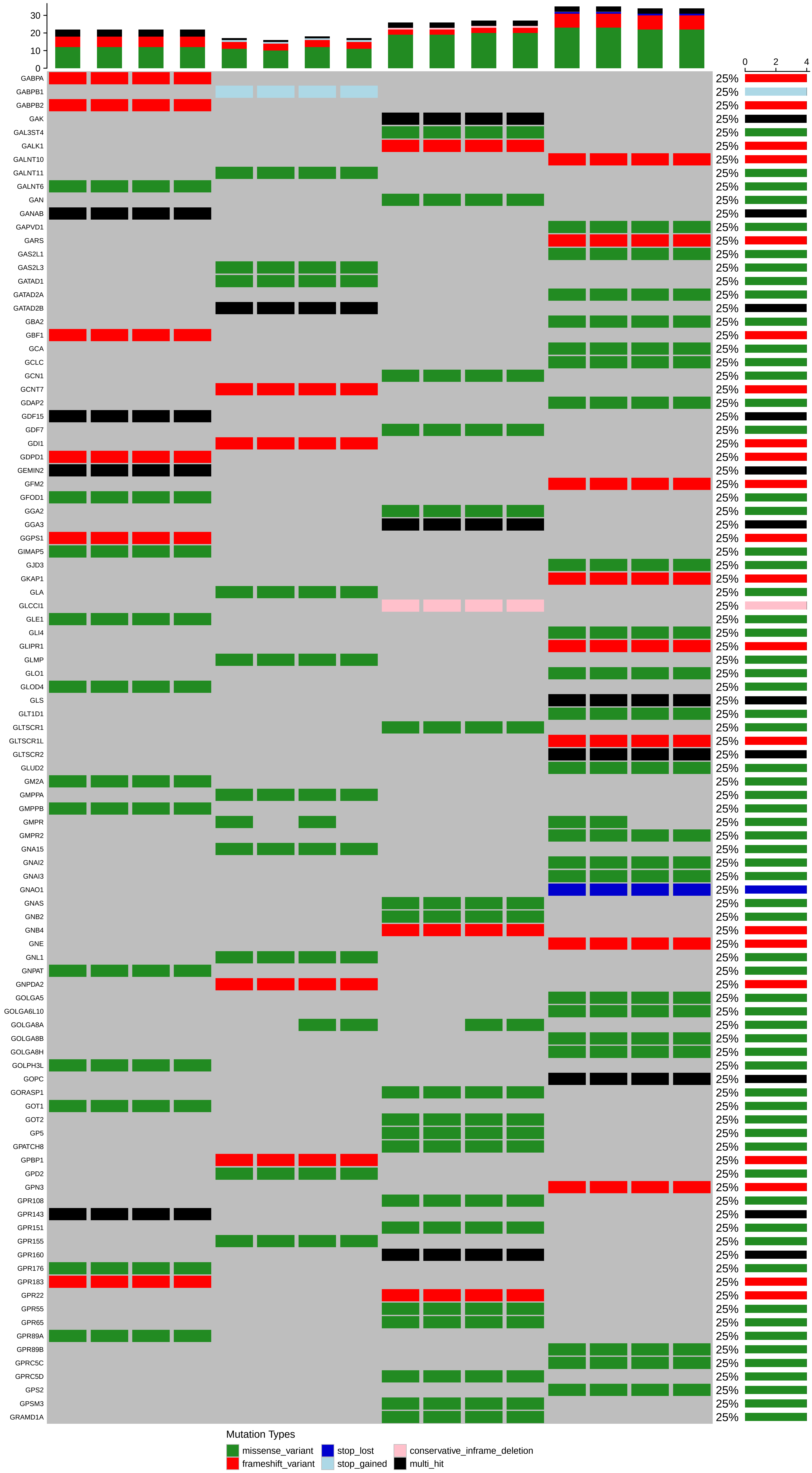
30
20
10
0
0
2
4
GABPA
GABPB1
GABPB2
GAK
GAL3ST4
GALK1
GALNT10
GALNT11
GALNT6
GAN
GANAB
GAPVD1
GARS
GAS2L1
GAS2L3
GATAD1
GATAD2A
GATAD2B
GBA2
GBF1
GCA
GCLC
GCN1
GCNT7
GDAP2
GDF15
GDF7
GDI1
GDPD1
GEMIN2
GFM2
GFOD1
GGA2
GGA3
GGPS1
GIMAP5
GJD3
GKAP1
GLA
GLCCI1
GLE1
GLI4
GLIPR1
GLMP
GLO1
GLOD4
GLS
GLT1D1
GLTSCR1
GLTSCR1L
GLTSCR2
GLUD2
GM2A
GMPPA
GMPPB
GMPR
GMPR2
GNA15
GNAI2
GNAI3
GNAO1
GNAS
GNB2
GNB4
GNE
GNL1
GNPAT
GNPDA2
GOLGA5
GOLGA6L10
GOLGA8A
GOLGA8B
GOLGA8H
GOLPH3L
GOPC
GORASP1
GOT1
GOT2
GP5
GPATCH8
GPBP1
GPD2
GPN3
GPR108
GPR143
GPR151
GPR155
GPR160
GPR176
GPR183
GPR22
GPR55
GPR65
GPR89A
GPR89B
GPRC5C
GPRC5D
GPS2
GPSM3
GRAMD1A
25%
Mutation Types
missense_variant
frameshift_variant
stop_lost
stop_gained
conservative_inframe_deletion
multi_hit

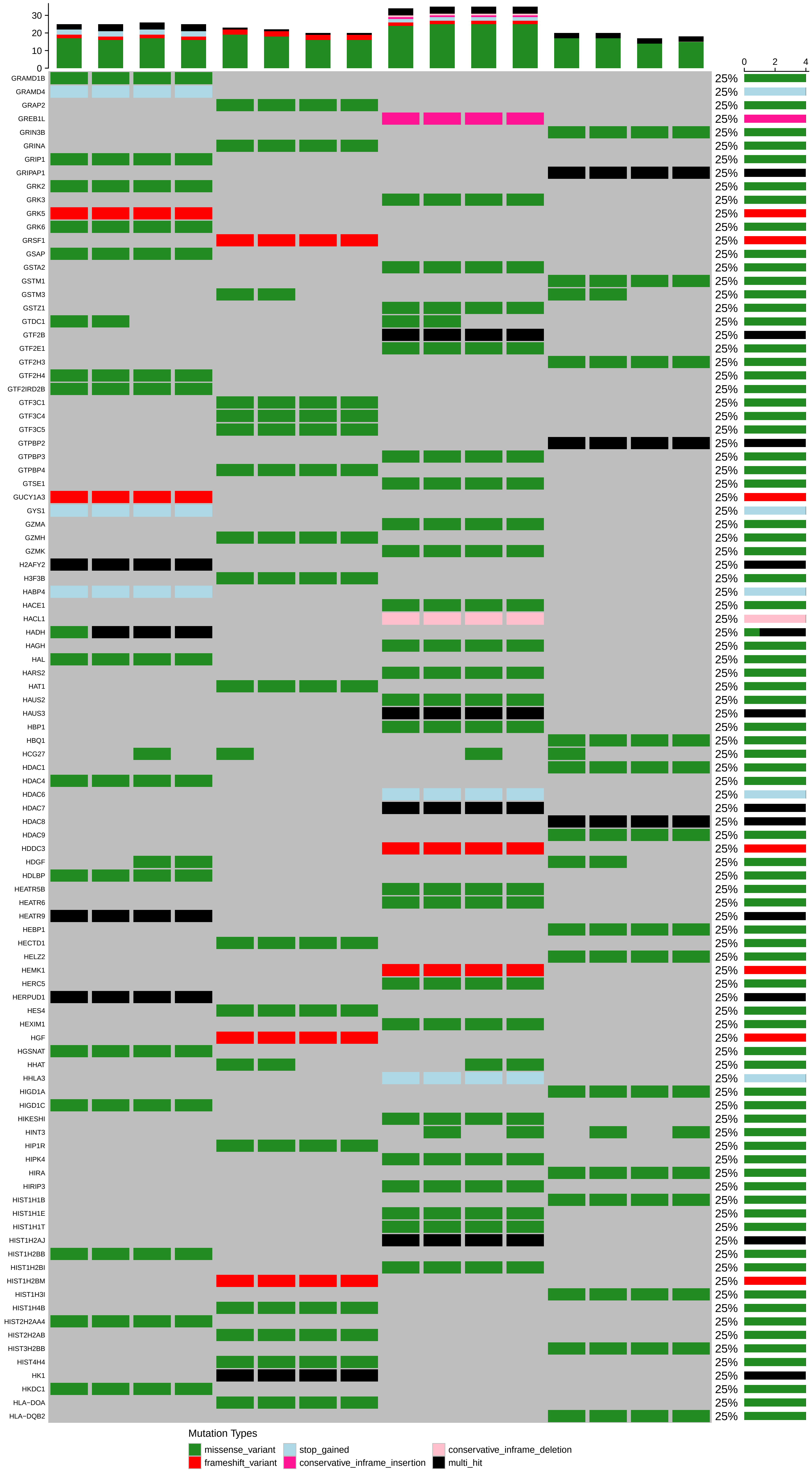
30
20
10
0
0
2
4
GRAMD1B
GRAMD4
GRAP2
GREB1L
GRIN3B
GRINA
GRIP1
GRIPAP1
GRK2
GRK3
GRK5
GRK6
GRSF1
GSAP
GSTA2
GSTM1
GSTM3
GSTZ1
GTDC1
GTF2B
GTF2E1
GTF2H3
GTF2H4
GTF2IRD2B
GTF3C1
GTF3C4
GTF3C5
GTPBP2
GTPBP3
GTPBP4
GTSE1
GUCY1A3
GYS1
GZMA
GZMH
GZMK
H2AFY2
H3F3B
HABP4
HACE1
HACL1
HADH
HAGH
HAL
HARS2
HAT1
HAUS2
HAUS3
HBP1
HBQ1
HCG27
HDAC1
HDAC4
HDAC6
HDAC7
HDAC8
HDAC9
HDDC3
HDGF
HDLBP
HEATR5B
HEATR6
HEATR9
HEBP1
HECTD1
HELZ2
HEMK1
HERC5
HERPUD1
HES4
HEXIM1
HGF
HGSNAT
HHAT
HHLA3
HIGD1A
HIGD1C
HIKESHI
HINT3
HIP1R
HIPK4
HIRA
HIRIP3
HIST1H1B
HIST1H1E
HIST1H1T
HIST1H2AJ
HIST1H2BB
HIST1H2BI
HIST1H2BM
HIST1H3I
HIST1H4B
HIST2H2AA4
HIST2H2AB
HIST3H2BB
HIST4H4
HK1
HKDC1
HLA−DOA
HLA−DQB2
25%
Mutation Types
missense_variant
frameshift_variant
stop_gained
conservative_inframe_insertion
conservative_inframe_deletion
multi_hit

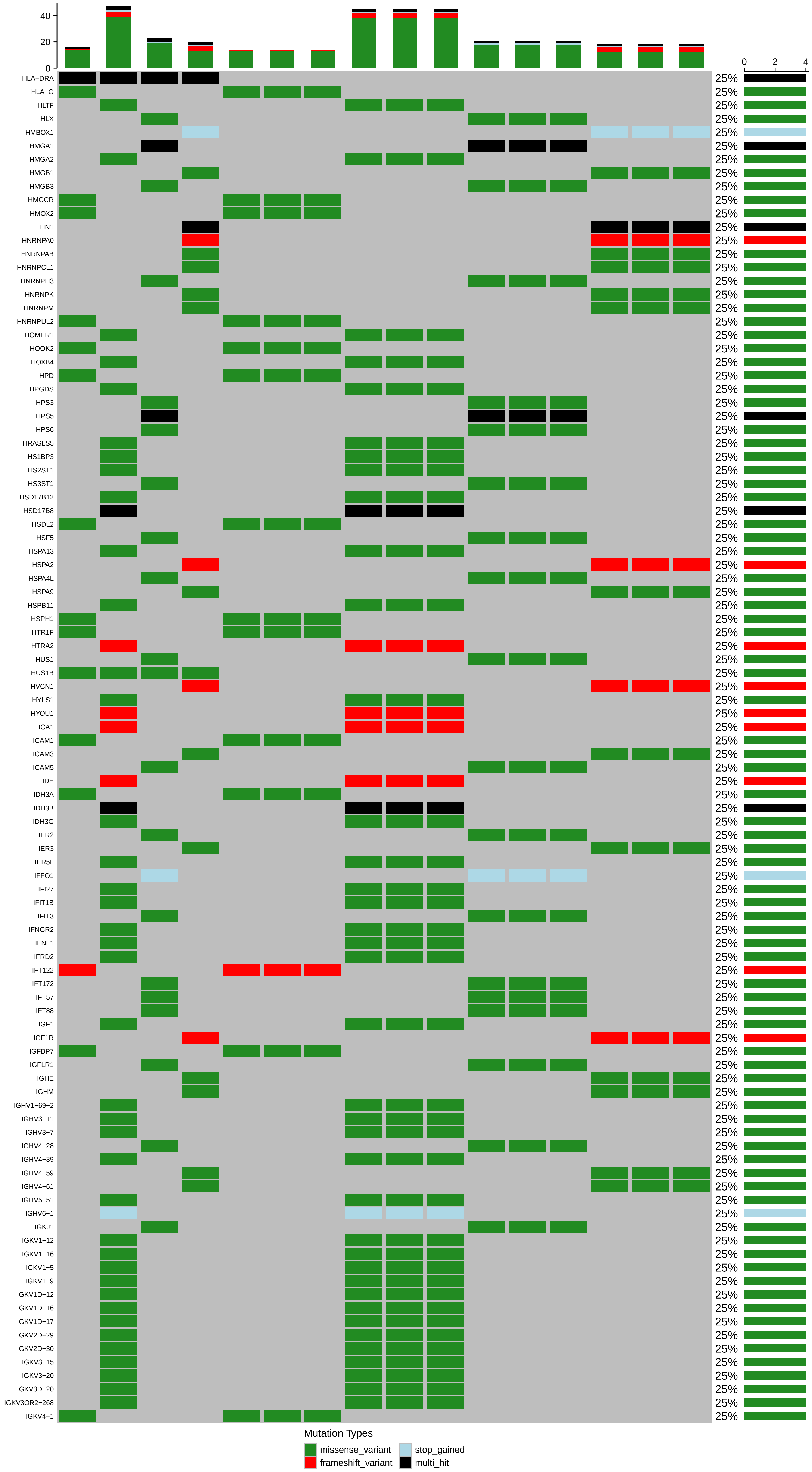

40
20
0
0
2
4
HLA−DRA
HLA−G
HLTF
HLX
HMBOX1
HMGA1
HMGA2
HMGB1
HMGB3
HMGCR
HMOX2
HN1
HNRNPA0
HNRNPAB
HNRNPCL1
HNRNPH3
HNRNPK
HNRNPM
HNRNPUL2
HOMER1
HOOK2
HOXB4
HPD
HPGDS
HPS3
HPS5
HPS6
HRASLS5
HS1BP3
HS2ST1
HS3ST1
HSD17B12
HSD17B8
HSDL2
HSF5
HSPA13
HSPA2
HSPA4L
HSPA9
HSPB11
HSPH1
HTR1F
HTRA2
HUS1
HUS1B
HVCN1
HYLS1
HYOU1
ICA1
ICAM1
ICAM3
ICAM5
IDE
IDH3A
IDH3B
IDH3G
IER2
IER3
IER5L
IFFO1
IFI27
IFIT1B
IFIT3
IFNGR2
IFNL1
IFRD2
IFT122
IFT172
IFT57
IFT88
IGF1
IGF1R
IGFBP7
IGFLR1
IGHE
IGHM
IGHV1−69−2
IGHV3−11
IGHV3−7
IGHV4−28
IGHV4−39
IGHV4−59
IGHV4−61
IGHV5−51
IGHV6−1
IGKJ1
IGKV1−12
IGKV1−16
IGKV1−5
IGKV1−9
IGKV1D−12
IGKV1D−16
IGKV1D−17
IGKV2D−29
IGKV2D−30
IGKV3−15
IGKV3−20
IGKV3D−20
IGKV3OR2−268
IGKV4−1
25% (printed beside each of the 100 gene rows)
Mutation Types
missense_variant
frameshift_variant
stop_gained
multi_hit

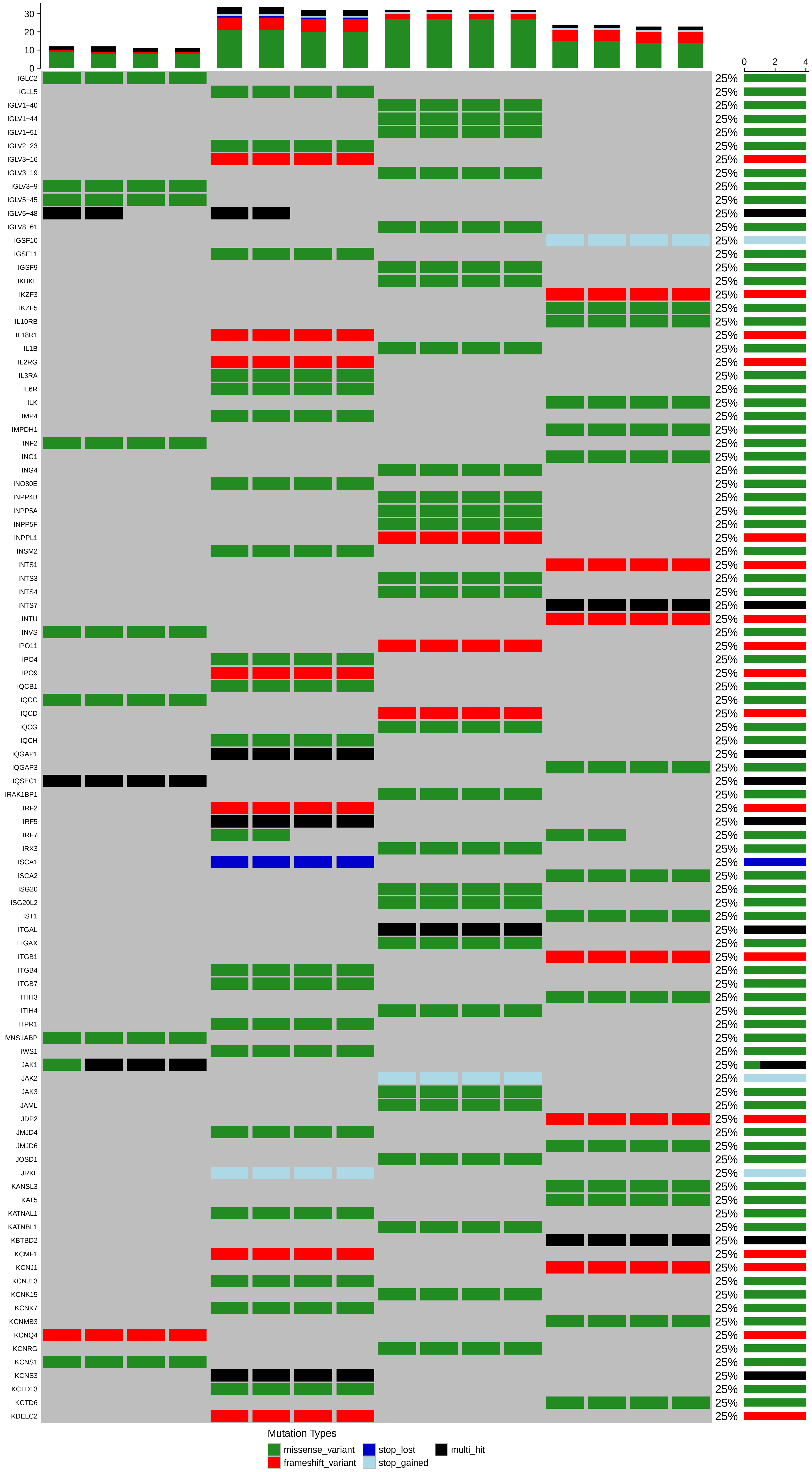

30
20
10
0
0
2
4
IGLC2
IGLL5
IGLV1−40
IGLV1−44
IGLV1−51
IGLV2−23
IGLV3−16
IGLV3−19
IGLV3−9
IGLV5−45
IGLV5−48
IGLV8−61
IGSF10
IGSF11
IGSF9
IKBKE
IKZF3
IKZF5
IL10RB
IL18R1
IL1B
IL2RG
IL3RA
IL6R
ILK
IMP4
IMPDH1
INF2
ING1
ING4
INO80E
INPP4B
INPP5A
INPP5F
INPPL1
INSM2
INTS1
INTS3
INTS4
INTS7
INTU
INVS
IPO11
IPO4
IPO9
IQCB1
IQCC
IQCD
IQCG
IQCH
IQGAP1
IQGAP3
IQSEC1
IRAK1BP1
IRF2
IRF5
IRF7
IRX3
ISCA1
ISCA2
ISG20
ISG20L2
IST1
ITGAL
ITGAX
ITGB1
ITGB4
ITGB7
ITIH3
ITIH4
ITPR1
IVNS1ABP
IWS1
JAK1
JAK2
JAK3
JAML
JDP2
JMJD4
JMJD6
JOSD1
JRKL
KANSL3
KAT5
KATNAL1
KATNBL1
KBTBD2
KCMF1
KCNJ1
KCNJ13
KCNK15
KCNK7
KCNMB3
KCNQ4
KCNRG
KCNS1
KCNS3
KCTD13
KCTD6
KDELC2
25%
Mutation Types
missense_variant
frameshift_variant
stop_lost
stop_gained
multi_hit

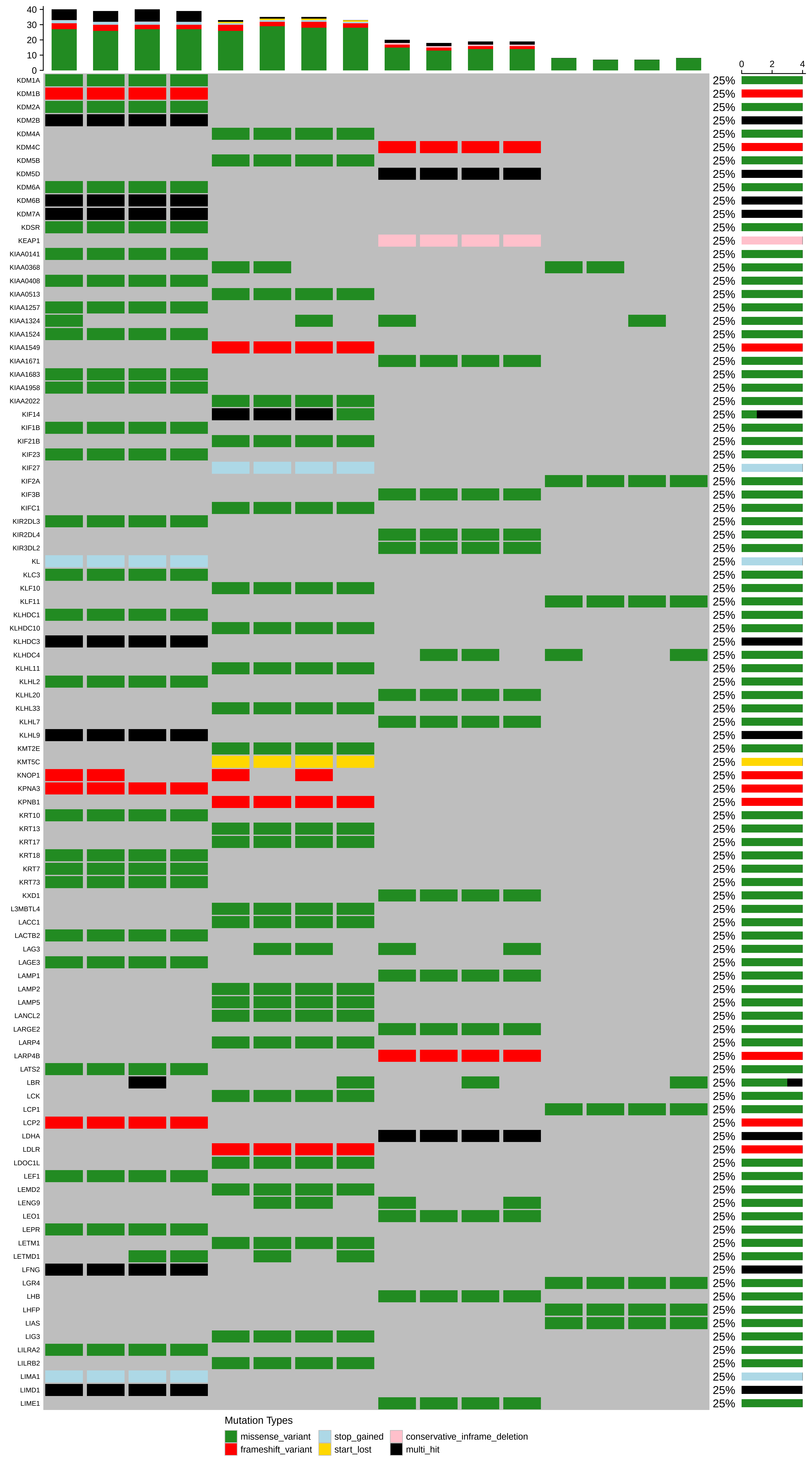
40
30
20
10
0
0
2
4
KDM1A
KDM1B
KDM2A
KDM2B
KDM4A
KDM4C
KDM5B
KDM5D
KDM6A
KDM6B
KDM7A
KDSR
KEAP1
KIAA0141
KIAA0368
KIAA0408
KIAA0513
KIAA1257
KIAA1324
KIAA1524
KIAA1549
KIAA1671
KIAA1683
KIAA1958
KIAA2022
KIF14
KIF1B
KIF21B
KIF23
KIF27
KIF2A
KIF3B
KIFC1
KIR2DL3
KIR2DL4
KIR3DL2
KL
KLC3
KLF10
KLF11
KLHDC1
KLHDC10
KLHDC3
KLHDC4
KLHL11
KLHL2
KLHL20
KLHL33
KLHL7
KLHL9
KMT2E
KMT5C
KNOP1
KPNA3
KPNB1
KRT10
KRT13
KRT17
KRT18
KRT7
KRT73
KXD1
L3MBTL4
LACC1
LACTB2
LAG3
LAGE3
LAMP1
LAMP2
LAMP5
LANCL2
LARGE2
LARP4
LARP4B
LATS2
LBR
LCK
LCP1
LCP2
LDHA
LDLR
LDOC1L
LEF1
LEMD2
LENG9
LEO1
LEPR
LETM1
LETMD1
LFNG
LGR4
LHB
LHFP
LIAS
LIG3
LILRA2
LILRB2
LIMA1
LIMD1
LIME1
25%
Mutation Types
missense_variant
frameshift_variant
stop_gained
start_lost
conservative_inframe_deletion
multi_hit

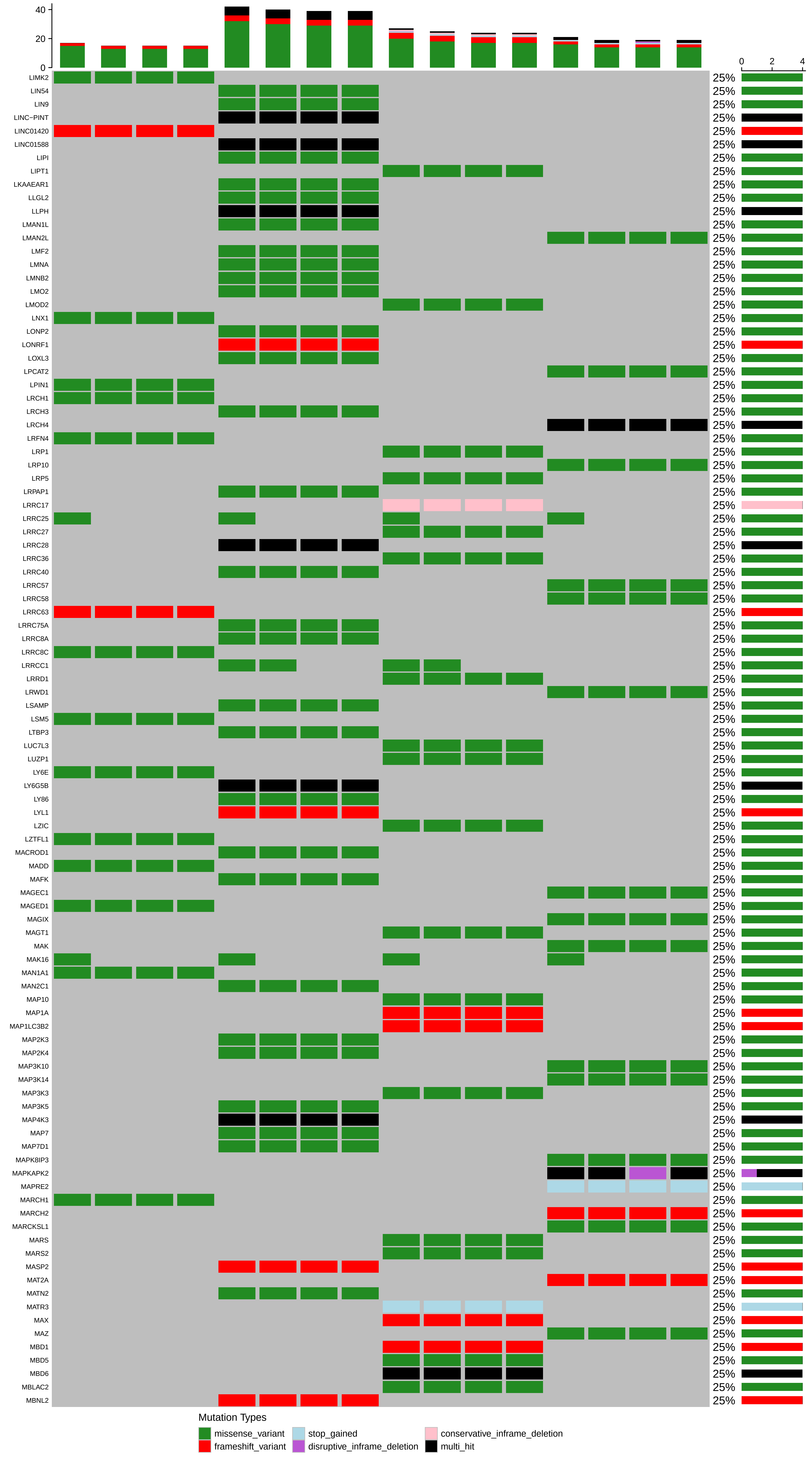
40
20
0
0
2
4
LIMK2
LIN54
LIN9
LINC−PINT
LINC01420
LINC01588
LIPI
LIPT1
LKAAEAR1
LLGL2
LLPH
LMAN1L
LMAN2L
LMF2
LMNA
LMNB2
LMO2
LMOD2
LNX1
LONP2
LONRF1
LOXL3
LPCAT2
LPIN1
LRCH1
LRCH3
LRCH4
LRFN4
LRP1
LRP10
LRP5
LRPAP1
LRRC17
LRRC25
LRRC27
LRRC28
LRRC36
LRRC40
LRRC57
LRRC58
LRRC63
LRRC75A
LRRC8A
LRRC8C
LRRCC1
LRRD1
LRWD1
LSAMP
LSM5
LTBP3
LUC7L3
LUZP1
LY6E
LY6G5B
LY86
LYL1
LZIC
LZTFL1
MACROD1
MADD
MAFK
MAGEC1
MAGED1
MAGIX
MAGT1
MAK
MAK16
MAN1A1
MAN2C1
MAP10
MAP1A
MAP1LC3B2
MAP2K3
MAP2K4
MAP3K10
MAP3K14
MAP3K3
MAP3K5
MAP4K3
MAP7
MAP7D1
MAPK8IP3
MAPKAPK2
MAPRE2
MARCH1
MARCH2
MARCKSL1
MARS
MARS2
MASP2
MAT2A
MATN2
MATR3
MAX
MAZ
MBD1
MBD5
MBD6
MBLAC2
MBNL2
25% (each row)
Mutation Types
missense_variant
frameshift_variant
stop_gained
disruptive_inframe_deletion
conservative_inframe_deletion
multi_hit

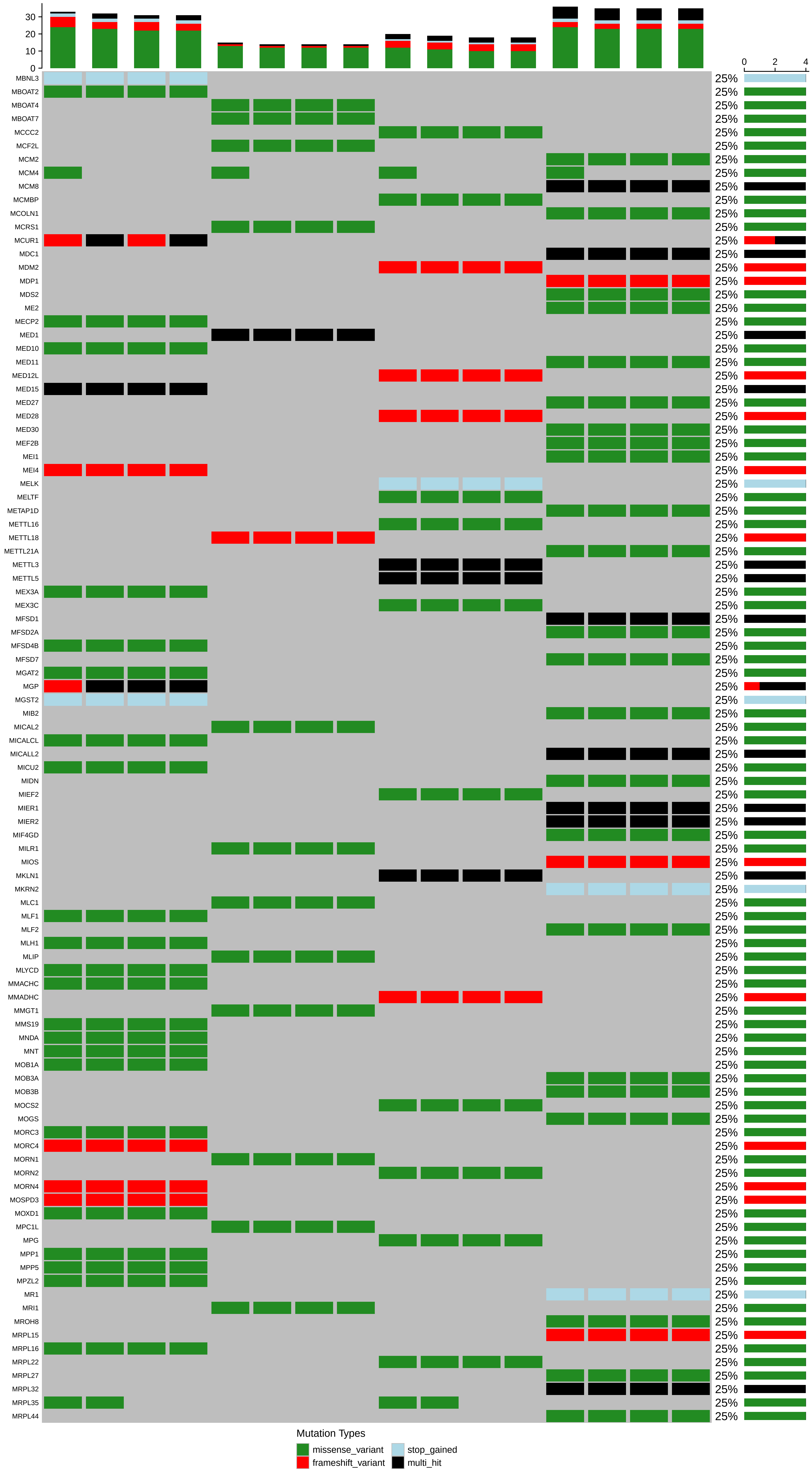

30
20
10
0
0
2
4
MBNL3
MBOAT2
MBOAT4
MBOAT7
MCCC2
MCF2L
MCM2
MCM4
MCM8
MCMBP
MCOLN1
MCRS1
MCUR1
MDC1
MDM2
MDP1
MDS2
ME2
MECP2
MED1
MED10
MED11
MED12L
MED15
MED27
MED28
MED30
MEF2B
MEI1
MEI4
MELK
MELTF
METAP1D
METTL16
METTL18
METTL21A
METTL3
METTL5
MEX3A
MEX3C
MFSD1
MFSD2A
MFSD4B
MFSD7
MGAT2
MGP
MGST2
MIB2
MICAL2
MICALCL
MICALL2
MICU2
MIDN
MIEF2
MIER1
MIER2
MIF4GD
MILR1
MIOS
MKLN1
MKRN2
MLC1
MLF1
MLF2
MLH1
MLIP
MLYCD
MMACHC
MMADHC
MMGT1
MMS19
MNDA
MNT
MOB1A
MOB3A
MOB3B
MOCS2
MOGS
MORC3
MORC4
MORN1
MORN2
MORN4
MOSPD3
MOXD1
MPC1L
MPG
MPP1
MPP5
MPZL2
MR1
MRI1
MROH8
MRPL15
MRPL16
MRPL22
MRPL27
MRPL32
MRPL35
MRPL44
25%
Mutation Types
missense_variant
frameshift_variant
stop_gained
multi_hit

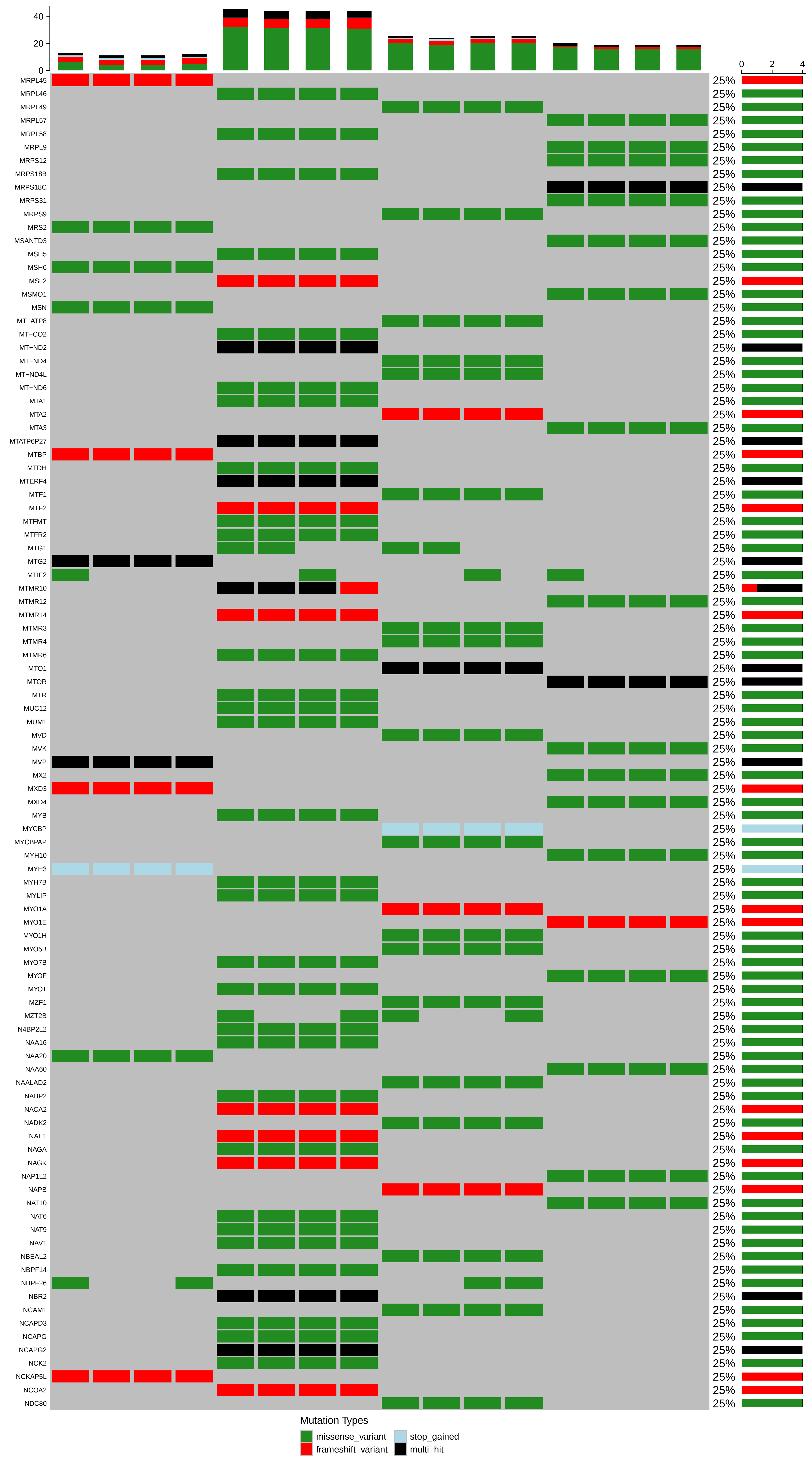
40
20
0
0
2
4
MRPL45 25%
MRPL46 25%
MRPL49 25%
MRPL57 25%
MRPL58 25%
MRPL9 25%
MRPS12 25%
MRPS18B 25%
MRPS18C 25%
MRPS31 25%
MRPS9 25%
MRS2 25%
MSANTD3 25%
MSH5 25%
MSH6 25%
MSL2 25%
MSMO1 25%
MSN 25%
MT−ATP8 25%
MT−CO2 25%
MT−ND2 25%
MT−ND4 25%
MT−ND4L 25%
MT−ND6 25%
MTA1 25%
MTA2 25%
MTA3 25%
MTATP6P27 25%
MTBP 25%
MTDH 25%
MTERF4 25%
MTF1 25%
MTF2 25%
MTFMT 25%
MTFR2 25%
MTG1 25%
MTG2 25%
MTIF2 25%
MTMR10 25%
MTMR12 25%
MTMR14 25%
MTMR3 25%
MTMR4 25%
MTMR6 25%
MTO1 25%
MTOR 25%
MTR 25%
MUC12 25%
MUM1 25%
MVD 25%
MVK 25%
MVP 25%
MX2 25%
MXD3 25%
MXD4 25%
MYB 25%
MYCBP 25%
MYCBPAP 25%
MYH10 25%
MYH3 25%
MYH7B 25%
MYLIP 25%
MYO1A 25%
MYO1E 25%
MYO1H 25%
MYO5B 25%
MYO7B 25%
MYOF 25%
MYOT 25%
MZF1 25%
MZT2B 25%
N4BP2L2 25%
NAA16 25%
NAA20 25%
NAA60 25%
NAALAD2 25%
NABP2 25%
NACA2 25%
NADK2 25%
NAE1 25%
NAGA 25%
NAGK 25%
NAP1L2 25%
NAPB 25%
NAT10 25%
NAT6 25%
NAT9 25%
NAV1 25%
NBEAL2 25%
NBPF14 25%
NBPF26 25%
NBR2 25%
NCAM1 25%
NCAPD3 25%
NCAPG 25%
NCAPG2 25%
NCK2 25%
NCKAP5L 25%
NCOA2 25%
NDC80 25%
Mutation Types
missense_variant
stop_gained
frameshift_variant
multi_hit

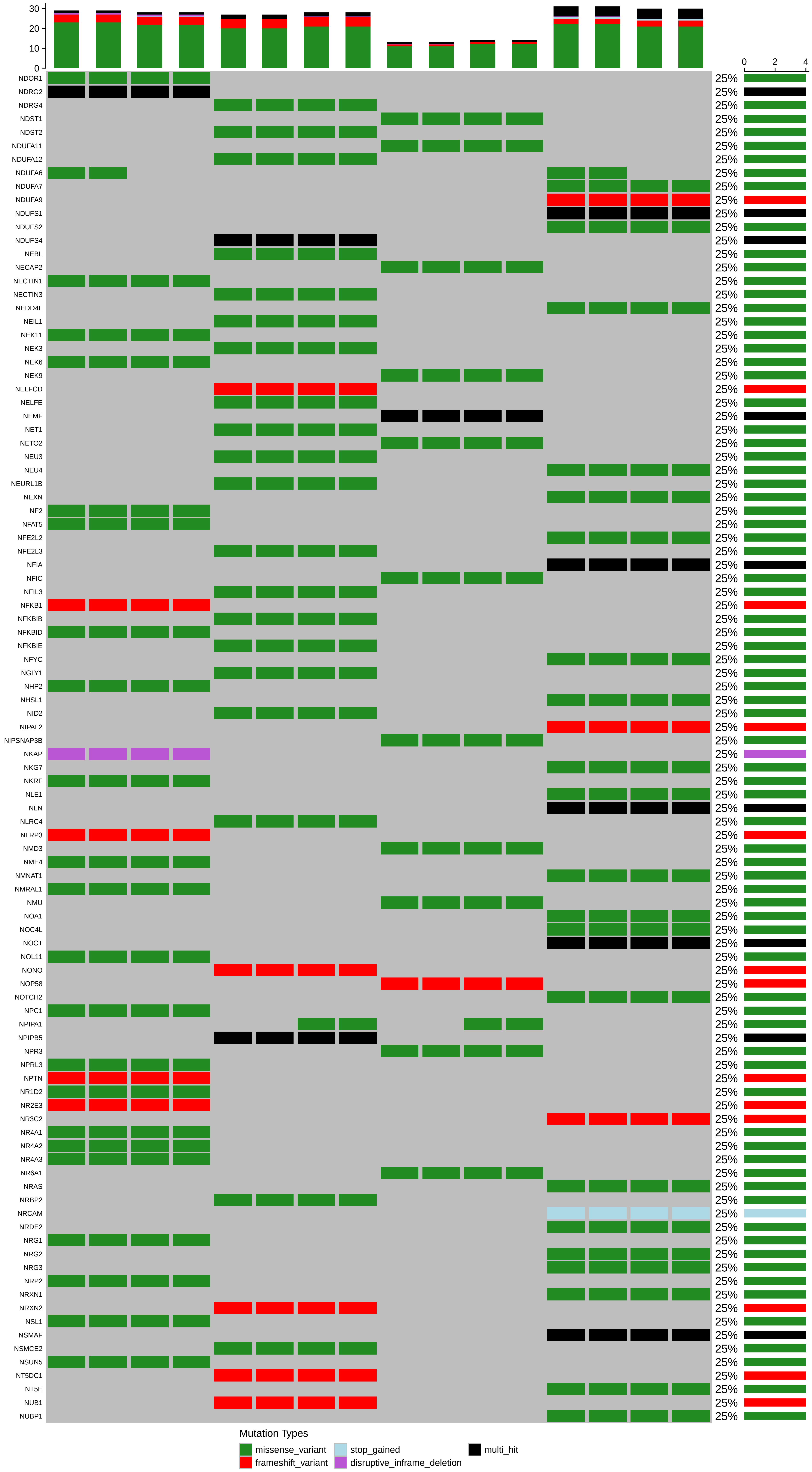

30
20
10
0
0
2
4
NDOR1
NDRG2
NDRG4
NDST1
NDST2
NDUFA11
NDUFA12
NDUFA6
NDUFA7
NDUFA9
NDUFS1
NDUFS2
NDUFS4
NEBL
NECAP2
NECTIN1
NECTIN3
NEDD4L
NEIL1
NEK11
NEK3
NEK6
NEK9
NELFCD
NELFE
NEMF
NET1
NETO2
NEU3
NEU4
NEURL1B
NEXN
NF2
NFAT5
NFE2L2
NFE2L3
NFIA
NFIC
NFIL3
NFKB1
NFKBIB
NFKBID
NFKBIE
NFYC
NGLY1
NHP2
NHSL1
NID2
NIPAL2
NIPSNAP3B
NKAP
NKG7
NKRF
NLE1
NLN
NLRC4
NLRP3
NMD3
NME4
NMNAT1
NMRAL1
NMU
NOA1
NOC4L
NOCT
NOL11
NONO
NOP58
NOTCH2
NPC1
NPIPA1
NPIPB5
NPR3
NPRL3
NPTN
NR1D2
NR2E3
NR3C2
NR4A1
NR4A2
NR4A3
NR6A1
NRAS
NRBP2
NRCAM
NRDE2
NRG1
NRG2
NRG3
NRP2
NRXN1
NRXN2
NSL1
NSMAF
NSMCE2
NSUN5
NT5DC1
NT5E
NUB1
NUBP1
25% (printed beside each of the 100 gene rows)
Mutation Types
missense_variant
frameshift_variant
stop_gained
disruptive_inframe_deletion
multi_hit

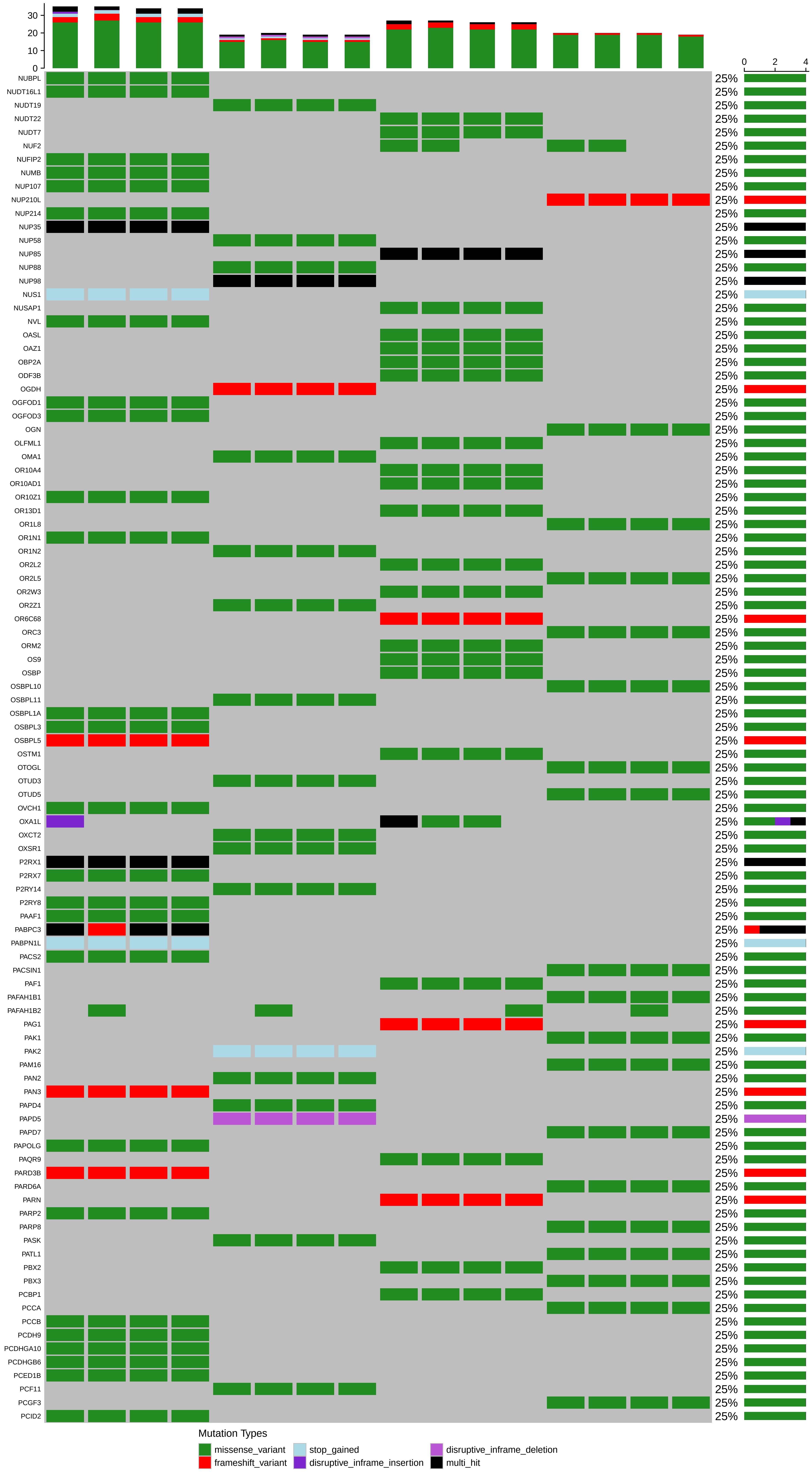

30
20
10
0
0
2
4
NUBPL
NUDT16L1
NUDT19
NUDT22
NUDT7
NUF2
NUFIP2
NUMB
NUP107
NUP210L
NUP214
NUP35
NUP58
NUP85
NUP88
NUP98
NUS1
NUSAP1
NVL
OASL
OAZ1
OBP2A
ODF3B
OGDH
OGFOD1
OGFOD3
OGN
OLFML1
OMA1
OR10A4
OR10AD1
OR10Z1
OR13D1
OR1L8
OR1N1
OR1N2
OR2L2
OR2L5
OR2W3
OR2Z1
OR6C68
ORC3
ORM2
OS9
OSBP
OSBPL10
OSBPL11
OSBPL1A
OSBPL3
OSBPL5
OSTM1
OTOGL
OTUD3
OTUD5
OVCH1
OXA1L
OXCT2
OXSR1
P2RX1
P2RX7
P2RY14
P2RY8
PAAF1
PABPC3
PABPN1L
PACS2
PACSIN1
PAF1
PAFAH1B1
PAFAH1B2
PAG1
PAK1
PAK2
PAM16
PAN2
PAN3
PAPD4
PAPD5
PAPD7
PAPOLG
PAQR9
PARD3B
PARD6A
PARN
PARP2
PARP8
PASK
PATL1
PBX2
PBX3
PCBP1
PCCA
PCCB
PCDH9
PCDHGA10
PCDHGB6
PCED1B
PCF11
PCGF3
PCID2
25% (each gene row)
Mutation Types
missense_variant
frameshift_variant
stop_gained
disruptive_inframe_insertion
disruptive_inframe_deletion
multi_hit

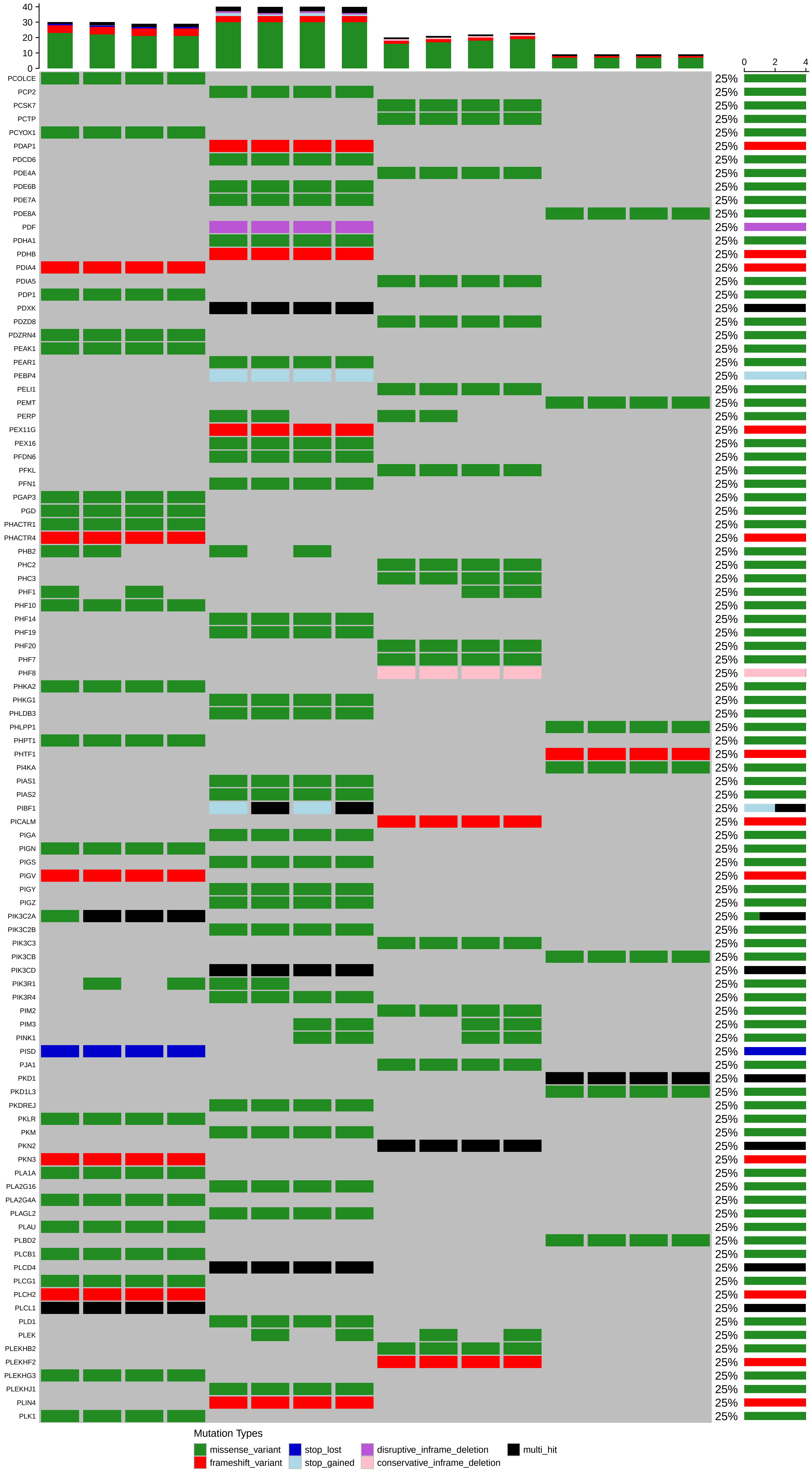
PCOLCE
PCP2
PCSK7
PCTP
PCYOX1
PDAP1
PDCD6
PDE4A
PDE6B
PDE7A
PDE8A
PDF
PDHA1
PDHB
PDIA4
PDIA5
PDP1
PDXK
PDZD8
PDZRN4
PEAK1
PEAR1
PEBP4
PELI1
PEMT
PERP
PEX11G
PEX16
PFDN6
PFKL
PFN1
PGAP3
PGD
PHACTR1
PHACTR4
PHB2
PHC2
PHC3
PHF1
PHF10
PHF14
PHF19
PHF20
PHF7
PHF8
PHKA2
PHKG1
PHLDB3
PHLPP1
PHPT1
PHTF1
PI4KA
PIAS1
PIAS2
PIBF1
PICALM
PIGA
PIGN
PIGS
PIGV
PIGY
PIGZ
PIK3C2A
PIK3C2B
PIK3C3
PIK3CB
PIK3CD
PIK3R1
PIK3R4
PIM2
PIM3
PINK1
PISD
PJA1
PKD1
PKD1L3
PKDREJ
PKLR
PKM
PKN2
PKN3
PLA1A
PLA2G16
PLA2G4A
PLAGL2
PLAU
PLBD2
PLCB1
PLCD4
PLCG1
PLCH2
PLCL1
PLD1
PLEK
PLEKHB2
PLEKHF2
PLEKHG3
PLEKHJ1
PLIN4
PLK1
25%
0 2 4
0 10 20 30 40
Mutation Types
missense_variant
frameshift_variant
stop_lost
stop_gained
disruptive_inframe_deletion
conservative_inframe_deletion
multi_hit

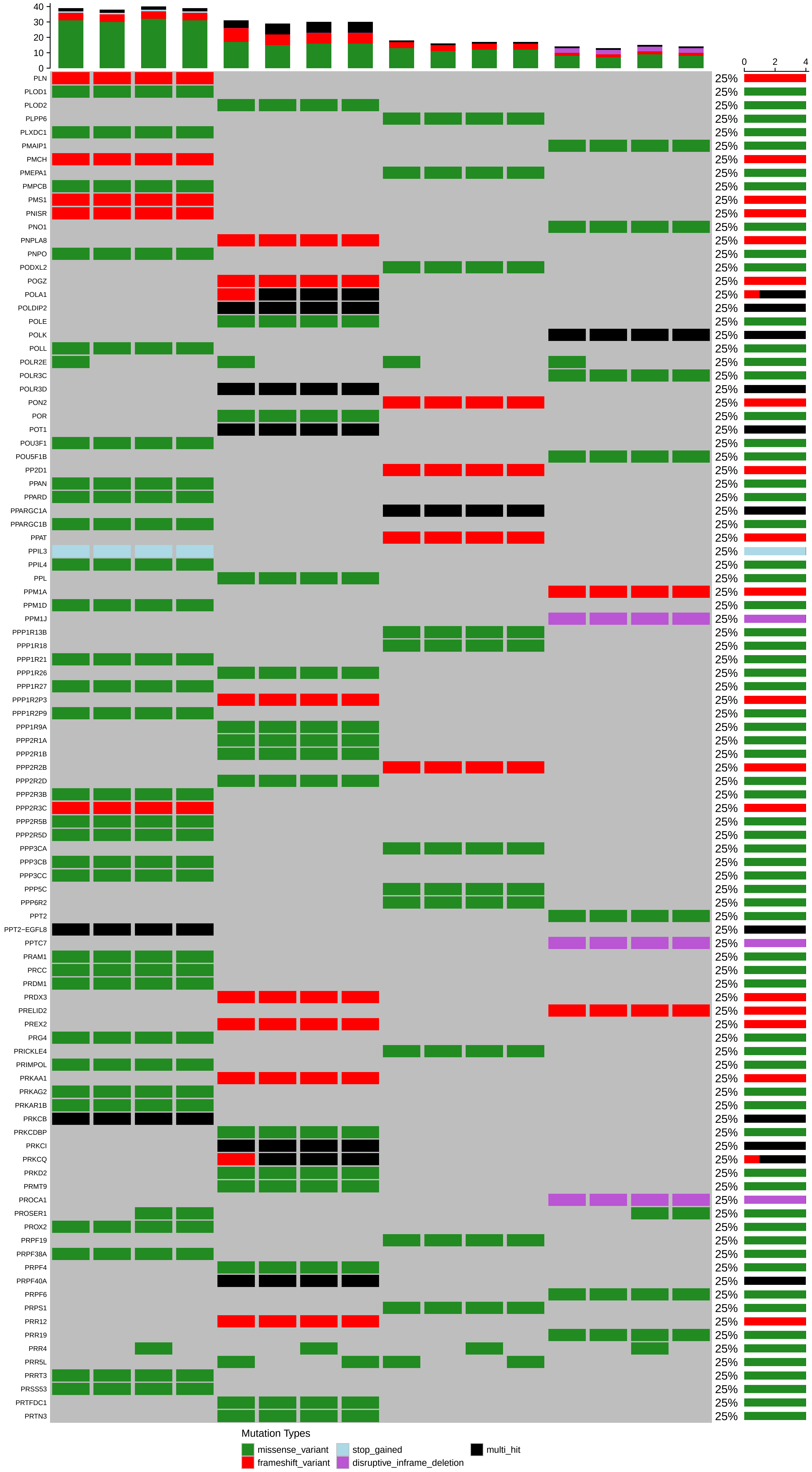

40
30
20
10
0
0
2
4
PLN
PLOD1
PLOD2
PLPP6
PLXDC1
PMAIP1
PMCH
PMEPA1
PMPCB
PMS1
PNISR
PNO1
PNPLA8
PNPO
PODXL2
POGZ
POLA1
POLDIP2
POLE
POLK
POLL
POLR2E
POLR3C
POLR3D
PON2
POR
POT1
POU3F1
POU5F1B
PP2D1
PPAN
PPARD
PPARGC1A
PPARGC1B
PPAT
PPIL3
PPIL4
PPL
PPM1A
PPM1D
PPM1J
PPP1R13B
PPP1R18
PPP1R21
PPP1R26
PPP1R27
PPP1R2P3
PPP1R2P9
PPP1R9A
PPP2R1A
PPP2R1B
PPP2R2B
PPP2R2D
PPP2R3B
PPP2R3C
PPP2R5B
PPP2R5D
PPP3CA
PPP3CB
PPP3CC
PPP5C
PPP6R2
PPT2
PPT2−EGFL8
PPTC7
PRAM1
PRCC
PRDM1
PRDX3
PRELID2
PREX2
PRG4
PRICKLE4
PRIMPOL
PRKAA1
PRKAG2
PRKAR1B
PRKCB
PRKCDBP
PRKCI
PRKCQ
PRKD2
PRMT9
PROCA1
PROSER1
PROX2
PRPF19
PRPF38A
PRPF4
PRPF40A
PRPF6
PRPS1
PRR12
PRR19
PRR4
PRR5L
PRRT3
PRSS53
PRTFDC1
PRTN3
25%
Mutation Types
missense_variant
frameshift_variant
stop_gained
disruptive_inframe_deletion
multi_hit

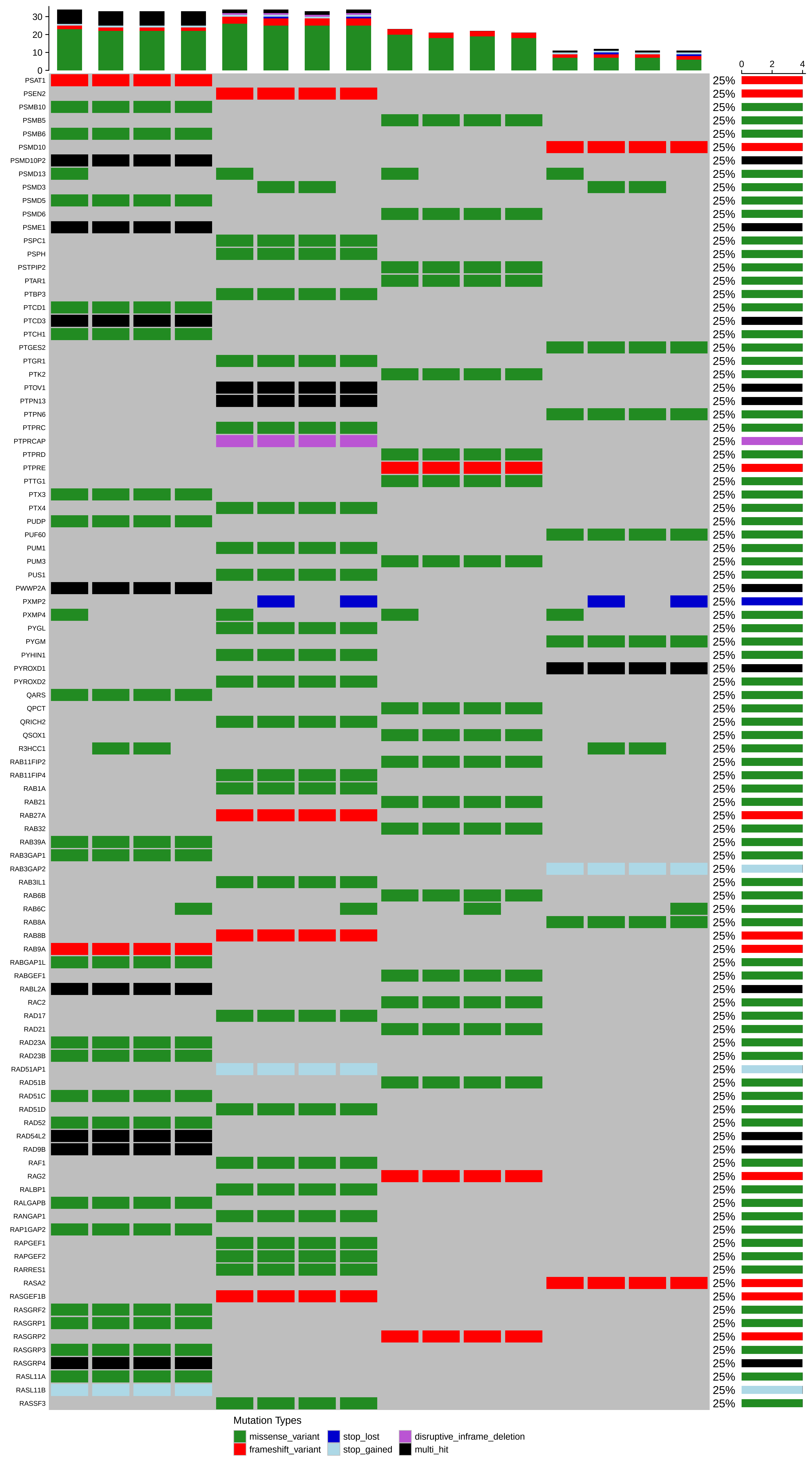

30
20
10
0
0
2
4
PSAT1
PSEN2
PSMB10
PSMB5
PSMB6
PSMD10
PSMD10P2
PSMD13
PSMD3
PSMD5
PSMD6
PSME1
PSPC1
PSPH
PSTPIP2
PTAR1
PTBP3
PTCD1
PTCD3
PTCH1
PTGES2
PTGR1
PTK2
PTOV1
PTPN13
PTPN6
PTPRC
PTPRCAP
PTPRD
PTPRE
PTTG1
PTX3
PTX4
PUDP
PUF60
PUM1
PUM3
PUS1
PWWP2A
PXMP2
PXMP4
PYGL
PYGM
PYHIN1
PYROXD1
PYROXD2
QARS
QPCT
QRICH2
QSOX1
R3HCC1
RAB11FIP2
RAB11FIP4
RAB1A
RAB21
RAB27A
RAB32
RAB39A
RAB3GAP1
RAB3GAP2
RAB3IL1
RAB6B
RAB6C
RAB8A
RAB8B
RAB9A
RABGAP1L
RABGEF1
RABL2A
RAC2
RAD17
RAD21
RAD23A
RAD23B
RAD51AP1
RAD51B
RAD51C
RAD51D
RAD52
RAD54L2
RAD9B
RAF1
RAG2
RALBP1
RALGAPB
RANGAP1
RAP1GAP2
RAPGEF1
RAPGEF2
RARRES1
RASA2
RASGEF1B
RASGRF2
RASGRP1
RASGRP2
RASGRP3
RASGRP4
RASL11A
RASL11B
RASSF3
25%
Mutation Types
missense_variant
frameshift_variant
stop_lost
stop_gained
disruptive_inframe_deletion
multi_hit

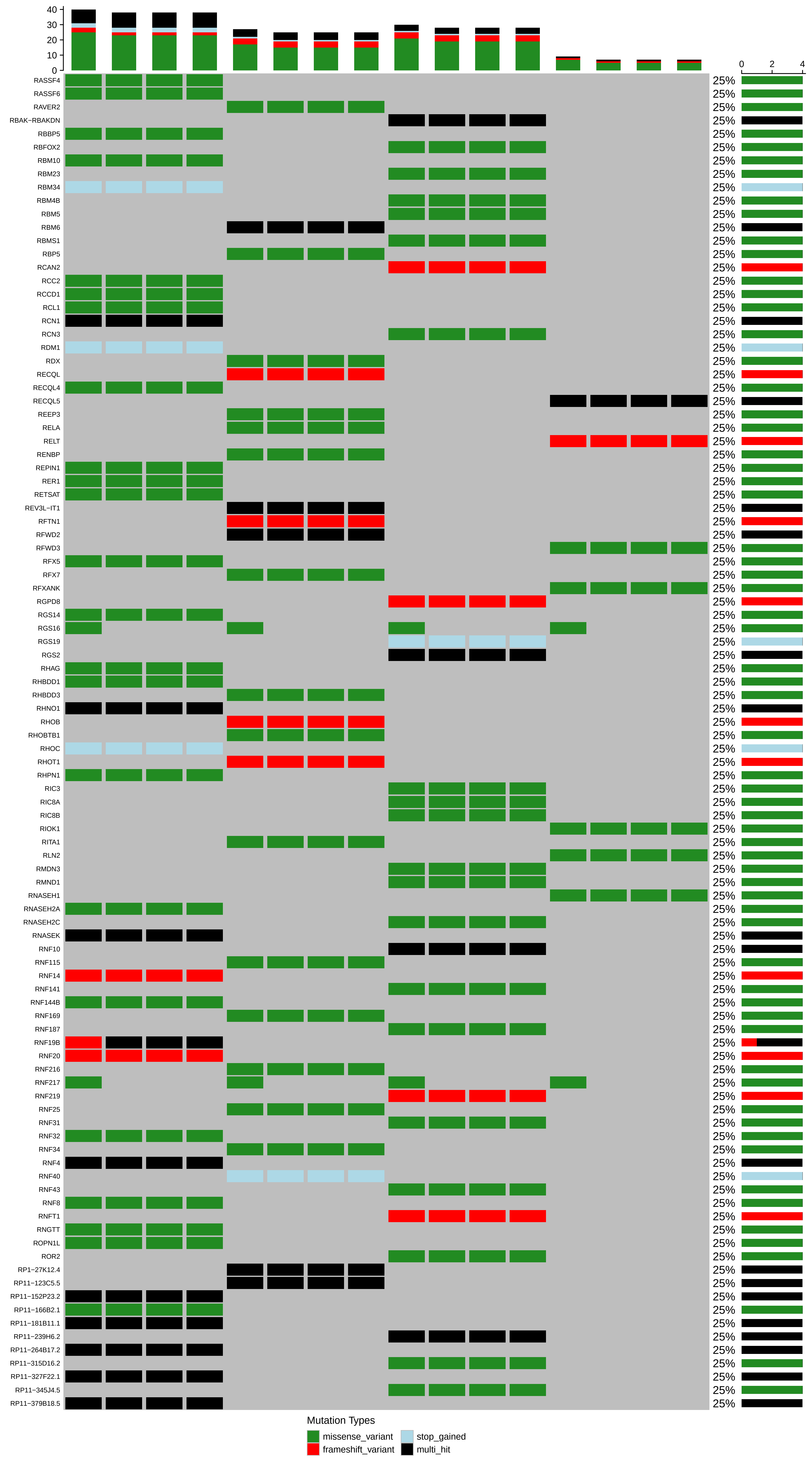

RASSF4
RASSF6
RAVER2
RBAK−RBAKDN
RBBP5
RBFOX2
RBM10
RBM23
RBM34
RBM4B
RBM5
RBM6
RBMS1
RBP5
RCAN2
RCC2
RCCD1
RCL1
RCN1
RCN3
RDM1
RDX
RECQL
RECQL4
RECQL5
REEP3
RELA
RELT
RENBP
REPIN1
RER1
RETSAT
REV3L−IT1
RFTN1
RFWD2
RFWD3
RFX5
RFX7
RFXANK
RGPD8
RGS14
RGS16
RGS19
RGS2
RHAG
RHBDD1
RHBDD3
RHNO1
RHOB
RHOBTB1
RHOC
RHOT1
RHPN1
RIC3
RIC8A
RIC8B
RIOK1
RITA1
RLN2
RMDN3
RMND1
RNASEH1
RNASEH2A
RNASEH2C
RNASEK
RNF10
RNF115
RNF14
RNF141
RNF144B
RNF169
RNF187
RNF19B
RNF20
RNF216
RNF217
RNF219
RNF25
RNF31
RNF32
RNF34
RNF4
RNF40
RNF43
RNF8
RNFT1
RNGTT
ROPN1L
ROR2
RP1−27K12.4
RP11−123C5.5
RP11−152P23.2
RP11−166B2.1
RP11−181B11.1
RP11−239H6.2
RP11−264B17.2
RP11−315D16.2
RP11−327F22.1
RP11−345J4.5
RP11−379B18.5
25%
Mutation Types
missense_variant
frameshift_variant
stop_gained
multi_hit

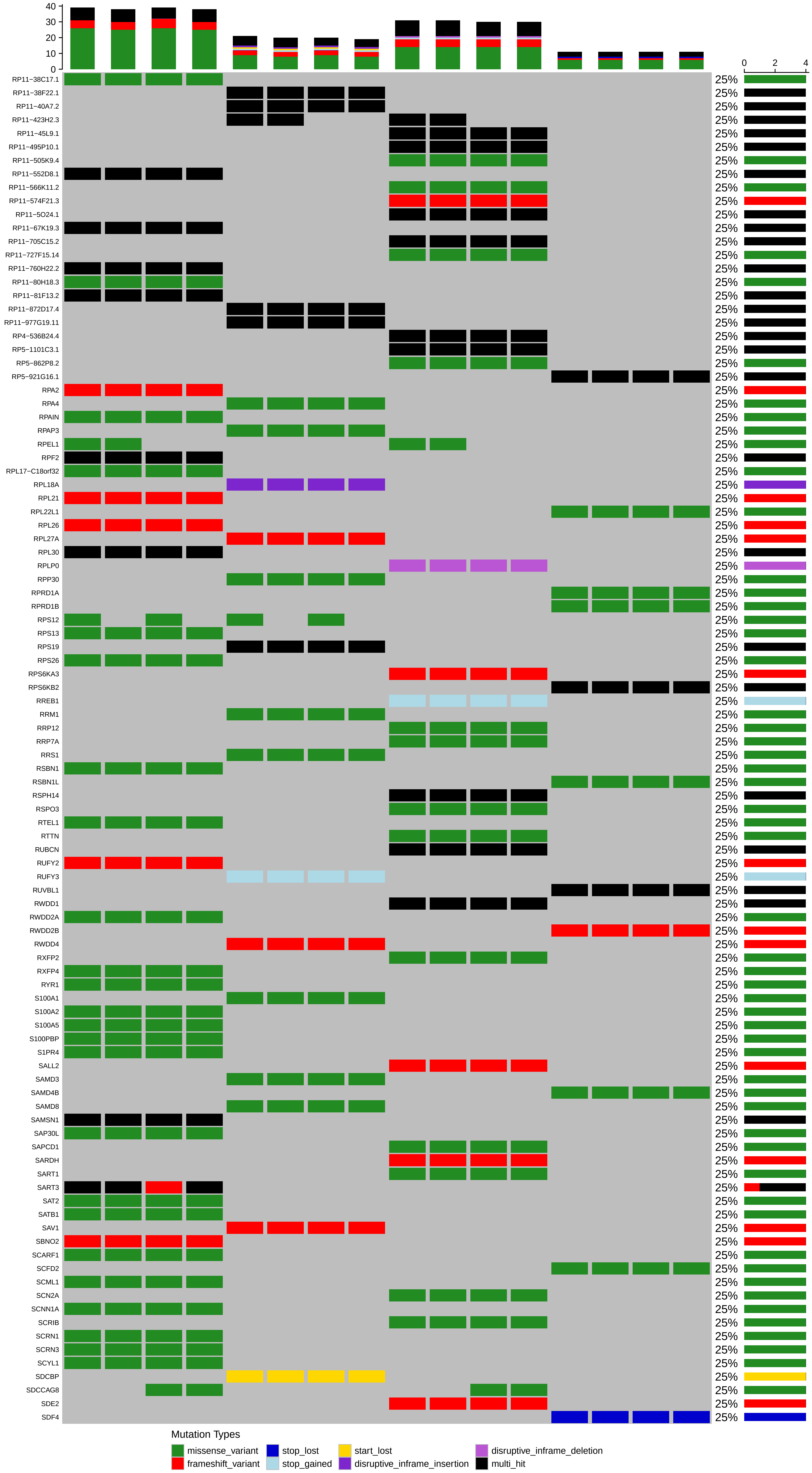

RP11−38C17.1
RP11−38F22.1
RP11−40A7.2
RP11−423H2.3
RP11−45L9.1
RP11−495P10.1
RP11−505K9.4
RP11−552D8.1
RP11−566K11.2
RP11−574F21.3
RP11−5O24.1
RP11−67K19.3
RP11−705C15.2
RP11−727F15.14
RP11−760H22.2
RP11−80H18.3
RP11−81F13.2
RP11−872D17.4
RP11−977G19.11
RP4−536B24.4
RP5−1101C3.1
RP5−862P8.2
RP5−921G16.1
RPA2
RPA4
RPAIN
RPAP3
RPEL1
RPF2
RPL17−C18orf32
RPL18A
RPL21
RPL22L1
RPL26
RPL27A
RPL30
RPLP0
RPP30
RPRD1A
RPRD1B
RPS12
RPS13
RPS19
RPS26
RPS6KA3
RPS6KB2
RREB1
RRM1
RRP12
RRP7A
RRS1
RSBN1
RSBN1L
RSPH14
RSPO3
RTEL1
RTTN
RUBCN
RUFY2
RUFY3
RUVBL1
RWDD1
RWDD2A
RWDD2B
RWDD4
RXFP2
RXFP4
RYR1
S100A1
S100A2
S100A5
S100PBP
S1PR4
SALL2
SAMD3
SAMD4B
SAMD8
SAMSN1
SAP30L
SAPCD1
SARDH
SART1
SART3
SAT2
SATB1
SAV1
SBNO2
SCARF1
SCFD2
SCML1
SCN2A
SCNN1A
SCRIB
SCRN1
SCRN3
SCYL1
SDCBP
SDCCAG8
SDE2
SDF4
Mutation Types
missense_variant
frameshift_variant
stop_lost
stop_gained
start_lost
disruptive_inframe_insertion
disruptive_inframe_deletion
multi_hit

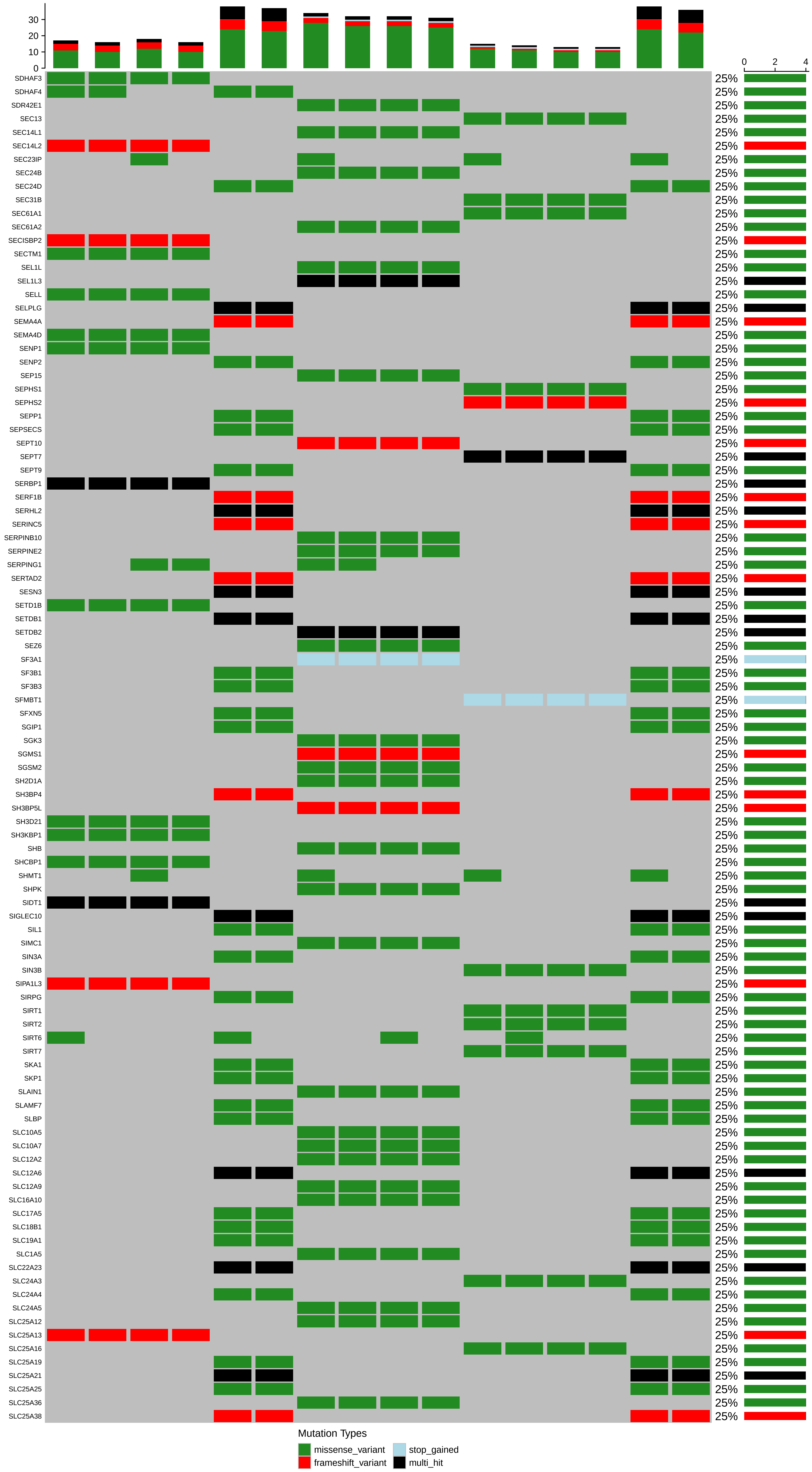

30
20
10
0
0
2
4
SDHAF3
SDHAF4
SDR42E1
SEC13
SEC14L1
SEC14L2
SEC23IP
SEC24B
SEC24D
SEC31B
SEC61A1
SEC61A2
SECISBP2
SECTM1
SEL1L
SEL1L3
SELL
SELPLG
SEMA4A
SEMA4D
SENP1
SENP2
SEP15
SEPHS1
SEPHS2
SEPP1
SEPSECS
SEPT10
SEPT7
SEPT9
SERBP1
SERF1B
SERHL2
SERINC5
SERPINB10
SERPINE2
SERPING1
SERTAD2
SESN3
SETD1B
SETDB1
SETDB2
SEZ6
SF3A1
SF3B1
SF3B3
SFMBT1
SFXN5
SGIP1
SGK3
SGMS1
SGSM2
SH2D1A
SH3BP4
SH3BP5L
SH3D21
SH3KBP1
SHB
SHCBP1
SHMT1
SHPK
SIDT1
SIGLEC10
SIL1
SIMC1
SIN3A
SIN3B
SIPA1L3
SIRPG
SIRT1
SIRT2
SIRT6
SIRT7
SKA1
SKP1
SLAIN1
SLAMF7
SLBP
SLC10A5
SLC10A7
SLC12A2
SLC12A6
SLC12A9
SLC16A10
SLC17A5
SLC18B1
SLC19A1
SLC1A5
SLC22A23
SLC24A3
SLC24A4
SLC24A5
SLC25A12
SLC25A13
SLC25A16
SLC25A19
SLC25A21
SLC25A25
SLC25A36
SLC25A38
25%
Mutation Types
missense_variant
frameshift_variant
stop_gained
multi_hit

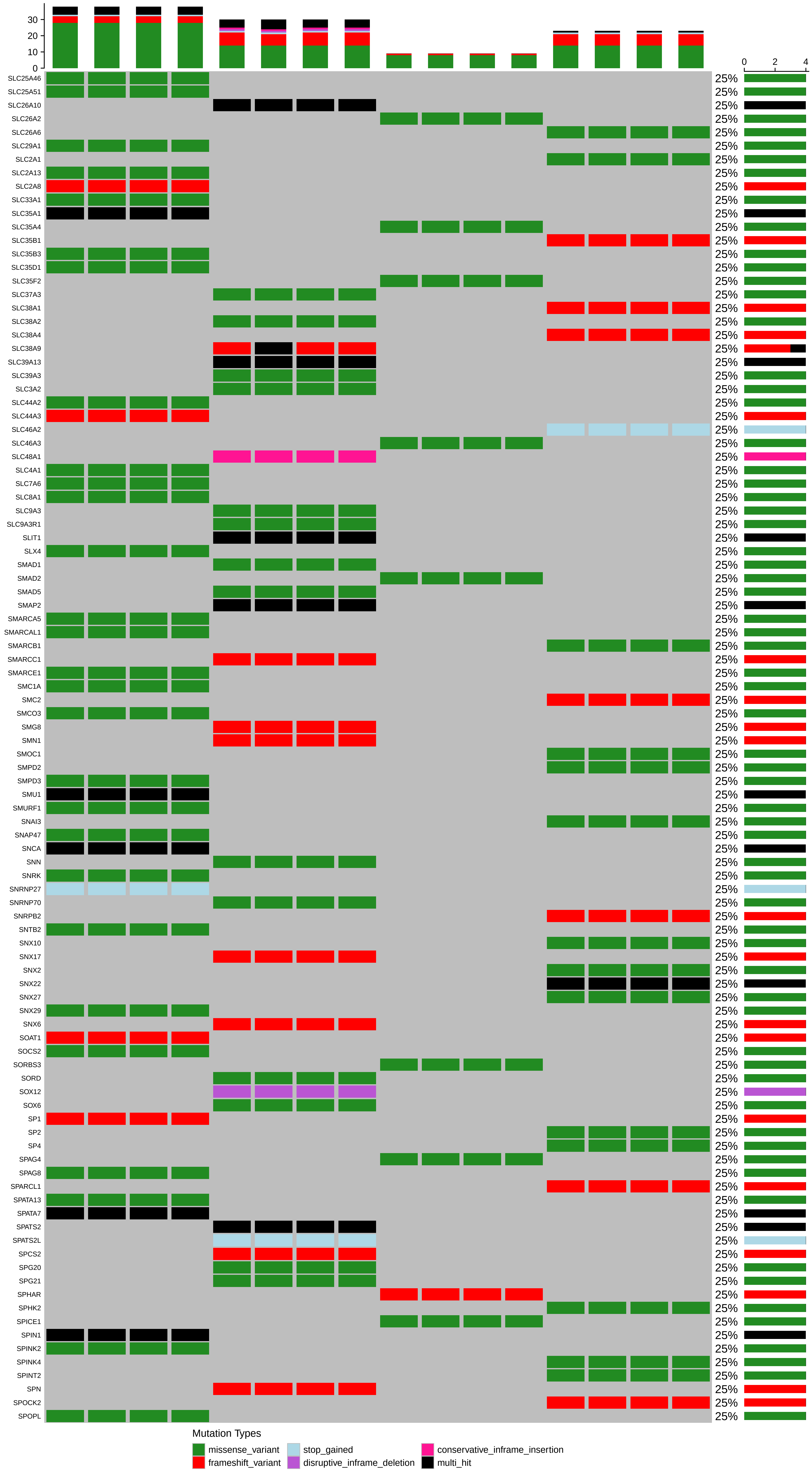

30
20
10
0
0
2
4
SLC25A46
SLC25A51
SLC26A10
SLC26A2
SLC26A6
SLC29A1
SLC2A1
SLC2A13
SLC2A8
SLC33A1
SLC35A1
SLC35A4
SLC35B1
SLC35B3
SLC35D1
SLC35F2
SLC37A3
SLC38A1
SLC38A2
SLC38A4
SLC38A9
SLC39A13
SLC39A3
SLC3A2
SLC44A2
SLC44A3
SLC46A2
SLC46A3
SLC48A1
SLC4A1
SLC7A6
SLC8A1
SLC9A3
SLC9A3R1
SLIT1
SLX4
SMAD1
SMAD2
SMAD5
SMAP2
SMARCA5
SMARCAL1
SMARCB1
SMARCC1
SMARCE1
SMC1A
SMC2
SMCO3
SMG8
SMN1
SMOC1
SMPD2
SMPD3
SMU1
SMURF1
SNAI3
SNAP47
SNCA
SNN
SNRK
SNRNP27
SNRNP70
SNRPB2
SNTB2
SNX10
SNX17
SNX2
SNX22
SNX27
SNX29
SNX6
SOAT1
SOCS2
SORBS3
SORD
SOX12
SOX6
SP1
SP2
SP4
SPAG4
SPAG8
SPARCL1
SPATA13
SPATA7
SPATS2
SPATS2L
SPCS2
SPG20
SPG21
SPHAR
SPHK2
SPICE1
SPIN1
SPINK2
SPINK4
SPINT2
SPN
SPOCK2
SPOPL
25%
Mutation Types
missense_variant
frameshift_variant
stop_gained
disruptive_inframe_deletion
conservative_inframe_insertion
multi_hit

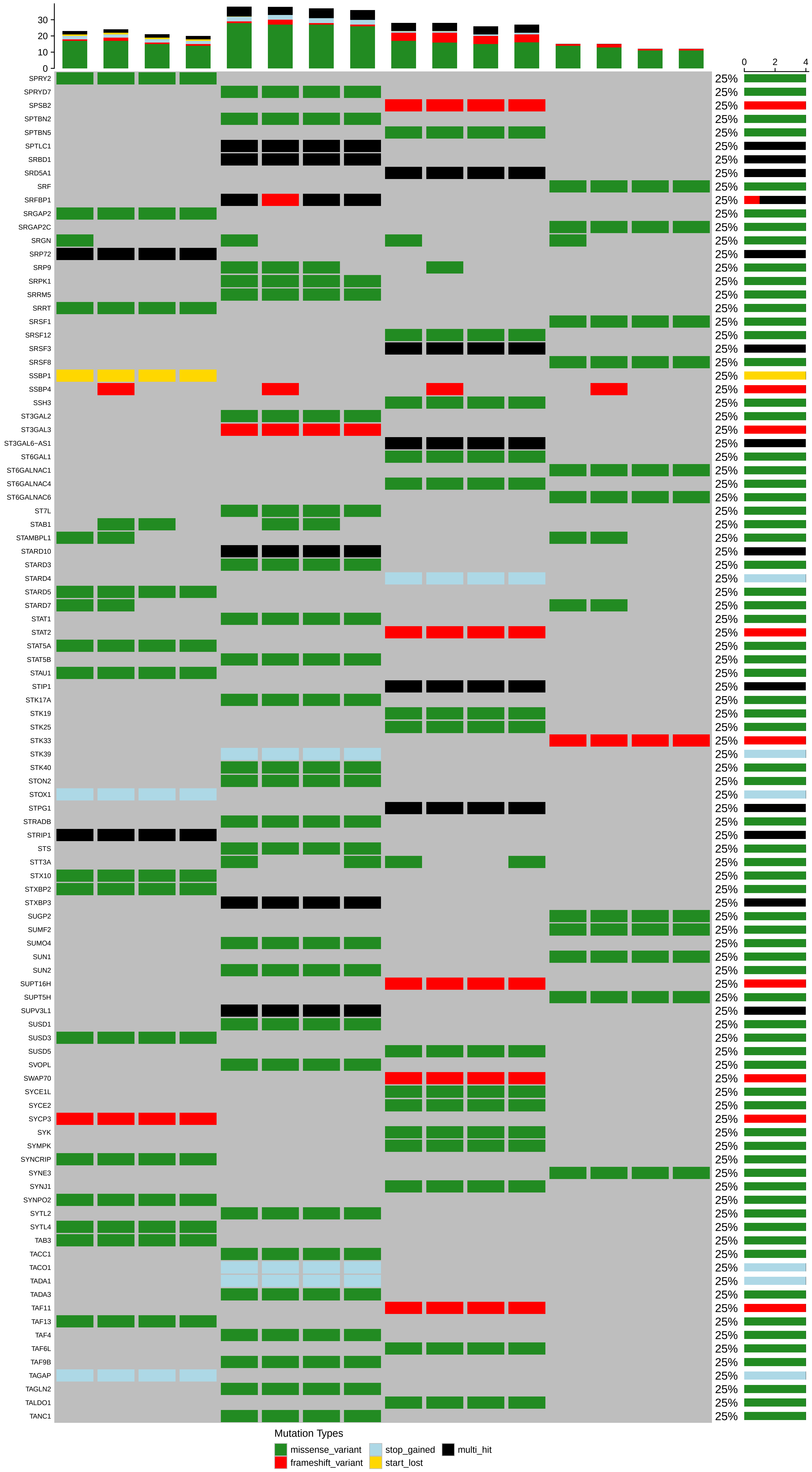

SPRY2
SPRYD7
SPSB2
SPTBN2
SPTBN5
SPTLC1
SRBD1
SRD5A1
SRF
SRFBP1
SRGAP2
SRGAP2C
SRGN
SRP72
SRP9
SRPK1
SRRM5
SRRT
SRSF1
SRSF12
SRSF3
SRSF8
SSBP1
SSBP4
SSH3
ST3GAL2
ST3GAL3
ST3GAL6−AS1
ST6GAL1
ST6GALNAC1
ST6GALNAC4
ST6GALNAC6
ST7L
STAB1
STAMBPL1
STARD10
STARD3
STARD4
STARD5
STARD7
STAT1
STAT2
STAT5A
STAT5B
STAU1
STIP1
STK17A
STK19
STK25
STK33
STK39
STK40
STON2
STOX1
STPG1
STRADB
STRIP1
STS
STT3A
STX10
STXBP2
STXBP3
SUGP2
SUMF2
SUMO4
SUN1
SUN2
SUPT16H
SUPT5H
SUPV3L1
SUSD1
SUSD3
SUSD5
SVOPL
SWAP70
SYCE1L
SYCE2
SYCP3
SYK
SYMPK
SYNCRIP
SYNE3
SYNJ1
SYNPO2
SYTL2
SYTL4
TAB3
TACC1
TACO1
TADA1
TADA3
TAF11
TAF13
TAF4
TAF6L
TAF9B
TAGAP
TAGLN2
TALDO1
TANC1
Mutation Types
missense_variant
frameshift_variant
stop_gained
start_lost
multi_hit

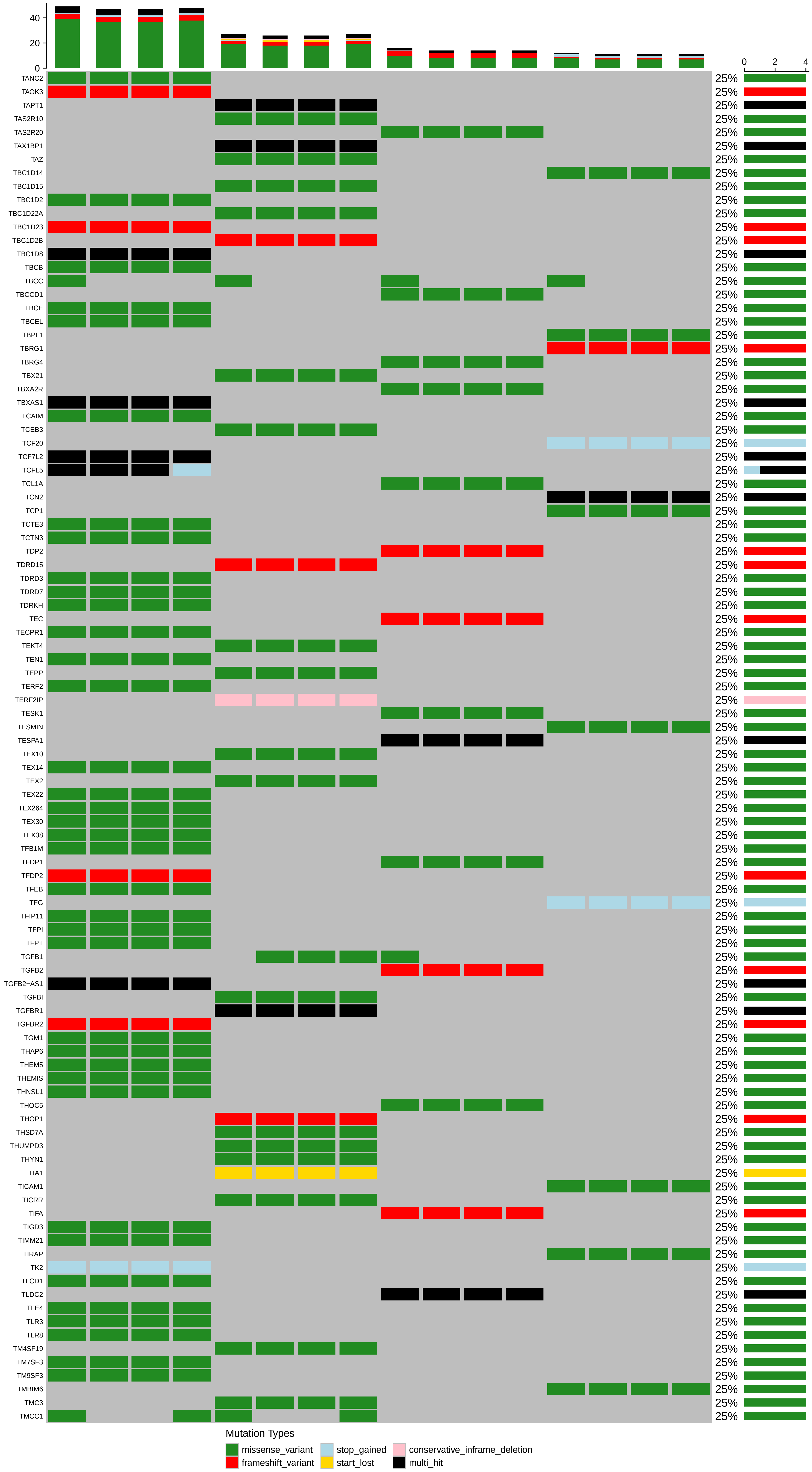

40
20
0
0
2
4
TANC2
TAOK3
TAPT1
TAS2R10
TAS2R20
TAX1BP1
TAZ
TBC1D14
TBC1D15
TBC1D2
TBC1D22A
TBC1D23
TBC1D2B
TBC1D8
TBCB
TBCC
TBCCD1
TBCE
TBCEL
TBPL1
TBRG1
TBRG4
TBX21
TBXA2R
TBXAS1
TCAIM
TCEB3
TCF20
TCF7L2
TCFL5
TCL1A
TCN2
TCP1
TCTE3
TCTN3
TDP2
TDRD15
TDRD3
TDRD7
TDRKH
TEC
TECPR1
TEKT4
TEN1
TEPP
TERF2
TERF2IP
TESK1
TESMIN
TESPA1
TEX10
TEX14
TEX2
TEX22
TEX264
TEX30
TEX38
TFB1M
TFDP1
TFDP2
TFEB
TFG
TFIP11
TFPI
TFPT
TGFB1
TGFB2
TGFB2−AS1
TGFBI
TGFBR1
TGFBR2
TGM1
THAP6
THEM5
THEMIS
THNSL1
THOC5
THOP1
THSD7A
THUMPD3
THYN1
TIA1
TICAM1
TICRR
TIFA
TIGD3
TIMM21
TIRAP
TK2
TLCD1
TLDC2
TLE4
TLR3
TLR8
TM4SF19
TM7SF3
TM9SF3
TMBIM6
TMC3
TMCC1
25%
Mutation Types
missense_variant
frameshift_variant
stop_gained
start_lost
conservative_inframe_deletion
multi_hit

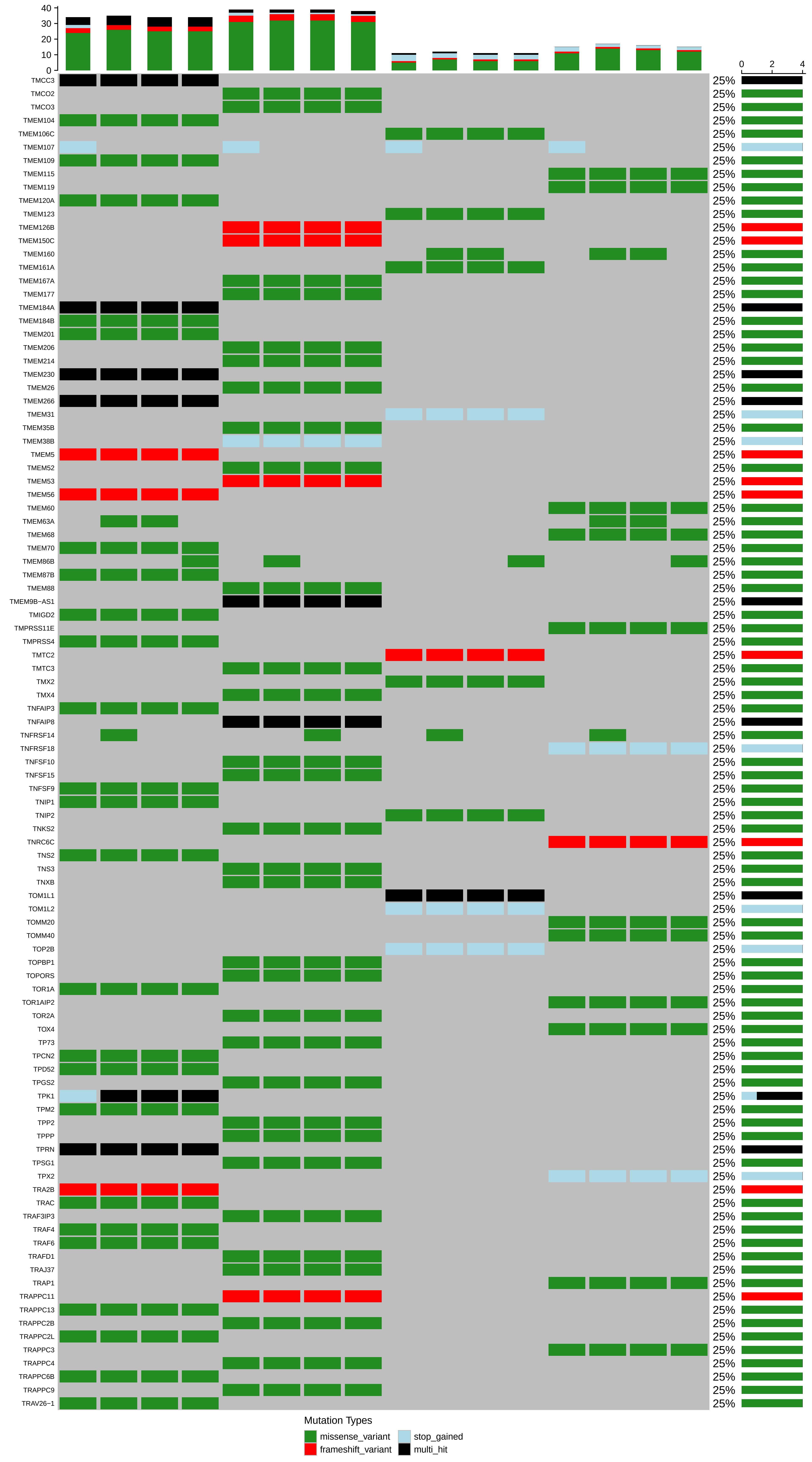
40
30
20
10
0
0
2
4
TMCC3
TMCO2
TMCO3
TMEM104
TMEM106C
TMEM107
TMEM109
TMEM115
TMEM119
TMEM120A
TMEM123
TMEM126B
TMEM150C
TMEM160
TMEM161A
TMEM167A
TMEM177
TMEM184A
TMEM184B
TMEM201
TMEM206
TMEM214
TMEM230
TMEM26
TMEM266
TMEM31
TMEM35B
TMEM38B
TMEM5
TMEM52
TMEM53
TMEM56
TMEM60
TMEM63A
TMEM68
TMEM70
TMEM86B
TMEM87B
TMEM88
TMEM9B−AS1
TMIGD2
TMPRSS11E
TMPRSS4
TMTC2
TMTC3
TMX2
TMX4
TNFAIP3
TNFAIP8
TNFRSF14
TNFRSF18
TNFSF10
TNFSF15
TNFSF9
TNIP1
TNIP2
TNKS2
TNRC6C
TNS2
TNS3
TNXB
TOM1L1
TOM1L2
TOMM20
TOMM40
TOP2B
TOPBP1
TOPORS
TOR1A
TOR1AIP2
TOR2A
TOX4
TP73
TPCN2
TPD52
TPGS2
TPK1
TPM2
TPP2
TPPP
TPRN
TPSG1
TPX2
TRA2B
TRAC
TRAF3IP3
TRAF4
TRAF6
TRAFD1
TRAJ37
TRAP1
TRAPPC11
TRAPPC13
TRAPPC2B
TRAPPC2L
TRAPPC3
TRAPPC4
TRAPPC6B
TRAPPC9
TRAV26−1
25%
Mutation Types
missense_variant
frameshift_variant
stop_gained
multi_hit

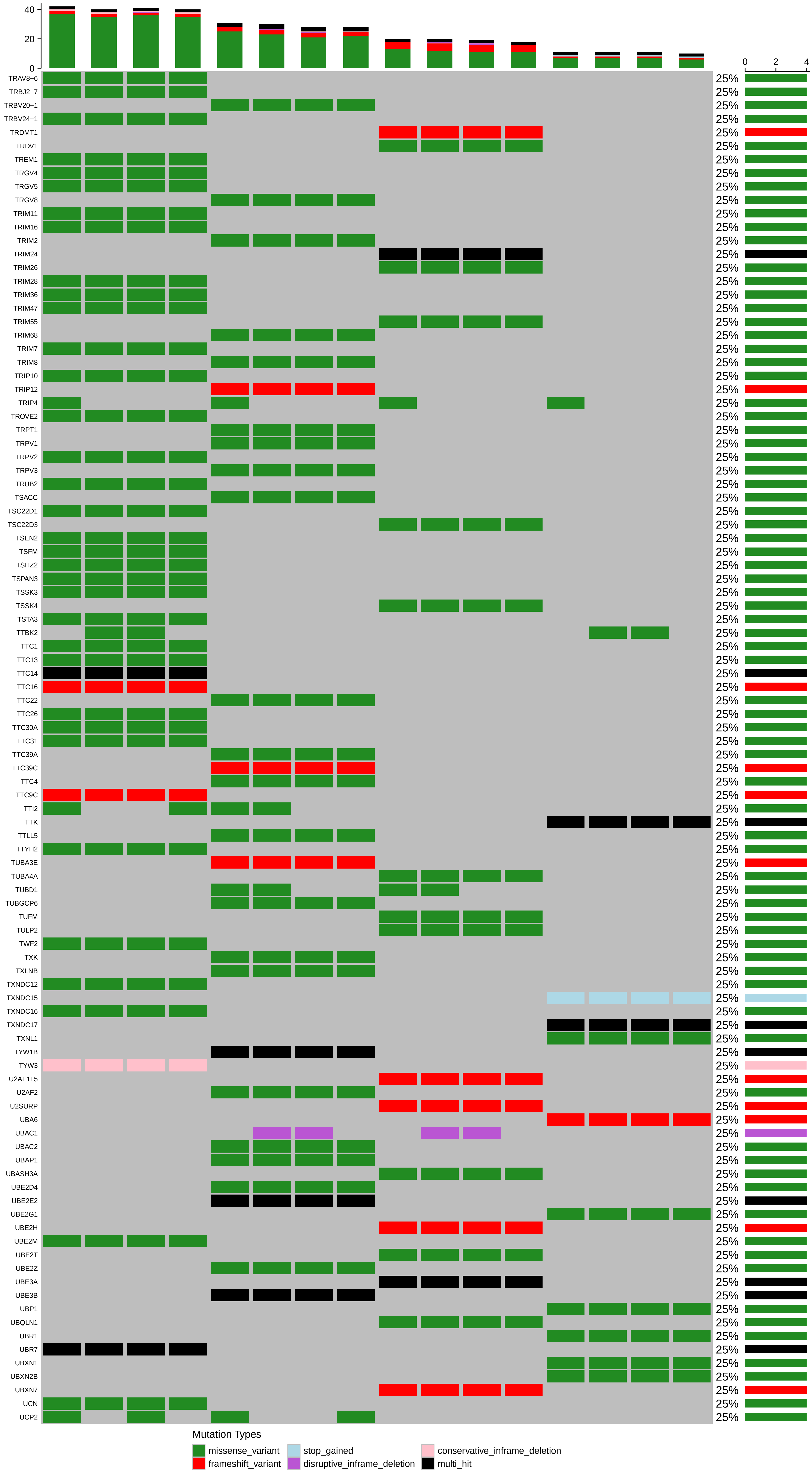
40
20
0
0
2
4
TRAV8−6
TRBJ2−7
TRBV20−1
TRBV24−1
TRDMT1
TRDV1
TREM1
TRGV4
TRGV5
TRGV8
TRIM11
TRIM16
TRIM2
TRIM24
TRIM26
TRIM28
TRIM36
TRIM47
TRIM55
TRIM68
TRIM7
TRIM8
TRIP10
TRIP12
TRIP4
TROVE2
TRPT1
TRPV1
TRPV2
TRPV3
TRUB2
TSACC
TSC22D1
TSC22D3
TSEN2
TSFM
TSHZ2
TSPAN3
TSSK3
TSSK4
TSTA3
TTBK2
TTC1
TTC13
TTC14
TTC16
TTC22
TTC26
TTC30A
TTC31
TTC39A
TTC39C
TTC4
TTC9C
TTI2
TTK
TTLL5
TTYH2
TUBA3E
TUBA4A
TUBD1
TUBGCP6
TUFM
TULP2
TWF2
TXK
TXLNB
TXNDC12
TXNDC15
TXNDC16
TXNDC17
TXNL1
TYW1B
TYW3
U2AF1L5
U2AF2
U2SURP
UBA6
UBAC1
UBAC2
UBAP1
UBASH3A
UBE2D4
UBE2E2
UBE2G1
UBE2H
UBE2M
UBE2T
UBE2Z
UBE3A
UBE3B
UBP1
UBQLN1
UBR1
UBR7
UBXN1
UBXN2B
UBXN7
UCN
UCP2
Mutation Types
missense_variant
frameshift_variant
stop_gained
disruptive_inframe_deletion
conservative_inframe_deletion
multi_hit

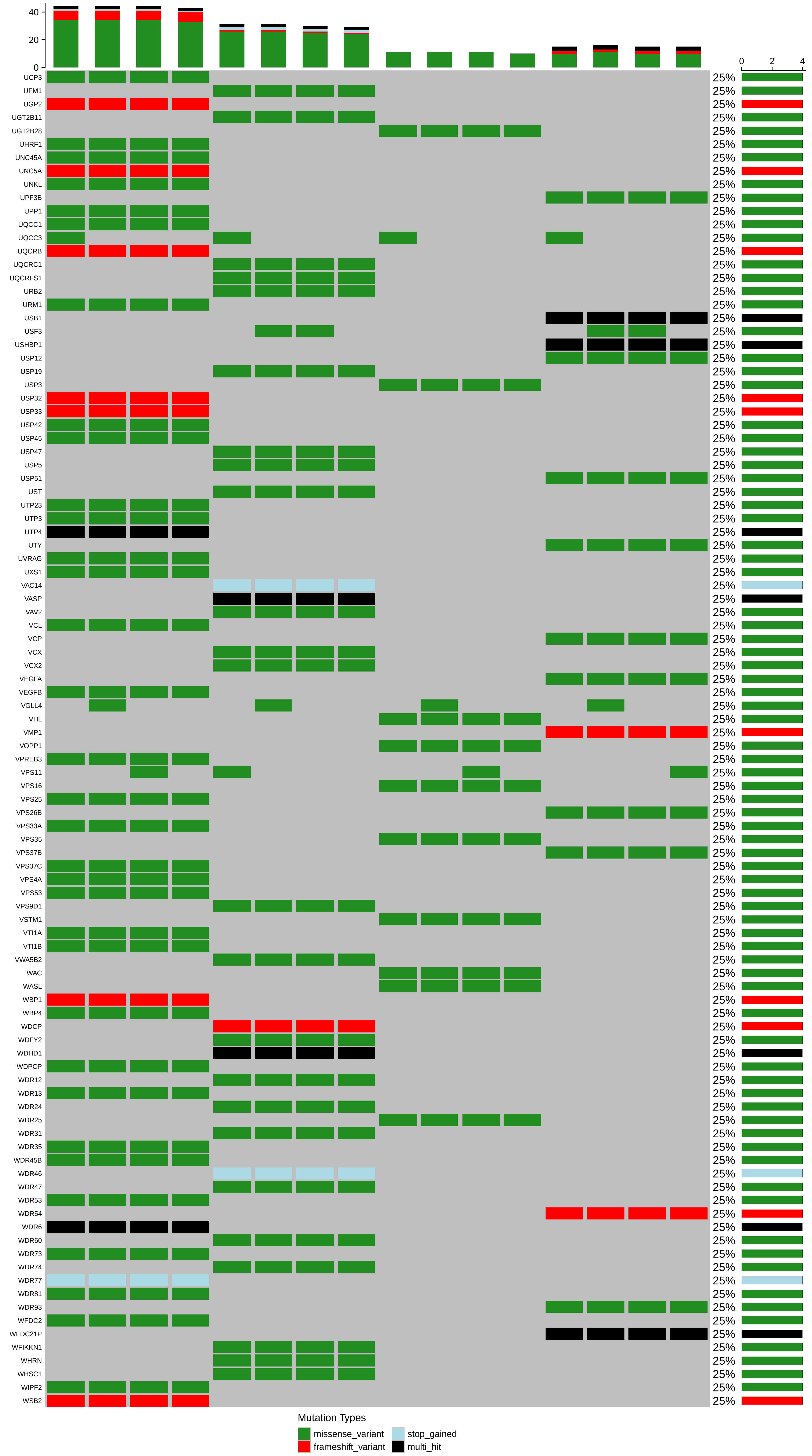

40
20
0
0 2 4
UCP3 25%
UFM1 25%
UGP2 25%
UGT2B11 25%
UGT2B28 25%
UHRF1 25%
UNC45A 25%
UNC5A 25%
UNKL 25%
UPF3B 25%
UPP1 25%
UQCC1 25%
UQCC3 25%
UQCRB 25%
UQCRC1 25%
UQCRFS1 25%
URB2 25%
URM1 25%
USB1 25%
USF3 25%
USHBP1 25%
USP12 25%
USP19 25%
USP3 25%
USP32 25%
USP33 25%
USP42 25%
USP45 25%
USP47 25%
USP5 25%
USP51 25%
UST 25%
UTP23 25%
UTP3 25%
UTP4 25%
UTY 25%
UVRAG 25%
UXS1 25%
VAC14 25%
VASP 25%
VAV2 25%
VCL 25%
VCP 25%
VCX 25%
VCX2 25%
VEGFA 25%
VEGFB 25%
VGLL4 25%
VHL 25%
VMP1 25%
VOPP1 25%
VPREB3 25%
VPS11 25%
VPS16 25%
VPS25 25%
VPS26B 25%
VPS33A 25%
VPS35 25%
VPS37B 25%
VPS37C 25%
VPS4A 25%
VPS53 25%
VPS9D1 25%
VSTM1 25%
VTI1A 25%
VTI1B 25%
VWA5B2 25%
WAC 25%
WASL 25%
WBP1 25%
WBP4 25%
WDCP 25%
WDFY2 25%
WDHD1 25%
WDPCP 25%
WDR12 25%
WDR13 25%
WDR24 25%
WDR25 25%
WDR31 25%
WDR35 25%
WDR45B 25%
WDR46 25%
WDR47 25%
WDR53 25%
WDR54 25%
WDR6 25%
WDR60 25%
WDR73 25%
WDR74 25%
WDR77 25%
WDR81 25%
WDR93 25%
WFDC2 25%
WFDC21P 25%
WFIKKN1 25%
WHRN 25%
WHSC1 25%
WIPF2 25%
WSB2 25%
Mutation Types
missense_variant
frameshift_variant
stop_gained
multi_hit

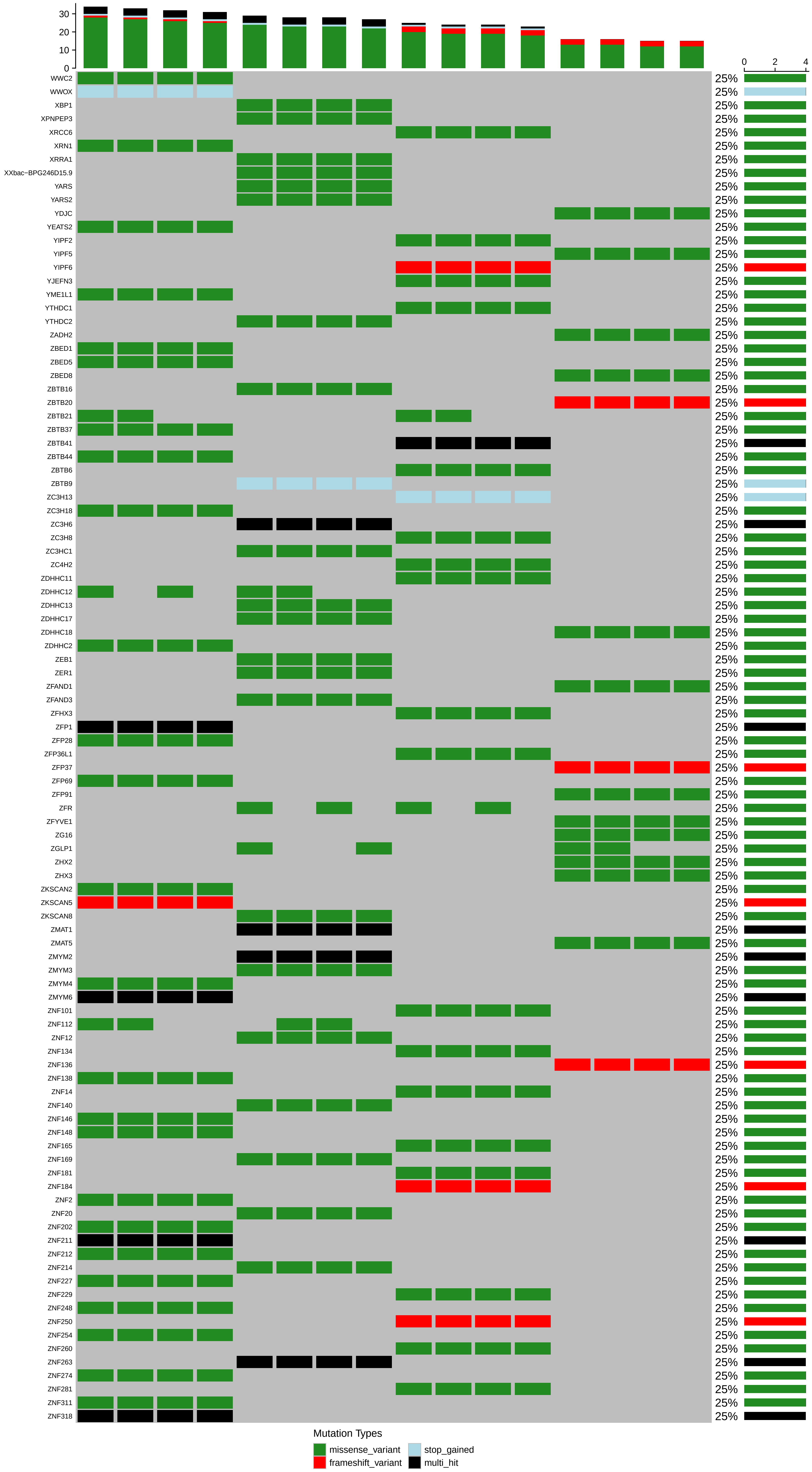

30
20
10
0
WWC2
WWOX
XBP1
XPNPEP3
XRCC6
XRN1
XRRA1
XXbac−BPG246D15.9
YARS
YARS2
YDJC
YEATS2
YIPF2
YIPF5
YIPF6
YJEFN3
YME1L1
YTHDC1
YTHDC2
ZADH2
ZBED1
ZBED5
ZBED8
ZBTB16
ZBTB20
ZBTB21
ZBTB37
ZBTB41
ZBTB44
ZBTB6
ZBTB9
ZC3H13
ZC3H18
ZC3H6
ZC3H8
ZC3HC1
ZC4H2
ZDHHC11
ZDHHC12
ZDHHC13
ZDHHC17
ZDHHC18
ZDHHC2
ZEB1
ZER1
ZFAND1
ZFAND3
ZFHX3
ZFP1
ZFP28
ZFP36L1
ZFP37
ZFP69
ZFP91
ZFR
ZFYVE1
ZG16
ZGLP1
ZHX2
ZHX3
ZKSCAN2
ZKSCAN5
ZKSCAN8
ZMAT1
ZMAT5
ZMYM2
ZMYM3
ZMYM4
ZMYM6
ZNF101
ZNF112
ZNF12
ZNF134
ZNF136
ZNF138
ZNF14
ZNF140
ZNF146
ZNF148
ZNF165
ZNF169
ZNF181
ZNF184
ZNF2
ZNF20
ZNF202
ZNF211
ZNF212
ZNF214
ZNF227
ZNF229
ZNF248
ZNF250
ZNF254
ZNF260
ZNF263
ZNF274
ZNF281
ZNF311
ZNF318
25%
0
2
4
Mutation Types
missense_variant
frameshift_variant
stop_gained
multi_hit

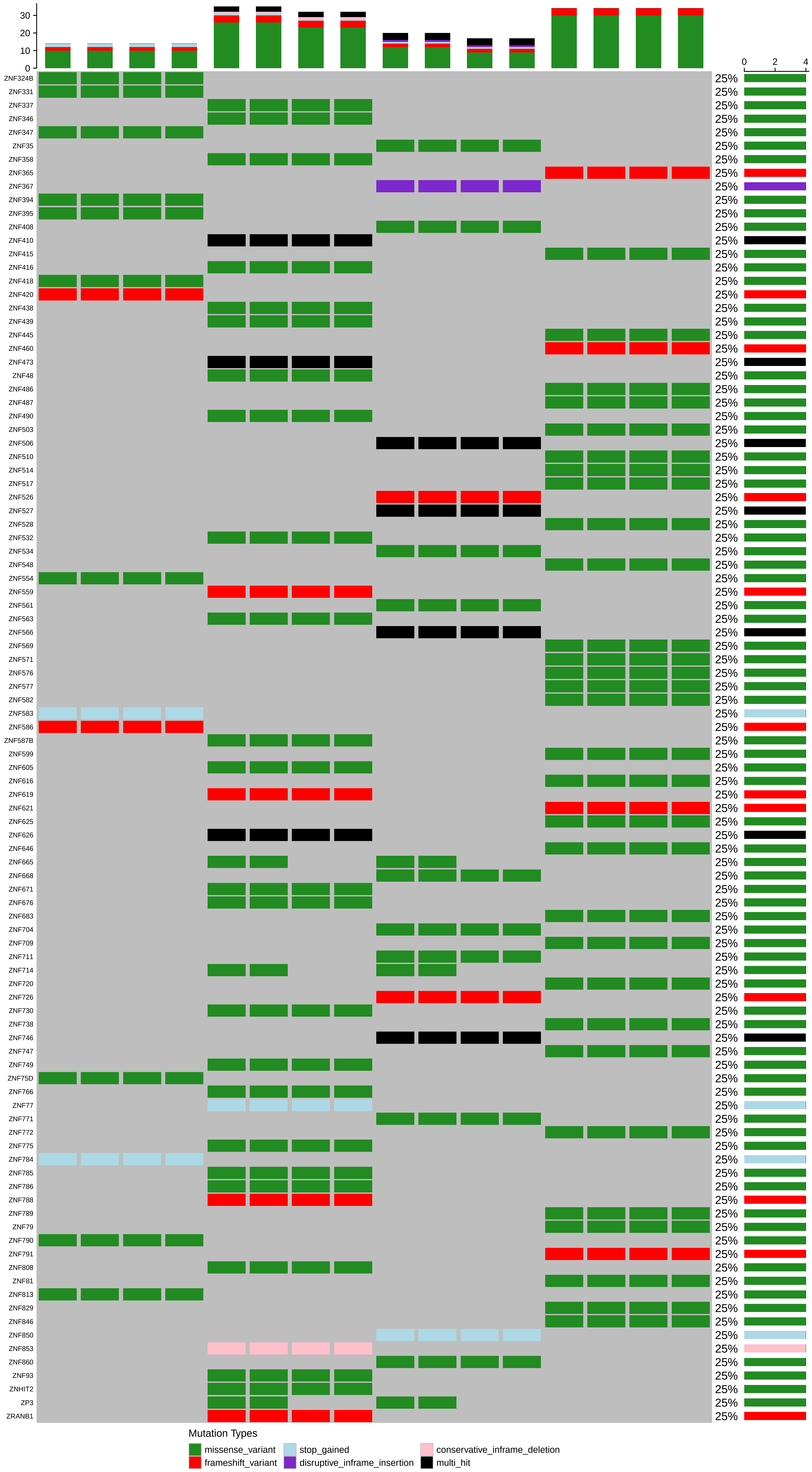

30
20
10
0
0
2
4
ZNF324B
ZNF331
ZNF337
ZNF346
ZNF347
ZNF35
ZNF358
ZNF365
ZNF367
ZNF394
ZNF395
ZNF408
ZNF410
ZNF415
ZNF416
ZNF418
ZNF420
ZNF438
ZNF439
ZNF445
ZNF460
ZNF473
ZNF48
ZNF486
ZNF487
ZNF490
ZNF503
ZNF506
ZNF510
ZNF514
ZNF517
ZNF526
ZNF527
ZNF528
ZNF532
ZNF534
ZNF548
ZNF554
ZNF559
ZNF561
ZNF563
ZNF566
ZNF569
ZNF571
ZNF576
ZNF577
ZNF582
ZNF583
ZNF586
ZNF587B
ZNF599
ZNF605
ZNF616
ZNF619
ZNF621
ZNF625
ZNF626
ZNF646
ZNF665
ZNF668
ZNF671
ZNF676
ZNF683
ZNF704
ZNF709
ZNF711
ZNF714
ZNF720
ZNF726
ZNF730
ZNF738
ZNF746
ZNF747
ZNF749
ZNF75D
ZNF766
ZNF77
ZNF771
ZNF772
ZNF775
ZNF784
ZNF785
ZNF786
ZNF788
ZNF789
ZNF79
ZNF790
ZNF791
ZNF808
ZNF81
ZNF813
ZNF829
ZNF846
ZNF850
ZNF853
ZNF860
ZNF93
ZNHIT2
ZP3
ZRANB1
25%
Mutation Types
missense_variant
frameshift_variant
stop_gained
disruptive_inframe_insertion
conservative_inframe_deletion
multi_hit

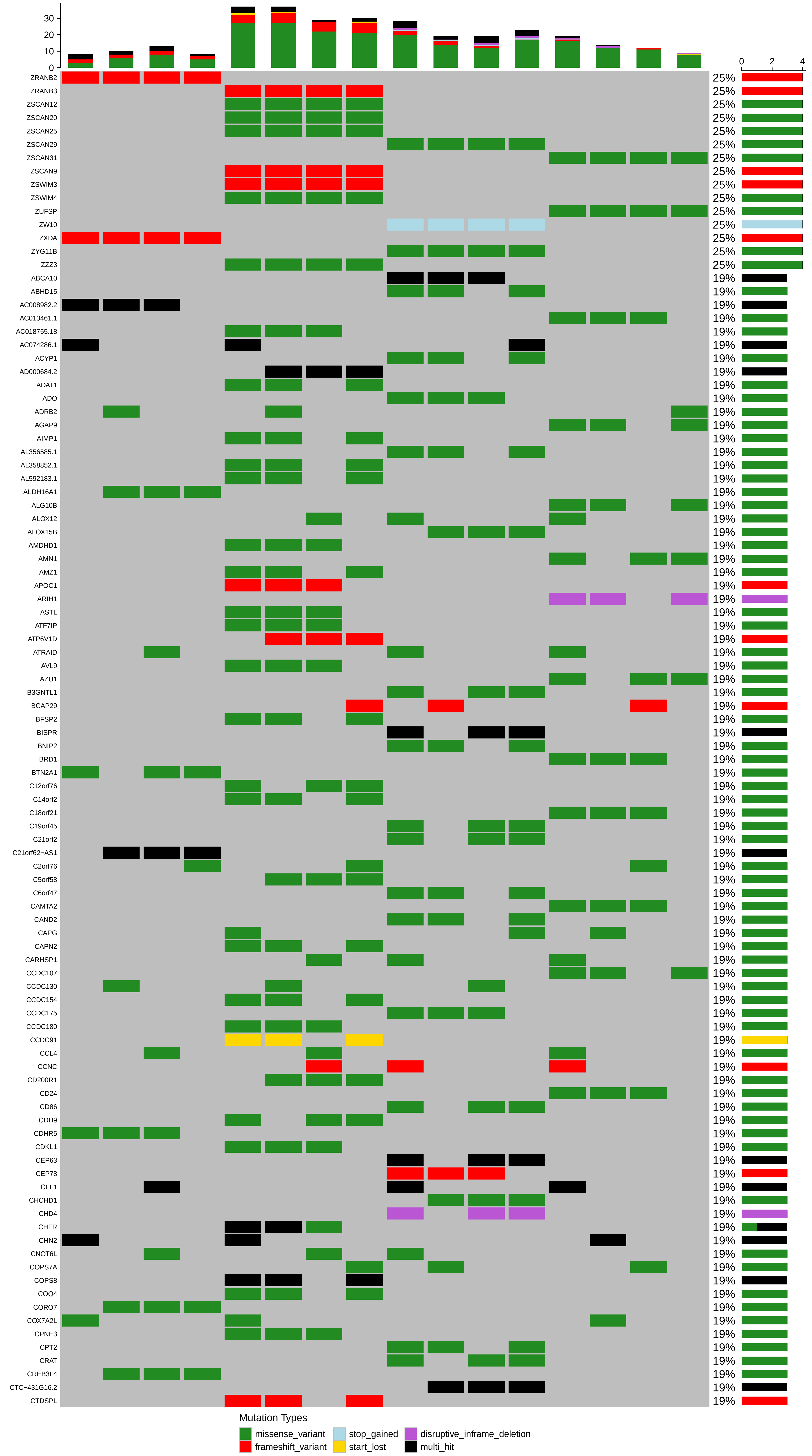

30
20
10
0
0
2
4
ZRANB2 25%
ZRANB3 25%
ZSCAN12 25%
ZSCAN20 25%
ZSCAN25 25%
ZSCAN29 25%
ZSCAN31 25%
ZSCAN9 25%
ZSWIM3 25%
ZSWIM4 25%
ZUFSP 25%
ZW10 25%
ZXDA 25%
ZYG11B 25%
ZZZ3 25%
ABCA10 19%
ABHD15 19%
AC008982.2 19%
AC013461.1 19%
AC018755.18 19%
AC074286.1 19%
ACYP1 19%
AD000684.2 19%
ADAT1 19%
ADO 19%
ADRB2 19%
AGAP9 19%
AIMP1 19%
AL356585.1 19%
AL358852.1 19%
AL592183.1 19%
ALDH16A1 19%
ALG10B 19%
ALOX12 19%
ALOX15B 19%
AMDHD1 19%
AMN1 19%
AMZ1 19%
APOC1 19%
ARIH1 19%
ASTL 19%
ATF7IP 19%
ATP6V1D 19%
ATRAID 19%
AVL9 19%
AZU1 19%
B3GNTL1 19%
BCAP29 19%
BFSP2 19%
BISPR 19%
BNIP2 19%
BRD1 19%
BTN2A1 19%
C12orf76 19%
C14orf2 19%
C18orf21 19%
C19orf45 19%
C21orf2 19%
C21orf62−AS1 19%
C2orf76 19%
C5orf58 19%
C6orf47 19%
CAMTA2 19%
CAND2 19%
CAPG 19%
CAPN2 19%
CARHSP1 19%
CCDC107 19%
CCDC130 19%
CCDC154 19%
CCDC175 19%
CCDC180 19%
CCDC91 19%
CCL4 19%
CCNC 19%
CD200R1 19%
CD24 19%
CD86 19%
CDH9 19%
CDHR5 19%
CDKL1 19%
CEP63 19%
CEP78 19%
CFL1 19%
CHCHD1 19%
CHD4 19%
CHFR 19%
CHN2 19%
CNOT6L 19%
COPS7A 19%
COPS8 19%
COQ4 19%
CORO7 19%
COX7A2L 19%
CPNE3 19%
CPT2 19%
CRAT 19%
CREB3L4 19%
CTC−431G16.2 19%
CTDSPL 19%
Mutation Types
missense_variant
frameshift_variant
stop_gained
start_lost
disruptive_inframe_deletion
multi_hit

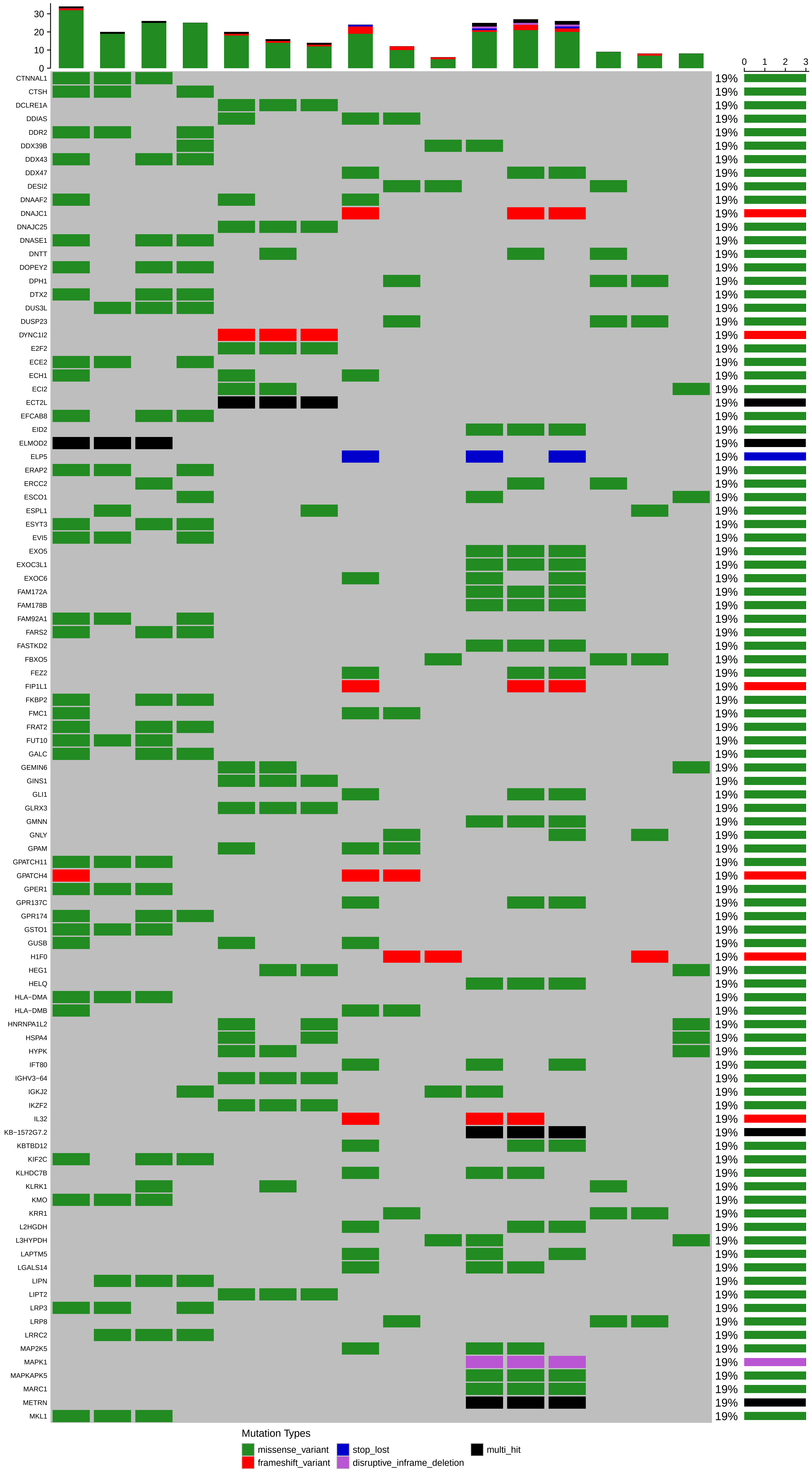

CTNNAL1
CTSH
DCLRE1A
DDIAS
DDR2
DDX39B
DDX43
DDX47
DESI2
DNAAF2
DNAJC1
DNAJC25
DNASE1
DNTT
DOPEY2
DPH1
DTX2
DUS3L
DUSP23
DYNC1I2
E2F2
ECE2
ECH1
ECI2
ECT2L
EFCAB8
EID2
ELMOD2
ELP5
ERAP2
ERCC2
ESCO1
ESPL1
ESYT3
EVI5
EXO5
EXOC3L1
EXOC6
FAM172A
FAM178B
FAM92A1
FARS2
FASTKD2
FBXO5
FEZ2
FIP1L1
FKBP2
FMC1
FRAT2
FUT10
GALC
GEMIN6
GINS1
GLI1
GLRX3
GMNN
GNLY
GPAM
GPATCH11
GPATCH4
GPER1
GPR137C
GPR174
GSTO1
GUSB
H1F0
HEG1
HELQ
HLA−DMA
HLA−DMB
HNRNPA1L2
HSPA4
HYPK
IFT80
IGHV3−64
IGKJ2
IKZF2
IL32
KB−1572G7.2
KBTBD12
KIF2C
KLHDC7B
KLRK1
KMO
KRR1
L2HGDH
L3HYPDH
LAPTM5
LGALS14
LIPN
LIPT2
LRP3
LRP8
LRRC2
MAP2K5
MAPK1
MAPKAPK5
MARC1
METRN
MKL1
Mutation Types
missense_variant
frameshift_variant
stop_lost
disruptive_inframe_deletion
multi_hit

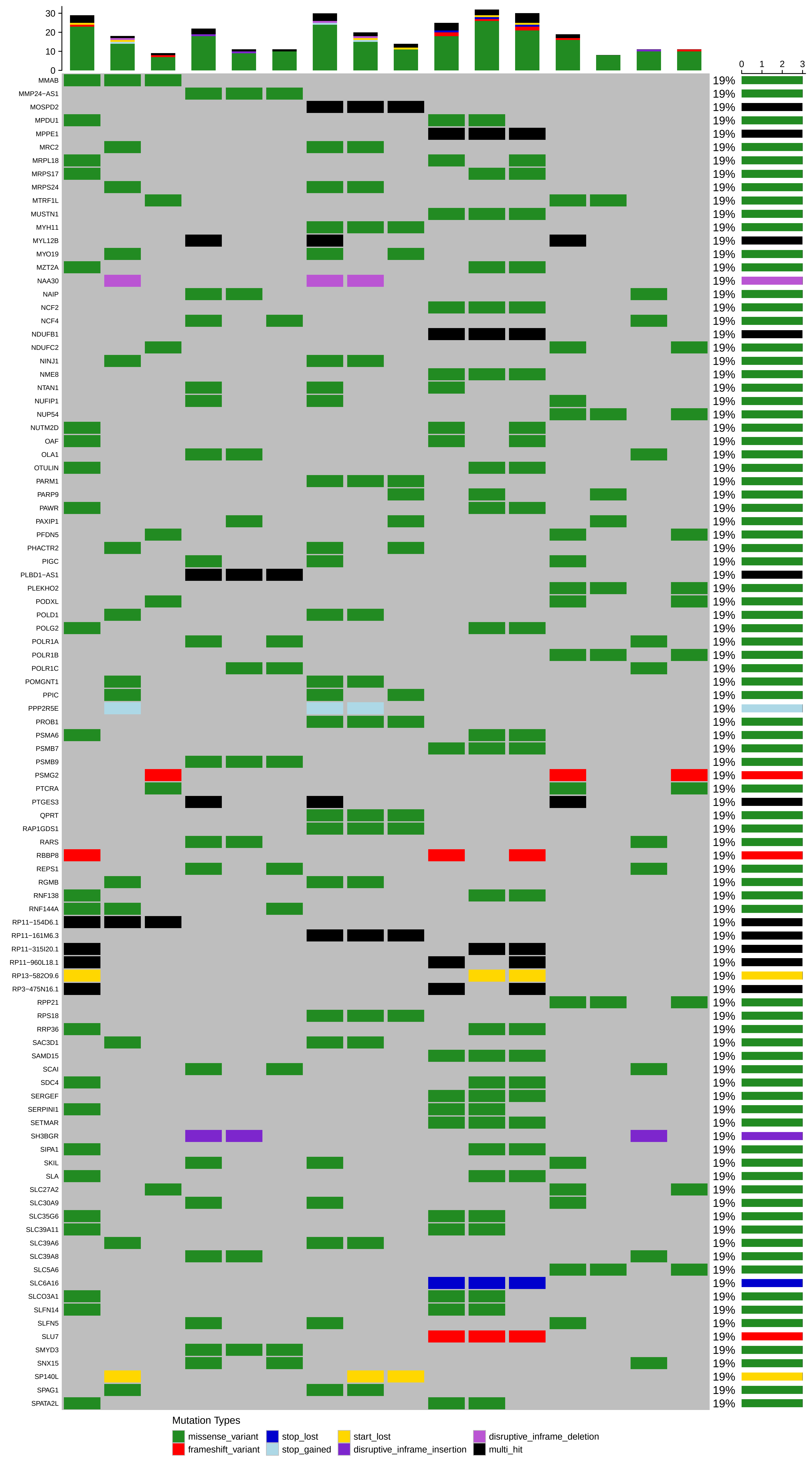

30
20
10
0
MMAB
MMP24−AS1
MOSPD2
MPDU1
MPPE1
MRC2
MRPL18
MRPS17
MRPS24
MTRF1L
MUSTN1
MYH11
MYL12B
MYO19
MZT2A
NAA30
NAIP
NCF2
NCF4
NDUFB1
NDUFC2
NINJ1
NME8
NTAN1
NUFIP1
NUP54
NUTM2D
OAF
OLA1
OTULIN
PARM1
PARP9
PAWR
PAXIP1
PFDN5
PHACTR2
PIGC
PLBD1−AS1
PLEKHO2
PODXL
POLD1
POLG2
POLR1A
POLR1B
POLR1C
POMGNT1
PPIC
PPP2R5E
PROB1
PSMA6
PSMB7
PSMB9
PSMG2
PTCRA
PTGES3
QPRT
RAP1GDS1
RARS
RBBP8
REPS1
RGMB
RNF138
RNF144A
RP11−154D6.1
RP11−161M6.3
RP11−315I20.1
RP11−960L18.1
RP13−582O9.6
RP3−475N16.1
RPP21
RPS18
RRP36
SAC3D1
SAMD15
SCAI
SDC4
SERGEF
SERPINI1
SETMAR
SH3BGR
SIPA1
SKIL
SLA
SLC27A2
SLC30A9
SLC35G6
SLC39A11
SLC39A6
SLC39A8
SLC5A6
SLC6A16
SLCO3A1
SLFN14
SLFN5
SLU7
SMYD3
SNX15
SP140L
SPAG1
SPATA2L
19%
0 1 2 3
Mutation Types
missense_variant
frameshift_variant
stop_lost
stop_gained
start_lost
disruptive_inframe_insertion
disruptive_inframe_deletion
multi_hit

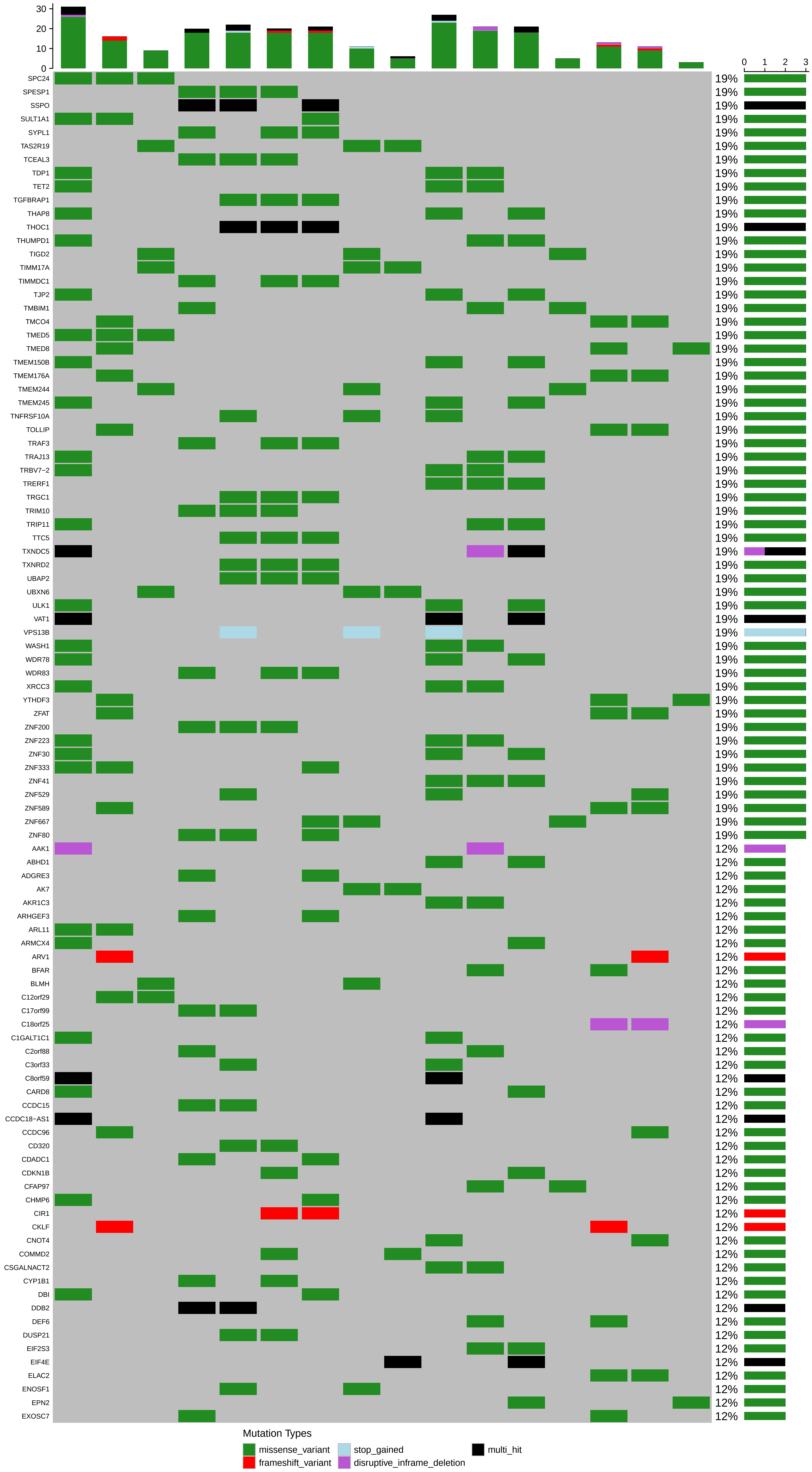

30
20
10
0
0 1 2 3
SPC24 19%
SPESP1 19%
SSPO 19%
SULT1A1 19%
SYPL1 19%
TAS2R19 19%
TCEAL3 19%
TDP1 19%
TET2 19%
TGFBRAP1 19%
THAP8 19%
THOC1 19%
THUMPD1 19%
TIGD2 19%
TIMM17A 19%
TIMMDC1 19%
TJP2 19%
TMBIM1 19%
TMCO4 19%
TMED5 19%
TMED8 19%
TMEM150B 19%
TMEM176A 19%
TMEM244 19%
TMEM245 19%
TNFRSF10A 19%
TOLLIP 19%
TRAF3 19%
TRAJ13 19%
TRBV7−2 19%
TRERF1 19%
TRGC1 19%
TRIM10 19%
TRIP11 19%
TTC5 19%
TXNDC5 19%
TXNRD2 19%
UBAP2 19%
UBXN6 19%
ULK1 19%
VAT1 19%
VPS13B 19%
WASH1 19%
WDR78 19%
WDR83 19%
XRCC3 19%
YTHDF3 19%
ZFAT 19%
ZNF200 19%
ZNF223 19%
ZNF30 19%
ZNF333 19%
ZNF41 19%
ZNF529 19%
ZNF589 19%
ZNF667 19%
ZNF80 19%
AAK1 12%
ABHD1 12%
ADGRE3 12%
AK7 12%
AKR1C3 12%
ARHGEF3 12%
ARL11 12%
ARMCX4 12%
ARV1 12%
BFAR 12%
BLMH 12%
C12orf29 12%
C17orf99 12%
C18orf25 12%
C1GALT1C1 12%
C2orf88 12%
C3orf33 12%
C8orf59 12%
CARD8 12%
CCDC15 12%
CCDC18−AS1 12%
CCDC96 12%
CD320 12%
CDADC1 12%
CDKN1B 12%
CFAP97 12%
CHMP6 12%
CIR1 12%
CKLF 12%
CNOT4 12%
COMMD2 12%
CSGALNACT2 12%
CYP1B1 12%
DBI 12%
DDB2 12%
DEF6 12%
DUSP21 12%
EIF2S3 12%
EIF4E 12%
ELAC2 12%
ENOSF1 12%
EPN2 12%
EXOSC7 12%
Mutation Types
missense_variant
frameshift_variant
stop_gained
disruptive_inframe_deletion
multi_hit

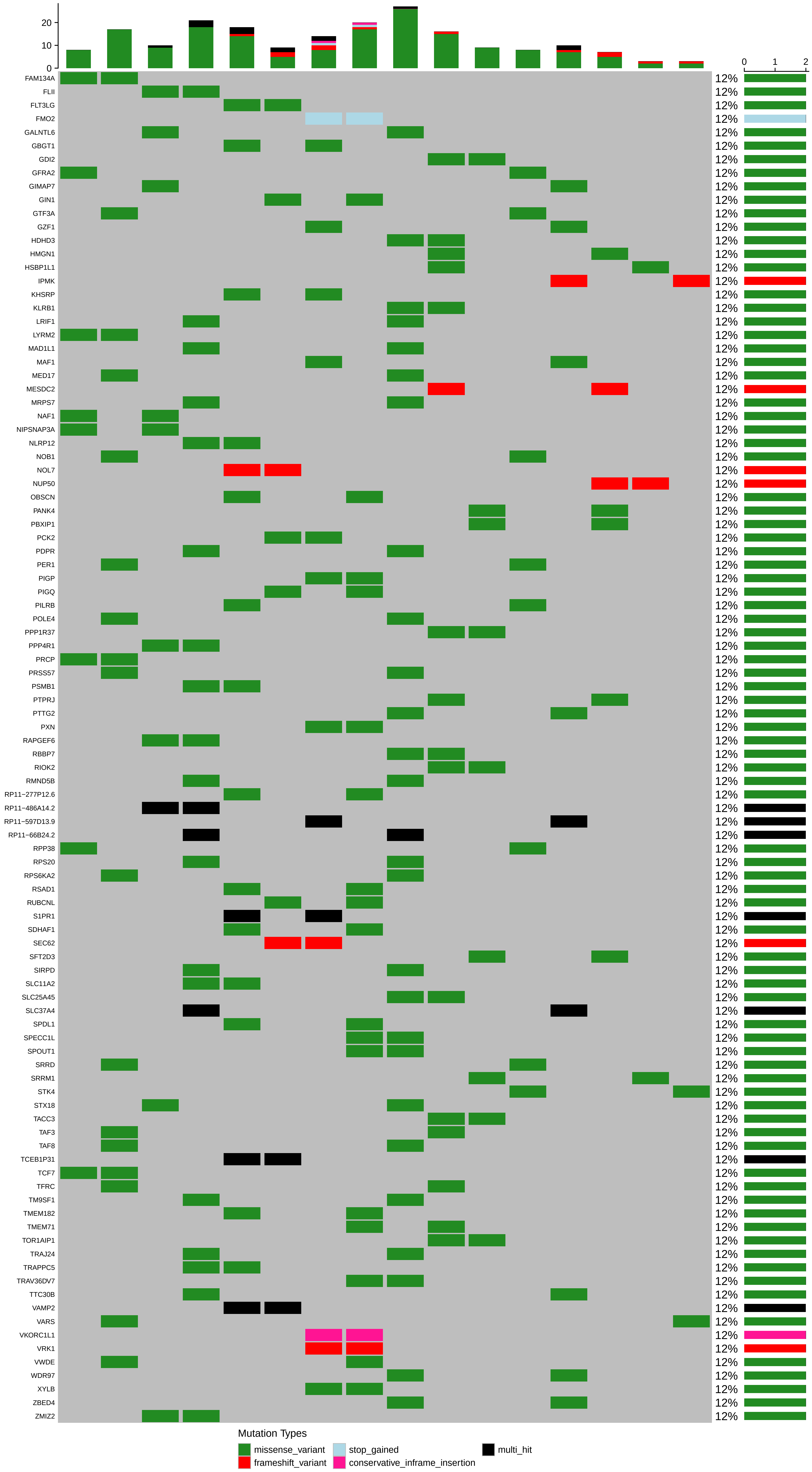
20
10
0
0 1 2
FAM134A 12%
FLII 12%
FLT3LG 12%
FMO2 12%
GALNTL6 12%
GBGT1 12%
GDI2 12%
GFRA2 12%
GIMAP7 12%
GIN1 12%
GTF3A 12%
GZF1 12%
HDHD3 12%
HMGN1 12%
HSBP1L1 12%
IPMK 12%
KHSRP 12%
KLRB1 12%
LRIF1 12%
LYRM2 12%
MAD1L1 12%
MAF1 12%
MED17 12%
MESDC2 12%
MRPS7 12%
NAF1 12%
NIPSNAP3A 12%
NLRP12 12%
NOB1 12%
NOL7 12%
NUP50 12%
OBSCN 12%
PANK4 12%
PBXIP1 12%
PCK2 12%
PDPR 12%
PER1 12%
PIGP 12%
PIGQ 12%
PILRB 12%
POLE4 12%
PPP1R37 12%
PPP4R1 12%
PRCP 12%
PRSS57 12%
PSMB1 12%
PTPRJ 12%
PTTG2 12%
PXN 12%
RAPGEF6 12%
RBBP7 12%
RIOK2 12%
RMND5B 12%
RP11−277P12.6 12%
RP11−486A14.2 12%
RP11−597D13.9 12%
RP11−66B24.2 12%
RPP38 12%
RPS20 12%
RPS6KA2 12%
RSAD1 12%
RUBCNL 12%
S1PR1 12%
SDHAF1 12%
SEC62 12%
SFT2D3 12%
SIRPD 12%
SLC11A2 12%
SLC25A45 12%
SLC37A4 12%
SPDL1 12%
SPECC1L 12%
SPOUT1 12%
SRRD 12%
SRRM1 12%
STK4 12%
STX18 12%
TACC3 12%
TAF3 12%
TAF8 12%
TCEB1P31 12%
TCF7 12%
TFRC 12%
TM9SF1 12%
TMEM182 12%
TMEM71 12%
TOR1AIP1 12%
TRAJ24 12%
TRAPPC5 12%
TRAV36DV7 12%
TTC30B 12%
VAMP2 12%
VARS 12%
VKORC1L1 12%
VRK1 12%
VWDE 12%
WDR97 12%
XYLB 12%
ZBED4 12%
ZMIZ2 12%
Mutation Types
missense_variant
frameshift_variant
stop_gained
conservative_inframe_insertion
multi_hit

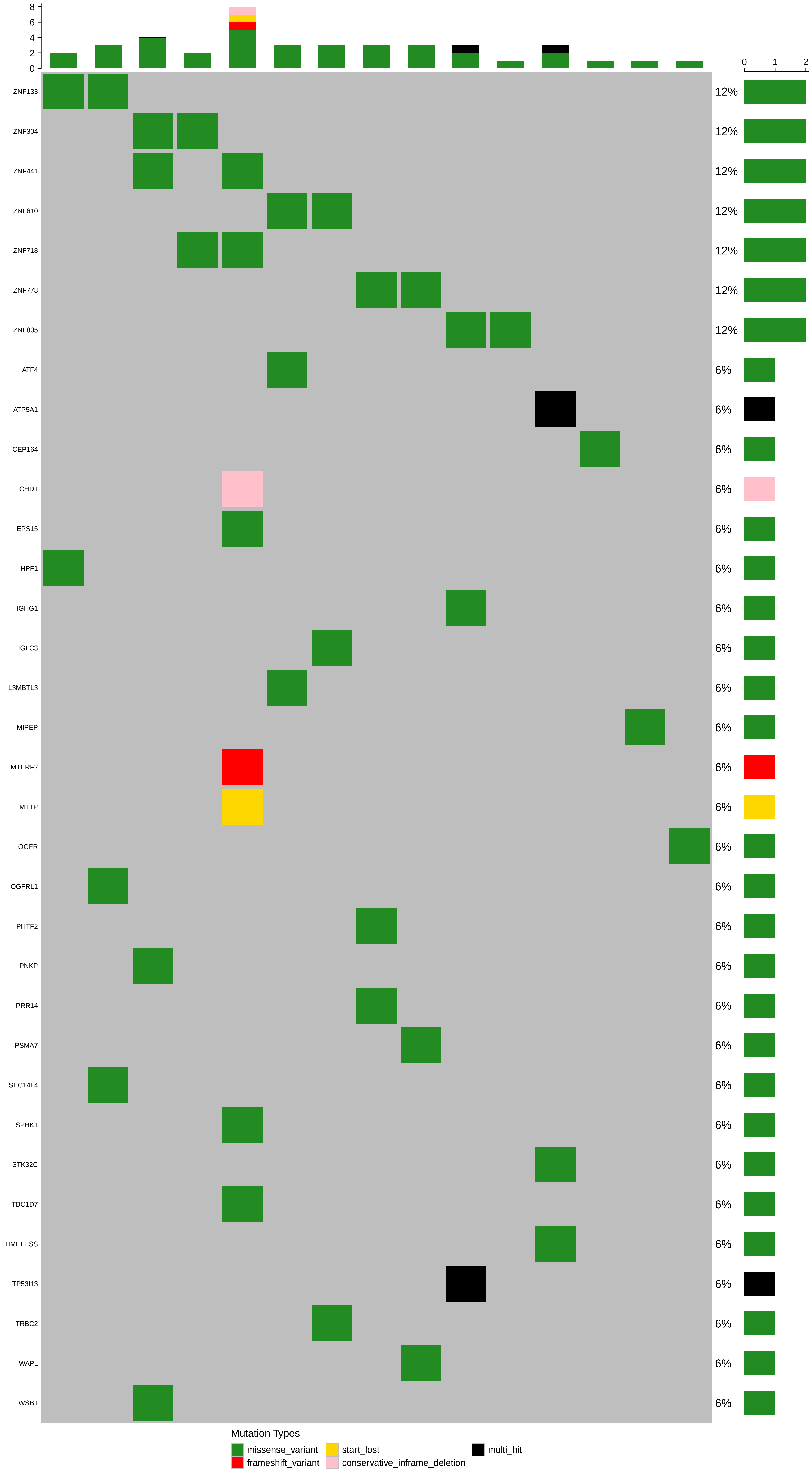
ZNF133
ZNF304
ZNF441
ZNF610
ZNF718
ZNF778
ZNF805
ATF4
ATP5A1
CEP164
CHD1
EPS15
HPF1
IGHG1
IGLC3
L3MBTL3
MIPEP
MTERF2
MTTP
OGFR
OGFRL1
PHTF2
PNKP
PRR14
PSMA7
SEC14L4
SPHK1
STK32C
TBC1D7
TIMELESS
TP53I13
TRBC2
WAPL
WSB1
12%
12%
12%
12%
12%
12%
12%
6%
Mutation Types
missense_variant
frameshift_variant
start_lost
conservative_inframe_deletion
multi_hit

Supplementary Figure S1.

Oncoprint of germline mutations identified in every Multiple Myeloma patient−control comparison. Mutation types are color−coded; Each box from left to right represents each comparison. The bar plot above indicates the number of mutations per comparison, while the percentage on the right shows the frequency of each gene mutation.
