# Supplementary material for: Genomic Insights into Tumorigenesis in Newly Diagnosed Multiple Myeloma
Source: Diagnostics (Basel). 2025 Aug 23;15(17):2130. doi: 10.3390/diagnostics15172130 (PMC12428068; doi:10.3390/diagnostics15172130)
Supplement: Supplementary file 1 [file diagnostics-15-02130-s001.zip › Supplementary Figure S2.pdf]

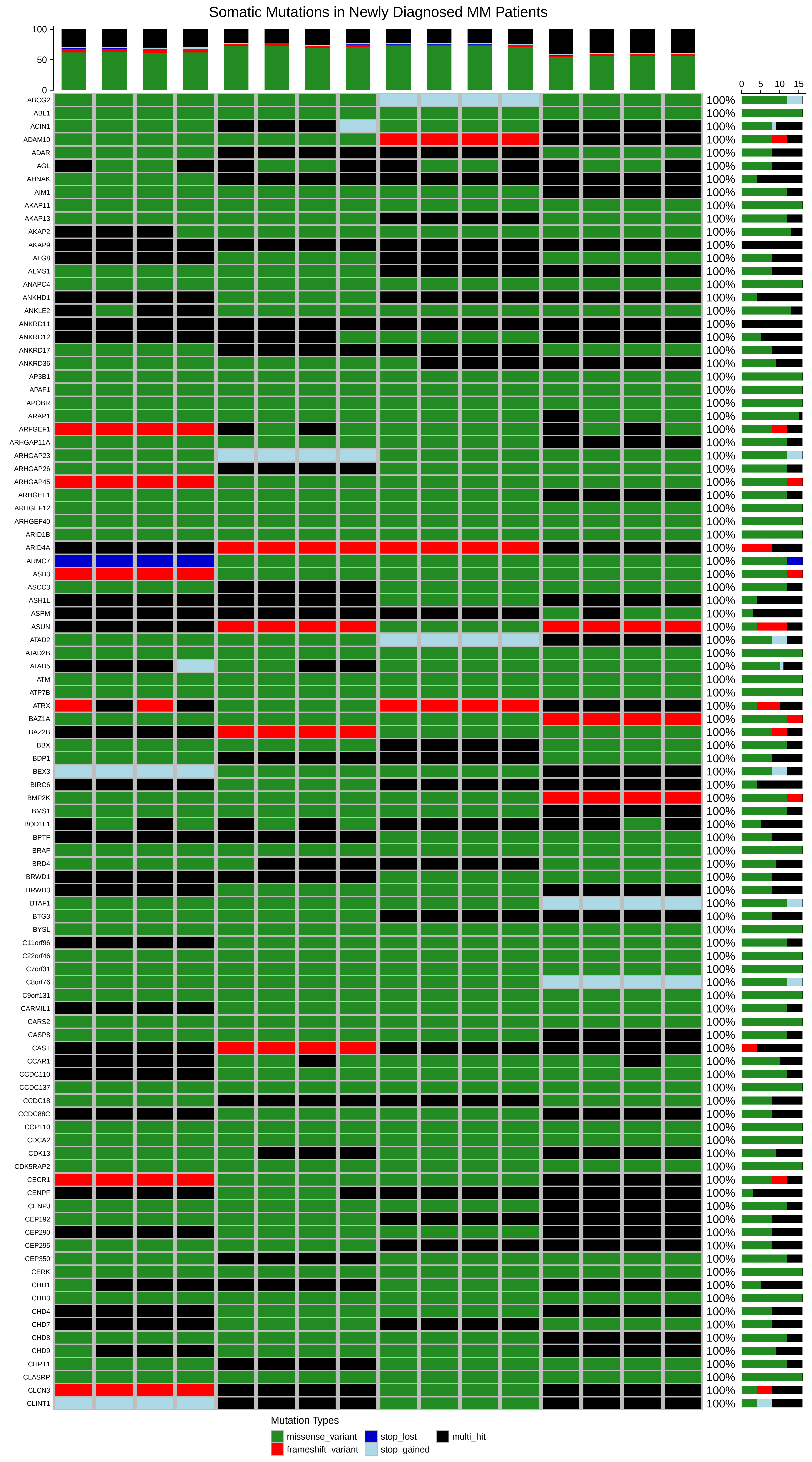

Somatic Mutations in Newly Diagnosed MM Patients
100
50
0
0 5 10 15
ABCG2
ABL1
ACIN1
ADAM10
ADAR
AGL
AHNAK
AIM1
AKAP11
AKAP13
AKAP2
AKAP9
ALG8
ALMS1
ANAPC4
ANKHD1
ANKLE2
ANKRD11
ANKRD12
ANKRD17
ANKRD36
AP3B1
APAF1
APOBR
ARAP1
ARFGEF1
ARHGAP11A
ARHGAP23
ARHGAP26
ARHGAP45
ARHGEF1
ARHGEF12
ARHGEF40
ARID1B
ARID4A
ARMC7
ASB3
ASCC3
ASH1L
ASPM
ASUN
ATAD2
ATAD2B
ATAD5
ATM
ATP7B
ATRX
BAZ1A
BAZ2B
BBX
BDP1
BEX3
BIRC6
BMP2K
BMS1
BOD1L1
BPTF
BRAF
BRD4
BRWD1
BRWD3
BTAF1
BTG3
BYSL
C11orf96
C22orf46
C7orf31
C8orf76
C9orf131
CARMIL1
CARS2
CASP8
CAST
CCAR1
CCDC110
CCDC137
CCDC18
CCDC88C
CCP110
CDCA2
CDK13
CDK5RAP2
CECR1
CENPF
CENPJ
CEP192
CEP290
CEP295
CEP350
CERK
CHD1
CHD3
CHD4
CHD7
CHD8
CHD9
CHPT1
CLASRP
CLCN3
CLINT1
100%
Mutation Types
missense_variant
frameshift_variant
stop_lost
stop_gained
multi_hit

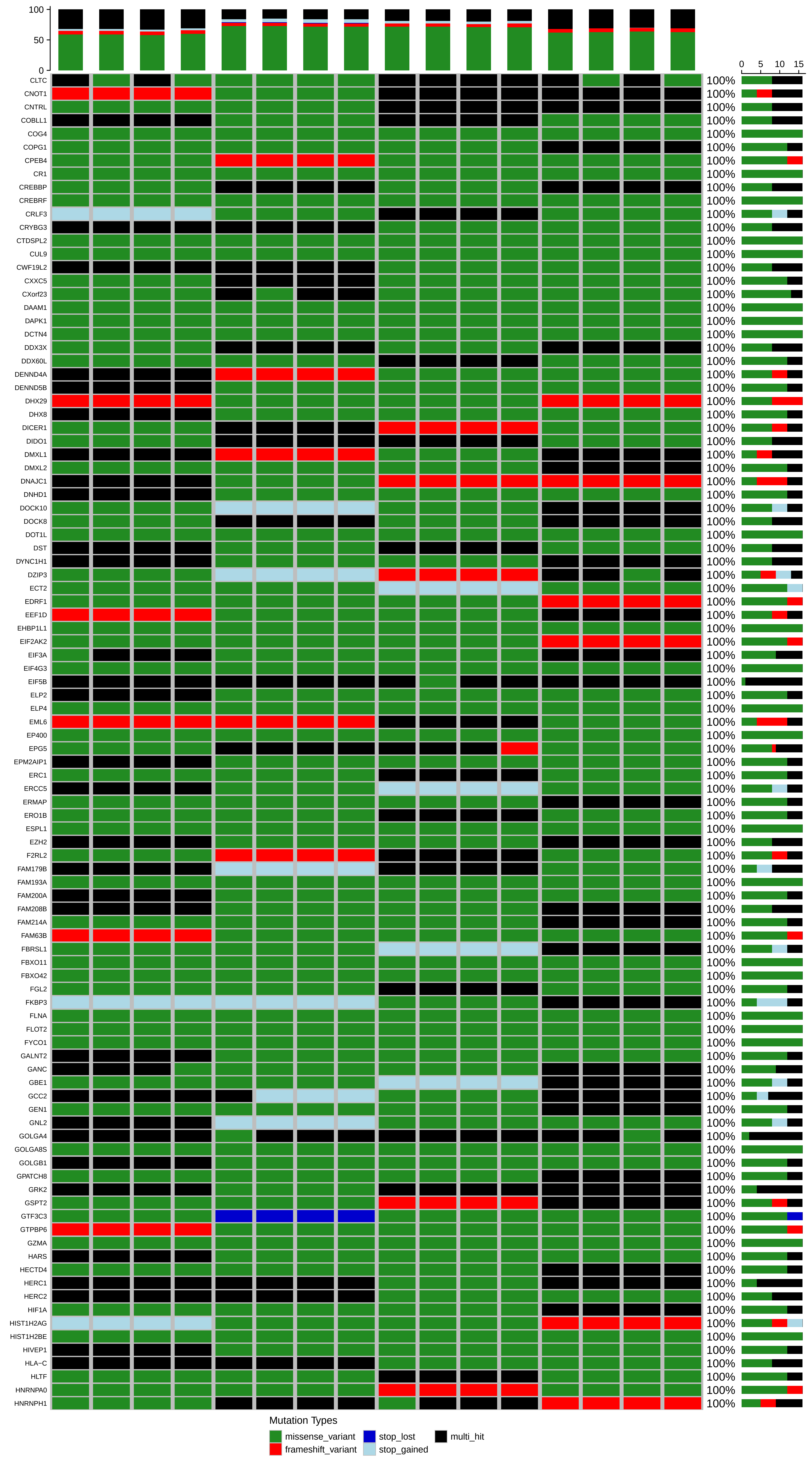
100
50
0
0 5 10 15
CLTC
CNOT1
CNTRL
COBLL1
COG4
COPG1
CPEB4
CR1
CREBBP
CREBRF
CRLF3
CRYBG3
CTDSPL2
CUL9
CWF19L2
CXXC5
CXorf23
DAAM1
DAPK1
DCTN4
DDX3X
DDX60L
DENND4A
DENND5B
DHX29
DHX8
DICER1
DIDO1
DMXL1
DMXL2
DNAJC1
DNHD1
DOCK10
DOCK8
DOT1L
DST
DYNC1H1
DZIP3
ECT2
EDRF1
EEF1D
EHBP1L1
EIF2AK2
EIF3A
EIF4G3
EIF5B
ELP2
ELP4
EML6
EP400
EPG5
EPM2AIP1
ERC1
ERCC5
ERMAP
ERO1B
ESPL1
EZH2
F2RL2
FAM179B
FAM193A
FAM200A
FAM208B
FAM214A
FAM63B
FBRSL1
FBXO11
FBXO42
FGL2
FKBP3
FLNA
FLOT2
FYCO1
GALNT2
GANC
GBE1
GCC2
GEN1
GNL2
GOLGA4
GOLGA8S
GOLGB1
GPATCH8
GRK2
GSPT2
GTF3C3
GTPBP6
GZMA
HARS
HECTD4
HERC1
HERC2
HIF1A
HIST1H2AG
HIST1H2BE
HIVEP1
HLA−C
HLTF
HNRNPA0
HNRNPH1
100%
Mutation Types
missense_variant
frameshift_variant
stop_lost
stop_gained
multi_hit

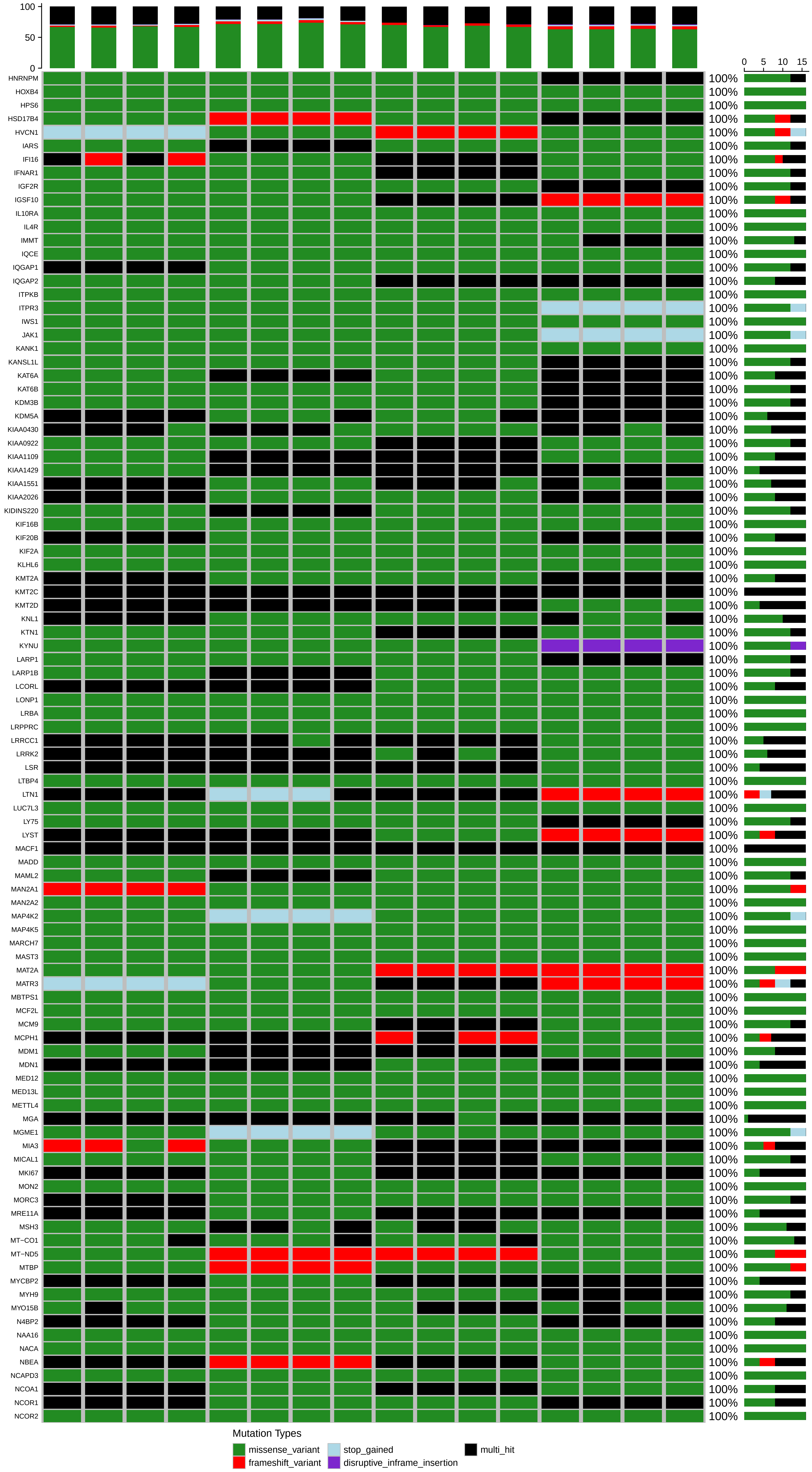
100
50
0
0 5 10 15
HNRNPM
HOXB4
HPS6
HSD17B4
HVCN1
IARS
IFI16
IFNAR1
IGF2R
IGSF10
IL10RA
IL4R
IMMT
IQCE
IQGAP1
IQGAP2
ITPKB
ITPR3
IWS1
JAK1
KANK1
KANSL1L
KAT6A
KAT6B
KDM3B
KDM5A
KIAA0430
KIAA0922
KIAA1109
KIAA1429
KIAA1551
KIAA2026
KIDINS220
KIF16B
KIF20B
KIF2A
KLHL6
KMT2A
KMT2C
KMT2D
KNL1
KTN1
KYNU
LARP1
LARP1B
LCORL
LONP1
LRBA
LRPPRC
LRRCC1
LRRK2
LSR
LTBP4
LTN1
LUC7L3
LY75
LYST
MACF1
MADD
MAML2
MAN2A1
MAN2A2
MAP4K2
MAP4K5
MARCH7
MAST3
MAT2A
MATR3
MBTPS1
MCF2L
MCM9
MCPH1
MDM1
MDN1
MED12
MED13L
METTL4
MGA
MGME1
MIA3
MICAL1
MKI67
MON2
MORC3
MRE11A
MSH3
MT−CO1
MT−ND5
MTBP
MYCBP2
MYH9
MYO15B
N4BP2
NAA16
NACA
NBEA
NCAPD3
NCOA1
NCOR1
NCOR2
100%
Mutation Types
missense_variant
frameshift_variant
stop_gained
disruptive_inframe_insertion
multi_hit

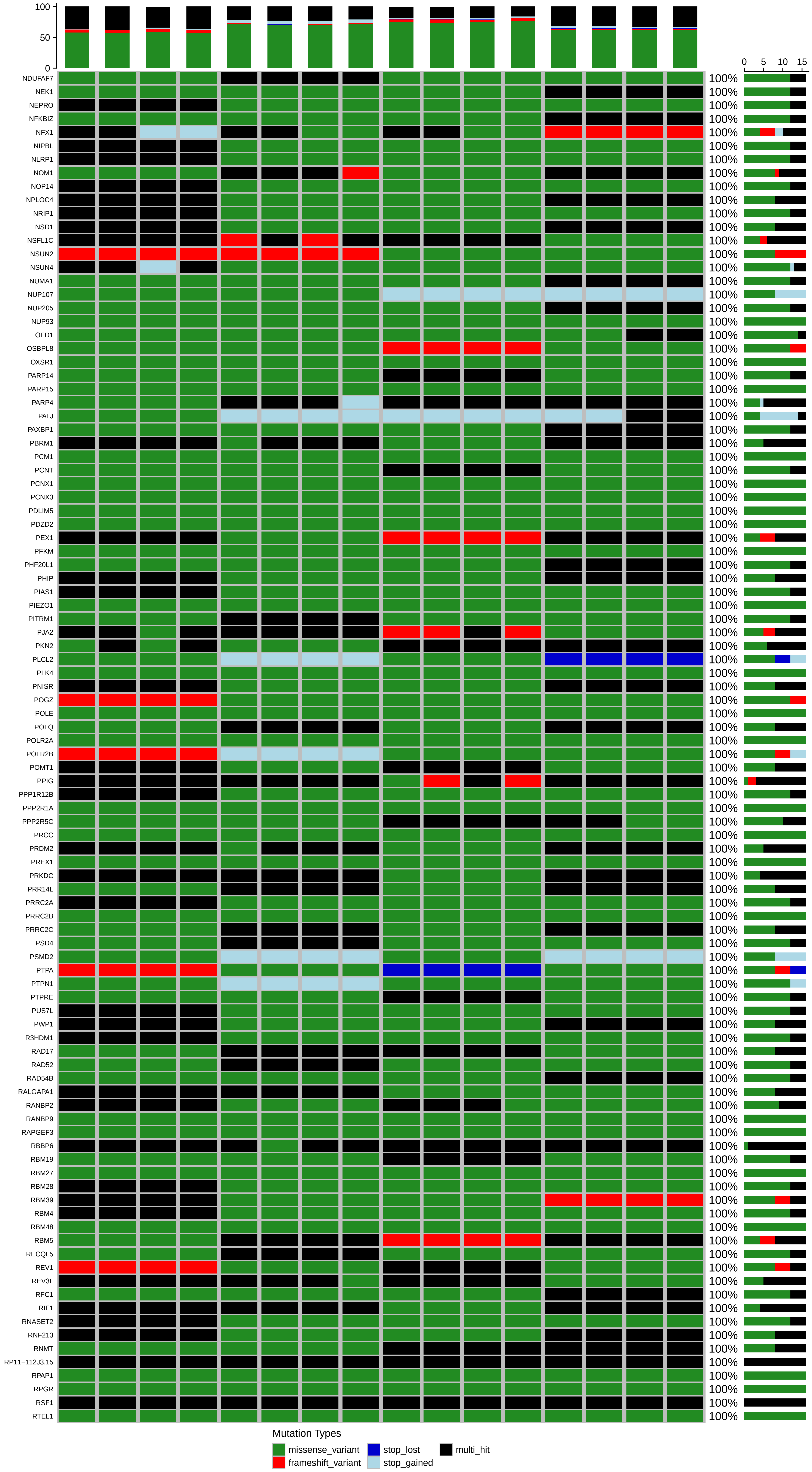

100
50
0
0
5
10
15
NDUFAF7
NEK1
NEPRO
NFKBIZ
NFX1
NIPBL
NLRP1
NOM1
NOP14
NPLOC4
NRIP1
NSD1
NSFL1C
NSUN2
NSUN4
NUMA1
NUP107
NUP205
NUP93
OFD1
OSBPL8
OXSR1
PARP14
PARP15
PARP4
PATJ
PAXBP1
PBRM1
PCM1
PCNT
PCNX1
PCNX3
PDLIM5
PDZD2
PEX1
PFKM
PHF20L1
PHIP
PIAS1
PIEZO1
PITRM1
PJA2
PKN2
PLCL2
PLK4
PNISR
POGZ
POLE
POLQ
POLR2A
POLR2B
POMT1
PPIG
PPP1R12B
PPP2R1A
PPP2R5C
PRCC
PRDM2
PREX1
PRKDC
PRR14L
PRRC2A
PRRC2B
PRRC2C
PSD4
PSMD2
PTPA
PTPN1
PTPRE
PUS7L
PWP1
R3HDM1
RAD17
RAD52
RAD54B
RALGAPA1
RANBP2
RANBP9
RAPGEF3
RBBP6
RBM19
RBM27
RBM28
RBM39
RBM4
RBM48
RBM5
RECQL5
REV1
REV3L
RFC1
RIF1
RNASET2
RNF213
RNMT
RP11−112J3.15
RPAP1
RPGR
RSF1
RTEL1
100%
Mutation Types
missense_variant
frameshift_variant
stop_lost
stop_gained
multi_hit

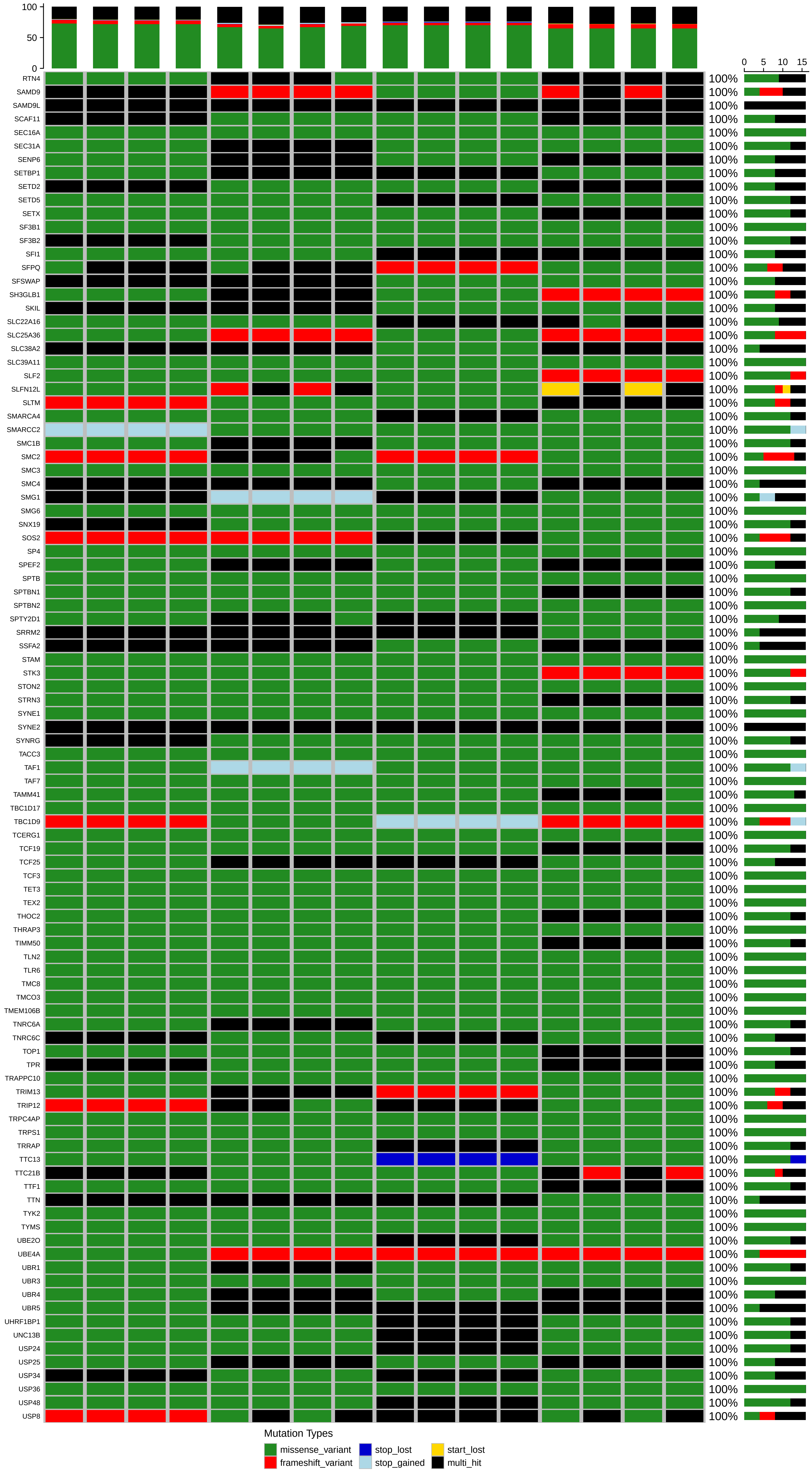

100
50
0
0 5 10 15
RTN4
SAMD9
SAMD9L
SCAF11
SEC16A
SEC31A
SENP6
SETBP1
SETD2
SETD5
SETX
SF3B1
SF3B2
SFI1
SFPQ
SFSWAP
SH3GLB1
SKIL
SLC22A16
SLC25A36
SLC38A2
SLC39A11
SLF2
SLFN12L
SLTM
SMARCA4
SMARCC2
SMC1B
SMC2
SMC3
SMC4
SMG1
SMG6
SNX19
SOS2
SP4
SPEF2
SPTB
SPTBN1
SPTBN2
SPTY2D1
SRRM2
SSFA2
STAM
STK3
STON2
STRN3
SYNE1
SYNE2
SYNRG
TACC3
TAF1
TAF7
TAMM41
TBC1D17
TBC1D9
TCERG1
TCF19
TCF25
TCF3
TET3
TEX2
THOC2
THRAP3
TIMM50
TLN2
TLR6
TMC8
TMCO3
TMEM106B
TNRC6A
TNRC6C
TOP1
TPR
TRAPPC10
TRIM13
TRIP12
TRPC4AP
TRPS1
TRRAP
TTC13
TTC21B
TTF1
TTN
TYK2
TYMS
UBE2O
UBE4A
UBR1
UBR3
UBR4
UBR5
UHRF1BP1
UNC13B
USP24
USP25
USP34
USP36
USP48
USP8
100%
Mutation Types
missense_variant
frameshift_variant
stop_lost
stop_gained
start_lost
multi_hit

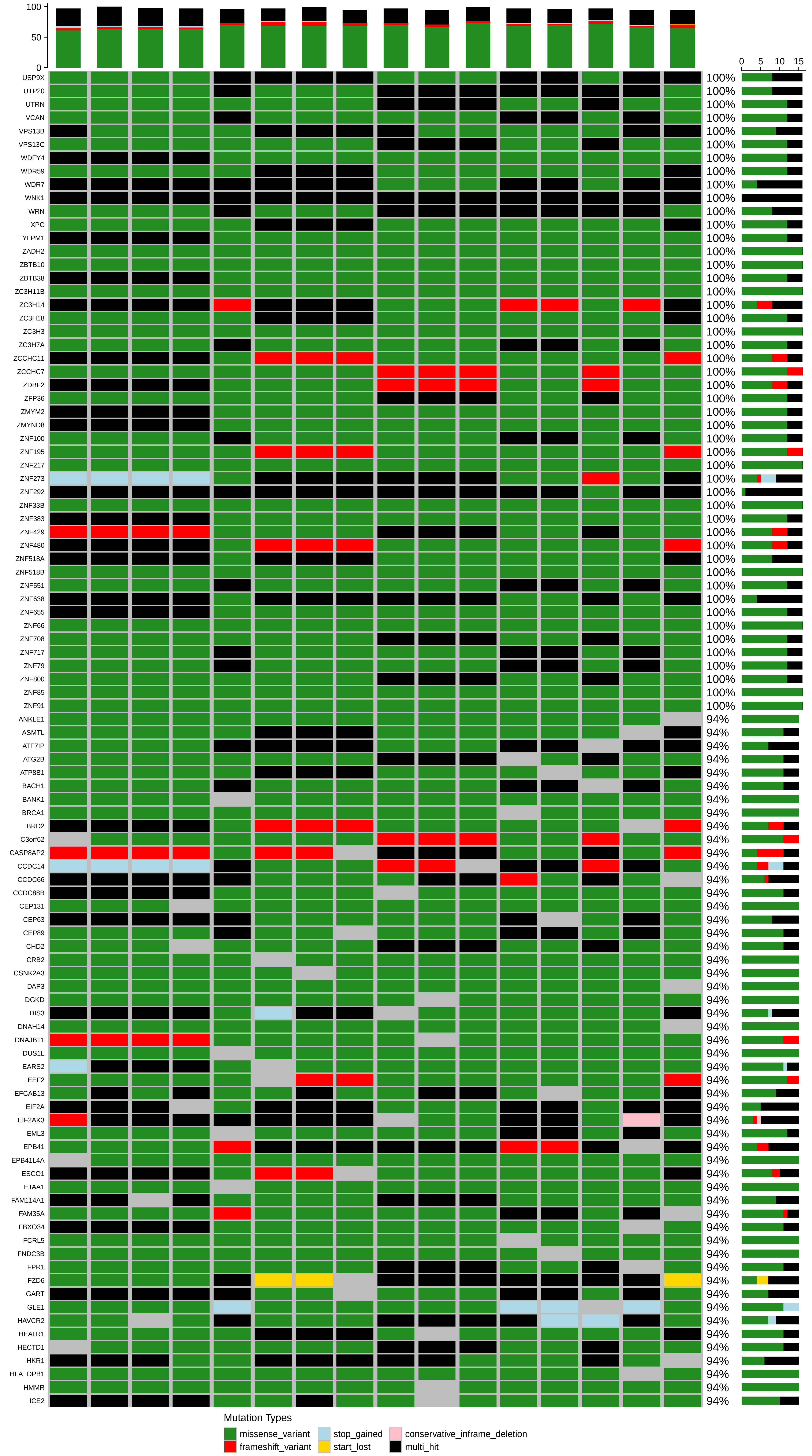
100
50
0
0 5 10 15
USP9X 100%
UTP20 100%
UTRN 100%
VCAN 100%
VPS13B 100%
VPS13C 100%
WDFY4 100%
WDR59 100%
WDR7 100%
WNK1 100%
WRN 100%
XPC 100%
YLPM1 100%
ZADH2 100%
ZBTB10 100%
ZBTB38 100%
ZC3H11B 100%
ZC3H14 100%
ZC3H18 100%
ZC3H3 100%
ZC3H7A 100%
ZCCHC11 100%
ZCCHC7 100%
ZDBF2 100%
ZFP36 100%
ZMYM2 100%
ZMYND8 100%
ZNF100 100%
ZNF195 100%
ZNF217 100%
ZNF273 100%
ZNF292 100%
ZNF33B 100%
ZNF383 100%
ZNF429 100%
ZNF480 100%
ZNF518A 100%
ZNF518B 100%
ZNF551 100%
ZNF638 100%
ZNF655 100%
ZNF66 100%
ZNF708 100%
ZNF717 100%
ZNF79 100%
ZNF800 100%
ZNF85 100%
ZNF91 100%
ANKLE1 94%
ASMTL 94%
ATF7IP 94%
ATG2B 94%
ATP8B1 94%
BACH1 94%
BANK1 94%
BRCA1 94%
BRD2 94%
C3orf62 94%
CASP8AP2 94%
CCDC14 94%
CCDC66 94%
CCDC88B 94%
CEP131 94%
CEP63 94%
CEP89 94%
CHD2 94%
CRB2 94%
CSNK2A3 94%
DAP3 94%
DGKD 94%
DIS3 94%
DNAH14 94%
DNAJB11 94%
DUS1L 94%
EARS2 94%
EEF2 94%
EFCAB13 94%
EIF2A 94%
EIF2AK3 94%
EML3 94%
EPB41 94%
EPB41L4A 94%
ESCO1 94%
ETAA1 94%
FAM114A1 94%
FAM35A 94%
FBXO34 94%
FCRL5 94%
FNDC3B 94%
FPR1 94%
FZD6 94%
GART 94%
GLE1 94%
HAVCR2 94%
HEATR1 94%
HECTD1 94%
HKR1 94%
HLA−DPB1 94%
HMMR 94%
ICE2 94%
Mutation Types
missense_variant
frameshift_variant
stop_gained
start_lost
conservative_inframe_deletion
multi_hit

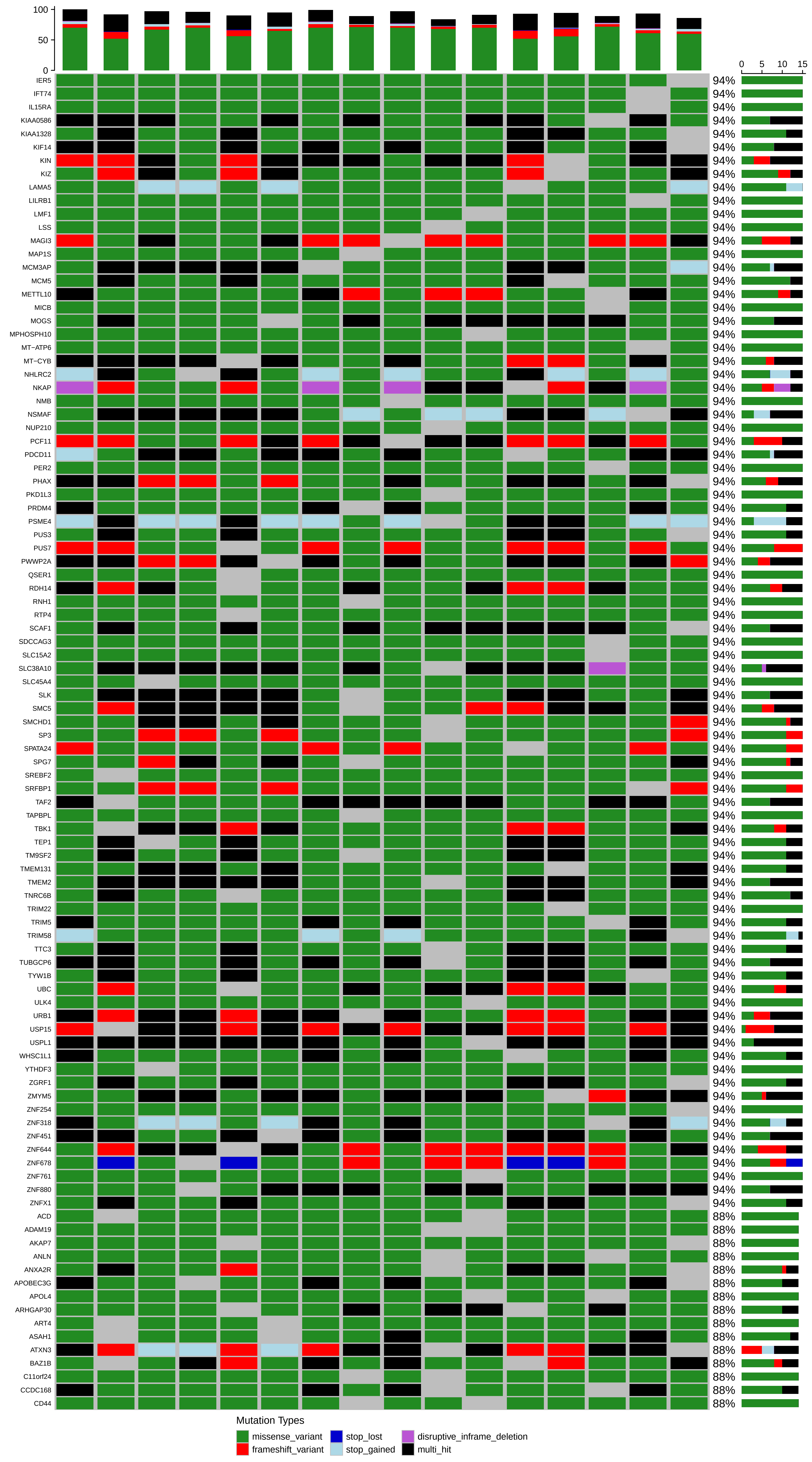

100
50
0
0
5
10
15
IER5
IFT74
IL15RA
KIAA0586
KIAA1328
KIF14
KIN
KIZ
LAMA5
LILRB1
LMF1
LSS
MAGI3
MAP1S
MCM3AP
MCM5
METTL10
MICB
MOGS
MPHOSPH10
MT−ATP6
MT−CYB
NHLRC2
NKAP
NMB
NSMAF
NUP210
PCF11
PDCD11
PER2
PHAX
PKD1L3
PRDM4
PSME4
PUS3
PUS7
PWWP2A
QSER1
RDH14
RNH1
RTP4
SCAF1
SDCCAG3
SLC15A2
SLC38A10
SLC45A4
SLK
SMC5
SMCHD1
SP3
SPATA24
SPG7
SREBF2
SRFBP1
TAF2
TAPBPL
TBK1
TEP1
TM9SF2
TMEM131
TMEM2
TNRC6B
TRIM22
TRIM5
TRIM58
TTC3
TUBGCP6
TYW1B
UBC
ULK4
URB1
USP15
USPL1
WHSC1L1
YTHDF3
ZGRF1
ZMYM5
ZNF254
ZNF318
ZNF451
ZNF644
ZNF678
ZNF761
ZNF880
ZNFX1
ACD
ADAM19
AKAP7
ANLN
ANXA2R
APOBEC3G
APOL4
ARHGAP30
ART4
ASAH1
ATXN3
BAZ1B
C11orf24
CCDC168
CD44
94%
88%
Mutation Types
missense_variant
frameshift_variant
stop_lost
stop_gained
disruptive_inframe_deletion
multi_hit

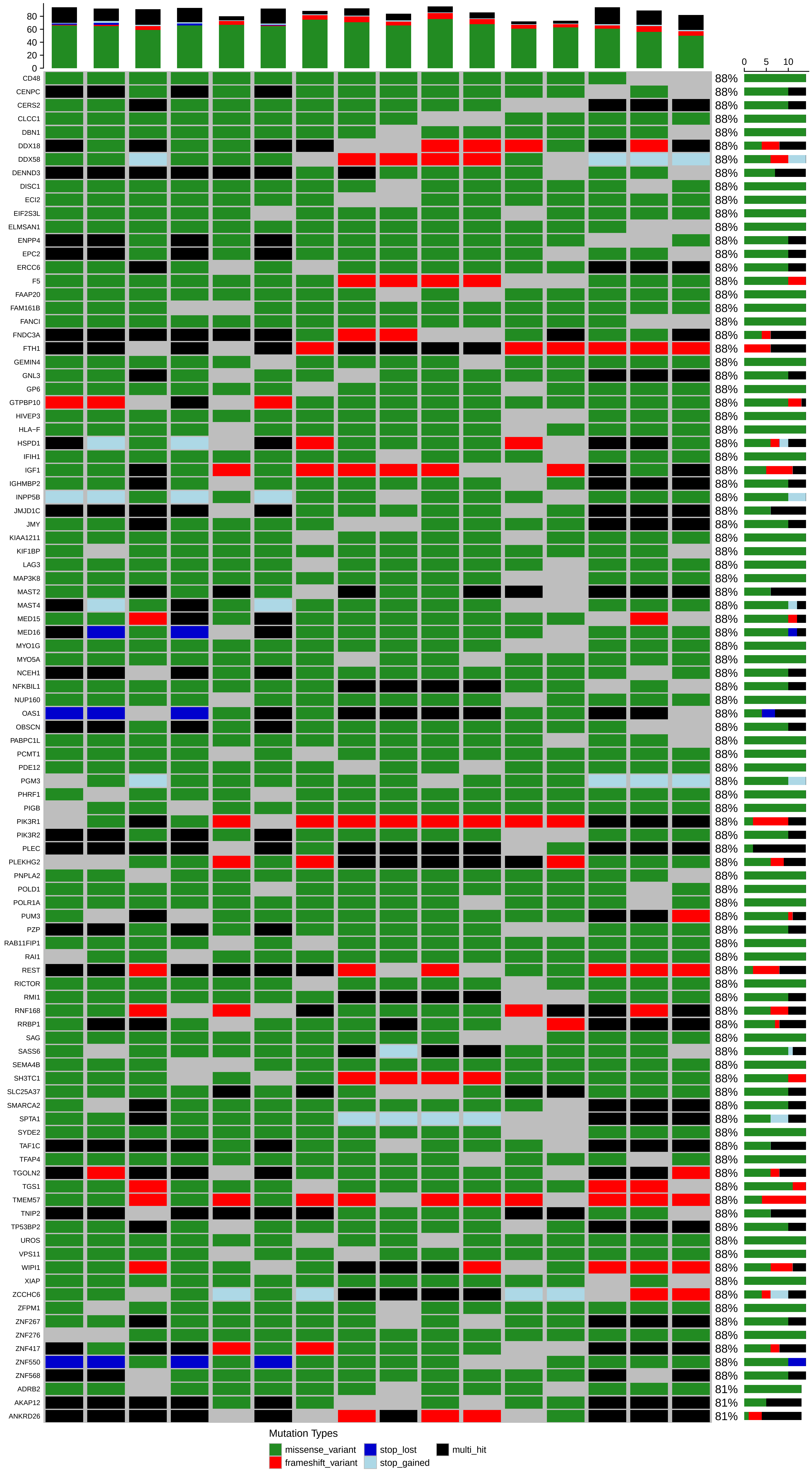

80
60
40
20
0
0
5
10
CD48
CENPC
CERS2
CLCC1
DBN1
DDX18
DDX58
DENND3
DISC1
ECI2
EIF2S3L
ELMSAN1
ENPP4
EPC2
ERCC6
F5
FAAP20
FAM161B
FANCI
FNDC3A
FTH1
GEMIN4
GNL3
GP6
GTPBP10
HIVEP3
HLA−F
HSPD1
IFIH1
IGF1
IGHMBP2
INPP5B
JMJD1C
JMY
KIAA1211
KIF1BP
LAG3
MAP3K8
MAST2
MAST4
MED15
MED16
MYO1G
MYO5A
NCEH1
NFKBIL1
NUP160
OAS1
OBSCN
PABPC1L
PCMT1
PDE12
PGM3
PHRF1
PIGB
PIK3R1
PIK3R2
PLEC
PLEKHG2
PNPLA2
POLD1
POLR1A
PUM3
PZP
RAB11FIP1
RAI1
REST
RICTOR
RMI1
RNF168
RRBP1
SAG
SASS6
SEMA4B
SH3TC1
SLC25A37
SMARCA2
SPTA1
SYDE2
TAF1C
TFAP4
TGOLN2
TGS1
TMEM57
TNIP2
TP53BP2
UROS
VPS11
WIPI1
XIAP
ZCCHC6
ZFPM1
ZNF267
ZNF276
ZNF417
ZNF550
ZNF568
ADRB2
AKAP12
ANKRD26
88% (×98: CD48 through ZNF568)
81% (×3: ADRB2, AKAP12, ANKRD26)
Mutation Types
missense_variant
frameshift_variant
stop_lost
stop_gained
multi_hit

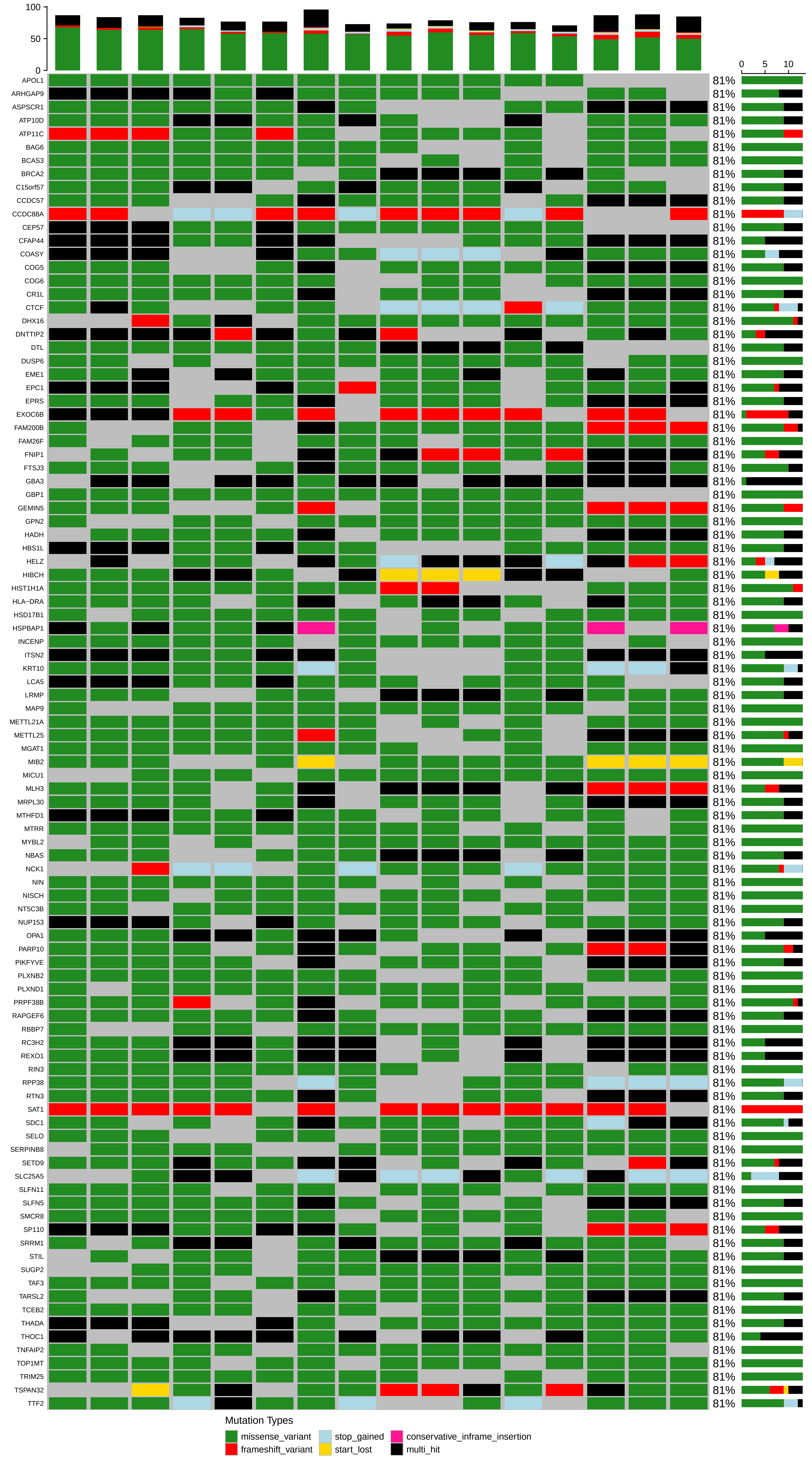
100
50
0
0
5
10
APOL1
ARHGAP9
ASPSCR1
ATP10D
ATP11C
BAG6
BCAS3
BRCA2
C15orf57
CCDC57
CCDC88A
CEP57
CFAP44
COASY
COG5
COG6
CR1L
CTCF
DHX16
DNTTIP2
DTL
DUSP6
EME1
EPC1
EPRS
EXOC6B
FAM200B
FAM26F
FNIP1
FTSJ3
GBA3
GBP1
GEMIN5
GPN2
HADH
HBS1L
HELZ
HIBCH
HIST1H1A
HLA−DRA
HSD17B1
HSPBAP1
INCENP
ITSN2
KRT10
LCA5
LRMP
MAP9
METTL21A
METTL25
MGAT1
MIB2
MICU1
MLH3
MRPL30
MTHFD1
MTRR
MYBL2
NBAS
NCK1
NIN
NISCH
NT5C3B
NUP153
OPA1
PARP10
PIKFYVE
PLXNB2
PLXND1
PRPF38B
RAPGEF6
RBBP7
RC3H2
REXO1
RIN3
RPP38
RTN3
SAT1
SDC1
SELO
SERPINB8
SETD9
SLC25A5
SLFN11
SLFN5
SMCR8
SP110
SRRM1
STIL
SUGP2
TAF3
TARSL2
TCEB2
THADA
THOC1
TNFAIP2
TOP1MT
TRIM25
TSPAN32
TTF2
81%
Mutation Types
missense_variant
frameshift_variant
stop_gained
start_lost
conservative_inframe_insertion
multi_hit

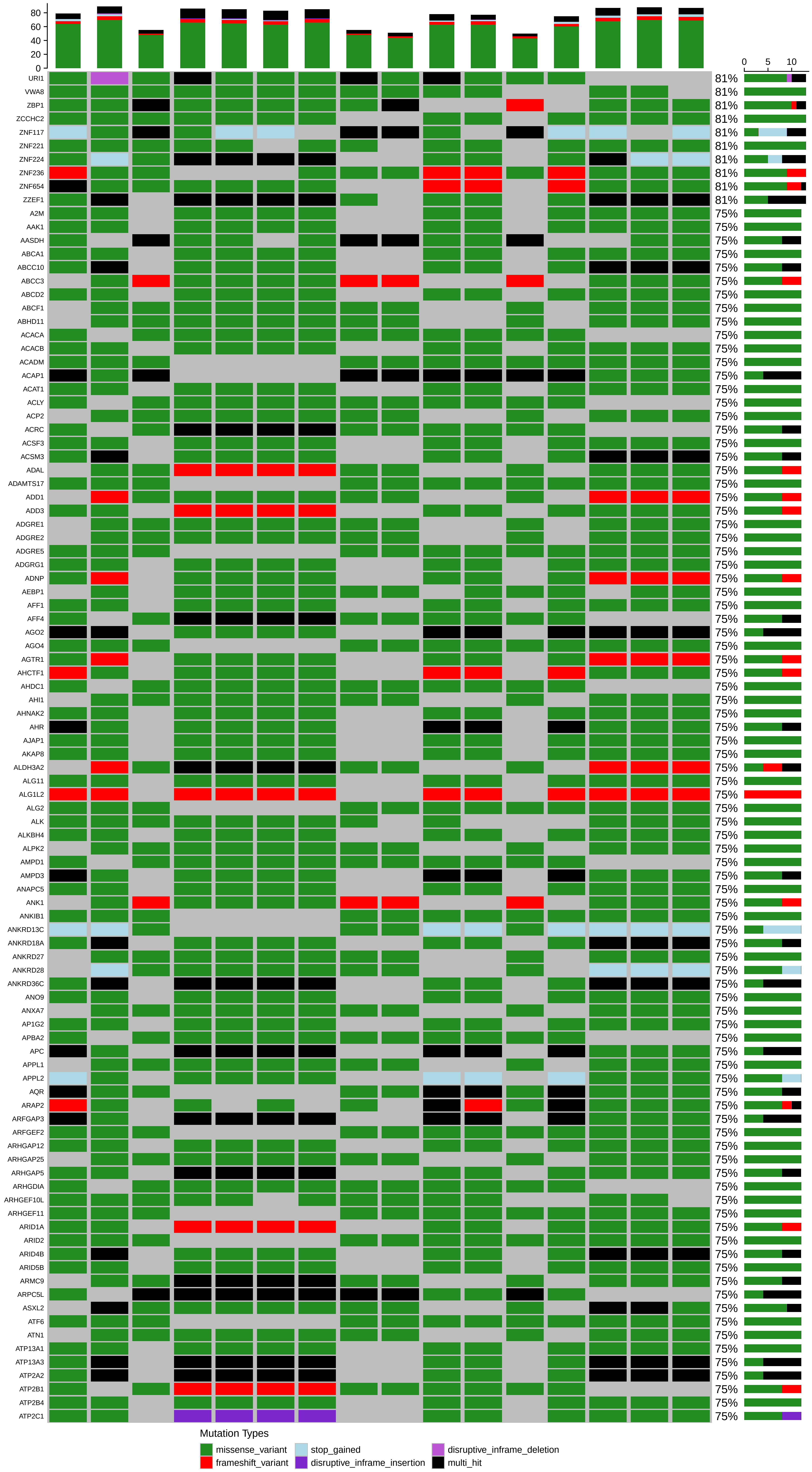

80
60
40
20
0
0
5
10
URI1
VWA8
ZBP1
ZCCHC2
ZNF117
ZNF221
ZNF224
ZNF236
ZNF654
ZZEF1
A2M
AAK1
AASDH
ABCA1
ABCC10
ABCC3
ABCD2
ABCF1
ABHD11
ACACA
ACACB
ACADM
ACAP1
ACAT1
ACLY
ACP2
ACRC
ACSF3
ACSM3
ADAL
ADAMTS17
ADD1
ADD3
ADGRE1
ADGRE2
ADGRE5
ADGRG1
ADNP
AEBP1
AFF1
AFF4
AGO2
AGO4
AGTR1
AHCTF1
AHDC1
AHI1
AHNAK2
AHR
AJAP1
AKAP8
ALDH3A2
ALG11
ALG1L2
ALG2
ALK
ALKBH4
ALPK2
AMPD1
AMPD3
ANAPC5
ANK1
ANKIB1
ANKRD13C
ANKRD18A
ANKRD27
ANKRD28
ANKRD36C
ANO9
ANXA7
AP1G2
APBA2
APC
APPL1
APPL2
AQR
ARAP2
ARFGAP3
ARFGEF2
ARHGAP12
ARHGAP25
ARHGAP5
ARHGDIA
ARHGEF10L
ARHGEF11
ARID1A
ARID2
ARID4B
ARID5B
ARMC9
ARPC5L
ASXL2
ATF6
ATN1
ATP13A1
ATP13A3
ATP2A2
ATP2B1
ATP2B4
ATP2C1
81%
81%
81%
81%
81%
81%
81%
81%
81%
81%
75%
Mutation Types
missense_variant
frameshift_variant
stop_gained
disruptive_inframe_insertion
disruptive_inframe_deletion
multi_hit

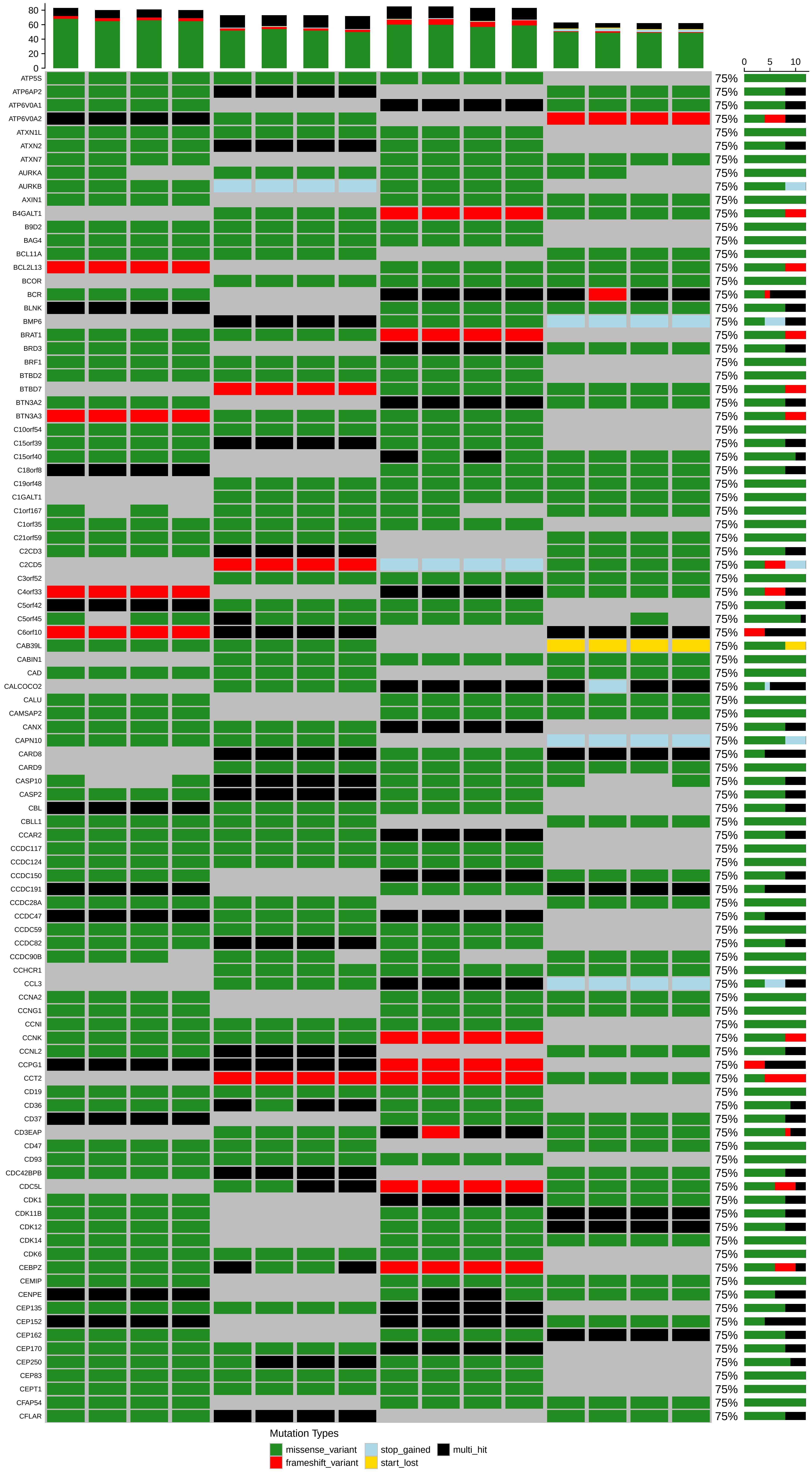
0
20
40
60
80
0
5
10
ATP5S
ATP6AP2
ATP6V0A1
ATP6V0A2
ATXN1L
ATXN2
ATXN7
AURKA
AURKB
AXIN1
B4GALT1
B9D2
BAG4
BCL11A
BCL2L13
BCOR
BCR
BLNK
BMP6
BRAT1
BRD3
BRF1
BTBD2
BTBD7
BTN3A2
BTN3A3
C10orf54
C15orf39
C15orf40
C18orf8
C19orf48
C1GALT1
C1orf167
C1orf35
C21orf59
C2CD3
C2CD5
C3orf52
C4orf33
C5orf42
C5orf45
C6orf10
CAB39L
CABIN1
CAD
CALCOCO2
CALU
CAMSAP2
CANX
CAPN10
CARD8
CARD9
CASP10
CASP2
CBL
CBLL1
CCAR2
CCDC117
CCDC124
CCDC150
CCDC191
CCDC28A
CCDC47
CCDC59
CCDC82
CCDC90B
CCHCR1
CCL3
CCNA2
CCNG1
CCNI
CCNK
CCNL2
CCPG1
CCT2
CD19
CD36
CD37
CD3EAP
CD47
CD93
CDC42BPB
CDC5L
CDK1
CDK11B
CDK12
CDK14
CDK6
CEBPZ
CEMIP
CENPE
CEP135
CEP152
CEP162
CEP170
CEP250
CEP83
CEPT1
CFAP54
CFLAR
75%
Mutation Types
missense_variant
frameshift_variant
stop_gained
start_lost
multi_hit

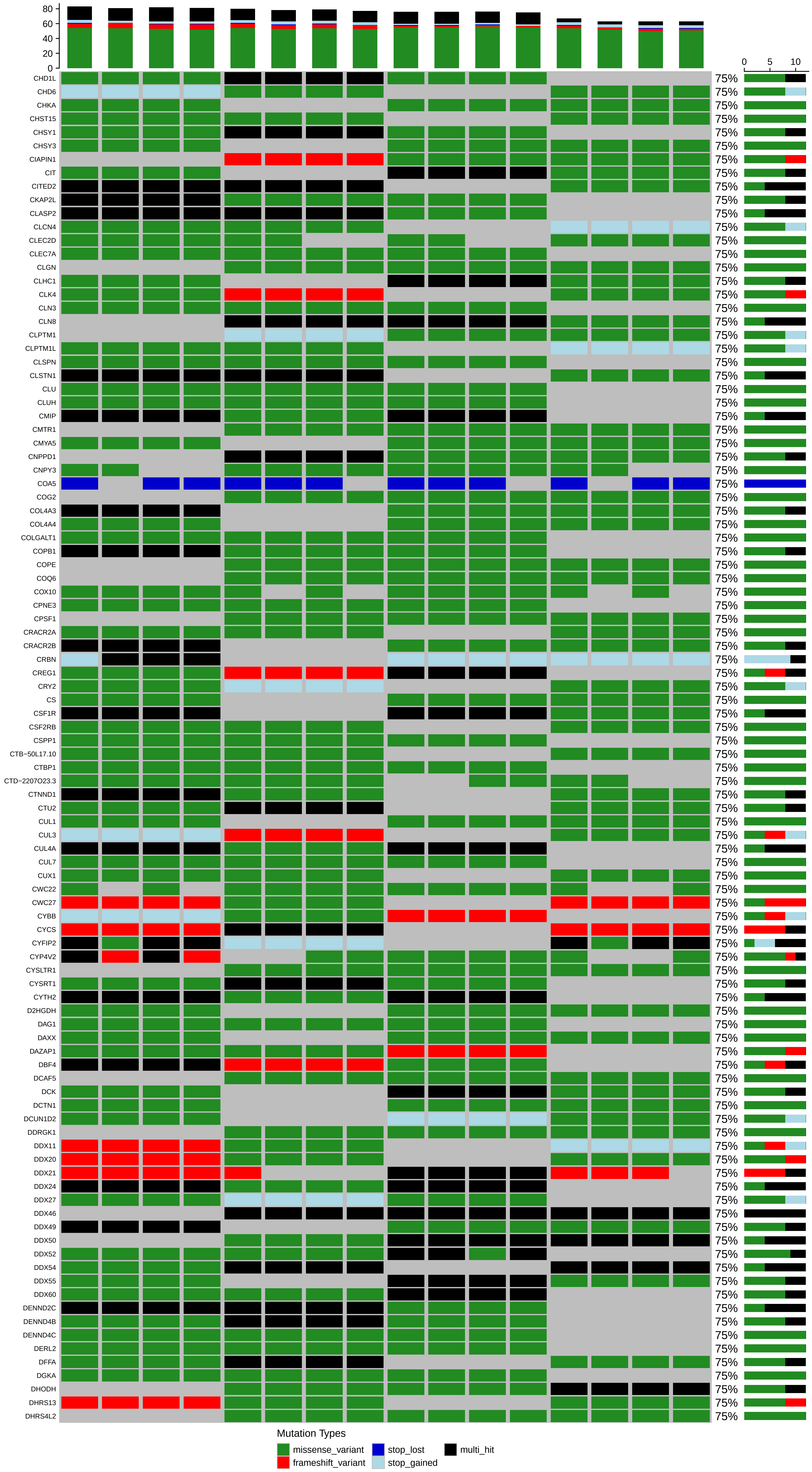

80
60
40
20
0
0
5
10
CHD1L
CHD6
CHKA
CHST15
CHSY1
CHSY3
CIAPIN1
CIT
CITED2
CKAP2L
CLASP2
CLCN4
CLEC2D
CLEC7A
CLGN
CLHC1
CLK4
CLN3
CLN8
CLPTM1
CLPTM1L
CLSPN
CLSTN1
CLU
CLUH
CMIP
CMTR1
CMYA5
CNPPD1
CNPY3
COA5
COG2
COL4A3
COL4A4
COLGALT1
COPB1
COPE
COQ6
COX10
CPNE3
CPSF1
CRACR2A
CRACR2B
CRBN
CREG1
CRY2
CS
CSF1R
CSF2RB
CSPP1
CTB−50L17.10
CTBP1
CTD−2207O23.3
CTNND1
CTU2
CUL1
CUL3
CUL4A
CUL7
CUX1
CWC22
CWC27
CYBB
CYCS
CYFIP2
CYP4V2
CYSLTR1
CYSRT1
CYTH2
D2HGDH
DAG1
DAXX
DAZAP1
DBF4
DCAF5
DCK
DCTN1
DCUN1D2
DDRGK1
DDX11
DDX20
DDX21
DDX24
DDX27
DDX46
DDX49
DDX50
DDX52
DDX54
DDX55
DDX60
DENND2C
DENND4B
DENND4C
DERL2
DFFA
DGKA
DHODH
DHRS13
DHRS4L2
75%
Mutation Types
missense_variant
frameshift_variant
stop_lost
stop_gained
multi_hit

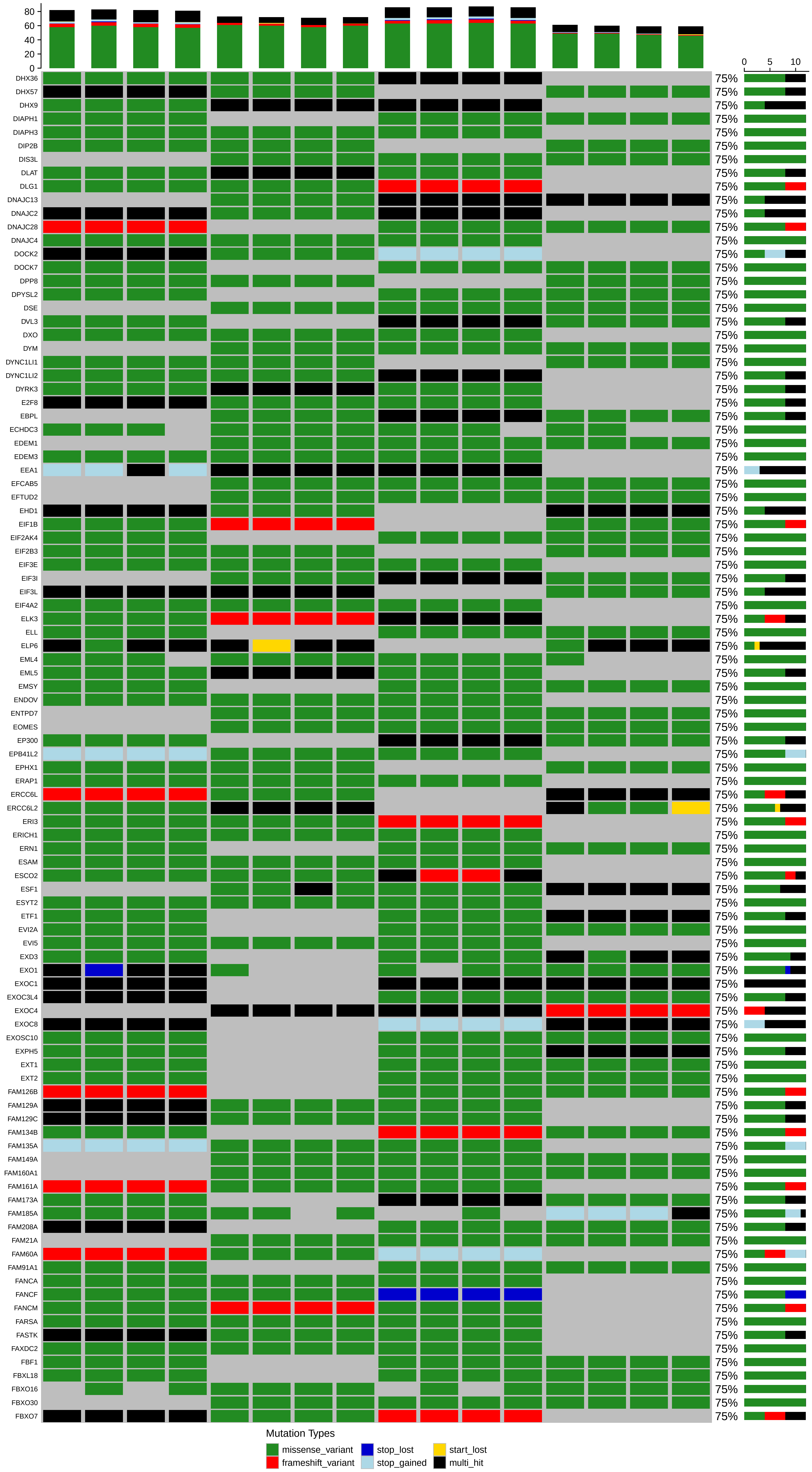
80
60
40
20
0
0
5
10
DHX36
DHX57
DHX9
DIAPH1
DIAPH3
DIP2B
DIS3L
DLAT
DLG1
DNAJC13
DNAJC2
DNAJC28
DNAJC4
DOCK2
DOCK7
DPP8
DPYSL2
DSE
DVL3
DXO
DYM
DYNC1LI1
DYNC1LI2
DYRK3
E2F8
EBPL
ECHDC3
EDEM1
EDEM3
EEA1
EFCAB5
EFTUD2
EHD1
EIF1B
EIF2AK4
EIF2B3
EIF3E
EIF3I
EIF3L
EIF4A2
ELK3
ELL
ELP6
EML4
EML5
EMSY
ENDOV
ENTPD7
EOMES
EP300
EPB41L2
EPHX1
ERAP1
ERCC6L
ERCC6L2
ERI3
ERICH1
ERN1
ESAM
ESCO2
ESF1
ESYT2
ETF1
EVI2A
EVI5
EXD3
EXO1
EXOC1
EXOC3L4
EXOC4
EXOC8
EXOSC10
EXPH5
EXT1
EXT2
FAM126B
FAM129A
FAM129C
FAM134B
FAM135A
FAM149A
FAM160A1
FAM161A
FAM173A
FAM185A
FAM208A
FAM21A
FAM60A
FAM91A1
FANCA
FANCF
FANCM
FARSA
FASTK
FAXDC2
FBF1
FBXL18
FBXO16
FBXO30
FBXO7
75%
Mutation Types
missense_variant
frameshift_variant
stop_lost
stop_gained
start_lost
multi_hit

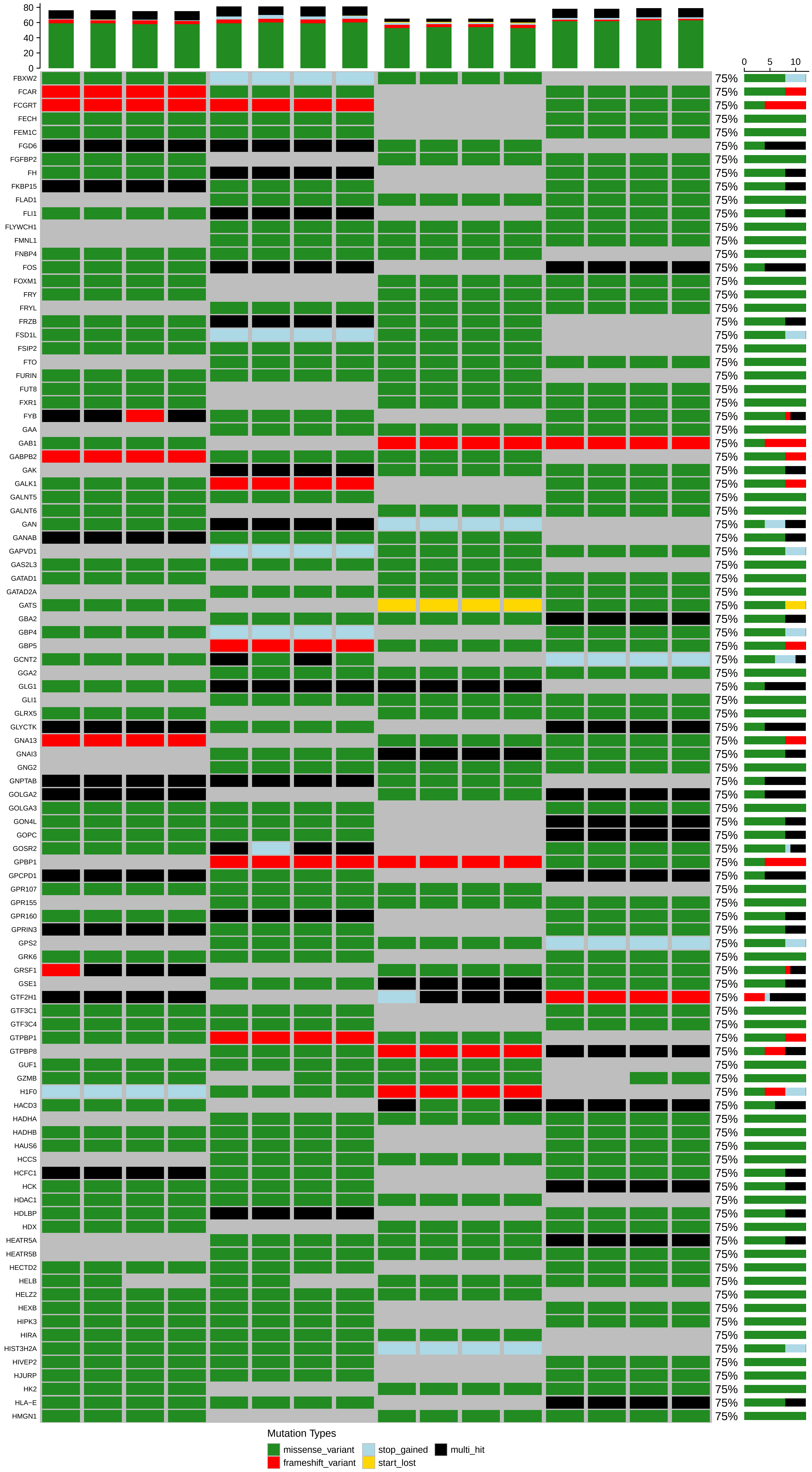

80
60
40
20
0
0
5
10
FBXW2
FCAR
FCGRT
FECH
FEM1C
FGD6
FGFBP2
FH
FKBP15
FLAD1
FLI1
FLYWCH1
FMNL1
FNBP4
FOS
FOXM1
FRY
FRYL
FRZB
FSD1L
FSIP2
FTO
FURIN
FUT8
FXR1
FYB
GAA
GAB1
GABPB2
GAK
GALK1
GALNT5
GALNT6
GAN
GANAB
GAPVD1
GAS2L3
GATAD1
GATAD2A
GATS
GBA2
GBP4
GBP5
GCNT2
GGA2
GLG1
GLI1
GLRX5
GLYCTK
GNA13
GNAI3
GNG2
GNPTAB
GOLGA2
GOLGA3
GON4L
GOPC
GOSR2
GPBP1
GPCPD1
GPR107
GPR155
GPR160
GPRIN3
GPS2
GRK6
GRSF1
GSE1
GTF2H1
GTF3C1
GTF3C4
GTPBP1
GTPBP8
GUF1
GZMB
H1F0
HACD3
HADHA
HADHB
HAUS6
HCCS
HCFC1
HCK
HDAC1
HDLBP
HDX
HEATR5A
HEATR5B
HECTD2
HELB
HELZ2
HEXB
HIPK3
HIRA
HIST3H2A
HIVEP2
HJURP
HK2
HLA−E
HMGN1
75%
Mutation Types
missense_variant
frameshift_variant
stop_gained
start_lost
multi_hit

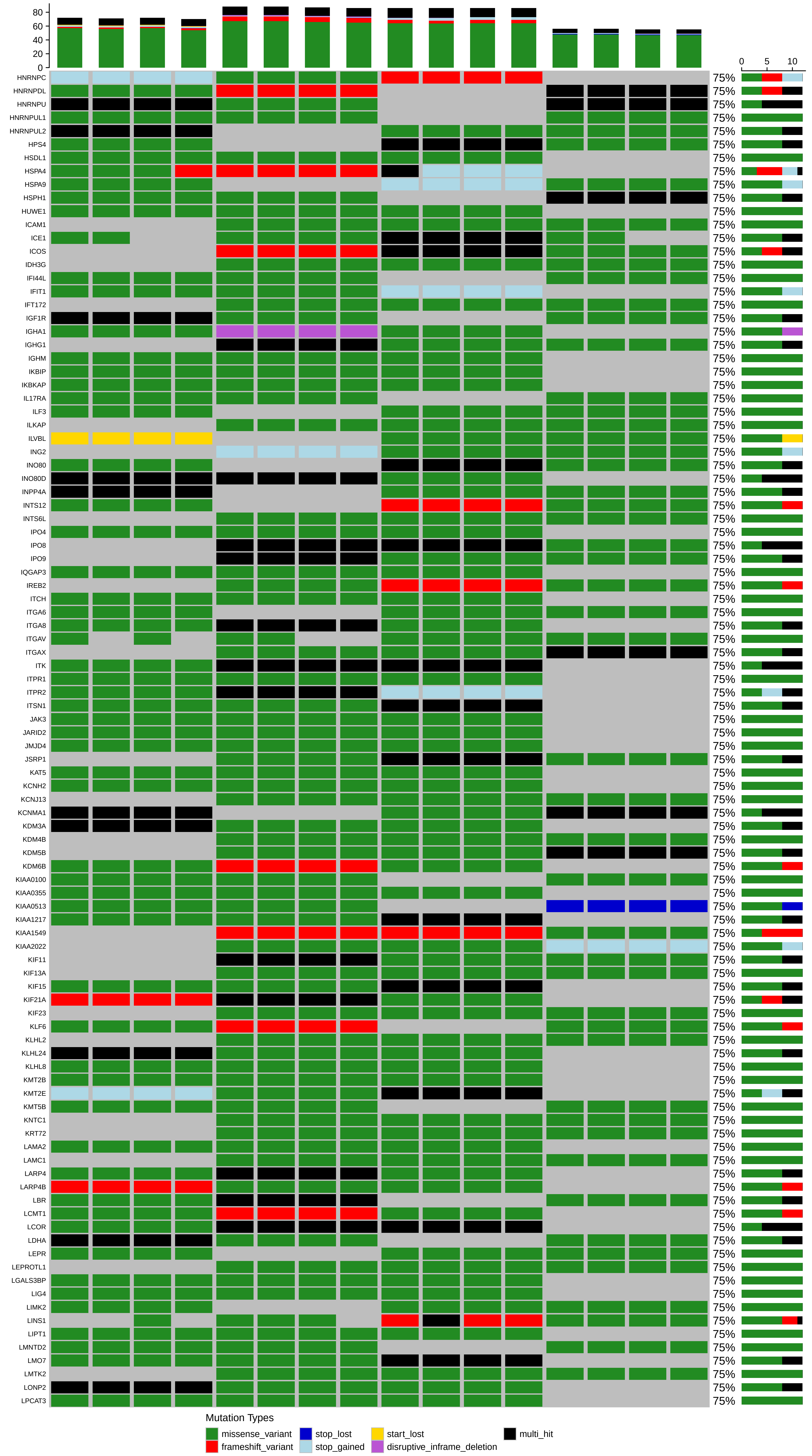
80
60
40
20
0
0
5
10
HNRNPC
HNRNPDL
HNRNPU
HNRNPUL1
HNRNPUL2
HPS4
HSDL1
HSPA4
HSPA9
HSPH1
HUWE1
ICAM1
ICE1
ICOS
IDH3G
IFI44L
IFIT1
IFT172
IGF1R
IGHA1
IGHG1
IGHM
IKBIP
IKBKAP
IL17RA
ILF3
ILKAP
ILVBL
ING2
INO80
INO80D
INPP4A
INTS12
INTS6L
IPO4
IPO8
IPO9
IQGAP3
IREB2
ITCH
ITGA6
ITGA8
ITGAV
ITGAX
ITK
ITPR1
ITPR2
ITSN1
JAK3
JARID2
JMJD4
JSRP1
KAT5
KCNH2
KCNJ13
KCNMA1
KDM3A
KDM4B
KDM5B
KDM6B
KIAA0100
KIAA0355
KIAA0513
KIAA1217
KIAA1549
KIAA2022
KIF11
KIF13A
KIF15
KIF21A
KIF23
KLF6
KLHL2
KLHL24
KLHL8
KMT2B
KMT2E
KMT5B
KNTC1
KRT72
LAMA2
LAMC1
LARP4
LARP4B
LBR
LCMT1
LCOR
LDHA
LEPR
LEPROTL1
LGALS3BP
LIG4
LIMK2
LINS1
LIPT1
LMNTD2
LMO7
LMTK2
LONP2
LPCAT3
75%
Mutation Types
missense_variant
frameshift_variant
stop_lost
stop_gained
start_lost
disruptive_inframe_deletion
multi_hit

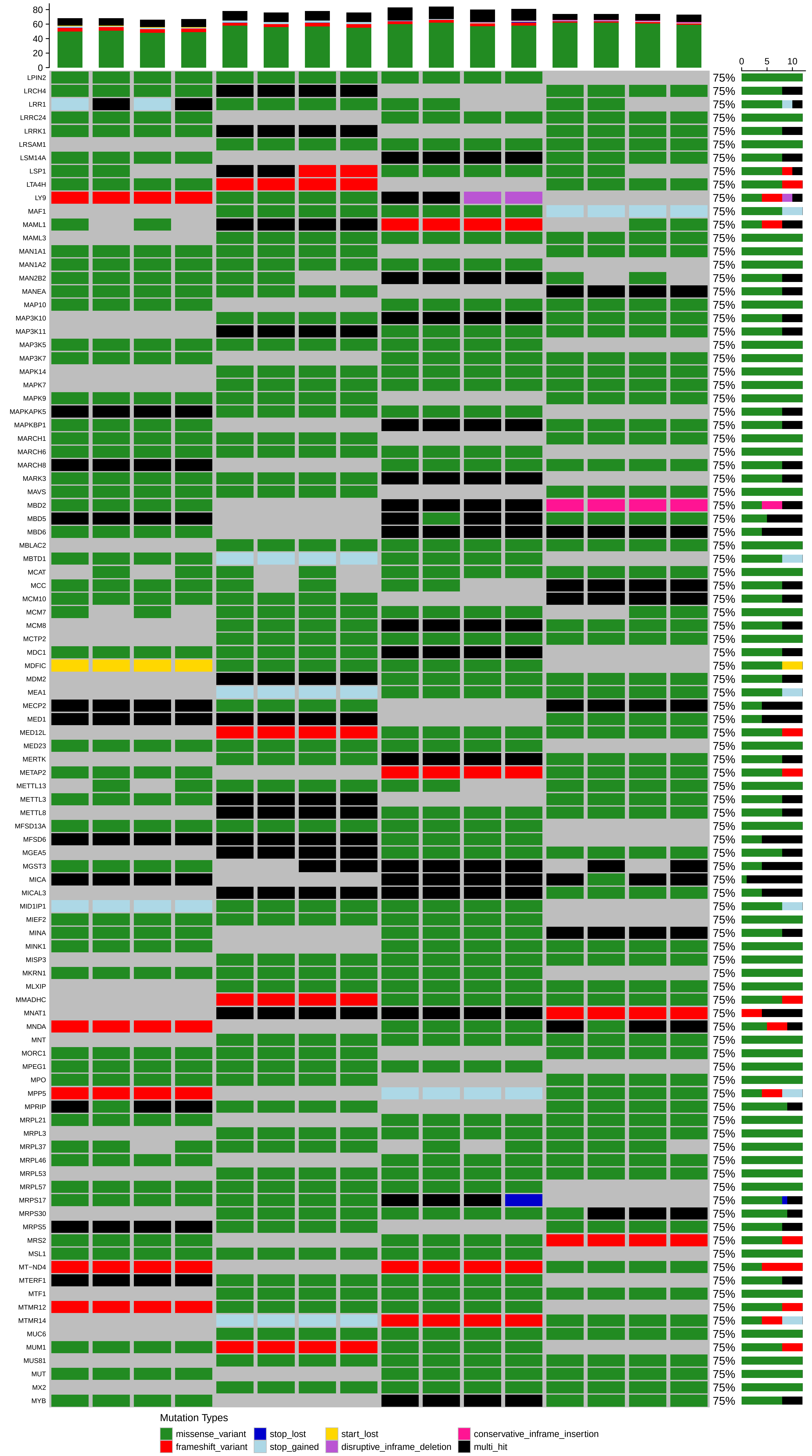

LPIN2
LRCH4
LRR1
LRRC24
LRRK1
LRSAM1
LSM14A
LSP1
LTA4H
LY9
MAF1
MAML1
MAML3
MAN1A1
MAN1A2
MAN2B2
MANEA
MAP10
MAP3K10
MAP3K11
MAP3K5
MAP3K7
MAPK14
MAPK7
MAPK9
MAPKAPK5
MAPKBP1
MARCH1
MARCH6
MARCH8
MARK3
MAVS
MBD2
MBD5
MBD6
MBLAC2
MBTD1
MCAT
MCC
MCM10
MCM7
MCM8
MCTP2
MDC1
MDFIC
MDM2
MEA1
MECP2
MED1
MED12L
MED23
MERTK
METAP2
METTL13
METTL3
METTL8
MFSD13A
MFSD6
MGEA5
MGST3
MICA
MICAL3
MID1IP1
MIEF2
MINA
MINK1
MISP3
MKRN1
MLXIP
MMADHC
MNAT1
MNDA
MNT
MORC1
MPEG1
MPO
MPP5
MPRIP
MRPL21
MRPL3
MRPL37
MRPL46
MRPL53
MRPL57
MRPS17
MRPS30
MRPS5
MRS2
MSL1
MT−ND4
MTERF1
MTF1
MTMR12
MTMR14
MUC6
MUM1
MUS81
MUT
MX2
MYB
75%
0
5
10
80
60
40
20
0
Mutation Types
missense_variant
frameshift_variant
stop_lost
stop_gained
start_lost
disruptive_inframe_deletion
conservative_inframe_insertion
multi_hit

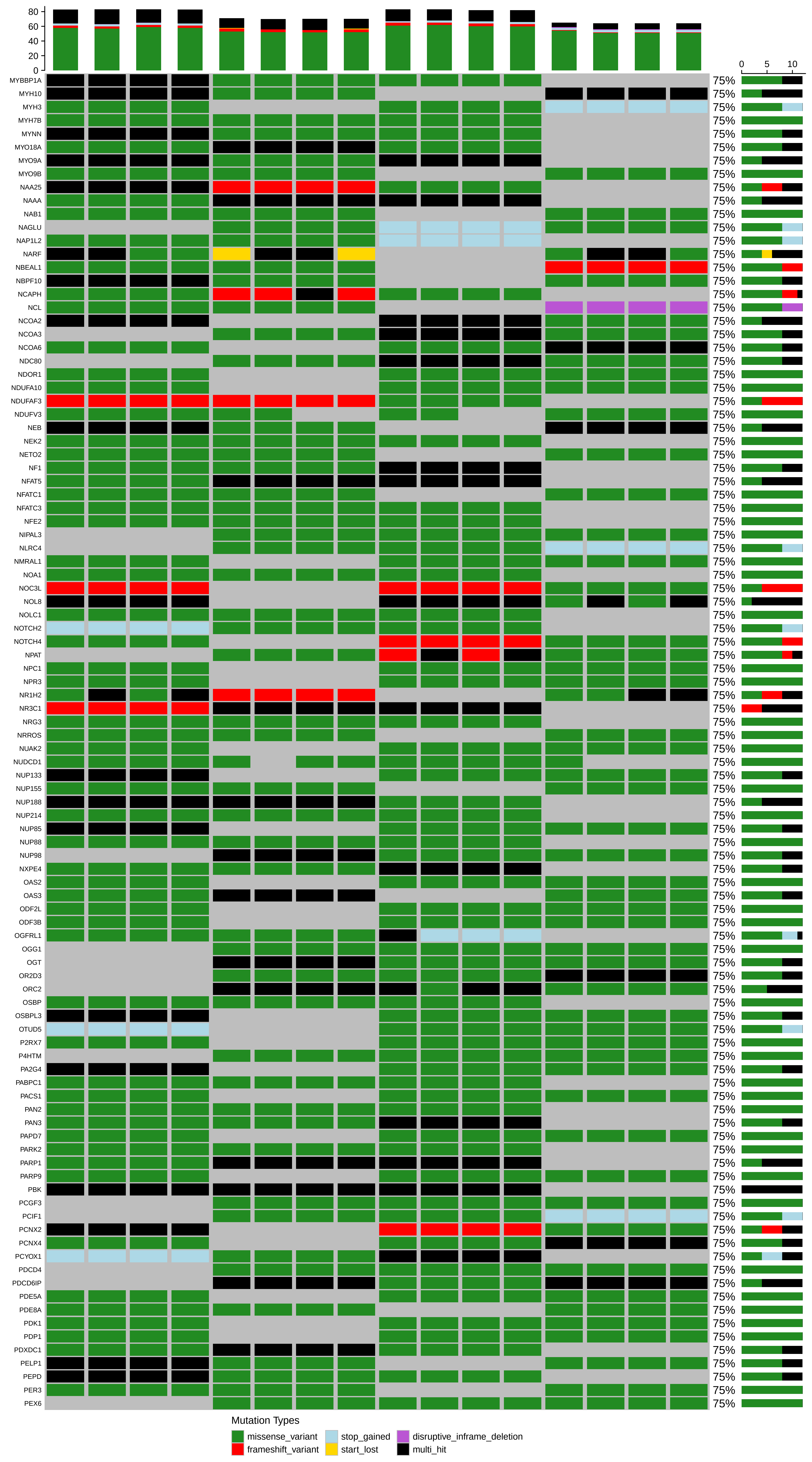

80
60
40
20
0
0
5
10
MYBBP1A
MYH10
MYH3
MYH7B
MYNN
MYO18A
MYO9A
MYO9B
NAA25
NAAA
NAB1
NAGLU
NAP1L2
NARF
NBEAL1
NBPF10
NCAPH
NCL
NCOA2
NCOA3
NCOA6
NDC80
NDOR1
NDUFA10
NDUFAF3
NDUFV3
NEB
NEK2
NETO2
NF1
NFAT5
NFATC1
NFATC3
NFE2
NIPAL3
NLRC4
NMRAL1
NOA1
NOC3L
NOL8
NOLC1
NOTCH2
NOTCH4
NPAT
NPC1
NPR3
NR1H2
NR3C1
NRG3
NRROS
NUAK2
NUDCD1
NUP133
NUP155
NUP188
NUP214
NUP85
NUP88
NUP98
NXPE4
OAS2
OAS3
ODF2L
ODF3B
OGFRL1
OGG1
OGT
OR2D3
ORC2
OSBP
OSBPL3
OTUD5
P2RX7
P4HTM
PA2G4
PABPC1
PACS1
PAN2
PAN3
PAPD7
PARK2
PARP1
PARP9
PBK
PCGF3
PCIF1
PCNX2
PCNX4
PCYOX1
PDCD4
PDCD6IP
PDE5A
PDE8A
PDK1
PDP1
PDXDC1
PELP1
PEPD
PER3
PEX6
75% (on each of the 101 gene rows)
Mutation Types
missense_variant
frameshift_variant
stop_gained
start_lost
disruptive_inframe_deletion
multi_hit

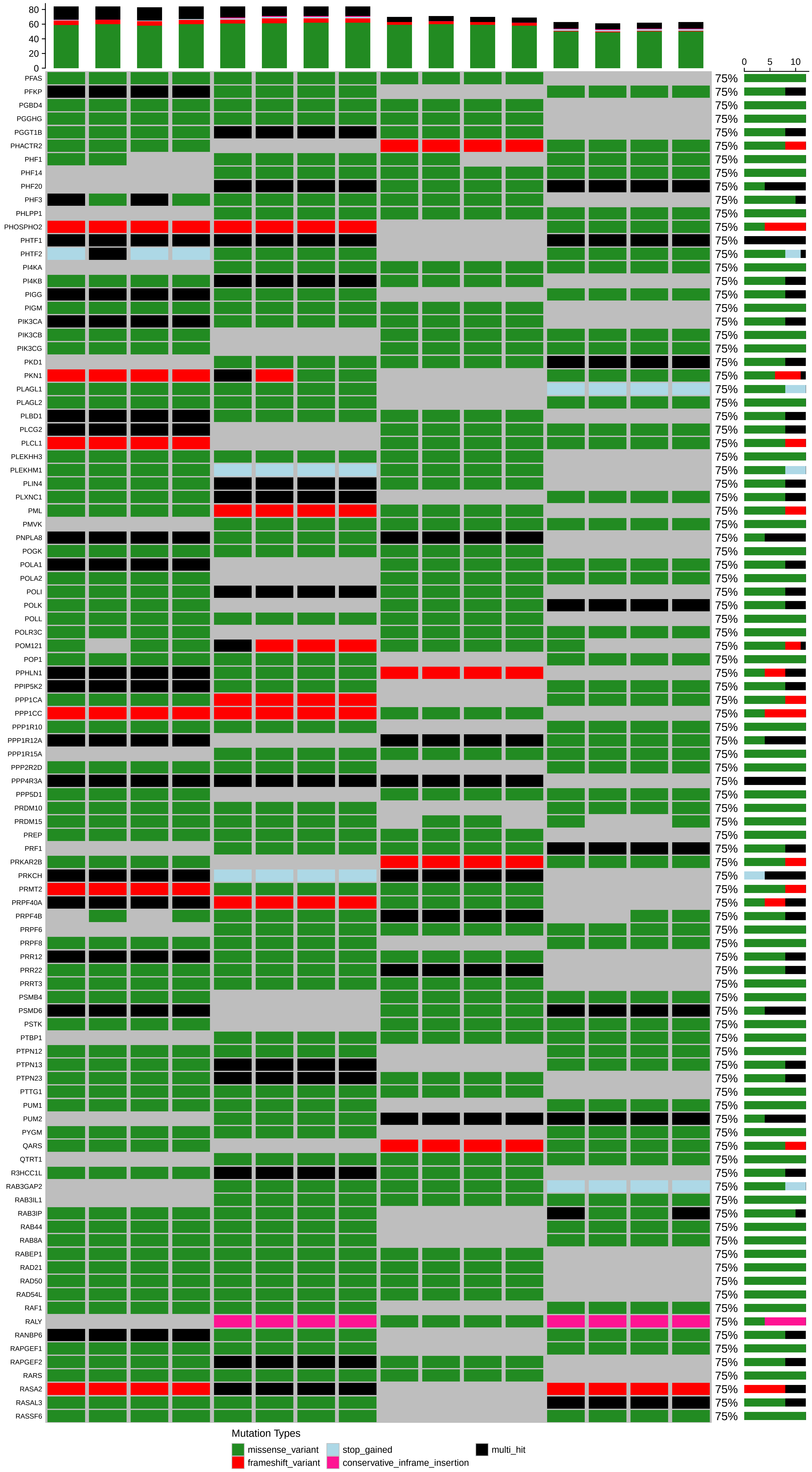

80
60
40
20
0
0
5
10
PFAS
PFKP
PGBD4
PGGHG
PGGT1B
PHACTR2
PHF1
PHF14
PHF20
PHF3
PHLPP1
PHOSPHO2
PHTF1
PHTF2
PI4KA
PI4KB
PIGG
PIGM
PIK3CA
PIK3CB
PIK3CG
PKD1
PKN1
PLAGL1
PLAGL2
PLBD1
PLCG2
PLCL1
PLEKHH3
PLEKHM1
PLIN4
PLXNC1
PML
PMVK
PNPLA8
POGK
POLA1
POLA2
POLI
POLK
POLL
POLR3C
POM121
POP1
PPHLN1
PPIP5K2
PPP1CA
PPP1CC
PPP1R10
PPP1R12A
PPP1R15A
PPP2R2D
PPP4R3A
PPP5D1
PRDM10
PRDM15
PREP
PRF1
PRKAR2B
PRKCH
PRMT2
PRPF40A
PRPF4B
PRPF6
PRPF8
PRR12
PRR22
PRRT3
PSMB4
PSMD6
PSTK
PTBP1
PTPN12
PTPN13
PTPN23
PTTG1
PUM1
PUM2
PYGM
QARS
QTRT1
R3HCC1L
RAB3GAP2
RAB3IL1
RAB3IP
RAB44
RAB8A
RABEP1
RAD21
RAD50
RAD54L
RAF1
RALY
RANBP6
RAPGEF1
RAPGEF2
RARS
RASA2
RASAL3
RASSF6
75%
Mutation Types
missense_variant
frameshift_variant
stop_gained
conservative_inframe_insertion
multi_hit

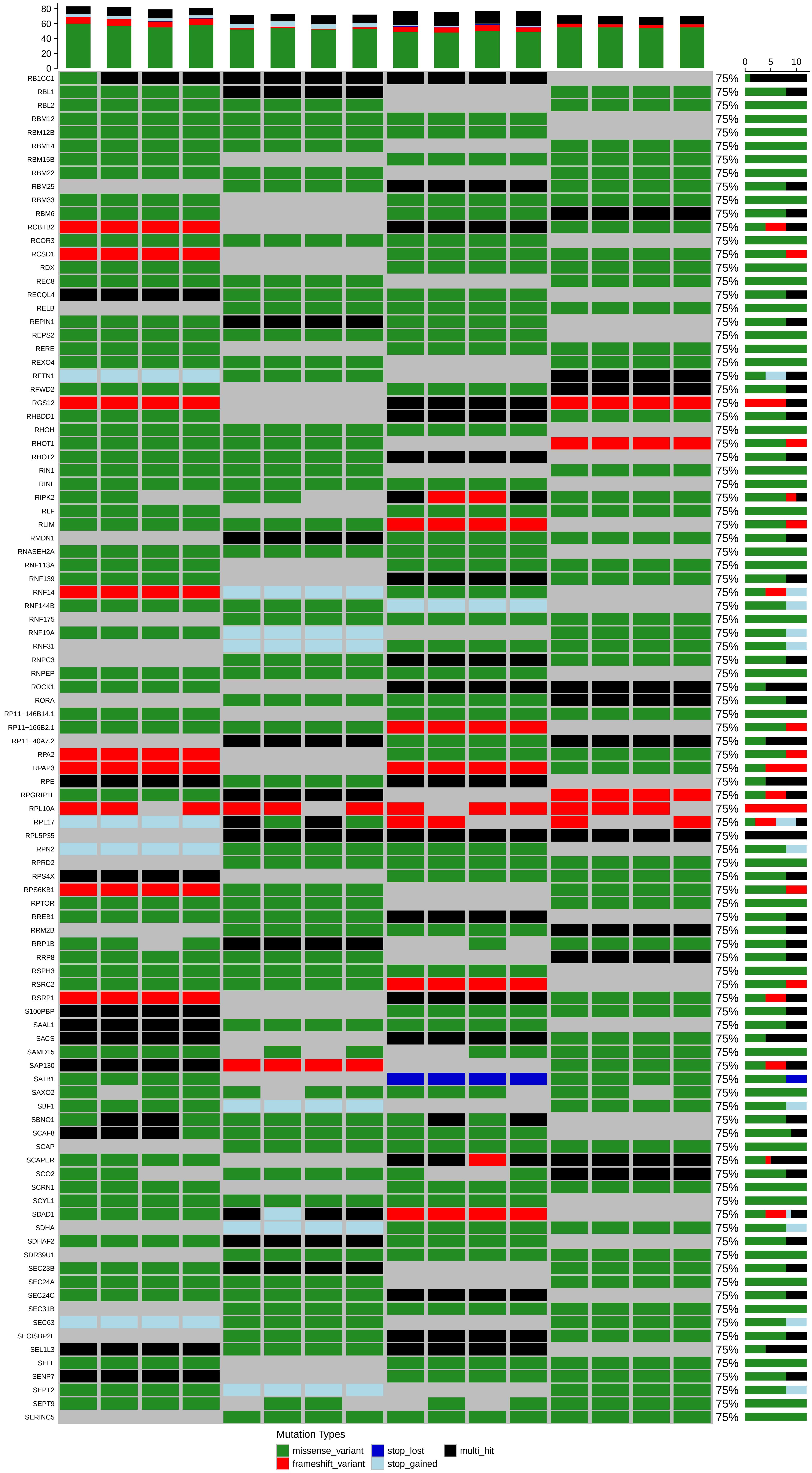
80
60
40
20
0
0
5
10
RB1CC1
RBL1
RBL2
RBM12
RBM12B
RBM14
RBM15B
RBM22
RBM25
RBM33
RBM6
RCBTB2
RCOR3
RCSD1
RDX
REC8
RECQL4
RELB
REPIN1
REPS2
RERE
REXO4
RFTN1
RFWD2
RGS12
RHBDD1
RHOH
RHOT1
RHOT2
RIN1
RINL
RIPK2
RLF
RLIM
RMDN1
RNASEH2A
RNF113A
RNF139
RNF14
RNF144B
RNF175
RNF19A
RNF31
RNPC3
RNPEP
ROCK1
RORA
RP11−146B14.1
RP11−166B2.1
RP11−40A7.2
RPA2
RPAP3
RPE
RPGRIP1L
RPL10A
RPL17
RPL5P35
RPN2
RPRD2
RPS4X
RPS6KB1
RPTOR
RREB1
RRM2B
RRP1B
RRP8
RSPH3
RSRC2
RSRP1
S100PBP
SAAL1
SACS
SAMD15
SAP130
SATB1
SAXO2
SBF1
SBNO1
SCAF8
SCAP
SCAPER
SCO2
SCRN1
SCYL1
SDAD1
SDHA
SDHAF2
SDR39U1
SEC23B
SEC24A
SEC24C
SEC31B
SEC63
SECISBP2L
SEL1L3
SELL
SENP7
SEPT2
SEPT9
SERINC5
75%
Mutation Types
missense_variant
frameshift_variant
stop_lost
stop_gained
multi_hit

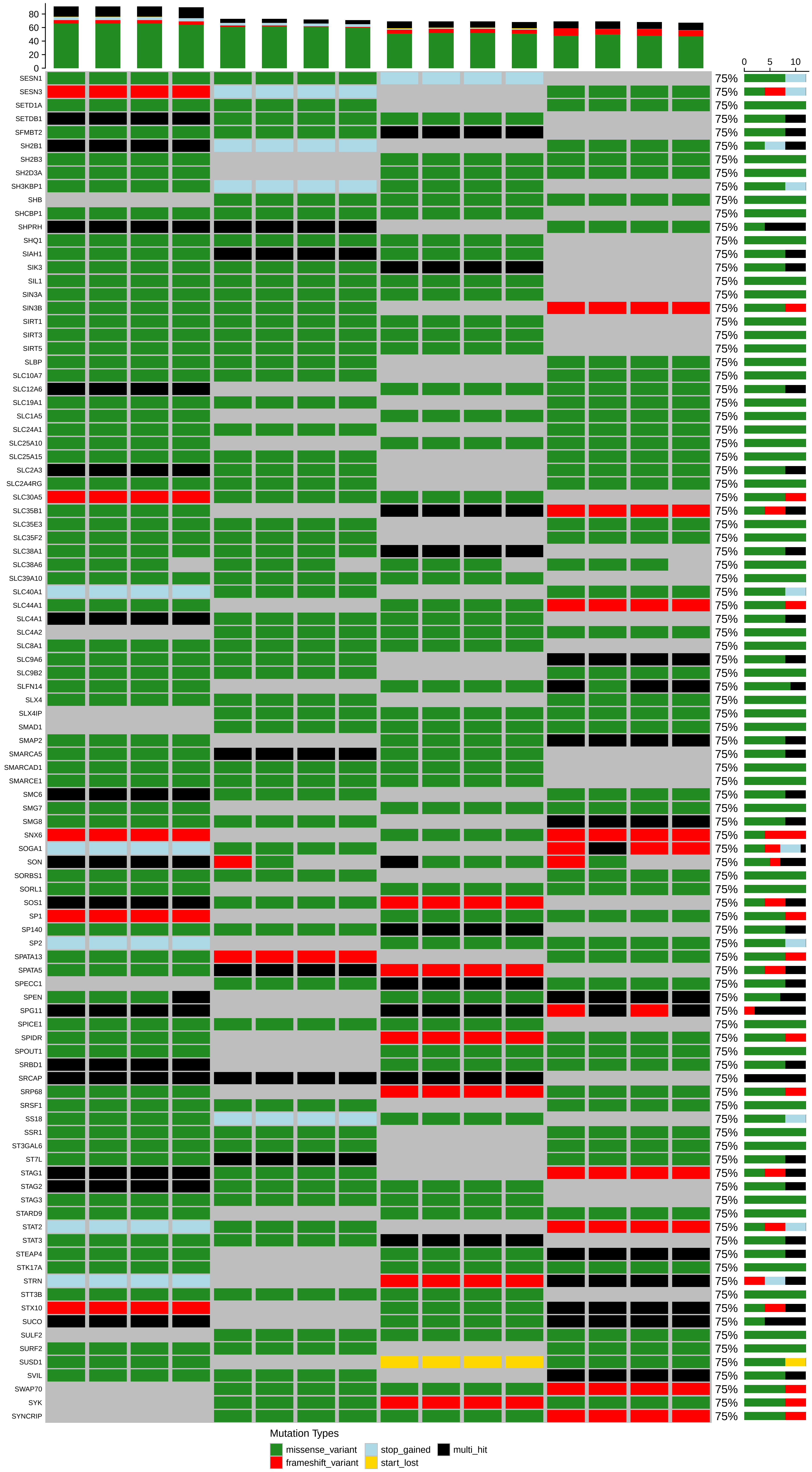

SESN1
SESN3
SETD1A
SETDB1
SFMBT2
SH2B1
SH2B3
SH2D3A
SH3KBP1
SHB
SHCBP1
SHPRH
SHQ1
SIAH1
SIK3
SIL1
SIN3A
SIN3B
SIRT1
SIRT3
SIRT5
SLBP
SLC10A7
SLC12A6
SLC19A1
SLC1A5
SLC24A1
SLC25A10
SLC25A15
SLC2A3
SLC2A4RG
SLC30A5
SLC35B1
SLC35E3
SLC35F2
SLC38A1
SLC38A6
SLC39A10
SLC40A1
SLC44A1
SLC4A1
SLC4A2
SLC8A1
SLC9A6
SLC9B2
SLFN14
SLX4
SLX4IP
SMAD1
SMAP2
SMARCA5
SMARCAD1
SMARCE1
SMC6
SMG7
SMG8
SNX6
SOGA1
SON
SORBS1
SORL1
SOS1
SP1
SP140
SP2
SPATA13
SPATA5
SPECC1
SPEN
SPG11
SPICE1
SPIDR
SPOUT1
SRBD1
SRCAP
SRP68
SRSF1
SS18
SSR1
ST3GAL6
ST7L
STAG1
STAG2
STAG3
STARD9
STAT2
STAT3
STEAP4
STK17A
STRN
STT3B
STX10
SUCO
SULF2
SURF2
SUSD1
SVIL
SWAP70
SYK
SYNCRIP
Mutation Types
missense_variant
frameshift_variant
stop_gained
start_lost
multi_hit

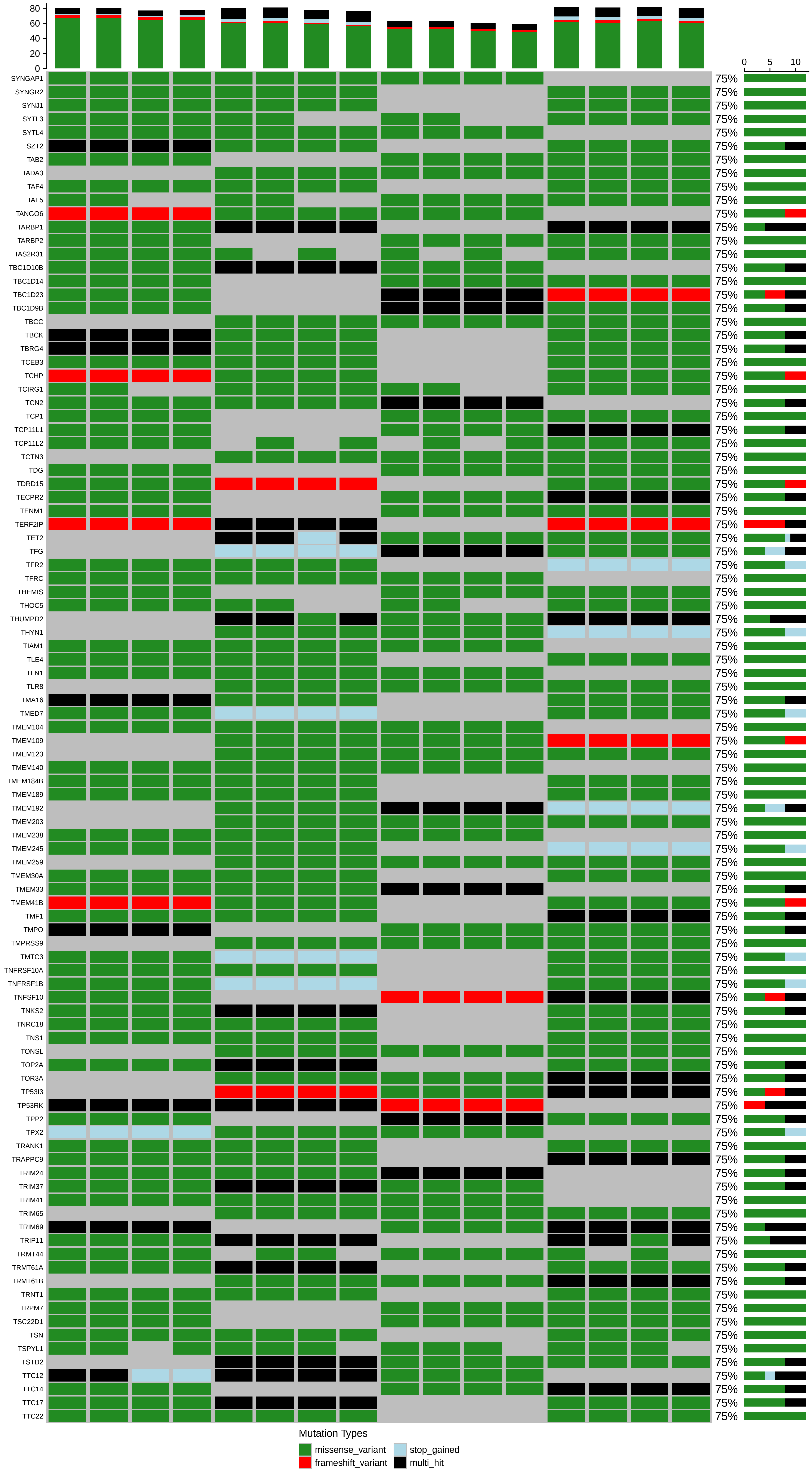

80
60
40
20
0
0
5
10
SYNGAP1
SYNGR2
SYNJ1
SYTL3
SYTL4
SZT2
TAB2
TADA3
TAF4
TAF5
TANGO6
TARBP1
TARBP2
TAS2R31
TBC1D10B
TBC1D14
TBC1D23
TBC1D9B
TBCC
TBCK
TBRG4
TCEB3
TCHP
TCIRG1
TCN2
TCP1
TCP11L1
TCP11L2
TCTN3
TDG
TDRD15
TECPR2
TENM1
TERF2IP
TET2
TFG
TFR2
TFRC
THEMIS
THOC5
THUMPD2
THYN1
TIAM1
TLE4
TLN1
TLR8
TMA16
TMED7
TMEM104
TMEM109
TMEM123
TMEM140
TMEM184B
TMEM189
TMEM192
TMEM203
TMEM238
TMEM245
TMEM259
TMEM30A
TMEM33
TMEM41B
TMF1
TMPO
TMPRSS9
TMTC3
TNFRSF10A
TNFRSF1B
TNFSF10
TNKS2
TNRC18
TNS1
TONSL
TOP2A
TOR3A
TP53I3
TP53RK
TPP2
TPX2
TRANK1
TRAPPC9
TRIM24
TRIM37
TRIM41
TRIM65
TRIM69
TRIP11
TRMT44
TRMT61A
TRMT61B
TRNT1
TRPM7
TSC22D1
TSN
TSPYL1
TSTD2
TTC12
TTC14
TTC17
TTC22
75%
Mutation Types
missense_variant
frameshift_variant
stop_gained
multi_hit

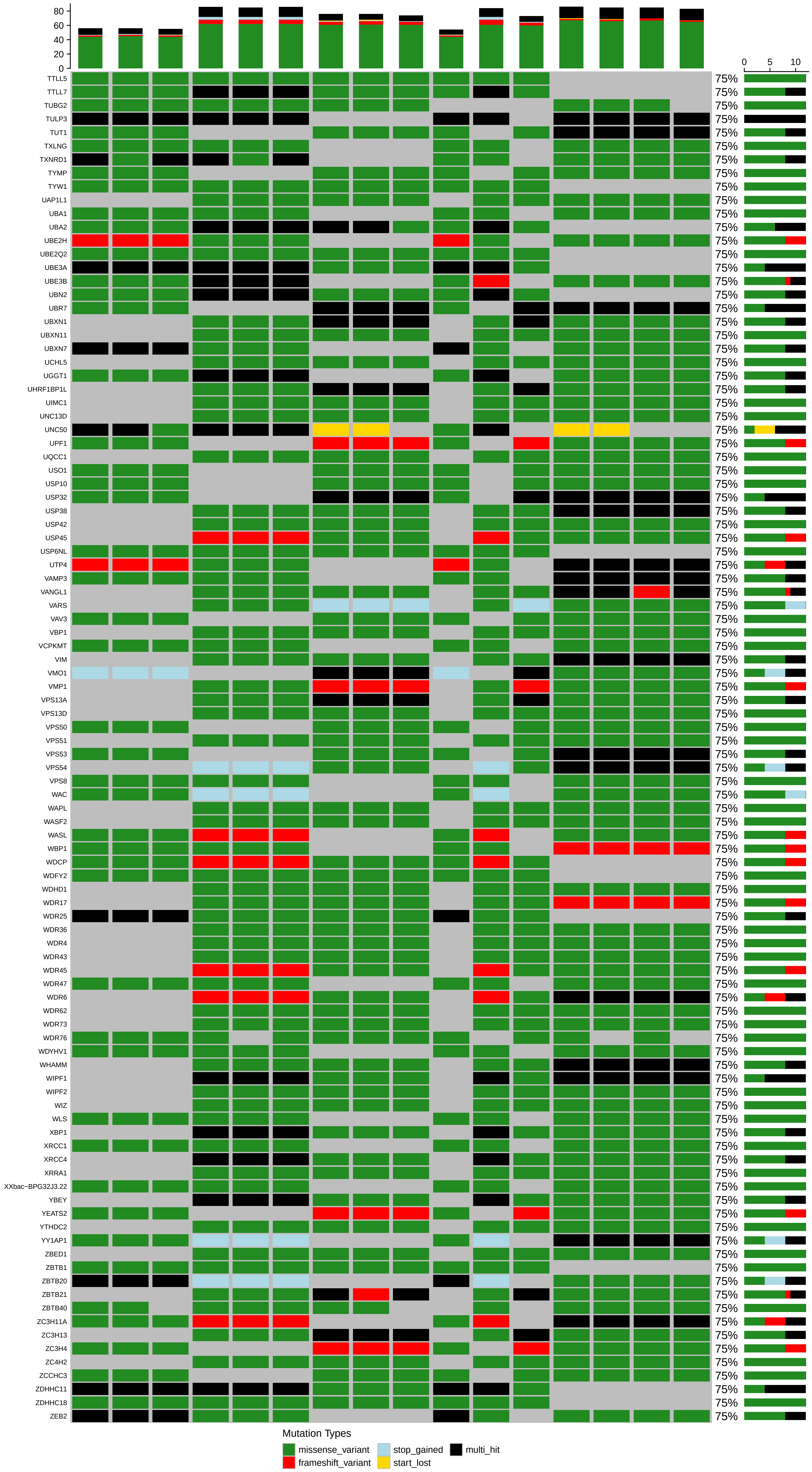
80
60
40
20
0
0
5
10
TTLL5
TTLL7
TUBG2
TULP3
TUT1
TXLNG
TXNRD1
TYMP
TYW1
UAP1L1
UBA1
UBA2
UBE2H
UBE2Q2
UBE3A
UBE3B
UBN2
UBR7
UBXN1
UBXN11
UBXN7
UCHL5
UGGT1
UHRF1BP1L
UIMC1
UNC13D
UNC50
UPF1
UQCC1
USO1
USP10
USP32
USP38
USP42
USP45
USP6NL
UTP4
VAMP3
VANGL1
VARS
VAV3
VBP1
VCPKMT
VIM
VMO1
VMP1
VPS13A
VPS13D
VPS50
VPS51
VPS53
VPS54
VPS8
WAC
WAPL
WASF2
WASL
WBP1
WDCP
WDFY2
WDHD1
WDR17
WDR25
WDR36
WDR4
WDR43
WDR45
WDR47
WDR6
WDR62
WDR73
WDR76
WDYHV1
WHAMM
WIPF1
WIPF2
WIZ
WLS
XBP1
XRCC1
XRCC4
XRRA1
XXbac−BPG32J3.22
YBEY
YEATS2
YTHDC2
YY1AP1
ZBED1
ZBTB1
ZBTB20
ZBTB21
ZBTB40
ZC3H11A
ZC3H13
ZC3H4
ZC4H2
ZCCHC3
ZDHHC11
ZDHHC18
ZEB2
75%
Mutation Types
missense_variant
frameshift_variant
stop_gained
start_lost
multi_hit

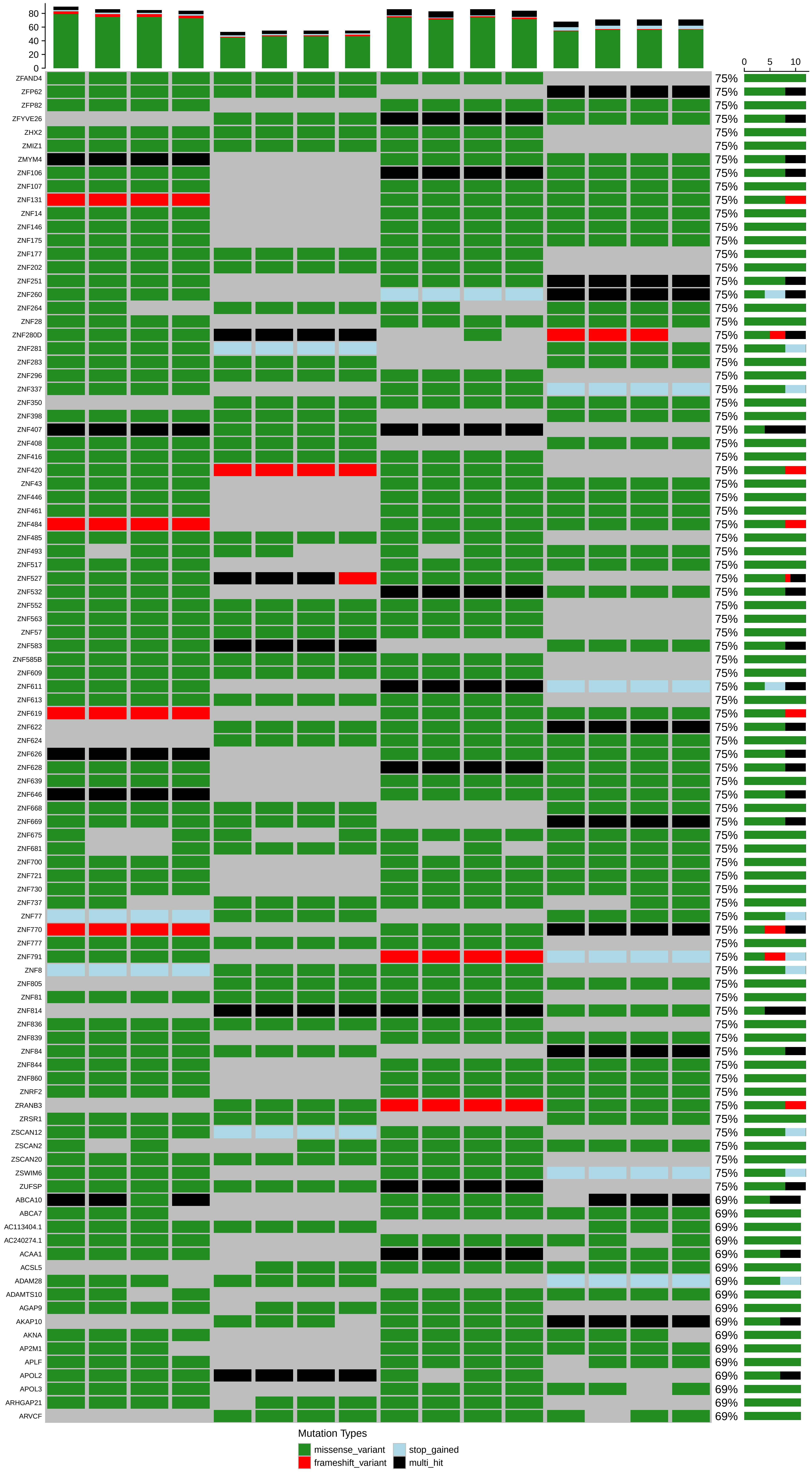
ZFAND4
ZFP62
ZFP82
ZFYVE26
ZHX2
ZMIZ1
ZMYM4
ZNF106
ZNF107
ZNF131
ZNF14
ZNF146
ZNF175
ZNF177
ZNF202
ZNF251
ZNF260
ZNF264
ZNF28
ZNF280D
ZNF281
ZNF283
ZNF296
ZNF337
ZNF350
ZNF398
ZNF407
ZNF408
ZNF416
ZNF420
ZNF43
ZNF446
ZNF461
ZNF484
ZNF485
ZNF493
ZNF517
ZNF527
ZNF532
ZNF552
ZNF563
ZNF57
ZNF583
ZNF585B
ZNF609
ZNF611
ZNF613
ZNF619
ZNF622
ZNF624
ZNF626
ZNF628
ZNF639
ZNF646
ZNF668
ZNF669
ZNF675
ZNF681
ZNF700
ZNF721
ZNF730
ZNF737
ZNF77
ZNF770
ZNF777
ZNF791
ZNF8
ZNF805
ZNF81
ZNF814
ZNF836
ZNF839
ZNF84
ZNF844
ZNF860
ZNRF2
ZRANB3
ZRSR1
ZSCAN12
ZSCAN2
ZSCAN20
ZSWIM6
ZUFSP
ABCA10
ABCA7
AC113404.1
AC240274.1
ACAA1
ACSL5
ADAM28
ADAMTS10
AGAP9
AKAP10
AKNA
AP2M1
APLF
APOL2
APOL3
ARHGAP21
ARVCF
Mutation Types
missense_variant
stop_gained
frameshift_variant
multi_hit

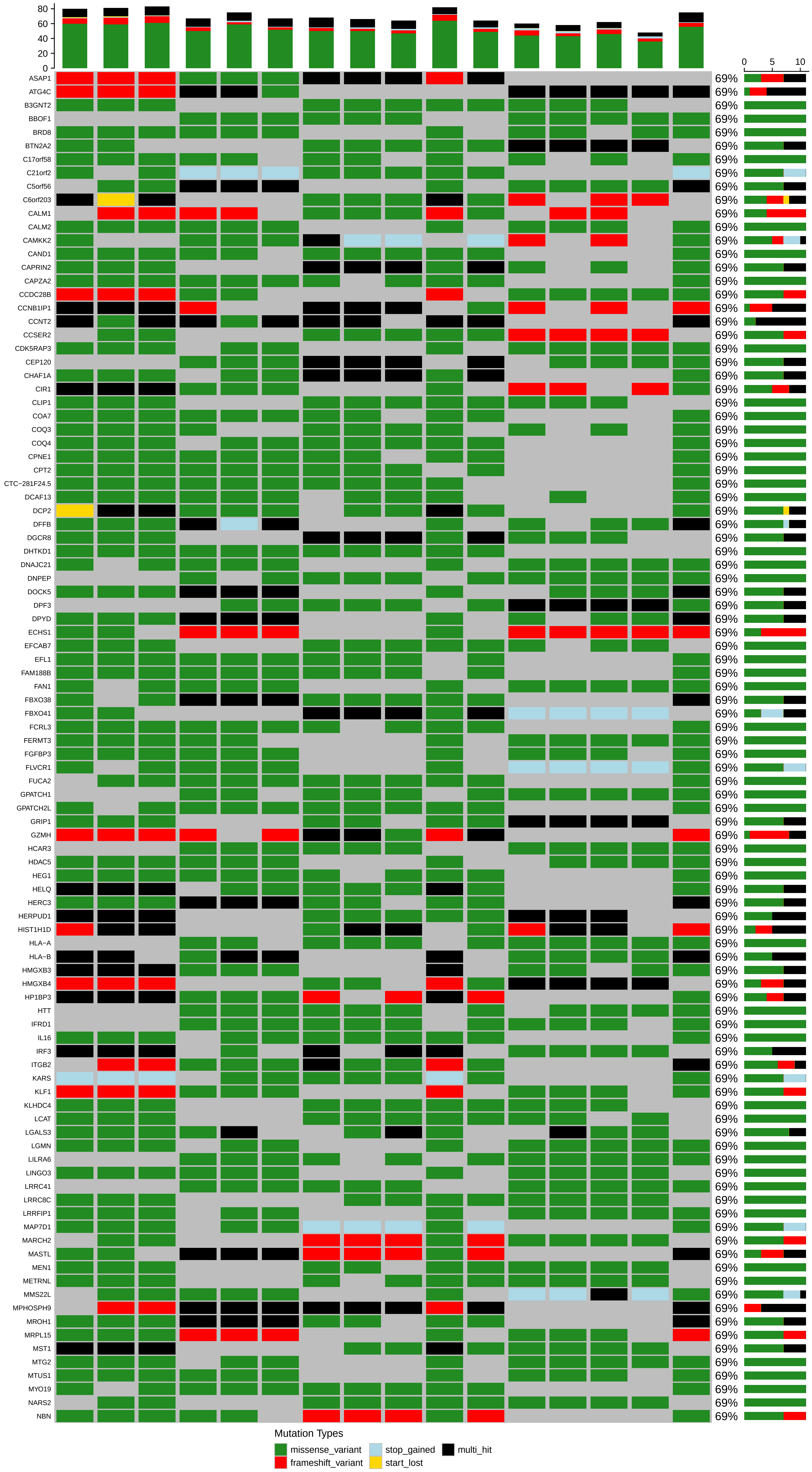

80
60
40
20
0
0
5
10
ASAP1
ATG4C
B3GNT2
BBOF1
BRD8
BTN2A2
C17orf58
C21orf2
C5orf56
C6orf203
CALM1
CALM2
CAMKK2
CAND1
CAPRIN2
CAPZA2
CCDC28B
CCNB1IP1
CCNT2
CCSER2
CDK5RAP3
CEP120
CHAF1A
CIR1
CLIP1
COA7
COQ3
COQ4
CPNE1
CPT2
CTC−281F24.5
DCAF13
DCP2
DFFB
DGCR8
DHTKD1
DNAJC21
DNPEP
DOCK5
DPF3
DPYD
ECHS1
EFCAB7
EFL1
FAM188B
FAN1
FBXO38
FBXO41
FCRL3
FERMT3
FGFBP3
FLVCR1
FUCA2
GPATCH1
GPATCH2L
GRIP1
GZMH
HCAR3
HDAC5
HEG1
HELQ
HERC3
HERPUD1
HIST1H1D
HLA−A
HLA−B
HMGXB3
HMGXB4
HP1BP3
HTT
IFRD1
IL16
IRF3
ITGB2
KARS
KLF1
KLHDC4
LCAT
LGALS3
LGMN
LILRA6
LINGO3
LRRC41
LRRC8C
LRRFIP1
MAP7D1
MARCH2
MASTL
MEN1
METRNL
MMS22L
MPHOSPH9
MROH1
MRPL15
MST1
MTG2
MTUS1
MYO19
NARS2
NBN
69%
Mutation Types
missense_variant
frameshift_variant
stop_gained
start_lost
multi_hit

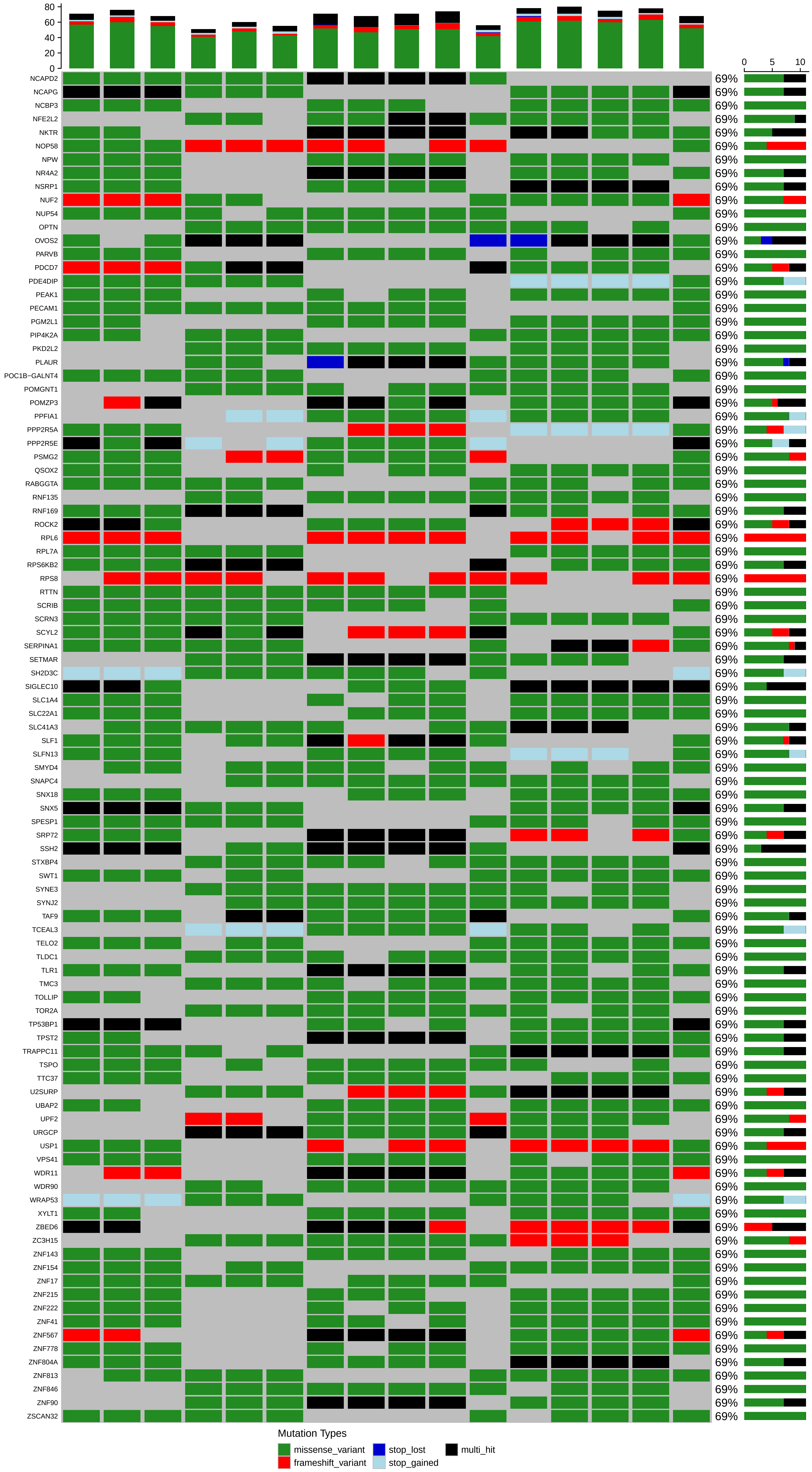

80
60
40
20
0
0
5
10
NCAPD2
NCAPG
NCBP3
NFE2L2
NKTR
NOP58
NPW
NR4A2
NSRP1
NUF2
NUP54
OPTN
OVOS2
PARVB
PDCD7
PDE4DIP
PEAK1
PECAM1
PGM2L1
PIP4K2A
PKD2L2
PLAUR
POC1B−GALNT4
POMGNT1
POMZP3
PPFIA1
PPP2R5A
PPP2R5E
PSMG2
QSOX2
RABGGTA
RNF135
RNF169
ROCK2
RPL6
RPL7A
RPS6KB2
RPS8
RTTN
SCRIB
SCRN3
SCYL2
SERPINA1
SETMAR
SH2D3C
SIGLEC10
SLC1A4
SLC22A1
SLC41A3
SLF1
SLFN13
SMYD4
SNAPC4
SNX18
SNX5
SPESP1
SRP72
SSH2
STXBP4
SWT1
SYNE3
SYNJ2
TAF9
TCEAL3
TELO2
TLDC1
TLR1
TMC3
TOLLIP
TOR2A
TP53BP1
TPST2
TRAPPC11
TSPO
TTC37
U2SURP
UBAP2
UPF2
URGCP
USP1
VPS41
WDR11
WDR90
WRAP53
XYLT1
ZBED6
ZC3H15
ZNF143
ZNF154
ZNF17
ZNF215
ZNF222
ZNF41
ZNF567
ZNF778
ZNF804A
ZNF813
ZNF846
ZNF90
ZSCAN32
69%
Mutation Types
missense_variant
frameshift_variant
stop_lost
stop_gained
multi_hit

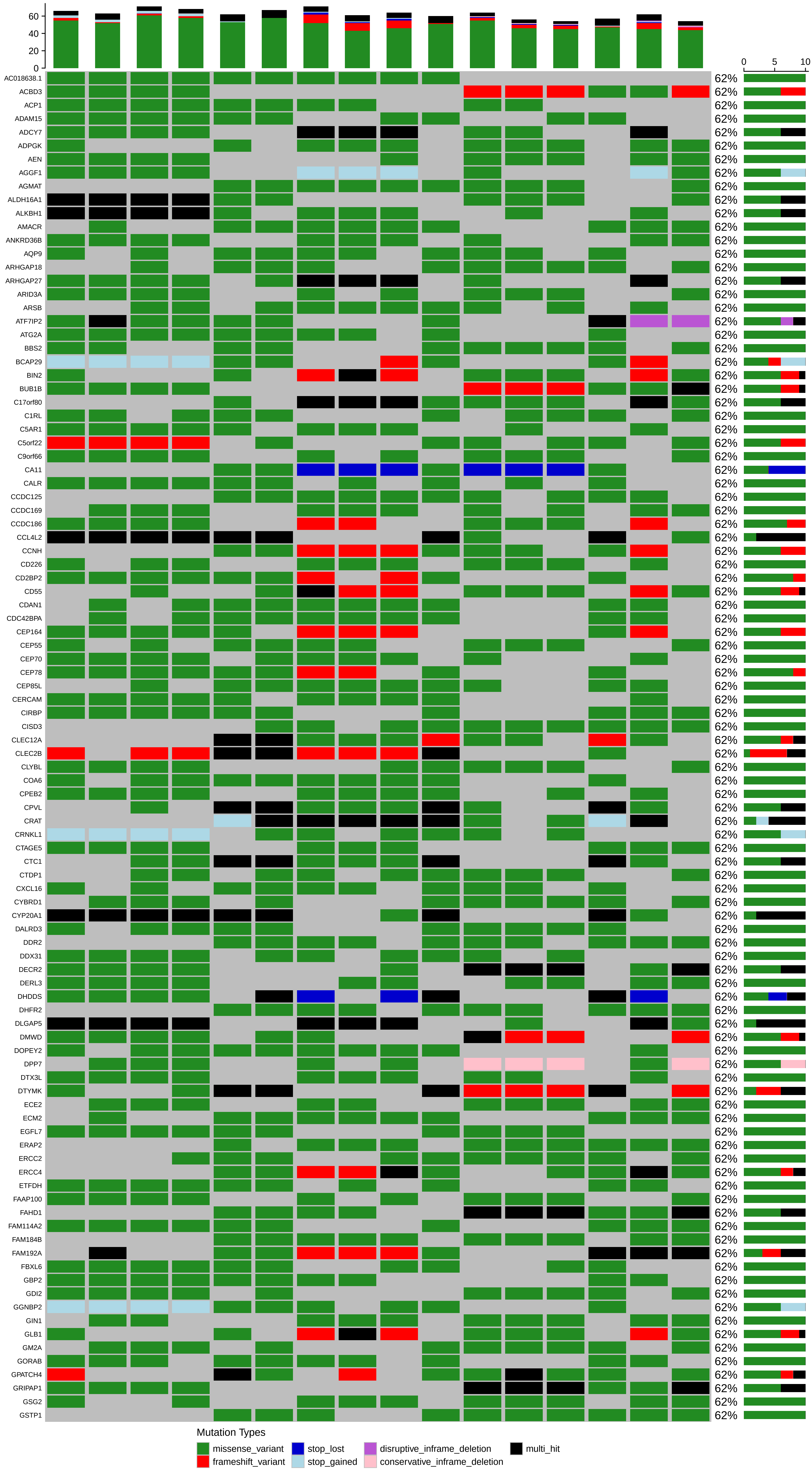

AC018638.1
ACBD3
ACP1
ADAM15
ADCY7
ADPGK
AEN
AGGF1
AGMAT
ALDH16A1
ALKBH1
AMACR
ANKRD36B
AQP9
ARHGAP18
ARHGAP27
ARID3A
ARSB
ATF7IP2
ATG2A
BBS2
BCAP29
BIN2
BUB1B
C17orf80
C1RL
C5AR1
C5orf22
C9orf66
CA11
CALR
CCDC125
CCDC169
CCDC186
CCL4L2
CCNH
CD226
CD2BP2
CD55
CDAN1
CDC42BPA
CEP164
CEP55
CEP70
CEP78
CEP85L
CERCAM
CIRBP
CISD3
CLEC12A
CLEC2B
CLYBL
COA6
CPEB2
CPVL
CRAT
CRNKL1
CTAGE5
CTC1
CTDP1
CXCL16
CYBRD1
CYP20A1
DALRD3
DDR2
DDX31
DECR2
DERL3
DHDDS
DHFR2
DLGAP5
DMWD
DOPEY2
DPP7
DTX3L
DTYMK
ECE2
ECM2
EGFL7
ERAP2
ERCC2
ERCC4
ETFDH
FAAP100
FAHD1
FAM114A2
FAM184B
FAM192A
FBXL6
GBP2
GDI2
GGNBP2
GIN1
GLB1
GM2A
GORAB
GPATCH4
GRIPAP1
GSG2
GSTP1
62%
Mutation Types
missense_variant
frameshift_variant
stop_lost
stop_gained
disruptive_inframe_deletion
conservative_inframe_deletion
multi_hit

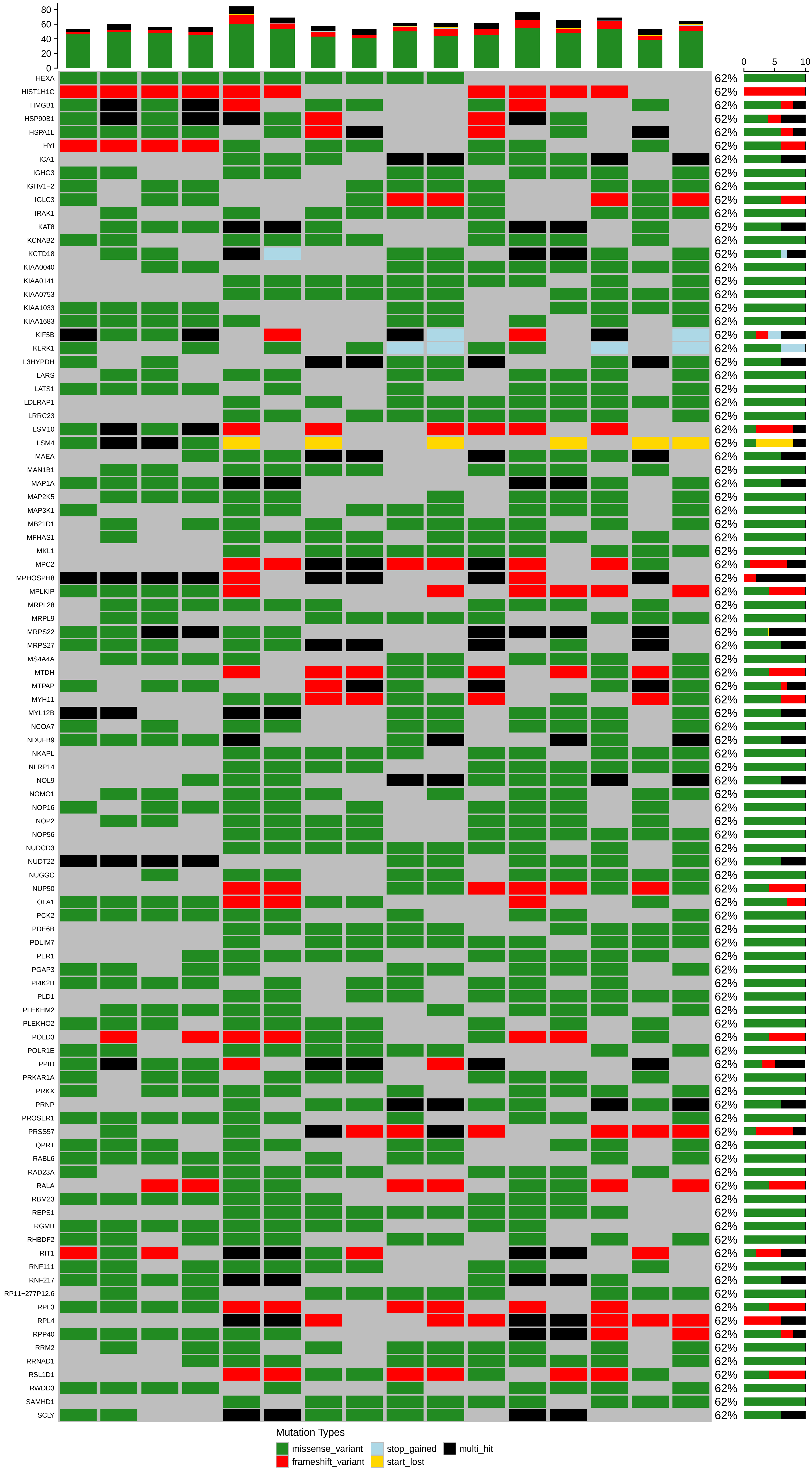
80
60
40
20
0
0
5
10
HEXA
HIST1H1C
HMGB1
HSP90B1
HSPA1L
HYI
ICA1
IGHG3
IGHV1−2
IGLC3
IRAK1
KAT8
KCNAB2
KCTD18
KIAA0040
KIAA0141
KIAA0753
KIAA1033
KIAA1683
KIF5B
KLRK1
L3HYPDH
LARS
LATS1
LDLRAP1
LRRC23
LSM10
LSM4
MAEA
MAN1B1
MAP1A
MAP2K5
MAP3K1
MB21D1
MFHAS1
MKL1
MPC2
MPHOSPH8
MPLKIP
MRPL28
MRPL9
MRPS22
MRPS27
MS4A4A
MTDH
MTPAP
MYH11
MYL12B
NCOA7
NDUFB9
NKAPL
NLRP14
NOL9
NOMO1
NOP16
NOP2
NOP56
NUDCD3
NUDT22
NUGGC
NUP50
OLA1
PCK2
PDE6B
PDLIM7
PER1
PGAP3
PI4K2B
PLD1
PLEKHM2
PLEKHO2
POLD3
POLR1E
PPID
PRKAR1A
PRKX
PRNP
PROSER1
PRSS57
QPRT
RABL6
RAD23A
RALA
RBM23
REPS1
RGMB
RHBDF2
RIT1
RNF111
RNF217
RP11−277P12.6
RPL3
RPL4
RPP40
RRM2
RRNAD1
RSL1D1
RWDD3
SAMHD1
SCLY
62%
Mutation Types
missense_variant
frameshift_variant
stop_gained
start_lost
multi_hit

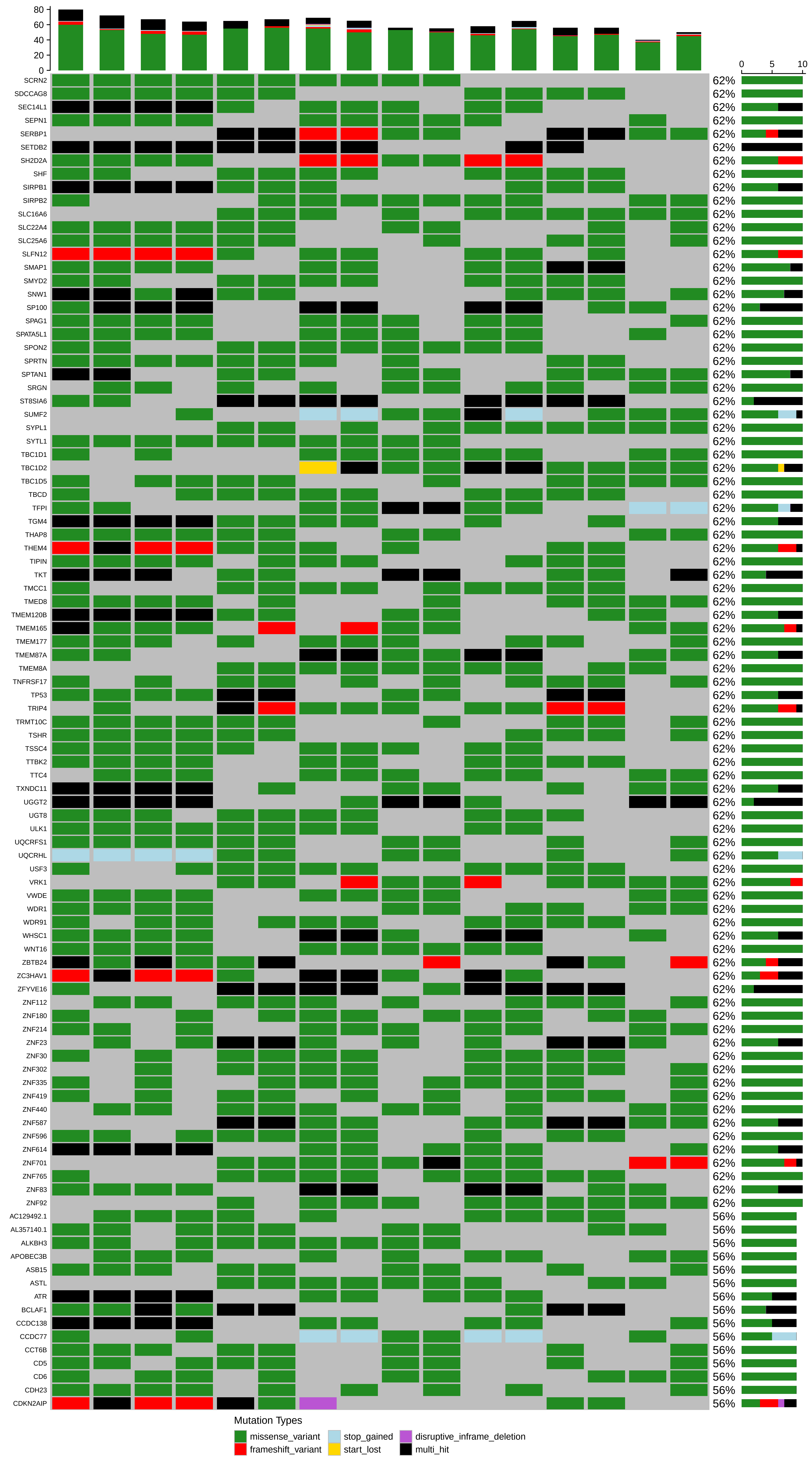
80
60
40
20
0
0
5
10
SCRN2
SDCCAG8
SEC14L1
SEPN1
SERBP1
SETDB2
SH2D2A
SHF
SIRPB1
SIRPB2
SLC16A6
SLC22A4
SLC25A6
SLFN12
SMAP1
SMYD2
SNW1
SP100
SPAG1
SPATA5L1
SPON2
SPRTN
SPTAN1
SRGN
ST8SIA6
SUMF2
SYPL1
SYTL1
TBC1D1
TBC1D2
TBC1D5
TBCD
TFPI
TGM4
THAP8
THEM4
TIPIN
TKT
TMCC1
TMED8
TMEM120B
TMEM165
TMEM177
TMEM87A
TMEM8A
TNFRSF17
TP53
TRIP4
TRMT10C
TSHR
TSSC4
TTBK2
TTC4
TXNDC11
UGGT2
UGT8
ULK1
UQCRFS1
UQCRHL
USF3
VRK1
VWDE
WDR1
WDR91
WHSC1
WNT16
ZBTB24
ZC3HAV1
ZFYVE16
ZNF112
ZNF180
ZNF214
ZNF23
ZNF30
ZNF302
ZNF335
ZNF419
ZNF440
ZNF587
ZNF596
ZNF614
ZNF701
ZNF765
ZNF83
ZNF92
AC129492.1
AL357140.1
ALKBH3
APOBEC3B
ASB15
ASTL
ATR
BCLAF1
CCDC138
CCDC77
CCT6B
CD5
CD6
CDH23
CDKN2AIP
62% (×85)
56% (×16)
Mutation Types
missense_variant
frameshift_variant
stop_gained
start_lost
disruptive_inframe_deletion
multi_hit

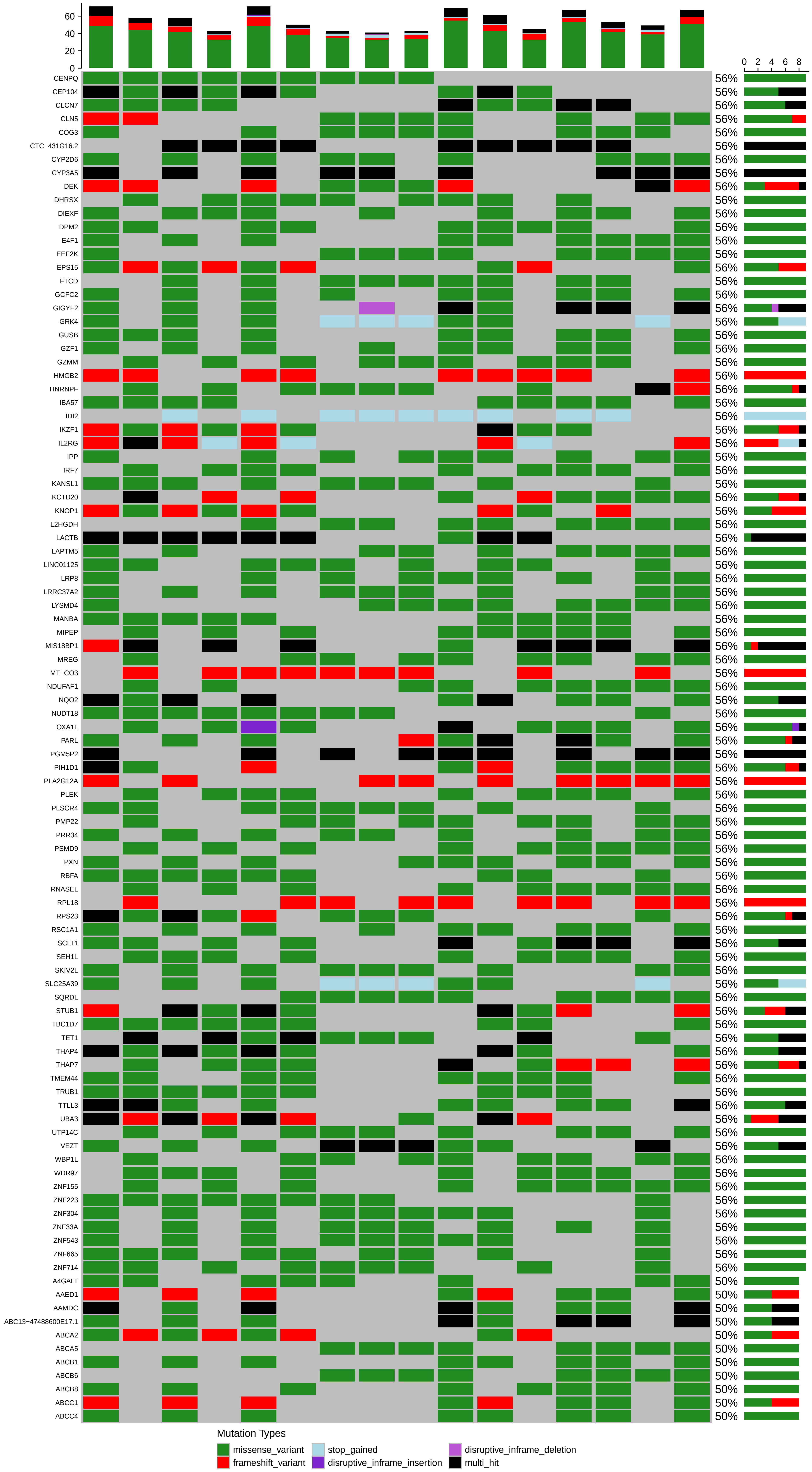

60
40
20
0
0 2 4 6 8
CENPQ
CEP104
CLCN7
CLN5
COG3
CTC−431G16.2
CYP2D6
CYP3A5
DEK
DHRSX
DIEXF
DPM2
E4F1
EEF2K
EPS15
FTCD
GCFC2
GIGYF2
GRK4
GUSB
GZF1
GZMM
HMGB2
HNRNPF
IBA57
IDI2
IKZF1
IL2RG
IPP
IRF7
KANSL1
KCTD20
KNOP1
L2HGDH
LACTB
LAPTM5
LINC01125
LRP8
LRRC37A2
LYSMD4
MANBA
MIPEP
MIS18BP1
MREG
MT−CO3
NDUFAF1
NQO2
NUDT18
OXA1L
PARL
PGM5P2
PIH1D1
PLA2G12A
PLEK
PLSCR4
PMP22
PRR34
PSMD9
PXN
RBFA
RNASEL
RPL18
RPS23
RSC1A1
SCLT1
SEH1L
SKIV2L
SLC25A39
SQRDL
STUB1
TBC1D7
TET1
THAP4
THAP7
TMEM44
TRUB1
TTLL3
UBA3
UTP14C
VEZT
WBP1L
WDR97
ZNF155
ZNF223
ZNF304
ZNF33A
ZNF543
ZNF665
ZNF714
A4GALT
AAED1
AAMDC
ABC13−47488600E17.1
ABCA2
ABCA5
ABCB1
ABCB6
ABCB8
ABCC1
ABCC4
56%
50%
Mutation Types
missense_variant
frameshift_variant
stop_gained
disruptive_inframe_insertion
disruptive_inframe_deletion
multi_hit

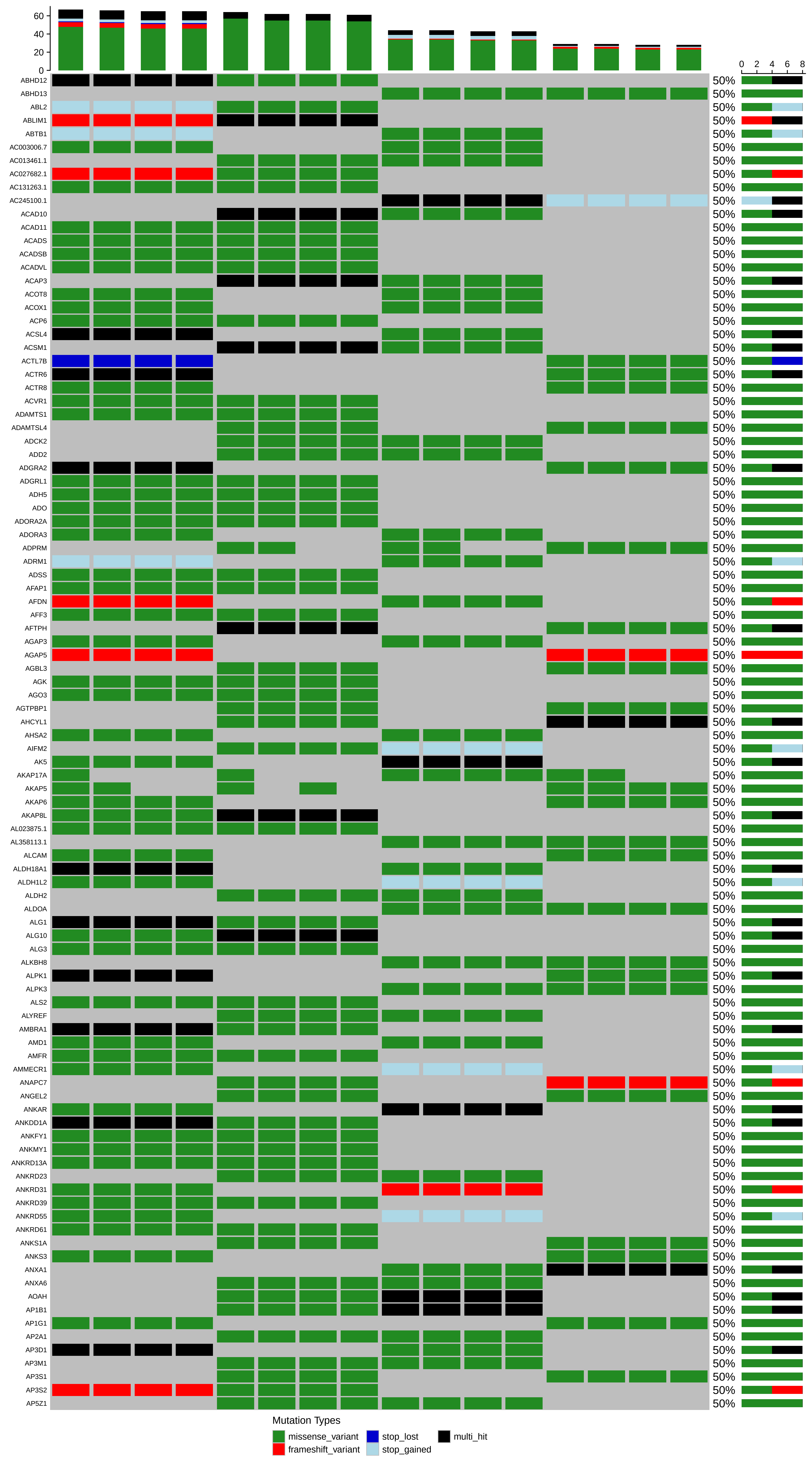

ABHD12
ABHD13
ABL2
ABLIM1
ABTB1
AC003006.7
AC013461.1
AC027682.1
AC131263.1
AC245100.1
ACAD10
ACAD11
ACADS
ACADSB
ACADVL
ACAP3
ACOT8
ACOX1
ACP6
ACSL4
ACSM1
ACTL7B
ACTR6
ACTR8
ACVR1
ADAMTS1
ADAMTSL4
ADCK2
ADD2
ADGRA2
ADGRL1
ADH5
ADO
ADORA2A
ADORA3
ADPRM
ADRM1
ADSS
AFAP1
AFDN
AFF3
AFTPH
AGAP3
AGAP5
AGBL3
AGK
AGO3
AGTPBP1
AHCYL1
AHSA2
AIFM2
AK5
AKAP17A
AKAP5
AKAP6
AKAP8L
AL023875.1
AL358113.1
ALCAM
ALDH18A1
ALDH1L2
ALDH2
ALDOA
ALG1
ALG10
ALG3
ALKBH8
ALPK1
ALPK3
ALS2
ALYREF
AMBRA1
AMD1
AMFR
AMMECR1
ANAPC7
ANGEL2
ANKAR
ANKDD1A
ANKFY1
ANKMY1
ANKRD13A
ANKRD23
ANKRD31
ANKRD39
ANKRD55
ANKRD61
ANKS1A
ANKS3
ANXA1
ANXA6
AOAH
AP1B1
AP1G1
AP2A1
AP3D1
AP3M1
AP3S1
AP3S2
AP5Z1
Mutation Types
missense_variant
frameshift_variant
stop_lost
stop_gained
multi_hit

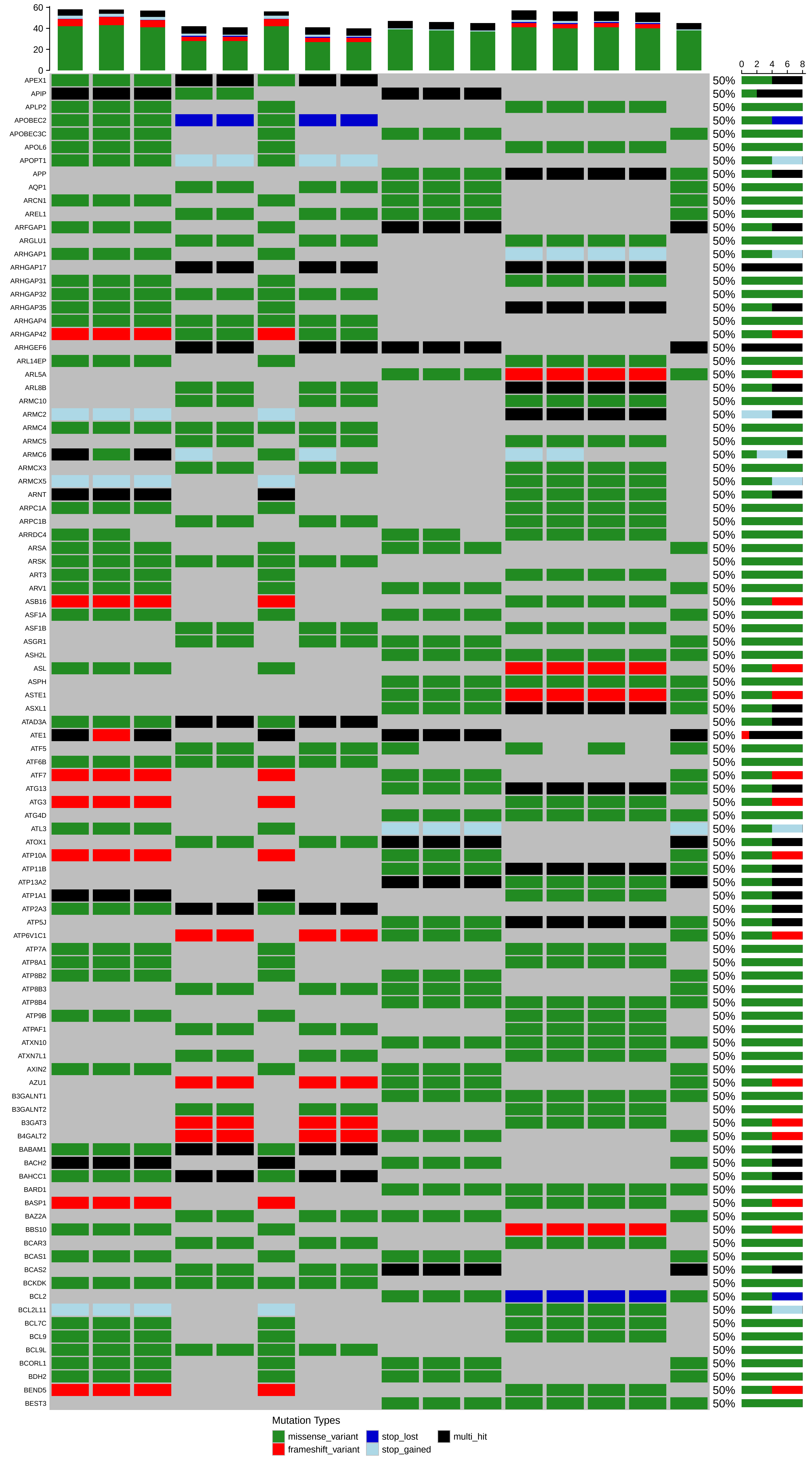

APEX1
APIP
APLP2
APOBEC2
APOBEC3C
APOL6
APOPT1
APP
AQP1
ARCN1
AREL1
ARFGAP1
ARGLU1
ARHGAP1
ARHGAP17
ARHGAP31
ARHGAP32
ARHGAP35
ARHGAP4
ARHGAP42
ARHGEF6
ARL14EP
ARL5A
ARL8B
ARMC10
ARMC2
ARMC4
ARMC5
ARMC6
ARMCX3
ARMCX5
ARNT
ARPC1A
ARPC1B
ARRDC4
ARSA
ARSK
ART3
ARV1
ASB16
ASF1A
ASF1B
ASGR1
ASH2L
ASL
ASPH
ASTE1
ASXL1
ATAD3A
ATE1
ATF5
ATF6B
ATF7
ATG13
ATG3
ATG4D
ATL3
ATOX1
ATP10A
ATP11B
ATP13A2
ATP1A1
ATP2A3
ATP5J
ATP6V1C1
ATP7A
ATP8A1
ATP8B2
ATP8B3
ATP8B4
ATP9B
ATPAF1
ATXN10
ATXN7L1
AXIN2
AZU1
B3GALNT1
B3GALNT2
B3GAT3
B4GALT2
BABAM1
BACH2
BAHCC1
BARD1
BASP1
BAZ2A
BBS10
BCAR3
BCAS1
BCAS2
BCKDK
BCL2
BCL2L11
BCL7C
BCL9
BCL9L
BCORL1
BDH2
BEND5
BEST3
50%
Mutation Types
missense_variant
frameshift_variant
stop_lost
stop_gained
multi_hit

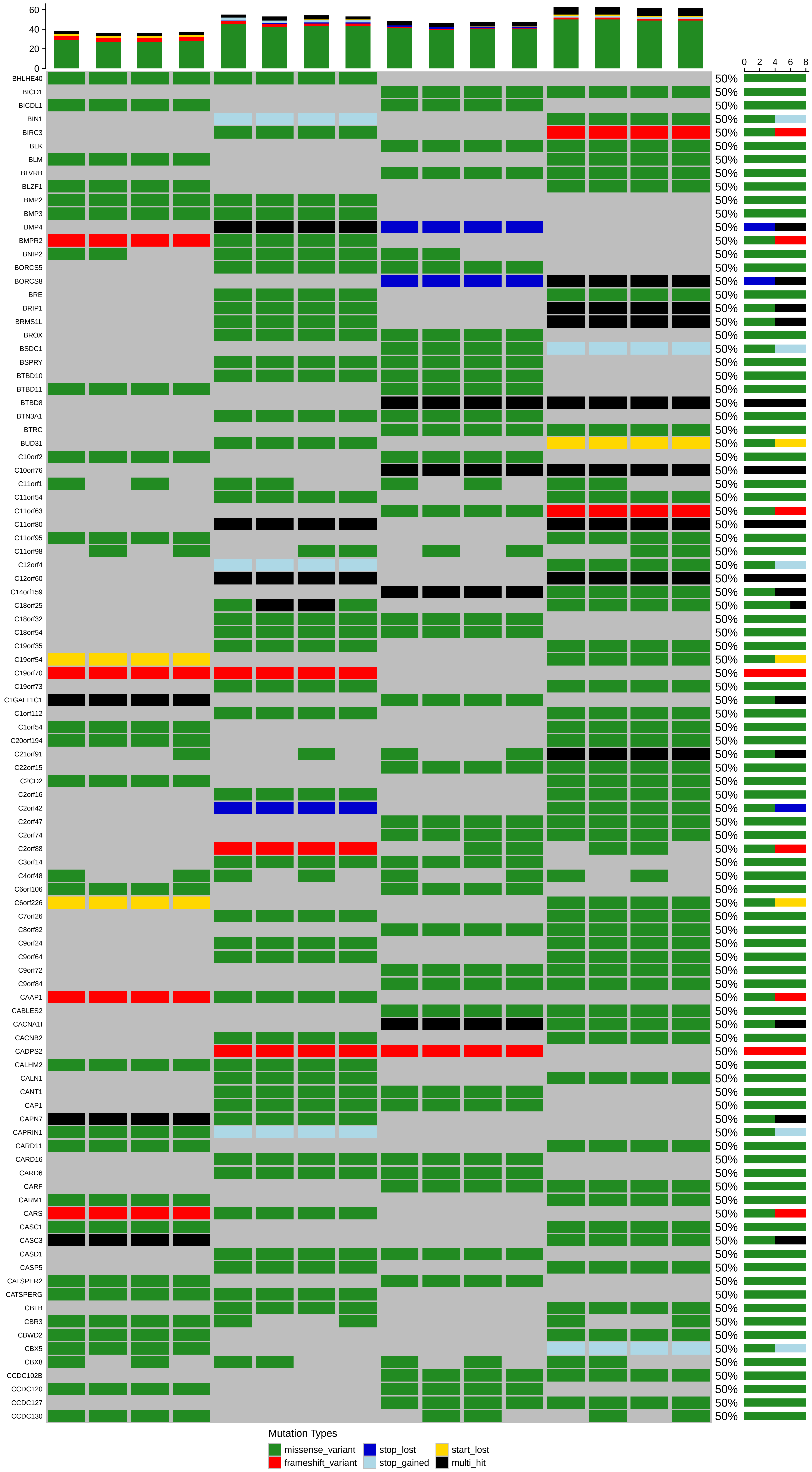

60
40
20
0
0 2 4 6 8
BHLHE40
BICD1
BICDL1
BIN1
BIRC3
BLK
BLM
BLVRB
BLZF1
BMP2
BMP3
BMP4
BMPR2
BNIP2
BORCS5
BORCS8
BRE
BRIP1
BRMS1L
BROX
BSDC1
BSPRY
BTBD10
BTBD11
BTBD8
BTN3A1
BTRC
BUD31
C10orf2
C10orf76
C11orf1
C11orf54
C11orf63
C11orf80
C11orf95
C11orf98
C12orf4
C12orf60
C14orf159
C18orf25
C18orf32
C18orf54
C19orf35
C19orf54
C19orf70
C19orf73
C1GALT1C1
C1orf112
C1orf54
C20orf194
C21orf91
C22orf15
C2CD2
C2orf16
C2orf42
C2orf47
C2orf74
C2orf88
C3orf14
C4orf48
C6orf106
C6orf226
C7orf26
C8orf82
C9orf24
C9orf64
C9orf72
C9orf84
CAAP1
CABLES2
CACNA1I
CACNB2
CADPS2
CALHM2
CALN1
CANT1
CAP1
CAPN7
CAPRIN1
CARD11
CARD16
CARD6
CARF
CARM1
CARS
CASC1
CASC3
CASD1
CASP5
CATSPER2
CATSPERG
CBLB
CBR3
CBWD2
CBX5
CBX8
CCDC102B
CCDC120
CCDC127
CCDC130
50%
Mutation Types
missense_variant
frameshift_variant
stop_lost
stop_gained
start_lost
multi_hit

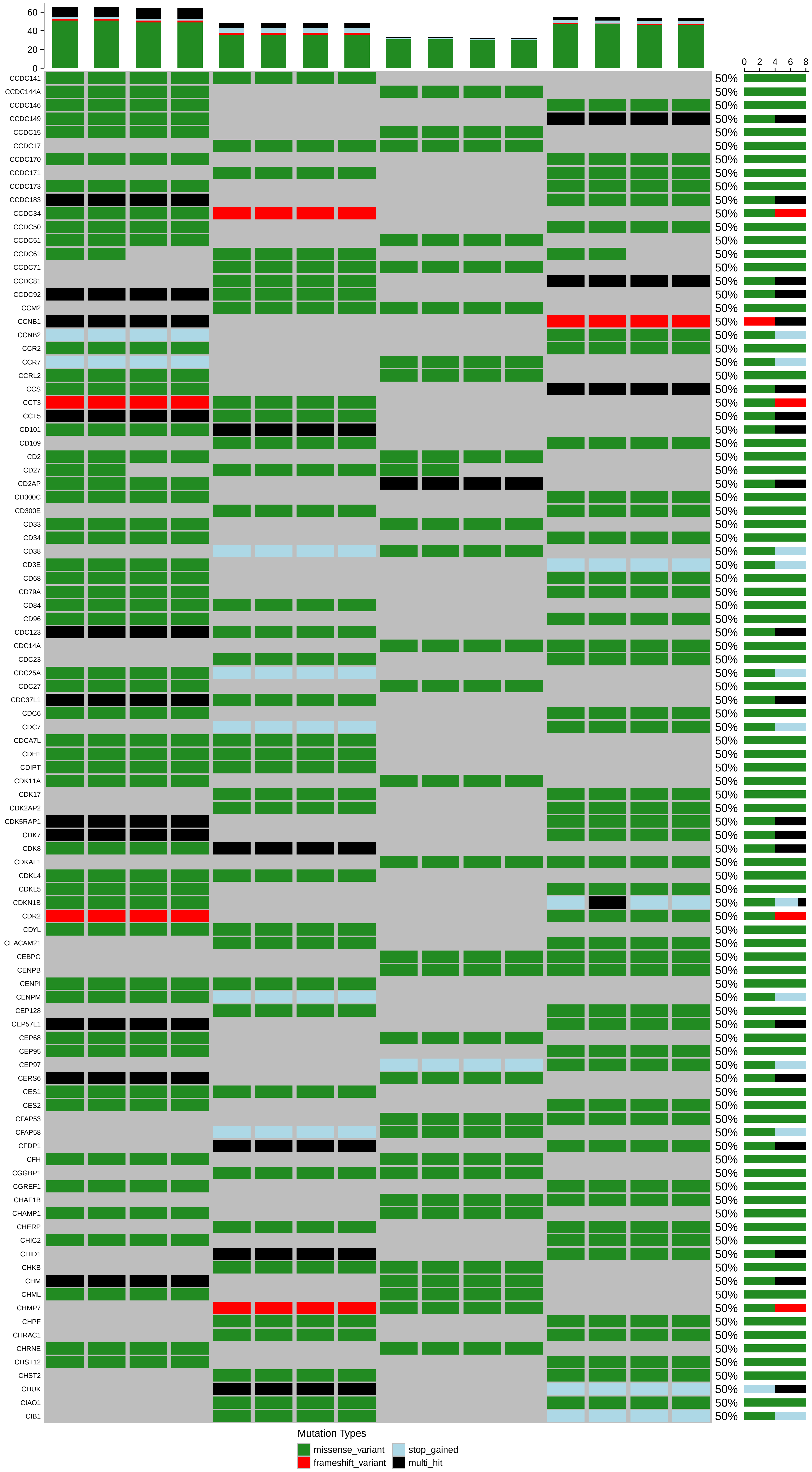

CCDC141
CCDC144A
CCDC146
CCDC149
CCDC15
CCDC17
CCDC170
CCDC171
CCDC173
CCDC183
CCDC34
CCDC50
CCDC51
CCDC61
CCDC71
CCDC81
CCDC92
CCM2
CCNB1
CCNB2
CCR2
CCR7
CCRL2
CCS
CCT3
CCT5
CD101
CD109
CD2
CD27
CD2AP
CD300C
CD300E
CD33
CD34
CD38
CD3E
CD68
CD79A
CD84
CD96
CDC123
CDC14A
CDC23
CDC25A
CDC27
CDC37L1
CDC6
CDC7
CDCA7L
CDH1
CDIPT
CDK11A
CDK17
CDK2AP2
CDK5RAP1
CDK7
CDK8
CDKAL1
CDKL4
CDKL5
CDKN1B
CDR2
CDYL
CEACAM21
CEBPG
CENPB
CENPI
CENPM
CEP128
CEP57L1
CEP68
CEP95
CEP97
CERS6
CES1
CES2
CFAP53
CFAP58
CFDP1
CFH
CGGBP1
CGREF1
CHAF1B
CHAMP1
CHERP
CHIC2
CHID1
CHKB
CHM
CHML
CHMP7
CHPF
CHRAC1
CHRNE
CHST12
CHST2
CHUK
CIAO1
CIB1
50%
Mutation Types
missense_variant
frameshift_variant
stop_gained
multi_hit

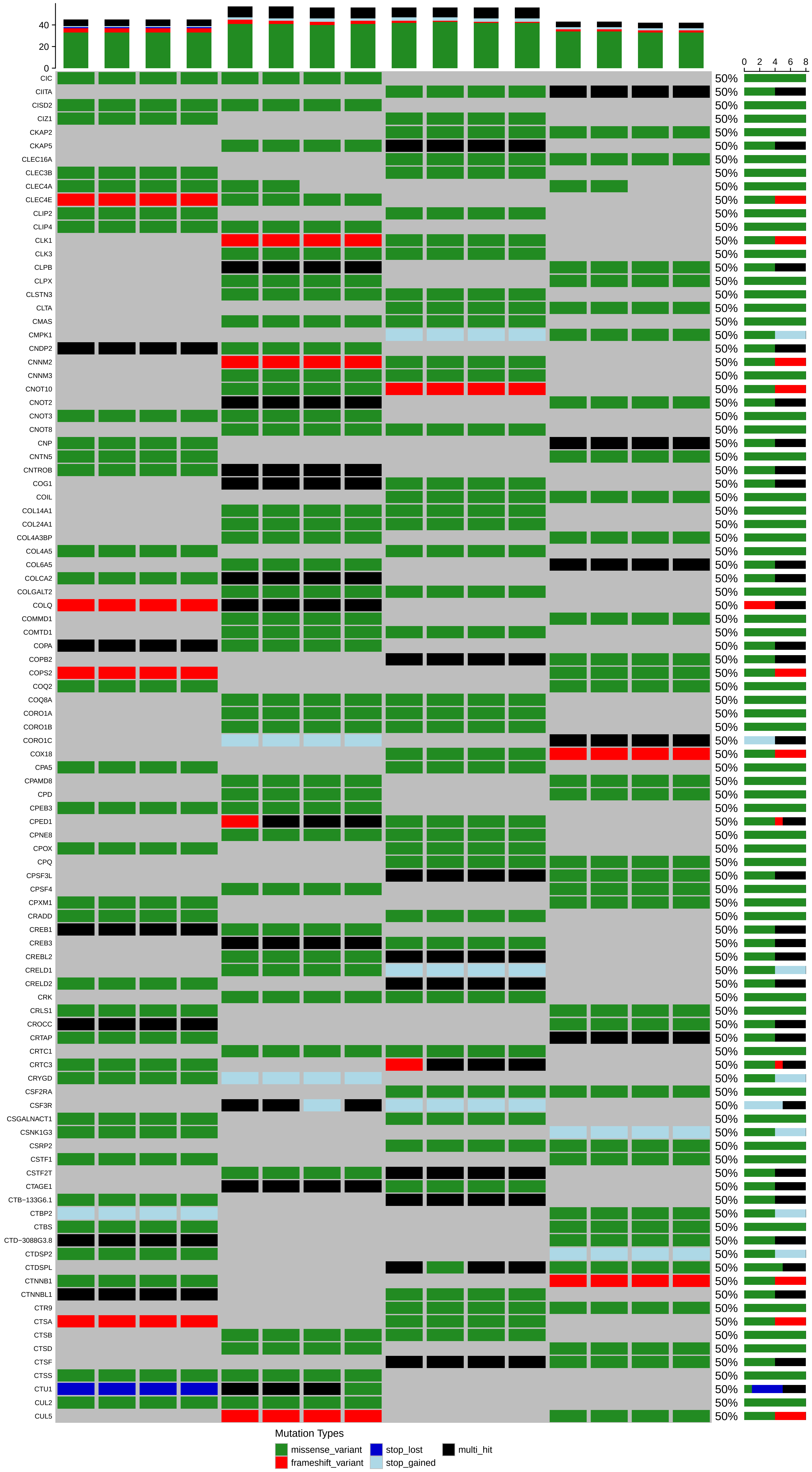
CIC
CIITA
CISD2
CIZ1
CKAP2
CKAP5
CLEC16A
CLEC3B
CLEC4A
CLEC4E
CLIP2
CLIP4
CLK1
CLK3
CLPB
CLPX
CLSTN3
CLTA
CMAS
CMPK1
CNDP2
CNNM2
CNNM3
CNOT10
CNOT2
CNOT3
CNOT8
CNP
CNTN5
CNTROB
COG1
COIL
COL14A1
COL24A1
COL4A3BP
COL4A5
COL6A5
COLCA2
COLGALT2
COLQ
COMMD1
COMTD1
COPA
COPB2
COPS2
COQ2
COQ8A
CORO1A
CORO1B
CORO1C
COX18
CPA5
CPAMD8
CPD
CPEB3
CPED1
CPNE8
CPOX
CPQ
CPSF3L
CPSF4
CPXM1
CRADD
CREB1
CREB3
CREBL2
CRELD1
CRELD2
CRK
CRLS1
CROCC
CRTAP
CRTC1
CRTC3
CRYGD
CSF2RA
CSF3R
CSGALNACT1
CSNK1G3
CSRP2
CSTF1
CSTF2T
CTAGE1
CTB−133G6.1
CTBP2
CTBS
CTD−3088G3.8
CTDSP2
CTDSPL
CTNNB1
CTNNBL1
CTR9
CTSA
CTSB
CTSD
CTSF
CTSS
CTU1
CUL2
CUL5
50%
Mutation Types
missense_variant
frameshift_variant
stop_lost
stop_gained
multi_hit

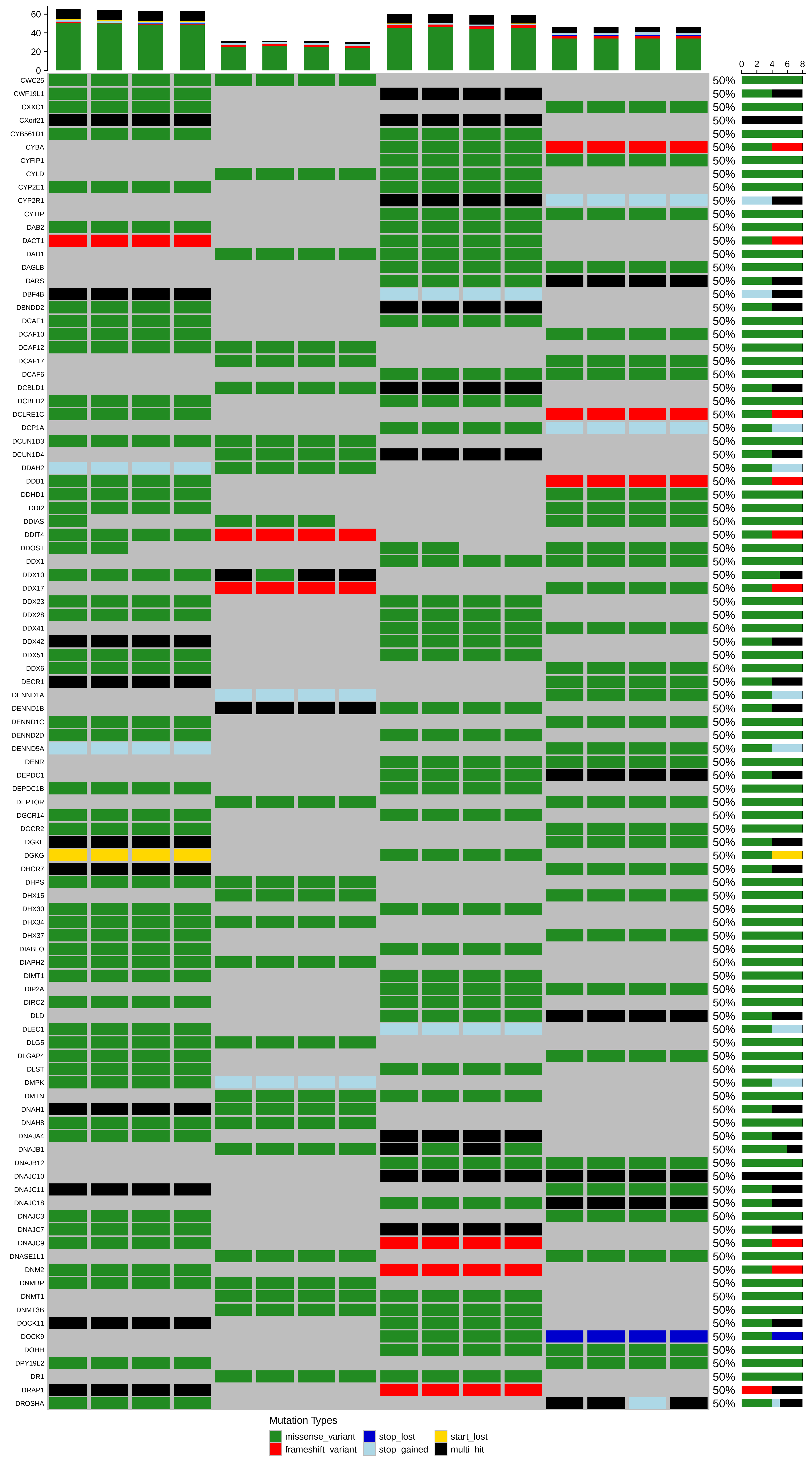

0
20
40
60
CWC25
CWF19L1
CXXC1
CXorf21
CYB561D1
CYBA
CYFIP1
CYLD
CYP2E1
CYP2R1
CYTIP
DAB2
DACT1
DAD1
DAGLB
DARS
DBF4B
DBNDD2
DCAF1
DCAF10
DCAF12
DCAF17
DCAF6
DCBLD1
DCBLD2
DCLRE1C
DCP1A
DCUN1D3
DCUN1D4
DDAH2
DDB1
DDHD1
DDI2
DDIAS
DDIT4
DDOST
DDX1
DDX10
DDX17
DDX23
DDX28
DDX41
DDX42
DDX51
DDX6
DECR1
DENND1A
DENND1B
DENND1C
DENND2D
DENND5A
DENR
DEPDC1
DEPDC1B
DEPTOR
DGCR14
DGCR2
DGKE
DGKG
DHCR7
DHPS
DHX15
DHX30
DHX34
DHX37
DIABLO
DIAPH2
DIMT1
DIP2A
DIRC2
DLD
DLEC1
DLG5
DLGAP4
DLST
DMPK
DMTN
DNAH1
DNAH8
DNAJA4
DNAJB1
DNAJB12
DNAJC10
DNAJC11
DNAJC18
DNAJC3
DNAJC7
DNAJC9
DNASE1L1
DNM2
DNMBP
DNMT1
DNMT3B
DOCK11
DOCK9
DOHH
DPY19L2
DR1
DRAP1
DROSHA
50%
0 2 4 6 8
Mutation Types
missense_variant
frameshift_variant
stop_lost
stop_gained
start_lost
multi_hit

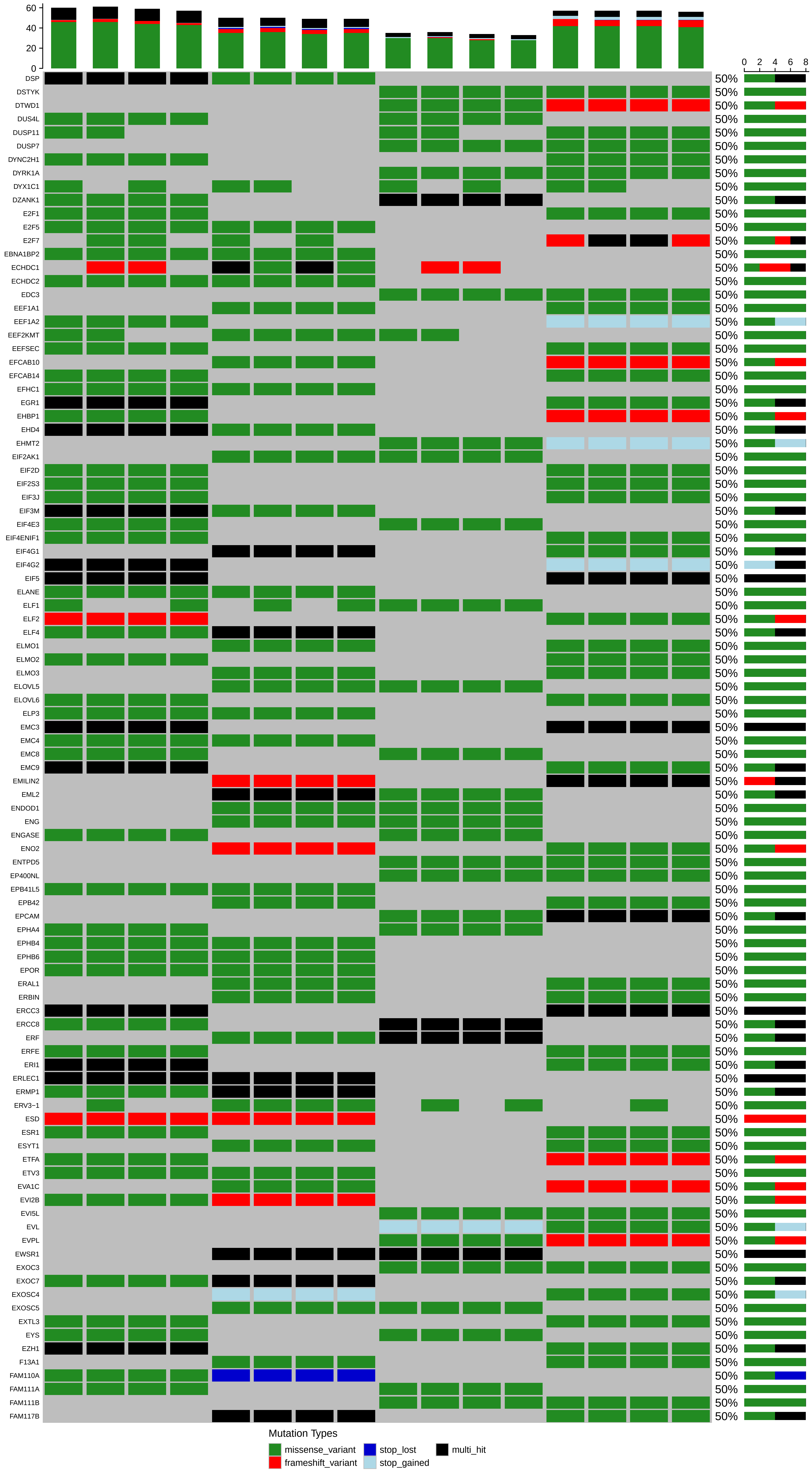

60
40
20
0
0 2 4 6 8
DSP
DSTYK
DTWD1
DUS4L
DUSP11
DUSP7
DYNC2H1
DYRK1A
DYX1C1
DZANK1
E2F1
E2F5
E2F7
EBNA1BP2
ECHDC1
ECHDC2
EDC3
EEF1A1
EEF1A2
EEF2KMT
EEFSEC
EFCAB10
EFCAB14
EFHC1
EGR1
EHBP1
EHD4
EHMT2
EIF2AK1
EIF2D
EIF2S3
EIF3J
EIF3M
EIF4E3
EIF4ENIF1
EIF4G1
EIF4G2
EIF5
ELANE
ELF1
ELF2
ELF4
ELMO1
ELMO2
ELMO3
ELOVL5
ELOVL6
ELP3
EMC3
EMC4
EMC8
EMC9
EMILIN2
EML2
ENDOD1
ENG
ENGASE
ENO2
ENTPD5
EP400NL
EPB41L5
EPB42
EPCAM
EPHA4
EPHB4
EPHB6
EPOR
ERAL1
ERBIN
ERCC3
ERCC8
ERF
ERFE
ERI1
ERLEC1
ERMP1
ERV3−1
ESD
ESR1
ESYT1
ETFA
ETV3
EVA1C
EVI2B
EVI5L
EVL
EVPL
EWSR1
EXOC3
EXOC7
EXOSC4
EXOSC5
EXTL3
EYS
EZH1
F13A1
FAM110A
FAM111A
FAM111B
FAM117B
50%
Mutation Types
missense_variant
frameshift_variant
stop_lost
stop_gained
multi_hit

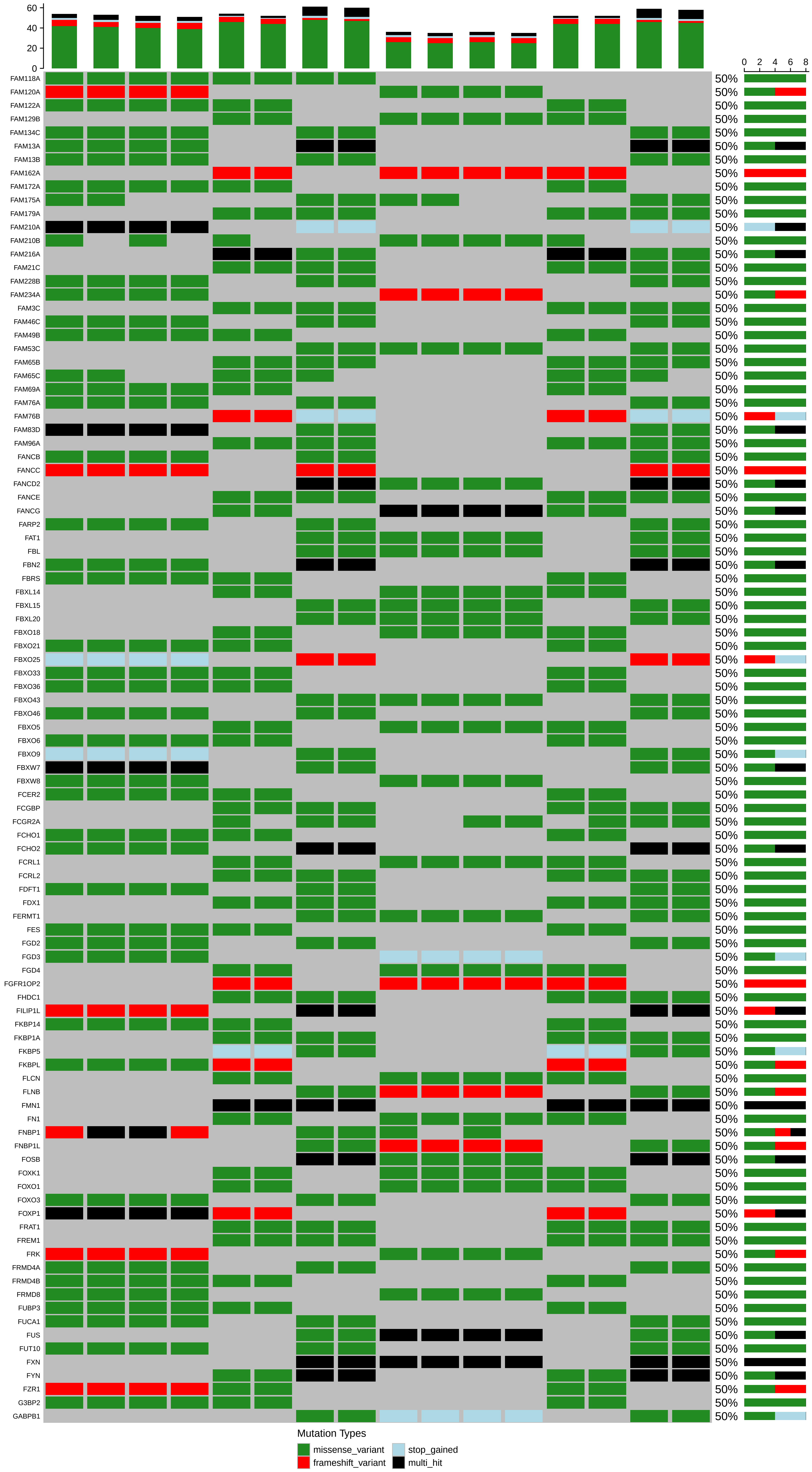

60
40
20
0
0 2 4 6 8
FAM118A
FAM120A
FAM122A
FAM129B
FAM134C
FAM13A
FAM13B
FAM162A
FAM172A
FAM175A
FAM179A
FAM210A
FAM210B
FAM216A
FAM21C
FAM228B
FAM234A
FAM3C
FAM46C
FAM49B
FAM53C
FAM65B
FAM65C
FAM69A
FAM76A
FAM76B
FAM83D
FAM96A
FANCB
FANCC
FANCD2
FANCE
FANCG
FARP2
FAT1
FBL
FBN2
FBRS
FBXL14
FBXL15
FBXL20
FBXO18
FBXO21
FBXO25
FBXO33
FBXO36
FBXO43
FBXO46
FBXO5
FBXO6
FBXO9
FBXW7
FBXW8
FCER2
FCGBP
FCGR2A
FCHO1
FCHO2
FCRL1
FCRL2
FDFT1
FDX1
FERMT1
FES
FGD2
FGD3
FGD4
FGFR1OP2
FHDC1
FILIP1L
FKBP14
FKBP1A
FKBP5
FKBPL
FLCN
FLNB
FMN1
FN1
FNBP1
FNBP1L
FOSB
FOXK1
FOXO1
FOXO3
FOXP1
FRAT1
FREM1
FRK
FRMD4A
FRMD4B
FRMD8
FUBP3
FUCA1
FUS
FUT10
FXN
FYN
FZR1
G3BP2
GABPB1
50%
Mutation Types
missense_variant
frameshift_variant
stop_gained
multi_hit

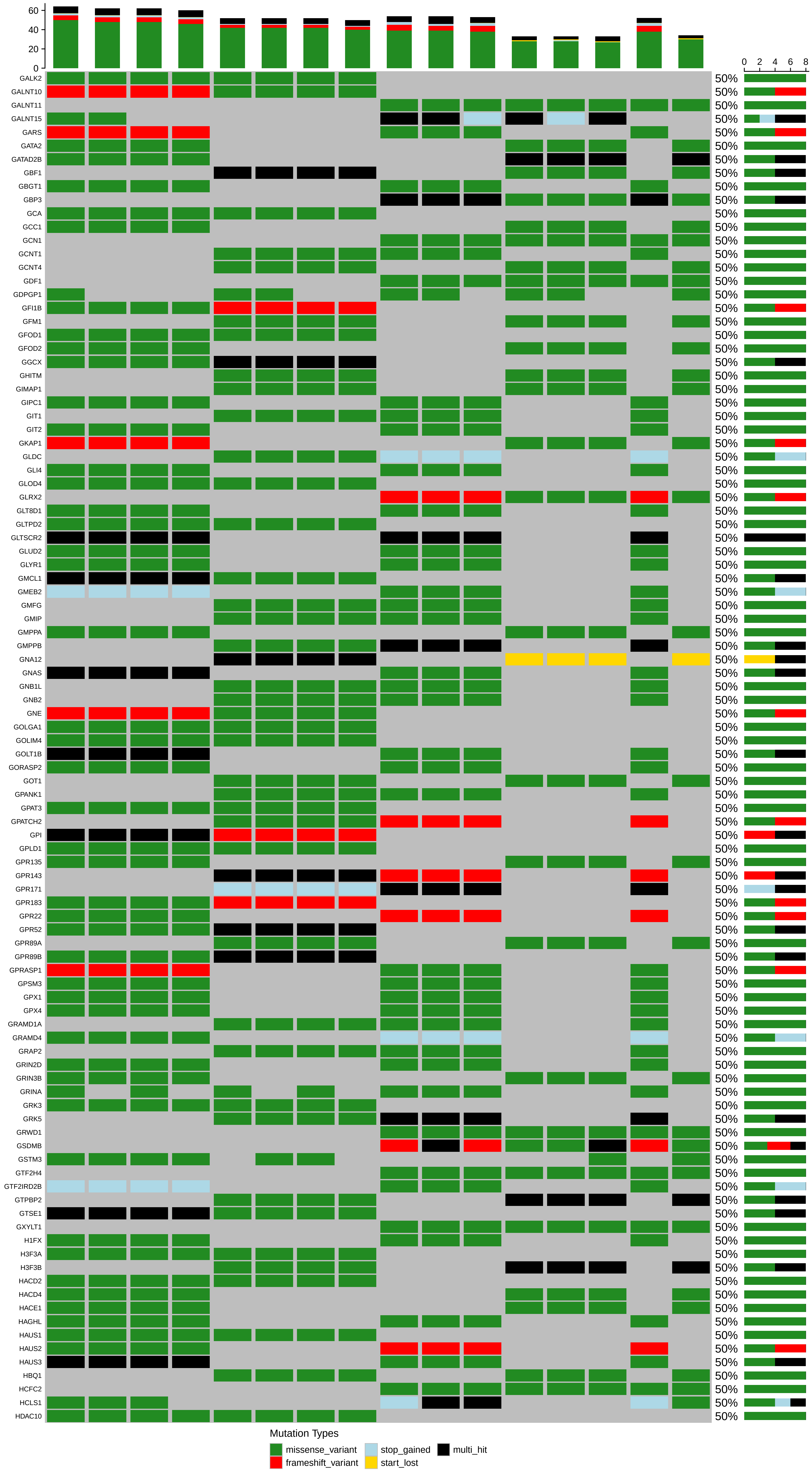

60
40
20
0
0 2 4 6 8
GALK2
GALNT10
GALNT11
GALNT15
GARS
GATA2
GATAD2B
GBF1
GBGT1
GBP3
GCA
GCC1
GCN1
GCNT1
GCNT4
GDF1
GDPGP1
GFI1B
GFM1
GFOD1
GFOD2
GGCX
GHITM
GIMAP1
GIPC1
GIT1
GIT2
GKAP1
GLDC
GLI4
GLOD4
GLRX2
GLT8D1
GLTPD2
GLTSCR2
GLUD2
GLYR1
GMCL1
GMEB2
GMFG
GMIP
GMPPA
GMPPB
GNA12
GNAS
GNB1L
GNB2
GNE
GOLGA1
GOLIM4
GOLT1B
GORASP2
GOT1
GPANK1
GPAT3
GPATCH2
GPI
GPLD1
GPR135
GPR143
GPR171
GPR183
GPR22
GPR52
GPR89A
GPR89B
GPRASP1
GPSM3
GPX1
GPX4
GRAMD1A
GRAMD4
GRAP2
GRIN2D
GRIN3B
GRINA
GRK3
GRK5
GRWD1
GSDMB
GSTM3
GTF2H4
GTF2IRD2B
GTPBP2
GTSE1
GXYLT1
H1FX
H3F3A
H3F3B
HACD2
HACD4
HACE1
HAGHL
HAUS1
HAUS2
HAUS3
HBQ1
HCFC2
HCLS1
HDAC10
50%
Mutation Types
missense_variant
frameshift_variant
stop_gained
start_lost
multi_hit

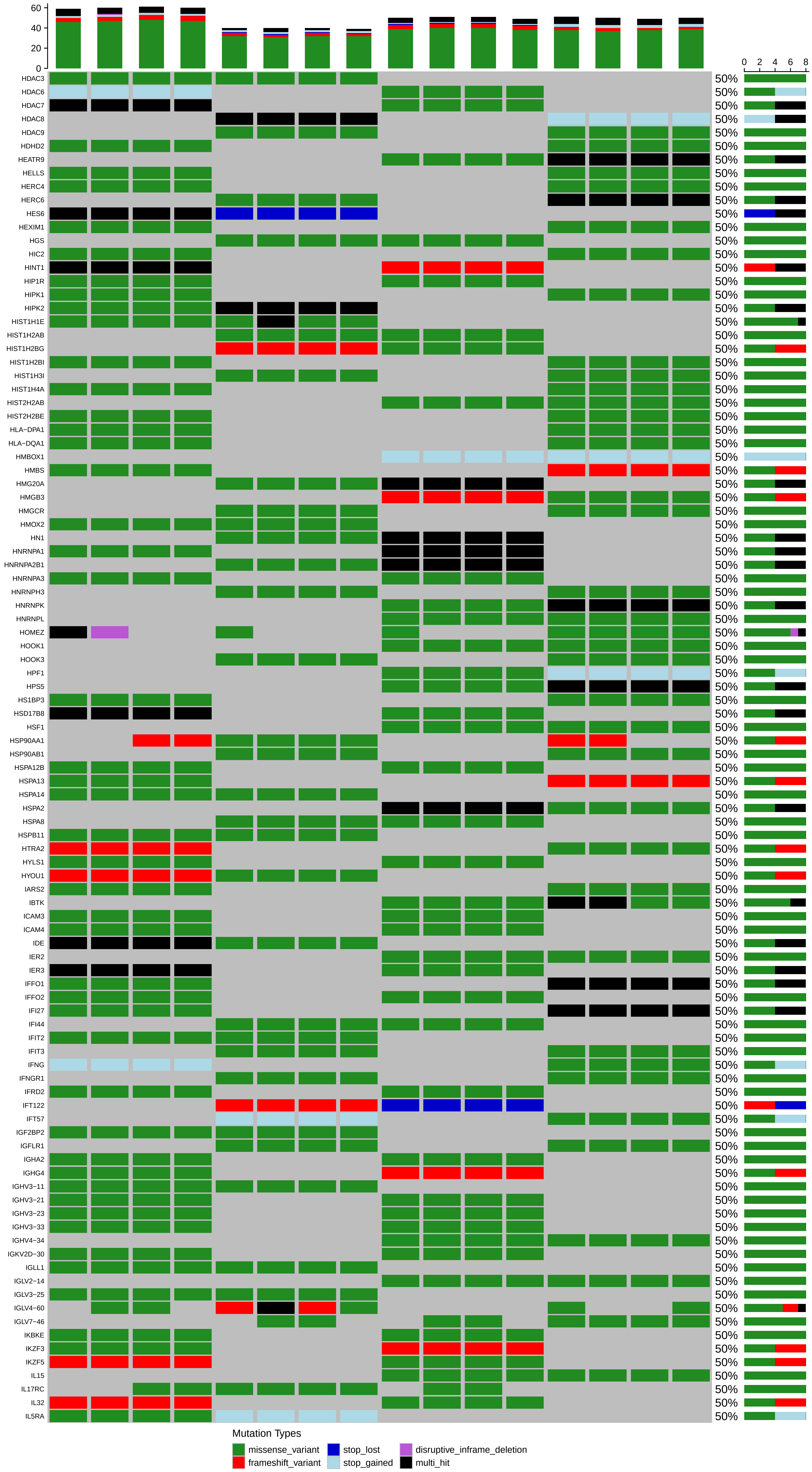

60
40
20
0
0 2 4 6 8
HDAC3
HDAC6
HDAC7
HDAC8
HDAC9
HDHD2
HEATR9
HELLS
HERC4
HERC6
HES6
HEXIM1
HGS
HIC2
HINT1
HIP1R
HIPK1
HIPK2
HIST1H1E
HIST1H2AB
HIST1H2BG
HIST1H2BI
HIST1H3I
HIST1H4A
HIST2H2AB
HIST2H2BE
HLA−DPA1
HLA−DQA1
HMBOX1
HMBS
HMG20A
HMGB3
HMGCR
HMOX2
HN1
HNRNPA1
HNRNPA2B1
HNRNPA3
HNRNPH3
HNRNPK
HNRNPL
HOMEZ
HOOK1
HOOK3
HPF1
HPS5
HS1BP3
HSD17B8
HSF1
HSP90AA1
HSP90AB1
HSPA12B
HSPA13
HSPA14
HSPA2
HSPA8
HSPB11
HTRA2
HYLS1
HYOU1
IARS2
IBTK
ICAM3
ICAM4
IDE
IER2
IER3
IFFO1
IFFO2
IFI27
IFI44
IFIT2
IFIT3
IFNG
IFNGR1
IFRD2
IFT122
IFT57
IGF2BP2
IGFLR1
IGHA2
IGHG4
IGHV3−11
IGHV3−21
IGHV3−23
IGHV3−33
IGHV4−34
IGKV2D−30
IGLL1
IGLV2−14
IGLV3−25
IGLV4−60
IGLV7−46
IKBKE
IKZF3
IKZF5
IL15
IL17RC
IL32
IL5RA
50%
Mutation Types
missense_variant
frameshift_variant
stop_lost
stop_gained
disruptive_inframe_deletion
multi_hit

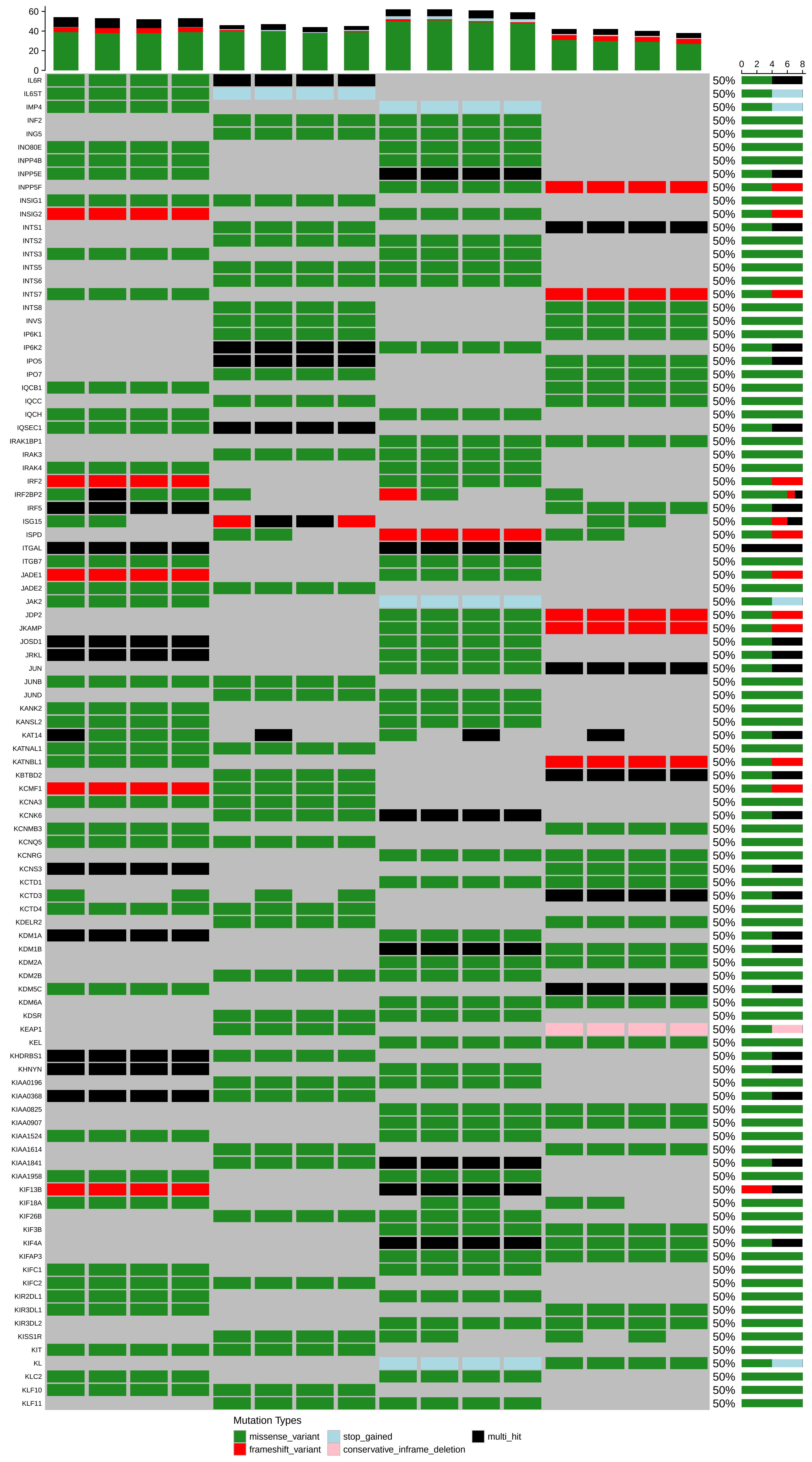

60
40
20
0
0 2 4 6 8
IL6R
IL6ST
IMP4
INF2
ING5
INO80E
INPP4B
INPP5E
INPP5F
INSIG1
INSIG2
INTS1
INTS2
INTS3
INTS5
INTS6
INTS7
INTS8
INVS
IP6K1
IP6K2
IPO5
IPO7
IQCB1
IQCC
IQCH
IQSEC1
IRAK1BP1
IRAK3
IRAK4
IRF2
IRF2BP2
IRF5
ISG15
ISPD
ITGAL
ITGB7
JADE1
JADE2
JAK2
JDP2
JKAMP
JOSD1
JRKL
JUN
JUNB
JUND
KANK2
KANSL2
KAT14
KATNAL1
KATNBL1
KBTBD2
KCMF1
KCNA3
KCNK6
KCNMB3
KCNQ5
KCNRG
KCNS3
KCTD1
KCTD3
KCTD4
KDELR2
KDM1A
KDM1B
KDM2A
KDM2B
KDM5C
KDM6A
KDSR
KEAP1
KEL
KHDRBS1
KHNYN
KIAA0196
KIAA0368
KIAA0825
KIAA0907
KIAA1524
KIAA1614
KIAA1841
KIAA1958
KIF13B
KIF18A
KIF26B
KIF3B
KIF4A
KIFAP3
KIFC1
KIFC2
KIR2DL1
KIR3DL1
KIR3DL2
KISS1R
KIT
KL
KLC2
KLF10
KLF11
50%
Mutation Types
missense_variant
frameshift_variant
stop_gained
conservative_inframe_deletion
multi_hit

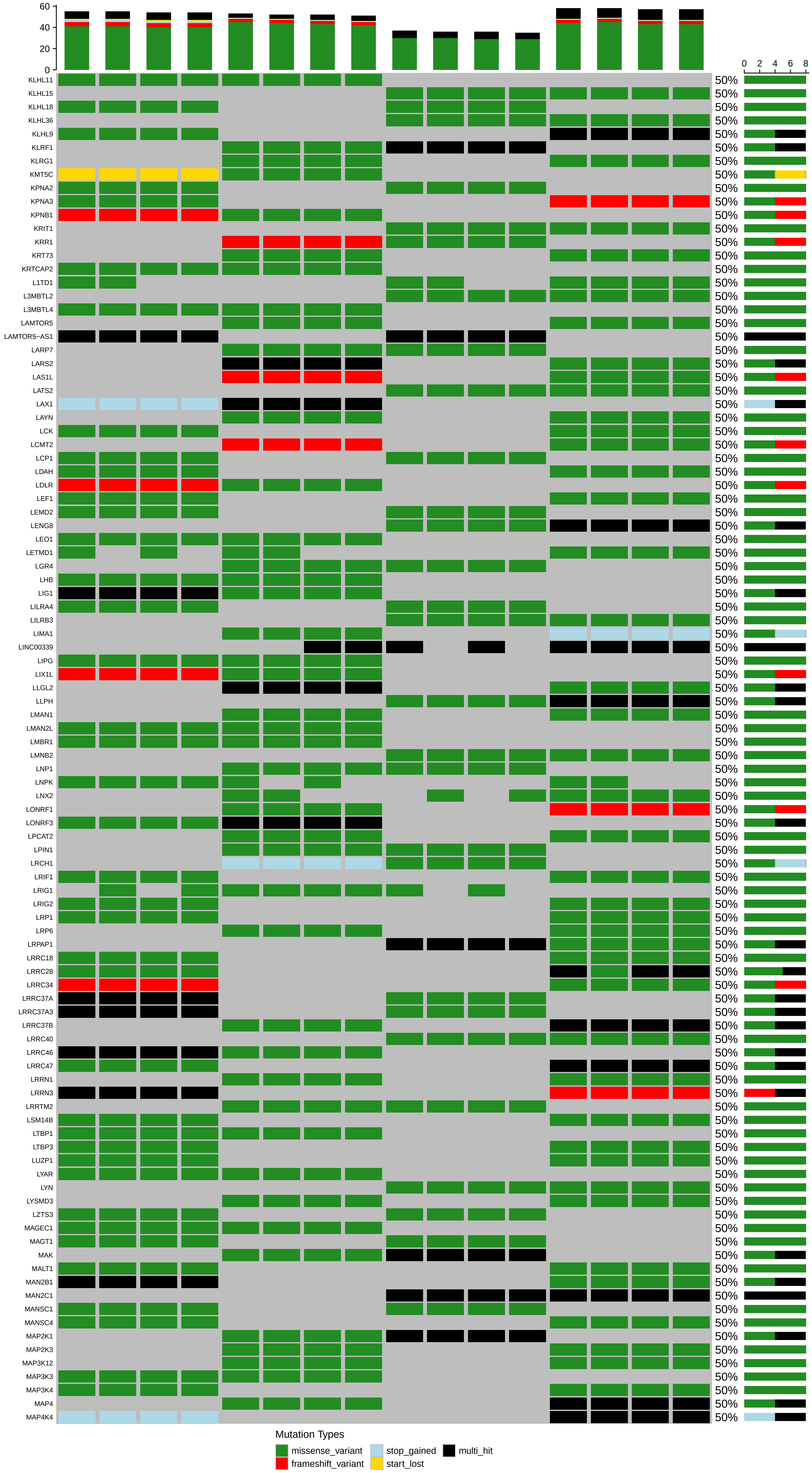
KLHL11
KLHL15
KLHL18
KLHL36
KLHL9
KLRF1
KLRG1
KMT5C
KPNA2
KPNA3
KPNB1
KRIT1
KRR1
KRT73
KRTCAP2
L1TD1
L3MBTL2
L3MBTL4
LAMTOR5
LAMTOR5−AS1
LARP7
LARS2
LAS1L
LATS2
LAX1
LAYN
LCK
LCMT2
LCP1
LDAH
LDLR
LEF1
LEMD2
LENG8
LEO1
LETMD1
LGR4
LHB
LIG1
LILRA4
LILRB3
LIMA1
LINC00339
LIPG
LIX1L
LLGL2
LLPH
LMAN1
LMAN2L
LMBR1
LMNB2
LNP1
LNPK
LNX2
LONRF1
LONRF3
LPCAT2
LPIN1
LRCH1
LRIF1
LRIG1
LRIG2
LRP1
LRP6
LRPAP1
LRRC18
LRRC28
LRRC34
LRRC37A
LRRC37A3
LRRC37B
LRRC40
LRRC46
LRRC47
LRRN1
LRRN3
LRRTM2
LSM14B
LTBP1
LTBP3
LUZP1
LYAR
LYN
LYSMD3
LZTS3
MAGEC1
MAGT1
MAK
MALT1
MAN2B1
MAN2C1
MANSC1
MANSC4
MAP2K1
MAP2K3
MAP3K12
MAP3K3
MAP3K4
MAP4
MAP4K4
50%
Mutation Types
missense_variant
frameshift_variant
stop_gained
start_lost
multi_hit

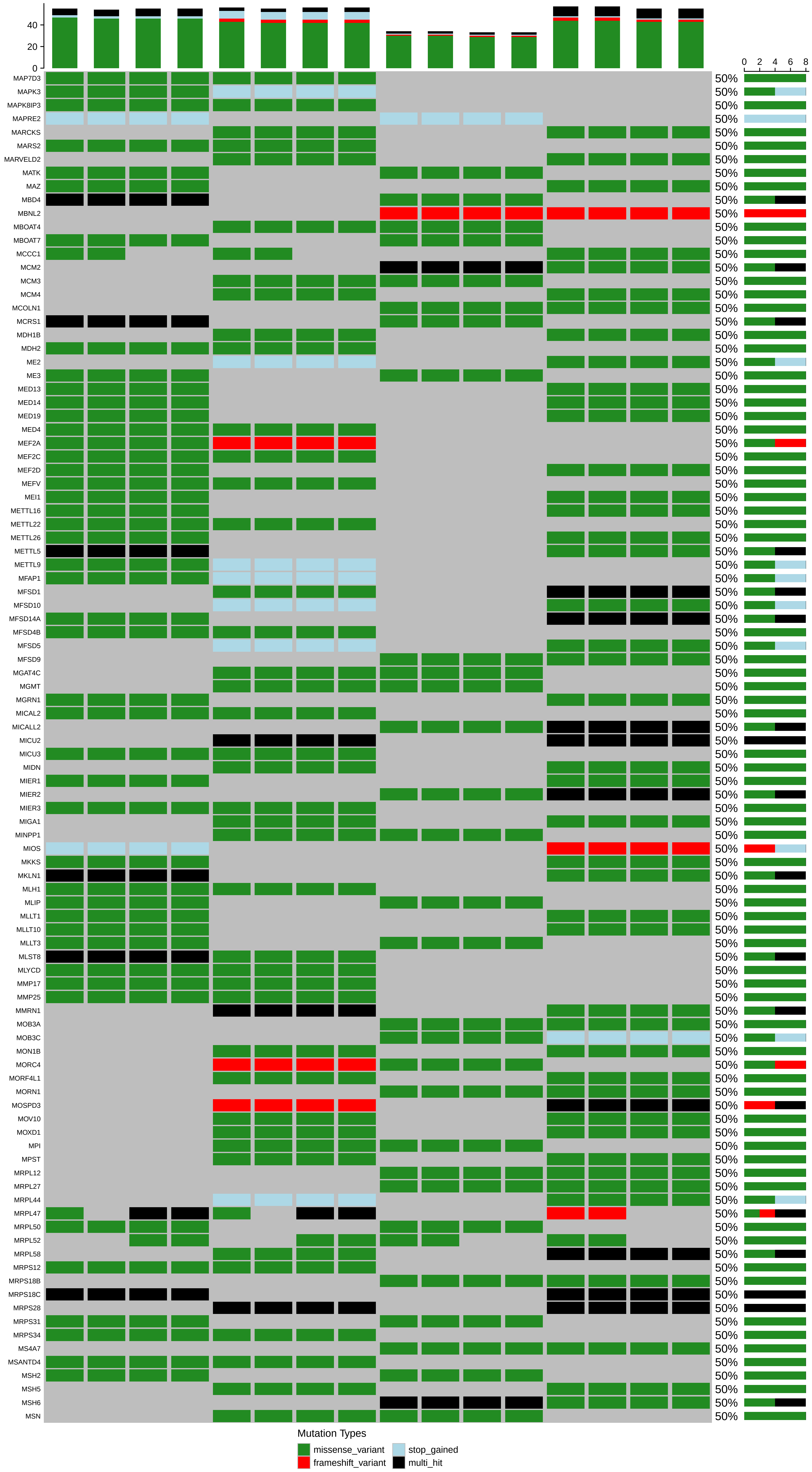
0
20
40
0 2 4 6 8
MAP7D3
MAPK3
MAPK8IP3
MAPRE2
MARCKS
MARS2
MARVELD2
MATK
MAZ
MBD4
MBNL2
MBOAT4
MBOAT7
MCCC1
MCM2
MCM3
MCM4
MCOLN1
MCRS1
MDH1B
MDH2
ME2
ME3
MED13
MED14
MED19
MED4
MEF2A
MEF2C
MEF2D
MEFV
MEI1
METTL16
METTL22
METTL26
METTL5
METTL9
MFAP1
MFSD1
MFSD10
MFSD14A
MFSD4B
MFSD5
MFSD9
MGAT4C
MGMT
MGRN1
MICAL2
MICALL2
MICU2
MICU3
MIDN
MIER1
MIER2
MIER3
MIGA1
MINPP1
MIOS
MKKS
MKLN1
MLH1
MLIP
MLLT1
MLLT10
MLLT3
MLST8
MLYCD
MMP17
MMP25
MMRN1
MOB3A
MOB3C
MON1B
MORC4
MORF4L1
MORN1
MOSPD3
MOV10
MOXD1
MPI
MPST
MRPL12
MRPL27
MRPL44
MRPL47
MRPL50
MRPL52
MRPL58
MRPS12
MRPS18B
MRPS18C
MRPS28
MRPS31
MRPS34
MS4A7
MSANTD4
MSH2
MSH5
MSH6
MSN
50%
Mutation Types
missense_variant
stop_gained
frameshift_variant
multi_hit

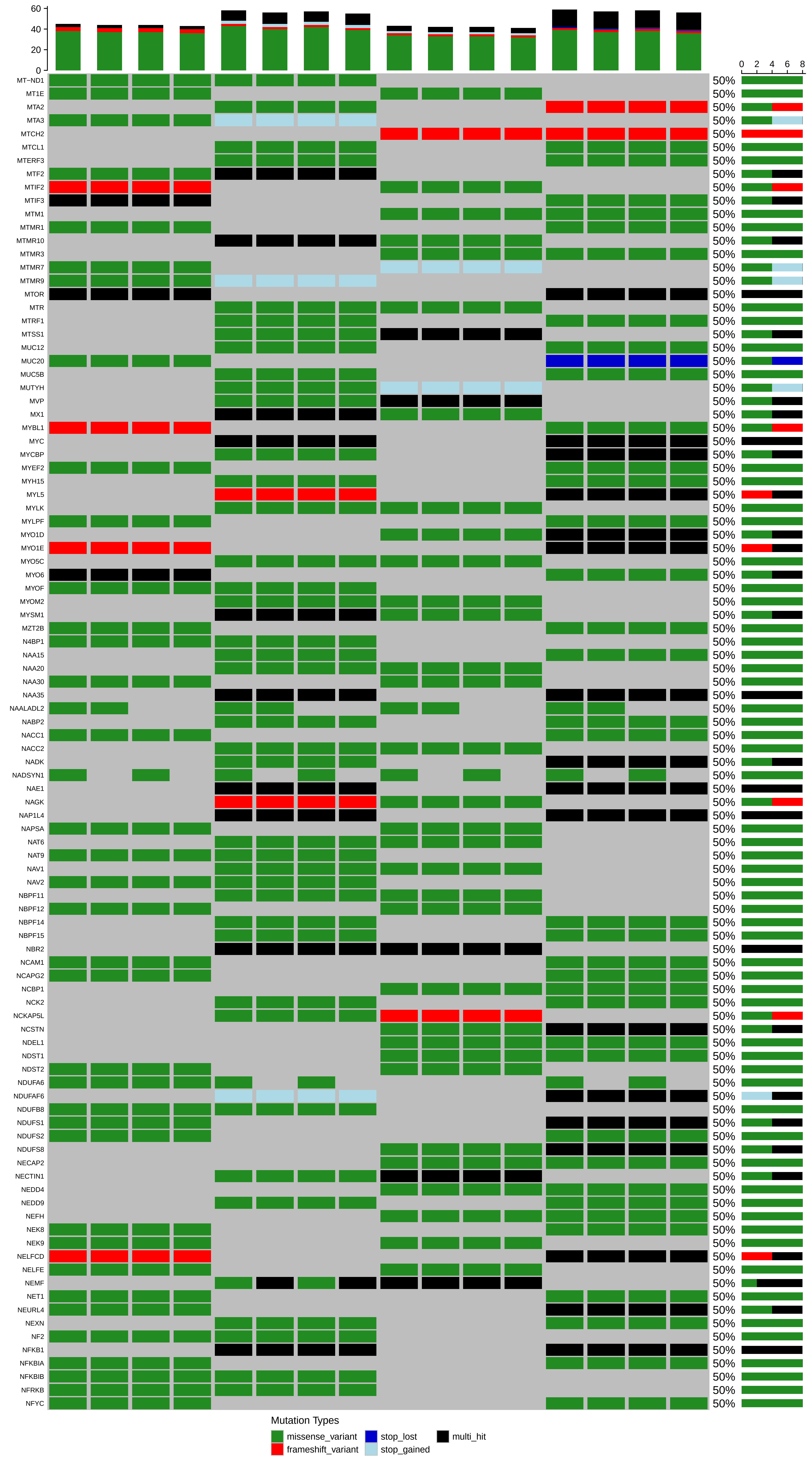

MT−ND1
MT1E
MTA2
MTA3
MTCH2
MTCL1
MTERF3
MTF2
MTIF2
MTIF3
MTM1
MTMR1
MTMR10
MTMR3
MTMR7
MTMR9
MTOR
MTR
MTRF1
MTSS1
MUC12
MUC20
MUC5B
MUTYH
MVP
MX1
MYBL1
MYC
MYCBP
MYEF2
MYH15
MYL5
MYLK
MYLPF
MYO1D
MYO1E
MYO5C
MYO6
MYOF
MYOM2
MYSM1
MZT2B
N4BP1
NAA15
NAA20
NAA30
NAA35
NAALADL2
NABP2
NACC1
NACC2
NADK
NADSYN1
NAE1
NAGK
NAP1L4
NAPSA
NAT6
NAT9
NAV1
NAV2
NBPF11
NBPF12
NBPF14
NBPF15
NBR2
NCAM1
NCAPG2
NCBP1
NCK2
NCKAP5L
NCSTN
NDEL1
NDST1
NDST2
NDUFA6
NDUFAF6
NDUFB8
NDUFS1
NDUFS2
NDUFS8
NECAP2
NECTIN1
NEDD4
NEDD9
NEFH
NEK8
NEK9
NELFCD
NELFE
NEMF
NET1
NEURL4
NEXN
NF2
NFKB1
NFKBIA
NFKBIB
NFRKB
NFYC
Mutation Types
missense_variant
frameshift_variant
stop_lost
stop_gained
multi_hit

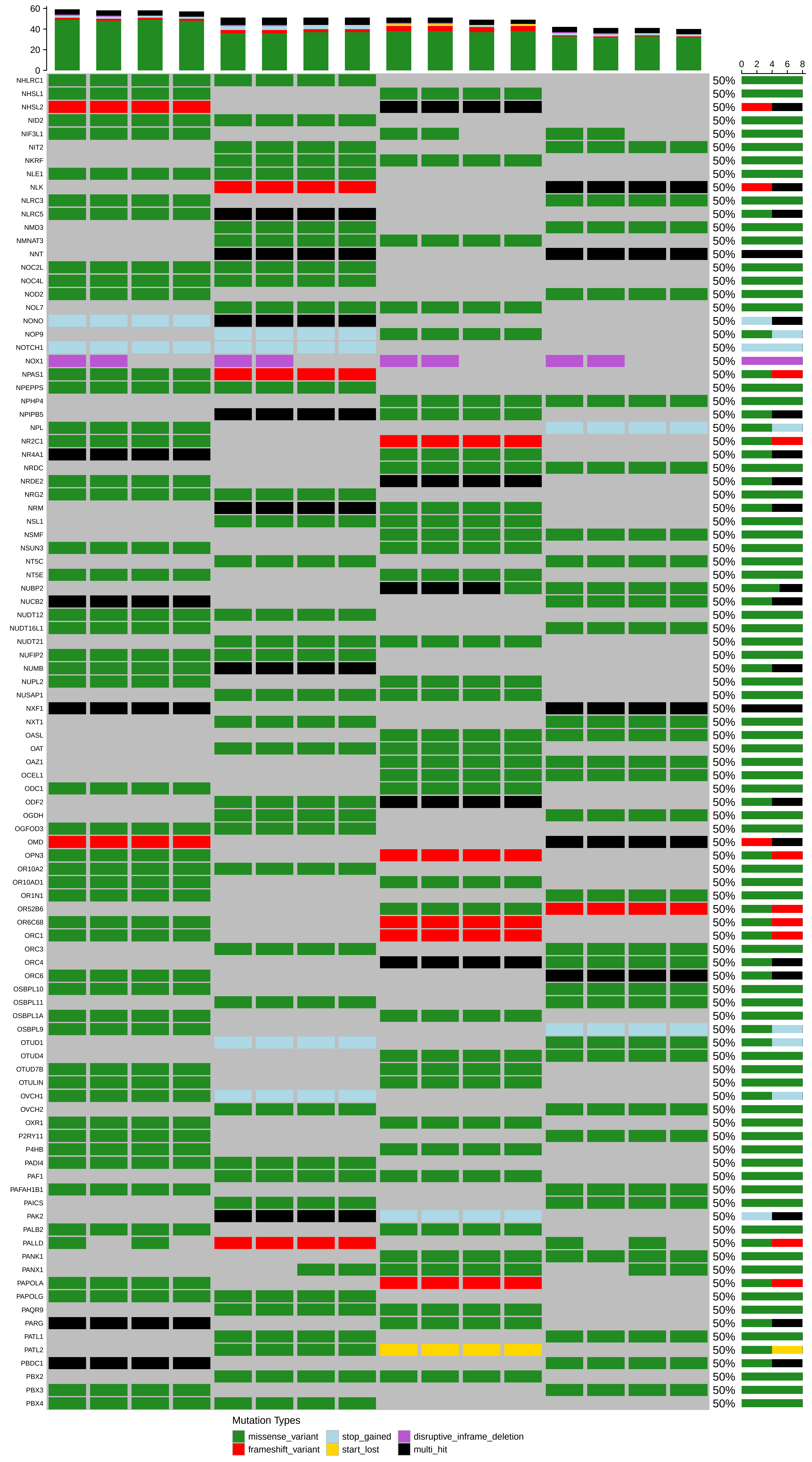

60
40
20
0
0 2 4 6 8
NHLRC1
NHSL1
NHSL2
NID2
NIF3L1
NIT2
NKRF
NLE1
NLK
NLRC3
NLRC5
NMD3
NMNAT3
NNT
NOC2L
NOC4L
NOD2
NOL7
NONO
NOP9
NOTCH1
NOX1
NPAS1
NPEPPS
NPHP4
NPIPB5
NPL
NR2C1
NR4A1
NRDC
NRDE2
NRG2
NRM
NSL1
NSMF
NSUN3
NT5C
NT5E
NUBP2
NUCB2
NUDT12
NUDT16L1
NUDT21
NUFIP2
NUMB
NUPL2
NUSAP1
NXF1
NXT1
OASL
OAT
OAZ1
OCEL1
ODC1
ODF2
OGDH
OGFOD3
OMD
OPN3
OR10A2
OR10AD1
OR1N1
OR52B6
OR6C68
ORC1
ORC3
ORC4
ORC6
OSBPL10
OSBPL11
OSBPL1A
OSBPL9
OTUD1
OTUD4
OTUD7B
OTULIN
OVCH1
OVCH2
OXR1
P2RY11
P4HB
PADI4
PAF1
PAFAH1B1
PAICS
PAK2
PALB2
PALLD
PANK1
PANX1
PAPOLA
PAPOLG
PAQR9
PARG
PATL1
PATL2
PBDC1
PBX2
PBX3
PBX4
50%
Mutation Types
missense_variant
frameshift_variant
stop_gained
start_lost
disruptive_inframe_deletion
multi_hit

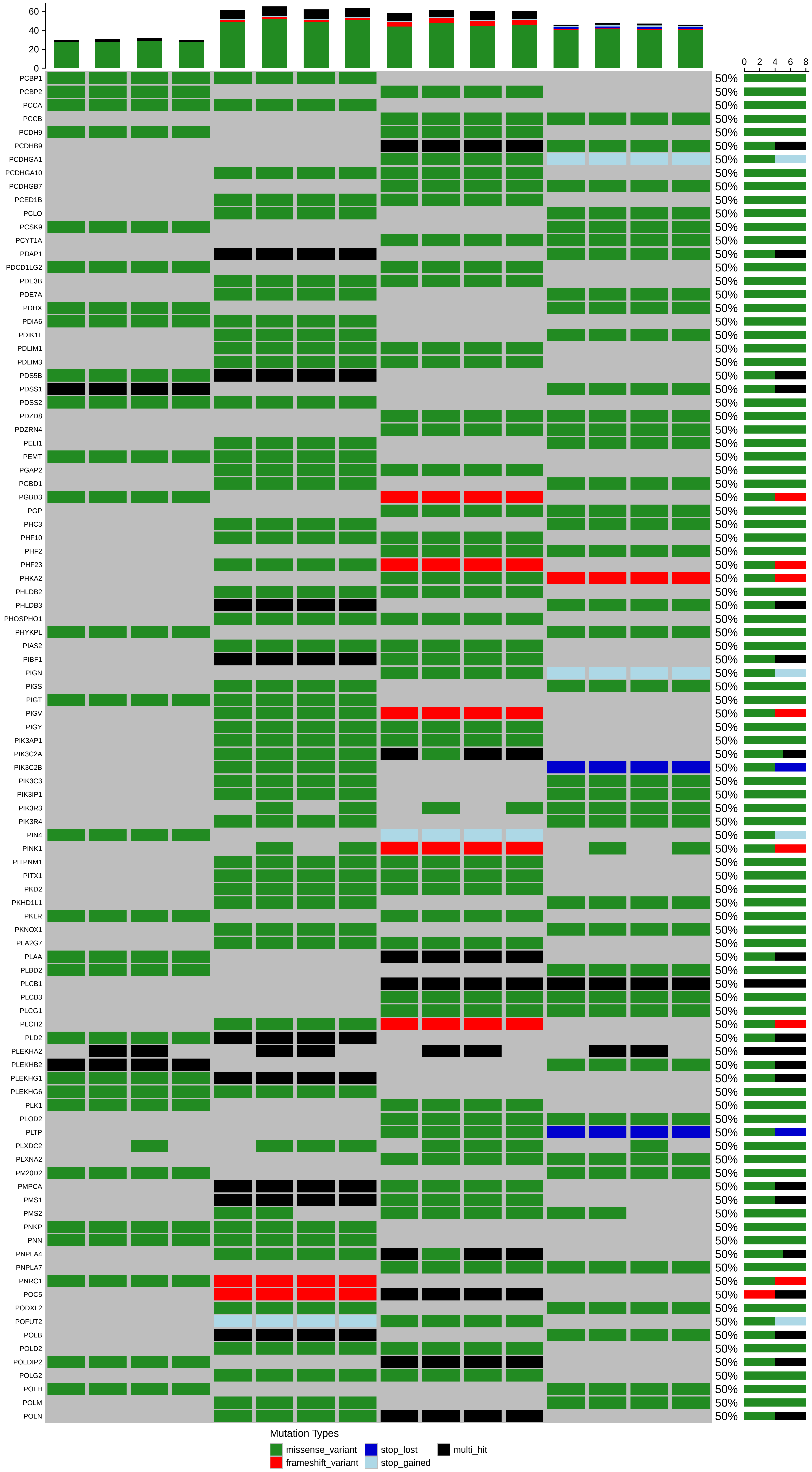

0
20
40
60
0 2 4 6 8
PCBP1
PCBP2
PCCA
PCCB
PCDH9
PCDHB9
PCDHGA1
PCDHGA10
PCDHGB7
PCED1B
PCLO
PCSK9
PCYT1A
PDAP1
PDCD1LG2
PDE3B
PDE7A
PDHX
PDIA6
PDIK1L
PDLIM1
PDLIM3
PDS5B
PDSS1
PDSS2
PDZD8
PDZRN4
PELI1
PEMT
PGAP2
PGBD1
PGBD3
PGP
PHC3
PHF10
PHF2
PHF23
PHKA2
PHLDB2
PHLDB3
PHOSPHO1
PHYKPL
PIAS2
PIBF1
PIGN
PIGS
PIGT
PIGV
PIGY
PIK3AP1
PIK3C2A
PIK3C2B
PIK3C3
PIK3IP1
PIK3R3
PIK3R4
PIN4
PINK1
PITPNM1
PITX1
PKD2
PKHD1L1
PKLR
PKNOX1
PLA2G7
PLAA
PLBD2
PLCB1
PLCB3
PLCG1
PLCH2
PLD2
PLEKHA2
PLEKHB2
PLEKHG1
PLEKHG6
PLK1
PLOD2
PLTP
PLXDC2
PLXNA2
PM20D2
PMPCA
PMS1
PMS2
PNKP
PNN
PNPLA4
PNPLA7
PNRC1
POC5
PODXL2
POFUT2
POLB
POLD2
POLDIP2
POLG2
POLH
POLM
POLN
50%
Mutation Types
missense_variant
frameshift_variant
stop_lost
stop_gained
multi_hit

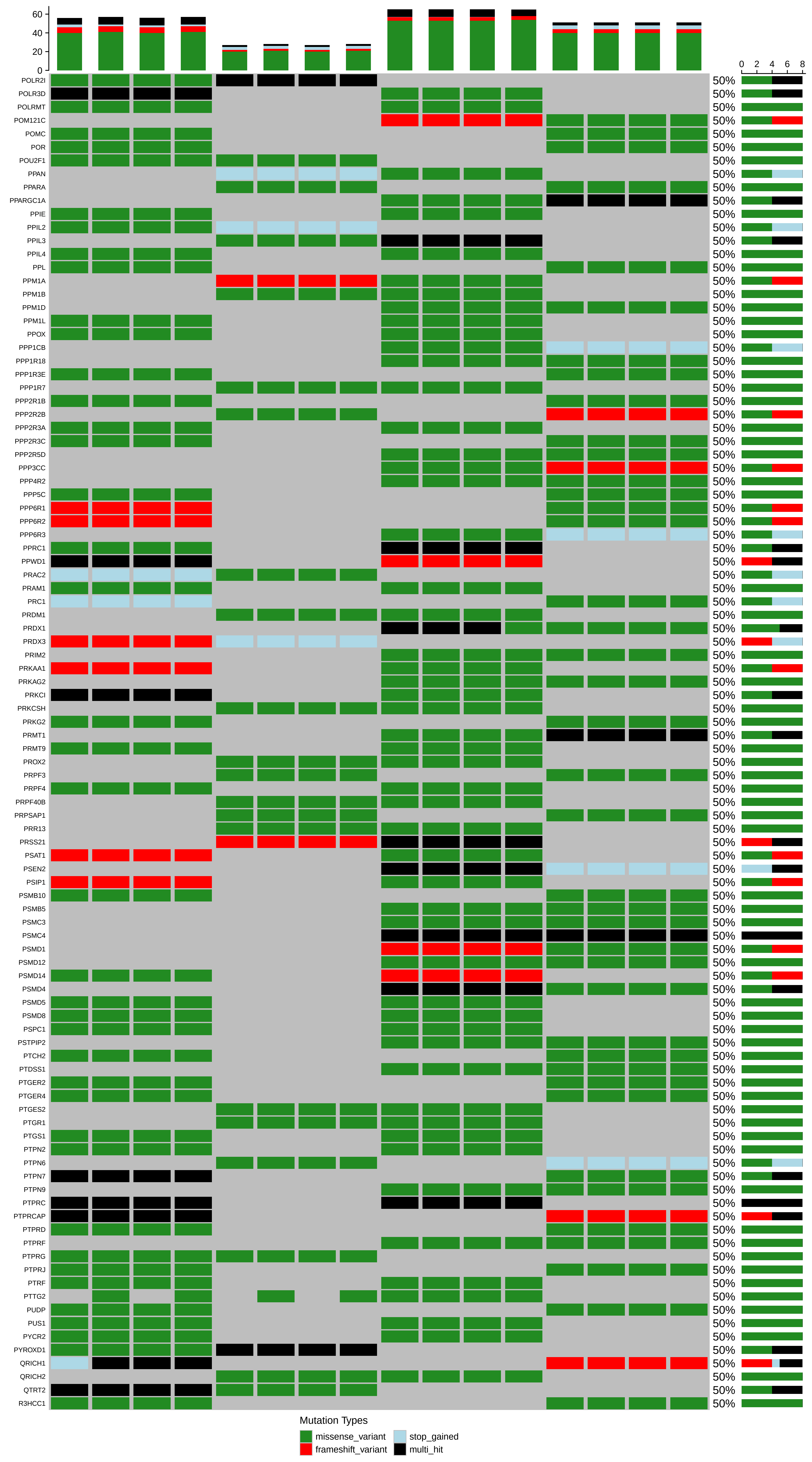

60
40
20
0
0 2 4 6 8
POLR2I
POLR3D
POLRMT
POM121C
POMC
POR
POU2F1
PPAN
PPARA
PPARGC1A
PPIE
PPIL2
PPIL3
PPIL4
PPL
PPM1A
PPM1B
PPM1D
PPM1L
PPOX
PPP1CB
PPP1R18
PPP1R3E
PPP1R7
PPP2R1B
PPP2R2B
PPP2R3A
PPP2R3C
PPP2R5D
PPP3CC
PPP4R2
PPP5C
PPP6R1
PPP6R2
PPP6R3
PPRC1
PPWD1
PRAC2
PRAM1
PRC1
PRDM1
PRDX1
PRDX3
PRIM2
PRKAA1
PRKAG2
PRKCI
PRKCSH
PRKG2
PRMT1
PRMT9
PROX2
PRPF3
PRPF4
PRPF40B
PRPSAP1
PRR13
PRSS21
PSAT1
PSEN2
PSIP1
PSMB10
PSMB5
PSMC3
PSMC4
PSMD1
PSMD12
PSMD14
PSMD4
PSMD5
PSMD8
PSPC1
PSTPIP2
PTCH2
PTDSS1
PTGER2
PTGER4
PTGES2
PTGR1
PTGS1
PTPN2
PTPN6
PTPN7
PTPN9
PTPRC
PTPRCAP
PTPRD
PTPRF
PTPRG
PTPRJ
PTRF
PTTG2
PUDP
PUS1
PYCR2
PYROXD1
QRICH1
QRICH2
QTRT2
R3HCC1
50% (each row)
Mutation Types
missense_variant
frameshift_variant
stop_gained
multi_hit

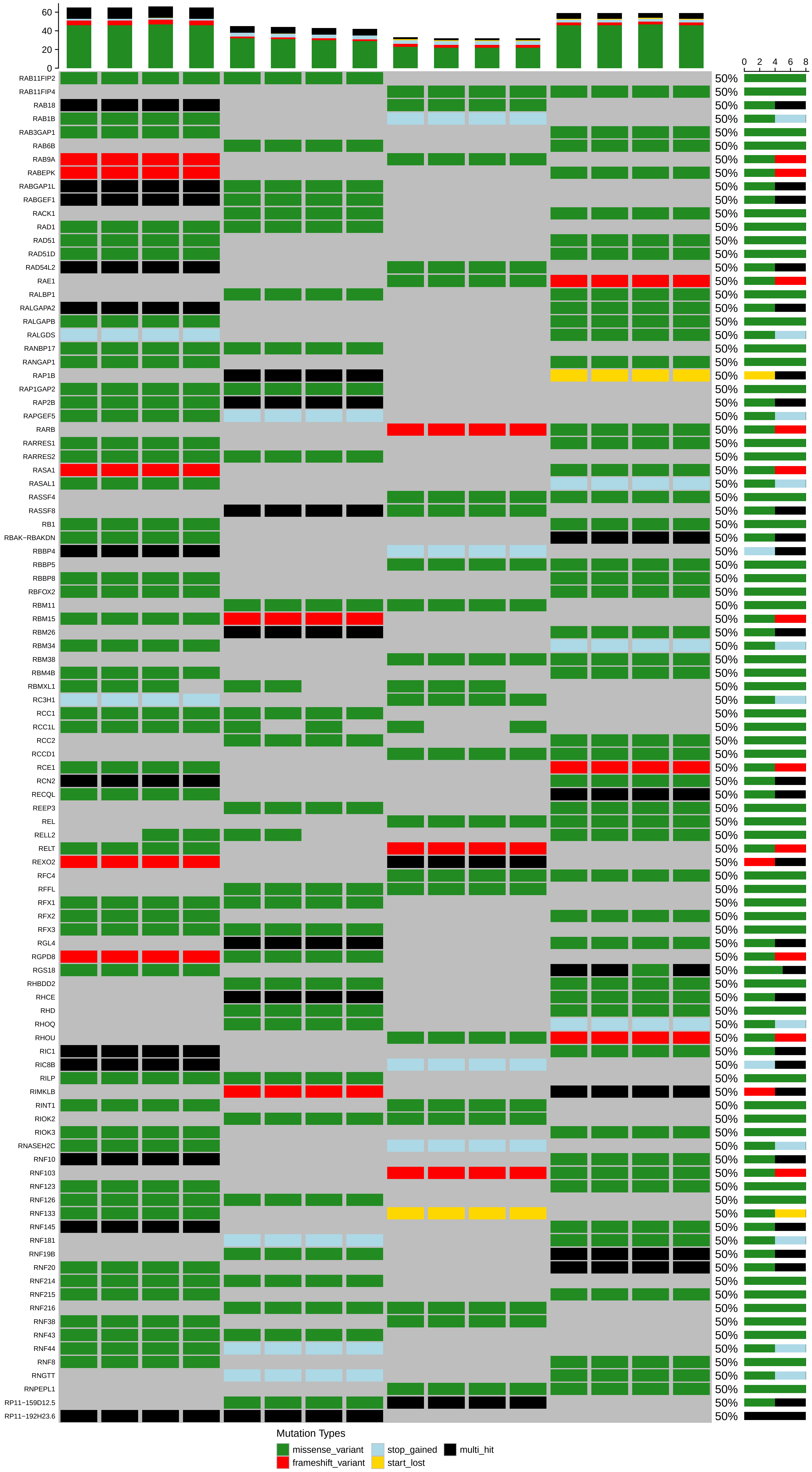

60
40
20
0
0 2 4 6 8
RAB11FIP2
RAB11FIP4
RAB18
RAB1B
RAB3GAP1
RAB6B
RAB9A
RABEPK
RABGAP1L
RABGEF1
RACK1
RAD1
RAD51
RAD51D
RAD54L2
RAE1
RALBP1
RALGAPA2
RALGAPB
RALGDS
RANBP17
RANGAP1
RAP1B
RAP1GAP2
RAP2B
RAPGEF5
RARB
RARRES1
RARRES2
RASA1
RASAL1
RASSF4
RASSF8
RB1
RBAK−RBAKDN
RBBP4
RBBP5
RBBP8
RBFOX2
RBM11
RBM15
RBM26
RBM34
RBM38
RBM4B
RBMXL1
RC3H1
RCC1
RCC1L
RCC2
RCCD1
RCE1
RCN2
RECQL
REEP3
REL
RELL2
RELT
REXO2
RFC4
RFFL
RFX1
RFX2
RFX3
RGL4
RGPD8
RGS18
RHBDD2
RHCE
RHD
RHOQ
RHOU
RIC1
RIC8B
RILP
RIMKLB
RINT1
RIOK2
RIOK3
RNASEH2C
RNF10
RNF103
RNF123
RNF126
RNF133
RNF145
RNF181
RNF19B
RNF20
RNF214
RNF215
RNF216
RNF38
RNF43
RNF44
RNF8
RNGTT
RNPEPL1
RP11−159D12.5
RP11−192H23.6
50%
Mutation Types
missense_variant
frameshift_variant
stop_gained
start_lost
multi_hit

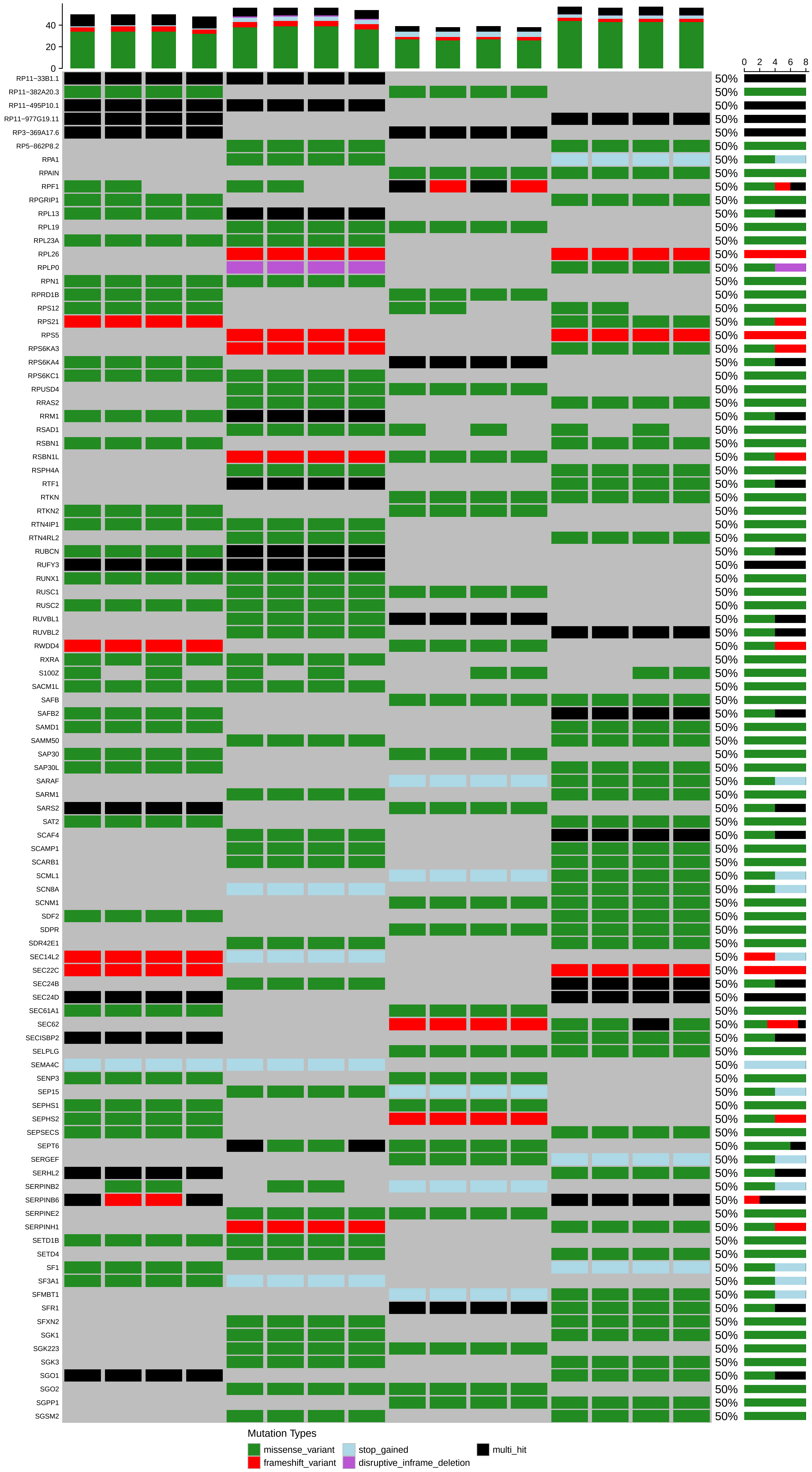

0
20
40
0 2 4 6 8
RP11−33B1.1
RP11−382A20.3
RP11−495P10.1
RP11−977G19.11
RP3−369A17.6
RP5−862P8.2
RPA1
RPAIN
RPF1
RPGRIP1
RPL13
RPL19
RPL23A
RPL26
RPLP0
RPN1
RPRD1B
RPS12
RPS21
RPS5
RPS6KA3
RPS6KA4
RPS6KC1
RPUSD4
RRAS2
RRM1
RSAD1
RSBN1
RSBN1L
RSPH4A
RTF1
RTKN
RTKN2
RTN4IP1
RTN4RL2
RUBCN
RUFY3
RUNX1
RUSC1
RUSC2
RUVBL1
RUVBL2
RWDD4
RXRA
S100Z
SACM1L
SAFB
SAFB2
SAMD1
SAMM50
SAP30
SAP30L
SARAF
SARM1
SARS2
SAT2
SCAF4
SCAMP1
SCARB1
SCML1
SCN8A
SCNM1
SDF2
SDPR
SDR42E1
SEC14L2
SEC22C
SEC24B
SEC24D
SEC61A1
SEC62
SECISBP2
SELPLG
SEMA4C
SENP3
SEP15
SEPHS1
SEPHS2
SEPSECS
SEPT6
SERGEF
SERHL2
SERPINB2
SERPINB6
SERPINE2
SERPINH1
SETD1B
SETD4
SF1
SF3A1
SFMBT1
SFR1
SFXN2
SGK1
SGK223
SGK3
SGO1
SGO2
SGPP1
SGSM2
50%
Mutation Types
missense_variant
frameshift_variant
stop_gained
disruptive_inframe_deletion
multi_hit

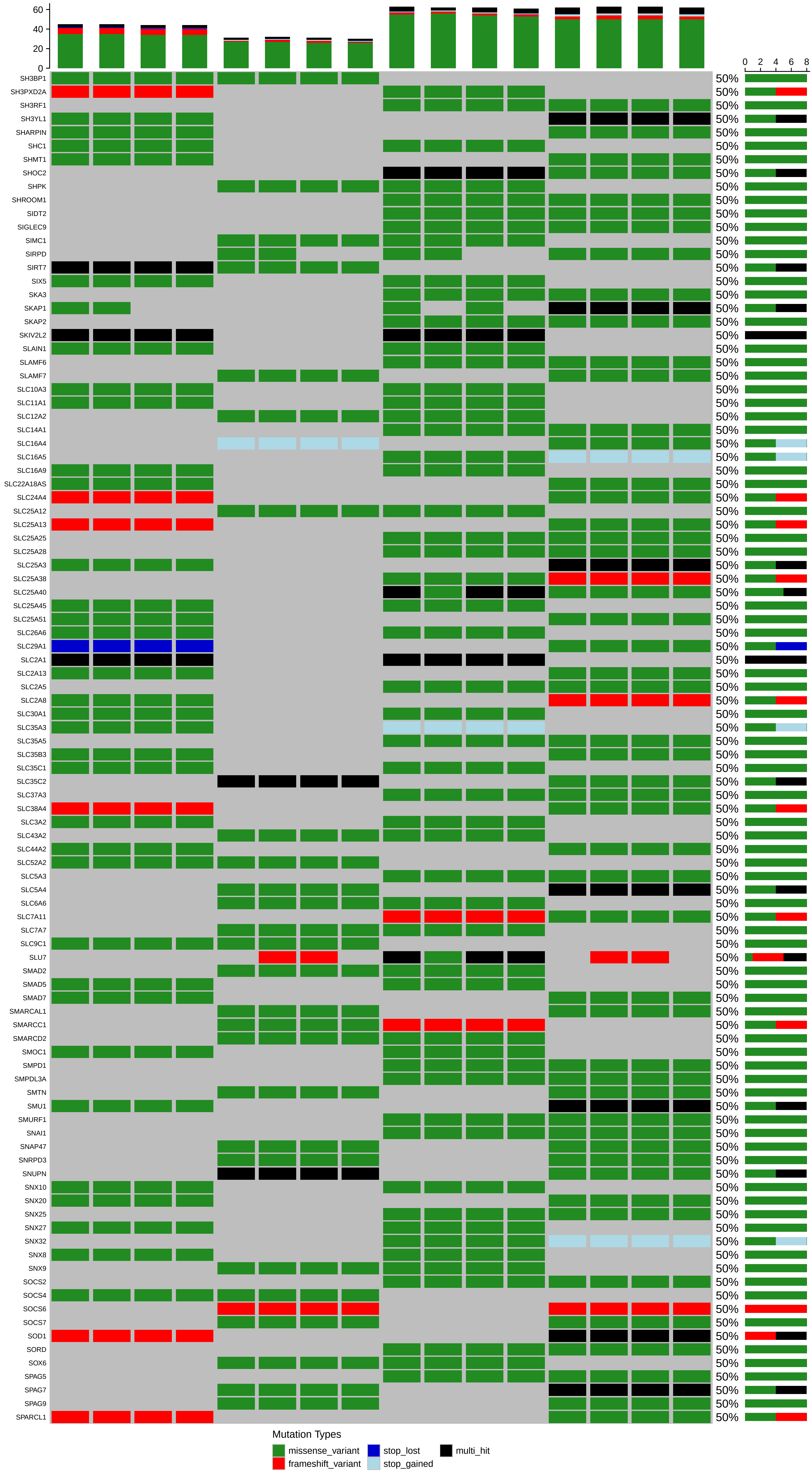

60
40
20
0
0 2 4 6 8
SH3BP1
SH3PXD2A
SH3RF1
SH3YL1
SHARPIN
SHC1
SHMT1
SHOC2
SHPK
SHROOM1
SIDT2
SIGLEC9
SIMC1
SIRPD
SIRT7
SIX5
SKA3
SKAP1
SKAP2
SKIV2L2
SLAIN1
SLAMF6
SLAMF7
SLC10A3
SLC11A1
SLC12A2
SLC14A1
SLC16A4
SLC16A5
SLC16A9
SLC22A18AS
SLC24A4
SLC25A12
SLC25A13
SLC25A25
SLC25A28
SLC25A3
SLC25A38
SLC25A40
SLC25A45
SLC25A51
SLC26A6
SLC29A1
SLC2A1
SLC2A13
SLC2A5
SLC2A8
SLC30A1
SLC35A3
SLC35A5
SLC35B3
SLC35C1
SLC35C2
SLC37A3
SLC38A4
SLC3A2
SLC43A2
SLC44A2
SLC52A2
SLC5A3
SLC5A4
SLC6A6
SLC7A11
SLC7A7
SLC9C1
SLU7
SMAD2
SMAD5
SMAD7
SMARCAL1
SMARCC1
SMARCD2
SMOC1
SMPD1
SMPDL3A
SMTN
SMU1
SMURF1
SNAI1
SNAP47
SNRPD3
SNUPN
SNX10
SNX20
SNX25
SNX27
SNX32
SNX8
SNX9
SOCS2
SOCS4
SOCS6
SOCS7
SOD1
SORD
SOX6
SPAG5
SPAG7
SPAG9
SPARCL1
50%
Mutation Types
missense_variant
frameshift_variant
stop_lost
stop_gained
multi_hit

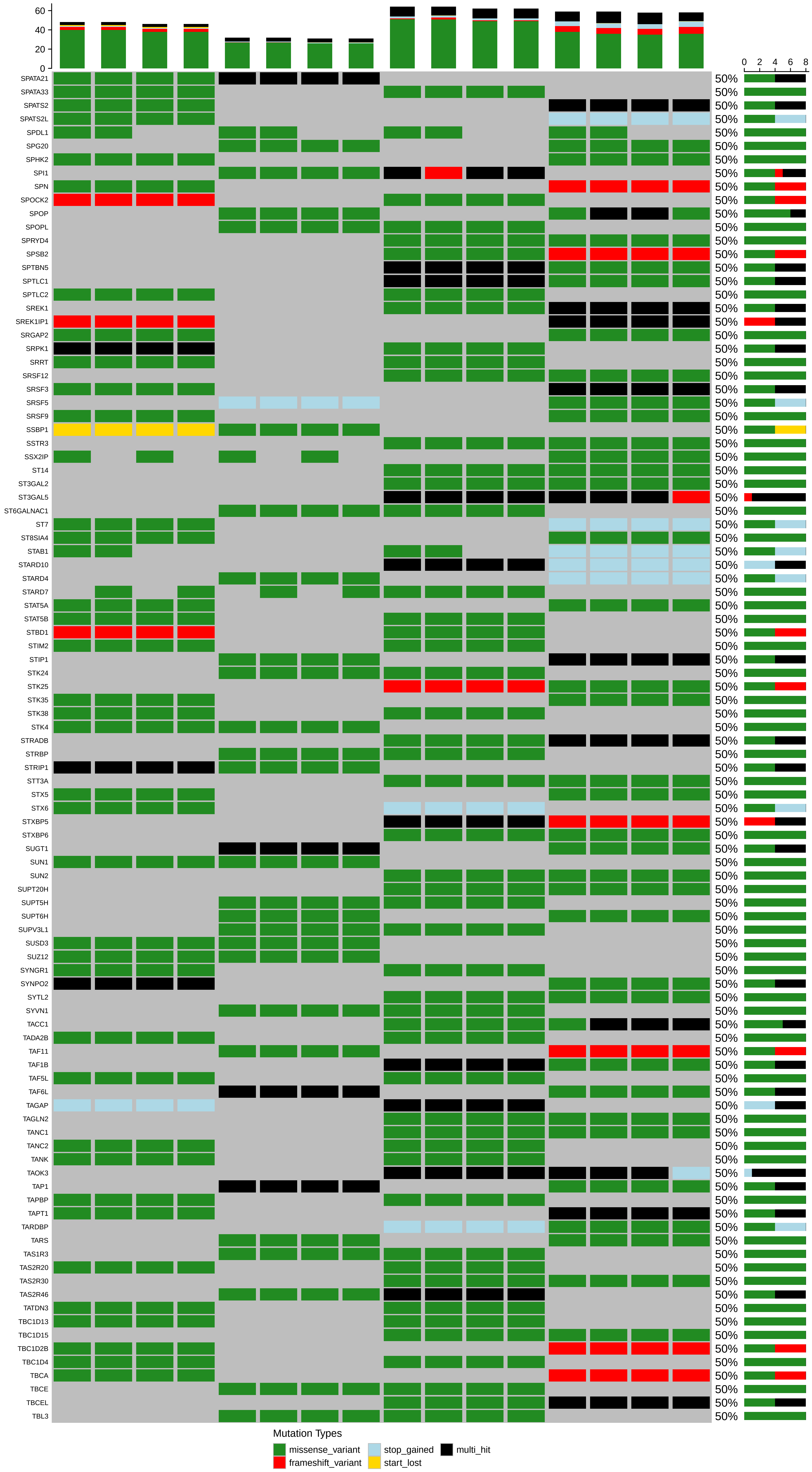

0
20
40
60
0 2 4 6 8
SPATA21
SPATA33
SPATS2
SPATS2L
SPDL1
SPG20
SPHK2
SPI1
SPN
SPOCK2
SPOP
SPOPL
SPRYD4
SPSB2
SPTBN5
SPTLC1
SPTLC2
SREK1
SREK1IP1
SRGAP2
SRPK1
SRRT
SRSF12
SRSF3
SRSF5
SRSF9
SSBP1
SSTR3
SSX2IP
ST14
ST3GAL2
ST3GAL5
ST6GALNAC1
ST7
ST8SIA4
STAB1
STARD10
STARD4
STARD7
STAT5A
STAT5B
STBD1
STIM2
STIP1
STK24
STK25
STK35
STK38
STK4
STRADB
STRBP
STRIP1
STT3A
STX5
STX6
STXBP5
STXBP6
SUGT1
SUN1
SUN2
SUPT20H
SUPT5H
SUPT6H
SUPV3L1
SUSD3
SUZ12
SYNGR1
SYNPO2
SYTL2
SYVN1
TACC1
TADA2B
TAF11
TAF1B
TAF5L
TAF6L
TAGAP
TAGLN2
TANC1
TANC2
TANK
TAOK3
TAP1
TAPBP
TAPT1
TARDBP
TARS
TAS1R3
TAS2R20
TAS2R30
TAS2R46
TATDN3
TBC1D13
TBC1D15
TBC1D2B
TBC1D4
TBCA
TBCE
TBCEL
TBL3
50%
Mutation Types
missense_variant
frameshift_variant
stop_gained
start_lost
multi_hit

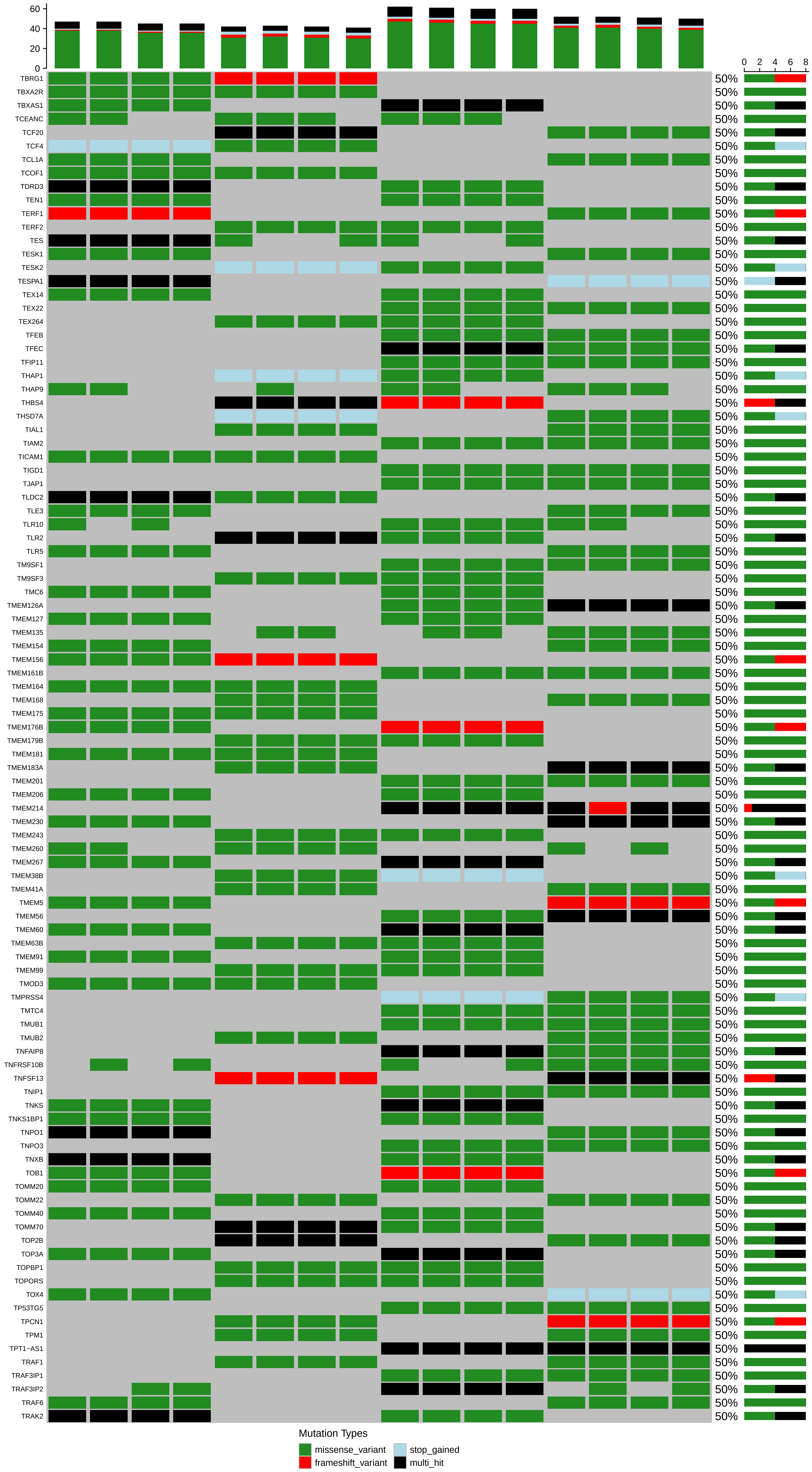

0
20
40
60
0 2 4 6 8
TBRG1
TBXA2R
TBXAS1
TCEANC
TCF20
TCF4
TCL1A
TCOF1
TDRD3
TEN1
TERF1
TERF2
TES
TESK1
TESK2
TESPA1
TEX14
TEX22
TEX264
TFEB
TFEC
TFIP11
THAP1
THAP9
THBS4
THSD7A
TIAL1
TIAM2
TICAM1
TIGD1
TJAP1
TLDC2
TLE3
TLR10
TLR2
TLR5
TM9SF1
TM9SF3
TMC6
TMEM126A
TMEM127
TMEM135
TMEM154
TMEM156
TMEM161B
TMEM164
TMEM168
TMEM175
TMEM176B
TMEM179B
TMEM181
TMEM183A
TMEM201
TMEM206
TMEM214
TMEM230
TMEM243
TMEM260
TMEM267
TMEM38B
TMEM41A
TMEM5
TMEM56
TMEM60
TMEM63B
TMEM91
TMEM99
TMOD3
TMPRSS4
TMTC4
TMUB1
TMUB2
TNFAIP8
TNFRSF10B
TNFSF13
TNIP1
TNKS
TNKS1BP1
TNPO1
TNPO3
TNXB
TOB1
TOMM20
TOMM22
TOMM40
TOMM70
TOP2B
TOP3A
TOPBP1
TOPORS
TOX4
TP53TG5
TPCN1
TPM1
TPT1−AS1
TRAF1
TRAF3IP1
TRAF3IP2
TRAF6
TRAK2
50%
Mutation Types
missense_variant
frameshift_variant
stop_gained
multi_hit

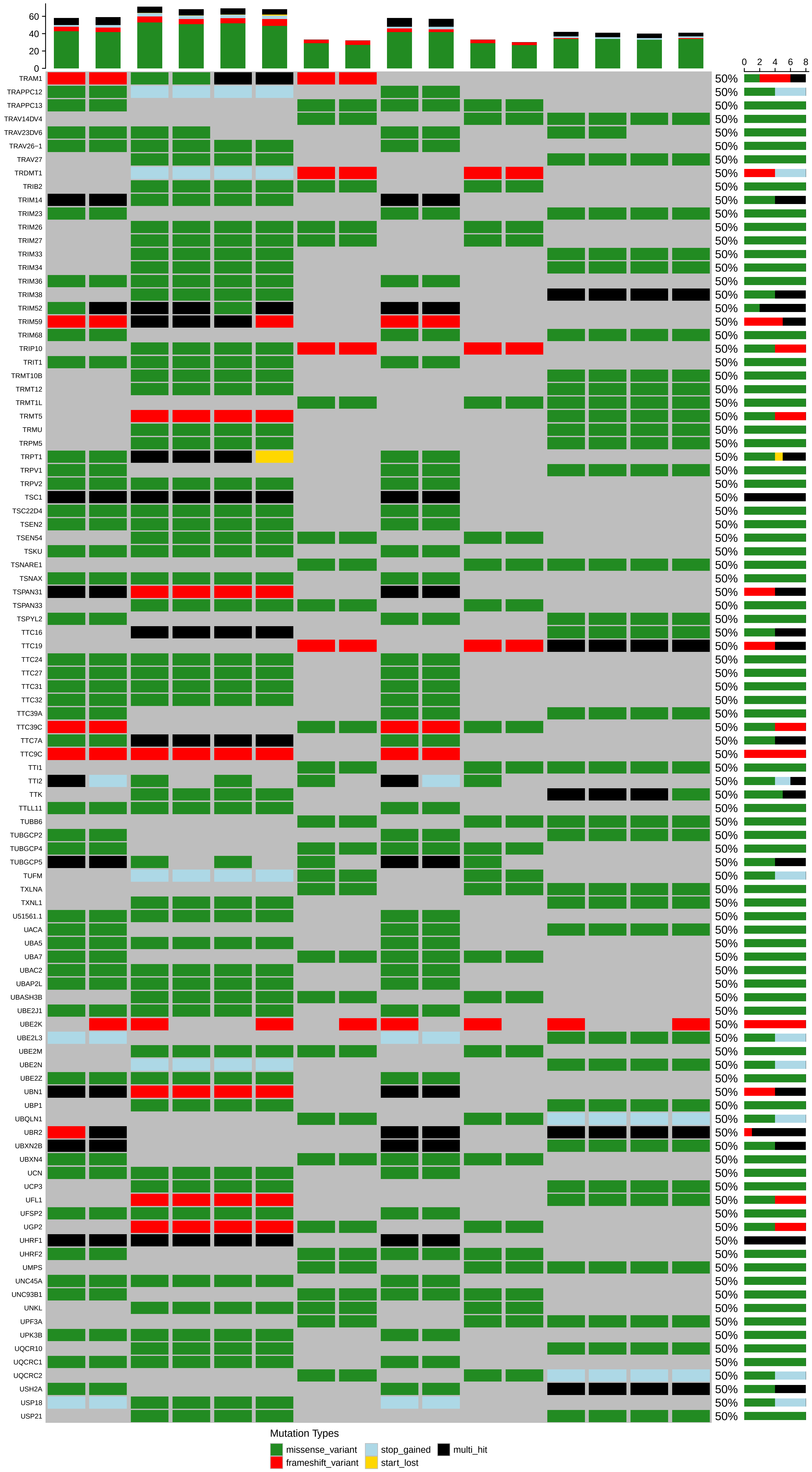

60
40
20
0
0 2 4 6 8
TRAM1
TRAPPC12
TRAPPC13
TRAV14DV4
TRAV23DV6
TRAV26−1
TRAV27
TRDMT1
TRIB2
TRIM14
TRIM23
TRIM26
TRIM27
TRIM33
TRIM34
TRIM36
TRIM38
TRIM52
TRIM59
TRIM68
TRIP10
TRIT1
TRMT10B
TRMT12
TRMT1L
TRMT5
TRMU
TRPM5
TRPT1
TRPV1
TRPV2
TSC1
TSC22D4
TSEN2
TSEN54
TSKU
TSNARE1
TSNAX
TSPAN31
TSPAN33
TSPYL2
TTC16
TTC19
TTC24
TTC27
TTC31
TTC32
TTC39A
TTC39C
TTC7A
TTC9C
TTI1
TTI2
TTK
TTLL11
TUBB6
TUBGCP2
TUBGCP4
TUBGCP5
TUFM
TXLNA
TXNL1
U51561.1
UACA
UBA5
UBA7
UBAC2
UBAP2L
UBASH3B
UBE2J1
UBE2K
UBE2L3
UBE2M
UBE2N
UBE2Z
UBN1
UBP1
UBQLN1
UBR2
UBXN2B
UBXN4
UCN
UCP3
UFL1
UFSP2
UGP2
UHRF1
UHRF2
UMPS
UNC45A
UNC93B1
UNKL
UPF3A
UPK3B
UQCR10
UQCRC1
UQCRC2
USH2A
USP18
USP21
50%
Mutation Types
missense_variant
frameshift_variant
stop_gained
start_lost
multi_hit

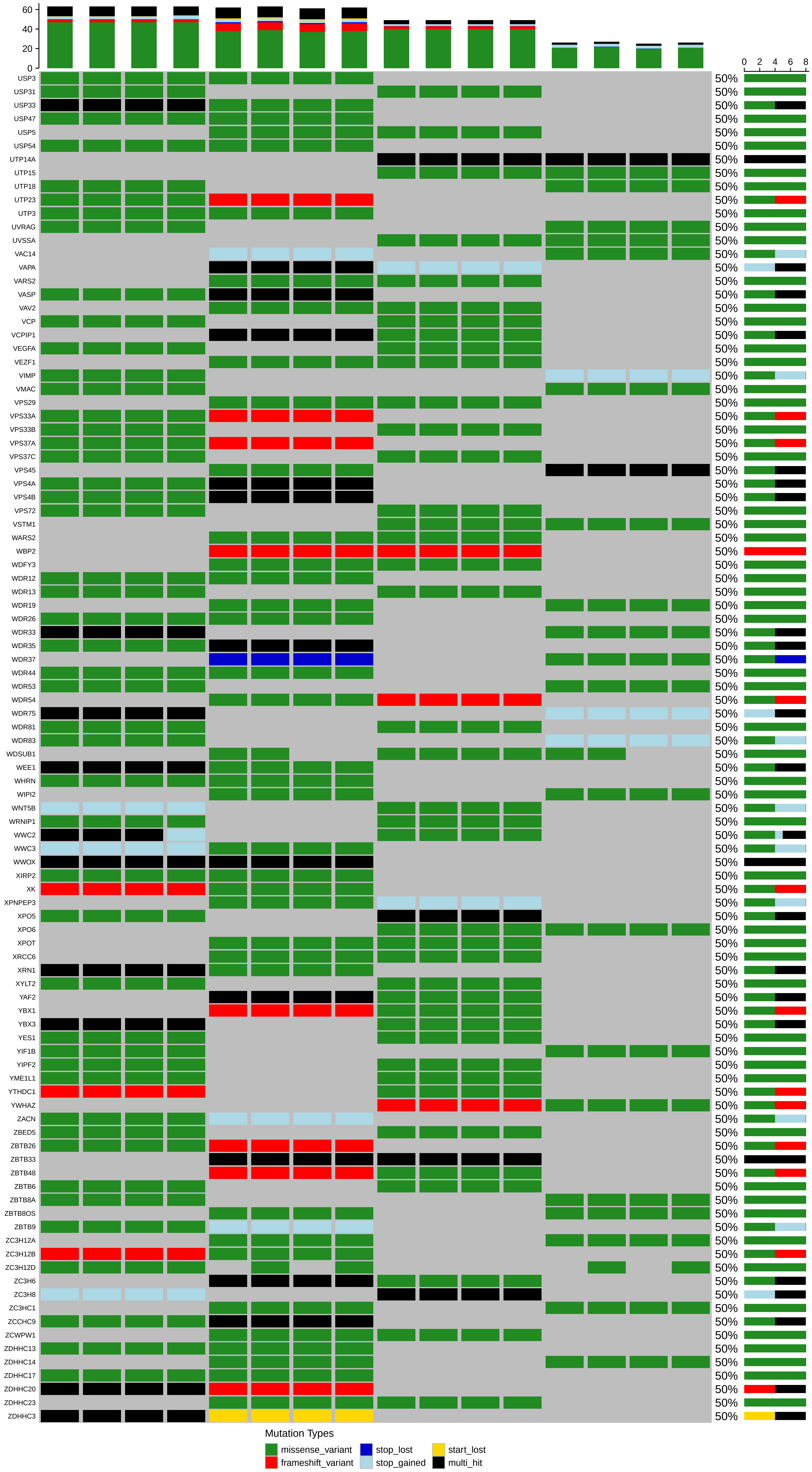

0
20
40
60
0 2 4 6 8
USP3
USP31
USP33
USP47
USP5
USP54
UTP14A
UTP15
UTP18
UTP23
UTP3
UVRAG
UVSSA
VAC14
VAPA
VARS2
VASP
VAV2
VCP
VCPIP1
VEGFA
VEZF1
VIMP
VMAC
VPS29
VPS33A
VPS33B
VPS37A
VPS37C
VPS45
VPS4A
VPS4B
VPS72
VSTM1
WARS2
WBP2
WDFY3
WDR12
WDR13
WDR19
WDR26
WDR33
WDR35
WDR37
WDR44
WDR53
WDR54
WDR75
WDR81
WDR83
WDSUB1
WEE1
WHRN
WIPI2
WNT5B
WRNIP1
WWC2
WWC3
WWOX
XIRP2
XK
XPNPEP3
XPO5
XPO6
XPOT
XRCC6
XRN1
XYLT2
YAF2
YBX1
YBX3
YES1
YIF1B
YIPF2
YME1L1
YTHDC1
YWHAZ
ZACN
ZBED5
ZBTB26
ZBTB33
ZBTB48
ZBTB6
ZBTB8A
ZBTB8OS
ZBTB9
ZC3H12A
ZC3H12B
ZC3H12D
ZC3H6
ZC3H8
ZC3HC1
ZCCHC9
ZCWPW1
ZDHHC13
ZDHHC14
ZDHHC17
ZDHHC20
ZDHHC23
ZDHHC3
50%
Mutation Types
missense_variant
frameshift_variant
stop_lost
stop_gained
start_lost
multi_hit

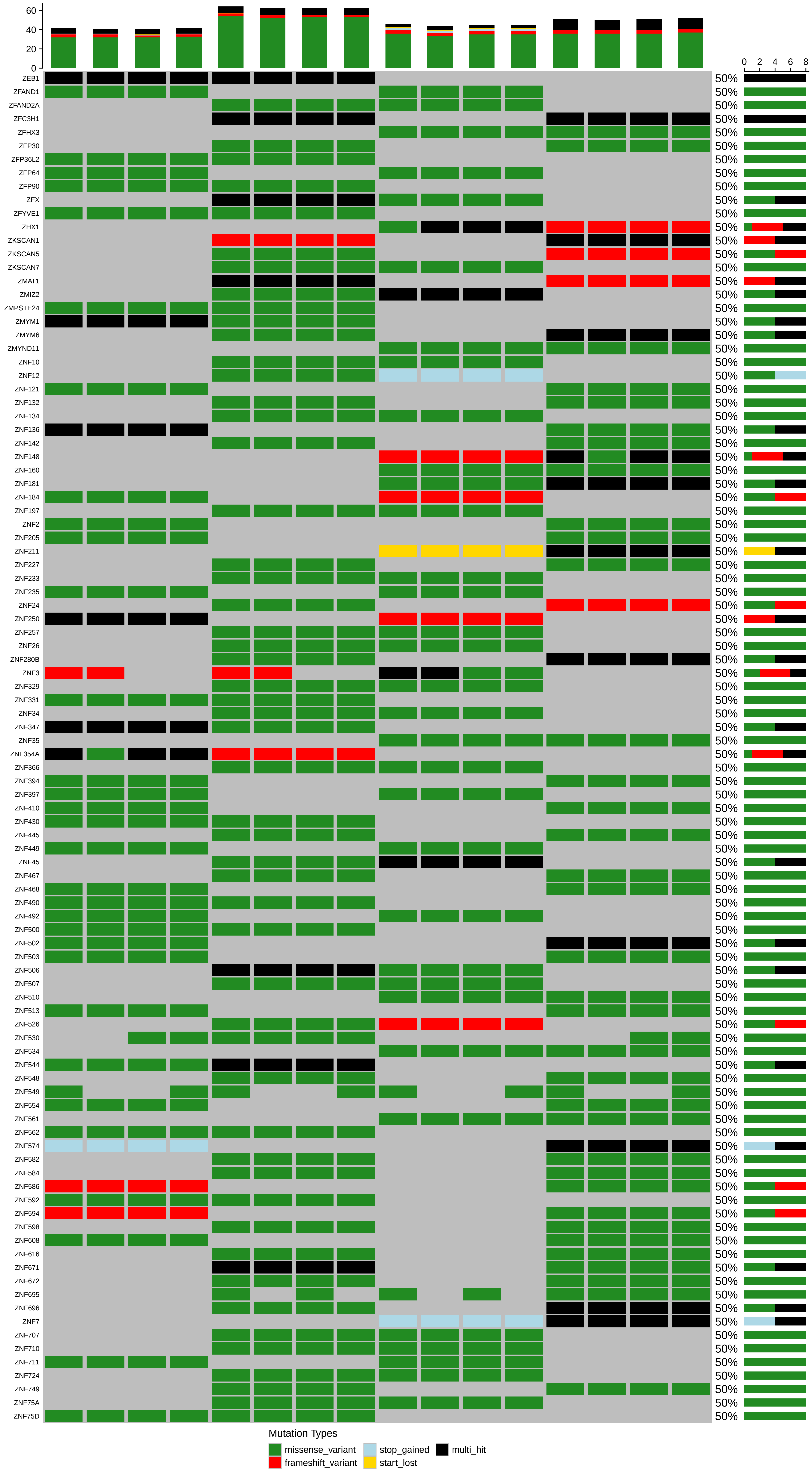

60
40
20
0
0 2 4 6 8
ZEB1
ZFAND1
ZFAND2A
ZFC3H1
ZFHX3
ZFP30
ZFP36L2
ZFP64
ZFP90
ZFX
ZFYVE1
ZHX1
ZKSCAN1
ZKSCAN5
ZKSCAN7
ZMAT1
ZMIZ2
ZMPSTE24
ZMYM1
ZMYM6
ZMYND11
ZNF10
ZNF12
ZNF121
ZNF132
ZNF134
ZNF136
ZNF142
ZNF148
ZNF160
ZNF181
ZNF184
ZNF197
ZNF2
ZNF205
ZNF211
ZNF227
ZNF233
ZNF235
ZNF24
ZNF250
ZNF257
ZNF26
ZNF280B
ZNF3
ZNF329
ZNF331
ZNF34
ZNF347
ZNF35
ZNF354A
ZNF366
ZNF394
ZNF397
ZNF410
ZNF430
ZNF445
ZNF449
ZNF45
ZNF467
ZNF468
ZNF490
ZNF492
ZNF500
ZNF502
ZNF503
ZNF506
ZNF507
ZNF510
ZNF513
ZNF526
ZNF530
ZNF534
ZNF544
ZNF548
ZNF549
ZNF554
ZNF561
ZNF562
ZNF574
ZNF582
ZNF584
ZNF586
ZNF592
ZNF594
ZNF598
ZNF608
ZNF616
ZNF671
ZNF672
ZNF695
ZNF696
ZNF7
ZNF707
ZNF710
ZNF711
ZNF724
ZNF749
ZNF75A
ZNF75D
50%
Mutation Types
missense_variant
frameshift_variant
stop_gained
start_lost
multi_hit

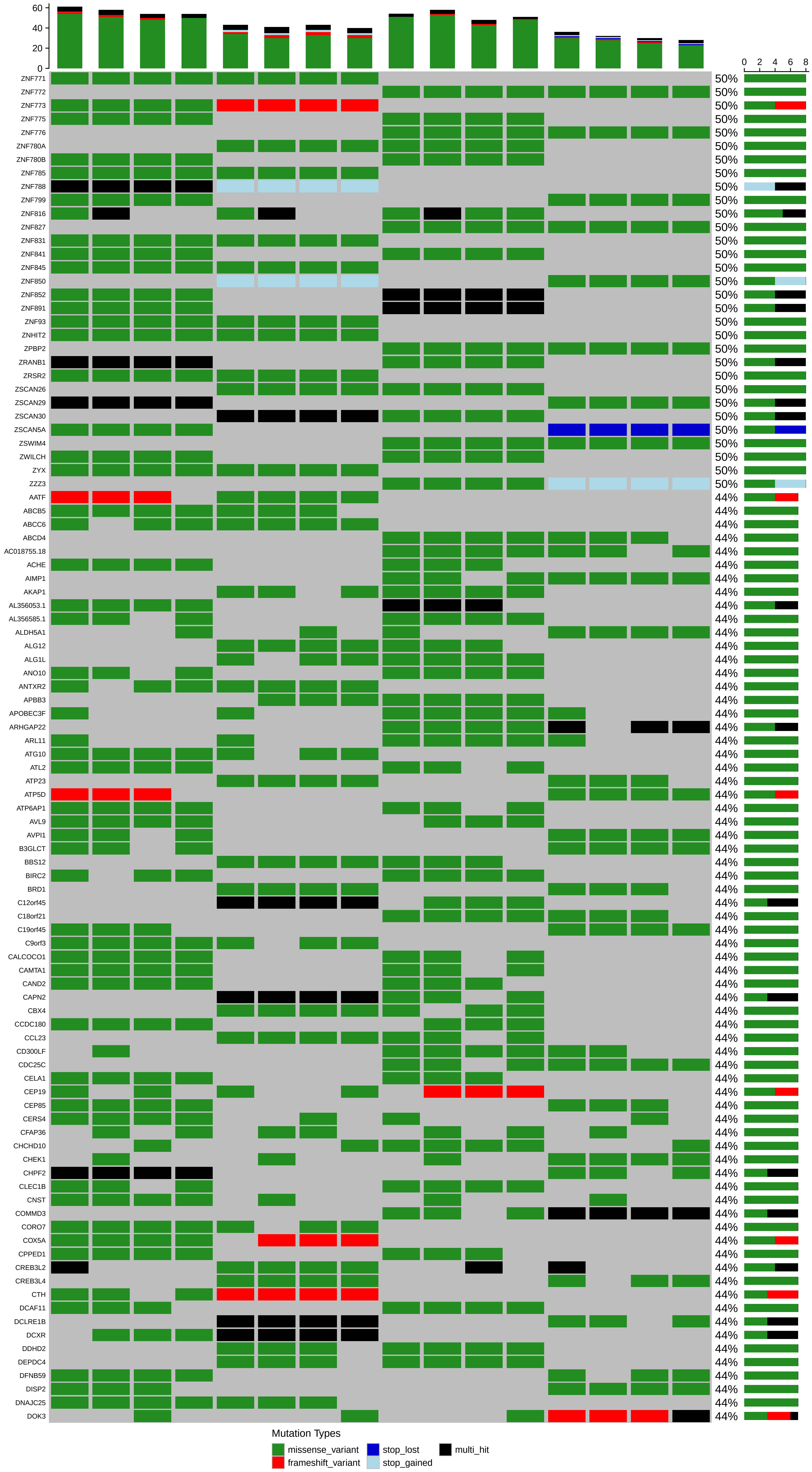
60
40
20
0
0 2 4 6 8
ZNF771 50%
ZNF772 50%
ZNF773 50%
ZNF775 50%
ZNF776 50%
ZNF780A 50%
ZNF780B 50%
ZNF785 50%
ZNF788 50%
ZNF799 50%
ZNF816 50%
ZNF827 50%
ZNF831 50%
ZNF841 50%
ZNF845 50%
ZNF850 50%
ZNF852 50%
ZNF891 50%
ZNF93 50%
ZNHIT2 50%
ZPBP2 50%
ZRANB1 50%
ZRSR2 50%
ZSCAN26 50%
ZSCAN29 50%
ZSCAN30 50%
ZSCAN5A 50%
ZSWIM4 50%
ZWILCH 50%
ZYX 50%
ZZZ3 50%
AATF 44%
ABCB5 44%
ABCC6 44%
ABCD4 44%
AC018755.18 44%
ACHE 44%
AIMP1 44%
AKAP1 44%
AL356053.1 44%
AL356585.1 44%
ALDH5A1 44%
ALG12 44%
ALG1L 44%
ANO10 44%
ANTXR2 44%
APBB3 44%
APOBEC3F 44%
ARHGAP22 44%
ARL11 44%
ATG10 44%
ATL2 44%
ATP23 44%
ATP5D 44%
ATP6AP1 44%
AVL9 44%
AVPI1 44%
B3GLCT 44%
BBS12 44%
BIRC2 44%
BRD1 44%
C12orf45 44%
C18orf21 44%
C19orf45 44%
C9orf3 44%
CALCOCO1 44%
CAMTA1 44%
CAND2 44%
CAPN2 44%
CBX4 44%
CCDC180 44%
CCL23 44%
CD300LF 44%
CDC25C 44%
CELA1 44%
CEP19 44%
CEP85 44%
CERS4 44%
CFAP36 44%
CHCHD10 44%
CHEK1 44%
CHPF2 44%
CLEC1B 44%
CNST 44%
COMMD3 44%
CORO7 44%
COX5A 44%
CPPED1 44%
CREB3L2 44%
CREB3L4 44%
CTH 44%
DCAF11 44%
DCLRE1B 44%
DCXR 44%
DDHD2 44%
DEPDC4 44%
DFNB59 44%
DISP2 44%
DNAJC25 44%
DOK3 44%
Mutation Types
missense_variant
frameshift_variant
stop_lost
stop_gained
multi_hit

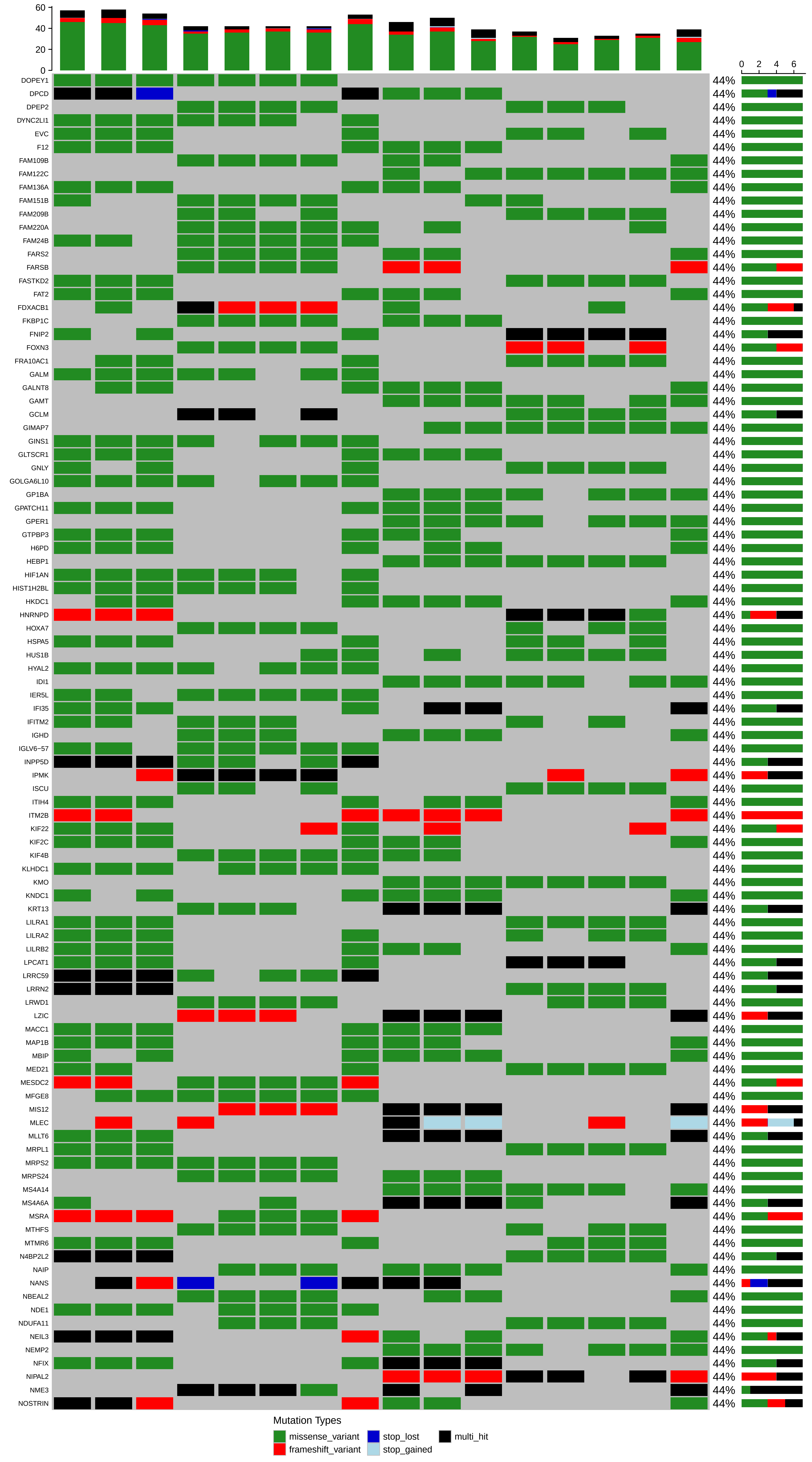
60
40
20
0
0 2 4 6
DOPEY1
DPCD
DPEP2
DYNC2LI1
EVC
F12
FAM109B
FAM122C
FAM136A
FAM151B
FAM209B
FAM220A
FAM24B
FARS2
FARSB
FASTKD2
FAT2
FDXACB1
FKBP1C
FNIP2
FOXN3
FRA10AC1
GALM
GALNT8
GAMT
GCLM
GIMAP7
GINS1
GLTSCR1
GNLY
GOLGA6L10
GP1BA
GPATCH11
GPER1
GTPBP3
H6PD
HEBP1
HIF1AN
HIST1H2BL
HKDC1
HNRNPD
HOXA7
HSPA5
HUS1B
HYAL2
IDI1
IER5L
IFI35
IFITM2
IGHD
IGLV6−57
INPP5D
IPMK
ISCU
ITIH4
ITM2B
KIF22
KIF2C
KIF4B
KLHDC1
KMO
KNDC1
KRT13
LILRA1
LILRA2
LILRB2
LPCAT1
LRRC59
LRRN2
LRWD1
LZIC
MACC1
MAP1B
MBIP
MED21
MESDC2
MFGE8
MIS12
MLEC
MLLT6
MRPL1
MRPS2
MRPS24
MS4A14
MS4A6A
MSRA
MTHFS
MTMR6
N4BP2L2
NAIP
NANS
NBEAL2
NDE1
NDUFA11
NEIL3
NEMP2
NFIX
NIPAL2
NME3
NOSTRIN
44%
Mutation Types
missense_variant
frameshift_variant
stop_lost
stop_gained
multi_hit

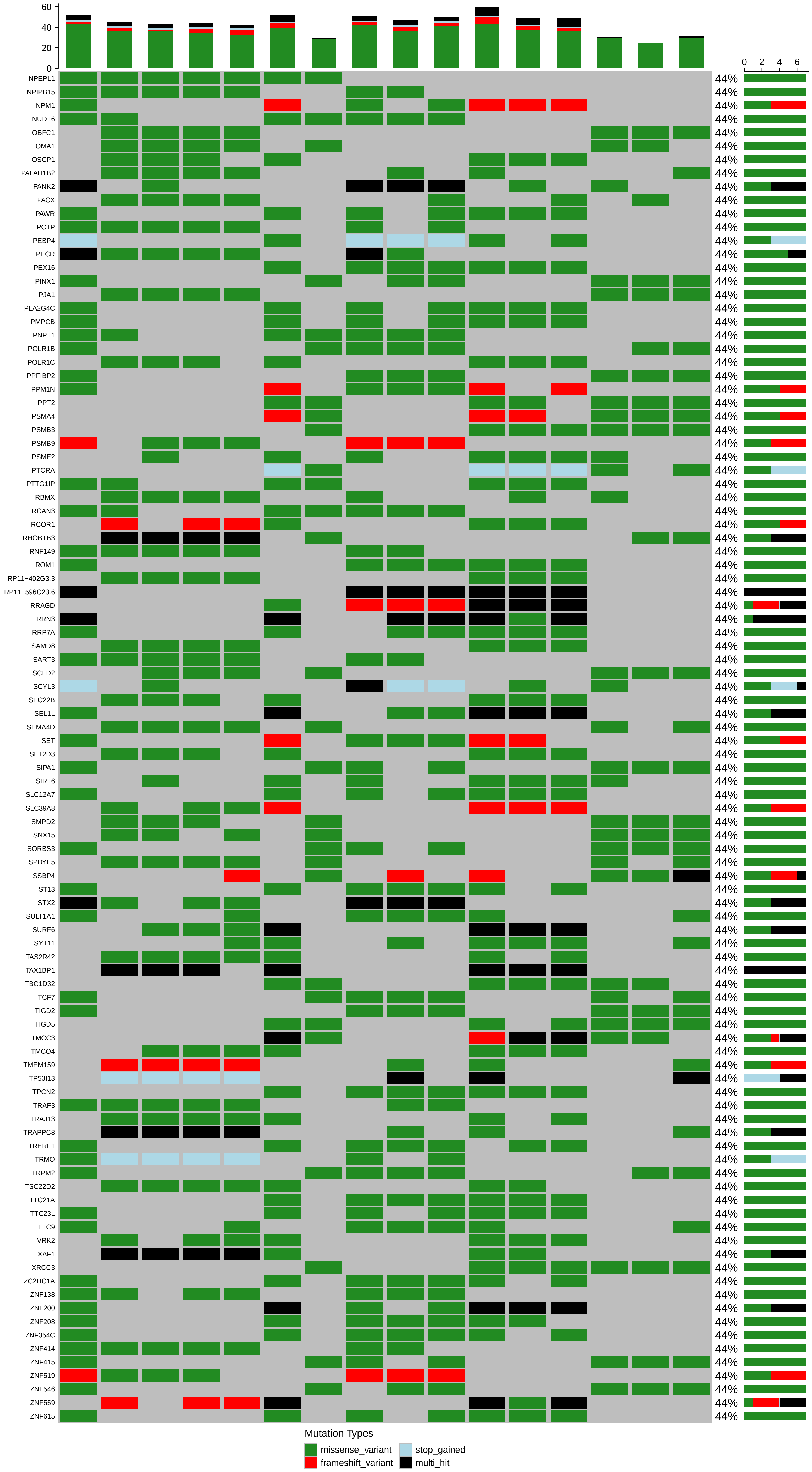

NPEPL1
NPIPB15
NPM1
NUDT6
OBFC1
OMA1
OSCP1
PAFAH1B2
PANK2
PAOX
PAWR
PCTP
PEBP4
PECR
PEX16
PINX1
PJA1
PLA2G4C
PMPCB
PNPT1
POLR1B
POLR1C
PPFIBP2
PPM1N
PPT2
PSMA4
PSMB3
PSMB9
PSME2
PTCRA
PTTG1IP
RBMX
RCAN3
RCOR1
RHOBTB3
RNF149
ROM1
RP11−402G3.3
RP11−596C23.6
RRAGD
RRN3
RRP7A
SAMD8
SART3
SCFD2
SCYL3
SEC22B
SEL1L
SEMA4D
SET
SFT2D3
SIPA1
SIRT6
SLC12A7
SLC39A8
SMPD2
SNX15
SORBS3
SPDYE5
SSBP4
ST13
STX2
SULT1A1
SURF6
SYT11
TAS2R42
TAX1BP1
TBC1D32
TCF7
TIGD2
TIGD5
TMCC3
TMCO4
TMEM159
TP53I13
TPCN2
TRAF3
TRAJ13
TRAPPC8
TRERF1
TRMO
TRPM2
TSC22D2
TTC21A
TTC23L
TTC9
VRK2
XAF1
XRCC3
ZC2HC1A
ZNF138
ZNF200
ZNF208
ZNF354C
ZNF414
ZNF415
ZNF519
ZNF546
ZNF559
ZNF615
44%
Mutation Types
missense_variant
stop_gained
frameshift_variant
multi_hit

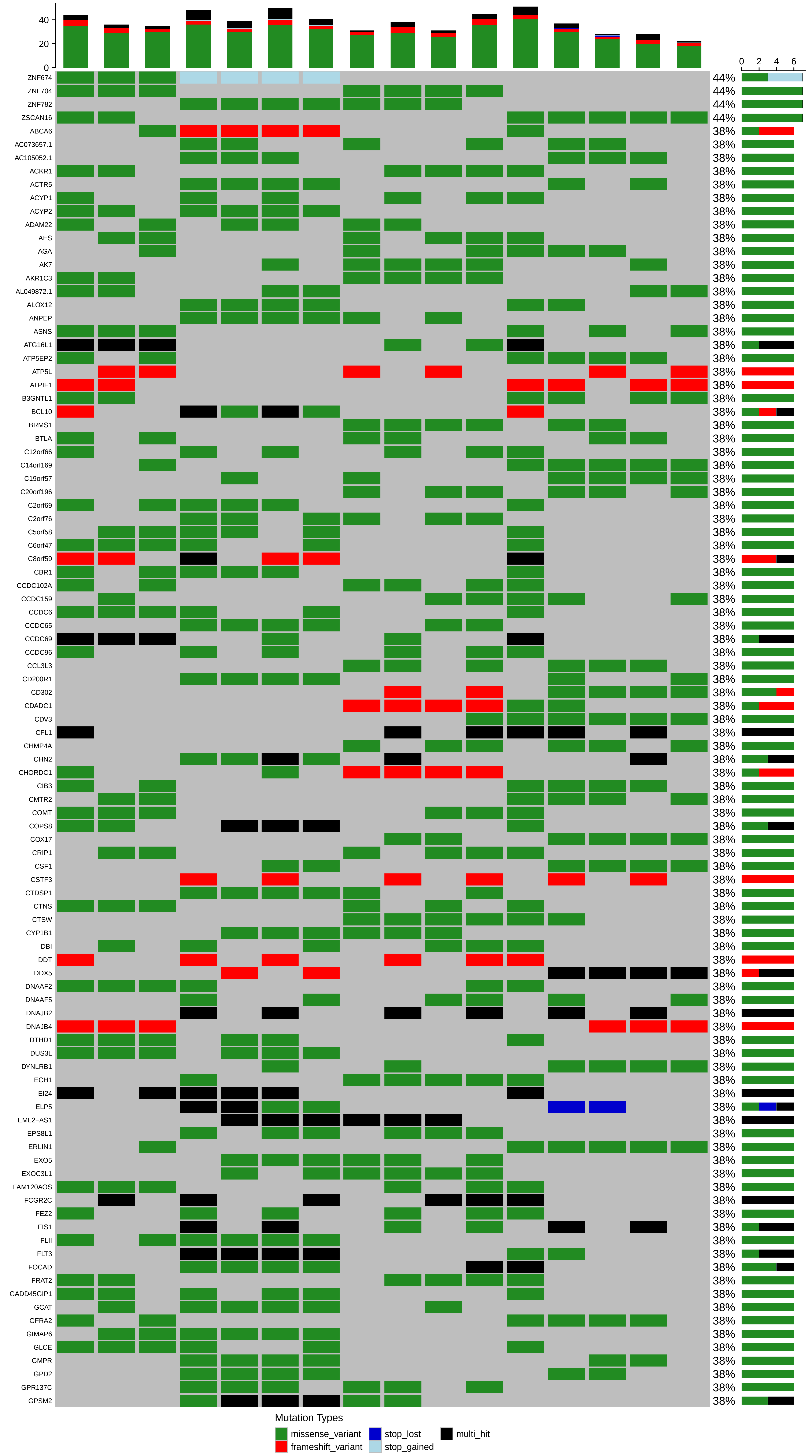

40
20
0
0 2 4 6
ZNF674 44%
ZNF704 44%
ZNF782 44%
ZSCAN16 44%
ABCA6 38%
AC073657.1 38%
AC105052.1 38%
ACKR1 38%
ACTR5 38%
ACYP1 38%
ACYP2 38%
ADAM22 38%
AES 38%
AGA 38%
AK7 38%
AKR1C3 38%
AL049872.1 38%
ALOX12 38%
ANPEP 38%
ASNS 38%
ATG16L1 38%
ATP5EP2 38%
ATP5L 38%
ATPIF1 38%
B3GNTL1 38%
BCL10 38%
BRMS1 38%
BTLA 38%
C12orf66 38%
C14orf169 38%
C19orf57 38%
C20orf196 38%
C2orf69 38%
C2orf76 38%
C5orf58 38%
C6orf47 38%
C8orf59 38%
CBR1 38%
CCDC102A 38%
CCDC159 38%
CCDC6 38%
CCDC65 38%
CCDC69 38%
CCDC96 38%
CCL3L3 38%
CD200R1 38%
CD302 38%
CDADC1 38%
CDV3 38%
CFL1 38%
CHMP4A 38%
CHN2 38%
CHORDC1 38%
CIB3 38%
CMTR2 38%
COMT 38%
COPS8 38%
COX17 38%
CRIP1 38%
CSF1 38%
CSTF3 38%
CTDSP1 38%
CTNS 38%
CTSW 38%
CYP1B1 38%
DBI 38%
DDT 38%
DDX5 38%
DNAAF2 38%
DNAAF5 38%
DNAJB2 38%
DNAJB4 38%
DTHD1 38%
DUS3L 38%
DYNLRB1 38%
ECH1 38%
EI24 38%
ELP5 38%
EML2−AS1 38%
EPS8L1 38%
ERLIN1 38%
EXO5 38%
EXOC3L1 38%
FAM120AOS 38%
FCGR2C 38%
FEZ2 38%
FIS1 38%
FLII 38%
FLT3 38%
FOCAD 38%
FRAT2 38%
GADD45GIP1 38%
GCAT 38%
GFRA2 38%
GIMAP6 38%
GLCE 38%
GMPR 38%
GPD2 38%
GPR137C 38%
GPSM2 38%
Mutation Types
missense_variant
frameshift_variant
stop_lost
stop_gained
multi_hit

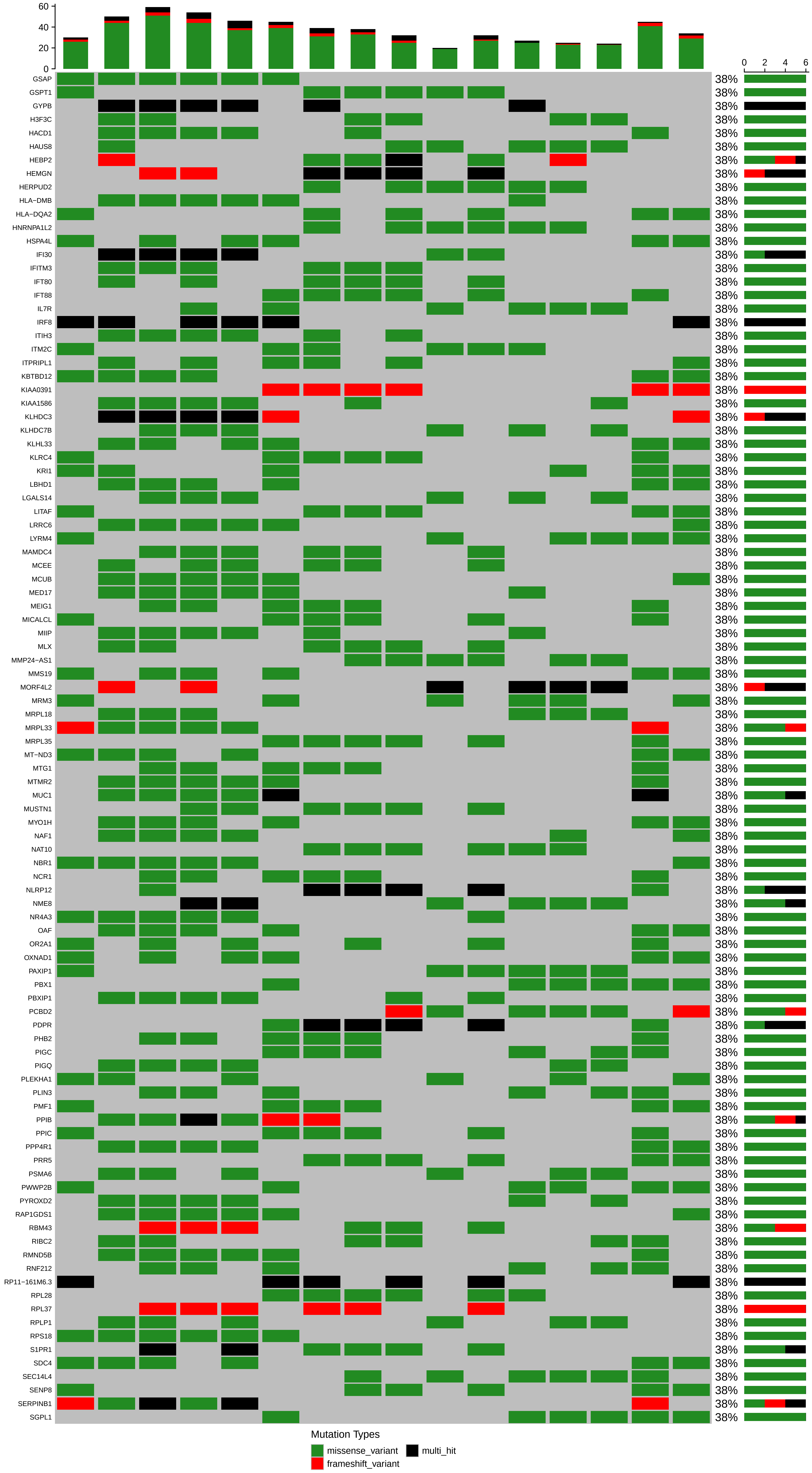

60
40
20
0
0 2 4 6
GSAP
GSPT1
GYPB
H3F3C
HACD1
HAUS8
HEBP2
HEMGN
HERPUD2
HLA−DMB
HLA−DQA2
HNRNPA1L2
HSPA4L
IFI30
IFITM3
IFT80
IFT88
IL7R
IRF8
ITIH3
ITM2C
ITPRIPL1
KBTBD12
KIAA0391
KIAA1586
KLHDC3
KLHDC7B
KLHL33
KLRC4
KRI1
LBHD1
LGALS14
LITAF
LRRC6
LYRM4
MAMDC4
MCEE
MCUB
MED17
MEIG1
MICALCL
MIIP
MLX
MMP24−AS1
MMS19
MORF4L2
MRM3
MRPL18
MRPL33
MRPL35
MT−ND3
MTG1
MTMR2
MUC1
MUSTN1
MYO1H
NAF1
NAT10
NBR1
NCR1
NLRP12
NME8
NR4A3
OAF
OR2A1
OXNAD1
PAXIP1
PBX1
PBXIP1
PCBD2
PDPR
PHB2
PIGC
PIGQ
PLEKHA1
PLIN3
PMF1
PPIB
PPIC
PPP4R1
PRR5
PSMA6
PWWP2B
PYROXD2
RAP1GDS1
RBM43
RIBC2
RMND5B
RNF212
RP11−161M6.3
RPL28
RPL37
RPLP1
RPS18
S1PR1
SDC4
SEC14L4
SENP8
SERPINB1
SGPL1
38%
Mutation Types
missense_variant
multi_hit
frameshift_variant

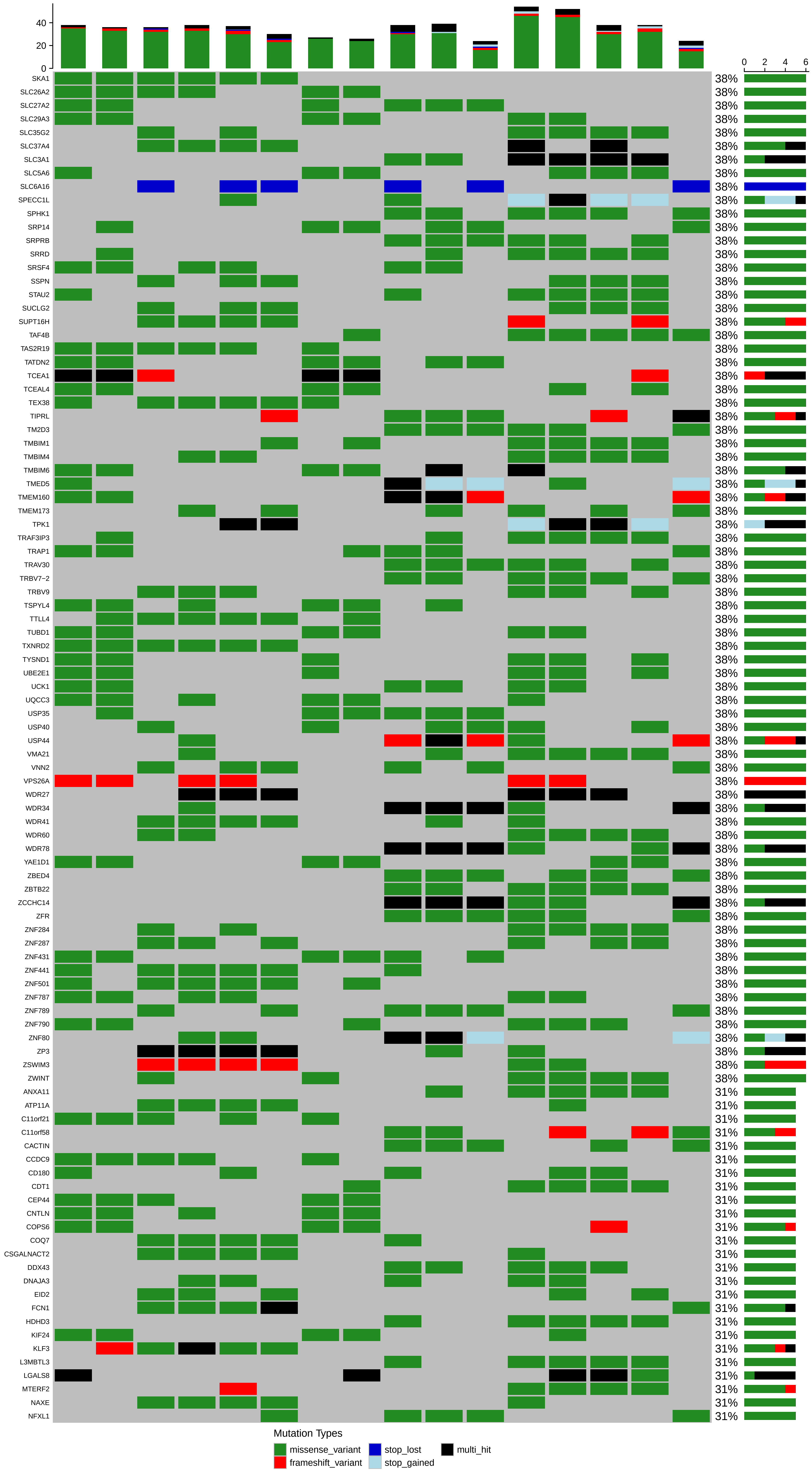

0
20
40
0
2
4
6
SKA1 38%
SLC26A2 38%
SLC27A2 38%
SLC29A3 38%
SLC35G2 38%
SLC37A4 38%
SLC3A1 38%
SLC5A6 38%
SLC6A16 38%
SPECC1L 38%
SPHK1 38%
SRP14 38%
SRPRB 38%
SRRD 38%
SRSF4 38%
SSPN 38%
STAU2 38%
SUCLG2 38%
SUPT16H 38%
TAF4B 38%
TAS2R19 38%
TATDN2 38%
TCEA1 38%
TCEAL4 38%
TEX38 38%
TIPRL 38%
TM2D3 38%
TMBIM1 38%
TMBIM4 38%
TMBIM6 38%
TMED5 38%
TMEM160 38%
TMEM173 38%
TPK1 38%
TRAF3IP3 38%
TRAP1 38%
TRAV30 38%
TRBV7−2 38%
TRBV9 38%
TSPYL4 38%
TTLL4 38%
TUBD1 38%
TXNRD2 38%
TYSND1 38%
UBE2E1 38%
UCK1 38%
UQCC3 38%
USP35 38%
USP40 38%
USP44 38%
VMA21 38%
VNN2 38%
VPS26A 38%
WDR27 38%
WDR34 38%
WDR41 38%
WDR60 38%
WDR78 38%
YAE1D1 38%
ZBED4 38%
ZBTB22 38%
ZCCHC14 38%
ZFR 38%
ZNF284 38%
ZNF287 38%
ZNF431 38%
ZNF441 38%
ZNF501 38%
ZNF787 38%
ZNF789 38%
ZNF790 38%
ZNF80 38%
ZP3 38%
ZSWIM3 38%
ZWINT 38%
ANXA11 31%
ATP11A 31%
C11orf21 31%
C11orf58 31%
CACTIN 31%
CCDC9 31%
CD180 31%
CDT1 31%
CEP44 31%
CNTLN 31%
COPS6 31%
COQ7 31%
CSGALNACT2 31%
DDX43 31%
DNAJA3 31%
EID2 31%
FCN1 31%
HDHD3 31%
KIF24 31%
KLF3 31%
L3MBTL3 31%
LGALS8 31%
MTERF2 31%
NAXE 31%
NFXL1 31%
Mutation Types
missense_variant
frameshift_variant
stop_lost
stop_gained
multi_hit

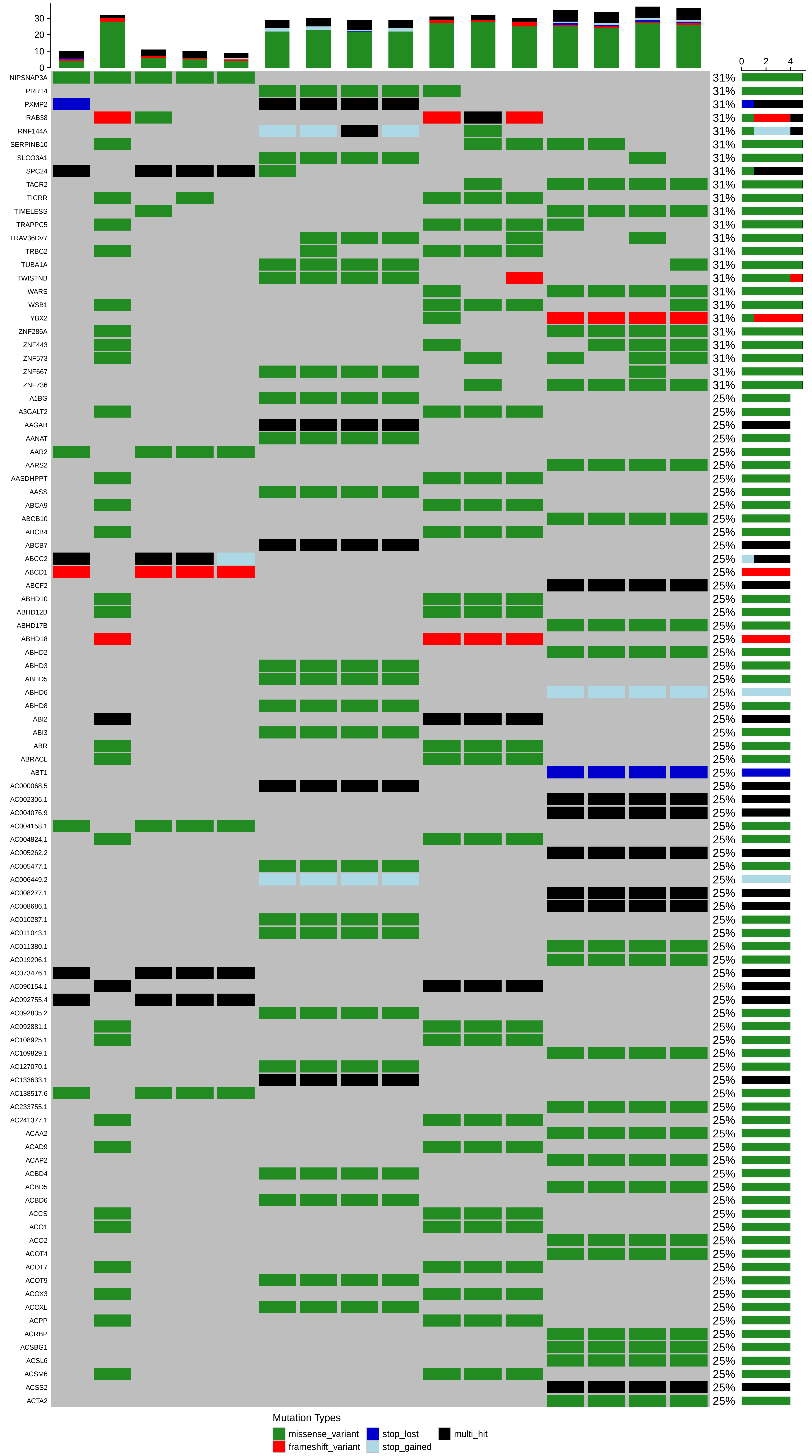

30
20
10
0
0 2 4
NIPSNAP3A 31%
PRR14 31%
PXMP2 31%
RAB38 31%
RNF144A 31%
SERPINB10 31%
SLCO3A1 31%
SPC24 31%
TACR2 31%
TICRR 31%
TIMELESS 31%
TRAPPC5 31%
TRAV36DV7 31%
TRBC2 31%
TUBA1A 31%
TWISTNB 31%
WARS 31%
WSB1 31%
YBX2 31%
ZNF286A 31%
ZNF443 31%
ZNF573 31%
ZNF667 31%
ZNF736 31%
A1BG 25%
A3GALT2 25%
AAGAB 25%
AANAT 25%
AAR2 25%
AARS2 25%
AASDHPPT 25%
AASS 25%
ABCA9 25%
ABCB10 25%
ABCB4 25%
ABCB7 25%
ABCC2 25%
ABCD1 25%
ABCF2 25%
ABHD10 25%
ABHD12B 25%
ABHD17B 25%
ABHD18 25%
ABHD2 25%
ABHD3 25%
ABHD5 25%
ABHD6 25%
ABHD8 25%
ABI2 25%
ABI3 25%
ABR 25%
ABRACL 25%
ABT1 25%
AC000068.5 25%
AC002306.1 25%
AC004076.9 25%
AC004158.1 25%
AC004824.1 25%
AC005262.2 25%
AC005477.1 25%
AC006449.2 25%
AC008277.1 25%
AC008686.1 25%
AC010287.1 25%
AC011043.1 25%
AC011380.1 25%
AC019206.1 25%
AC073476.1 25%
AC090154.1 25%
AC092755.4 25%
AC092835.2 25%
AC092881.1 25%
AC108925.1 25%
AC109829.1 25%
AC127070.1 25%
AC133633.1 25%
AC138517.6 25%
AC233755.1 25%
AC241377.1 25%
ACAA2 25%
ACAD9 25%
ACAP2 25%
ACBD4 25%
ACBD5 25%
ACBD6 25%
ACCS 25%
ACO1 25%
ACO2 25%
ACOT4 25%
ACOT7 25%
ACOT9 25%
ACOX3 25%
ACOXL 25%
ACPP 25%
ACRBP 25%
ACSBG1 25%
ACSL6 25%
ACSM6 25%
ACSS2 25%
ACTA2 25%
Mutation Types
missense_variant
stop_lost
multi_hit
frameshift_variant
stop_gained

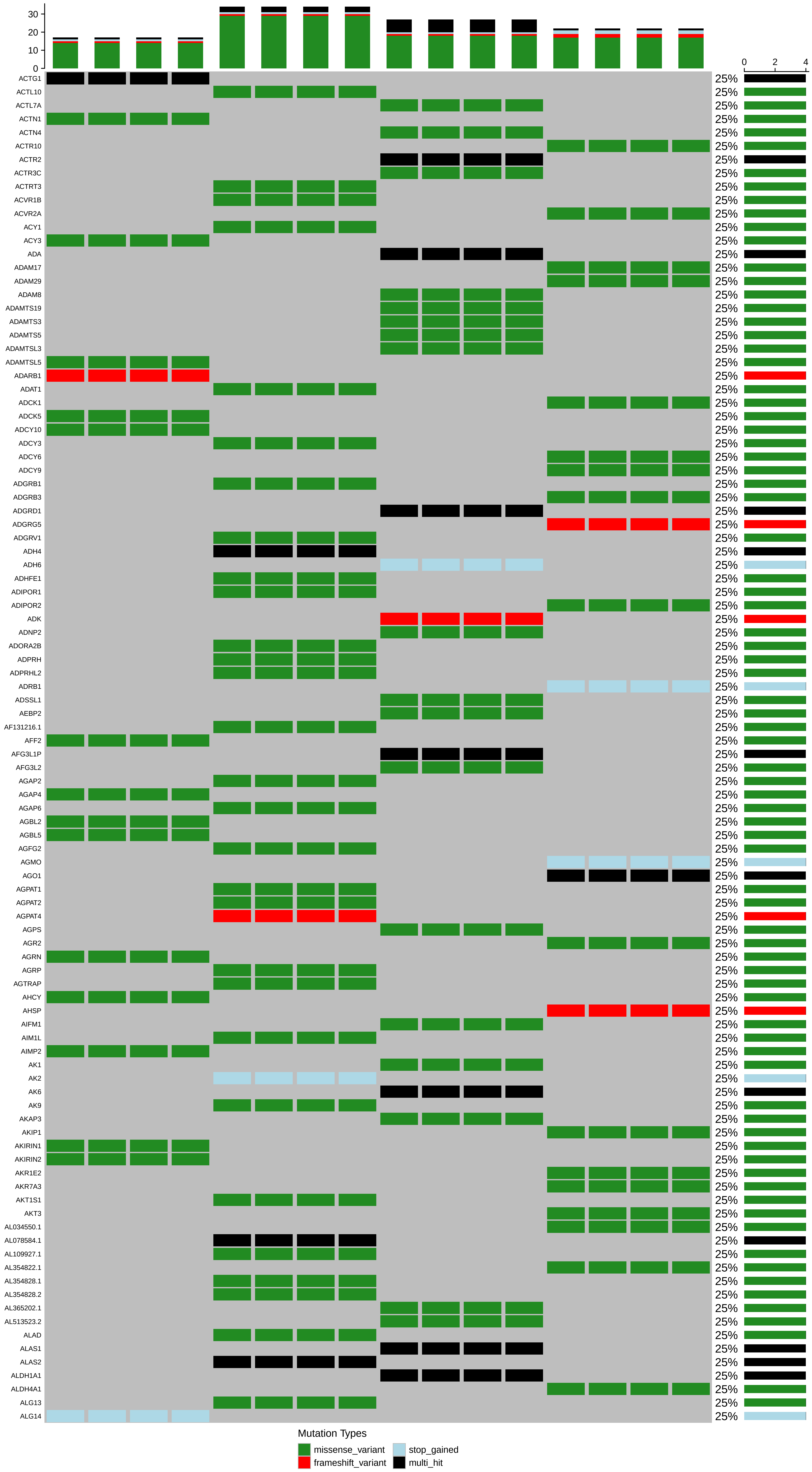
30
20
10
0
0
2
4
ACTG1
ACTL10
ACTL7A
ACTN1
ACTN4
ACTR10
ACTR2
ACTR3C
ACTRT3
ACVR1B
ACVR2A
ACY1
ACY3
ADA
ADAM17
ADAM29
ADAM8
ADAMTS19
ADAMTS3
ADAMTS5
ADAMTSL3
ADAMTSL5
ADARB1
ADAT1
ADCK1
ADCK5
ADCY10
ADCY3
ADCY6
ADCY9
ADGRB1
ADGRB3
ADGRD1
ADGRG5
ADGRV1
ADH4
ADH6
ADHFE1
ADIPOR1
ADIPOR2
ADK
ADNP2
ADORA2B
ADPRH
ADPRHL2
ADRB1
ADSSL1
AEBP2
AF131216.1
AFF2
AFG3L1P
AFG3L2
AGAP2
AGAP4
AGAP6
AGBL2
AGBL5
AGFG2
AGMO
AGO1
AGPAT1
AGPAT2
AGPAT4
AGPS
AGR2
AGRN
AGRP
AGTRAP
AHCY
AHSP
AIFM1
AIM1L
AIMP2
AK1
AK2
AK6
AK9
AKAP3
AKIP1
AKIRIN1
AKIRIN2
AKR1E2
AKR7A3
AKT1S1
AKT3
AL034550.1
AL078584.1
AL109927.1
AL354822.1
AL354828.1
AL354828.2
AL365202.1
AL513523.2
ALAD
ALAS1
ALAS2
ALDH1A1
ALDH4A1
ALG13
ALG14
25%
Mutation Types
missense_variant
frameshift_variant
stop_gained
multi_hit

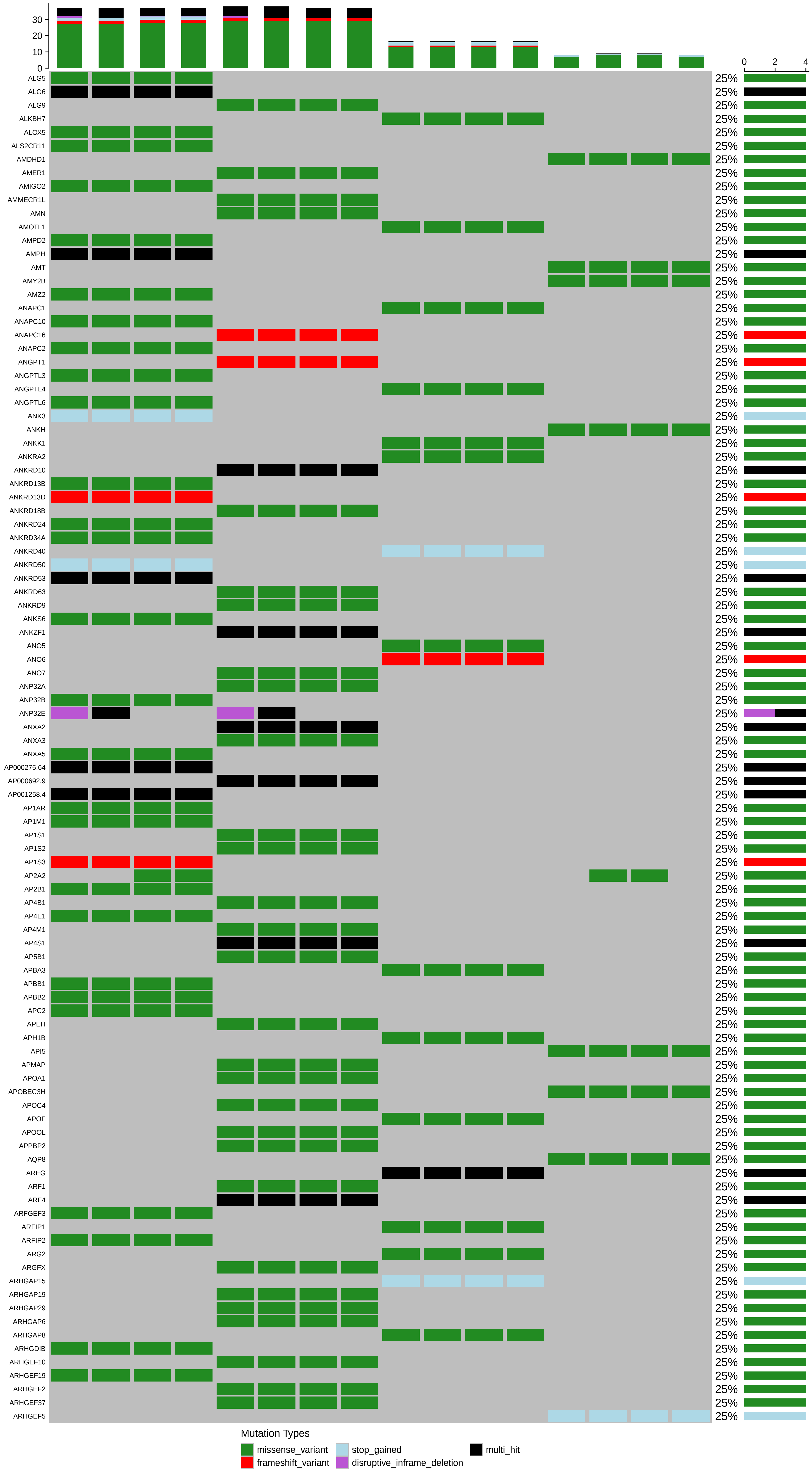

0
10
20
30
0
2
4
ALG5
ALG6
ALG9
ALKBH7
ALOX5
ALS2CR11
AMDHD1
AMER1
AMIGO2
AMMECR1L
AMN
AMOTL1
AMPD2
AMPH
AMT
AMY2B
AMZ2
ANAPC1
ANAPC10
ANAPC16
ANAPC2
ANGPT1
ANGPTL3
ANGPTL4
ANGPTL6
ANK3
ANKH
ANKK1
ANKRA2
ANKRD10
ANKRD13B
ANKRD13D
ANKRD18B
ANKRD24
ANKRD34A
ANKRD40
ANKRD50
ANKRD53
ANKRD63
ANKRD9
ANKS6
ANKZF1
ANO5
ANO6
ANO7
ANP32A
ANP32B
ANP32E
ANXA2
ANXA3
ANXA5
AP000275.64
AP000692.9
AP001258.4
AP1AR
AP1M1
AP1S1
AP1S2
AP1S3
AP2A2
AP2B1
AP4B1
AP4E1
AP4M1
AP4S1
AP5B1
APBA3
APBB1
APBB2
APC2
APEH
APH1B
API5
APMAP
APOA1
APOBEC3H
APOC4
APOF
APOOL
APPBP2
AQP8
AREG
ARF1
ARF4
ARFGEF3
ARFIP1
ARFIP2
ARG2
ARGFX
ARHGAP15
ARHGAP19
ARHGAP29
ARHGAP6
ARHGAP8
ARHGDIB
ARHGEF10
ARHGEF19
ARHGEF2
ARHGEF37
ARHGEF5
25%
Mutation Types
missense_variant
frameshift_variant
stop_gained
disruptive_inframe_deletion
multi_hit

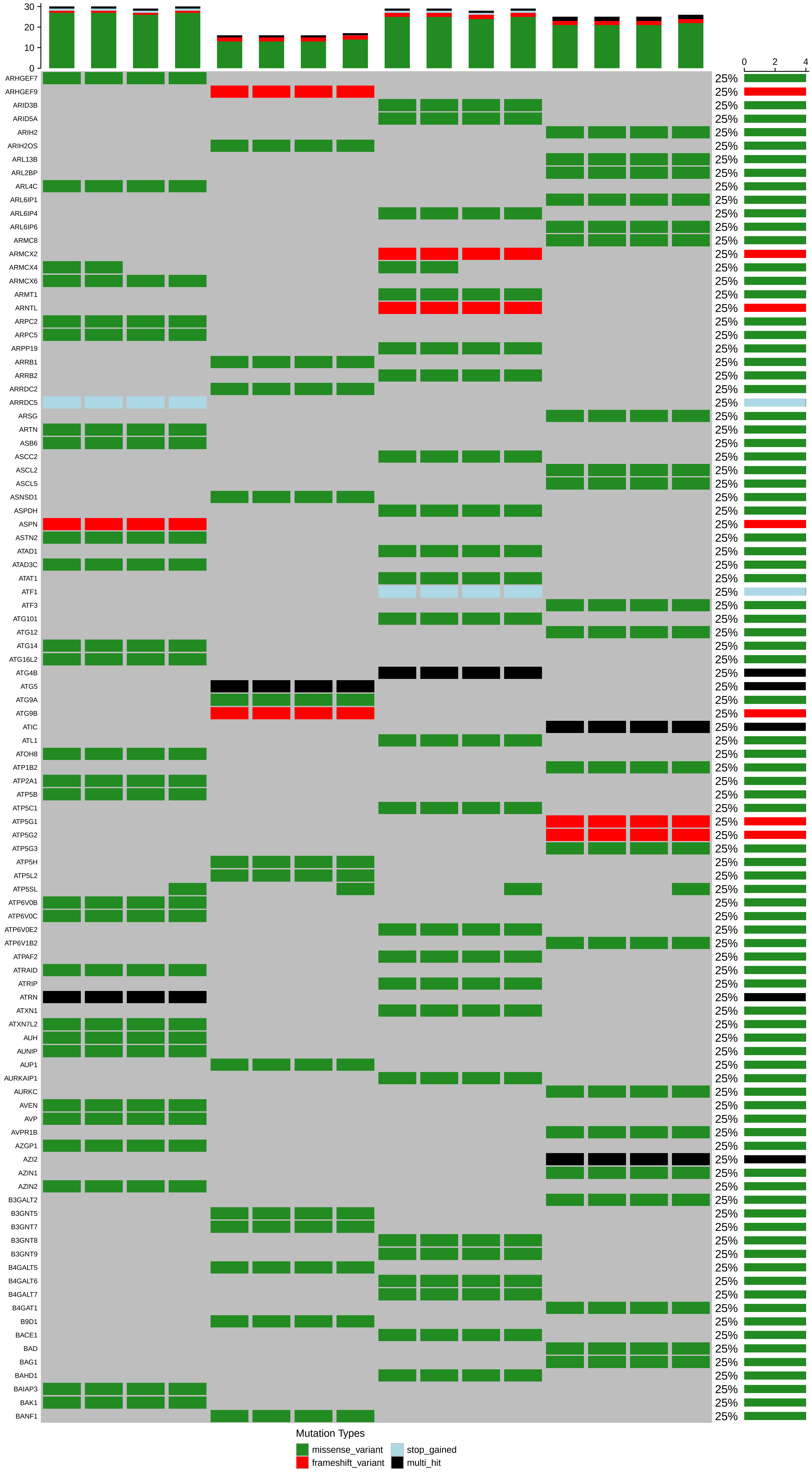

30
20
10
0
0
2
4
ARHGEF7
ARHGEF9
ARID3B
ARID5A
ARIH2
ARIH2OS
ARL13B
ARL2BP
ARL4C
ARL6IP1
ARL6IP4
ARL6IP6
ARMC8
ARMCX2
ARMCX4
ARMCX6
ARMT1
ARNTL
ARPC2
ARPC5
ARPP19
ARRB1
ARRB2
ARRDC2
ARRDC5
ARSG
ARTN
ASB6
ASCC2
ASCL2
ASCL5
ASNSD1
ASPDH
ASPN
ASTN2
ATAD1
ATAD3C
ATAT1
ATF1
ATF3
ATG101
ATG12
ATG14
ATG16L2
ATG4B
ATG5
ATG9A
ATG9B
ATIC
ATL1
ATOH8
ATP1B2
ATP2A1
ATP5B
ATP5C1
ATP5G1
ATP5G2
ATP5G3
ATP5H
ATP5L2
ATP5SL
ATP6V0B
ATP6V0C
ATP6V0E2
ATP6V1B2
ATPAF2
ATRAID
ATRIP
ATRN
ATXN1
ATXN7L2
AUH
AUNIP
AUP1
AURKAIP1
AURKC
AVEN
AVP
AVPR1B
AZGP1
AZI2
AZIN1
AZIN2
B3GALT2
B3GNT5
B3GNT7
B3GNT8
B3GNT9
B4GALT5
B4GALT6
B4GALT7
B4GAT1
B9D1
BACE1
BAD
BAG1
BAHD1
BAIAP3
BAK1
BANF1
25%
Mutation Types
missense_variant
frameshift_variant
stop_gained
multi_hit

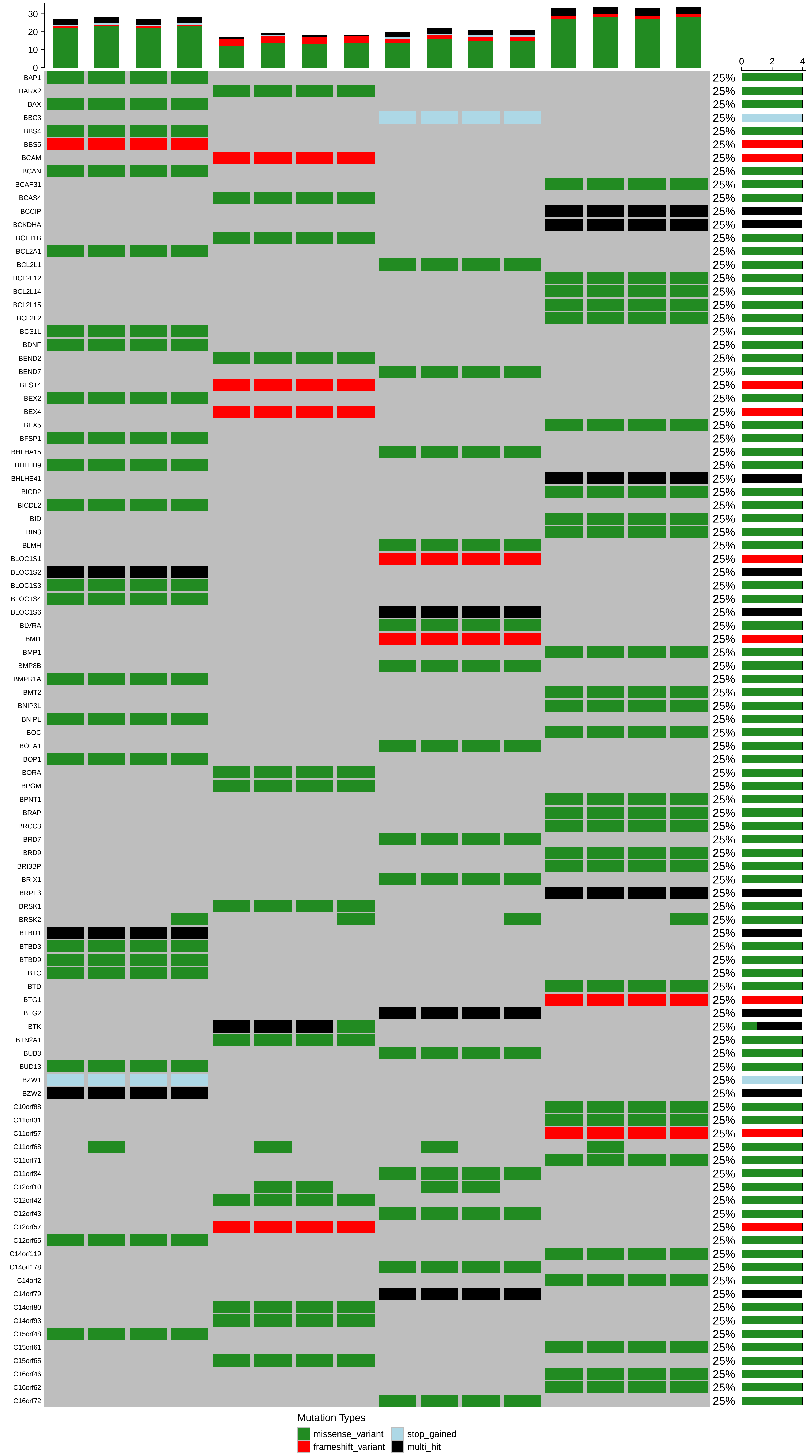

30
20
10
0
0
2
4
BAP1
BARX2
BAX
BBC3
BBS4
BBS5
BCAM
BCAN
BCAP31
BCAS4
BCCIP
BCKDHA
BCL11B
BCL2A1
BCL2L1
BCL2L12
BCL2L14
BCL2L15
BCL2L2
BCS1L
BDNF
BEND2
BEND7
BEST4
BEX2
BEX4
BEX5
BFSP1
BHLHA15
BHLHB9
BHLHE41
BICD2
BICDL2
BID
BIN3
BLMH
BLOC1S1
BLOC1S2
BLOC1S3
BLOC1S4
BLOC1S6
BLVRA
BMI1
BMP1
BMP8B
BMPR1A
BMT2
BNIP3L
BNIPL
BOC
BOLA1
BOP1
BORA
BPGM
BPNT1
BRAP
BRCC3
BRD7
BRD9
BRI3BP
BRIX1
BRPF3
BRSK1
BRSK2
BTBD1
BTBD3
BTBD9
BTC
BTD
BTG1
BTG2
BTK
BTN2A1
BUB3
BUD13
BZW1
BZW2
C10orf88
C11orf31
C11orf57
C11orf68
C11orf71
C11orf84
C12orf10
C12orf42
C12orf43
C12orf57
C12orf65
C14orf119
C14orf178
C14orf2
C14orf79
C14orf80
C14orf93
C15orf48
C15orf61
C15orf65
C16orf46
C16orf62
C16orf72
Mutation Types
missense_variant
frameshift_variant
stop_gained
multi_hit

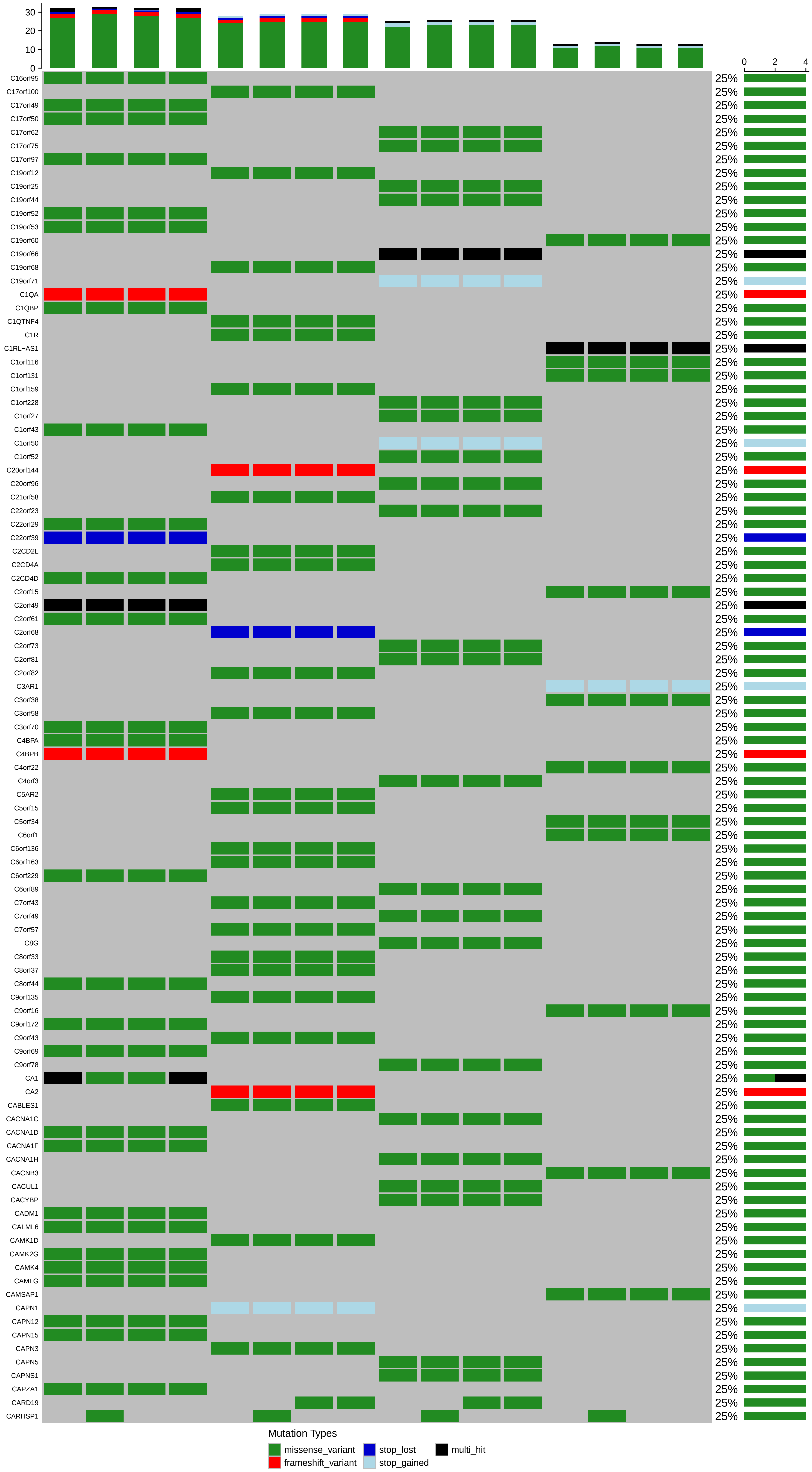

30
20
10
0
0
2
4
C16orf95
C17orf100
C17orf49
C17orf50
C17orf62
C17orf75
C17orf97
C19orf12
C19orf25
C19orf44
C19orf52
C19orf53
C19orf60
C19orf66
C19orf68
C19orf71
C1QA
C1QBP
C1QTNF4
C1R
C1RL−AS1
C1orf116
C1orf131
C1orf159
C1orf228
C1orf27
C1orf43
C1orf50
C1orf52
C20orf144
C20orf96
C21orf58
C22orf23
C22orf29
C22orf39
C2CD2L
C2CD4A
C2CD4D
C2orf15
C2orf49
C2orf61
C2orf68
C2orf73
C2orf81
C2orf82
C3AR1
C3orf38
C3orf58
C3orf70
C4BPA
C4BPB
C4orf22
C4orf3
C5AR2
C5orf15
C5orf34
C6orf1
C6orf136
C6orf163
C6orf229
C6orf89
C7orf43
C7orf49
C7orf57
C8G
C8orf33
C8orf37
C8orf44
C9orf135
C9orf16
C9orf172
C9orf43
C9orf69
C9orf78
CA1
CA2
CABLES1
CACNA1C
CACNA1D
CACNA1F
CACNA1H
CACNB3
CACUL1
CACYBP
CADM1
CALML6
CAMK1D
CAMK2G
CAMK4
CAMLG
CAMSAP1
CAPN1
CAPN12
CAPN15
CAPN3
CAPN5
CAPNS1
CAPZA1
CARD19
CARHSP1
25%
Mutation Types
missense_variant
frameshift_variant
stop_lost
stop_gained
multi_hit

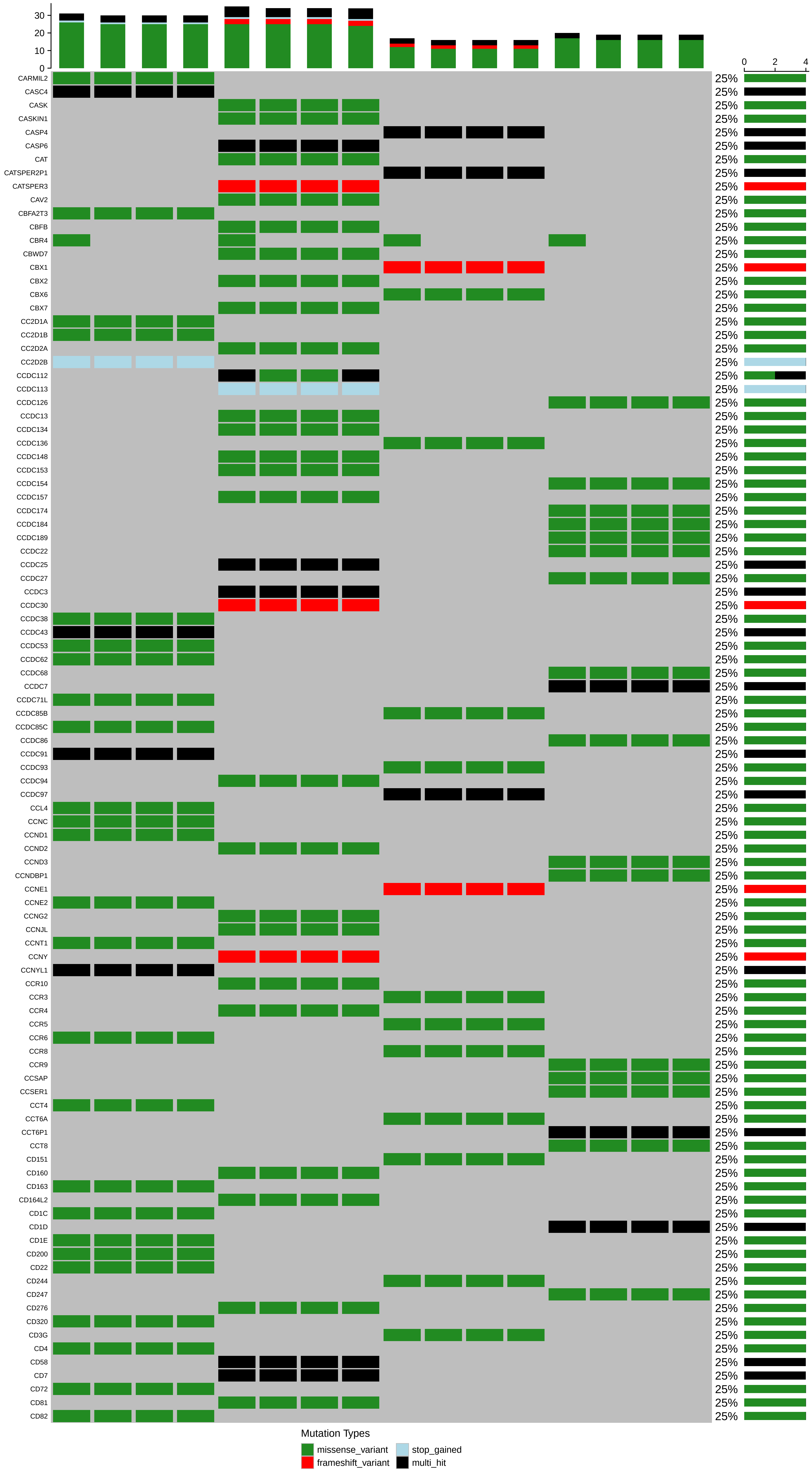

30
20
10
0
0
2
4
CARMIL2
CASC4
CASK
CASKIN1
CASP4
CASP6
CAT
CATSPER2P1
CATSPER3
CAV2
CBFA2T3
CBFB
CBR4
CBWD7
CBX1
CBX2
CBX6
CBX7
CC2D1A
CC2D1B
CC2D2A
CC2D2B
CCDC112
CCDC113
CCDC126
CCDC13
CCDC134
CCDC136
CCDC148
CCDC153
CCDC154
CCDC157
CCDC174
CCDC184
CCDC189
CCDC22
CCDC25
CCDC27
CCDC3
CCDC30
CCDC38
CCDC43
CCDC53
CCDC62
CCDC68
CCDC7
CCDC71L
CCDC85B
CCDC85C
CCDC86
CCDC91
CCDC93
CCDC94
CCDC97
CCL4
CCNC
CCND1
CCND2
CCND3
CCNDBP1
CCNE1
CCNE2
CCNG2
CCNJL
CCNT1
CCNY
CCNYL1
CCR10
CCR3
CCR4
CCR5
CCR6
CCR8
CCR9
CCSAP
CCSER1
CCT4
CCT6A
CCT6P1
CCT8
CD151
CD160
CD163
CD164L2
CD1C
CD1D
CD1E
CD200
CD22
CD244
CD247
CD276
CD320
CD3G
CD4
CD58
CD7
CD72
CD81
CD82
25% (each of the 100 rows above)
Mutation Types
missense_variant
frameshift_variant
stop_gained
multi_hit

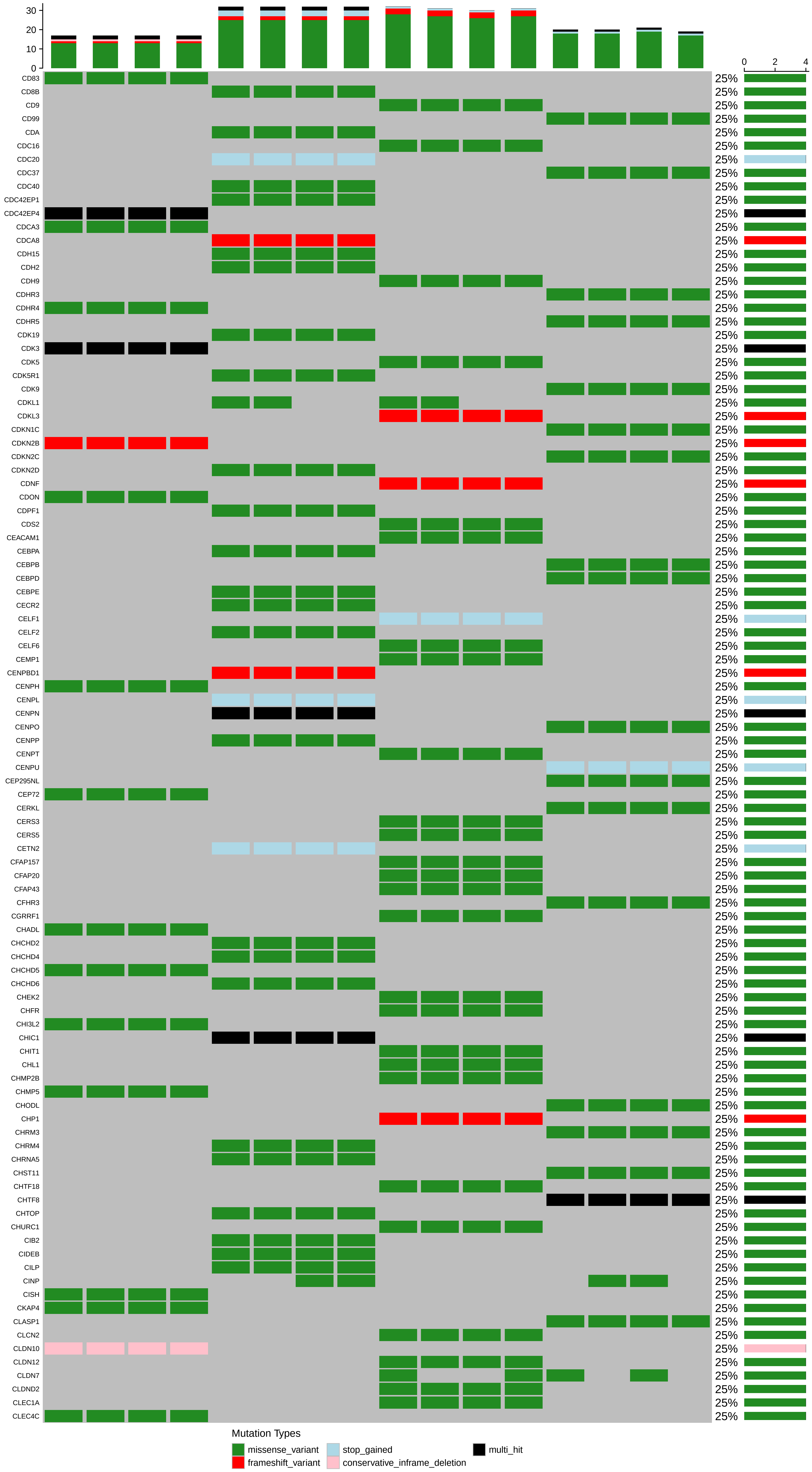
30
20
10
0
0
2
4
CD83
CD8B
CD9
CD99
CDA
CDC16
CDC20
CDC37
CDC40
CDC42EP1
CDC42EP4
CDCA3
CDCA8
CDH15
CDH2
CDH9
CDHR3
CDHR4
CDHR5
CDK19
CDK3
CDK5
CDK5R1
CDK9
CDKL1
CDKL3
CDKN1C
CDKN2B
CDKN2C
CDKN2D
CDNF
CDON
CDPF1
CDS2
CEACAM1
CEBPA
CEBPB
CEBPD
CEBPE
CECR2
CELF1
CELF2
CELF6
CEMP1
CENPBD1
CENPH
CENPL
CENPN
CENPO
CENPP
CENPT
CENPU
CEP295NL
CEP72
CERKL
CERS3
CERS5
CETN2
CFAP157
CFAP20
CFAP43
CFHR3
CGRRF1
CHADL
CHCHD2
CHCHD4
CHCHD5
CHCHD6
CHEK2
CHFR
CHI3L2
CHIC1
CHIT1
CHL1
CHMP2B
CHMP5
CHODL
CHP1
CHRM3
CHRM4
CHRNA5
CHST11
CHTF18
CHTF8
CHTOP
CHURC1
CIB2
CIDEB
CILP
CINP
CISH
CKAP4
CLASP1
CLCN2
CLDN10
CLDN12
CLDN7
CLDND2
CLEC1A
CLEC4C
25% (each row)
Mutation Types
missense_variant
frameshift_variant
stop_gained
conservative_inframe_deletion
multi_hit

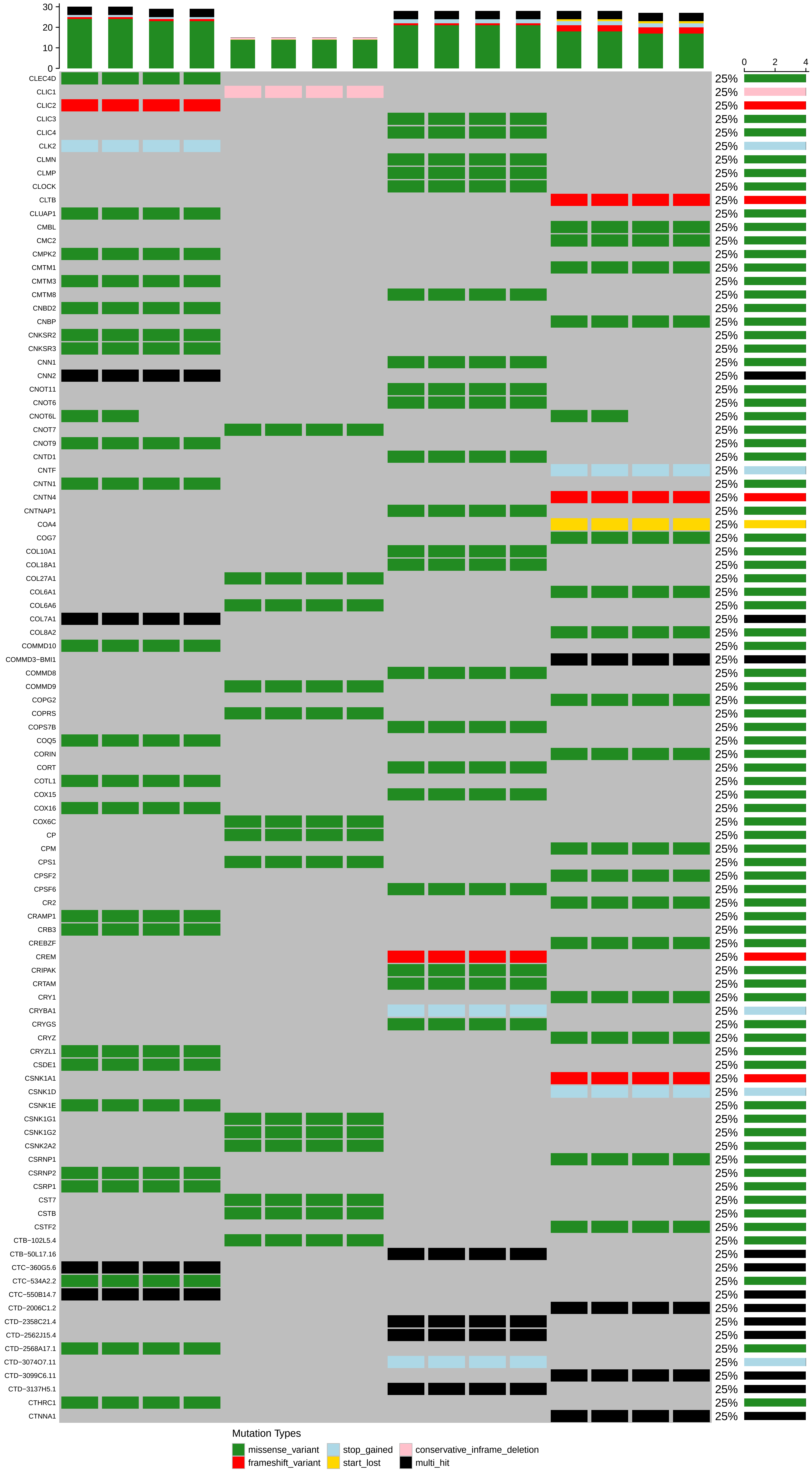

CLEC4D
CLIC1
CLIC2
CLIC3
CLIC4
CLK2
CLMN
CLMP
CLOCK
CLTB
CLUAP1
CMBL
CMC2
CMPK2
CMTM1
CMTM3
CMTM8
CNBD2
CNBP
CNKSR2
CNKSR3
CNN1
CNN2
CNOT11
CNOT6
CNOT6L
CNOT7
CNOT9
CNTD1
CNTF
CNTN1
CNTN4
CNTNAP1
COA4
COG7
COL10A1
COL18A1
COL27A1
COL6A1
COL6A6
COL7A1
COL8A2
COMMD10
COMMD3−BMI1
COMMD8
COMMD9
COPG2
COPRS
COPS7B
COQ5
CORIN
CORT
COTL1
COX15
COX16
COX6C
CP
CPM
CPS1
CPSF2
CPSF6
CR2
CRAMP1
CRB3
CREBZF
CREM
CRIPAK
CRTAM
CRY1
CRYBA1
CRYGS
CRYZ
CRYZL1
CSDE1
CSNK1A1
CSNK1D
CSNK1E
CSNK1G1
CSNK1G2
CSNK2A2
CSRNP1
CSRNP2
CSRP1
CST7
CSTB
CSTF2
CTB−102L5.4
CTB−50L17.16
CTC−360G5.6
CTC−534A2.2
CTC−550B14.7
CTD−2006C1.2
CTD−2358C21.4
CTD−2562J15.4
CTD−2568A17.1
CTD−3074O7.11
CTD−3099C6.11
CTD−3137H5.1
CTHRC1
CTNNA1
Mutation Types
missense_variant
frameshift_variant
stop_gained
start_lost
conservative_inframe_deletion
multi_hit

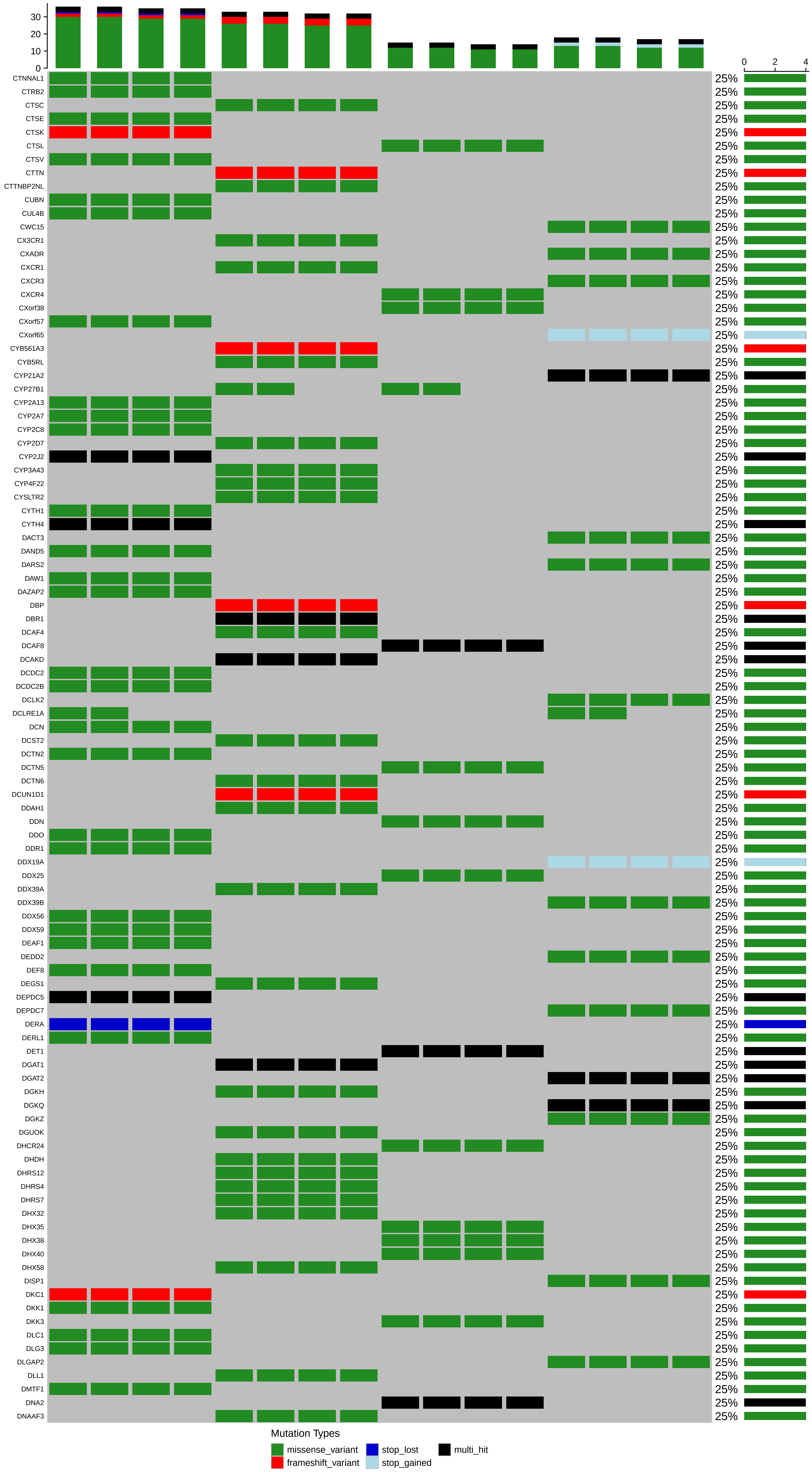
30
20
10
0
0
2
4
CTNNAL1
CTRB2
CTSC
CTSE
CTSK
CTSL
CTSV
CTTN
CTTNBP2NL
CUBN
CUL4B
CWC15
CX3CR1
CXADR
CXCR1
CXCR3
CXCR4
CXorf38
CXorf57
CXorf65
CYB561A3
CYB5RL
CYP21A2
CYP27B1
CYP2A13
CYP2A7
CYP2C8
CYP2D7
CYP2J2
CYP3A43
CYP4F22
CYSLTR2
CYTH1
CYTH4
DACT3
DAND5
DARS2
DAW1
DAZAP2
DBP
DBR1
DCAF4
DCAF8
DCAKD
DCDC2
DCDC2B
DCLK2
DCLRE1A
DCN
DCST2
DCTN2
DCTN5
DCTN6
DCUN1D1
DDAH1
DDN
DDO
DDR1
DDX19A
DDX25
DDX39A
DDX39B
DDX56
DDX59
DEAF1
DEDD2
DEF8
DEGS1
DEPDC5
DEPDC7
DERA
DERL1
DET1
DGAT1
DGAT2
DGKH
DGKQ
DGKZ
DGUOK
DHCR24
DHDH
DHRS12
DHRS4
DHRS7
DHX32
DHX35
DHX38
DHX40
DHX58
DISP1
DKC1
DKK1
DKK3
DLC1
DLG3
DLGAP2
DLL1
DMTF1
DNA2
DNAAF3
25% (each of the 100 genes)
Mutation Types
missense_variant
frameshift_variant
stop_lost
stop_gained
multi_hit

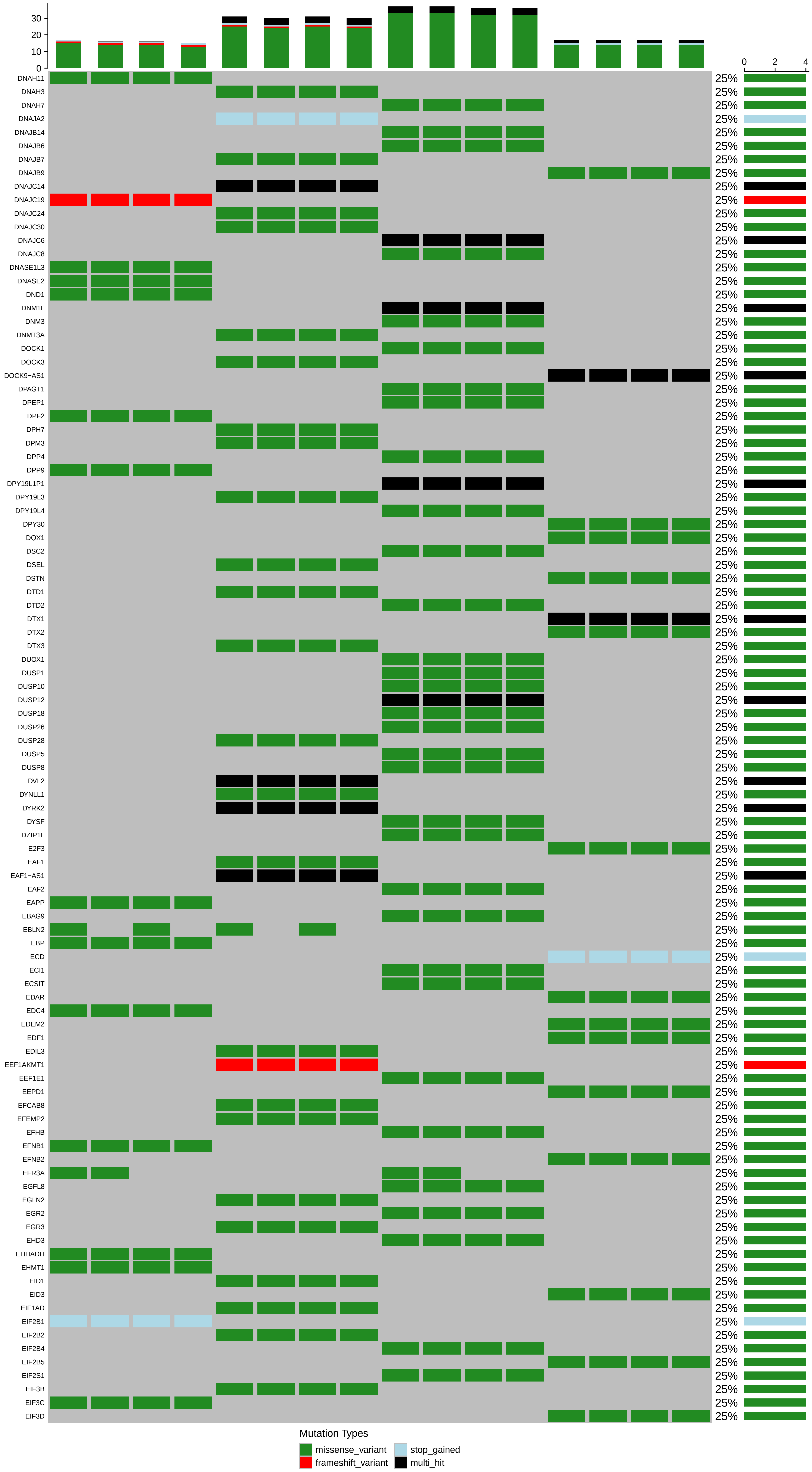

DNAH11
DNAH3
DNAH7
DNAJA2
DNAJB14
DNAJB6
DNAJB7
DNAJB9
DNAJC14
DNAJC19
DNAJC24
DNAJC30
DNAJC6
DNAJC8
DNASE1L3
DNASE2
DND1
DNM1L
DNM3
DNMT3A
DOCK1
DOCK3
DOCK9−AS1
DPAGT1
DPEP1
DPF2
DPH7
DPM3
DPP4
DPP9
DPY19L1P1
DPY19L3
DPY19L4
DPY30
DQX1
DSC2
DSEL
DSTN
DTD1
DTD2
DTX1
DTX2
DTX3
DUOX1
DUSP1
DUSP10
DUSP12
DUSP18
DUSP26
DUSP28
DUSP5
DUSP8
DVL2
DYNLL1
DYRK2
DYSF
DZIP1L
E2F3
EAF1
EAF1−AS1
EAF2
EAPP
EBAG9
EBLN2
EBP
ECD
ECI1
ECSIT
EDAR
EDC4
EDEM2
EDF1
EDIL3
EEF1AKMT1
EEF1E1
EEPD1
EFCAB8
EFEMP2
EFHB
EFNB1
EFNB2
EFR3A
EGFL8
EGLN2
EGR2
EGR3
EHD3
EHHADH
EHMT1
EID1
EID3
EIF1AD
EIF2B1
EIF2B2
EIF2B4
EIF2B5
EIF2S1
EIF3B
EIF3C
EIF3D
25%
0
10
20
30
0
2
4
Mutation Types
missense_variant
frameshift_variant
stop_gained
multi_hit

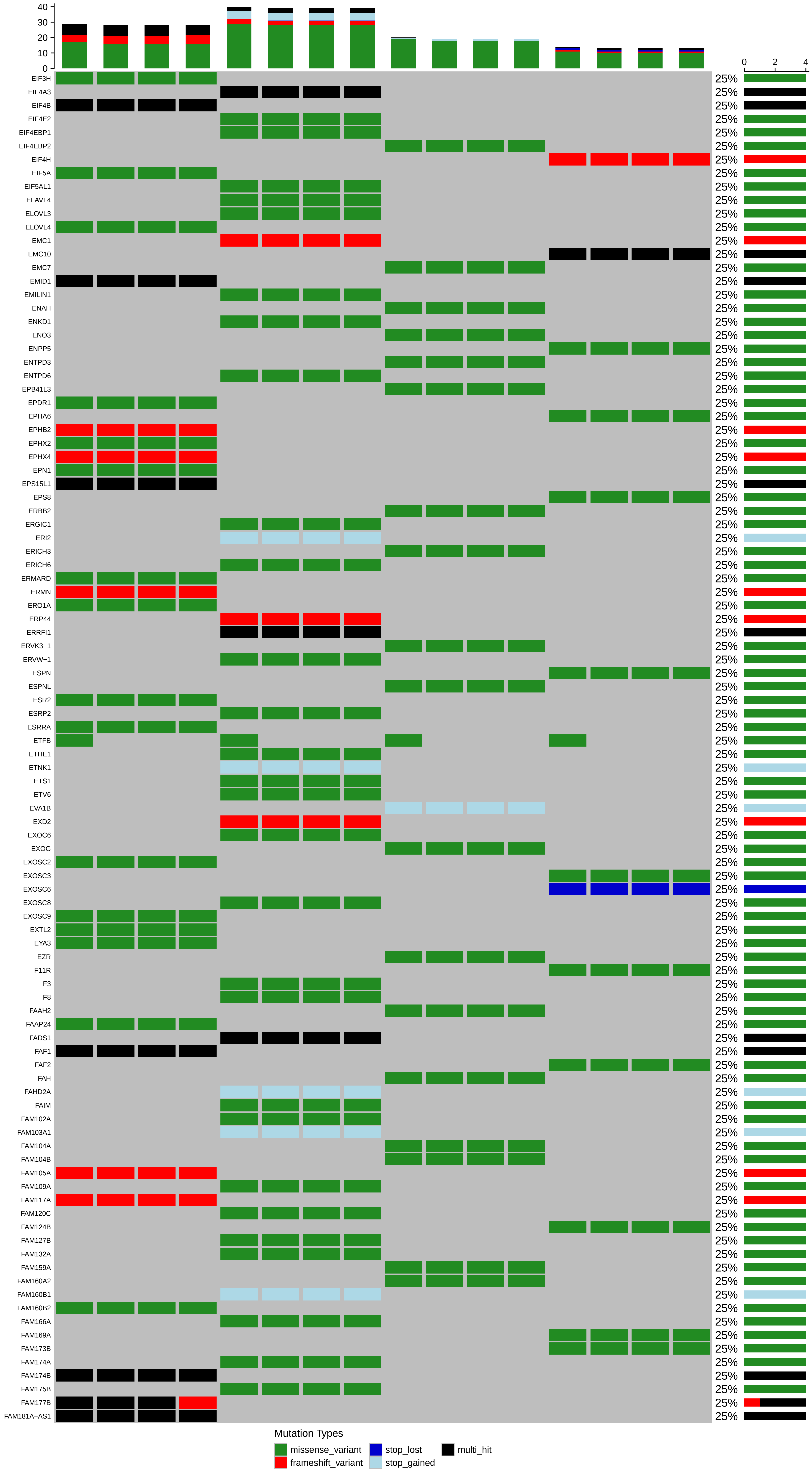

40
30
20
10
0
0 2 4
EIF3H
EIF4A3
EIF4B
EIF4E2
EIF4EBP1
EIF4EBP2
EIF4H
EIF5A
EIF5AL1
ELAVL4
ELOVL3
ELOVL4
EMC1
EMC10
EMC7
EMID1
EMILIN1
ENAH
ENKD1
ENO3
ENPP5
ENTPD3
ENTPD6
EPB41L3
EPDR1
EPHA6
EPHB2
EPHX2
EPHX4
EPN1
EPS15L1
EPS8
ERBB2
ERGIC1
ERI2
ERICH3
ERICH6
ERMARD
ERMN
ERO1A
ERP44
ERRFI1
ERVK3−1
ERVW−1
ESPN
ESPNL
ESR2
ESRP2
ESRRA
ETFB
ETHE1
ETNK1
ETS1
ETV6
EVA1B
EXD2
EXOC6
EXOG
EXOSC2
EXOSC3
EXOSC6
EXOSC8
EXOSC9
EXTL2
EYA3
EZR
F11R
F3
F8
FAAH2
FAAP24
FADS1
FAF1
FAF2
FAH
FAHD2A
FAIM
FAM102A
FAM103A1
FAM104A
FAM104B
FAM105A
FAM109A
FAM117A
FAM120C
FAM124B
FAM127B
FAM132A
FAM159A
FAM160A2
FAM160B1
FAM160B2
FAM166A
FAM169A
FAM173B
FAM174A
FAM174B
FAM175B
FAM177B
FAM181A−AS1
25%
Mutation Types
missense_variant
frameshift_variant
stop_lost
stop_gained
multi_hit

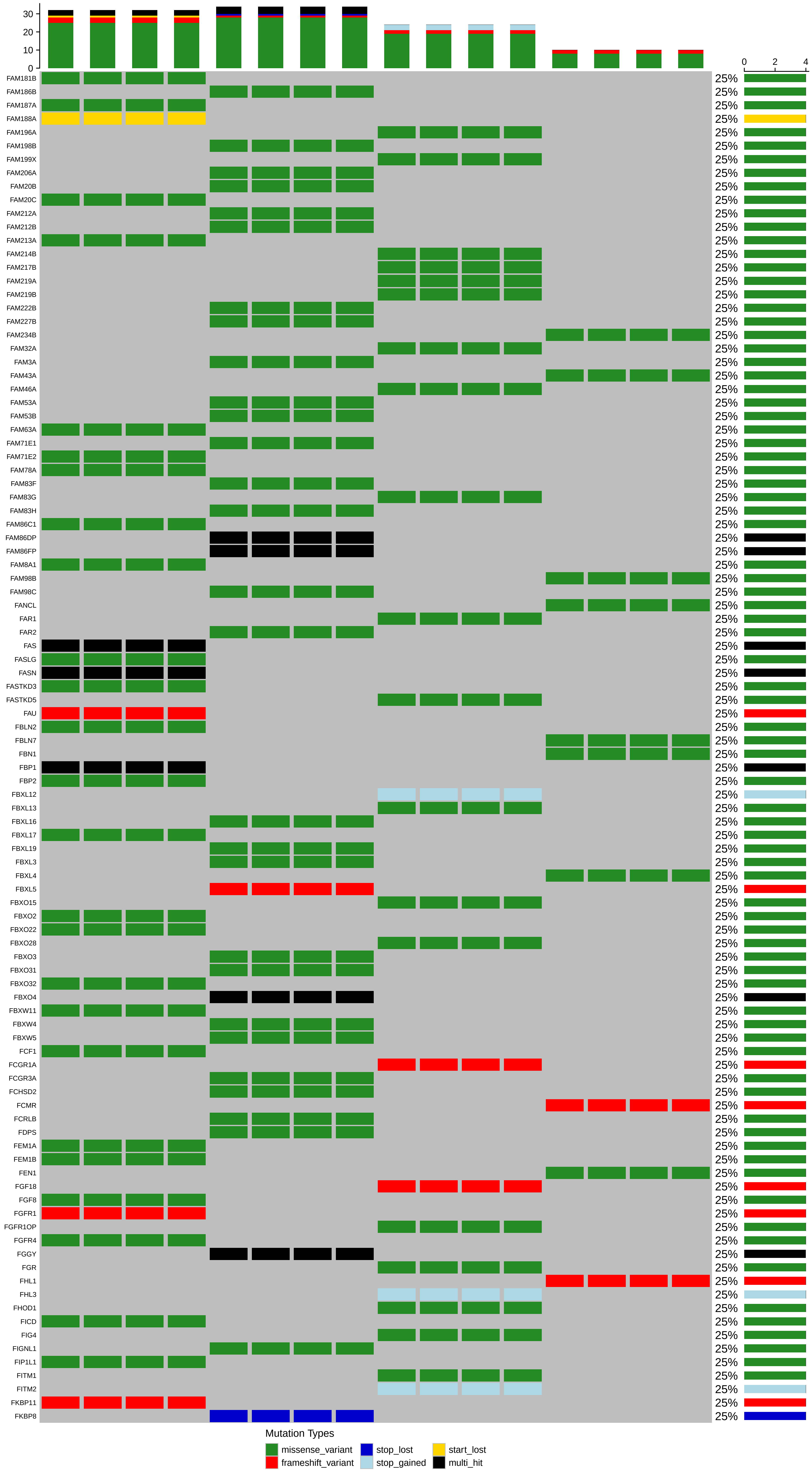
30
20
10
0
0
2
4
FAM181B
FAM186B
FAM187A
FAM188A
FAM196A
FAM198B
FAM199X
FAM206A
FAM20B
FAM20C
FAM212A
FAM212B
FAM213A
FAM214B
FAM217B
FAM219A
FAM219B
FAM222B
FAM227B
FAM234B
FAM32A
FAM3A
FAM43A
FAM46A
FAM53A
FAM53B
FAM63A
FAM71E1
FAM71E2
FAM78A
FAM83F
FAM83G
FAM83H
FAM86C1
FAM86DP
FAM86FP
FAM8A1
FAM98B
FAM98C
FANCL
FAR1
FAR2
FAS
FASLG
FASN
FASTKD3
FASTKD5
FAU
FBLN2
FBLN7
FBN1
FBP1
FBP2
FBXL12
FBXL13
FBXL16
FBXL17
FBXL19
FBXL3
FBXL4
FBXL5
FBXO15
FBXO2
FBXO22
FBXO28
FBXO3
FBXO31
FBXO32
FBXO4
FBXW11
FBXW4
FBXW5
FCF1
FCGR1A
FCGR3A
FCHSD2
FCMR
FCRLB
FDPS
FEM1A
FEM1B
FEN1
FGF18
FGF8
FGFR1
FGFR1OP
FGFR4
FGGY
FGR
FHL1
FHL3
FHOD1
FICD
FIG4
FIGNL1
FIP1L1
FITM1
FITM2
FKBP11
FKBP8
25% (on every row, 100 rows)
Mutation Types
missense_variant
frameshift_variant
stop_lost
stop_gained
start_lost
multi_hit

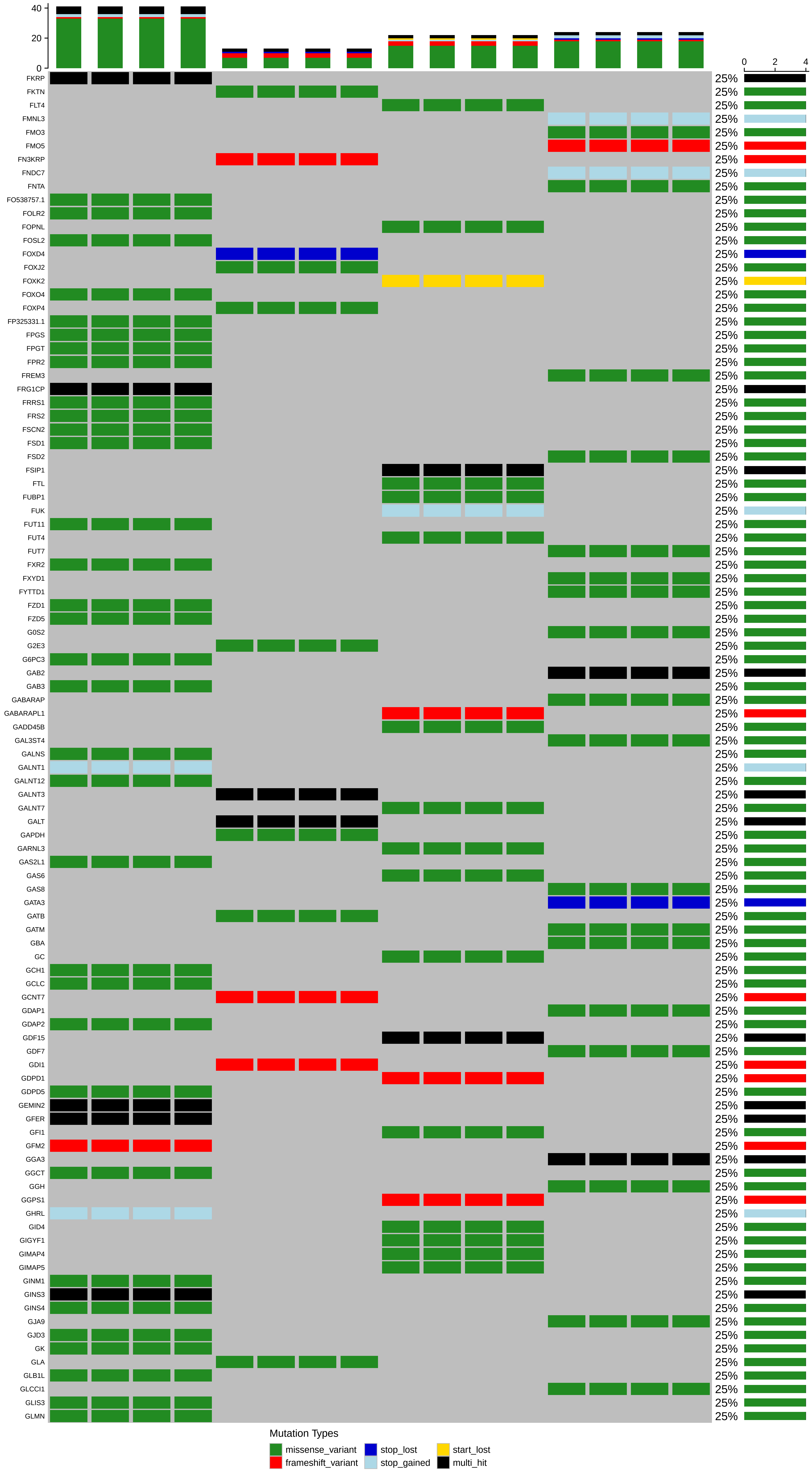
40
20
0
0
2
4
FKRP
FKTN
FLT4
FMNL3
FMO3
FMO5
FN3KRP
FNDC7
FNTA
FO538757.1
FOLR2
FOPNL
FOSL2
FOXD4
FOXJ2
FOXK2
FOXO4
FOXP4
FP325331.1
FPGS
FPGT
FPR2
FREM3
FRG1CP
FRRS1
FRS2
FSCN2
FSD1
FSD2
FSIP1
FTL
FUBP1
FUK
FUT11
FUT4
FUT7
FXR2
FXYD1
FYTTD1
FZD1
FZD5
G0S2
G2E3
G6PC3
GAB2
GAB3
GABARAP
GABARAPL1
GADD45B
GAL3ST4
GALNS
GALNT1
GALNT12
GALNT3
GALNT7
GALT
GAPDH
GARNL3
GAS2L1
GAS6
GAS8
GATA3
GATB
GATM
GBA
GC
GCH1
GCLC
GCNT7
GDAP1
GDAP2
GDF15
GDF7
GDI1
GDPD1
GDPD5
GEMIN2
GFER
GFI1
GFM2
GGA3
GGCT
GGH
GGPS1
GHRL
GID4
GIGYF1
GIMAP4
GIMAP5
GINM1
GINS3
GINS4
GJA9
GJD3
GK
GLA
GLB1L
GLCCI1
GLIS3
GLMN
25% (each of the 100 genes)
Mutation Types
missense_variant
frameshift_variant
stop_lost
stop_gained
start_lost
multi_hit

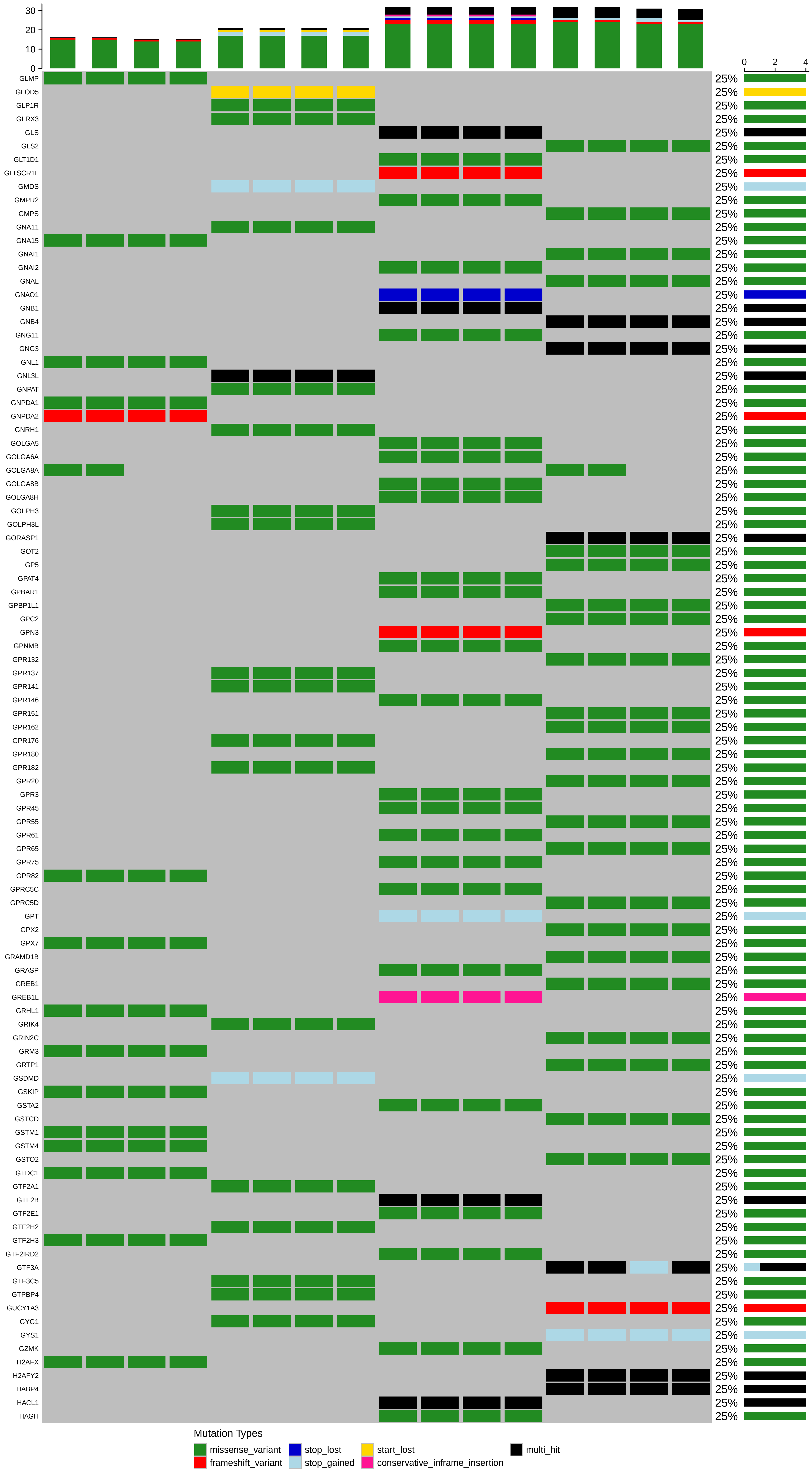

30
20
10
0
0 2 4
GLMP
GLOD5
GLP1R
GLRX3
GLS
GLS2
GLT1D1
GLTSCR1L
GMDS
GMPR2
GMPS
GNA11
GNA15
GNAI1
GNAI2
GNAL
GNAO1
GNB1
GNB4
GNG11
GNG3
GNL1
GNL3L
GNPAT
GNPDA1
GNPDA2
GNRH1
GOLGA5
GOLGA6A
GOLGA8A
GOLGA8B
GOLGA8H
GOLPH3
GOLPH3L
GORASP1
GOT2
GP5
GPAT4
GPBAR1
GPBP1L1
GPC2
GPN3
GPNMB
GPR132
GPR137
GPR141
GPR146
GPR151
GPR162
GPR176
GPR180
GPR182
GPR20
GPR3
GPR45
GPR55
GPR61
GPR65
GPR75
GPR82
GPRC5C
GPRC5D
GPT
GPX2
GPX7
GRAMD1B
GRASP
GREB1
GREB1L
GRHL1
GRIK4
GRIN2C
GRM3
GRTP1
GSDMD
GSKIP
GSTA2
GSTCD
GSTM1
GSTM4
GSTO2
GTDC1
GTF2A1
GTF2B
GTF2E1
GTF2H2
GTF2H3
GTF2IRD2
GTF3A
GTF3C5
GTPBP4
GUCY1A3
GYG1
GYS1
GZMK
H2AFX
H2AFY2
HABP4
HACL1
HAGH
25% (each gene, 97 rows)
Mutation Types
missense_variant
frameshift_variant
stop_lost
stop_gained
start_lost
conservative_inframe_insertion
multi_hit

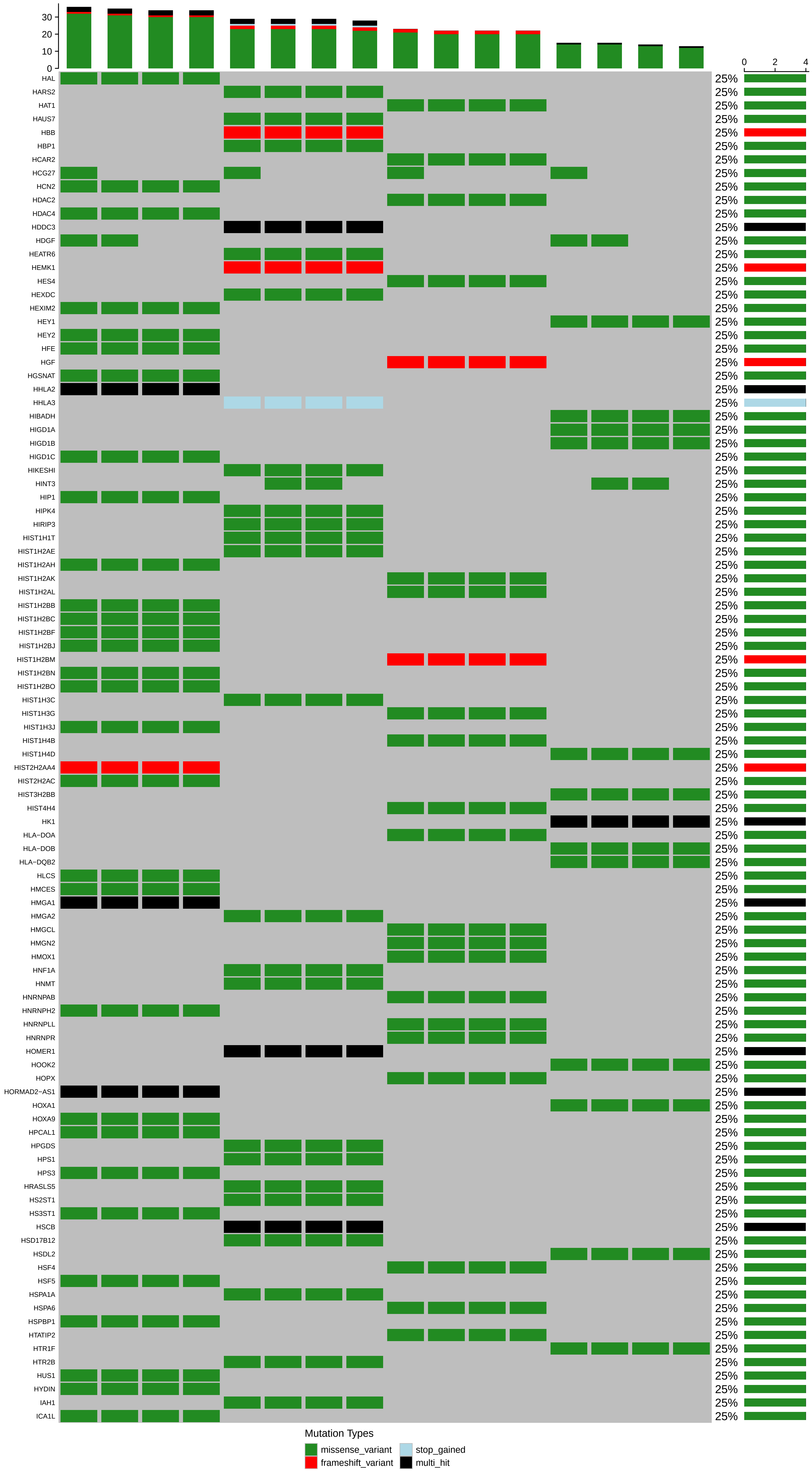

HAL
HARS2
HAT1
HAUS7
HBB
HBP1
HCAR2
HCG27
HCN2
HDAC2
HDAC4
HDDC3
HDGF
HEATR6
HEMK1
HES4
HEXDC
HEXIM2
HEY1
HEY2
HFE
HGF
HGSNAT
HHLA2
HHLA3
HIBADH
HIGD1A
HIGD1B
HIGD1C
HIKESHI
HINT3
HIP1
HIPK4
HIRIP3
HIST1H1T
HIST1H2AE
HIST1H2AH
HIST1H2AK
HIST1H2AL
HIST1H2BB
HIST1H2BC
HIST1H2BF
HIST1H2BJ
HIST1H2BM
HIST1H2BN
HIST1H2BO
HIST1H3C
HIST1H3G
HIST1H3J
HIST1H4B
HIST1H4D
HIST2H2AA4
HIST2H2AC
HIST3H2BB
HIST4H4
HK1
HLA−DOA
HLA−DOB
HLA−DQB2
HLCS
HMCES
HMGA1
HMGA2
HMGCL
HMGN2
HMOX1
HNF1A
HNMT
HNRNPAB
HNRNPH2
HNRNPLL
HNRNPR
HOMER1
HOOK2
HOPX
HORMAD2−AS1
HOXA1
HOXA9
HPCAL1
HPGDS
HPS1
HPS3
HRASLS5
HS2ST1
HS3ST1
HSCB
HSD17B12
HSDL2
HSF4
HSF5
HSPA1A
HSPA6
HSPBP1
HTATIP2
HTR1F
HTR2B
HUS1
HYDIN
IAH1
ICA1L
25%
0
10
20
30
0
2
4
Mutation Types
missense_variant
frameshift_variant
stop_gained
multi_hit

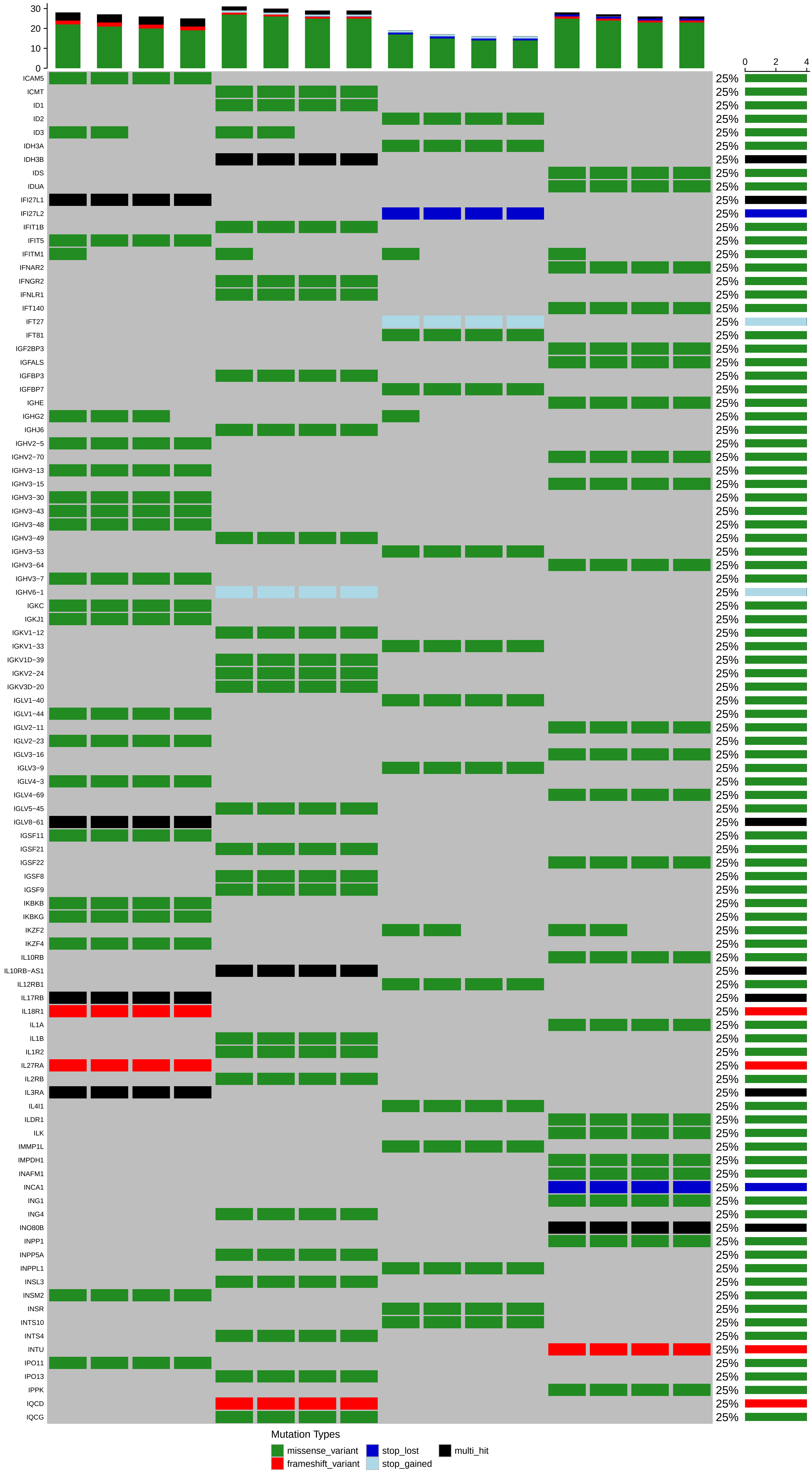
ICAM5
ICMT
ID1
ID2
ID3
IDH3A
IDH3B
IDS
IDUA
IFI27L1
IFI27L2
IFIT1B
IFIT5
IFITM1
IFNAR2
IFNGR2
IFNLR1
IFT140
IFT27
IFT81
IGF2BP3
IGFALS
IGFBP3
IGFBP7
IGHE
IGHG2
IGHJ6
IGHV2−5
IGHV2−70
IGHV3−13
IGHV3−15
IGHV3−30
IGHV3−43
IGHV3−48
IGHV3−49
IGHV3−53
IGHV3−64
IGHV3−7
IGHV6−1
IGKC
IGKJ1
IGKV1−12
IGKV1−33
IGKV1D−39
IGKV2−24
IGKV3D−20
IGLV1−40
IGLV1−44
IGLV2−11
IGLV2−23
IGLV3−16
IGLV3−9
IGLV4−3
IGLV4−69
IGLV5−45
IGLV8−61
IGSF11
IGSF21
IGSF22
IGSF8
IGSF9
IKBKB
IKBKG
IKZF2
IKZF4
IL10RB
IL10RB−AS1
IL12RB1
IL17RB
IL18R1
IL1A
IL1B
IL1R2
IL27RA
IL2RB
IL3RA
IL4I1
ILDR1
ILK
IMMP1L
IMPDH1
INAFM1
INCA1
ING1
ING4
INO80B
INPP1
INPP5A
INPPL1
INSL3
INSM2
INSR
INTS10
INTS4
INTU
IPO11
IPO13
IPPK
IQCD
IQCG
25%
Mutation Types
missense_variant
frameshift_variant
stop_lost
stop_gained
multi_hit

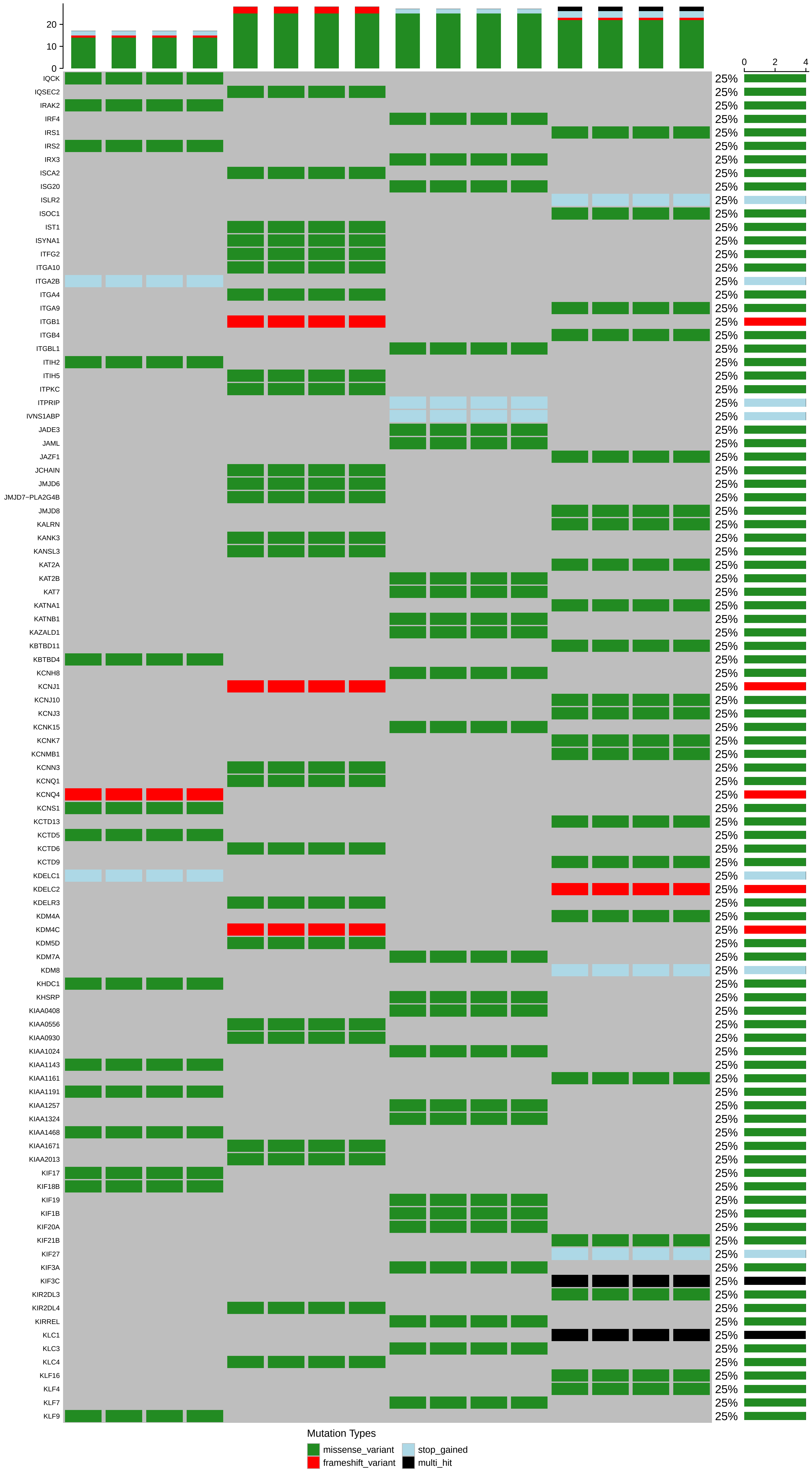

Mutation Types
missense_variant
frameshift_variant
stop_gained
multi_hit

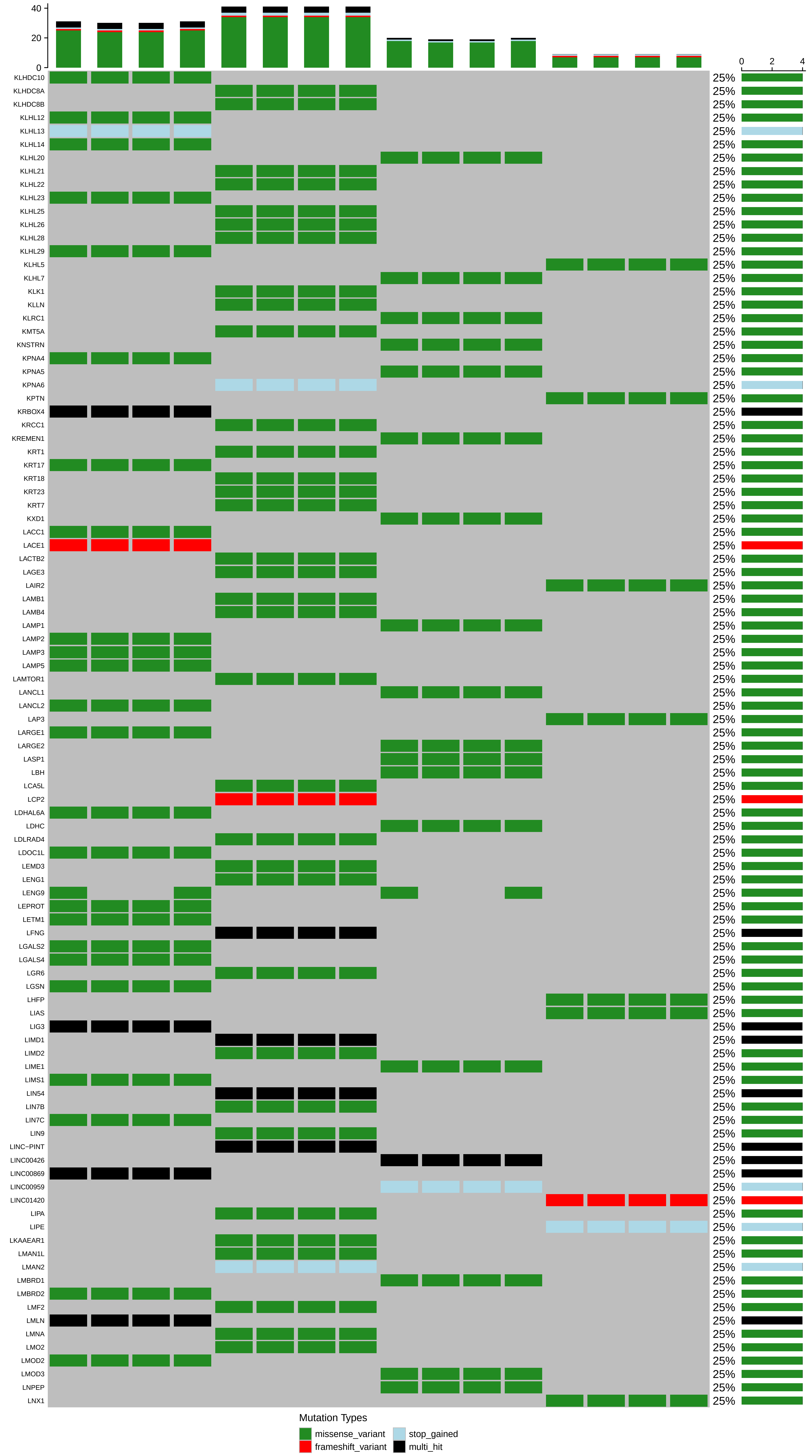

40
20
0
0
2
4
KLHDC10
KLHDC8A
KLHDC8B
KLHL12
KLHL13
KLHL14
KLHL20
KLHL21
KLHL22
KLHL23
KLHL25
KLHL26
KLHL28
KLHL29
KLHL5
KLHL7
KLK1
KLLN
KLRC1
KMT5A
KNSTRN
KPNA4
KPNA5
KPNA6
KPTN
KRBOX4
KRCC1
KREMEN1
KRT1
KRT17
KRT18
KRT23
KRT7
KXD1
LACC1
LACE1
LACTB2
LAGE3
LAIR2
LAMB1
LAMB4
LAMP1
LAMP2
LAMP3
LAMP5
LAMTOR1
LANCL1
LANCL2
LAP3
LARGE1
LARGE2
LASP1
LBH
LCA5L
LCP2
LDHAL6A
LDHC
LDLRAD4
LDOC1L
LEMD3
LENG1
LENG9
LEPROT
LETM1
LFNG
LGALS2
LGALS4
LGR6
LGSN
LHFP
LIAS
LIG3
LIMD1
LIMD2
LIME1
LIMS1
LIN54
LIN7B
LIN7C
LIN9
LINC−PINT
LINC00426
LINC00869
LINC00959
LINC01420
LIPA
LIPE
LKAAEAR1
LMAN1L
LMAN2
LMBRD1
LMBRD2
LMF2
LMLN
LMNA
LMO2
LMOD2
LMOD3
LNPEP
LNX1
25%
Mutation Types
missense_variant
frameshift_variant
stop_gained
multi_hit

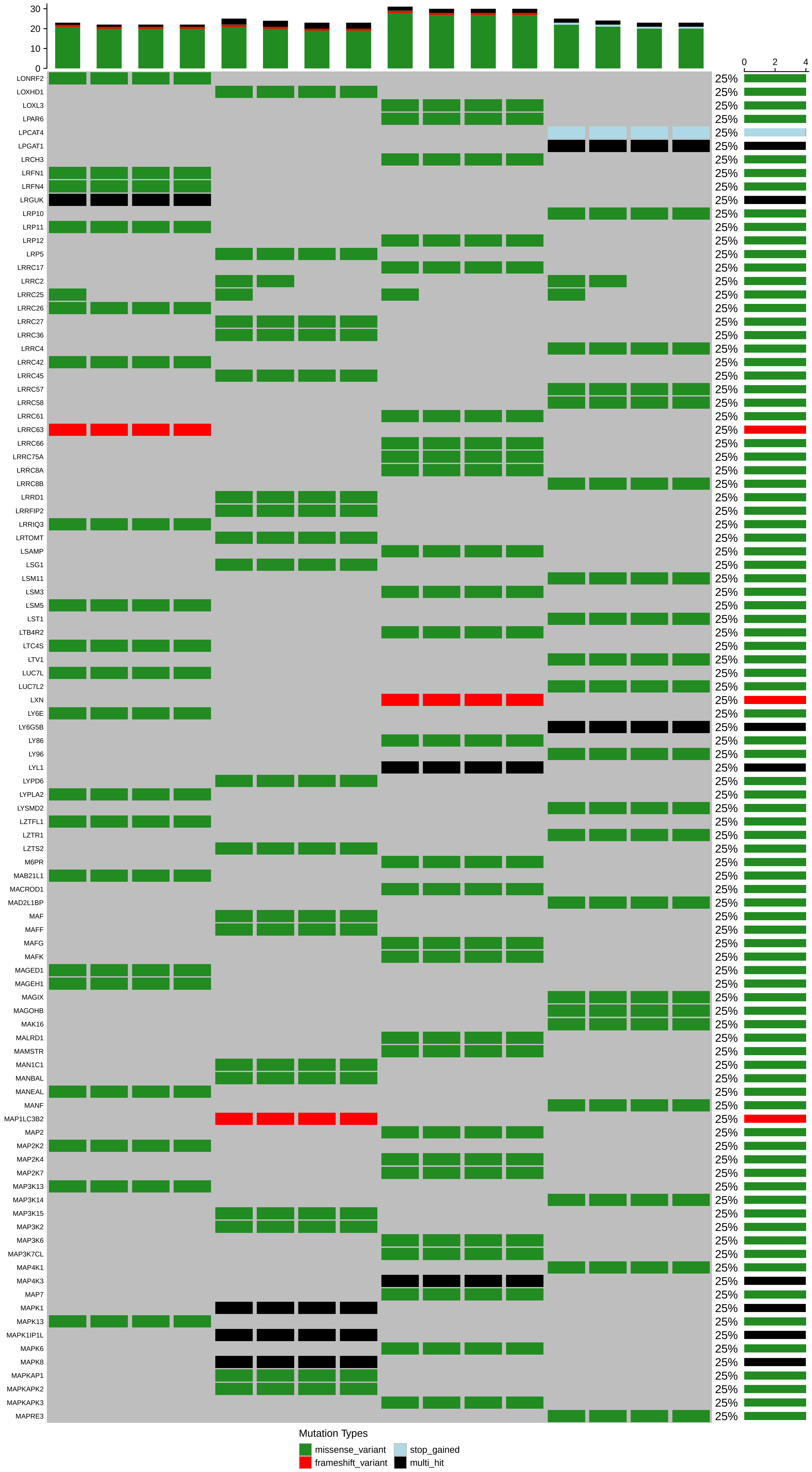

30
20
10
0
0
2
4
LONRF2
LOXHD1
LOXL3
LPAR6
LPCAT4
LPGAT1
LRCH3
LRFN1
LRFN4
LRGUK
LRP10
LRP11
LRP12
LRP5
LRRC17
LRRC2
LRRC25
LRRC26
LRRC27
LRRC36
LRRC4
LRRC42
LRRC45
LRRC57
LRRC58
LRRC61
LRRC63
LRRC66
LRRC75A
LRRC8A
LRRC8B
LRRD1
LRRFIP2
LRRIQ3
LRTOMT
LSAMP
LSG1
LSM11
LSM3
LSM5
LST1
LTB4R2
LTC4S
LTV1
LUC7L
LUC7L2
LXN
LY6E
LY6G5B
LY86
LY96
LYL1
LYPD6
LYPLA2
LYSMD2
LZTFL1
LZTR1
LZTS2
M6PR
MAB21L1
MACROD1
MAD2L1BP
MAF
MAFF
MAFG
MAFK
MAGED1
MAGEH1
MAGIX
MAGOHB
MAK16
MALRD1
MAMSTR
MAN1C1
MANBAL
MANEAL
MANF
MAP1LC3B2
MAP2
MAP2K2
MAP2K4
MAP2K7
MAP3K13
MAP3K14
MAP3K15
MAP3K2
MAP3K6
MAP3K7CL
MAP4K1
MAP4K3
MAP7
MAPK1
MAPK13
MAPK1IP1L
MAPK6
MAPK8
MAPKAP1
MAPKAPK2
MAPKAPK3
MAPRE3
25%
Mutation Types
missense_variant
frameshift_variant
stop_gained
multi_hit

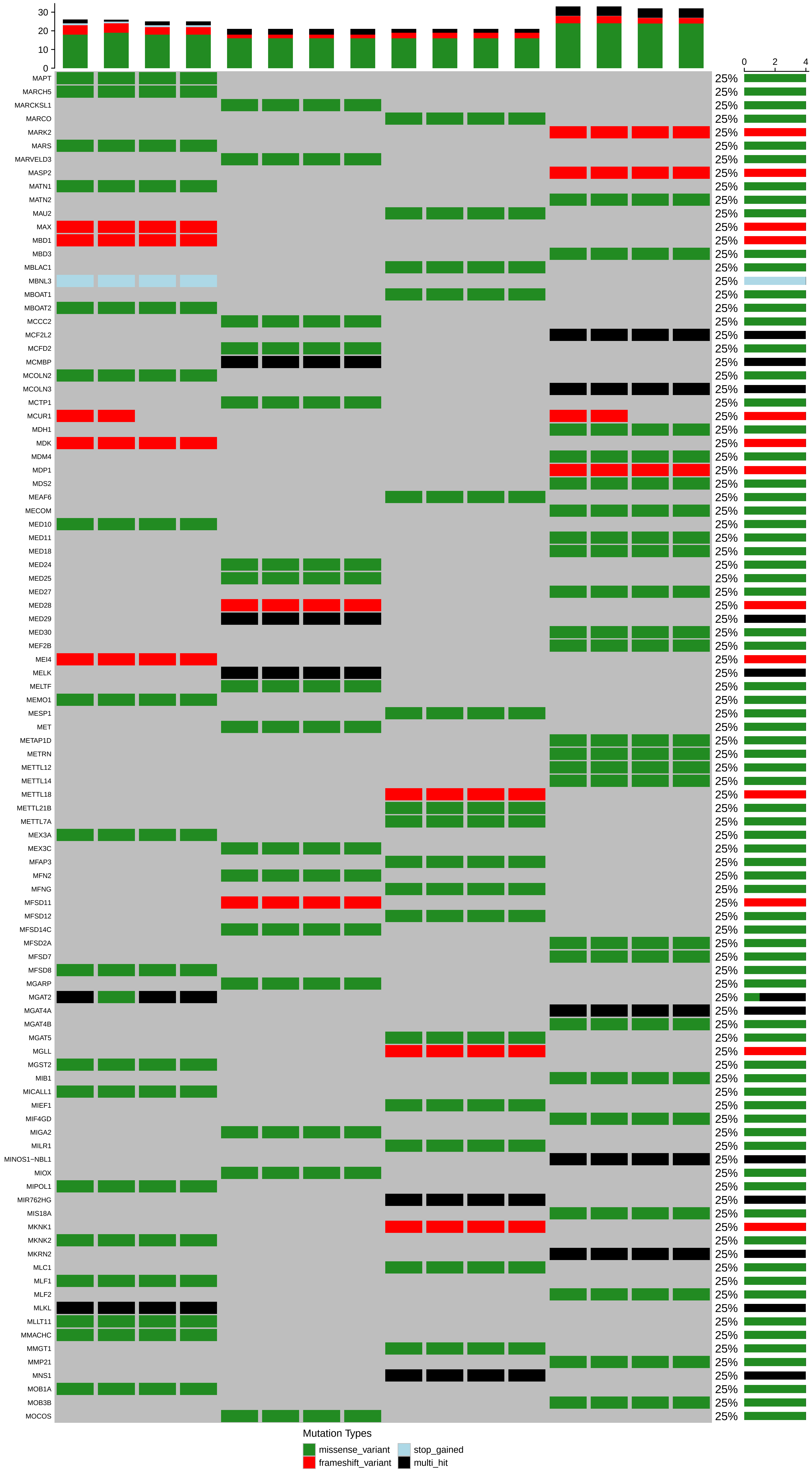

MAPT
MARCH5
MARCKSL1
MARCO
MARK2
MARS
MARVELD3
MASP2
MATN1
MATN2
MAU2
MAX
MBD1
MBD3
MBLAC1
MBNL3
MBOAT1
MBOAT2
MCCC2
MCF2L2
MCFD2
MCMBP
MCOLN2
MCOLN3
MCTP1
MCUR1
MDH1
MDK
MDM4
MDP1
MDS2
MEAF6
MECOM
MED10
MED11
MED18
MED24
MED25
MED27
MED28
MED29
MED30
MEF2B
MEI4
MELK
MELTF
MEMO1
MESP1
MET
METAP1D
METRN
METTL12
METTL14
METTL18
METTL21B
METTL7A
MEX3A
MEX3C
MFAP3
MFN2
MFNG
MFSD11
MFSD12
MFSD14C
MFSD2A
MFSD7
MFSD8
MGARP
MGAT2
MGAT4A
MGAT4B
MGAT5
MGLL
MGST2
MIB1
MICALL1
MIEF1
MIF4GD
MIGA2
MILR1
MINOS1−NBL1
MIOX
MIPOL1
MIR762HG
MIS18A
MKNK1
MKNK2
MKRN2
MLC1
MLF1
MLF2
MLKL
MLLT11
MMACHC
MMGT1
MMP21
MNS1
MOB1A
MOB3B
MOCOS
25%
Mutation Types
missense_variant
frameshift_variant
stop_gained
multi_hit

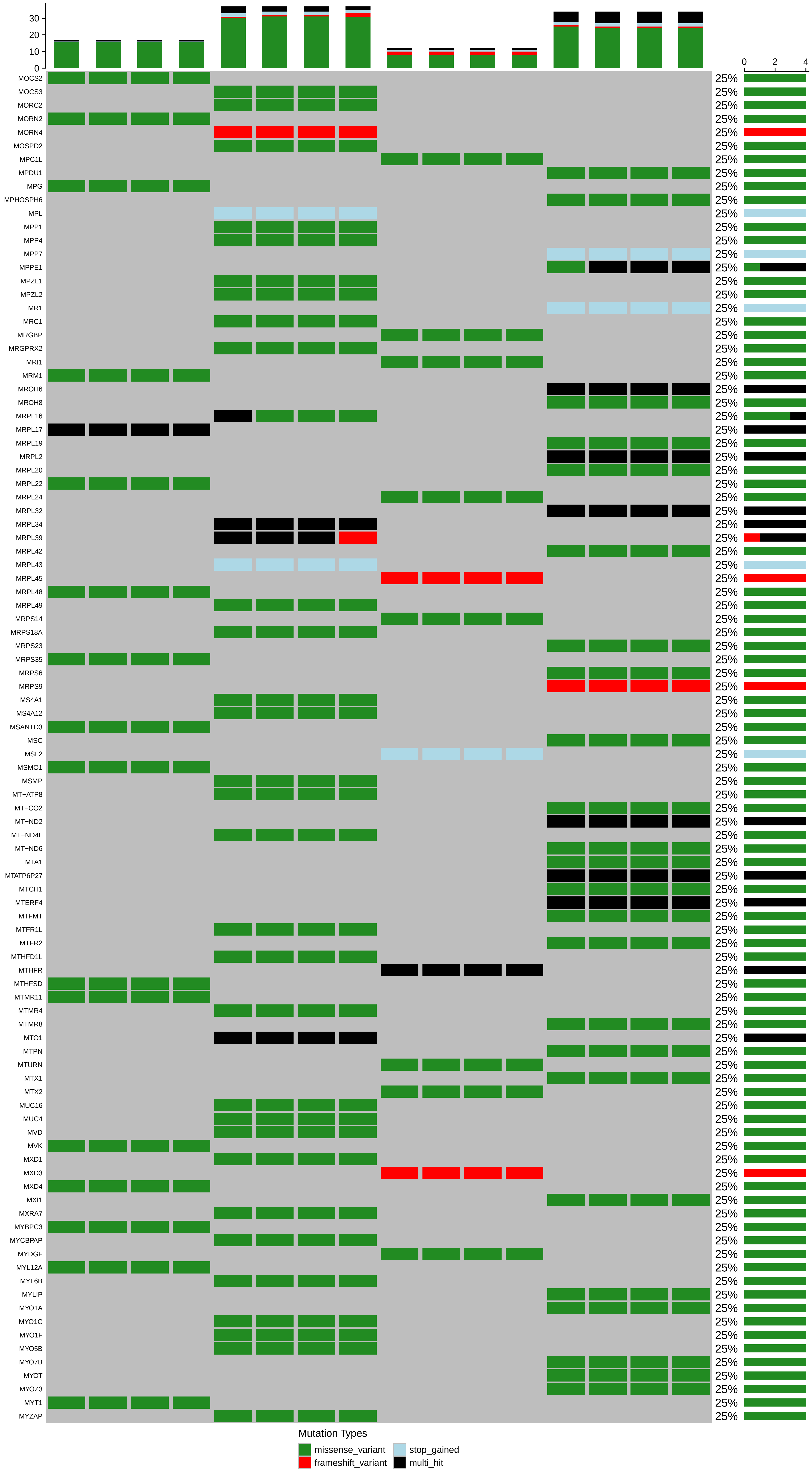
30
20
10
0
0
2
4
MOCS2
MOCS3
MORC2
MORN2
MORN4
MOSPD2
MPC1L
MPDU1
MPG
MPHOSPH6
MPL
MPP1
MPP4
MPP7
MPPE1
MPZL1
MPZL2
MR1
MRC1
MRGBP
MRGPRX2
MRI1
MRM1
MROH6
MROH8
MRPL16
MRPL17
MRPL19
MRPL2
MRPL20
MRPL22
MRPL24
MRPL32
MRPL34
MRPL39
MRPL42
MRPL43
MRPL45
MRPL48
MRPL49
MRPS14
MRPS18A
MRPS23
MRPS35
MRPS6
MRPS9
MS4A1
MS4A12
MSANTD3
MSC
MSL2
MSMO1
MSMP
MT−ATP8
MT−CO2
MT−ND2
MT−ND4L
MT−ND6
MTA1
MTATP6P27
MTCH1
MTERF4
MTFMT
MTFR1L
MTFR2
MTHFD1L
MTHFR
MTHFSD
MTMR11
MTMR4
MTMR8
MTO1
MTPN
MTURN
MTX1
MTX2
MUC16
MUC4
MVD
MVK
MXD1
MXD3
MXD4
MXI1
MXRA7
MYBPC3
MYCBPAP
MYDGF
MYL12A
MYL6B
MYLIP
MYO1A
MYO1C
MYO1F
MYO5B
MYO7B
MYOT
MYOZ3
MYT1
MYZAP
25%
Mutation Types
missense_variant
frameshift_variant
stop_gained
multi_hit

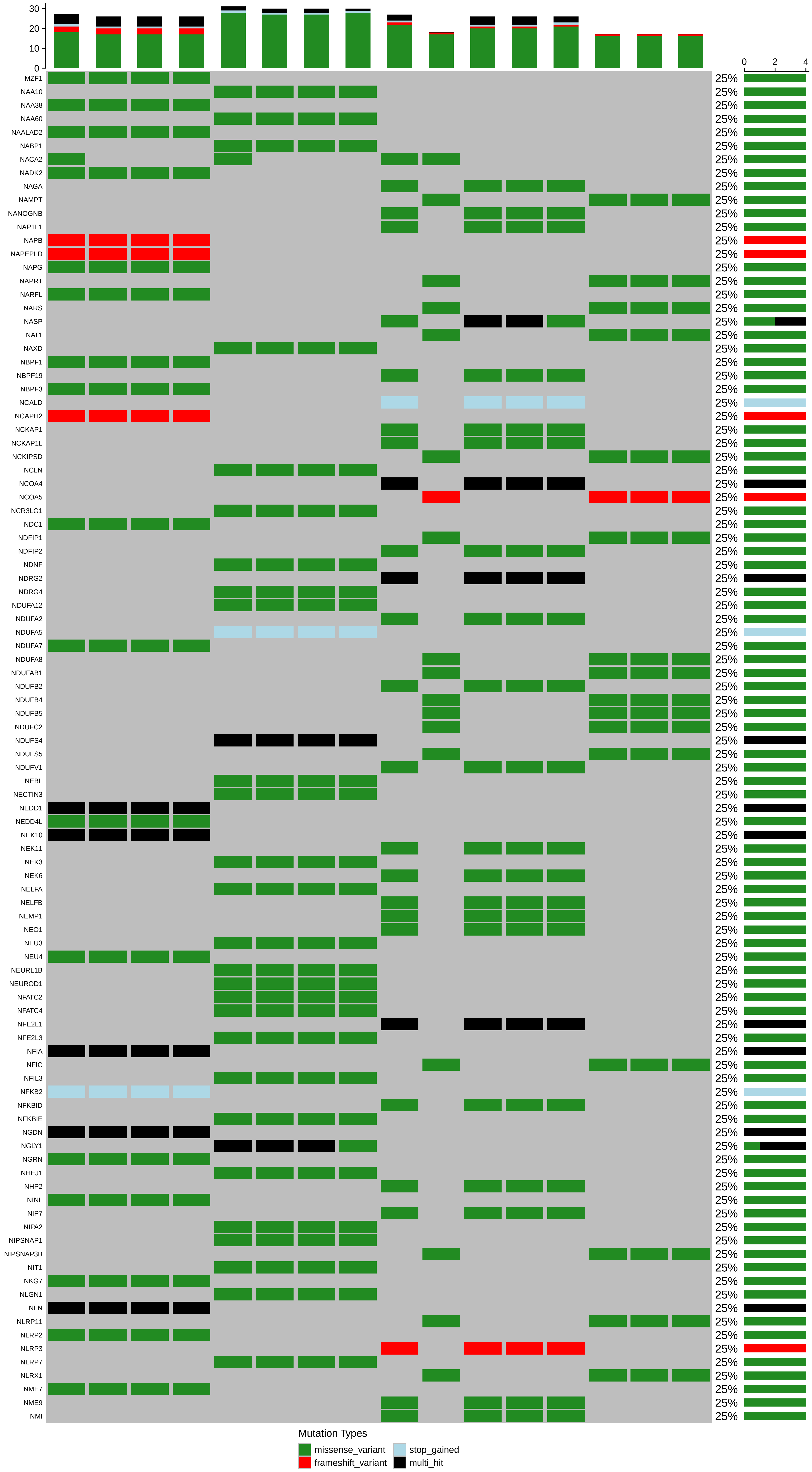

MZF1
NAA10
NAA38
NAA60
NAALAD2
NABP1
NACA2
NADK2
NAGA
NAMPT
NANOGNB
NAP1L1
NAPB
NAPEPLD
NAPG
NAPRT
NARFL
NARS
NASP
NAT1
NAXD
NBPF1
NBPF19
NBPF3
NCALD
NCAPH2
NCKAP1
NCKAP1L
NCKIPSD
NCLN
NCOA4
NCOA5
NCR3LG1
NDC1
NDFIP1
NDFIP2
NDNF
NDRG2
NDRG4
NDUFA12
NDUFA2
NDUFA5
NDUFA7
NDUFA8
NDUFAB1
NDUFB2
NDUFB4
NDUFB5
NDUFC2
NDUFS4
NDUFS5
NDUFV1
NEBL
NECTIN3
NEDD1
NEDD4L
NEK10
NEK11
NEK3
NEK6
NELFA
NELFB
NEMP1
NEO1
NEU3
NEU4
NEURL1B
NEUROD1
NFATC2
NFATC4
NFE2L1
NFE2L3
NFIA
NFIC
NFIL3
NFKB2
NFKBID
NFKBIE
NGDN
NGLY1
NGRN
NHEJ1
NHP2
NINL
NIP7
NIPA2
NIPSNAP1
NIPSNAP3B
NIT1
NKG7
NLGN1
NLN
NLRP11
NLRP2
NLRP3
NLRP7
NLRX1
NME7
NME9
NMI
Mutation Types
missense_variant
frameshift_variant
stop_gained
multi_hit

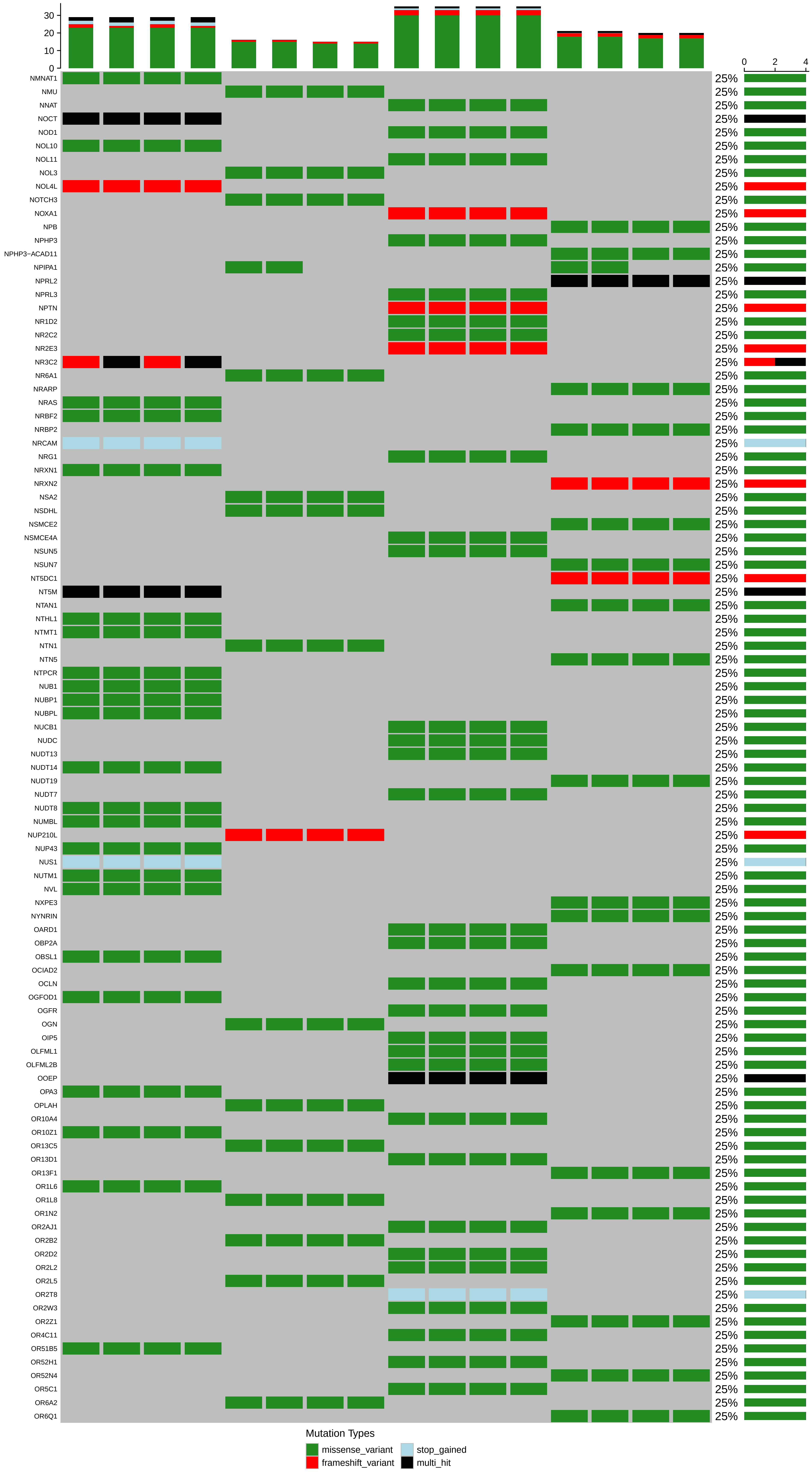

30
20
10
0
0
2
4
NMNAT1
NMU
NNAT
NOCT
NOD1
NOL10
NOL11
NOL3
NOL4L
NOTCH3
NOXA1
NPB
NPHP3
NPHP3−ACAD11
NPIPA1
NPRL2
NPRL3
NPTN
NR1D2
NR2C2
NR2E3
NR3C2
NR6A1
NRARP
NRAS
NRBF2
NRBP2
NRCAM
NRG1
NRXN1
NRXN2
NSA2
NSDHL
NSMCE2
NSMCE4A
NSUN5
NSUN7
NT5DC1
NT5M
NTAN1
NTHL1
NTMT1
NTN1
NTN5
NTPCR
NUB1
NUBP1
NUBPL
NUCB1
NUDC
NUDT13
NUDT14
NUDT19
NUDT7
NUDT8
NUMBL
NUP210L
NUP43
NUS1
NUTM1
NVL
NXPE3
NYNRIN
OARD1
OBP2A
OBSL1
OCIAD2
OCLN
OGFOD1
OGFR
OGN
OIP5
OLFML1
OLFML2B
OOEP
OPA3
OPLAH
OR10A4
OR10Z1
OR13C5
OR13D1
OR13F1
OR1L6
OR1L8
OR1N2
OR2AJ1
OR2B2
OR2D2
OR2L2
OR2L5
OR2T8
OR2W3
OR2Z1
OR4C11
OR51B5
OR52H1
OR52N4
OR5C1
OR6A2
OR6Q1
25%
Mutation Types
missense_variant
frameshift_variant
stop_gained
multi_hit

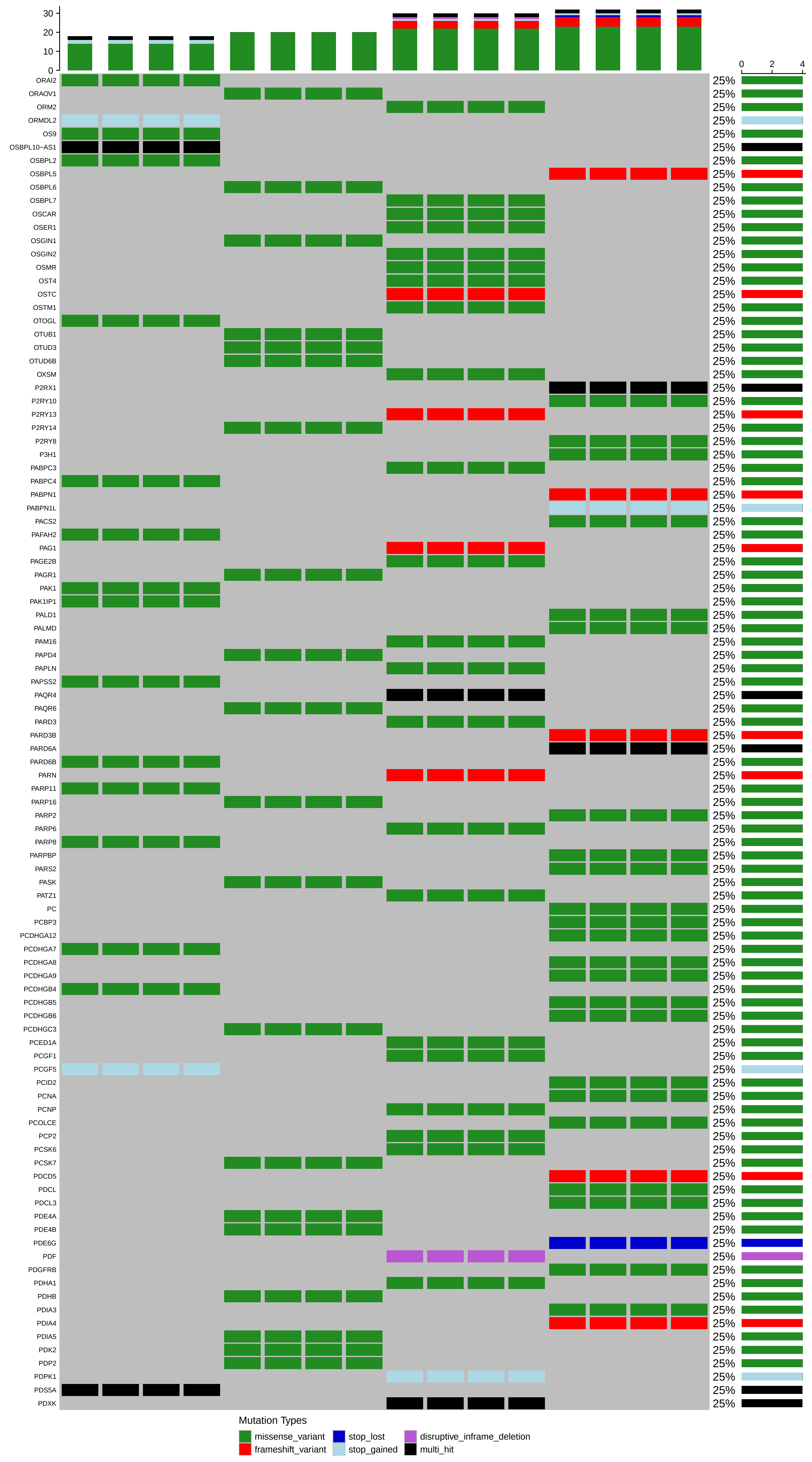

ORAI2
ORAOV1
ORM2
ORMDL2
OS9
OSBPL10−AS1
OSBPL2
OSBPL5
OSBPL6
OSBPL7
OSCAR
OSER1
OSGIN1
OSGIN2
OSMR
OST4
OSTC
OSTM1
OTOGL
OTUB1
OTUD3
OTUD6B
OXSM
P2RX1
P2RY10
P2RY13
P2RY14
P2RY8
P3H1
PABPC3
PABPC4
PABPN1
PABPN1L
PACS2
PAFAH2
PAG1
PAGE2B
PAGR1
PAK1
PAK1IP1
PALD1
PALMD
PAM16
PAPD4
PAPLN
PAPSS2
PAQR4
PAQR6
PARD3
PARD3B
PARD6A
PARD6B
PARN
PARP11
PARP16
PARP2
PARP6
PARP8
PARPBP
PARS2
PASK
PATZ1
PC
PCBP3
PCDHGA12
PCDHGA7
PCDHGA8
PCDHGA9
PCDHGB4
PCDHGB5
PCDHGB6
PCDHGC3
PCED1A
PCGF1
PCGF5
PCID2
PCNA
PCNP
PCOLCE
PCP2
PCSK6
PCSK7
PDCD5
PDCL
PDCL3
PDE4A
PDE4B
PDE6G
PDF
PDGFRB
PDHA1
PDHB
PDIA3
PDIA4
PDIA5
PDK2
PDP2
PDPK1
PDS5A
PDXK
Mutation Types
missense_variant
frameshift_variant
stop_lost
stop_gained
disruptive_inframe_deletion
multi_hit

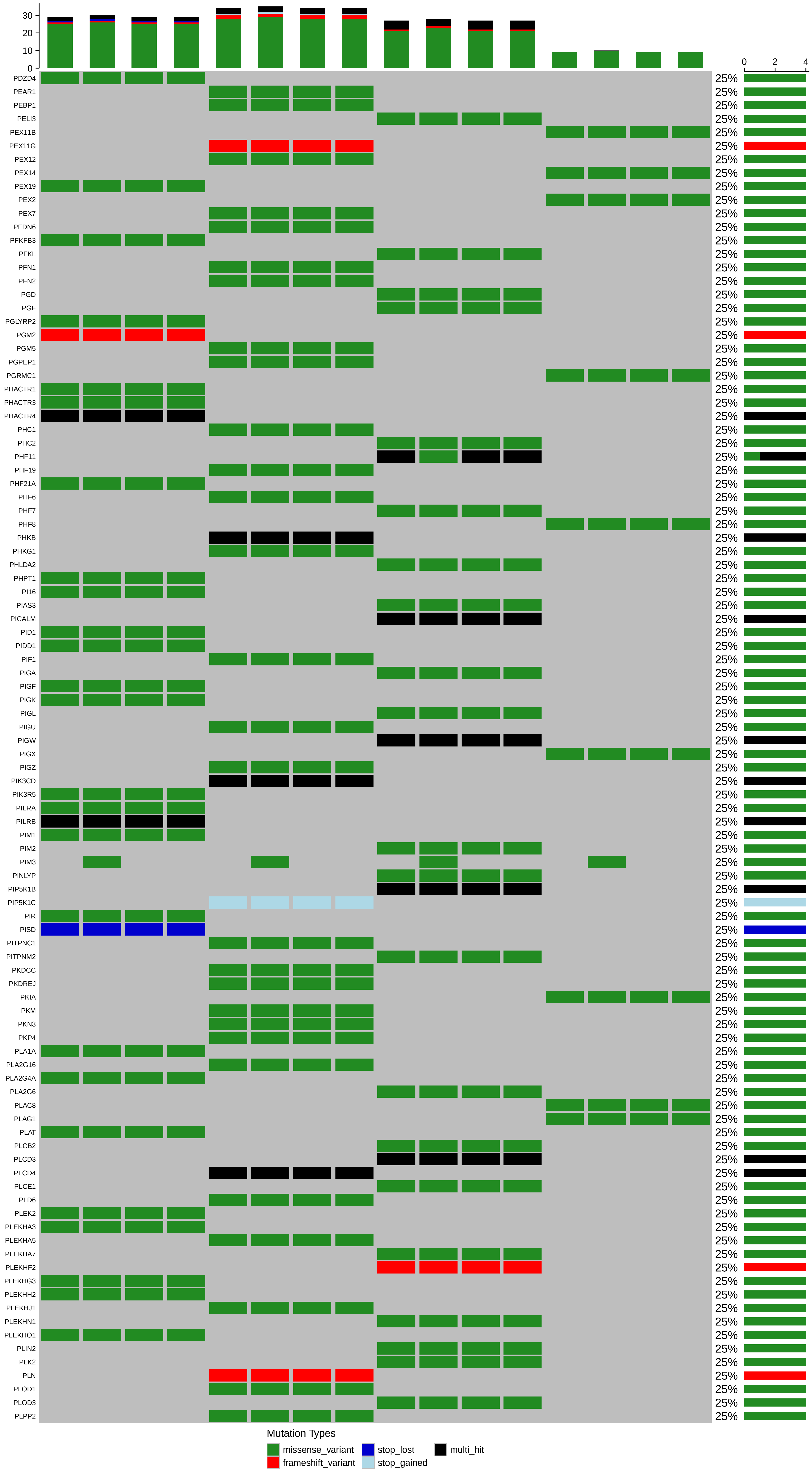

30
20
10
0
0 2 4
PDZD4
PEAR1
PEBP1
PELI3
PEX11B
PEX11G
PEX12
PEX14
PEX19
PEX2
PEX7
PFDN6
PFKFB3
PFKL
PFN1
PFN2
PGD
PGF
PGLYRP2
PGM2
PGM5
PGPEP1
PGRMC1
PHACTR1
PHACTR3
PHACTR4
PHC1
PHC2
PHF11
PHF19
PHF21A
PHF6
PHF7
PHF8
PHKB
PHKG1
PHLDA2
PHPT1
PI16
PIAS3
PICALM
PID1
PIDD1
PIF1
PIGA
PIGF
PIGK
PIGL
PIGU
PIGW
PIGX
PIGZ
PIK3CD
PIK3R5
PILRA
PILRB
PIM1
PIM2
PIM3
PINLYP
PIP5K1B
PIP5K1C
PIR
PISD
PITPNC1
PITPNM2
PKDCC
PKDREJ
PKIA
PKM
PKN3
PKP4
PLA1A
PLA2G16
PLA2G4A
PLA2G6
PLAC8
PLAG1
PLAT
PLCB2
PLCD3
PLCD4
PLCE1
PLD6
PLEK2
PLEKHA3
PLEKHA5
PLEKHA7
PLEKHF2
PLEKHG3
PLEKHH2
PLEKHJ1
PLEKHN1
PLEKHO1
PLIN2
PLK2
PLN
PLOD1
PLOD3
PLPP2
25% (each row)
Mutation Types
missense_variant
frameshift_variant
stop_lost
stop_gained
multi_hit

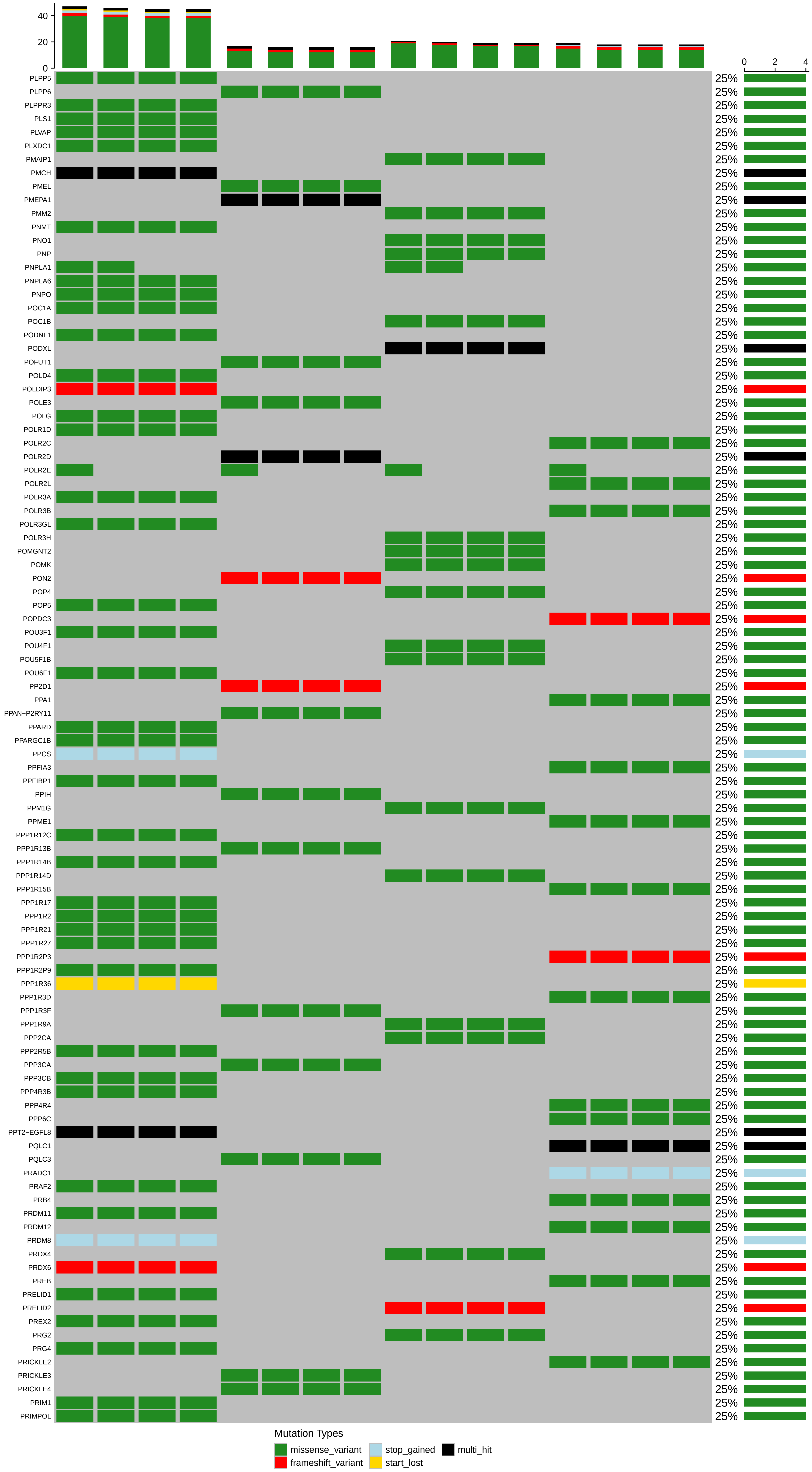

0
20
40
0
2
4
PLPP5
PLPP6
PLPPR3
PLS1
PLVAP
PLXDC1
PMAIP1
PMCH
PMEL
PMEPA1
PMM2
PNMT
PNO1
PNP
PNPLA1
PNPLA6
PNPO
POC1A
POC1B
PODNL1
PODXL
POFUT1
POLD4
POLDIP3
POLE3
POLG
POLR1D
POLR2C
POLR2D
POLR2E
POLR2L
POLR3A
POLR3B
POLR3GL
POLR3H
POMGNT2
POMK
PON2
POP4
POP5
POPDC3
POU3F1
POU4F1
POU5F1B
POU6F1
PP2D1
PPA1
PPAN−P2RY11
PPARD
PPARGC1B
PPCS
PPFIA3
PPFIBP1
PPIH
PPM1G
PPME1
PPP1R12C
PPP1R13B
PPP1R14B
PPP1R14D
PPP1R15B
PPP1R17
PPP1R2
PPP1R21
PPP1R27
PPP1R2P3
PPP1R2P9
PPP1R36
PPP1R3D
PPP1R3F
PPP1R9A
PPP2CA
PPP2R5B
PPP3CA
PPP3CB
PPP4R3B
PPP4R4
PPP6C
PPT2−EGFL8
PQLC1
PQLC3
PRADC1
PRAF2
PRB4
PRDM11
PRDM12
PRDM8
PRDX4
PRDX6
PREB
PRELID1
PRELID2
PREX2
PRG2
PRG4
PRICKLE2
PRICKLE3
PRICKLE4
PRIM1
PRIMPOL
25%
Mutation Types
missense_variant
frameshift_variant
stop_gained
start_lost
multi_hit

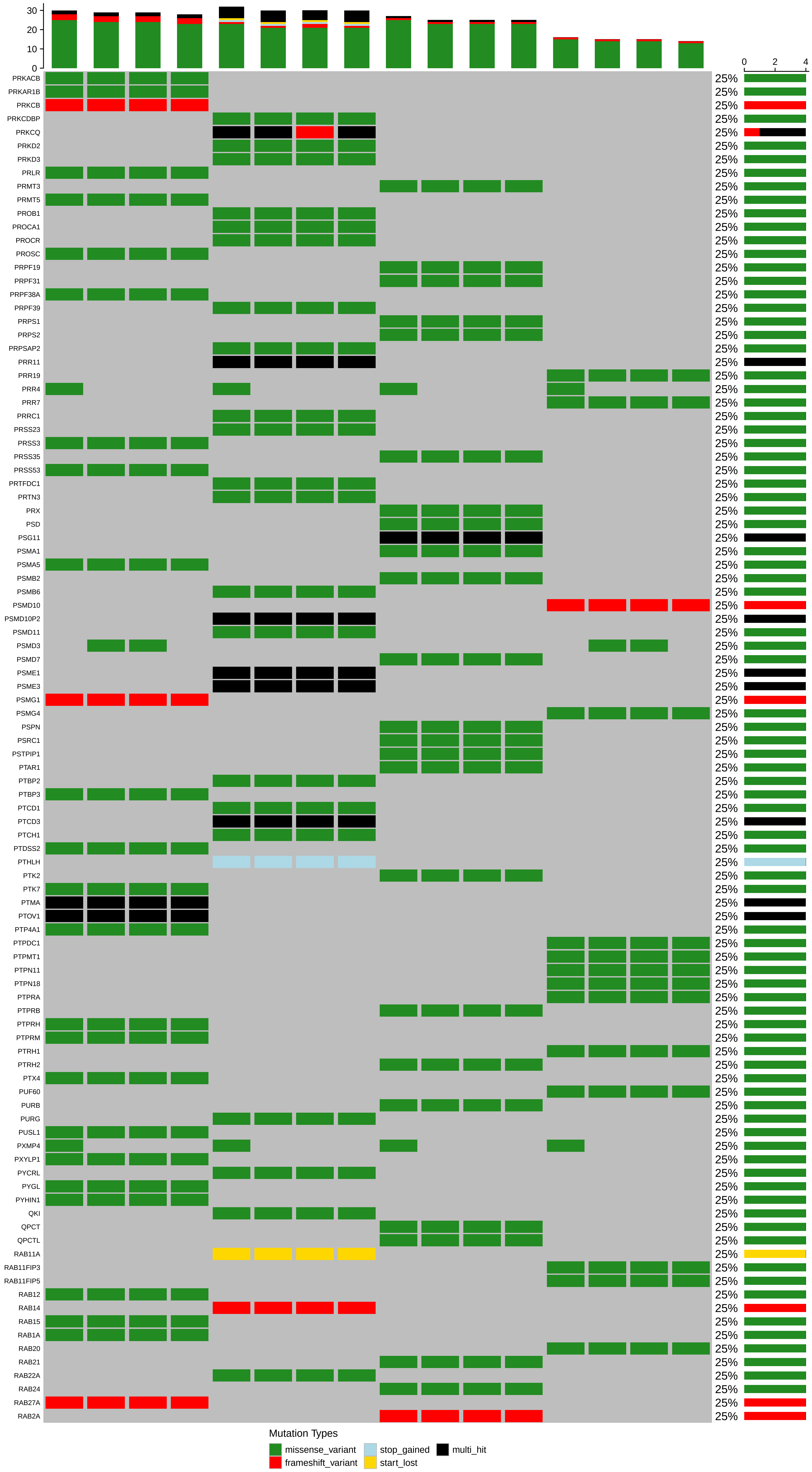

30
20
10
0
0
2
4
PRKACB
PRKAR1B
PRKCB
PRKCDBP
PRKCQ
PRKD2
PRKD3
PRLR
PRMT3
PRMT5
PROB1
PROCA1
PROCR
PROSC
PRPF19
PRPF31
PRPF38A
PRPF39
PRPS1
PRPS2
PRPSAP2
PRR11
PRR19
PRR4
PRR7
PRRC1
PRSS23
PRSS3
PRSS35
PRSS53
PRTFDC1
PRTN3
PRX
PSD
PSG11
PSMA1
PSMA5
PSMB2
PSMB6
PSMD10
PSMD10P2
PSMD11
PSMD3
PSMD7
PSME1
PSME3
PSMG1
PSMG4
PSPN
PSRC1
PSTPIP1
PTAR1
PTBP2
PTBP3
PTCD1
PTCD3
PTCH1
PTDSS2
PTHLH
PTK2
PTK7
PTMA
PTOV1
PTP4A1
PTPDC1
PTPMT1
PTPN11
PTPN18
PTPRA
PTPRB
PTPRH
PTPRM
PTRH1
PTRH2
PTX4
PUF60
PURB
PURG
PUSL1
PXMP4
PXYLP1
PYCRL
PYGL
PYHIN1
QKI
QPCT
QPCTL
RAB11A
RAB11FIP3
RAB11FIP5
RAB12
RAB14
RAB15
RAB1A
RAB20
RAB21
RAB22A
RAB24
RAB27A
RAB2A
25% (printed once beside each of the 100 gene rows)
Mutation Types
missense_variant
frameshift_variant
stop_gained
start_lost
multi_hit

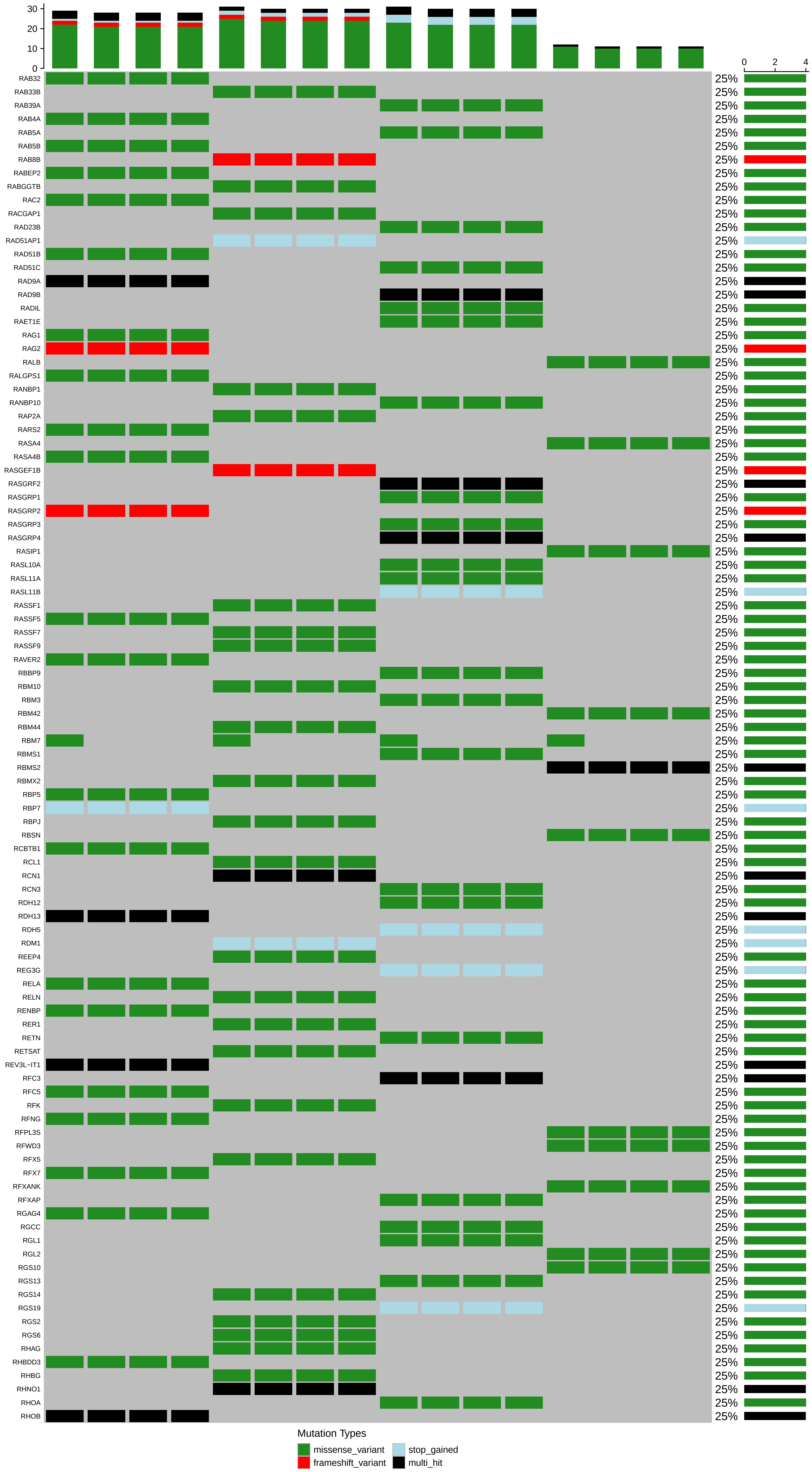

30
20
10
0
0
2
4
RAB32
RAB33B
RAB39A
RAB4A
RAB5A
RAB5B
RAB8B
RABEP2
RABGGTB
RAC2
RACGAP1
RAD23B
RAD51AP1
RAD51B
RAD51C
RAD9A
RAD9B
RADIL
RAET1E
RAG1
RAG2
RALB
RALGPS1
RANBP1
RANBP10
RAP2A
RARS2
RASA4
RASA4B
RASGEF1B
RASGRF2
RASGRP1
RASGRP2
RASGRP3
RASGRP4
RASIP1
RASL10A
RASL11A
RASL11B
RASSF1
RASSF5
RASSF7
RASSF9
RAVER2
RBBP9
RBM10
RBM3
RBM42
RBM44
RBM7
RBMS1
RBMS2
RBMX2
RBP5
RBP7
RBPJ
RBSN
RCBTB1
RCL1
RCN1
RCN3
RDH12
RDH13
RDH5
RDM1
REEP4
REG3G
RELA
RELN
RENBP
RER1
RETN
RETSAT
REV3L−IT1
RFC3
RFC5
RFK
RFNG
RFPL3S
RFWD3
RFX5
RFX7
RFXANK
RFXAP
RGAG4
RGCC
RGL1
RGL2
RGS10
RGS13
RGS14
RGS19
RGS2
RGS6
RHAG
RHBDD3
RHBG
RHNO1
RHOA
RHOB
25%
Mutation Types
missense_variant
frameshift_variant
stop_gained
multi_hit

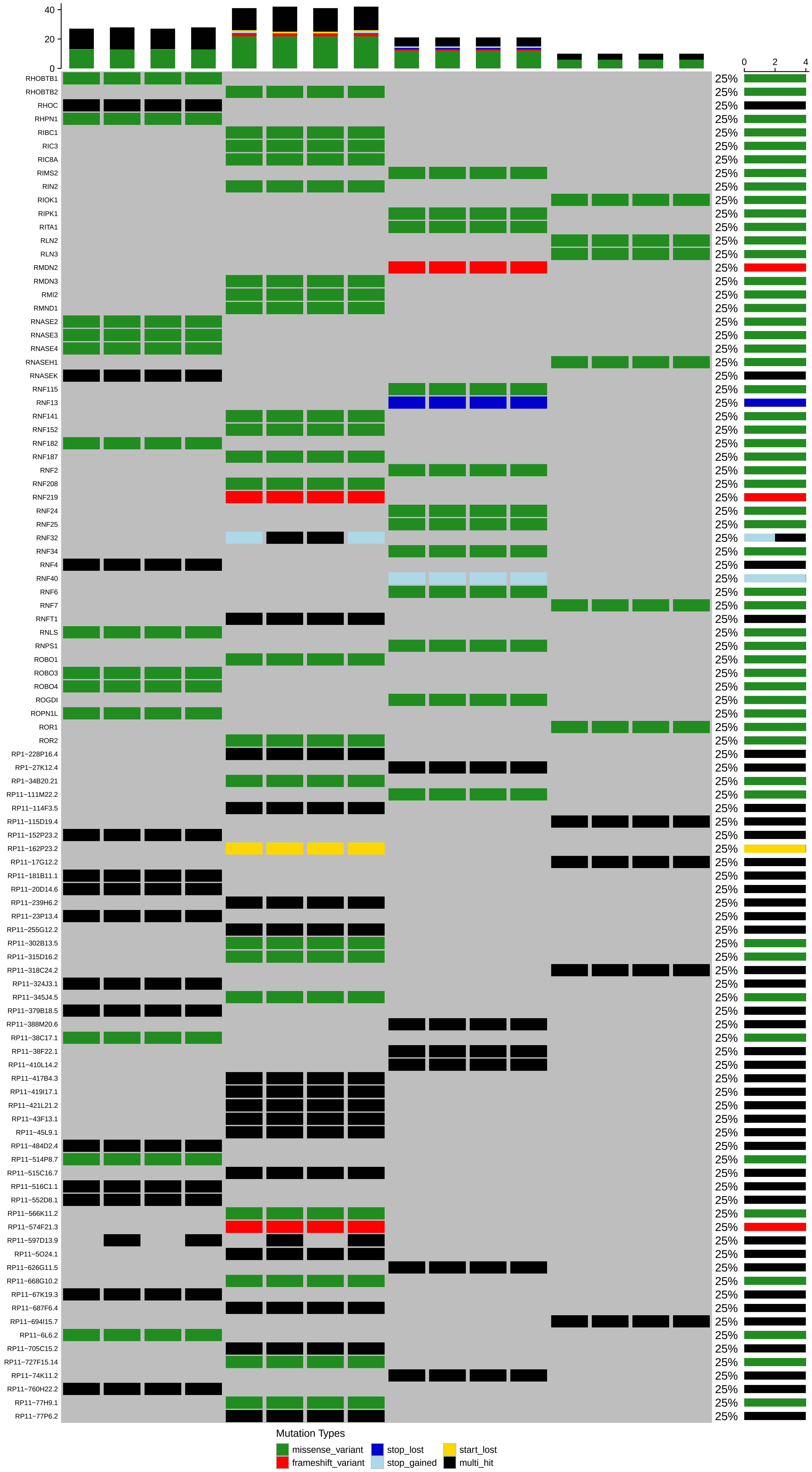
40
20
0
0
2
4
RHOBTB1
RHOBTB2
RHOC
RHPN1
RIBC1
RIC3
RIC8A
RIMS2
RIN2
RIOK1
RIPK1
RITA1
RLN2
RLN3
RMDN2
RMDN3
RMI2
RMND1
RNASE2
RNASE3
RNASE4
RNASEH1
RNASEK
RNF115
RNF13
RNF141
RNF152
RNF182
RNF187
RNF2
RNF208
RNF219
RNF24
RNF25
RNF32
RNF34
RNF4
RNF40
RNF6
RNF7
RNFT1
RNLS
RNPS1
ROBO1
ROBO3
ROBO4
ROGDI
ROPN1L
ROR1
ROR2
RP1−228P16.4
RP1−27K12.4
RP1−34B20.21
RP11−111M22.2
RP11−114F3.5
RP11−115D19.4
RP11−152P23.2
RP11−162P23.2
RP11−17G12.2
RP11−181B11.1
RP11−20D14.6
RP11−239H6.2
RP11−23P13.4
RP11−255G12.2
RP11−302B13.5
RP11−315D16.2
RP11−318C24.2
RP11−324J3.1
RP11−345J4.5
RP11−379B18.5
RP11−388M20.6
RP11−38C17.1
RP11−38F22.1
RP11−410L14.2
RP11−417B4.3
RP11−419I17.1
RP11−421L21.2
RP11−43F13.1
RP11−45L9.1
RP11−484D2.4
RP11−514P8.7
RP11−515C16.7
RP11−516C1.1
RP11−552D8.1
RP11−566K11.2
RP11−574F21.3
RP11−597D13.9
RP11−5O24.1
RP11−626G11.5
RP11−668G10.2
RP11−67K19.3
RP11−687F6.4
RP11−694I15.7
RP11−6L6.2
RP11−705C15.2
RP11−727F15.14
RP11−74K11.2
RP11−760H22.2
RP11−77H9.1
RP11−77P6.2
25% (each row)
Mutation Types
missense_variant
frameshift_variant
stop_lost
stop_gained
start_lost
multi_hit

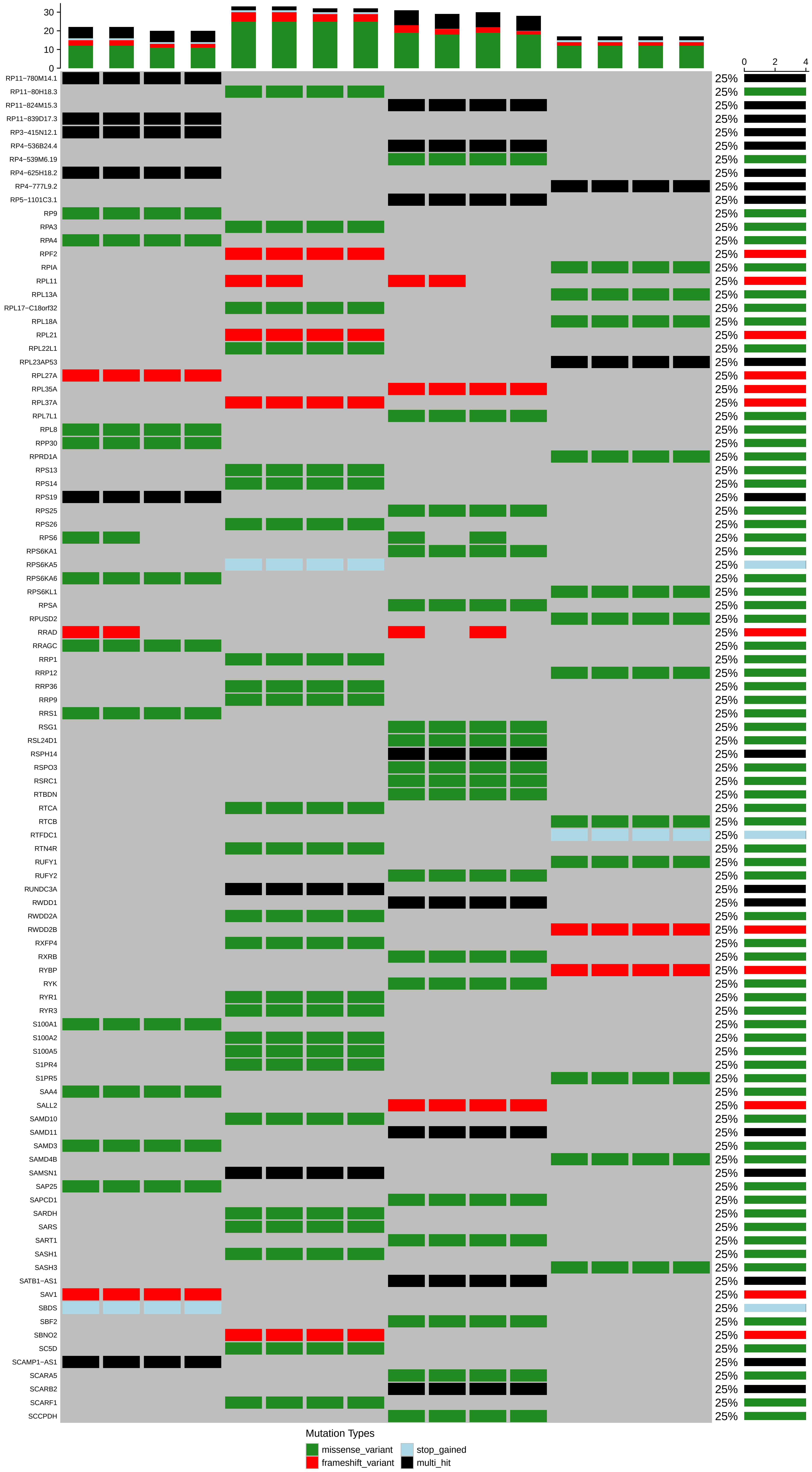

30
20
10
0
0
2
4
RP11−780M14.1
RP11−80H18.3
RP11−824M15.3
RP11−839D17.3
RP3−415N12.1
RP4−536B24.4
RP4−539M6.19
RP4−625H18.2
RP4−777L9.2
RP5−1101C3.1
RP9
RPA3
RPA4
RPF2
RPIA
RPL11
RPL13A
RPL17−C18orf32
RPL18A
RPL21
RPL22L1
RPL23AP53
RPL27A
RPL35A
RPL37A
RPL7L1
RPL8
RPP30
RPRD1A
RPS13
RPS14
RPS19
RPS25
RPS26
RPS6
RPS6KA1
RPS6KA5
RPS6KA6
RPS6KL1
RPSA
RPUSD2
RRAD
RRAGC
RRP1
RRP12
RRP36
RRP9
RRS1
RSG1
RSL24D1
RSPH14
RSPO3
RSRC1
RTBDN
RTCA
RTCB
RTFDC1
RTN4R
RUFY1
RUFY2
RUNDC3A
RWDD1
RWDD2A
RWDD2B
RXFP4
RXRB
RYBP
RYK
RYR1
RYR3
S100A1
S100A2
S100A5
S1PR4
S1PR5
SAA4
SALL2
SAMD10
SAMD11
SAMD3
SAMD4B
SAMSN1
SAP25
SAPCD1
SARDH
SARS
SART1
SASH1
SASH3
SATB1−AS1
SAV1
SBDS
SBF2
SBNO2
SC5D
SCAMP1−AS1
SCARA5
SCARB2
SCARF1
SCCPDH
25%
Mutation Types
missense_variant
frameshift_variant
stop_gained
multi_hit

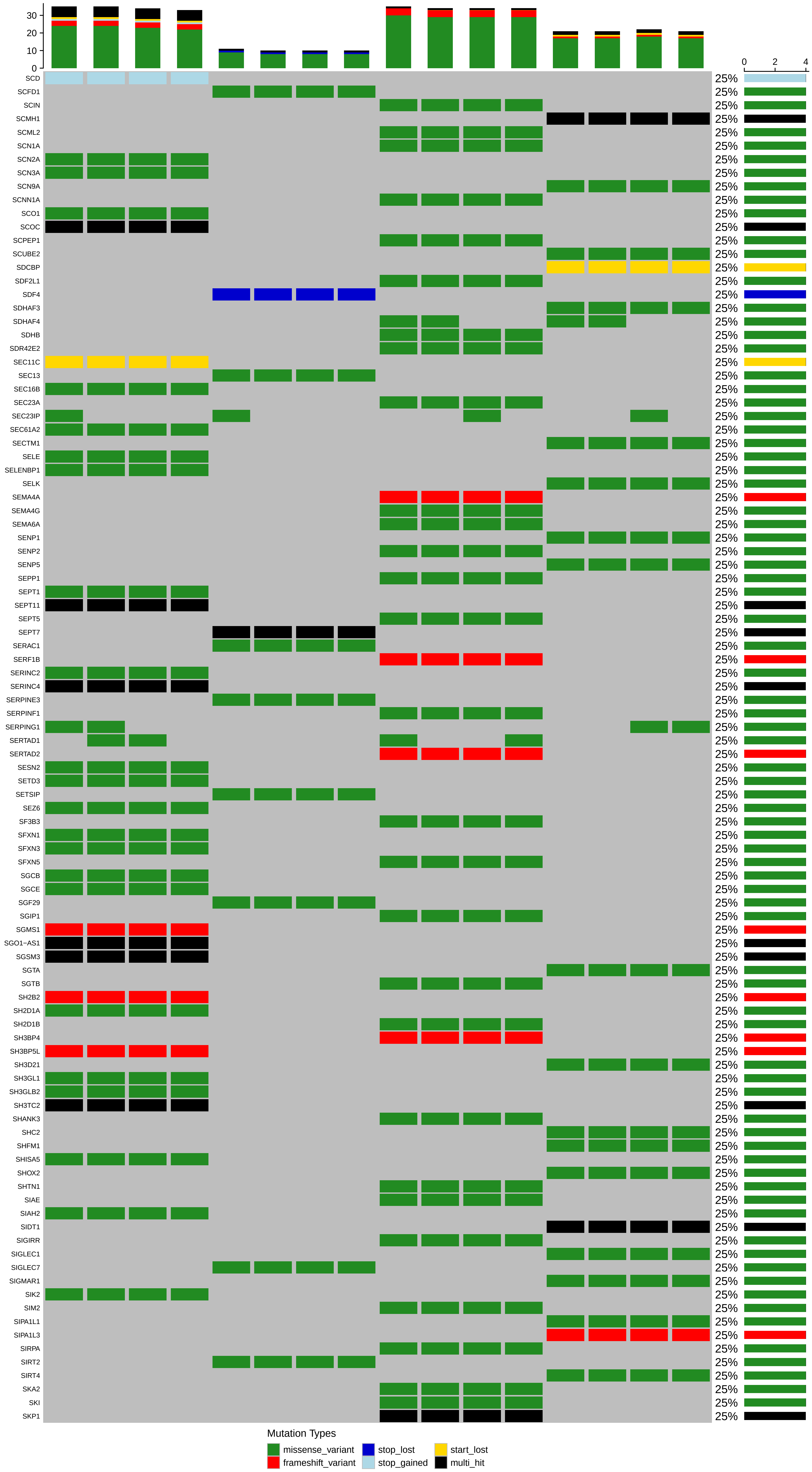

SCD
SCFD1
SCIN
SCMH1
SCML2
SCN1A
SCN2A
SCN3A
SCN9A
SCNN1A
SCO1
SCOC
SCPEP1
SCUBE2
SDCBP
SDF2L1
SDF4
SDHAF3
SDHAF4
SDHB
SDR42E2
SEC11C
SEC13
SEC16B
SEC23A
SEC23IP
SEC61A2
SECTM1
SELE
SELENBP1
SELK
SEMA4A
SEMA4G
SEMA6A
SENP1
SENP2
SENP5
SEPP1
SEPT1
SEPT11
SEPT5
SEPT7
SERAC1
SERF1B
SERINC2
SERINC4
SERPINE3
SERPINF1
SERPING1
SERTAD1
SERTAD2
SESN2
SETD3
SETSIP
SEZ6
SF3B3
SFXN1
SFXN3
SFXN5
SGCB
SGCE
SGF29
SGIP1
SGMS1
SGO1−AS1
SGSM3
SGTA
SGTB
SH2B2
SH2D1A
SH2D1B
SH3BP4
SH3BP5L
SH3D21
SH3GL1
SH3GLB2
SH3TC2
SHANK3
SHC2
SHFM1
SHISA5
SHOX2
SHTN1
SIAE
SIAH2
SIDT1
SIGIRR
SIGLEC1
SIGLEC7
SIGMAR1
SIK2
SIM2
SIPA1L1
SIPA1L3
SIRPA
SIRT2
SIRT4
SKA2
SKI
SKP1
Mutation Types
missense_variant
frameshift_variant
stop_lost
stop_gained
start_lost
multi_hit

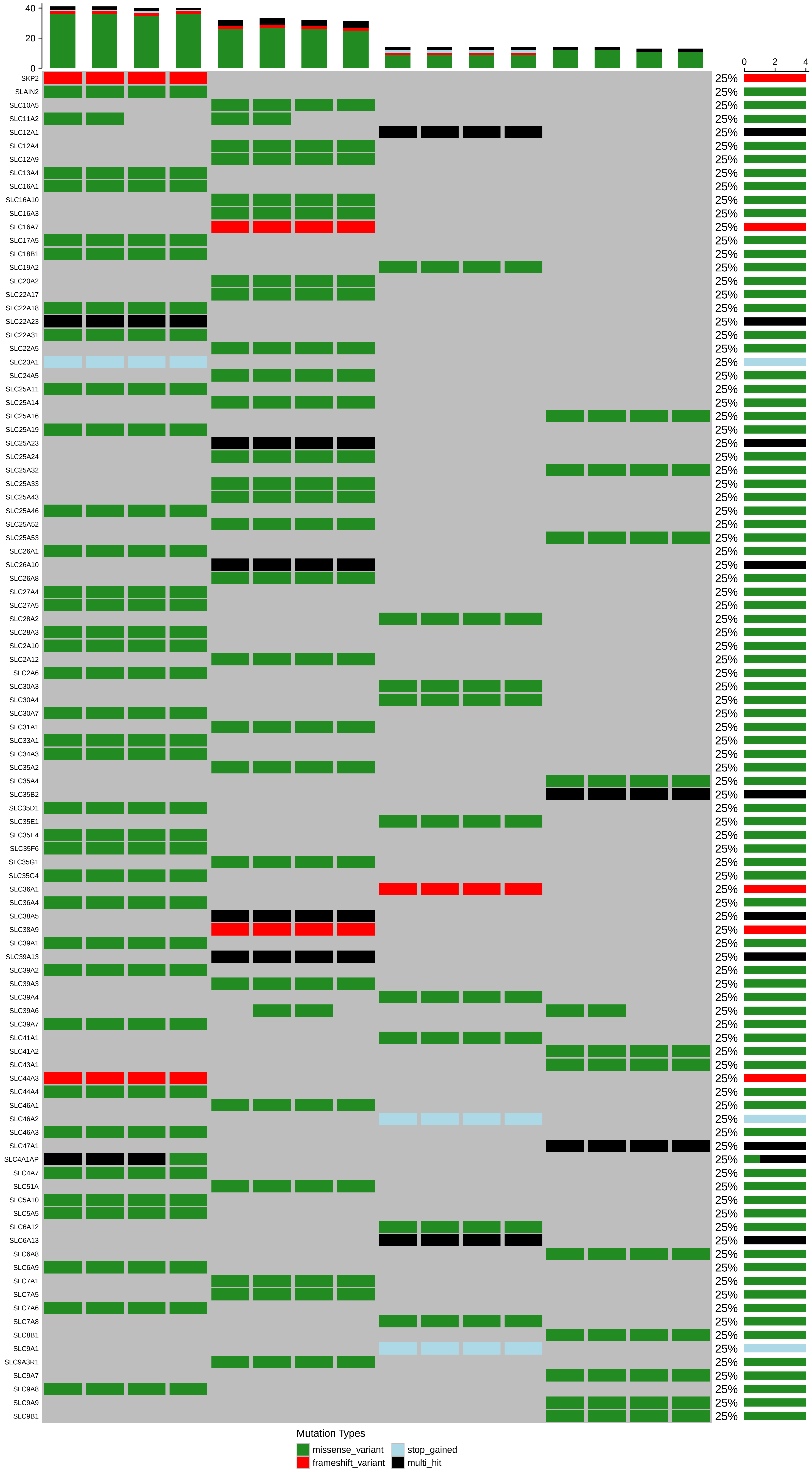

40
20
0
0
2
4
SKP2
SLAIN2
SLC10A5
SLC11A2
SLC12A1
SLC12A4
SLC12A9
SLC13A4
SLC16A1
SLC16A10
SLC16A3
SLC16A7
SLC17A5
SLC18B1
SLC19A2
SLC20A2
SLC22A17
SLC22A18
SLC22A23
SLC22A31
SLC22A5
SLC23A1
SLC24A5
SLC25A11
SLC25A14
SLC25A16
SLC25A19
SLC25A23
SLC25A24
SLC25A32
SLC25A33
SLC25A43
SLC25A46
SLC25A52
SLC25A53
SLC26A1
SLC26A10
SLC26A8
SLC27A4
SLC27A5
SLC28A2
SLC28A3
SLC2A10
SLC2A12
SLC2A6
SLC30A3
SLC30A4
SLC30A7
SLC31A1
SLC33A1
SLC34A3
SLC35A2
SLC35A4
SLC35B2
SLC35D1
SLC35E1
SLC35E4
SLC35F6
SLC35G1
SLC35G4
SLC36A1
SLC36A4
SLC38A5
SLC38A9
SLC39A1
SLC39A13
SLC39A2
SLC39A3
SLC39A4
SLC39A6
SLC39A7
SLC41A1
SLC41A2
SLC43A1
SLC44A3
SLC44A4
SLC46A1
SLC46A2
SLC46A3
SLC47A1
SLC4A1AP
SLC4A7
SLC51A
SLC5A10
SLC5A5
SLC6A12
SLC6A13
SLC6A8
SLC6A9
SLC7A1
SLC7A5
SLC7A6
SLC7A8
SLC8B1
SLC9A1
SLC9A3R1
SLC9A7
SLC9A8
SLC9A9
SLC9B1
25%
Mutation Types
missense_variant
frameshift_variant
stop_gained
multi_hit

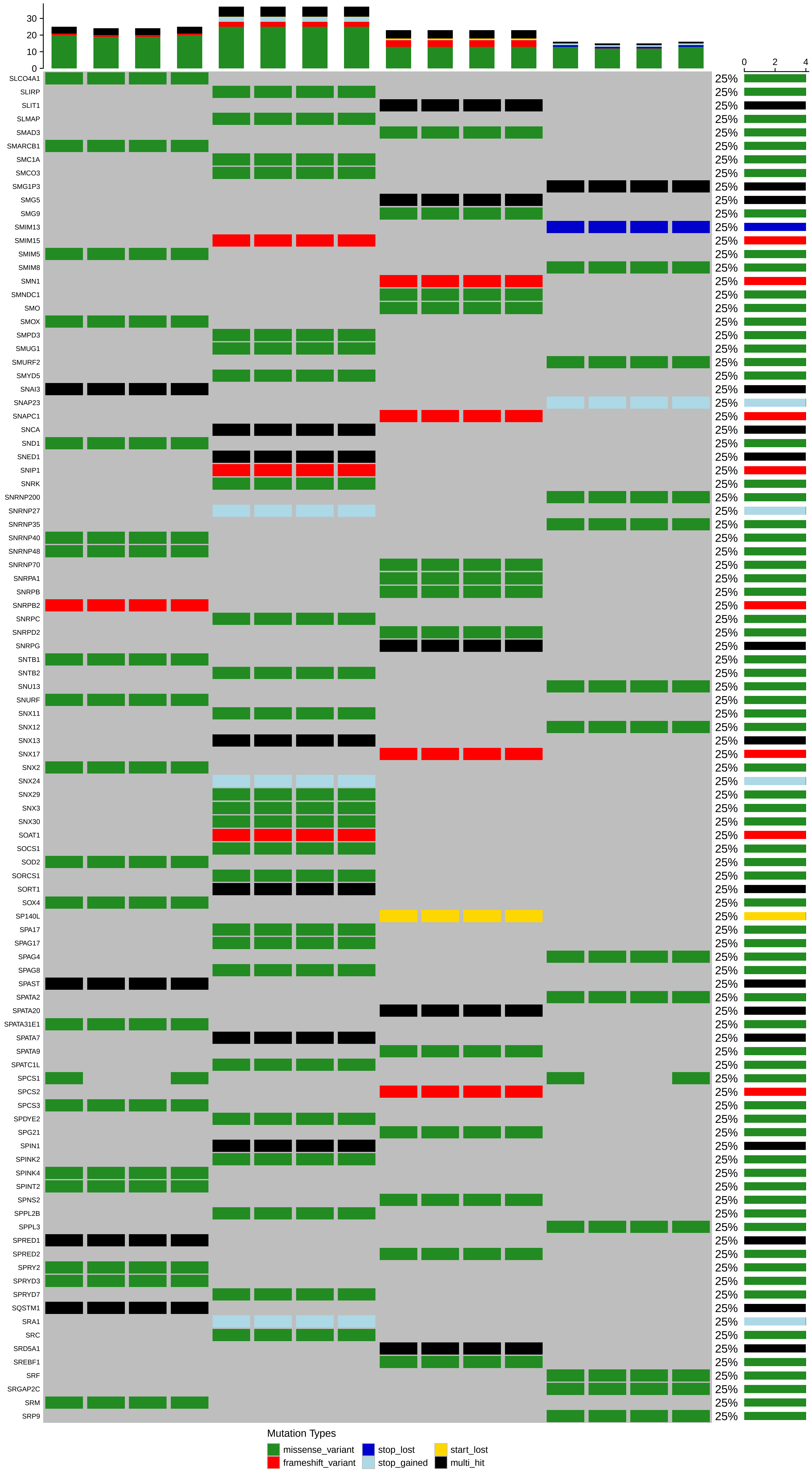
30
20
10
0
0
2
4
SLCO4A1
SLIRP
SLIT1
SLMAP
SMAD3
SMARCB1
SMC1A
SMCO3
SMG1P3
SMG5
SMG9
SMIM13
SMIM15
SMIM5
SMIM8
SMN1
SMNDC1
SMO
SMOX
SMPD3
SMUG1
SMURF2
SMYD5
SNAI3
SNAP23
SNAPC1
SNCA
SND1
SNED1
SNIP1
SNRK
SNRNP200
SNRNP27
SNRNP35
SNRNP40
SNRNP48
SNRNP70
SNRPA1
SNRPB
SNRPB2
SNRPC
SNRPD2
SNRPG
SNTB1
SNTB2
SNU13
SNURF
SNX11
SNX12
SNX13
SNX17
SNX2
SNX24
SNX29
SNX3
SNX30
SOAT1
SOCS1
SOD2
SORCS1
SORT1
SOX4
SP140L
SPA17
SPAG17
SPAG4
SPAG8
SPAST
SPATA2
SPATA20
SPATA31E1
SPATA7
SPATA9
SPATC1L
SPCS1
SPCS2
SPCS3
SPDYE2
SPG21
SPIN1
SPINK2
SPINK4
SPINT2
SPNS2
SPPL2B
SPPL3
SPRED1
SPRED2
SPRY2
SPRYD3
SPRYD7
SQSTM1
SRA1
SRC
SRD5A1
SREBF1
SRF
SRGAP2C
SRM
SRP9
25% (printed beside each of the 101 gene rows)
Mutation Types
missense_variant
frameshift_variant
stop_lost
stop_gained
start_lost
multi_hit

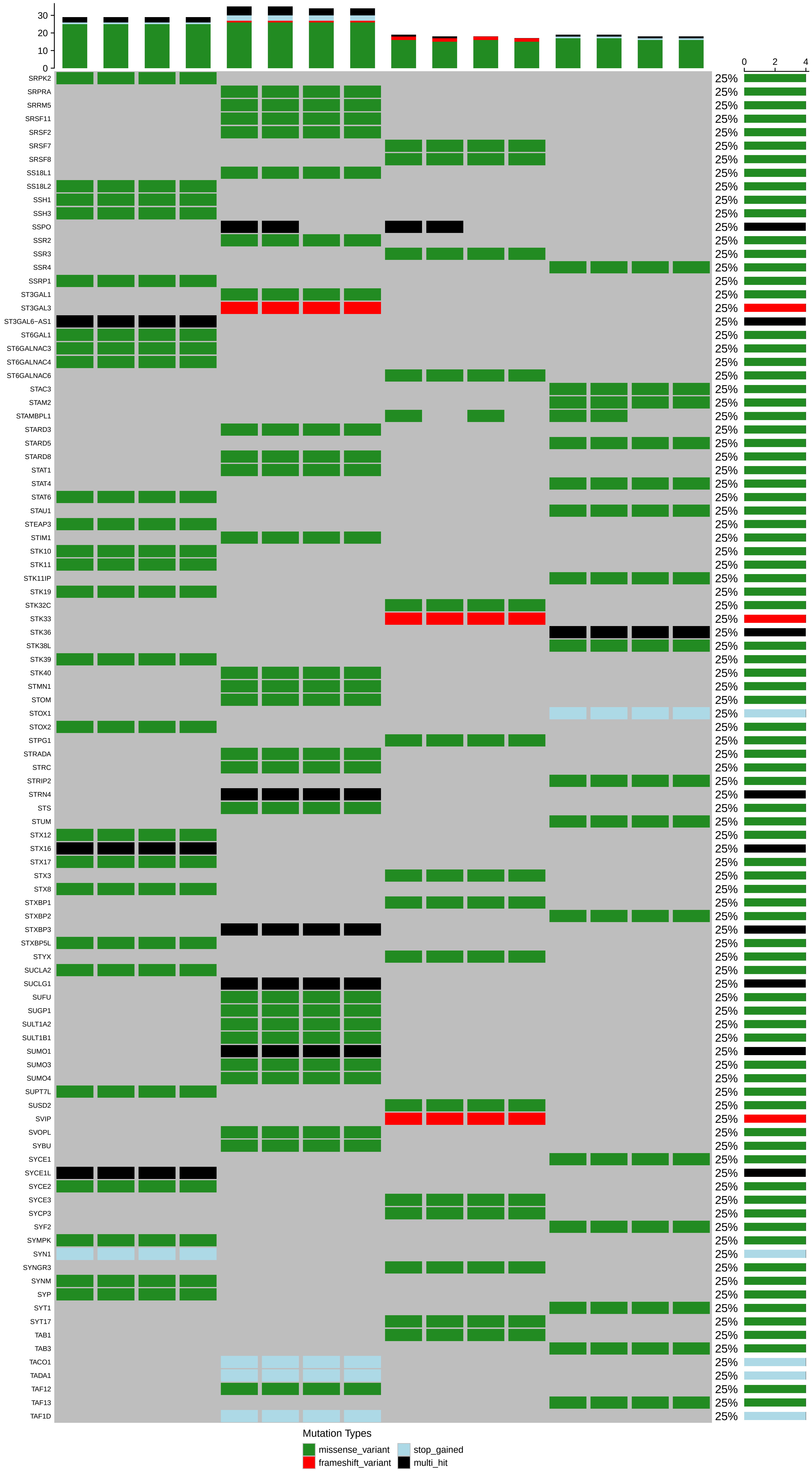
30
20
10
0
0
2
4
SRPK2
SRPRA
SRRM5
SRSF11
SRSF2
SRSF7
SRSF8
SS18L1
SS18L2
SSH1
SSH3
SSPO
SSR2
SSR3
SSR4
SSRP1
ST3GAL1
ST3GAL3
ST3GAL6−AS1
ST6GAL1
ST6GALNAC3
ST6GALNAC4
ST6GALNAC6
STAC3
STAM2
STAMBPL1
STARD3
STARD5
STARD8
STAT1
STAT4
STAT6
STAU1
STEAP3
STIM1
STK10
STK11
STK11IP
STK19
STK32C
STK33
STK36
STK38L
STK39
STK40
STMN1
STOM
STOX1
STOX2
STPG1
STRADA
STRC
STRIP2
STRN4
STS
STUM
STX12
STX16
STX17
STX3
STX8
STXBP1
STXBP2
STXBP3
STXBP5L
STYX
SUCLA2
SUCLG1
SUFU
SUGP1
SULT1A2
SULT1B1
SUMO1
SUMO3
SUMO4
SUPT7L
SUSD2
SVIP
SVOPL
SYBU
SYCE1
SYCE1L
SYCE2
SYCE3
SYCP3
SYF2
SYMPK
SYN1
SYNGR3
SYNM
SYP
SYT1
SYT17
TAB1
TAB3
TACO1
TADA1
TAF12
TAF13
TAF1D
25% (printed beside each of the 101 gene rows)
Mutation Types
missense_variant
frameshift_variant
stop_gained
multi_hit

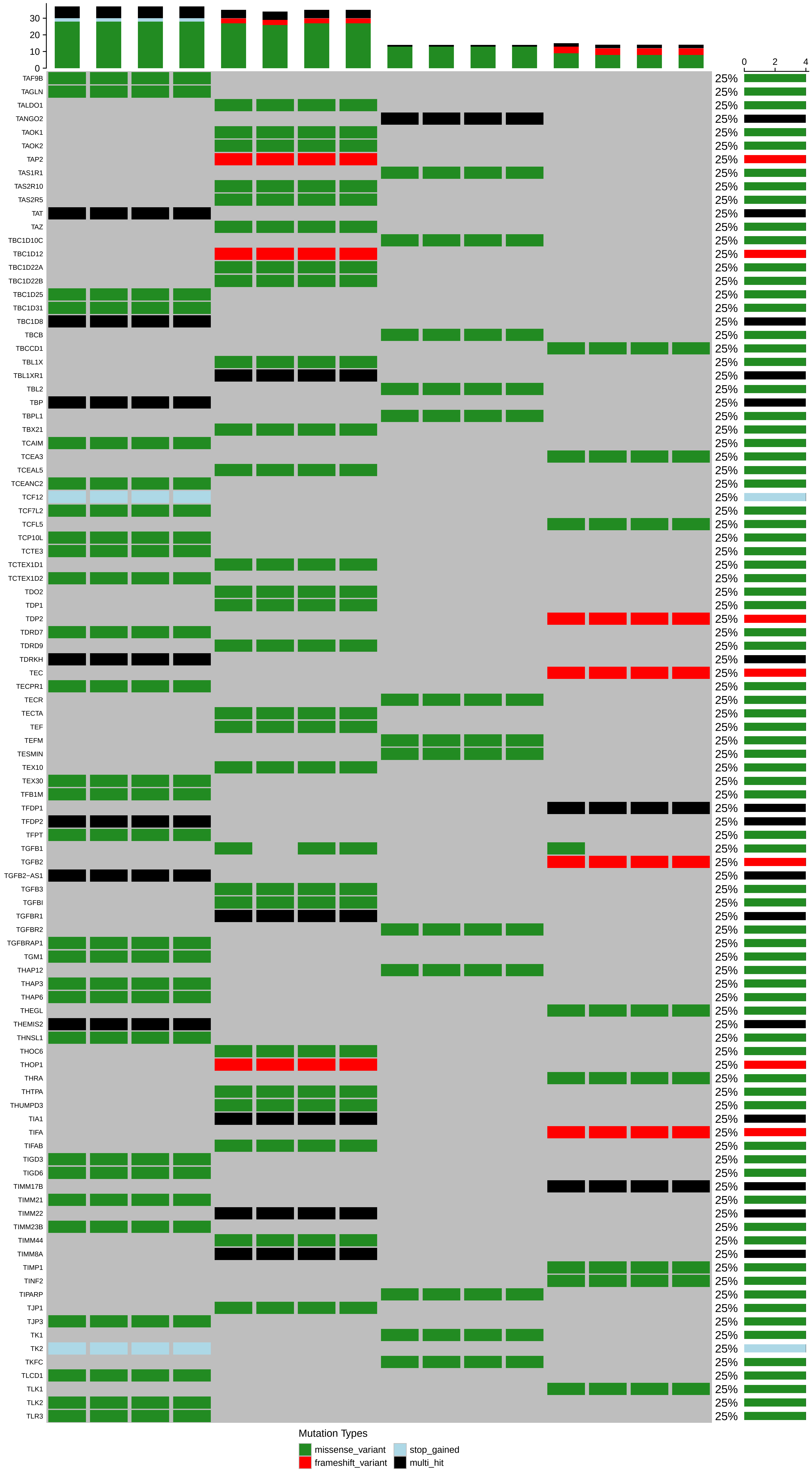

TAF9B
TAGLN
TALDO1
TANGO2
TAOK1
TAOK2
TAP2
TAS1R1
TAS2R10
TAS2R5
TAT
TAZ
TBC1D10C
TBC1D12
TBC1D22A
TBC1D22B
TBC1D25
TBC1D31
TBC1D8
TBCB
TBCCD1
TBL1X
TBL1XR1
TBL2
TBP
TBPL1
TBX21
TCAIM
TCEA3
TCEAL5
TCEANC2
TCF12
TCF7L2
TCFL5
TCP10L
TCTE3
TCTEX1D1
TCTEX1D2
TDO2
TDP1
TDP2
TDRD7
TDRD9
TDRKH
TEC
TECPR1
TECR
TECTA
TEF
TEFM
TESMIN
TEX10
TEX30
TFB1M
TFDP1
TFDP2
TFPT
TGFB1
TGFB2
TGFB2−AS1
TGFB3
TGFBI
TGFBR1
TGFBR2
TGFBRAP1
TGM1
THAP12
THAP3
THAP6
THEGL
THEMIS2
THNSL1
THOC6
THOP1
THRA
THTPA
THUMPD3
TIA1
TIFA
TIFAB
TIGD3
TIGD6
TIMM17B
TIMM21
TIMM22
TIMM23B
TIMM44
TIMM8A
TIMP1
TINF2
TIPARP
TJP1
TJP3
TK1
TK2
TKFC
TLCD1
TLK1
TLK2
TLR3
25%
0
10
20
30
0
2
4
Mutation Types
missense_variant
frameshift_variant
stop_gained
multi_hit

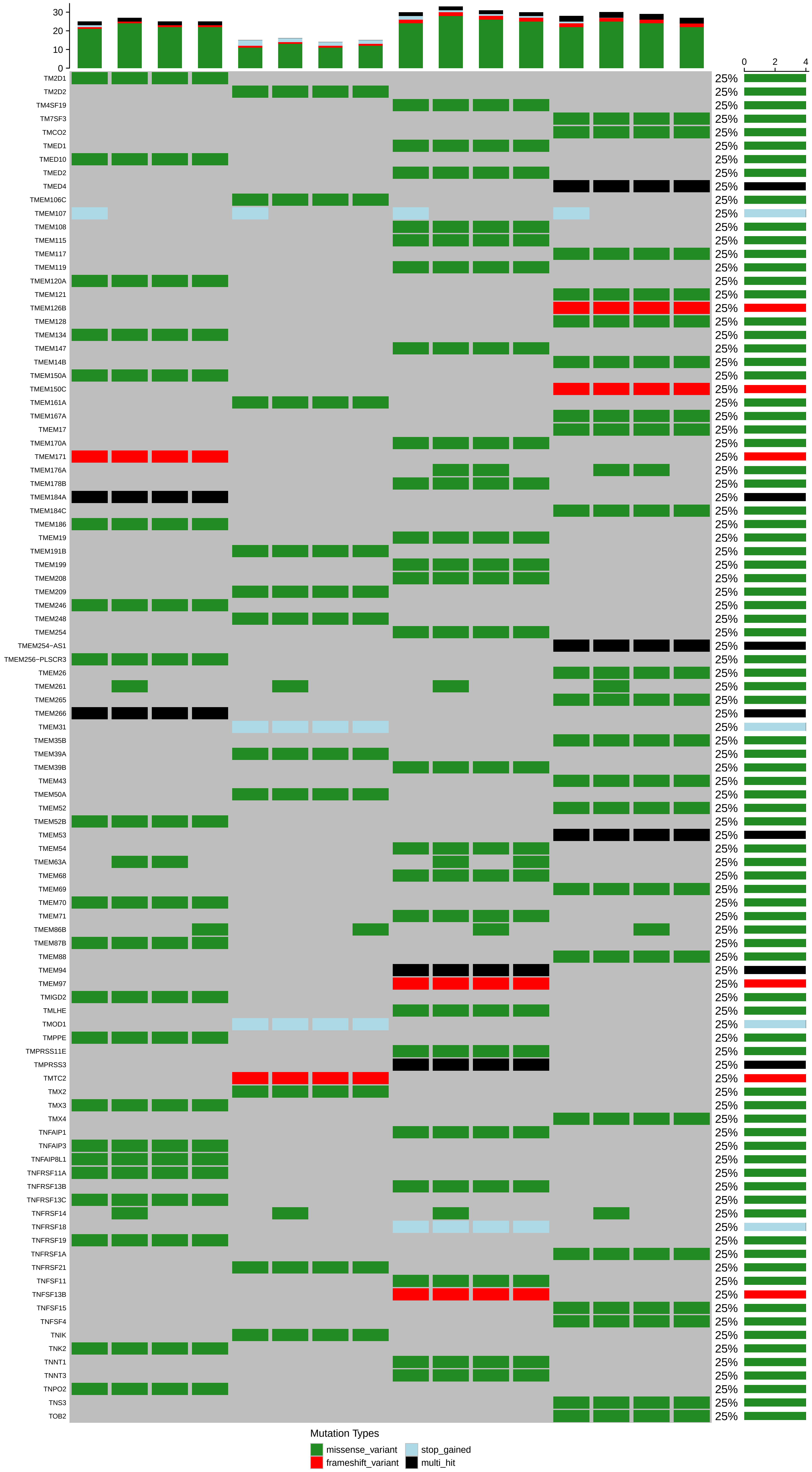

TM2D1
TM2D2
TM4SF19
TM7SF3
TMCO2
TMED1
TMED10
TMED2
TMED4
TMEM106C
TMEM107
TMEM108
TMEM115
TMEM117
TMEM119
TMEM120A
TMEM121
TMEM126B
TMEM128
TMEM134
TMEM147
TMEM14B
TMEM150A
TMEM150C
TMEM161A
TMEM167A
TMEM17
TMEM170A
TMEM171
TMEM176A
TMEM178B
TMEM184A
TMEM184C
TMEM186
TMEM19
TMEM191B
TMEM199
TMEM208
TMEM209
TMEM246
TMEM248
TMEM254
TMEM254−AS1
TMEM256−PLSCR3
TMEM26
TMEM261
TMEM265
TMEM266
TMEM31
TMEM35B
TMEM39A
TMEM39B
TMEM43
TMEM50A
TMEM52
TMEM52B
TMEM53
TMEM54
TMEM63A
TMEM68
TMEM69
TMEM70
TMEM71
TMEM86B
TMEM87B
TMEM88
TMEM94
TMEM97
TMIGD2
TMLHE
TMOD1
TMPPE
TMPRSS11E
TMPRSS3
TMTC2
TMX2
TMX3
TMX4
TNFAIP1
TNFAIP3
TNFAIP8L1
TNFRSF11A
TNFRSF13B
TNFRSF13C
TNFRSF14
TNFRSF18
TNFRSF19
TNFRSF1A
TNFRSF21
TNFSF11
TNFSF13B
TNFSF15
TNFSF4
TNIK
TNK2
TNNT1
TNNT3
TNPO2
TNS3
TOB2
25%
Mutation Types
missense_variant
frameshift_variant
stop_gained
multi_hit

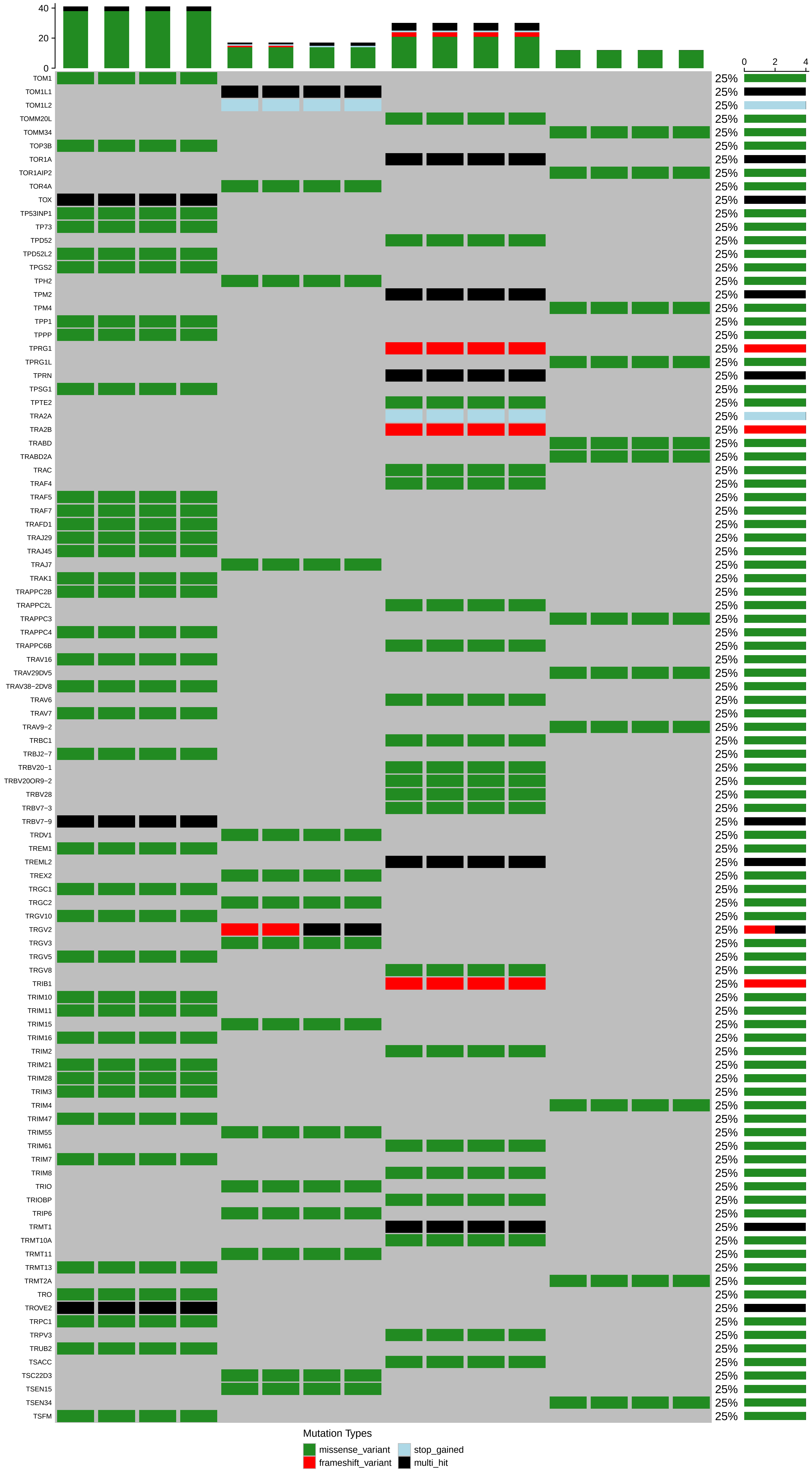

40
20
0
0
2
4
TOM1
TOM1L1
TOM1L2
TOMM20L
TOMM34
TOP3B
TOR1A
TOR1AIP2
TOR4A
TOX
TP53INP1
TP73
TPD52
TPD52L2
TPGS2
TPH2
TPM2
TPM4
TPP1
TPPP
TPRG1
TPRG1L
TPRN
TPSG1
TPTE2
TRA2A
TRA2B
TRABD
TRABD2A
TRAC
TRAF4
TRAF5
TRAF7
TRAFD1
TRAJ29
TRAJ45
TRAJ7
TRAK1
TRAPPC2B
TRAPPC2L
TRAPPC3
TRAPPC4
TRAPPC6B
TRAV16
TRAV29DV5
TRAV38−2DV8
TRAV6
TRAV7
TRAV9−2
TRBC1
TRBJ2−7
TRBV20−1
TRBV20OR9−2
TRBV28
TRBV7−3
TRBV7−9
TRDV1
TREM1
TREML2
TREX2
TRGC1
TRGC2
TRGV10
TRGV2
TRGV3
TRGV5
TRGV8
TRIB1
TRIM10
TRIM11
TRIM15
TRIM16
TRIM2
TRIM21
TRIM28
TRIM3
TRIM4
TRIM47
TRIM55
TRIM61
TRIM7
TRIM8
TRIO
TRIOBP
TRIP6
TRMT1
TRMT10A
TRMT11
TRMT13
TRMT2A
TRO
TROVE2
TRPC1
TRPV3
TRUB2
TSACC
TSC22D3
TSEN15
TSEN34
TSFM
25% (each of the 100 rows)
Mutation Types
missense_variant
frameshift_variant
stop_gained
multi_hit

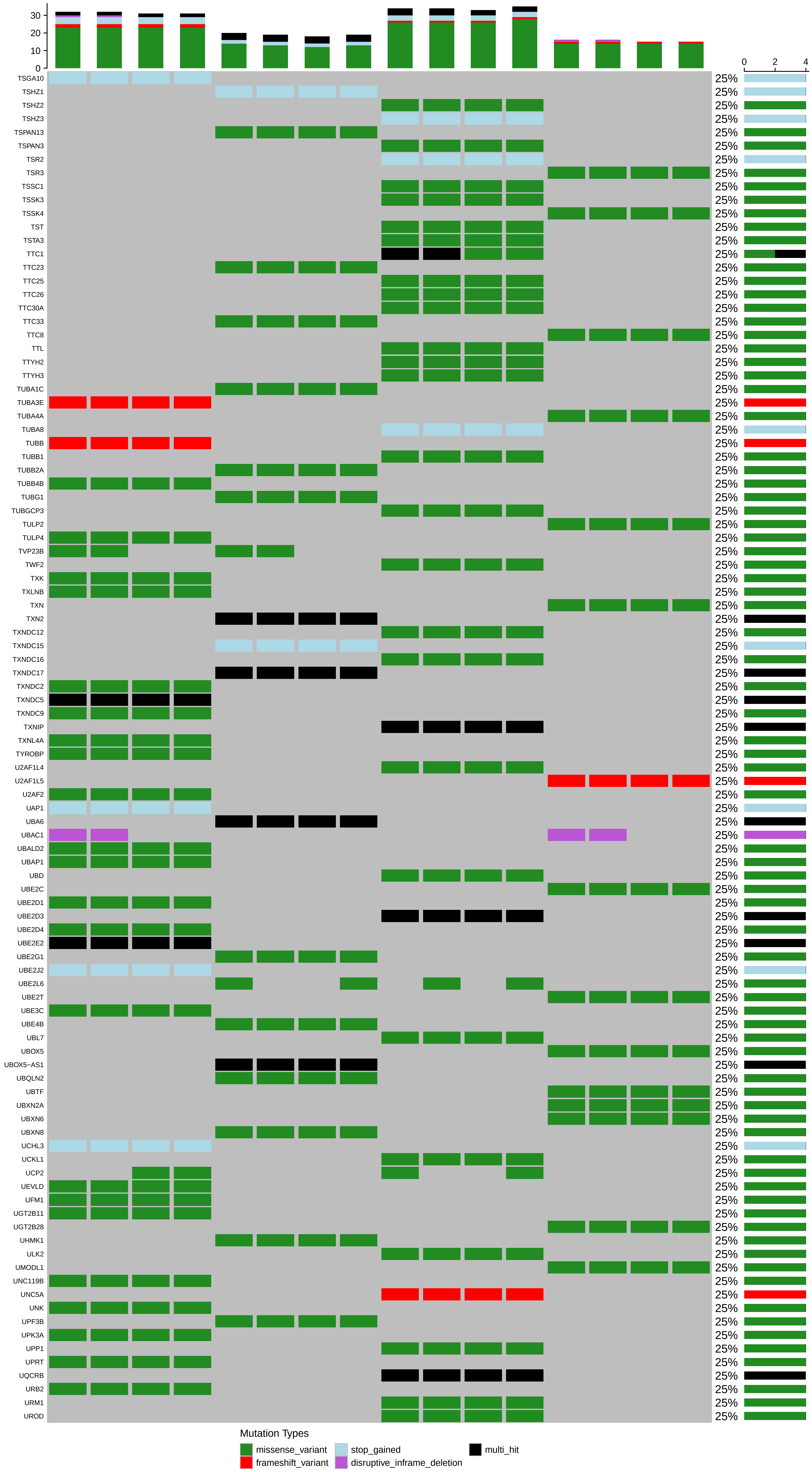
30
20
10
0
0
2
4
TSGA10
TSHZ1
TSHZ2
TSHZ3
TSPAN13
TSPAN3
TSR2
TSR3
TSSC1
TSSK3
TSSK4
TST
TSTA3
TTC1
TTC23
TTC25
TTC26
TTC30A
TTC33
TTC8
TTL
TTYH2
TTYH3
TUBA1C
TUBA3E
TUBA4A
TUBA8
TUBB
TUBB1
TUBB2A
TUBB4B
TUBG1
TUBGCP3
TULP2
TULP4
TVP23B
TWF2
TXK
TXLNB
TXN
TXN2
TXNDC12
TXNDC15
TXNDC16
TXNDC17
TXNDC2
TXNDC5
TXNDC9
TXNIP
TXNL4A
TYROBP
U2AF1L4
U2AF1L5
U2AF2
UAP1
UBA6
UBAC1
UBALD2
UBAP1
UBD
UBE2C
UBE2D1
UBE2D3
UBE2D4
UBE2E2
UBE2G1
UBE2J2
UBE2L6
UBE2T
UBE3C
UBE4B
UBL7
UBOX5
UBOX5-AS1
UBQLN2
UBTF
UBXN2A
UBXN6
UBXN8
UCHL3
UCKL1
UCP2
UEVLD
UFM1
UGT2B11
UGT2B28
UHMK1
ULK2
UMODL1
UNC119B
UNC5A
UNK
UPF3B
UPK3A
UPP1
UPRT
UQCRB
URB2
URM1
UROD
25% (each of the 101 rows above)
Mutation Types
missense_variant
frameshift_variant
stop_gained
disruptive_inframe_deletion
multi_hit

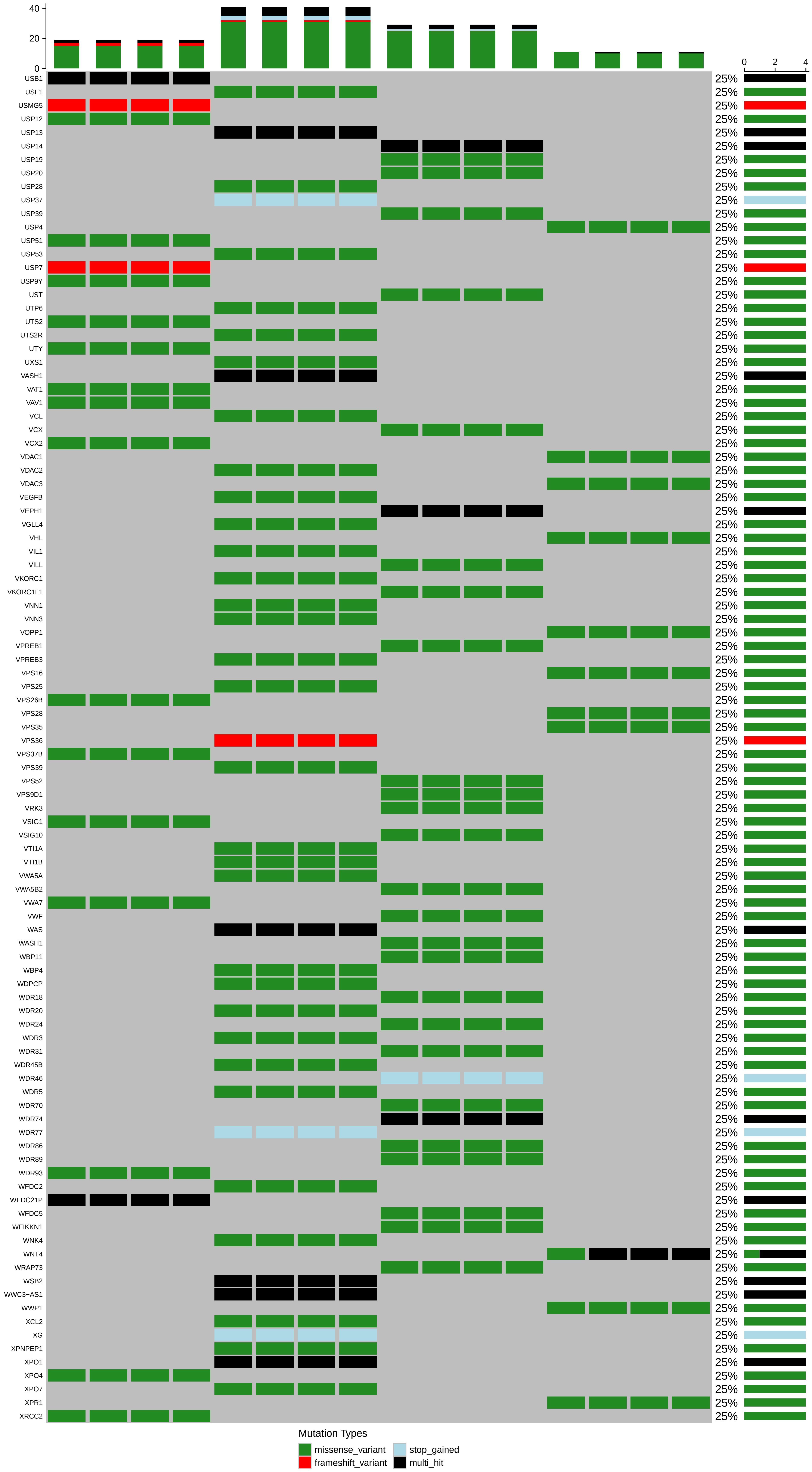

40
20
0
0
2
4
USB1
USF1
USMG5
USP12
USP13
USP14
USP19
USP20
USP28
USP37
USP39
USP4
USP51
USP53
USP7
USP9Y
UST
UTP6
UTS2
UTS2R
UTY
UXS1
VASH1
VAT1
VAV1
VCL
VCX
VCX2
VDAC1
VDAC2
VDAC3
VEGFB
VEPH1
VGLL4
VHL
VIL1
VILL
VKORC1
VKORC1L1
VNN1
VNN3
VOPP1
VPREB1
VPREB3
VPS16
VPS25
VPS26B
VPS28
VPS35
VPS36
VPS37B
VPS39
VPS52
VPS9D1
VRK3
VSIG1
VSIG10
VTI1A
VTI1B
VWA5A
VWA5B2
VWA7
VWF
WAS
WASH1
WBP11
WBP4
WDPCP
WDR18
WDR20
WDR24
WDR3
WDR31
WDR45B
WDR46
WDR5
WDR70
WDR74
WDR77
WDR86
WDR89
WDR93
WFDC2
WFDC21P
WFDC5
WFIKKN1
WNK4
WNT4
WRAP73
WSB2
WWC3−AS1
WWP1
XCL2
XG
XPNPEP1
XPO1
XPO4
XPO7
XPR1
XRCC2
25%
Mutation Types
missense_variant
frameshift_variant
stop_gained
multi_hit

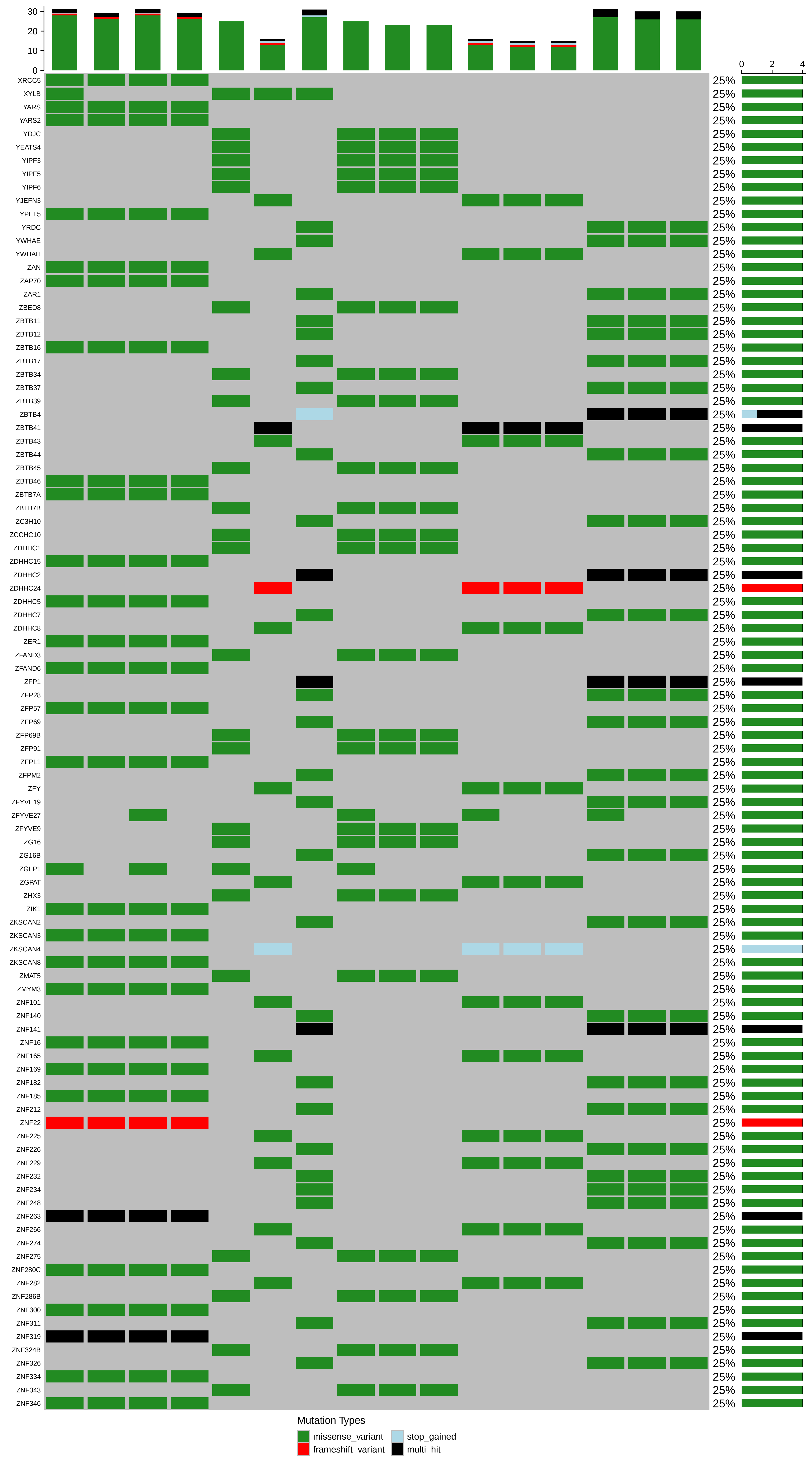

30
20
10
0
0
2
4
XRCC5
XYLB
YARS
YARS2
YDJC
YEATS4
YIPF3
YIPF5
YIPF6
YJEFN3
YPEL5
YRDC
YWHAE
YWHAH
ZAN
ZAP70
ZAR1
ZBED8
ZBTB11
ZBTB12
ZBTB16
ZBTB17
ZBTB34
ZBTB37
ZBTB39
ZBTB4
ZBTB41
ZBTB43
ZBTB44
ZBTB45
ZBTB46
ZBTB7A
ZBTB7B
ZC3H10
ZCCHC10
ZDHHC1
ZDHHC15
ZDHHC2
ZDHHC24
ZDHHC5
ZDHHC7
ZDHHC8
ZER1
ZFAND3
ZFAND6
ZFP1
ZFP28
ZFP57
ZFP69
ZFP69B
ZFP91
ZFPL1
ZFPM2
ZFY
ZFYVE19
ZFYVE27
ZFYVE9
ZG16
ZG16B
ZGLP1
ZGPAT
ZHX3
ZIK1
ZKSCAN2
ZKSCAN3
ZKSCAN4
ZKSCAN8
ZMAT5
ZMYM3
ZNF101
ZNF140
ZNF141
ZNF16
ZNF165
ZNF169
ZNF182
ZNF185
ZNF212
ZNF22
ZNF225
ZNF226
ZNF229
ZNF232
ZNF234
ZNF248
ZNF263
ZNF266
ZNF274
ZNF275
ZNF280C
ZNF282
ZNF286B
ZNF300
ZNF311
ZNF319
ZNF324B
ZNF326
ZNF334
ZNF343
ZNF346
25%
Mutation Types
missense_variant
frameshift_variant
stop_gained
multi_hit

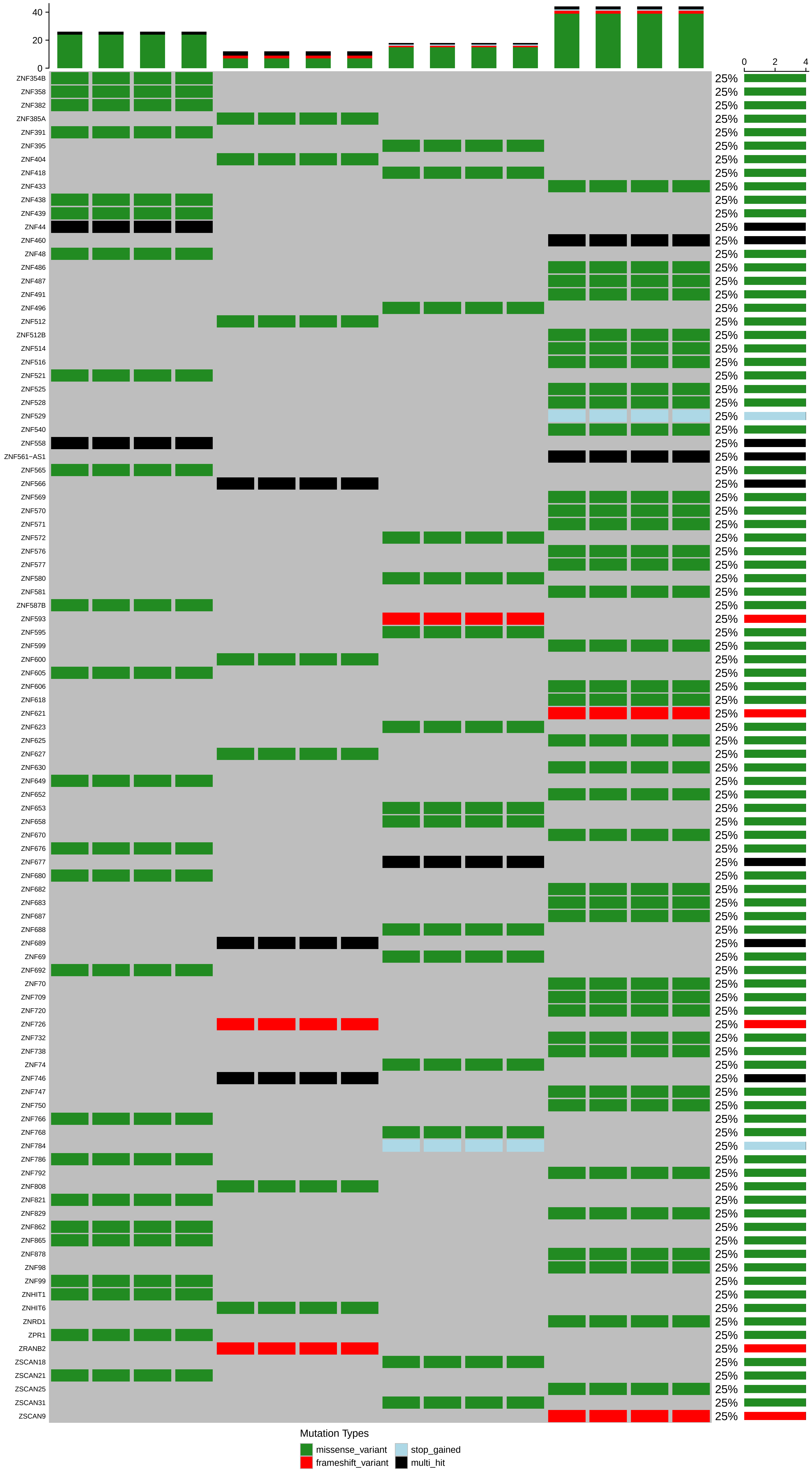
40
20
0
0
2
4
ZNF354B
ZNF358
ZNF382
ZNF385A
ZNF391
ZNF395
ZNF404
ZNF418
ZNF433
ZNF438
ZNF439
ZNF44
ZNF460
ZNF48
ZNF486
ZNF487
ZNF491
ZNF496
ZNF512
ZNF512B
ZNF514
ZNF516
ZNF521
ZNF525
ZNF528
ZNF529
ZNF540
ZNF558
ZNF561−AS1
ZNF565
ZNF566
ZNF569
ZNF570
ZNF571
ZNF572
ZNF576
ZNF577
ZNF580
ZNF581
ZNF587B
ZNF593
ZNF595
ZNF599
ZNF600
ZNF605
ZNF606
ZNF618
ZNF621
ZNF623
ZNF625
ZNF627
ZNF630
ZNF649
ZNF652
ZNF653
ZNF658
ZNF670
ZNF676
ZNF677
ZNF680
ZNF682
ZNF683
ZNF687
ZNF688
ZNF689
ZNF69
ZNF692
ZNF70
ZNF709
ZNF720
ZNF726
ZNF732
ZNF738
ZNF74
ZNF746
ZNF747
ZNF750
ZNF766
ZNF768
ZNF784
ZNF786
ZNF792
ZNF808
ZNF821
ZNF829
ZNF862
ZNF865
ZNF878
ZNF98
ZNF99
ZNHIT1
ZNHIT6
ZNRD1
ZPR1
ZRANB2
ZSCAN18
ZSCAN21
ZSCAN25
ZSCAN31
ZSCAN9
25% (printed beside each gene)
Mutation Types
missense_variant
stop_gained
frameshift_variant
multi_hit

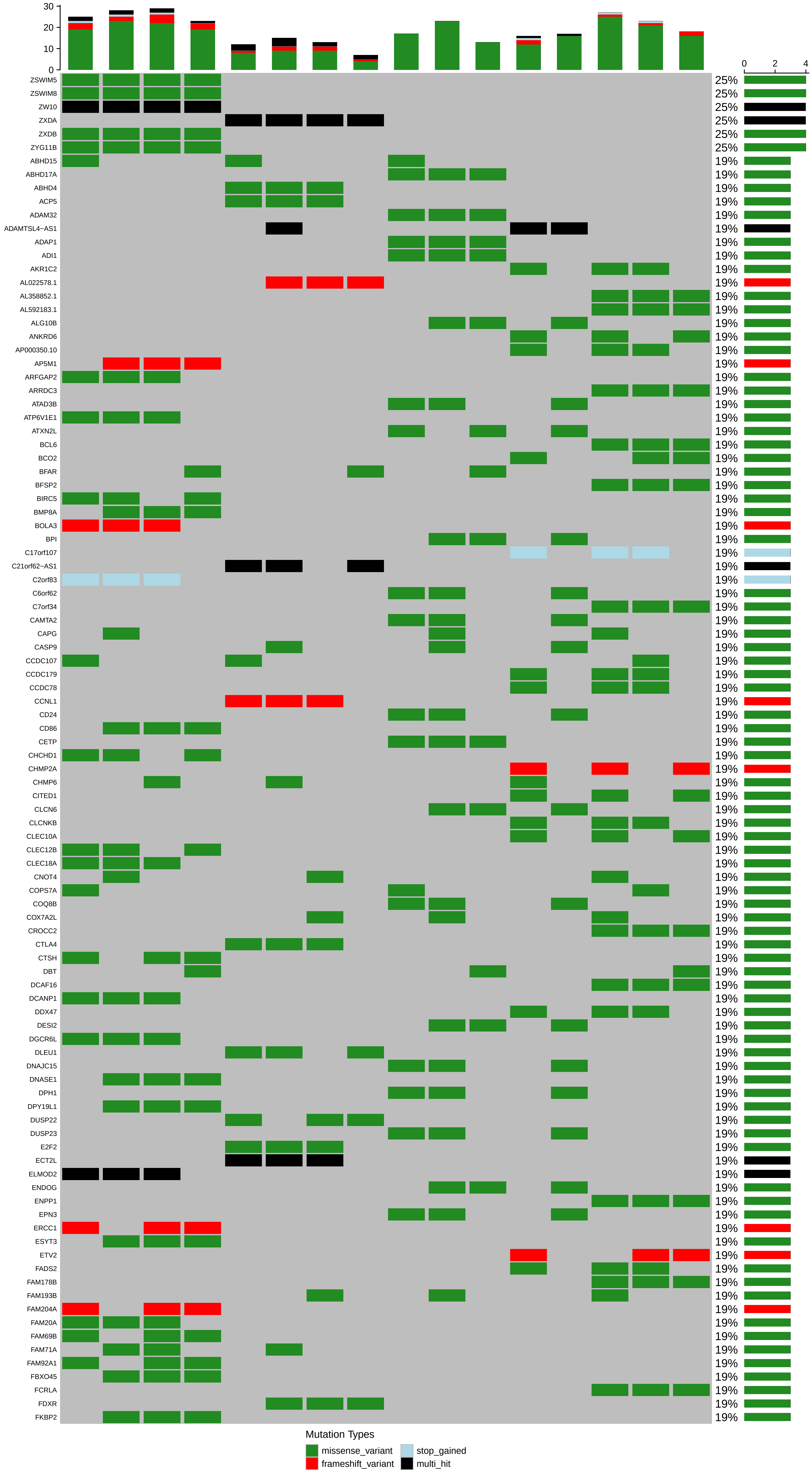

ZSWIM5
ZSWIM8
ZW10
ZXDA
ZXDB
ZYG11B
ABHD15
ABHD17A
ABHD4
ACP5
ADAM32
ADAMTSL4−AS1
ADAP1
ADI1
AKR1C2
AL022578.1
AL358852.1
AL592183.1
ALG10B
ANKRD6
AP000350.10
AP5M1
ARFGAP2
ARRDC3
ATAD3B
ATP6V1E1
ATXN2L
BCL6
BCO2
BFAR
BFSP2
BIRC5
BMP8A
BOLA3
BPI
C17orf107
C21orf62−AS1
C2orf83
C6orf62
C7orf34
CAMTA2
CAPG
CASP9
CCDC107
CCDC179
CCDC78
CCNL1
CD24
CD86
CETP
CHCHD1
CHMP2A
CHMP6
CITED1
CLCN6
CLCNKB
CLEC10A
CLEC12B
CLEC18A
CNOT4
COPS7A
COQ8B
COX7A2L
CROCC2
CTLA4
CTSH
DBT
DCAF16
DCANP1
DDX47
DESI2
DGCR6L
DLEU1
DNAJC15
DNASE1
DPH1
DPY19L1
DUSP22
DUSP23
E2F2
ECT2L
ELMOD2
ENDOG
ENPP1
EPN3
ERCC1
ESYT3
ETV2
FADS2
FAM178B
FAM193B
FAM204A
FAM20A
FAM69B
FAM71A
FAM92A1
FBXO45
FCRLA
FDXR
FKBP2
25%
19%
Mutation Types
missense_variant
frameshift_variant
stop_gained
multi_hit

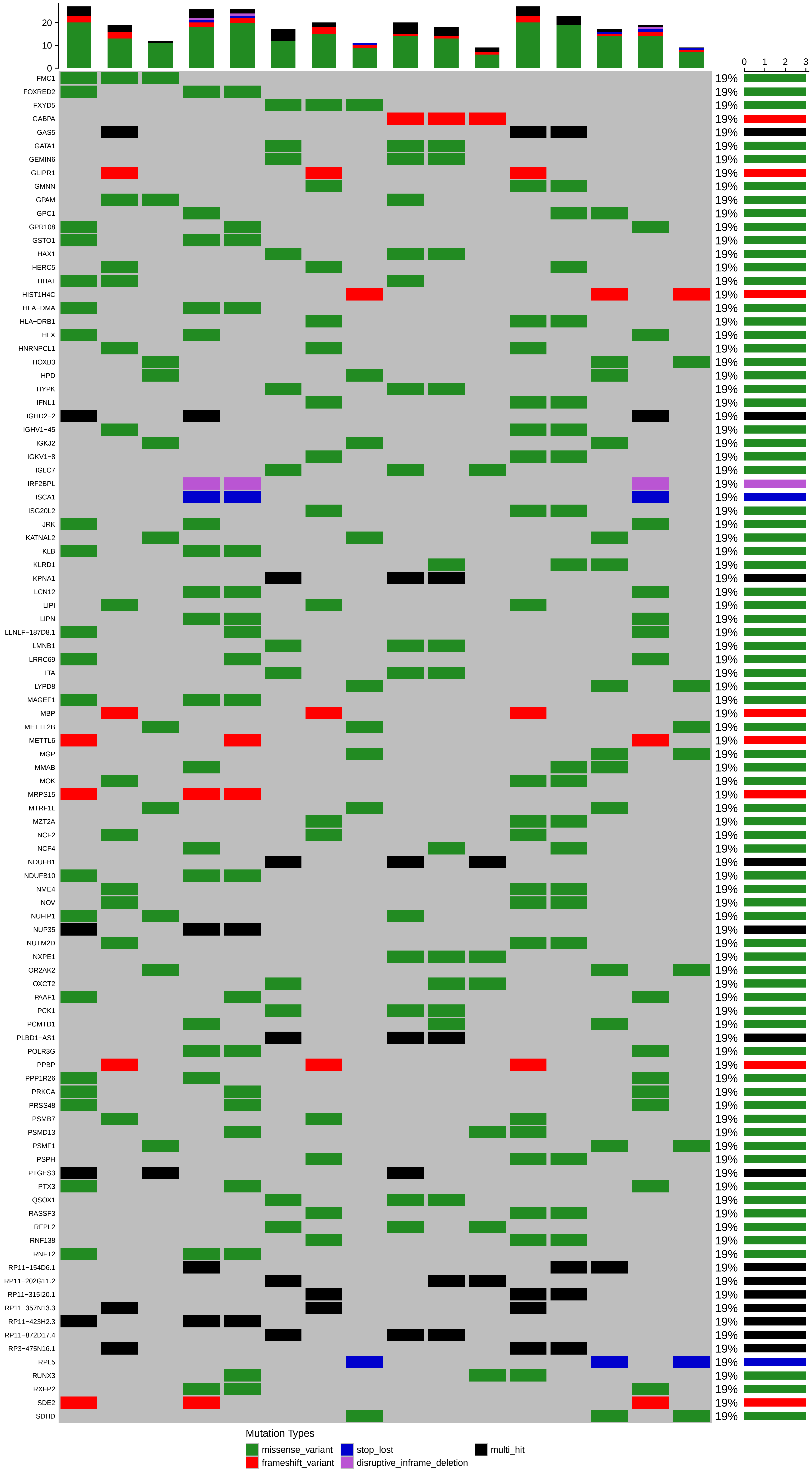

20
10
0
0
1
2
3
FMC1
FOXRED2
FXYD5
GABPA
GAS5
GATA1
GEMIN6
GLIPR1
GMNN
GPAM
GPC1
GPR108
GSTO1
HAX1
HERC5
HHAT
HIST1H4C
HLA−DMA
HLA−DRB1
HLX
HNRNPCL1
HOXB3
HPD
HYPK
IFNL1
IGHD2−2
IGHV1−45
IGKJ2
IGKV1−8
IGLC7
IRF2BPL
ISCA1
ISG20L2
JRK
KATNAL2
KLB
KLRD1
KPNA1
LCN12
LIPI
LIPN
LLNLF−187D8.1
LMNB1
LRRC69
LTA
LYPD8
MAGEF1
MBP
METTL2B
METTL6
MGP
MMAB
MOK
MRPS15
MTRF1L
MZT2A
NCF2
NCF4
NDUFB1
NDUFB10
NME4
NOV
NUFIP1
NUP35
NUTM2D
NXPE1
OR2AK2
OXCT2
PAAF1
PCK1
PCMTD1
PLBD1−AS1
POLR3G
PPBP
PPP1R26
PRKCA
PRSS48
PSMB7
PSMD13
PSMF1
PSPH
PTGES3
PTX3
QSOX1
RASSF3
RFPL2
RNF138
RNFT2
RP11−154D6.1
RP11−202G11.2
RP11−315I20.1
RP11−357N13.3
RP11−423H2.3
RP11−872D17.4
RP3−475N16.1
RPL5
RUNX3
RXFP2
SDE2
SDHD
19%
Mutation Types
missense_variant
frameshift_variant
stop_lost
disruptive_inframe_deletion
multi_hit

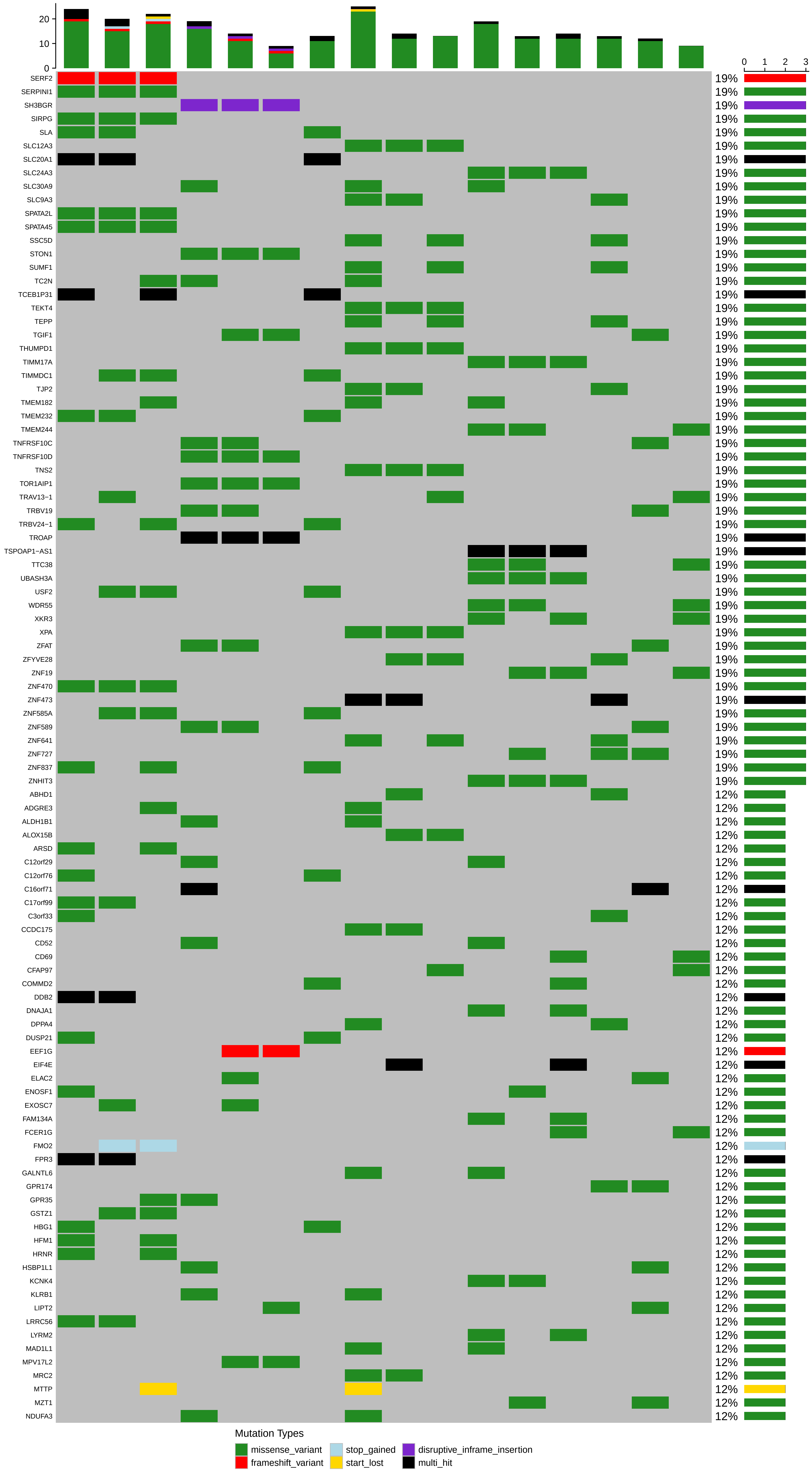

0
10
20
0 1 2 3
SERF2 19%
SERPINI1 19%
SH3BGR 19%
SIRPG 19%
SLA 19%
SLC12A3 19%
SLC20A1 19%
SLC24A3 19%
SLC30A9 19%
SLC9A3 19%
SPATA2L 19%
SPATA45 19%
SSC5D 19%
STON1 19%
SUMF1 19%
TC2N 19%
TCEB1P31 19%
TEKT4 19%
TEPP 19%
TGIF1 19%
THUMPD1 19%
TIMM17A 19%
TIMMDC1 19%
TJP2 19%
TMEM182 19%
TMEM232 19%
TMEM244 19%
TNFRSF10C 19%
TNFRSF10D 19%
TNS2 19%
TOR1AIP1 19%
TRAV13−1 19%
TRBV19 19%
TRBV24−1 19%
TROAP 19%
TSPOAP1−AS1 19%
TTC38 19%
UBASH3A 19%
USF2 19%
WDR55 19%
XKR3 19%
XPA 19%
ZFAT 19%
ZFYVE28 19%
ZNF19 19%
ZNF470 19%
ZNF473 19%
ZNF585A 19%
ZNF589 19%
ZNF641 19%
ZNF727 19%
ZNF837 19%
ZNHIT3 19%
ABHD1 12%
ADGRE3 12%
ALDH1B1 12%
ALOX15B 12%
ARSD 12%
C12orf29 12%
C12orf76 12%
C16orf71 12%
C17orf99 12%
C3orf33 12%
CCDC175 12%
CD52 12%
CD69 12%
CFAP97 12%
COMMD2 12%
DDB2 12%
DNAJA1 12%
DPPA4 12%
DUSP21 12%
EEF1G 12%
EIF4E 12%
ELAC2 12%
ENOSF1 12%
EXOSC7 12%
FAM134A 12%
FCER1G 12%
FMO2 12%
FPR3 12%
GALNTL6 12%
GPR174 12%
GPR35 12%
GSTZ1 12%
HBG1 12%
HFM1 12%
HRNR 12%
HSBP1L1 12%
KCNK4 12%
KLRB1 12%
LIPT2 12%
LRRC56 12%
LYRM2 12%
MAD1L1 12%
MPV17L2 12%
MRC2 12%
MTTP 12%
MZT1 12%
NDUFA3 12%
Mutation Types
missense_variant
stop_gained
disruptive_inframe_insertion
frameshift_variant
start_lost
multi_hit

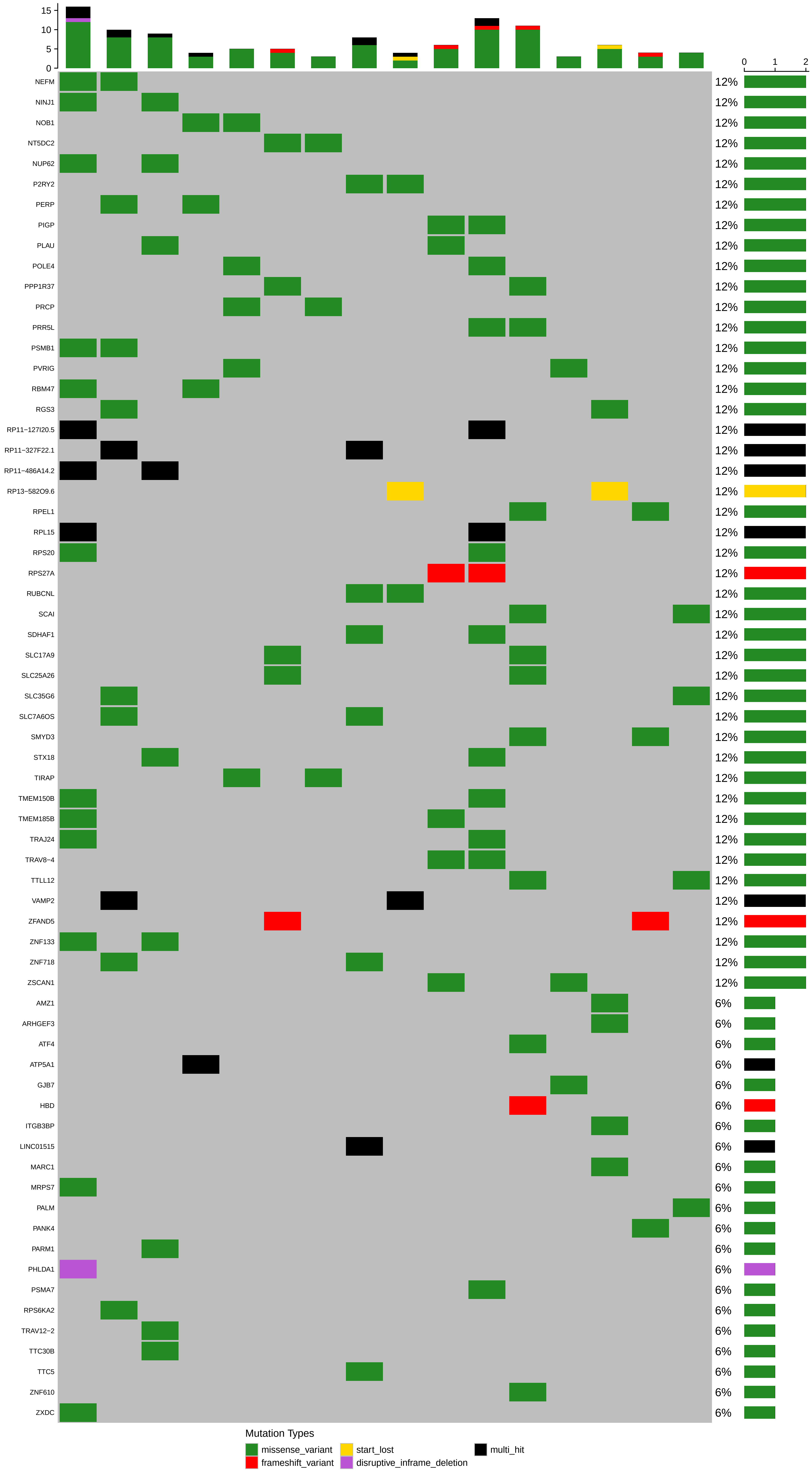

NEFM 12%
NINJ1 12%
NOB1 12%
NT5DC2 12%
NUP62 12%
P2RY2 12%
PERP 12%
PIGP 12%
PLAU 12%
POLE4 12%
PPP1R37 12%
PRCP 12%
PRR5L 12%
PSMB1 12%
PVRIG 12%
RBM47 12%
RGS3 12%
RP11−127I20.5 12%
RP11−327F22.1 12%
RP11−486A14.2 12%
RP13−582O9.6 12%
RPEL1 12%
RPL15 12%
RPS20 12%
RPS27A 12%
RUBCNL 12%
SCAI 12%
SDHAF1 12%
SLC17A9 12%
SLC25A26 12%
SLC35G6 12%
SLC7A6OS 12%
SMYD3 12%
STX18 12%
TIRAP 12%
TMEM150B 12%
TMEM185B 12%
TRAJ24 12%
TRAV8−4 12%
TTLL12 12%
VAMP2 12%
ZFAND5 12%
ZNF133 12%
ZNF718 12%
ZSCAN1 12%
AMZ1 6%
ARHGEF3 6%
ATF4 6%
ATP5A1 6%
GJB7 6%
HBD 6%
ITGB3BP 6%
LINC01515 6%
MARC1 6%
MRPS7 6%
PALM 6%
PANK4 6%
PARM1 6%
PHLDA1 6%
PSMA7 6%
RPS6KA2 6%
TRAV12−2 6%
TTC30B 6%
TTC5 6%
ZNF610 6%
ZXDC 6%
Mutation Types
missense_variant
frameshift_variant
start_lost
disruptive_inframe_deletion
multi_hit

Supplementary Figure S2.

Oncoprint of somatic mutations identified in every Multiple Myeloma patient−control comparison. Mutation types are color−coded; Each box from left to right represents each comparison. The bar plot above indicates the number of mutations per comparison, while the percentage on the right shows the frequency of each gene mutation.
